# Supplementary figures and images for: Deconvolution of intergenic polymorphisms determining high expression of Factor H binding protein in meningococcus and their association with invasive disease
Source: PLoS Pathog. 2021 Mar 26;17(3):e1009461. doi: 10.1371/journal.ppat.1009461 (PMC8026042; doi:10.1371/journal.ppat.1009461)

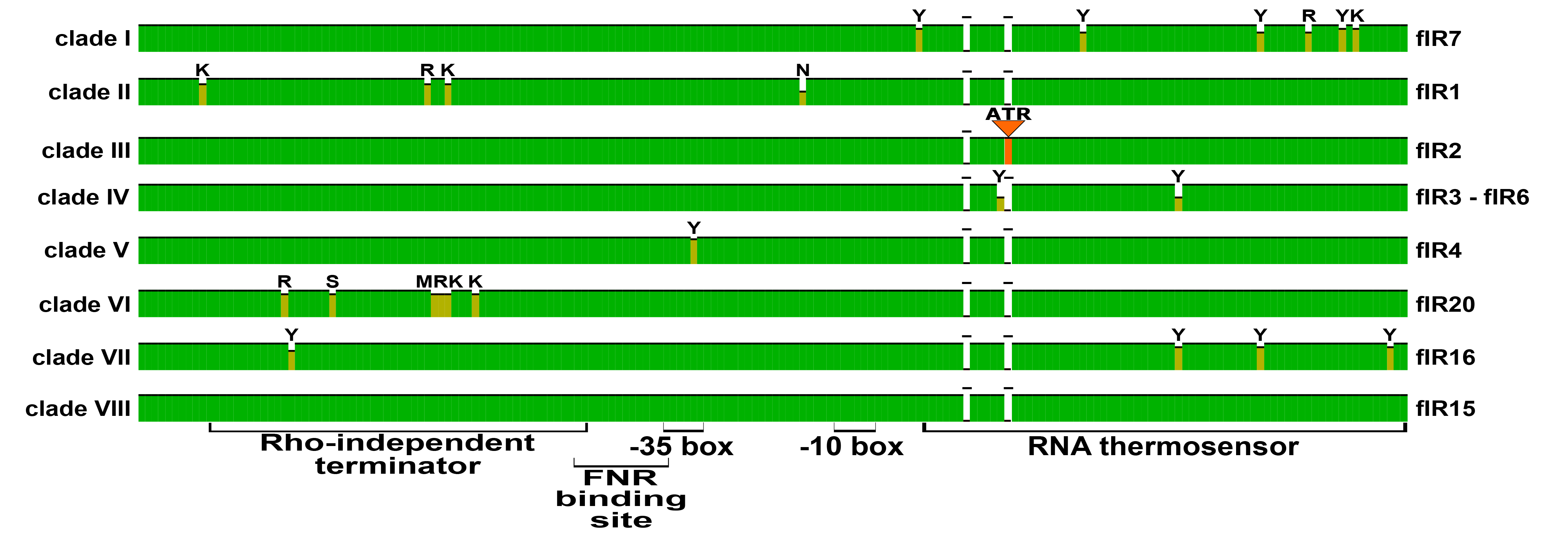

Supplement: S1 Fig — Within each clade the most representative fIR sequence is indicated on the right. Green areas indicate nucleotides with 100% identity with the most representative fIR allele for the clade, and variable regions are indicated in yellow. Polymorphisms are indicated as Y (pyrimidine, C or T), R (purine, A or G), K (keto, G or T), M (amino, A or C), S (strong, C or G), and N (any nucleotide). Gaps are indicated with “-”. At position +128 of the alignment the presence of the ATR insertion element (181 bp sequence, not to scale) in clade III is indicated. (TIF) [file ppat.1009461.s001.tif]

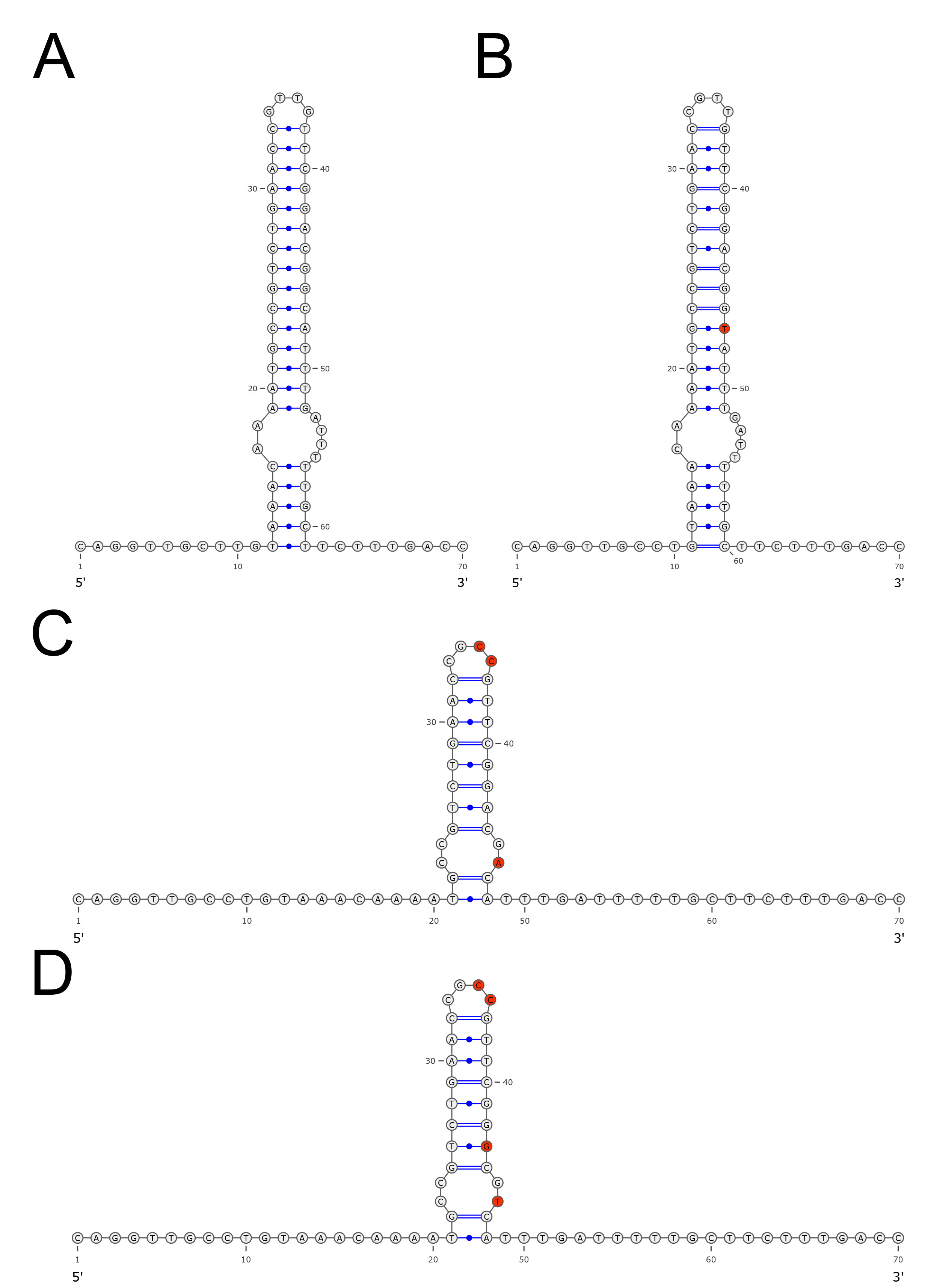

Supplement: S2 Fig — The structures of the terminators with ΔG = -27.3 kcal/mol (A), -24.7 kcal/mol (B), -14.8 kcal/mol (C) and -13.0 kcal/mol (D) are represented. SNPs from the strong terminator sequence are highlighted in orange. (TIF) [file ppat.1009461.s002.tif]

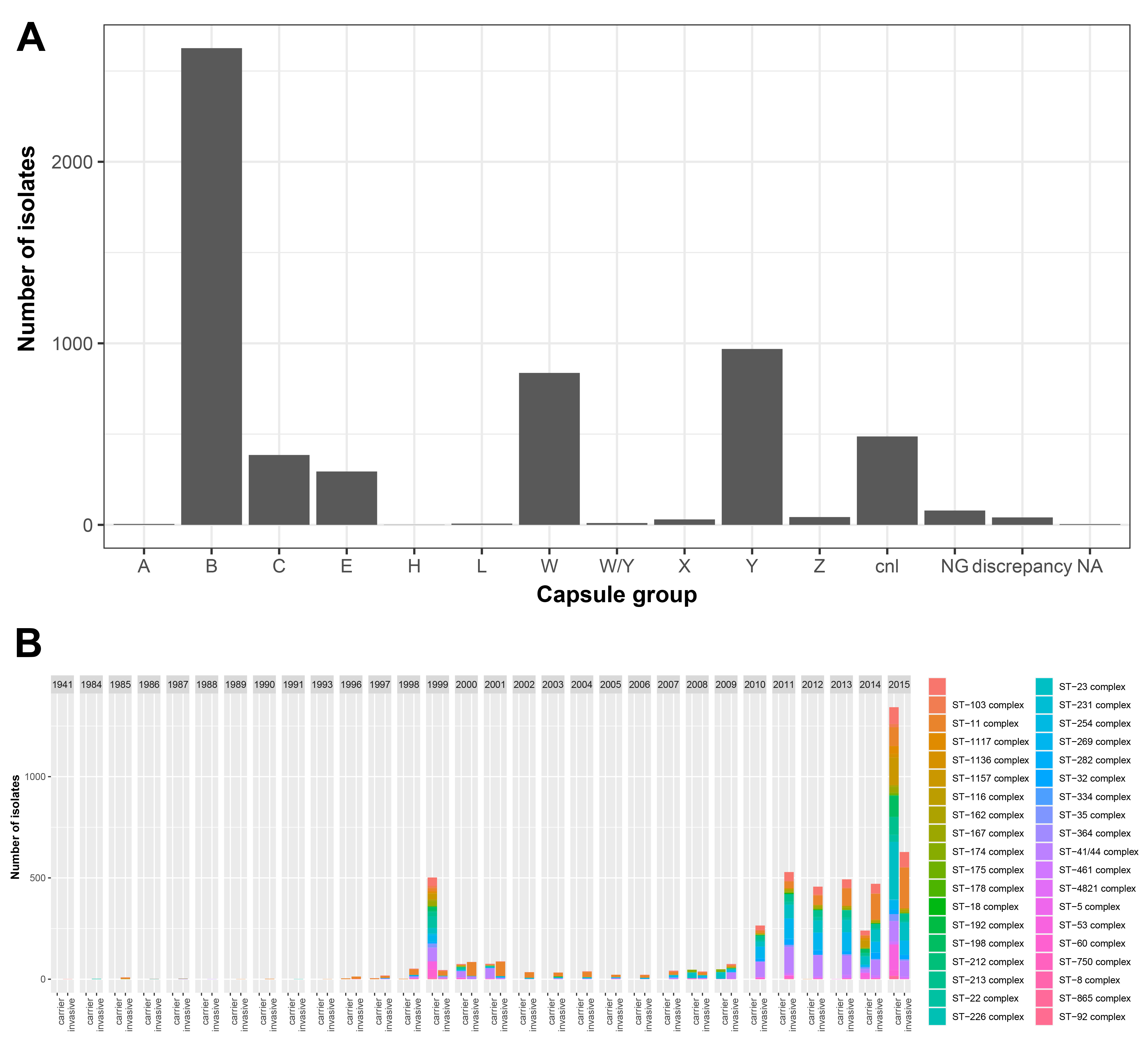

Supplement: S3 Fig — The heights of the bars refer to the number of strains carrying each capsule group (A) and clonal complex (B) in the public collection. NA: Not Available; NG: Not Groupable; cnl: capsule null; Various singlets: all strains that have not been assigned to a clonal complex. (TIF) [file ppat.1009461.s003.tif]

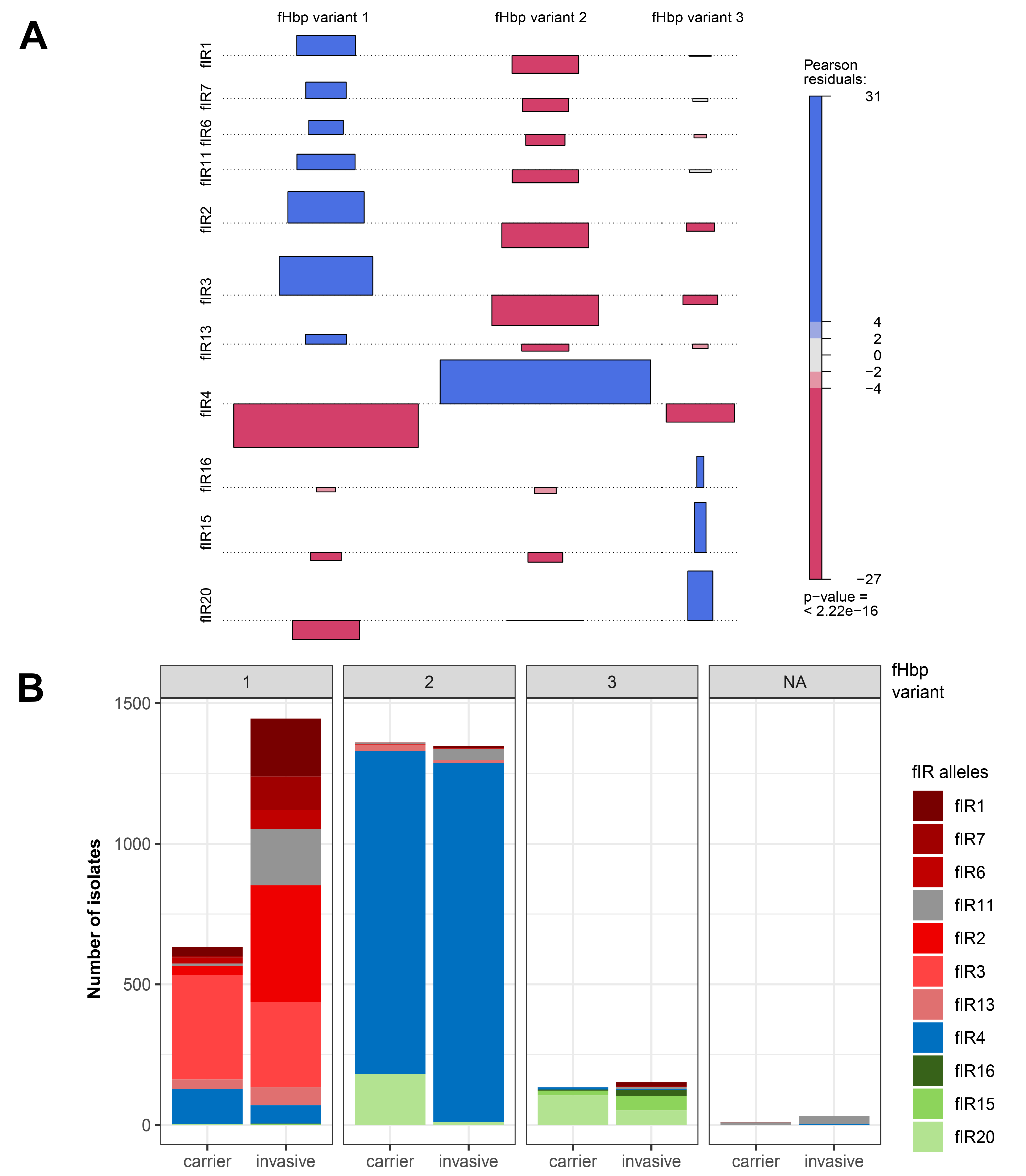

Supplement: S4 Fig — (A) In the plot are reported the Pearson’s residuals for the 11 most common fIRs in relation to fHbp variants. Statistics have been computed as described in the Materials and methods section. As reported in the legends on the right side, the cells are colored in blue when the number for that combination of alleles is higher than expected by the null hypothesis (that envisions independence), red is used when the number is lower than expected. The darker the color, the bigger the importance of that combination in the p-value determination. The height of each bar is proportional to the (signed) residual and the width is proportional to the square root of the expected counts, so that the area of the box is proportional to the difference in observed and expected frequencies. (B) Histograms representing number of isolates of each fIR alleles, divided by carrier or invasive disease, in strains harboring fHbp variant 1, variant 2 or variant 3. (TIF) [file ppat.1009461.s004.tif]

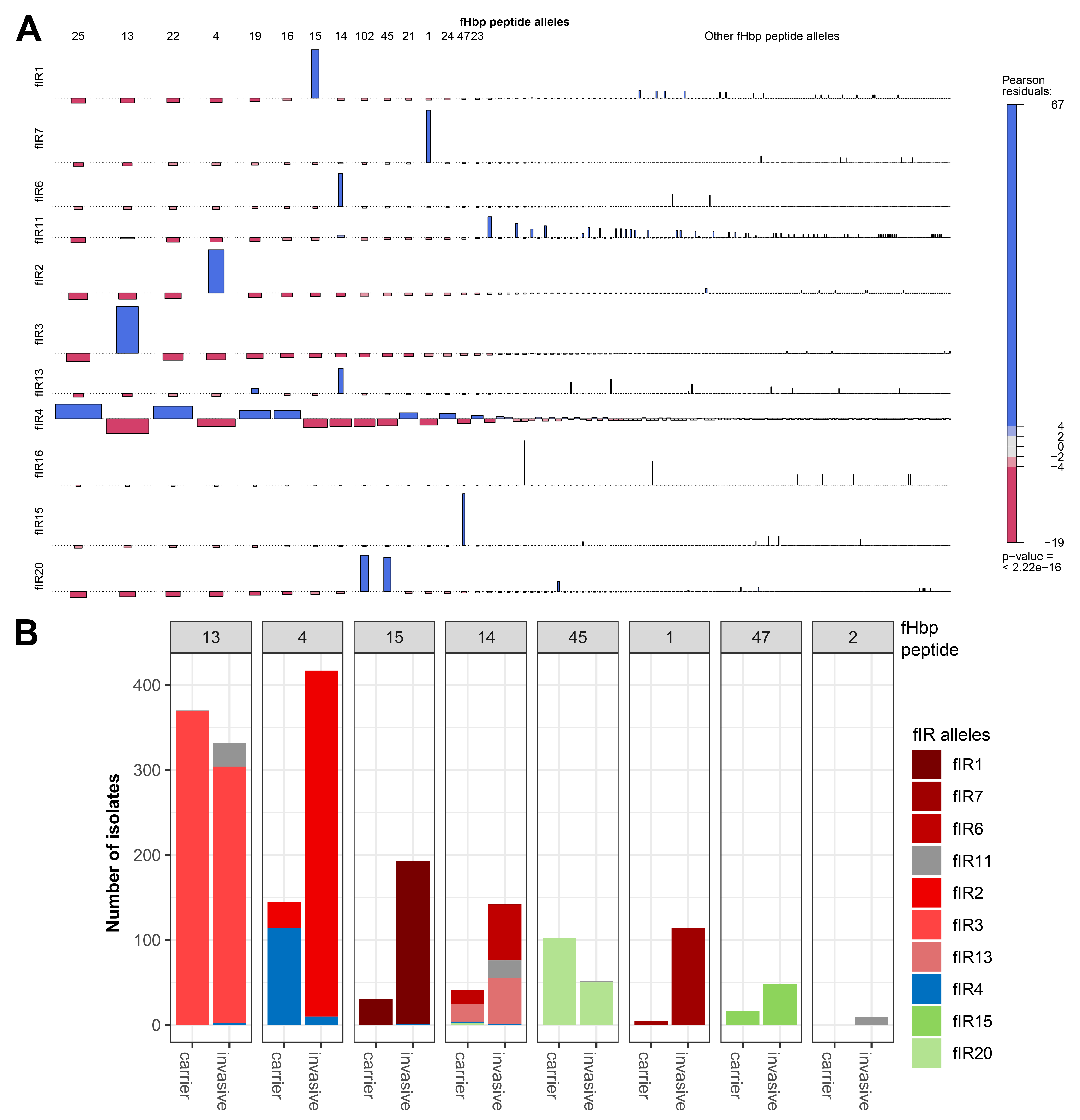

Supplement: S5 Fig — (A) In the plot are reported the Pearson’s residuals for the 11 most common fIRs in relation to fHbp peptides. Statistics have been computed as described in the Materials and methods section. As reported in the legends on the right side, the cells are colored in blue when the number for that combination of alleles is higher than expected by the null hypothesis (that envisions independence), red is used when the number is lower than expected. The darker the color, the bigger the importance of that combination in the p-value determination. The height of each bar is proportional to the (signed) residual and the width is proportional to the square root of the expected counts, so that the area of the box is proportional to the difference in observed and expected frequencies. (B) Histograms representing number of isolates of each fIR alleles, divided by carrier or invasive disease, in strains harboring fHbp peptides 1, 2, 4, 13, 14, 15, 45 or 47. (TIF) [file ppat.1009461.s005.tif]

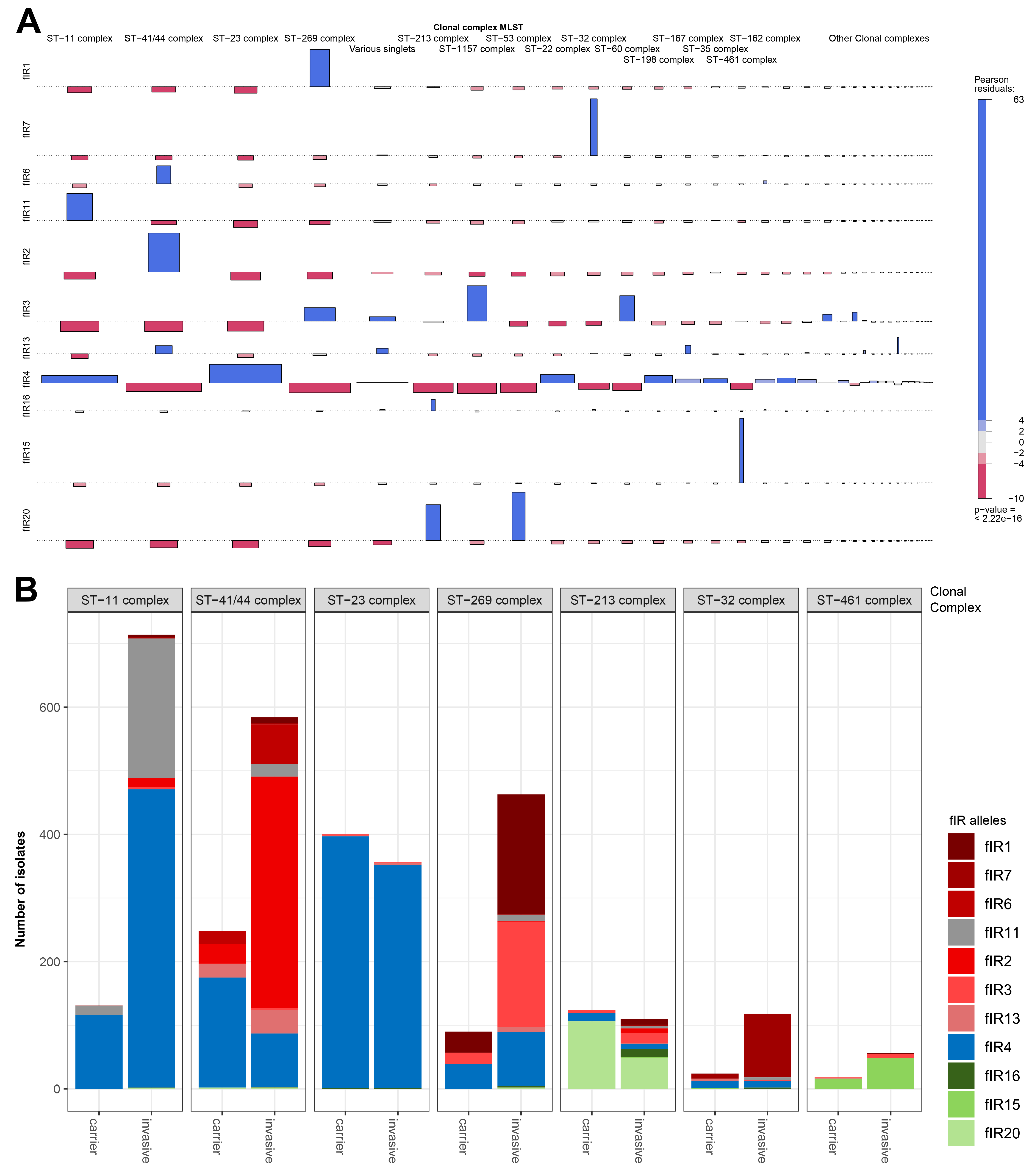

Supplement: S6 Fig — (A) In the plot are reported the Pearson’s residuals for the 11 most common fIRs in relation to clonal complexes. Statistics have been computed as described in the Materials and methods section. As reported in the legends on the right side, the cells are colored in blue when the number for that combination of alleles is higher than expected by the null hypothesis (that envisions independence), red is used when the number is lower than expected. The darker the color, the bigger the importance of that combination in the p-value determination. The height of each bar is proportional to the (signed) residual and the width is proportional to the square root of the expected counts, so that the area of the box is proportional to the difference in observed and expected frequencies. (B) Histograms representing number of isolates of each fIR alleles, divided by carrier or invasive disease, in strains of clonal complexes ST-11, ST-23, ST-32, ST-41/44, ST-213, ST-269 or ST-461. (TIF) [file ppat.1009461.s006.tif]

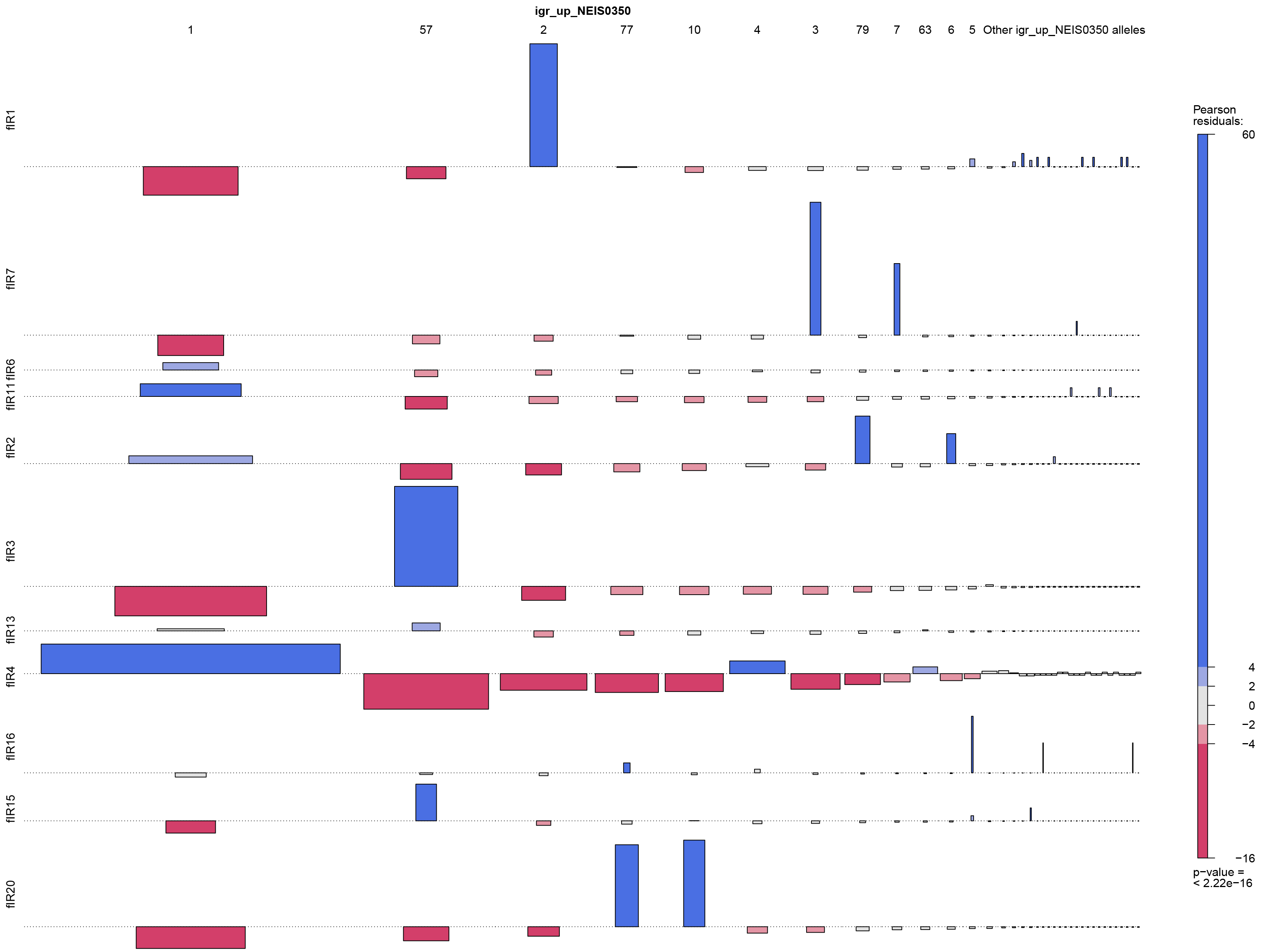

Supplement: S7 Fig — In the plot are reported the Pearson’s residuals for the 11 most common fIRs in relation to cbbA promoters. Statistics have been computed as described in the Materials and methods section. As reported in the legends on the right side, the cells are colored in blue when the number for that combination of alleles is higher than expected by the null hypothesis (that envisions independence), red is used when the number is lower than expected. The darker the color, the bigger the importance of that combination in the p-value determination. The height of each bar is proportional to the (signed) residual and the width is proportional to the square root of the expected counts, so that the area of the box is proportional to the difference in observed and expected frequencies. (TIF) [file ppat.1009461.s007.tif]
