# Supplementary material for: Deconvolution of intergenic polymorphisms determining high expression of Factor H binding protein in meningococcus and their association with invasive disease
Source: PLoS Pathog. 2021 Mar 26;17(3):e1009461. doi: 10.1371/journal.ppat.1009461 (PMC8026042; doi:10.1371/journal.ppat.1009461)
Supplement: S1 Table — Median, first quartile and third quartile of fHbp amounts plotted according to either the clades or the fIR sequence alleles were extracted from the two box plots. IQR, interquartile range, is calculated as “third—first quartiles” and is an indication of the variability of the set of data. The larger the IQR, the more variable the data set is. (DOCX) [file ppat.1009461.s008.docx]

S1 Table. Values calculated from the box plots in Biagini *et al*. [26] and in Fig 1B.

|  | **Median** | **First quartile** | **Third quartile** | **IQR** |
| --- | --- | --- | --- | --- |
| clade I | 411,93 | 340,22 | 521,16 | 180,94 |
| fIR7 | 459,30 | 326,66 | 564,97 | 238,31 |
| clade II | 608,73 | 440,92 | 726,70 | 285,78 |
| fIR1 | 704,31 | 547,13 | 940,56 | 393,44 |
| clade III | 258,80 | 159,28 | 324,49 | 165,21 |
| fIR2 | 258,80 | 159,28 | 324,49 | 165,21 |
| clade IV | 237,13 | 152,21 | 326,85 | 174,63 |
| fIR6 | 324,87 | 293,01 | 375,86 | 82,85 |
| fIR3 | 166,53 | 128,41 | 197,68 | 69,27 |
| clade V | 156,17 | 135,45 | 198,16 | 62,71 |
| fIR4 | 155,94 | 135,55 | 194,55 | 58,99 |
| clade VI | 113,57 | 108,00 | 138,58 | 30,58 |
| fIR20 | 108,00 | 108,00 | 108,00 | 0,00 |
| clade VII | 148,64 | 120,41 | 243,84 | 123,43 |
| fIR16 | 137,72 | 108,00 | 154,63 | 46,63 |
| clade VIII | 136,92 | 108,00 | 215,54 | 107,54 |
| fIR15 | 136,92 | 108,00 | 215,54 | 107,54 |

Median, first quartile and third quartile of fHbp amounts plotted according to either the clades or the fIR sequence alleles were extracted from the two box plots. IQR, interquartile range, is calculated as “third - first quartiles” and is an indication of the variability of the set of data. The larger the IQR, the more variable the data set is.
