# Supplementary material for: Deconvolution of intergenic polymorphisms determining high expression of Factor H binding protein in meningococcus and their association with invasive disease
Source: PLoS Pathog. 2021 Mar 26;17(3):e1009461. doi: 10.1371/journal.ppat.1009461 (PMC8026042; doi:10.1371/journal.ppat.1009461)
Supplement: S2 Table — For each of the fIR alleles the characteristics are listed. Definitions of “weak”, “medium” or “strong” depend on the free energy prediction of the Rho-independent terminator. The numbers are referred to the position within the multiple sequence alignment (Fig 2E). ATR indicates the presence of the AT-rich insertion element. The sequence of the ribosome binding site (RBS) is underlined. (DOCX) [file ppat.1009461.s009.docx]

**S2 Table. Polymorphisms that distinguish the 11 fIR alleles identified.**

| **fIR allele** | **Rho-independent terminator strength** | **+85** | **+86** | **+87** | **+90** | **-10 box** | **+113** | **+115** | **+120** | **+126** | **ATR** | **+137** | **RBS** | **+163** | **+170** | **+178** | **+182** |
| --- | --- | --- | --- | --- | --- | --- | --- | --- | --- | --- | --- | --- | --- | --- | --- | --- | --- |
| fIR1 | weak | A | G | T | G | TACCGC | A | C | A | T |  | T | AGGAGT | C | A | C | C |
| fIR7 | weak | G | G | T | G | TACCAT | A | T | A | T |  | C | AGGAGT | T | G | C | C |
| fIR6 | strong | A | G | T | G | TACCAT | A | T | A | C |  | C | AGGAGC | C | G | C | T |
| fIR11 | strong | G | G | T | A | TACCAT | G | T | T | T |  | C | AGGAGT | T | G | C | C |
| fIR2 | strong | A | G | T | G | TACCAT | A | T | A | T | ATR | C | AGGAGC | C | G | C | C |
| fIR3 | strong | A | G | T | G | TACCAT | A | T | A | T |  | C | AGGAGT | C | G | C | T |
| fIR13 | strong | A | G | T | G | TACCAT | A | T | A | T |  | C | AGGAGC | C | G | C | T |
| fIR4 | strong | A | G | T | G | TACCAT | A | T | A | T |  | C | AGGAGC | C | G | C | C |
| fIR16 | medium | A | A | T | G | TACCGC | A | C | A | T |  | T | AGGAGT | C | G | C | C |
| fIR15 | strong | A | G | T | G | TACCAT | A | T | A | T |  | C | AGGAGC | C | A | T | C |
| fIR20 | strong | A | G | C | G | TACCGC | A | C | A | T |  | T | AGGAGT | C | G | C | C |

For each of the fIR alleles the characteristics are listed. Definitions of “weak”, “medium” or “strong” depend on the free energy prediction of the Rho-independent terminator. The numbers are referred to the position within the multiple sequence alignment (Fig 2E). ATR indicates the presence of the AT-rich insertion element. The sequence of the ribosome binding site (RBS) is underlined.
