# Supplementary material for: Deconvolution of intergenic polymorphisms determining high expression of Factor H binding protein in meningococcus and their association with invasive disease
Source: PLoS Pathog. 2021 Mar 26;17(3):e1009461. doi: 10.1371/journal.ppat.1009461 (PMC8026042; doi:10.1371/journal.ppat.1009461)
Supplement: S3 Table — EryR, erythromycin resistance; CmR, chloramphenicol resistance. (DOCX) [file ppat.1009461.s010.docx]

**S3 Table. List of strains used in this study.**

| **Name** | **Description** | **Antibiotic resistance** | **Reference** |
| --- | --- | --- | --- |
| MC58 | *Neisseria meningitidis* laboratory-adapted reference strain |  | Tettelin *et al*., [71] |
| MC58 Δ*fHbp* | MC58 derivative, lacking fHbp gene | EryR | Masignani *et al*., [24] |
| MC58 c-*fHbp* (fIR7) | MC58 derivative, complemented *in locus* with the wt *fHbp* gene and its upstream intergenic region (fIR7) | CmR | This study |
| MC58 c-*fHbp* fIR1 | MC58 derivative, complemented *in locus* with the wt *fHbp* gene and the upstream fIR1 intergenic region allele | CmR | This study |
| MC58 c-*fHbp* fIR2 | MC58 derivative, complemented *in locus* with the wt *fHbp* gene and the upstream fIR2 intergenic region allele | CmR | This study |
| MC58 c-*fHbp* fIR3 | MC58 derivative, complemented *in locus* with the wt *fHbp* gene and the upstream fIR3 intergenic region allele | CmR | This study |
| MC58 c-*fHbp* fIR4 | MC58 derivative, complemented *in locus* with the wt *fHbp* gene and the upstream fIR4 intergenic region allele | CmR | This study |
| MC58 c-*fHbp* fIR6 | MC58 derivative, complemented *in locus* with the wt *fHbp* gene and the upstream fIR6 intergenic region allele | CmR | This study |
| MC58 c-*fHbp* fIR11 | MC58 derivative, complemented *in locus* with the wt *fHbp* gene and the upstream fIR11 intergenic region allele | CmR | This study |
| MC58 c-*fHbp* fIR13 | MC58 derivative, complemented *in locus* with the wt *fHbp* gene and the upstream fIR13 intergenic region allele | CmR | This study |
| MC58 c-*fHbp* fIR15 | MC58 derivative, complemented *in locus* with the wt *fHbp* gene and the upstream fIR15 intergenic region allele | CmR | This study |
| MC58 c-*fHbp* fIR16 | MC58 derivative, complemented *in locus* with the wt *fHbp* gene and the upstream fIR16 intergenic region allele | CmR | This study |
| MC58 c-*fHbp* fIR20 | MC58 derivative, complemented *in locus* with the wt *fHbp* gene and the upstream fIR20 intergenic region allele | CmR | This study |
| MC58 Δ*fHbp* c-*fHbp* var1.1 | MC58 derivative, lacking *fHbp* gene, with a copy of *fHbp* var1.1 reintroduced out of locus under the control of an IPTG-inducible P_TAC_ promoter | CmR | Biagini *et al.*, [26] |
| MC58 Δ*fHbp* c-*fHbp* var1.14 | MC58 derivative, lacking *fHbp* gene, with a copy of *fHbp* var1.14 reintroduced out of locus under the control of an IPTG-inducible P_TAC_ promoter | CmR | This study |
| MC58 Δ*fHbp* c-*fHbp* var2.16 | MC58 derivative, lacking *fHbp* gene, with a copy of *fHbp* var2.16 reintroduced out of locus under the control of an IPTG-inducible P_TAC_ promoter | CmR | This study |
| MC58 Δ*fHbp* c-*fHbp* var2.21 | MC58 derivative, lacking *fHbp* gene, with a copy of *fHbp* var2.21 reintroduced out of locus under the control of an IPTG-inducible P_TAC_ promoter | CmR | This study |
| MC58 Δ*fHbp* c-*fHbp* var2.25 | MC58 derivative, lacking *fHbp* gene, with a copy of *fHbp* var2.25 reintroduced out of locus under the control of an IPTG-inducible P_TAC_ promoter | CmR | This study |
| MC58 Δ*fHbp* c-*fHbp* var3.28 | MC58 derivative, lacking *fHbp* gene, with a copy of *fHbp* var3.28 reintroduced out of locus under the control of an IPTG-inducible P_TAC_ promoter | CmR | This study |
| MC58 Δ*fHbp* c-*fHbp* var3.45 | MC58 derivative, lacking *fHbp* gene, with a copy of *fHbp* var3.45 reintroduced out of locus under the control of an IPTG-inducible P_TAC_ promoter | CmR | This study |
| MC58 Δ*fHbp* c-*fHbp* var3.47 | MC58 derivative, lacking *fHbp* gene, with a copy of *fHbp* var3.47 reintroduced out of locus under the control of an IPTG-inducible P_TAC_ promoter | CmR | This study |
| MC58 c-*fHbp* -10 box | MC58 derivative, complemented *in locus* with the wt *fHbp* gene and its upstream intergenic region (fIR7) mutated in the -10 box: TACCGC | CmR | This study |
| MC58 c-*fHbp* term | MC58 derivative, complemented *in locus* with the wt *fHbp* gene and its upstream intergenic region (fIR7) mutated in the terminator: strong | CmR | This study |
| MC58 c-*fHbp* term, -10 box | MC58 derivative, complemented *in locus* with the wt *fHbp* gene and its upstream intergenic region (fIR7) mutated in the terminator: strong; and in the -10 box: TACCGC | CmR | This study |
| MC58 c-*fHbp* term, spacer1 | MC58 derivative, complemented *in locus* with the wt *fHbp* gene and its upstream intergenic region (fIR7) mutated in the terminator: strong; spacer: CAGTATGCAAAAAAAGA | CmR | This study |
| MC58 c-*fHbp* term, spacer2 | MC58 derivative, complemented *in locus* with the wt *fHbp* gene and its upstream intergenic region (fIR7) mutated in the terminator: strong; spacer: CAATATGCAAAAAAAGA | CmR | This study |
| MC58 c-*fHbp* term, spacer3 | MC58 derivative, complemented *in locus* with the wt *fHbp* gene and its upstream intergenic region (fIR7) mutated in the terminator: strong; spacer: CAGCATGCAAAAAAAGA | CmR | This study |
| MC58 c-*fHbp* term, -35 box | MC58 derivative, complemented *in locus* with the wt *fHbp* gene and its upstream intergenic region (fIR7) mutated in the terminator: strong; and in the -35 box: TTGACG | CmR | This study |

EryR, erythromycin resistance; CmR, chloramphenicol resistance.
