# Supplementary material for: Deconvolution of intergenic polymorphisms determining high expression of Factor H binding protein in meningococcus and their association with invasive disease
Source: PLoS Pathog. 2021 Mar 26;17(3):e1009461. doi: 10.1371/journal.ppat.1009461 (PMC8026042; doi:10.1371/journal.ppat.1009461)
Supplement: S4 Table — (DOCX) [file ppat.1009461.s011.docx]

**S4 Table. List of primers used in this study.**

| **Name** | **Sequence^a^** | **Application** | **Reference** |
| --- | --- | --- | --- |
| TACCAC_TACCGC-F | GCGGTATGCAAAAAAAGATACCGCAACCAAAATGTTTATATATTATC | mutate box -10 TACCGC (Fw) | This study |
| TACCAC_TACCGC-R | GATAATATATAAACATTTTGGTTGCGGTATCTTTTTTTGCATACCGC | mutate box -10 TACCGC (Rev) | This study |
| GACGACA_GACGGCA-F | GAACCGCCGTTCGGACGGCATTTGATTTTTGCTTC | mutate terminator from weak to strong (Fw) | This study |
| GACGACA_GACGGCA-R | GAAGCAAAAATCAAATGCCGTCCGAACGGCGGTTC | mutate terminator from weak to strong (Rev) | This study |
| CGGTATG_CAGTATG-F | CCTGCCTCATTGATGCAGTATGCAAAAAAAGATACC | mutate spacer into spacer 1 (Fw) | This study |
| CGGTATG_CAGTATG-R | GGTATCTTTTTTTGCATACTGCATCAATGAGGCAGG | mutate spacer into spacer 1 (Rev) | This study |
| CAGTATG_CAATATG-F | CCTGCCTCATTGATGCAATATGCAAAAAAAGATACC | mutate spacer into spacer 2 (Fw) | This study |
| CAGTATG_CAATATG-R | GGTATCTTTTTTTGCATATTGCATCAATGAGGCAGG | mutate spacer into spacer 2 (Rev) | This study |
| CAGTATG_CAGCATG-F | CCTGCCTCATTGATGCAGCATGCAAAAAAAGATACC | mutate spacer into spacer 3 (Fw) | This study |
| CAGTATG_CAGCATG-R | GGTATCTTTTTTTGCATGCTGCATCAATGAGGCAGG | mutate spacer into spacer 3 (Rev) | This study |
| TTGATG_TTGACG-F | CTTTGACCTGCCTCATTGACGCGGTATGCAAAAAAAG | mutate box -35 TTGACG (Fw) | This study |
| TTGATG_TTGACG-R | CTTTTTTTGCATACCGCGTCAATGAGGCAGGTCAAAG | mutate box -35 TTGACG (Rev) | This study |
| iPCRprom-F | caagggcgaattgaaccaaatCGTCAAATAACAGGTTG | iPCR - universal for the 11 most represented promoter alleles (including leader peptide) (Fw) | This study |
| iPCRprom-R | CCCCCTCCGCTGCTGCAGGCGGTCAGAATCAG | iPCR - universal for the 11 most represented promoter alleles (including leader peptide) (Rev) | This study |
| vPCRpBSc741-F | CAGCAGCGGAGGGGGTGGTGTCGCCGCCGAC | vPCR - pBS-c741 wt CmR (Fw) | This study |
| vPCRpBSc741-R | ATTTGGTTCAATTCGCCCTTGgcataacggcttgcc | vPCR - pBS-c741 wt CmR (Rev) | This study |
| EP1For1.1 | CGCggatcccatatgGTGAATCGAACTGCCTTC | Amplification fHbp var1.14 and var3.28 (Fw). NdeI | Seib *et al.*, [58] |
| EP5RV1.4 | TGCATGCATTTACTGCTTGGCGGCAAG | Amplification fHbp var1.14 (Rev). NsiI | Seib *et al.*, [58] |
| EP2For1.4 | CGCggatcccatatgGTGAACCGAACTGCCTTC | Amplification fHbp var2.16 and var2.25 (Fw). NdeI | Seib *et al.*, [58] |
| EP6RV2.1 | TGCATGCATCTACTGTTTGCCGGCGAT | Amplification fHbp var2.16, var2.25 and var3.28 (Rev). NsiI | Seib *et al.*, [58] |
| 2.21 fw | ATTCGcatatgGTGAACCGAACTGCCTTCTGCTGCC | Amplification fHbp var2.21 (Fw). NdeI | This study |
| 2.21 rev | ATTCGatgcatCTACTGTTTGCCGGCGATGCCG | Amplification fHbp var2.21 (Rev). NsiI | This study |
| 3.45 fw | ATTCGcatatgGTGAACCGAACTGCCTTCTGCTG | Amplification fHbp var3.45 (Fw). NdeI | This study |
| 3.47 fw | ATTCGcatatgGTGAACCGAACTACCTTCTGTTG | Amplification fHbp var3.47 (Fw). NdeI | This study |
| 3.45/47 rev | ATTCGatgcatCTACTGTTTGCCGGCGATGC | Amplification fHbp var3.45 and var3.47 (Rev). NsiI | This study |
| CmR-down | GCACTTCTATACTCTCTGTCG | Complementation check *in locus* (Fw), downstream recombination | This study |
| complcheck-dsGENOME-R | CTGATAATCGCTCAAACG | Complementation check *in locus* (Rev), downstream recombination | This study |
| COM-C-Fw | CCTCGAGCCGCTGACCGAAGG | Complementation check in the Com region (Fw), upstream recombination | This study |
| CM-UP-C | GGTCGAAATACTCTTTTCGTGTCC | Complementation check in the Com region (Rev), upstream recombination | This study |
| COM-C-Rev | ACCGGCATCGGCAACTACAC | Complementation check in the Com region (Rev), downstream recombination | This study |
| pRTNM_nmb1869U-F | GTATCGACCGCATCAAAG | qRT-PCR *cbbA* (Fw) and complementation check *in locus* (Fw), upstream recombination | This study |
| pRTNM_nmb1869U-R | TGACTTTCAGCCATTCTTG | qRT-PCR *cbbA* (Rev) | This study |
| pRTNM_3P-1869-F | CGAATTGAACCAAATCGTC | qRT-PCR intergenic region (Fw) | This study |
| pRTNM_IG-R | CAATGAGGCAGGTCAAAG | qRT-PCR intergenic region (Rev) | This study |
| pRTfHbpU.F | GGCTTGCCGATGCACTAAC | qRT-PCR *fHbp* (Fw) and complementation check in the Com region (Fw), downstream recombination | This study |
| pRTfHbpU.R | GTTTTTTCCGCACCTTGTGC | qRT-PCR *fHbp* (Rev) and complementation check *in locus* (Rev), upstream recombination | This study |
| pRTNM16sII.F1 | GTGGGGAATTTTGGACAATG | qRT-PCR *16S RNA* (Fw) | This study |
| pRTNM16sII.R1 | CAACAGCCTTTTCTTCCCTG | qRT-PCR *16S RNA* (Rev) | This study |
| adk_+45_F | TCAGGCGCAATTCATCAC | qRT-PCR *adk* (Fw) | This study |
| adk_+102_R | CATGTCGCCGGTAGAGATT | qRT-PCR *adk* (Rev) | This study |
