# Supplementary material for: Deconvolution of intergenic polymorphisms determining high expression of Factor H binding protein in meningococcus and their association with invasive disease
Source: PLoS Pathog. 2021 Mar 26;17(3):e1009461. doi: 10.1371/journal.ppat.1009461 (PMC8026042; doi:10.1371/journal.ppat.1009461)
Supplement: S5 Table — AmpR, ampicillin resistance; CmR, chloramphenicol resistance. (DOCX) [file ppat.1009461.s012.docx]

**S5 Table. List of plasmids used in this study.**

| **Name** | **Description** | **Antibiotic resistance** | **Reference** |
| --- | --- | --- | --- |
| pBS-c741 wt CmR | *In locus* complementation of the *fHbp* gene including its upstream promoter region (fIR7). Downstream of *fHbp* is cloned a *cat* cassette | AmpR, CmR | This study |
| pBS-c741 fIR1 CmR | *In locus* complementation of the *fHbp* gene including the upstream fIR1 sequence allele. | AmpR, CmR | This study |
| pBS-c741 fIR2 CmR | *In locus* complementation of the *fHbp* gene including the upstream fIR2 sequence allele. | AmpR, CmR | This study |
| pBS-c741 fIR3 CmR | *In locus* complementation of the *fHbp* gene including the upstream fIR3 sequence allele. | AmpR, CmR | This study |
| pBS-c741 fIR4 CmR | *In locus* complementation of the *fHbp* gene including the upstream fIR4 sequence allele. | AmpR, CmR | This study |
| pBS-c741 fIR6 CmR | *In locus* complementation of the *fHbp* gene including the upstream fIR6 sequence allele. | AmpR, CmR | This study |
| pBS-c741 fIR11 CmR | *In locus* complementation of the *fHbp* gene including the upstream fIR11 sequence allele. | AmpR, CmR | This study |
| pBS-c741 fIR13 CmR | *In locus* complementation of the *fHbp* gene including the upstream fIR13 sequence allele. | AmpR, CmR | This study |
| pBS-c741 fIR15 CmR | *In locus* complementation of the *fHbp* gene including the upstream fIR15 sequence allele. | AmpR, CmR | This study |
| pBS-c741 fIR16 CmR | *In locus* complementation of the *fHbp* gene including the upstream fIR16 sequence allele. | AmpR, CmR | This study |
| pBS-c741 fIR20 CmR | *In locus* complementation of the *fHbp* gene including the upstream fIR20 sequence allele. | AmpR, CmR | This study |
| pComP_IND_ CmR-fHbp var1.1 | Complementation of the *fHbp* gene var1.1 in the Com region with an IPTG-inducible P_TAC_. | AmpR, CmR | Biagini *et al*., [26] |
| pComP_IND_ CmR-fHbp var1.14 | Complementation of the *fHbp* gene var1.14 in the Com region with an IPTG-inducible P_TAC_. | AmpR, CmR | This study |
| pComP_IND_ CmR-fHbp var2.16 | Complementation of the *fHbp* gene var2.16 in the Com region with an IPTG-inducible P_TAC_. | AmpR, CmR | This study |
| pComP_IND_ CmR-fHbp var2.21 | Complementation of the *fHbp* gene var2.21 in the Com region with an IPTG-inducible P_TAC_. | AmpR, CmR | This study |
| pComP_IND_ CmR-fHbp var2.25 | Complementation of the *fHbp* gene var2.25 in the Com region with an IPTG-inducible P_TAC_. | AmpR, CmR | This study |
| pComP_IND_ CmR-fHbp var3.28 | Complementation of the *fHbp* gene var3.28 in the Com region with an IPTG-inducible P_TAC_. | AmpR, CmR | This study |
| pComP_IND_ CmR-fHbp var3.45 | Complementation of the *fHbp* gene var3.45 in the Com region with an IPTG-inducible P_TAC_. | AmpR, CmR | This study |
| pComP_IND_ CmR-fHbp var3.47 | Complementation of the *fHbp* gene var3.47 in the Com region with an IPTG-inducible P_TAC_. | AmpR, CmR | This study |
| pBS-c741 PfHbp -10 box (TACCGC) CmR | *In locus* complementation of the *fHbp* gene including its upstream promoter region (fIR7) mutated in the -10 box: TACCGC. | AmpR, CmR | This study |
| pBS-c741 PfHbp term -27 CmR | *In locus* complementation of the *fHbp* gene including its upstream promoter region (fIR7) mutated in the terminator: strong. | AmpR, CmR | This study |
| pBS-c741 PfHbp term -27, -10 box (TACCGC) CmR | *In locus* complementation of the *fHbp* gene including its upstream promoter region (fIR7) mutated in the terminator: strong; and in the -10 box: TACCGC. | AmpR, CmR | This study |
| pBS-c741 PfHbp term -27, spacer1 CmR | *In locus* complementation of the *fHbp* gene including its upstream promoter region (fIR7) mutated in the terminator: strong; spacer: CAGTATGCAAAAAAAGA. | AmpR, CmR | This study |
| pBS-c741 PfHbp term -27, spacer2 CmR | *In locus* complementation of the *fHbp* gene including its upstream promoter region (fIR7) mutated in the terminator: strong; spacer: CAATATGCAAAAAAAGA. | AmpR, CmR | This study |
| pBS-c741 PfHbp term -27, spacer3 CmR | *In locus* complementation of the *fHbp* gene including its upstream promoter region (fIR7) mutated in the terminator: strong; spacer: CAGCATGCAAAAAAAGA. | AmpR, CmR | This study |
| pBS-c741 PfHbp term -27, -35 box (TTGACG) CmR | *In locus* complementation of the *fHbp* gene including its upstream promoter region (fIR7) mutated in the terminator: strong; and in the -35 box: TTGACG. | AmpR, CmR | This study |

AmpR, ampicillin resistance; CmR, chloramphenicol resistance.
