# Supplementary material for: Deconvolution of intergenic polymorphisms determining high expression of Factor H binding protein in meningococcus and their association with invasive disease
Source: PLoS Pathog. 2021 Mar 26;17(3):e1009461. doi: 10.1371/journal.ppat.1009461 (PMC8026042; doi:10.1371/journal.ppat.1009461)
Supplement: S6 Table — (PDF) [file ppat.1009461.s013.pdf]

Table S6. Dataset used for this study.

id	isolate	country	year	disease	species	serogroup	genogroup	capsule_group	fHbp_peptide	igr_up_NEIS0349	igr_up_NEIS0350	NEIS0349	fIR	abcZ	adk	aroE	fumC	gdh	pdhC	pgm	clonal_complex..MLST.	ST..MLST.	fIR_most	expr_group	fHbpVar
35227	09.1126.Y	UK [Scotland]	2009	invasive (unspecified/other)	Neisseria meningitidis	B	B	B	13	3	57	145	3	4	10	34	5	38	11	9	ST-269 complex	1161	3	3	1
35228	09.1208.P	UK [Scotland]	2009	invasive (unspecified/other)	Neisseria meningitidis	B	B	B	309	1	1	244	4	1	5	13	53	26	41	3	ST-162 complex	162	4	3	1
35229	09.1211.Y	UK [Scotland]	2009	invasive (unspecified/other)	Neisseria meningitidis	B	B	B	13	3	57	145	3	4	10	2	5	38	11	9	ST-269 complex	275	3	3	1
35230	09.1292.T	UK [Scotland]	2009	invasive (unspecified/other)	Neisseria meningitidis	B	B	B	309	1	1	244	4	1	5	13	53	26	41	3	ST-162 complex	162	4	3	1
35231	09.1331.Y	UK [Scotland]	2009	invasive (unspecified/other)	Neisseria meningitidis	B	B	B	4	2	79	84	2	3	16	325	5	9	6	9	ST-41/44 complex	5981	2	3	1
36668	09.1347.W	UK [Scotland]	2009	invasive (unspecified/other)	Neisseria meningitidis	B	B	B	13	3	57	145	3	4	5	2	5	38	11	7		11384	3	3	1
35232	09.1368.F	UK [Scotland]	2009	invasive (unspecified/other)	Neisseria meningitidis	B	B	B	19	1	1	65	4	28	6	9	9	9	6	9	ST-41/44 complex	207	4	4	2
35233	09.1385.H	UK [Scotland]	2009	invasive (unspecified/other)	Neisseria meningitidis	Y	Y	Y	25	1	1	26	4	12	5	18	NA	11	9	17		NA	4	4	2
35234	09.1402.B	UK [Scotland]	2009	invasive (unspecified/other)	Neisseria meningitidis	B	B	B	45	8	77	87	20	7	5	771	13	36	53	15	ST-213 complex	11221	20	4	3
35235	09.1453.F	UK [Scotland]	2009	invasive (unspecified/other)	Neisseria meningitidis	B	B	B	4	2	1	84	2	3	6	9	5	9	6	9	ST-41/44 complex	41	2	3	1
35236	09.1507.Q	UK [Scotland]	2009	invasive (unspecified/other)	Neisseria meningitidis	B	B	B	4	2	1	84	2	3	6	9	11	11	6	9	ST-41/44 complex	2989	2	3	1
35237	09.1514.A	UK [Scotland]	2009	invasive (unspecified/other)	Neisseria meningitidis	B	B	B	13	3	57	145	3	4	10	2	5	38	11	9	ST-269 complex	275	3	3	1
35238	09.1557.B	UK [Scotland]	2009	invasive (unspecified/other)	Neisseria meningitidis	B	B	B	45	91	77	87	172	7	5	1	13	36	53	15	ST-213 complex	213	NA	NA	3
35239	09.1580.V	UK [Scotland]	2009	invasive (unspecified/other)	Neisseria meningitidis	B	B	B	1	6	7	100	7	4	10	5	4	6	3	8	ST-32 complex	32	7	1	1
35240	09.1595.J	UK [Scotland]	2009	invasive (unspecified/other)	Neisseria meningitidis	B	B	B	16	1	4	59	4	4	10	11	18	6	10	12	ST-35 complex	35	4	4	2
35241	09.1619.R	UK [Scotland]	2009	invasive (unspecified/other)	Neisseria meningitidis	B	B	B	4	2	1	84	2	3	6	9	5	9	6	9	ST-41/44 complex	41	2	3	1
35242	09.1857.M	UK [Scotland]	2009	invasive (unspecified/other)	Neisseria meningitidis	B	B	B	13	3	57	209	3	8	25	7	17	21	26	49	ST-1157 complex	1157	3	3	1
35243	09.1858.V	UK [Scotland]	2009	invasive (unspecified/other)	Neisseria meningitidis	B	B	B	15	10	2	193	17	4	10	15	9	8	11	9	ST-269 complex	269	NA	NA	1
35244	09.1893.P	UK [Scotland]	2009	invasive (unspecified/other)	Neisseria meningitidis	B	B	B	15	10	2	193	17	4	10	15	9	8	11	9	ST-269 complex	269	NA	NA	1
35378	09.1936.F	UK [Scotland]	2009	invasive (unspecified/other)	Neisseria meningitidis	B	B	B	4	2	1	84	2	3	6	9	5	9	6	9	ST-41/44 complex	41	2	3	1
35245	09.1976.T	UK [Scotland]	2009	invasive (unspecified/other)	Neisseria meningitidis	B	B	B	19	1	1	83	4	4	5	2	5	38	11	9		2307	4	4	2
35246	09.1999.X	UK [Scotland]	2009	invasive (unspecified/other)	Neisseria meningitidis	B	B	B	309	1	1	244	4	1	5	13	53	26	41	3	ST-162 complex	162	4	3	1
35247	09.2014.G	UK [Scotland]	2009	invasive (unspecified/other)	Neisseria meningitidis	B	B	B	19	1	1	65	4	9	20	9	9	9	6	2	ST-41/44 complex	1097	4	4	2
35248	09.2045.J	UK [Scotland]	2009	invasive (unspecified/other)	Neisseria meningitidis	B	B	B	4	2	1	84	2	3	6	9	5	9	6	9	ST-41/44 complex	41	2	3	1
35249	09.2061.P	UK [Scotland]	2009	invasive (unspecified/other)	Neisseria meningitidis	Y	Y	Y	25	1	1	26	4	12	5	18	9	11	9	17	ST-23 complex	1655	4	4	2
35250	09.2082.C	UK [Scotland]	2009	invasive (unspecified/other)	Neisseria meningitidis	B	B	B	19	1	1	65	4	27	6	9	5	36	6	9	ST-41/44 complex	7415	4	4	2
35251	09.2204.A	UK [Scotland]	2009	invasive (unspecified/other)	Neisseria meningitidis	B	B	B	19	1	1	65	4	17	5	19	17	3	26	740	ST-60 complex	11222	4	4	2
35252	09.2270.V	UK [Scotland]	2009	invasive (unspecified/other)	Neisseria meningitidis	B	B	B	4	1	59	84	4	3	6	9	5	9	21	9	ST-41/44 complex	2266	4	3	1
35253	09.2386.V	UK [Scotland]	2009	invasive (unspecified/other)	Neisseria meningitidis	B	B	B	14	5	1	204	11	3	6	19	5	3	6	9	ST-41/44 complex	340	11	2	1
35254	09.2439.W	UK [Scotland]	2009	invasive (unspecified/other)	Neisseria meningitidis	B	B	B	22	1	1	1	4	7	5	1	13	36	53	15	ST-213 complex	213	4	4	2
35255	09.2522.Q	UK [Scotland]	2009	invasive (unspecified/other)	Neisseria meningitidis	B	B	B	14	7	1	142	6	10	6	9	5	9	6	9	ST-41/44 complex	42	6	2	1
35256	09.2622.N	UK [Scotland]	2009	invasive (unspecified/other)	Neisseria meningitidis	B	B	B	15	4	2	193	1	4	10	15	17	8	11	17	ST-269 complex	479	1	1	1
35257	09.2658.E	UK [Scotland]	2009	invasive (unspecified/other)	Neisseria meningitidis	B	B	B	4	2	1	84	2	3	6	9	5	9	6	9	ST-41/44 complex	41	2	3	1
35258	09.2799.W	UK [Scotland]	2009	invasive (unspecified/other)	Neisseria meningitidis	W	W	W	16	1	1	60	4	11	5	18	8	78	24	21	ST-22 complex	1224	4	4	2
35379	09.2807.A	UK [Scotland]	2009	invasive (unspecified/other)	Neisseria meningitidis	B	B	B	29	12	77	49	76	7	5	1	13	36	53	15	ST-213 complex	213	NA	NA	3
35259	09.2935.B	UK [Scotland]	2009	invasive (unspecified/other)	Neisseria meningitidis	B	B	B	25	1	1	26	4	8	4	6	17	5	18	2	ST-103 complex	103	4	4	2
35260	09.2939.N	UK [Scotland]	2009	invasive (unspecified/other)	Neisseria meningitidis	B	B	B	4	2	1	84	2	7	5	1	13	36	53	15	ST-213 complex	213	2	3	1
35261	09.2961.S	UK [Scotland]	2009	invasive (unspecified/other)	Neisseria meningitidis	B	B	B	4	2	1	84	2	3	6	9	5	11	6	9	ST-41/44 complex	154	2	3	1
35262	09.3026.N	UK [Scotland]	2009	invasive (unspecified/other)	Neisseria meningitidis	B	B	B	13	3	57	145	3	4	10	2	5	38	315	9		4388	3	3	1
35263	09.3029.P	UK [Scotland]	2009	invasive (unspecified/other)	Neisseria meningitidis	B	B	B	13	3	57	145	3	4	10	2	5	38	315	9		4388	3	3	1
35264	09.3063.L	UK [Scotland]	2009	invasive (unspecified/other)	Neisseria meningitidis	B	B	B	21	1	1	7	4	1	5	13	53	26	41	3	ST-162 complex	162	4	4	2
35265	09.3091.W	UK [Scotland]	2009	invasive (unspecified/other)	Neisseria meningitidis	B	B	B	19	1	1	448	4	4	10	2	5	3	11	9	ST-269 complex	1163	4	4	2
35266	09.3167.H	UK [Scotland]	2009	invasive (unspecified/other)	Neisseria meningitidis	Y	Y	Y	21	1	1	7	4	6	5	124	13	5	24	17	ST-174 complex	7867	4	4	2
35268	09.3302.K	UK [Scotland]	2009	invasive (unspecified/other)	Neisseria meningitidis	Y	Y	Y	21	1	1	7	4	6	5	173	13	5	24	17	ST-174 complex	1466	4	4	2
35269	09.3351.N	UK [Scotland]	2009	invasive (unspecified/other)	Neisseria meningitidis	B	B	B	15	4	2	193	1	4	10	15	17	8	11	17	ST-269 complex	479	1	1	1
35270	09.3368.H	UK [Scotland]	2009	invasive (unspecified/other)	Neisseria meningitidis	B	B	B	13	3	1	145	3	3	6	9	5	9	6	9	ST-41/44 complex	41	3	3	1
35271	10.1171.X	UK [Scotland]	2010	invasive (unspecified/other)	Neisseria meningitidis	Y	Y	Y	293	23	1	114	49	10	5	18	9	11	9	17	ST-23 complex	23	NA	NA	3
35272	10.1177.P	UK [Scotland]	2010	invasive (unspecified/other)	Neisseria meningitidis	B	B	B	1	6	3	100	7	4	10	5	40	6	3	8	ST-32 complex	259	7	1	1
35273	10.1199.K	UK [Scotland]	2010	invasive (unspecified/other)	Neisseria meningitidis	B	B	B	15	4	2	193	1	4	10	15	9	8	5	9	ST-269 complex	1195	1	1	1
35274	10.1352.R	UK [Scotland]	2010	invasive (unspecified/other)	Neisseria meningitidis	B	B	B	45	8	77	87	20	7	5	1	13	36	53	15	ST-213 complex	213	20	4	3
35275	10.1430.T	UK [Scotland]	2010	invasive (unspecified/other)	Neisseria meningitidis	B	B	B	1	6	7	100	7	4	10	5	4	6	3	9	ST-32 complex	800	7	1	1
35276	10.1516.S	UK [Scotland]	2010	invasive (unspecified/other)	Neisseria meningitidis	B	B	B	15	4	2	193	1	4	10	15	9	8	5	9	ST-269 complex	1195	1	1	1
35277	10.1718.Z	UK [Scotland]	2010	invasive (unspecified/other)	Neisseria meningitidis	B	B	B	13	3	1	145	3	17	5	19	17	3	26	12	ST-60 complex	2002	3	3	1
35278	10.1746.F	UK [Scotland]	2010	invasive (unspecified/other)	Neisseria meningitidis	B	B	B	19	1	1	83	4	4	10	2	5	38	11	9	ST-269 complex	275	4	4	2
35279	10.1962.Z	UK [Scotland]	2010	invasive (unspecified/other)	Neisseria meningitidis	B	B	B	13	3	57	145	3	4	10	2	5	38	315	9		4388	3	3	1
35280	10.2012.H	UK [Scotland]	2010	invasive (unspecified/other)	Neisseria meningitidis	B	B	B	4	2	79	84	2	3	6	9	5	26	6	9	ST-41/44 complex	8054	2	3	1
35281	10.2126.Z	UK [Scotland]	2010	invasive (unspecified/other)	Neisseria meningitidis	B	B	B	2	5	1	280	11	4	10	15	17	8	11	17	ST-269 complex	479	11	2	1
35282	10.2240.T	UK [Scotland]	2010	invasive (unspecified/other)	Neisseria meningitidis	B	B	B	13	3	57	145	3	4	10	34	5	60	11	9	ST-269 complex	8440	3	3	1
35283	10.2273.P	UK [Scotland]	2010	invasive (unspecified/other)	Neisseria meningitidis	W	W	W	16	1	1	60	4	11	5	18	8	11	4	21	ST-22 complex	184	4	4	2
35284	10.2289.A	UK [Scotland]	2010	invasive (unspecified/other)	Neisseria meningitidis	B	B	B	13	3	57	145	3	4	10	34	5	38	11	9	ST-269 complex	1161	3	3	1
35285	10.2352.E	UK [Scotland]	2010	invasive (unspecified/other)	Neisseria meningitidis	B	B	B	13	3	57	145	3	4	10	34	5	38	11	9	ST-269 complex	1161	3	3	1
35286	10.2397.V	UK [Scotland]	2010	invasive (unspecified/other)	Neisseria meningitidis	B	B	B	220	92	1	449	139	3	10	15	17	6	10	12	ST-35 complex	1764	NA	NA	1
35287	10.2422.K	UK [Scotland]	2010	invasive (unspecified/other)	Neisseria meningitidis	B	B	B	14	5	1	204	11	3	6	19	5	3	6	9	ST-41/44 complex	340	11	2	1
35288	10.2509.N	UK [Scotland]	2010	invasive (unspecified/other)	Neisseria meningitidis	B	B	B	15	4	1	193	1	4	10	15	9	8	53	9	ST-269 complex	8487	1	1	1
35289	10.2631.L	UK [Scotland]	2010	invasive (unspecified/other)	Neisseria meningitidis	Z	Z	Z	25	1	1	26	4	8	4	6	17	5	18	2	ST-103 complex	103	4	4	2
35290	10.2763.M	UK [Scotland]	2010	invasive (unspecified/other)	Neisseria meningitidis	B	B	B	4	2	1	84	2	3	6	15	5	247	6	9	ST-41/44 complex	8539	2	3	1
35291	10.2850.R	UK [Scotland]	2010	invasive (unspecified/other)	Neisseria meningitidis	B	B	B	4	2	1	84	2	3	6	9	5	11	6	9	ST-41/44 complex	154	2	3	1
35292	10.2854.Q	UK [Scotland]	2010	invasive (unspecified/other)	Neisseria meningitidis	B	B	B	354	93	9	41	155	7	5	1	13	36	53	741	ST-213 complex	11223	NA	NA	3
35293	10.2884.V	UK [Scotland]	2010	invasive (unspecified/other)	Neisseria meningitidis	B	B	B	19	1	1	83	4	4	10	2	5	563	11	9	ST-269 complex	8616	4	4	2
35294	10.3051.M	UK [Scotland]	2010	invasive (unspecified/other)	Neisseria meningitidis	B	B	B	19	1	2	450	4	20	6	63	9	9	11	9	ST-41/44 complex	944	4	4	2
35295	10.3134.R	UK [Scotland]	2010	invasive (unspecified/other)	Neisseria meningitidis	Y	Y	Y	16	1	1	60	4	11	5	18	17	11	24	21	ST-22 complex	114	4	4	2
35296	11.1071.G	UK [Scotland]	2011	invasive (unspecified/other)	Neisseria meningitidis	B	B	B	14	5	1	204	11	3	6	19	5	3	6	9	ST-41/44 complex	340	11	2	1
35297	11.1190.L	UK [Scotland]	2010	invasive (unspecified/other)	Neisseria meningitidis	B	B	B	4	2	1	84	2	3	6	34	13	11	6	9	ST-41/44 complex	8731	2	3	1
35298	11.1286.D	UK [Scotland]	2011	invasive (unspecified/other)	Neisseria meningitidis	B	B	B	4	2	1	84	2	3	6	34	8	11	6	9	ST-41/44 complex	8722	2	3	1
35299	11.1312.X	UK [Scotland]	2011	invasive (unspecified/other)	Neisseria meningitidis	B	B	B	14	7	1	142	6	10	6	9	5	9	6	9	ST-41/44 complex	42	6	2	1
35300	11.1376.W	UK [Scotland]	2011	invasive (unspecified/other)	Neisseria meningitidis	B	B	B	1	6	7	100	7	4	10	5	4	6	3	9	ST-32 complex	800	7	1	1
35301	11.1392.G	UK [Scotland]	2011	invasive (unspecified/other)	Neisseria meningitidis	B	B	B	15	4	2	193	1	4	10	15	9	8	11	9	ST-269 complex	269	1	1	1
35302	11.1426.J	UK [Scotland]	2011	invasive (unspecified/other)	Neisseria meningitidis	B	B	B	4	2	1	84	2	3	6	9	5	11	6	9	ST-41/44 complex	154	2	3	1
35303	11.1479.T	UK [Scotland]	2011	invasive (unspecified/other)	Neisseria meningitidis	B	B	B	4	2	6	84	2	3	6	9	5	9	6	9	ST-41/44 complex	41	2	3	1
35304	11.1489.Z	UK [Scotland]	2011	invasive (unspecified/other)	Neisseria meningitidis	B	B	B	13	3	1	145	3	17	5	1	17	3	26	2	ST-60 complex	2923	3	3	1
35305	11.1575.Q	UK [Scotland]	2011	invasive (unspecified/other)	Neisseria meningitidis	B	B	B	13	3	57	145	3	23	3	12	25	3	22	12		3318	3	3	1
35306	11.1688.D	UK [Scotland]	2011	invasive (unspecified/other)	Neisseria meningitidis	B	B	B	45	8	77	87	20	7	5	1	13	36	6	15	ST-213 complex	2660	20	4	3
35307	11.1709.B	UK [Scotland]	2011	invasive (unspecified/other)	Neisseria meningitidis	B	B	B	45	94	77	87	154	7	5	1	13	36	53	15	ST-213 complex	213	NA	NA	3
35308	11.1825.A	UK [Scotland]	2011	invasive (unspecified/other)	Neisseria meningitidis	B	B	B	19	1	1	65	4	8	5	9	9	9	6	9	ST-41/44 complex	6566	4	4	2
35309	11.1826.C	UK [Scotland]	2011	invasive (unspecified/other)	Neisseria meningitidis	B	B	B	19	1	1	65	4	8	5	9	9	9	6	9	ST-41/44 complex	6566	4	4	2
35310	11.2087.F	UK [Scotland]	2011	invasive (unspecified/other)	Neisseria meningitidis	B	B	B	45	8	77	87	20	7	5	1	13	36	53	15	ST-213 complex	213	20	4	3
35311	11.2169.T	UK [Scotland]	2011	invasive (unspecified/other)	Neisseria meningitidis	B	B	B	19	1	1	83	4	4	10	2	5	3	11	9	ST-269 complex	1163	4	4	2
35312	11.2463.D	UK [Scotland]	2011	invasive (unspecified/other)	Neisseria meningitidis	B	B	B	4	2	1	84	2	10	6	9	5	9	18	9	ST-41/44 complex	2806	2	3	1
35313	11.2525.L	UK [Scotland]	2011	invasive (unspecified/other)	Neisseria meningitidis	B	B	B	13	3	57	145	3	4	10	34	5	38	11	9	ST-269 complex	1161	3	3	1
36669	11.2796.K	UK [Scotland]	2011	invasive (unspecified/other)	Neisseria meningitidis	B	B	B	47	1	57	90	15	12	5	12	35	60	22	17	ST-461 complex	461	15	4	3
35314	11.2909.Z	UK [Scotland]	2011	invasive (unspecified/other)	Neisseria meningitidis	Y	Y	Y	25	1	1	26	4	12	5	18	9	11	9	17	ST-23 complex	1655	4	4	2
35315	11.3133.X	UK [Scotland]	2011	invasive (unspecified/other)	Neisseria meningitidis	B	B	B	309	1	1	244	4	1	5	13	53	26	41	3	ST-162 complex	162	4	3	1
35316	11.3808.A	UK [Scotland]	2011	invasive (unspecified/other)	Neisseria meningitidis	B	B	B	210	1	1	267	4	134	10	15	9	14	11	12	ST-269 complex	8926	4	3	1
35317	11.3888.K	UK [Scotland]	2011	invasive (unspecified/other)	Neisseria meningitidis	Y	Y	Y	25	1	1	26	4	12	5	18	9	11	9	17	ST-23 complex	1655	4	4	2
35318	11.4891.D	UK [Scotland]	2011	invasive (unspecified/other)	Neisseria meningitidis	B	B	B	13	3	57	145	3	4	10	18	5	38	11	9	ST-269 complex	8227	3	3	1
35319	11.4999.T	UK [Scotland]	2011	invasive (unspecified/other)	Neisseria meningitidis	B	B	B	14	30	1	142	71	3	6	9	5	111	6	9	ST-41/44 complex	7385	NA	NA	1
35320	11.5380.D	UK [Scotland]	2011	invasive (unspecified/other)	Neisseria meningitidis	B	B	B	232	2	1	339	2	12	2	9	9	15	54	10		9000	2	3	1
35321	11.5384.H	UK [Scotland]	2011	invasive (unspecified/other)	Neisseria meningitidis	B	B	B	19	1	1	83	4	4	10	2	5	38	11	9	ST-269 complex	275	4	4	2
35322	11.5445.E	UK [Scotland]	2011	invasive (unspecified/other)	Neisseria meningitidis	B	B	B	13	3	57	209	3	8	25	7	17	21	26	49	ST-1157 complex	1157	3	3	1
35323	11.5563.G	UK [Scotland]	2011	invasive (unspecified/other)	Neisseria meningitidis	W	W	W	16	1	1	60	4	11	5	18	8	11	4	21	ST-22 complex	184	4	4	2
52830	11,562	UK [Scotland]	2011	invasive (unspecified/other)	Neisseria meningitidis	B	B	B	14	1	1	142	13	3	6	108	5	9	6	9	ST-41/44 complex	1475	13	3	1
35324	11.5842.B	UK [Scotland]	2011	invasive (unspecified/other)	Neisseria meningitidis	B	B	B	14	1	1	142	13	3	6	9	5	3	10	9	ST-41/44 complex	3866	13	3	1
35325	11.5915.A	UK [Scotland]	2011	invasive (unspecified/other)	Neisseria meningitidis	B	B	B	45	8	77	87	20	7	5	1	NA	36	53	15		NA	20	4	3
35326	11.6644.A	UK [Scotland]	2011	invasive (unspecified/other)	Neisseria meningitidis	B	B	B	13	3	57	145	3	4	10	34	5	616	11	9	ST-269 complex	9198	3	3	1
35327	11.6835.D	UK [Scotland]	2011	invasive (unspecified/other)	Neisseria meningitidis	B	B	B	95	5	1	32	11	4	10	72	9	6	10	12	ST-35 complex	2380	11	2	2
35328	11.7141.B	UK [Scotland]	2011	invasive (unspecified/other)	Neisseria meningitidis	B	B	B	141	1	4	116	4	4	10	15	9	8	11	9	ST-269 complex	269	4	4	3
35329	12.1092.Y	UK [Scotland]	2012	invasive (unspecified/other)	Neisseria meningitidis	B	B	B	25	1	1	26	4	8	4	6	17	5	18	2	ST-103 complex	103	4	4	2
35330	12.1192.H	UK [Scotland]	2012	invasive (unspecified/other)	Neisseria meningitidis	B	B	B	47	1	57	90	15	12	5	12	35	192	22	17	ST-461 complex	1946	15	4	3
35331	12.1222.T	UK [Scotland]	2012	invasive (unspecified/other)	Neisseria meningitidis	B	B	B	589	1	1	451	4	17	5	19	17	3	26	2	ST-60 complex	60	4	3	1
35332	12.1512.D	UK [Scotland]	2012	invasive (unspecified/other)	Neisseria meningitidis	Y	Y	Y	25	1	1	26	4	12	5	18	9	11	9	17	ST-23 complex	1655	4	4	2
35333	12.1555.H	UK [Scotland]	2012	invasive (unspecified/other)	Neisseria meningitidis	W	W	W	16	1	25	60	4	11	5	367	8	11	4	21	ST-22 complex	4475	4	4	2
35356	12.1860.E	UK [Scotland]	2012	invasive (unspecified/other)	Neisseria meningitidis	B	B	B	19	1	1	448	4	4	10	2	5	3	11	9	ST-269 complex	1163	4	4	2
35357	12.1954.S	UK [Scotland]	2012	invasive (unspecified/other)	Neisseria meningitidis	B	B	B	19	8	10	129	20	4	10	2	5	38	11	9	ST-269 complex	275	20	4	2
35358	12.2326.H	UK [Scotland]	2012	invasive (unspecified/other)	Neisseria meningitidis	B	B	B	238	21	5	210	19	20	6	63	9	9	11	2		284	NA	NA	1
35359	12.2412.W	UK [Scotland]	2012	invasive (unspecified/other)	Neisseria meningitidis	Y	Y	Y	25	1	1	26	4	10	5	18	9	11	9	17	ST-23 complex	23	4	4	2
35360	12.2589.T	UK [Scotland]	2012	invasive (unspecified/other)	Neisseria meningitidis	B	B	B	21	1	1	7	4	1	5	13	53	26	41	623	ST-162 complex	9293	4	4	2
35361	12.2953.Y	UK [Scotland]	2012	invasive (unspecified/other)	Neisseria meningitidis	B	B	B	260	1	2	453	4	6	6	2	9	3	11	20		9383	4	3	1
35362	12.3021.W	UK [Scotland]	2012	invasive (unspecified/other)	Neisseria meningitidis	B	B	B	4	2	1	84	2	3	6	9	5	3	6	9	ST-41/44 complex	46	2	3	1
35363	12.3084.V	UK [Scotland]	2012	invasive (unspecified/other)	Neisseria meningitidis	B	B	B	30	17	11	54	37	9	6	351	9	8	24	311		9408	NA	NA	3
35380	12.3882.E	UK [Scotland]	2012	invasive (unspecified/other)	Neisseria meningitidis	Y	Y	Y	94	57	56	89	NA	11	5	18	17	11	24	21	ST-22 complex	114	NA	NA	3
35364	12.4811.X	UK [Scotland]	2012	invasive (unspecified/other)	Neisseria meningitidis	B	B	B	19	1	1	83	4	4	10	2	5	3	11	9	ST-269 complex	1163	4	4	2
35365	12.4819.T	UK [Scotland]	2012	invasive (unspecified/other)	Neisseria meningitidis	B	B	B	15	4	2	193	1	4	10	15	9	8	11	9	ST-269 complex	269	1	1	1
35366	12.4885.X	UK [Scotland]	2012	invasive (unspecified/other)	Neisseria meningitidis	C	C	C	819	53	1	454	105	8	3	154	17	26	3	2		6610	NA	NA	1
35367	12.4886.L	UK [Scotland]	2012	invasive (unspecified/other)	Neisseria meningitidis	C	C	C	819	53	1	454	105	8	3	154	17	26	3	2		6610	NA	NA	1
35368	12.6364.V	UK [Scotland]	2012	invasive (unspecified/other)	Neisseria meningitidis	B	B	B	19	1	2	65	4	6	6	63	9	6	11	9		5952	4	4	2
35369	12.6501.H	UK [Scotland]	2012	invasive (unspecified/other)	Neisseria meningitidis	B	B	B	170	9	77	94	21	604	5	1	13	36	53	15	ST-213 complex	9753	NA	NA	3
35370	12.7012.F	UK [Scotland]	2012	invasive (unspecified/other)	Neisseria meningitidis	Y	Y	Y	25	1	1	26	4	12	5	18	9	11	9	17	ST-23 complex	1655	4	4	2
35371	12.7350.H	UK [Scotland]	2012	invasive (unspecified/other)	Neisseria meningitidis	W	W	W	22	1	1	1	4	2	3	4	3	8	4	6	ST-11 complex	11	4	4	2
35372	12.7377.D	UK [Scotland]	2012	invasive (unspecified/other)	Neisseria meningitidis	B	B	B	13	3	57	145	3	4	10	2	5	38	315	9		4388	3	3	1
35373	12.7548.W	UK [Scotland]	2012	invasive (unspecified/other)	Neisseria meningitidis	B	B	B	13	3	57	145	3	4	10	2	5	38	11	9	ST-269 complex	275	3	3	1
35374	12.8180.Y	UK [Scotland]	2012	invasive (unspecified/other)	Neisseria meningitidis	B	B	B	109	95	1	455	174	4	10	2	5	38	11	9	ST-269 complex	275	NA	NA	2
35375	12.8381.Y	UK [Scotland]	2012	invasive (unspecified/other)	Neisseria meningitidis	B	B	B	260	1	1	452	13	4	10	2	9	38	11	16	ST-269 complex	5372	13	3	1
35376	12.8408.J	UK [Scotland]	2012	invasive (unspecified/other)	Neisseria meningitidis	Y	Y	Y	25	1	1	26	4	12	5	18	9	11	9	17	ST-23 complex	1655	4	4	2
35377	12.8444.A	UK [Scotland]	2012	invasive (unspecified/other)	Neisseria meningitidis	C	C	C	16	1	4	59	4	2	3	7	2	8	5	2	ST-8 complex	8	4	4	2
35334	12.8515.D	UK [Scotland]	2012	invasive (unspecified/other)	Neisseria meningitidis	B	B	B	260	1	1	452	13	4	10	2	9	38	11	16	ST-269 complex	5372	13	3	1

35335	12.8534.X	UK [Scotland]	2012	invasive (unspecified/other)	Neisseria meningitidis	B	B	B	4	2	1	84	2	3	6	9	17	5	6	9	ST-41/44 complex	9996	2	3	1
35336	12.8840.D	UK [Scotland]	2012	invasive (unspecified/other)	Neisseria meningitidis	Y	Y	Y	25	1	1	26	4	12	5	18	9	11	9	17	ST-23 complex	1655	4	4	2
35337	13.1012.H	UK [Scotland]	2012	invasive (unspecified/other)	Neisseria meningitidis	B	B	B	13	3	57	145	3	4	10	34	5	38	11	9	ST-269 complex	1161	3	3	1
35338	13.1296.J	UK [Scotland]	2013	invasive (unspecified/other)	Neisseria meningitidis	Y	Y	Y	24	1	2	25	4	12	5	18	9	11	9	17	ST-23 complex	1655	4	4	2
35339	13.1297.B	UK [Scotland]	2013	invasive (unspecified/other)	Neisseria meningitidis	B	B	B	260	1	1	452	13	4	10	2	9	38	11	16	ST-269 complex	5372	13	3	1
35340	13.1370.Z	UK [Scotland]	2013	invasive (unspecified/other)	Neisseria meningitidis	B	B	B	393	34	77	387	173	7	5	1	13	36	53	15	ST-213 complex	213	NA	NA	1
35341	13.1395.T	UK [Scotland]	2013	invasive (unspecified/other)	Neisseria meningitidis	B	B	B	13	3	57	145	3	4	10	34	5	38	11	9	ST-269 complex	1161	3	3	1
35342	13.1396.M	UK [Scotland]	2013	invasive (unspecified/other)	Neisseria meningitidis	B	B	B	15	4	2	193	1	4	10	15	9	8	11	9	ST-269 complex	269	1	1	1
35343	13.1682.M	UK [Scotland]	2013	invasive (unspecified/other)	Neisseria meningitidis	B	B	B	13	3	57	145	3	4	10	34	5	38	11	9	ST-269 complex	1161	3	3	1
35344	13.2342.Z	UK [Scotland]	2013	invasive (unspecified/other)	Neisseria meningitidis	B	B	B	13	3	57	145	3	4	10	34	5	38	11	9	ST-269 complex	1161	3	3	1
35345	13.2973.F	UK [Scotland]	2013	invasive (unspecified/other)	Neisseria meningitidis	B	B	B	47	1	57	90	15	12	5	12	35	60	22	17	ST-461 complex	461	15	4	3
35346	13.3065.D	UK [Scotland]	2013	invasive (unspecified/other)	Neisseria meningitidis	B	B	B	47	1	57	90	15	12	5	12	35	60	22	17	ST-461 complex	461	15	4	3
35347	13.3207.X	UK [Scotland]	2013	invasive (unspecified/other)	Neisseria meningitidis	B	B	B	45	42	1	87	53	7	5	6	13	36	53	22	ST-213 complex	10201	NA	NA	3
35348	13.3348.S	UK [Scotland]	2013	invasive (unspecified/other)	Neisseria meningitidis	Y	Y	Y	25	1	1	26	4	10	5	18	9	11	9	17	ST-23 complex	23	4	4	2
35349	13.3789.S	UK [Scotland]	2013	invasive (unspecified/other)	Neisseria meningitidis	Y	Y	Y	25	1	1	26	4	12	5	18	9	11	9	17	ST-23 complex	1655	4	4	2
35350	13.3813.B	UK [Scotland]	2013	invasive (unspecified/other)	Neisseria meningitidis	B	B	B	19	1	1	83	4	4	10	2	5	38	11	9	ST-269 complex	275	4	4	2
35351	13.3867.V	UK [Scotland]	2013	invasive (unspecified/other)	Neisseria meningitidis	B	B	B	13	3	57	145	3	4	10	34	5	38	11	9	ST-269 complex	1161	3	3	1
35352	13.4061.L	UK [Scotland]	2013	invasive (unspecified/other)	Neisseria meningitidis	B	B	B	260	1	1	452	13	4	10	2	9	38	11	16	ST-269 complex	5372	13	3	1
35353	13.4142.L	UK [Scotland]	2013	invasive (unspecified/other)	Neisseria meningitidis	B	B	B	15	10	2	193	17	4	10	15	9	8	53	9	ST-269 complex	8487	NA	NA	1
35354	13.4278.S	UK [Scotland]	2013	invasive (unspecified/other)	Neisseria meningitidis	B	B	B	4	2	1	84	2	3	6	9	5	9	6	9	ST-41/44 complex	41	2	3	1
35355	13.4514.P	UK [Scotland]	2013	invasive (unspecified/other)	Neisseria meningitidis	B	B	B	1	6	3	100	7	8	83	5	4	5	3	8	ST-32 complex	1098	7	1	1
35381	13.4765.S	UK [Scotland]	2013	invasive (unspecified/other)	Neisseria meningitidis	B	B	B	4	2	1	84	2	3	6	9	5	9	6	9	ST-41/44 complex	41	2	3	1
35382	13.4767.Q	UK [Scotland]	2013	invasive (unspecified/other)	Neisseria meningitidis	Y	Y	Y	25	1	1	26	4	12	5	18	9	11	9	17	ST-23 complex	1655	4	4	2
35383	13.5135.Z	UK [Scotland]	2013	invasive (unspecified/other)	Neisseria meningitidis	B	B	B	16	1	4	59	4	4	10	11	18	6	10	12	ST-35 complex	35	4	4	2
35384	13.5154.G	UK [Scotland]	2013	invasive (unspecified/other)	Neisseria meningitidis	Y	Y	Y	25	1	1	26	4	12	5	18	9	11	9	17	ST-23 complex	1655	4	4	2
35385	13.5226.K	UK [Scotland]	2013	invasive (unspecified/other)	Neisseria meningitidis	B	B	B	19	1	1	83	4	4	10	2	NA	3	11	9		NA	4	4	2
35386	13.5556.G	UK [Scotland]	2013	invasive (unspecified/other)	Neisseria meningitidis	B	B	B	45	54	77	87	175	7	5	1	722	36	53	15	ST-213 complex	11224	NA	NA	3
35387	13.5557.N	UK [Scotland]	2013	invasive (unspecified/other)	Neisseria meningitidis	B	B	B	45	54	77	87	175	7	5	1	722	36	53	15	ST-213 complex	11224	NA	NA	3
35388	13.6067.H	UK [Scotland]	2013	invasive (unspecified/other)	Neisseria meningitidis	B	B	B	14	1	1	142	13	3	25	19	5	3	6	9	ST-41/44 complex	10806	13	3	1
35389	13.6117.K	UK [Scotland]	2013	invasive (unspecified/other)	Neisseria meningitidis	B	B	B	13	96	57	209	176	8	25	7	17	21	26	49	ST-1157 complex	1157	NA	NA	1
35390	13.6161.J	UK [Scotland]	2013	invasive (unspecified/other)	Neisseria meningitidis	B	B	B	14	7	1	142	6	10	6	9	5	9	6	9	ST-41/44 complex	42	6	2	1
35391	13.6352.Q	UK [Scotland]	2013	invasive (unspecified/other)	Neisseria meningitidis	Y	Y	Y	25	1	1	26	4	12	5	18	9	11	9	17	ST-23 complex	1655	4	4	2
35392	13.6756.W	UK [Scotland]	2013	invasive (unspecified/other)	Neisseria meningitidis	B	B	B	19	1	1	65	4	23	6	9	9	9	6	9	ST-41/44 complex	8931	4	4	2
35393	13.7214.J	UK [Scotland]	2013	invasive (unspecified/other)	Neisseria meningitidis	W	W	W	16	55	1	60	NA	11	5	18	8	78	24	21	ST-22 complex	1224	NA	NA	2
35394	13.7552.F	UK [Scotland]	2013	invasive (unspecified/other)	Neisseria meningitidis	B	B	B	4	2	1	84	2	3	6	9	5	9	6	9	ST-41/44 complex	41	2	3	1
35395	13.7896.B	UK [Scotland]	2013	invasive (unspecified/other)	Neisseria meningitidis	B	B	B	37	1	1	408	4	7	8	10	19	10	1	2	ST-18 complex	18	4	3	1
35396	13.8092.Q	UK [Scotland]	2013	invasive (unspecified/other)	Neisseria meningitidis	B	B	B	13	3	57	209	3	8	5	7	17	21	26	2	ST-1157 complex	3135	3	3	1
35397	13.8552.X	UK [Scotland]	2013	invasive (unspecified/other)	Neisseria meningitidis	W	W	W	22	1	1	1	4	2	3	4	3	8	4	6	ST-11 complex	11	4	4	2
51599	14,8701242	UK [Scotland]	2014	invasive (unspecified/other)	Neisseria meningitidis	W	W	W	22	1	1	1	4	2	3	4	3	8	4	6	ST-11 complex	11	4	4	2
51600	14,8701329	UK [Scotland]	2014	invasive (unspecified/other)	Neisseria meningitidis	Y	Y	Y	25	1	1	26	4	12	5	18	9	11	9	17	ST-23 complex	1655	4	4	2
51601	14,870165	UK [Scotland]	2014	invasive (unspecified/other)	Neisseria meningitidis	B	B	B	309	1	1	244	4	1	5	13	53	26	41	3	ST-162 complex	162	4	3	1
51602	14,8701672	UK [Scotland]	2014	invasive (unspecified/other)	Neisseria meningitidis	B	B	B	24	1	4	25	4	4	10	752	17	6	2	12	ST-35 complex	11015	4	4	2
51603	14,8701876	UK [Scotland]	2014	invasive (unspecified/other)	Neisseria meningitidis	B	B	B	309	1	1	244	4	1	5	13	53	26	41	3	ST-162 complex	162	4	3	1
51604	14,870206	UK [Scotland]	2014	invasive (unspecified/other)	Neisseria meningitidis	Y	Y	Y	25	1	1	26	4	12	5	18	9	11	9	17	ST-23 complex	1655	4	4	2
51605	14,8702529	UK [Scotland]	2014	invasive (unspecified/other)	Neisseria meningitidis	B	B	B	95	5	1	32	11	4	10	72	13	6	10	12	ST-35 complex	10821	11	2	2
51606	14,870322	UK [Scotland]	2014	invasive (unspecified/other)	Neisseria meningitidis	B	B	B	47	1	1	90	15	12	NA	NA	35	60	22	17		NA	15	4	3
51607	14,8703318	UK [Scotland]	2014	invasive (unspecified/other)	Neisseria meningitidis	W	W	W	16	1	1	60	4	11	5	18	8	11	4	21	ST-22 complex	184	4	4	2
51608	14,8703475	UK [Scotland]	2014	invasive (unspecified/other)	Neisseria meningitidis	W	W	W	22	1	1	1	4	2	3	4	3	8	4	6	ST-11 complex	11	4	4	2
51609	14,8703663	UK [Scotland]	2014	invasive (unspecified/other)	Neisseria meningitidis	B	B	B	95	5	1	32	11	4	10	72	9	6	10	12	ST-35 complex	2380	11	2	2
51610	14,8703675	UK [Scotland]	2014	invasive (unspecified/other)	Neisseria meningitidis	B	B	B	4	2	1	84	2	3	6	9	5	11	6	9	ST-41/44 complex	154	2	3	1
51611	14,8703916	UK [Scotland]	2014	invasive (unspecified/other)	Neisseria meningitidis	B	B	B	1056	249	1	919	190	4	10	2	5	3	11	9	ST-269 complex	1163	NA	NA	1
51612	14,8703919	UK [Scotland]	2014	invasive (unspecified/other)	Neisseria meningitidis	Y	Y	Y	25	1	1	26	4	10	5	18	9	11	9	17	ST-23 complex	23	4	4	2
51613	14,8703928	UK [Scotland]	2014	invasive (unspecified/other)	Neisseria meningitidis	C	C	C	22	1	1	1	4	2	3	4	3	8	4	6	ST-11 complex	11	4	4	2
52831	14,8704457	UK [Scotland]	2014	invasive (unspecified/other)	Neisseria meningitidis	Y	Y	Y	25	1	1	26	4	10	5	18	9	11	9	17	ST-23 complex	23	4	4	2
52832	14,870532	UK [Scotland]	2014	invasive (unspecified/other)	Neisseria meningitidis	Y		Y	0	1	NA	0	4	0	NA	NA	0	NA	NA	NA		NA	4	4	NA
52833	14,8705466	UK [Scotland]	2014	invasive (unspecified/other)	Neisseria meningitidis	Y	Y	Y	25	1	1	26	4	10	5	18	9	11	9	17	ST-23 complex	23	4	4	2
52834	14,8705522	UK [Scotland]	2014	invasive (unspecified/other)	Neisseria meningitidis	Y	Y	Y	25	1	1	26	4	12	5	18	9	11	9	17	ST-23 complex	1655	4	4	2
52835	14,8705635	UK [Scotland]	2014	invasive (unspecified/other)	Neisseria meningitidis	C	C	C	22	1	1	1	4	2	3	4	3	8	4	6	ST-11 complex	11	4	4	2
52836	14,8706298	UK [Scotland]	2014	invasive (unspecified/other)	Neisseria meningitidis	Y	Y	Y	25	1	1	26	4	12	5	18	9	11	9	17	ST-23 complex	1655	4	4	2
52837	14,870699	UK [Scotland]	2014	invasive (unspecified/other)	Neisseria meningitidis	W	W	W	22	1	1	1	4	2	3	4	3	8	4	6	ST-11 complex	11	4	4	2
52838	14,8707646	UK [Scotland]	2014	invasive (unspecified/other)	Neisseria meningitidis	W	W	W	22	1	1	1	4	2	3	4	3	8	4	6	ST-11 complex	11	4	4	2
42616	14-8704161	UK [Scotland]	2014	invasive (unspecified/other)	Neisseria meningitidis	B	B	B	19	1	1	83	4	4	10	2	5	38	11	8		3539	4	4	2
42617	14-8704253	UK [Scotland]	2014	invasive (unspecified/other)	Neisseria meningitidis	B	B	B	4	2	1	84	2	3	6	15	5	247	6	9	ST-41/44 complex	8539	2	3	1
42618	14-8704273	UK [Scotland]	2014	invasive (unspecified/other)	Neisseria meningitidis	B	B	B	4	2	1	84	2	3	6	15	5	247	6	9	ST-41/44 complex	8539	2	3	1
42619	14-8704599	UK [Scotland]	2014	invasive (unspecified/other)	Neisseria meningitidis	B	B	B	13	3	57	209	3	8	25	7	17	21	26	49	ST-1157 complex	1157	3	3	1
42620	14-8704743	UK [Scotland]	2014	invasive (unspecified/other)	Neisseria meningitidis	B	B	B	13	3	57	145	3	4	10	2	5	38	11	9	ST-269 complex	275	3	3	1
42621	14-8705223	UK [Scotland]	2014	invasive (unspecified/other)	Neisseria meningitidis	B	B	B	13	3	57	145	3	4	10	34	5	38	11	9	ST-269 complex	1161	3	3	1
42622	14-8705421	UK [Scotland]	2014	invasive (unspecified/other)	Neisseria meningitidis	B	B	B	25	1	1	26	4	8	5	15	17	9	21	2	ST-865 complex	11103	4	4	2
42623	14-8705521	UK [Scotland]	2014	invasive (unspecified/other)	Neisseria meningitidis	B	B	B	95	5	1	32	11	4	10	72	9	6	10	12	ST-35 complex	2380	11	2	2
42624	14-8705636	UK [Scotland]	2014	invasive (unspecified/other)	Neisseria meningitidis	B	B	B	224	15	1	252	68	4	10	5	4	6	3	8	ST-32 complex	32	NA	NA	1
42625	14-8706036	UK [Scotland]	2014	invasive (unspecified/other)	Neisseria meningitidis	B	B	B	45	8	77	87	20	7	5	1	13	36	53	15	ST-213 complex	213	20	4	3
42626	14-8706144	UK [Scotland]	2014	invasive (unspecified/other)	Neisseria meningitidis	B	B	B	15	4	2	193	1	4	10	15	9	8	11	9	ST-269 complex	269	1	1	1
42627	14-8706349	UK [Scotland]	2014	invasive (unspecified/other)	Neisseria meningitidis	B	B	B	37	1	1	408	4	7	8	10	19	10	1	2	ST-18 complex	18	4	3	1
42628	14-8706436	UK [Scotland]	2014	invasive (unspecified/other)	Neisseria meningitidis	B	B	B	15	4	2	193	1	4	10	15	17	8	11	17	ST-269 complex	479	1	1	1
42629	14-8706586	UK [Scotland]	2014	invasive (unspecified/other)	Neisseria meningitidis	B	B	B	19	1	1	83	4	4	10	2	5	3	11	9	ST-269 complex	1163	4	4	2
42630	14-8707385	UK [Scotland]	2014	invasive (unspecified/other)	Neisseria meningitidis	B	B	B	4	2	1	84	2	3	6	9	10	9	6	9	ST-41/44 complex	5550	2	3	1
42631	14-8707662	UK [Scotland]	2014	invasive (unspecified/other)	Neisseria meningitidis	B	B	B	13	3	57	145	3	4	10	34	5	38	11	9	ST-269 complex	1161	3	3	1
42446	15 8704767	UK [Scotland]	2015	invasive (unspecified/other)	Neisseria meningitidis	B	B	B	13	3	1	145	3	7	5	1	13	82	7	15	ST-213 complex	6607	3	3	1
38995	15,8700107	UK [Scotland]	2015	invasive (unspecified/other)	Neisseria meningitidis	W	W	W	22	1	1	1	4	NA	3	4	3	8	4	6		NA	4	4	2
38996	15,8700914	UK [Scotland]	2015	invasive (unspecified/other)	Neisseria meningitidis	W	W	W	22	1	1	1	4	2	3	4	3	8	4	6	ST-11 complex	11	4	4	2
38997	15,8701412	UK [Scotland]	2015	invasive (unspecified/other)	Neisseria meningitidis	W	W	W	22	1	1	1	4	2	3	4	3	8	4	6	ST-11 complex	11	4	4	2
52839	15,870305	UK [Scotland]	2015	invasive (unspecified/other)	Neisseria meningitidis	Y	Y	Y	25	1	1	26	4	12	5	18	9	11	9	17	ST-23 complex	1655	4	4	2
38998	15,8703241	UK [Scotland]	2015	invasive (unspecified/other)	Neisseria meningitidis	W	W	W	22	1	1	1	4	2	3	4	3	8	4	6	ST-11 complex	11	4	4	2
38999	15,8703378	UK [Scotland]	2015	invasive (unspecified/other)	Neisseria meningitidis	W	W	W	22	1	1	1	4	2	3	4	3	8	4	6	ST-11 complex	11	4	4	2
39000	15,8703957	UK [Scotland]	2015	invasive (unspecified/other)	Neisseria meningitidis	W	W	W	22	1	1	1	4	2	3	4	3	8	4	6	ST-11 complex	11	4	4	2
52840	15,8704035	UK [Scotland]	2015	invasive (unspecified/other)	Neisseria meningitidis	C	C	C	15	4	2	193	1	4	NA	15	NA	8	11	NA		NA	1	1	1
39001	15,8705087	UK [Scotland]	2015	invasive (unspecified/other)	Neisseria meningitidis	W	W	W	22	1	1	1	4	2	3	4	3	8	4	6	ST-11 complex	11	4	4	2
52842	15,8705782	UK [Scotland]	2015	invasive (unspecified/other)	Neisseria meningitidis	Y	Y	Y	25	1	1	26	4	12	5	18	9	11	9	17	ST-23 complex	1655	4	4	2
38306	15,8705948	UK [Scotland]	2015	invasive (unspecified/other)	Neisseria meningitidis	W	W	W	22	1	1	1	4	2	3	4	3	8	4	6	ST-11 complex	11	4	4	2
38307	15,8706012	UK [Scotland]	2015	invasive (unspecified/other)	Neisseria meningitidis	W	W	W	22	1	1	1	4	2	3	4	3	8	4	6	ST-11 complex	11	4	4	2
38308	15,870609	UK [Scotland]	2015	invasive (unspecified/other)	Neisseria meningitidis	W	W	W	22	1	1	1	4	2	3	4	3	8	4	6	ST-11 complex	11	4	4	2
38309	15,8706113	UK [Scotland]	2015	invasive (unspecified/other)	Neisseria meningitidis	W	W	W	22	1	1	1	4	2	3	4	3	8	4	6	ST-11 complex	11	4	4	2
52843	15,8706232	UK [Scotland]	2015	invasive (unspecified/other)	Neisseria meningitidis	Y	Y	Y	25	1	1	26	4	12	5	18	9	11	9	17	ST-23 complex	1655	4	4	2
52844	15,8706311	UK [Scotland]	2015	invasive (unspecified/other)	Neisseria meningitidis	Y	Y	Y	25	1	1	26	4	10	5	18	9	11	9	17	ST-23 complex	23	4	4	2
38417	15,8706763	UK [Scotland]	2015	invasive (unspecified/other)	Neisseria meningitidis	Y	Y	Y	19	1	1	93	13	2	16	6	17	9	11	2		11520	13	4	2
52845	15,8707666	UK [Scotland]	2015	invasive (unspecified/other)	Neisseria meningitidis	W	W	W	22	1	1	1	4	2	3	4	3	8	4	6	ST-11 complex	11	4	4	2
52846	15,8708749	UK [Scotland]	2015	invasive (unspecified/other)	Neisseria meningitidis	W	W	W	22	1	1	1	4	2	3	4	3	8	4	6	ST-11 complex	11	4	4	2
52847	15,8708877	UK [Scotland]	2015	invasive (unspecified/other)	Neisseria meningitidis	W	W	W	22	1	1	1	4	2	3	4	3	8	4	6	ST-11 complex	11	4	4	2
52848	15,8709301	UK [Scotland]	2015	invasive (unspecified/other)	Neisseria meningitidis	W	W	W	22	1	1	1	4	2	3	4	3	8	4	6	ST-11 complex	11	4	4	2
42632	15-8700108	UK [Scotland]	2015	invasive (unspecified/other)	Neisseria meningitidis	B	B	B	18	1	4	67	4	706	3	13	7	715	41	77		11105	4	4	2
42633	15-8700319	UK [Scotland]	2015	invasive (unspecified/other)	Neisseria meningitidis	B	B	B	21	1	1	7	4	1	5	13	53	26	41	3	ST-162 complex	162	4	4	2
42634	15-8700456	UK [Scotland]	2015	invasive (unspecified/other)	Neisseria meningitidis	B	B	B	15	4	2	231	1	4	10	15	17	8	11	9	ST-269 complex	1049	1	1	1
42635	15-8700522	UK [Scotland]	2015	invasive (unspecified/other)	Neisseria meningitidis	B	B	B	13	3	57	145	3	4	10	2	5	38	11	9	ST-269 complex	275	3	3	1
42636	15-8700571	UK [Scotland]	2015	invasive (unspecified/other)	Neisseria meningitidis	B	B	B	13	3	57	145	3	NA	10	2	5	38	11	9		NA	3	3	1
42637	15-8700655	UK [Scotland]	2015	invasive (unspecified/other)	Neisseria meningitidis	B	B	B	15	4	2	193	1	4	10	15	17	8	11	17	ST-269 complex	479	1	1	1
42639	15-8700842	UK [Scotland]	2015	invasive (unspecified/other)	Neisseria meningitidis	B	B	B	19	1	1	83	4	4	10	2	5	38	6	9		3068	4	4	2
42640	15-8701359	UK [Scotland]	2015	invasive (unspecified/other)	Neisseria meningitidis	B	B	B	4	2	1	84	2	3	6	9	0	8	6	9		NA	2	3	1
42641	15-8701697	UK [Scotland]	2015	invasive (unspecified/other)	Neisseria meningitidis	B	B	B	45	61	77	87	148	7	5	1	9	36	53	15	ST-213 complex	9193	NA	NA	3
42642	15-8701782	UK [Scotland]	2015	invasive (unspecified/other)	Neisseria meningitidis	B	B	B	15	4	2	193	1	4	25	15	9	8	11	9	ST-269 complex	11140	1	1	1
42643	15-8701871	UK [Scotland]	2015	invasive (unspecified/other)	Neisseria meningitidis	B	B	B	19	1	1	83	4	4	10	2	5	3	11	9	ST-269 complex	1163	4	4	2
42644	15-8702724	UK [Scotland]	2015	invasive (unspecified/other)	Neisseria meningitidis	B	B	B	4	2	1	84	2	3	6	9	5	8	6	9	ST-41/44 complex	485	2	3	1
42645	15-8703451	UK [Scotland]	2015	invasive (unspecified/other)	Neisseria meningitidis	B	B	B	47	1	57	90	15	12	5	12	35	192	22	17	ST-461 complex	1946	15	4	3
42646	15-8703750	UK [Scotland]	2015	invasive (unspecified/other)	Neisseria meningitidis	B	B	B	1	6	3	100	7	8	10	5	4	6	3	8	ST-32 complex	33	7	1	1
42647	15-8704692	UK [Scotland]	2015	invasive (unspecified/other)	Neisseria meningitidis	B	B	B	13	3	1	145	3	7	5	1	13	82	7	15	ST-213 complex	6607	3	3	1
42648	15-8704767	UK [Scotland]	2015	invasive (unspecified/other)	Neisseria meningitidis	B	B	B	13	3	1	145	3	7	5	1	13	82	7	15	ST-213 complex	6607	3	3	1
42649	15-8705815	UK [Scotland]	2015	invasive (unspecified/other)	Neisseria meningitidis	B	B	B	15	4	2	193	1	4	10	6	9	21	732	9	ST-269 complex	11238	1	1	1
44681	15-8707130	UK [Scotland]	2015	invasive (unspecified/other)	Neisseria meningitidis	B	B	B	309	1	1	244	4	1	5	13	53	26	41	3	ST-162 complex	162	4	3	1
44680	15-8708102	UK [Scotland]	2015	invasive (unspecified/other)	Neisseria meningitidis	B	B	B	13	3	57	209	3	8	25	7	17	21	26	49	ST-1157 complex	1157	3	3	1
42650	15-8708141	UK [Scotland]	2015	invasive (unspecified/other)	Neisseria meningitidis	B	B	B	14	7	1	142	6	10	6	9	5	9	6	9	ST-41/44 complex	42	6	2	1
44682	15-8708162	UK [Scotland]	2015	invasive (unspecified/other)	Neisseria meningitidis	B	B	B	13	3	1	145	3	7	5	1	13	82	18	15	ST-213 complex	11601	3	3	1
44683	15-8708164	UK [Scotland]	2015	invasive (unspecified/other)	Neisseria meningitidis	B	B	B	950	113	1	722	131	8	25	7	17	21	24	49	ST-1157 complex	1727	NA	NA	3
42651	15-8708206	UK [Scotland]	2015	invasive (unspecified/other)	Neisseria meningitidis	B	B	B	4	2	1	84	2	3	132	9	5	9	6	9	ST-41/44 complex	8601	2	3	1
42652	15-8708361	UK [Scotland]	2015	invasive (unspecified/other)	Neisseria meningitidis	B	B	B	45	14	77	87	45	7	5	1	13	36	53	15	ST-213 complex	213	NA	NA	3
42653	15-8708563	UK [Scotland]	2015	invasive (unspecified/other)	Neisseria meningitidis	B	B	B	15	4	2	193	1	4	10	15	9	8	11	9	ST-269 complex	269	1	1	1
42654	15-8708670	UK [Scotland]	2015	invasive (unspecified/other)	Neisseria meningitidis	B	B	B	15	4	2	231	1	NA	10	15	17	8	11	9		NA	1	1	1
662	2837	UK	1997	invasive (unspecified/other)	Neisseria meningitidis	C	C	C	95	5	1	32	11	2	3	19	3	8	4	6	ST-11 complex	50	11	2	2
41785	2838	UK	1997	carrier	Neisseria meningitidis	C	C	C	NA	5	1	917	11	2	3	4	3	8	4	6	ST-11 complex	11	11	2	NA
41784	2839	UK	1997	invasive (unspecified/other)	Neisseria meningitidis	C	C	C	110	21	1	171	19	2	3	4	3	8	4	6	ST-11 complex	11	NA	NA	1
670	2840	UK	1997	invasive (unspecified/other)	Neisseria meningitidis	C	C	C	95	5	1	32	11	2	3	19	3	8	4	6	ST-11 complex	50	11	2	2
667	2842	UK	1997	carrier	Neisseria meningitidis	C	C	C	22	1	1	1	4	2	3	4	23	8	6	6	ST-11 complex	51	4	4	2
666	2843	UK	1997	carrier	Neisseria meningitidis	C	C	C	22	1	1	1	4	2	3	4	23	8	6	6	ST-11 complex	51	4	4	2
671	2844	UK	1997	invasive (unspecified/other)	Neisseria meningitidis	C	C	C	95	5	1	32	11	2	3	19	3	8	4	6	ST-11 complex	50	11	2	2
665	2845	UK	1997	carrier	Neisseria meningitidis	C	C	C	22	1	1	1	4	2	3	4	24	8	4	6	ST-11 complex	67	4	4	2
642	312 901	UK [England]	1996	invasive (unspecified/other)	Neisseria meningitidis	C	C	C	16	1	4	59	4	2	3	7	2	8	5	2	ST-8 complex	8	4	4	2
38305	88/03415	UK [Scotland]	1988	invasive (unspecified/other)	Neisseria meningitidis	B	B	B	14	163	1	142	146	3	6	9	5	3	6	9	ST-41/44 complex	46	NA	NA	1
51145	B003	UK [England]	2015	carrier	Neisseria meningitidis		W	W	22	1	1	1	4	2	3	4	3	8	4	6	ST-11 complex	11	4	4	2
54403	B005	UK [England]	2015	carrier	Neisseria meningitidis		W	W	22	1	1	1	4	2	3	4	3	8	4	6	ST-11 complex	11	4	4	2
51146	B009	UK [England]	2015	carrier	Neisseria meningitidis		W	W	22	1	1	1	4	2	3	4	3	8	4	6	ST-11 complex	11	4	4	2

54405	B018	UK [England]	2015	carrier	Neisseria meningitidis		W	W	22	1	1	1	4	2	3	4	3	8	4	6	ST-11 complex	11	4	4	2
51147	B021	UK [England]	2015	carrier	Neisseria meningitidis		W	W	22	1	1	1	4	2	3	4	3	8	4	6	ST-11 complex	11	4	4	2
51215	B025	UK [England]	2015	carrier	Neisseria meningitidis		cnl	cnl	4	1	1	206	4	5	4	17	15	30	7	12	ST-198 complex	823	4	3	1
51216	B044	UK [England]	2015	carrier	Neisseria meningitidis		cnl	cnl	4	1	1	206	4	5	4	17	15	30	7	12	ST-198 complex	823	4	3	1
54412	B064	UK [England]	2015	carrier	Neisseria meningitidis		W	W	22	1	1	1	4	2	3	4	3	8	4	6	ST-11 complex	11	4	4	2
51221	B073	UK [England]	2015	carrier	Neisseria meningitidis		NG	NG	16	1	4	59	4	4	10	11	18	6	10	12	ST-35 complex	35	4	4	2
51222	B081	UK [England]	2015	carrier	Neisseria meningitidis		cnl	cnl	4	1	1	206	4	5	4	17	15	30	7	12	ST-198 complex	823	4	3	1
51223	B083	UK [England]	2015	carrier	Neisseria meningitidis		cnl	cnl	4	1	1	206	4	5	4	17	17	30	7	12	ST-198 complex	2384	4	3	1
51148	B091	UK [England]	2015	carrier	Neisseria meningitidis		W	W	22	1	1	1	4	2	3	4	3	8	4	6	ST-11 complex	11	4	4	2
51149	B100	UK [England]	2015	carrier	Neisseria meningitidis		W	W	16	1	1	60	4	11	5	901	8	11	24	21	ST-22 complex	13387	4	4	2
51226	B101	UK [England]	2015	carrier	Neisseria meningitidis		B	B	16	1	4	59	4	4	10	11	18	3	10	12	ST-35 complex	1417	4	4	2
54421	B103	UK [England]	2015	carrier	Neisseria meningitidis		W	W	22	1	1	1	4	2	3	4	3	8	4	6	ST-11 complex	11	4	4	2
51150	B110	UK [England]	2015	carrier	Neisseria meningitidis		W	W	22	1	1	1	4	662	3	4	3	8	4	6	ST-11 complex	10651	4	4	2
54422	B114	UK [England]	2015	carrier	Neisseria meningitidis		W	W	22	1	1	1	4	2	3	4	3	8	4	6	ST-11 complex	11	4	4	2
51229	B119	UK [England]	2015	carrier	Neisseria meningitidis		Y	Y	25	1	1	26	4	10	5	18	9	11	9	17	ST-23 complex	23	4	4	2
50242	BR40013	UK [England]	2014	carrier	Neisseria meningitidis	B	B	B	14	1	57	142	13	6	5	2	151	12	11	14		1976	13	3	1
52539	BR40021	UK [England]	2014	carrier	Neisseria meningitidis	NG	NG	NG	13	3	57	209	3	8	25	7	17	21	26	49	ST-1157 complex	1157	3	3	1
50244	BR40044	UK [England]	2014	carrier	Neisseria meningitidis	NG	Y	Y	25	1	1	26	4	12	5	18	9	11	9	17	ST-23 complex	1655	4	4	2
43567	BR40069	UK [England]	2014	carrier	Neisseria meningitidis	NG	E	E	12	258	57	266	NA	7	16	55	10	3	56	46	ST-178 complex	178	NA	NA	1
50245	BR40076	UK [England]	2014	carrier	Neisseria meningitidis	B	B	B	45	8	77	87	20	7	5	1	13	36	53	15	ST-213 complex	213	20	4	3
43569	BR40099	UK [England]	2014	carrier	Neisseria meningitidis	B	B	B	45	8	77	87	20	7	5	1	13	36	53	15	ST-213 complex	213	20	4	3
43571	BR40110	UK [England]	2014	carrier	Neisseria meningitidis	NG	cnl	cnl	102	8	1	2	20	16	2	6	25	17	25	22	ST-53 complex	53	20	4	2
52540	BR40152	UK [England]	2014	carrier	Neisseria meningitidis	NG	NG	NG	16	1	1	60	4	2	7	6	17	5	18	8	ST-167 complex	3039	4	4	2
50247	BR40158	UK [England]	2014	carrier	Neisseria meningitidis	B	B	B	45	8	77	87	20	7	5	1	13	36	53	15	ST-213 complex	213	20	4	3
43575	BR40196	UK [England]	2014	carrier	Neisseria meningitidis	NG	E	E	13	3	57	209	3	8	25	7	17	21	26	49	ST-1157 complex	1157	3	3	1
50248	BR40197	UK [England]	2014	carrier	Neisseria meningitidis	NG	Y	Y	22	1	1	1	4	2	3	4	3	8	4	6	ST-11 complex	11	4	4	2
50249	BR40250	UK [England]	2014	carrier	Neisseria meningitidis	NG	Y	Y	25	1	1	26	4	12	5	18	9	11	9	17	ST-23 complex	1655	4	4	2
50251	BR40257	UK [England]	2014	carrier	Neisseria meningitidis	Y	Y	Y	25	1	1	26	4	12	5	18	9	11	9	17	ST-23 complex	1655	4	4	2
43580	BR40263	UK [England]	2014	carrier	Neisseria meningitidis	NG	B	B	45	8	77	87	20	7	5	1	13	36	53	15	ST-213 complex	213	20	4	3
50252	BR40285	UK [England]	2014	carrier	Neisseria meningitidis	NG	Y	Y	25	1	1	26	4	12	5	18	9	11	9	17	ST-23 complex	1655	4	4	2
50253	BR40286	UK [England]	2014	carrier	Neisseria meningitidis	NG	B	B	302	24	1	121	55	9	20	9	9	9	6	9	ST-41/44 complex	9200	NA	NA	2
50254	BR40312	UK [England]	2014	carrier	Neisseria meningitidis	Y	Y	Y	25	1	1	26	4	12	5	18	9	11	9	17	ST-23 complex	1655	4	4	2
43586	BR40314	UK [England]	2014	carrier	Neisseria meningitidis	NG	cnl	cnl	21	1	1	7	4	16	2	159	92	77	25	112	ST-1117 complex	1117	4	4	2
52541	BR40329	UK [England]	2014	carrier	Neisseria meningitidis	NG	B	B	13	3	57	209	3	8	25	7	17	21	26	3	ST-1157 complex	12055	3	3	1
43588	BR40346	UK [England]	2014	carrier	Neisseria meningitidis	NG	E	E	13	3	57	209	3	8	25	7	17	21	26	49	ST-1157 complex	1157	3	3	1
43590	BR40359	UK [England]	2014	carrier	Neisseria meningitidis	NG	E	E	13	96	57	209	176	6	25	7	17	21	26	49	ST-1157 complex	12056	NA	NA	1
50255	BR40378	UK [England]	2014	carrier	Neisseria meningitidis	NG	cnl	cnl	102	8	10	2	20	16	2	6	25	17	25	22	ST-53 complex	53	20	4	2
43548	BR40421	UK [England]	2014	carrier	Neisseria meningitidis	NG	cnl	cnl	102	8	10	2	20	16	2	6	25	17	25	22	ST-53 complex	53	20	4	2
43595	BR40431	UK [England]	2014	carrier	Neisseria meningitidis	NG	cnl	cnl	94	138	56	69	NA	5	4	17	15	14	7	12	ST-198 complex	198	NA	NA	3
43596	BR40454	UK [England]	2014	carrier	Neisseria meningitidis	NG	B	B	19	1	4	83	4	4	10	161	9	6	21	3		1167	4	4	2
50256	BR40458	UK [England]	2014	carrier	Neisseria meningitidis	NG	B	B	4	NA	1	84	NA	3	6	9	5	9	6	9	ST-41/44 complex	41	NA	NA	1
43599	BR40459	UK [England]	2014	carrier	Neisseria meningitidis	NG	B	B	302	24	1	121	55	9	20	9	9	9	6	9	ST-41/44 complex	9200	NA	NA	2
43600	BR40460	UK [England]	2014	carrier	Neisseria meningitidis	NG	B	B	302	24	1	121	55	9	20	9	9	9	6	9	ST-41/44 complex	9200	NA	NA	2
50857	BR40482	UK [England]	2014	carrier	Neisseria meningitidis	NG	E	E	13	3	57	209	3	8	25	7	17	21	26	49	ST-1157 complex	1157	3	3	1
43603	BR40485	UK [England]	2014	carrier	Neisseria meningitidis	NG	E	E	13	3	57	209	3	8	25	7	17	21	26	49	ST-1157 complex	1157	3	3	1
50258	BR40490	UK [England]	2014	carrier	Neisseria meningitidis	NG	cnl	cnl	102	8	1	2	20	16	2	6	25	17	25	22	ST-53 complex	53	20	4	2
50259	BR40491	UK [England]	2014	carrier	Neisseria meningitidis	NG	E	E	13	3	57	209	3	8	25	7	17	21	26	49	ST-1157 complex	1157	3	3	1
50260	BR40494	UK [England]	2014	carrier	Neisseria meningitidis	NG	cnl	cnl	102	8	1	2	20	16	2	6	25	17	25	22	ST-53 complex	53	20	4	2
43732	BR40497	UK [England]	2014	carrier	Neisseria meningitidis	NG	E	E	13	3	57	209	3	8	25	7	17	21	26	49	ST-1157 complex	1157	3	3	1
43733	BR40498	UK [England]	2014	carrier	Neisseria meningitidis	B	B	B	47	1	57	90	15	12	5	12	35	60	22	17	ST-461 complex	461	15	4	3
50261	BR40499	UK [England]	2014	carrier	Neisseria meningitidis	W	W	W	22	1	1	1	4	662	3	4	3	8	4	6	ST-11 complex	10651	4	4	2
50262	BR40503	UK [England]	2014	carrier	Neisseria meningitidis	B	B	B	14	1	57	142	13	6	5	105	12	6	13	17		4954	13	3	1
50263	BR40510	UK [England]	2014	carrier	Neisseria meningitidis	W	W	W	22	1	1	1	4	662	3	4	3	8	4	6	ST-11 complex	10651	4	4	2
43738	BR40533	UK [England]	2014	carrier	Neisseria meningitidis	NG	E	E	13	3	57	209	3	205	25	7	17	21	26	49	ST-1157 complex	12067	3	3	1
43739	BR40598	UK [England]	2014	carrier	Neisseria meningitidis	NG	cnl	cnl	102	8	10	2	20	16	2	6	25	17	25	22	ST-53 complex	53	20	4	2
50264	BR40614	UK [England]	2014	carrier	Neisseria meningitidis	Y	Y	Y	25	1	1	26	4	12	286	18	9	11	9	17	ST-23 complex	6463	4	4	2
43743	BR40622	UK [England]	2014	carrier	Neisseria meningitidis	NG	E	E	13	3	57	209	3	8	25	7	17	21	26	49	ST-1157 complex	1157	3	3	1
44111	BR40627	UK [England]	2014	carrier	Neisseria meningitidis	W	W	W	22	1	1	1	4	2	3	4	3	8	4	6	ST-11 complex	11	4	4	2
44112	BR40632	UK [England]	2014	carrier	Neisseria meningitidis	B	B	B	45	8	77	87	20	7	5	1	13	36	53	15	ST-213 complex	213	20	4	3
43745	BR40670	UK [England]	2014	carrier	Neisseria meningitidis	B	B	B	45	8	77	87	20	7	5	1	13	783	53	15	ST-213 complex	12146	20	4	3
43748	BR40748	UK [England]	2014	carrier	Neisseria meningitidis	NG	E	E	13	3	1	145	3	175	5	484	17	3	4	124		6798	3	3	1
43749	BR40753	UK [England]	2014	carrier	Neisseria meningitidis	NG	B	B	16	1	4	681	4	4	10	11	17	6	10	12	ST-35 complex	457	4	4	2
44115	BR40761	UK [England]	2014	carrier	Neisseria meningitidis	NG	Y	Y	24	1	1	27	4	2	7	6	17	9	6	8	ST-167 complex	1624	4	4	2
52542	BR40765	UK [England]	2014	carrier	Neisseria meningitidis	NG	NG	NG	16	1	4	59	4	4	10	11	18	6	10	12	ST-35 complex	35	4	4	2
44116	BR40767	UK [England]	2014	carrier	Neisseria meningitidis	NG	E	E	13	3	1	209	3	8	25	7	17	21	26	49	ST-1157 complex	1157	3	3	1
50266	BR40804	UK [England]	2014	carrier	Neisseria meningitidis	NG	cnl	cnl	21	1	1	7	4	16	2	159	92	77	25	112	ST-1117 complex	1117	4	4	2
43776	BR40843	UK [England]	2014	carrier	Neisseria meningitidis	B	B	B	106	1	1	146	4	4	10	15	9	8	24	16	ST-269 complex	12071	4	4	2
43777	BR40912	UK [England]	2014	carrier	Neisseria meningitidis	NG	E	E	13	3	57	209	3	8	25	7	17	21	26	49	ST-1157 complex	1157	3	3	1
52543	BR40934	UK [England]	2014	carrier	Neisseria meningitidis	B	B	B	45	8	77	87	20	7	5	1	13	36	53	15	ST-213 complex	213	20	4	3
43779	BR40955	UK [England]	2014	carrier	Neisseria meningitidis	NG	cnl	cnl	4	1	1	206	4	5	4	17	15	30	7	12	ST-198 complex	823	4	3	1
43783	BR40979	UK [England]	2014	carrier	Neisseria meningitidis	NG	cnl	cnl	102	8	10	2	20	16	2	6	25	17	25	22	ST-53 complex	53	20	4	2
50267	BR40981	UK [England]	2014	carrier	Neisseria meningitidis	Y	Y	Y	23	1	1	4	4	2	7	159	92	93	6	8		6464	4	4	2
50268	BR40999	UK [England]	2014	carrier	Neisseria meningitidis	NG	E	E	13	3	57	209	3	8	25	7	17	21	26	49	ST-1157 complex	1157	3	3	1
43789	BR41090	UK [England]	2014	carrier	Neisseria meningitidis	NG	E	E	13	3	57	209	3	8	25	7	17	21	26	49	ST-1157 complex	1157	3	3	1
50269	BR41099	UK [England]	2014	carrier	Neisseria meningitidis	NG	E	E	13	3	57	209	3	8	25	7	17	21	26	49	ST-1157 complex	1157	3	3	1
50270	BR41121	UK [England]	2014	carrier	Neisseria meningitidis	Y	Y	Y	25	1	1	26	4	12	5	18	9	11	9	17	ST-23 complex	1655	4	4	2
52544	BR41123	UK [England]	2014	carrier	Neisseria meningitidis	B	B	B	47	1	57	90	15	12	5	12	35	2	22	17	ST-461 complex	12058	15	4	3
43631	BR41131	UK [England]	2014	carrier	Neisseria meningitidis	NG	B	B	21	1	1	7	4	4	10	5	4	6	3	8	ST-32 complex	32	4	4	2
43632	BR41150	UK [England]	2014	carrier	Neisseria meningitidis	NG	cnl	cnl	102	8	1	2	20	16	2	6	25	17	25	22	ST-53 complex	53	20	4	2
52545	BR41160	UK [England]	2014	carrier	Neisseria meningitidis	NG	B	B	45	8	77	87	20	7	5	1	13	36	53	15	ST-213 complex	213	20	4	3
43551	BR41173	UK [England]	2014	carrier	Neisseria meningitidis	NG	B	B	16	1	4	59	4	4	10	136	17	5	789	12		12137	4	4	2
43552	BR41176	UK [England]	2014	carrier	Neisseria meningitidis	NG	E	E	13	3	1	145	3	17	5	19	17	62	26	2	ST-60 complex	10454	3	3	1
43553	BR41204	UK [England]	2014	carrier	Neisseria meningitidis	NG	cnl	cnl	102	8	10	2	20	16	2	6	25	777	25	22	ST-53 complex	12138	20	4	2
52546	BR41208	UK [England]	2014	carrier	Neisseria meningitidis	B	B	B	45	8	1	87	20	7	5	1	13	11	53	15	ST-213 complex	3739	20	4	3
52547	BR41219	UK [England]	2014	carrier	Neisseria meningitidis	NG	C	C	15	NA	2	193	NA	4	10	15	9	8	11	13	ST-269 complex	467	NA	NA	1
43558	BR41225	UK [England]	2014	carrier	Neisseria meningitidis	NG	E	E	13	3	57	209	3	8	25	7	17	21	26	49	ST-1157 complex	1157	3	3	1
50271	BR41252	UK [England]	2014	carrier	Neisseria meningitidis	B	B	B	14	1	1	142	13	3	6	19	5	3	6	9	ST-41/44 complex	340	13	3	1
50272	BR41257	UK [England]	2014	carrier	Neisseria meningitidis	NG	E	E	13	3	57	209	3	8	25	7	17	21	26	49	ST-1157 complex	1157	3	3	1
43561	BR41272	UK [England]	2014	carrier	Neisseria meningitidis	NG	cnl	cnl	334	1	1	527	4	6	41	108	15	9	6	9		845	4	4	2
43564	BR41285	UK [England]	2015	carrier	Neisseria meningitidis	NG	B	B	16	1	4	59	4	4	10	11	18	6	10	12	ST-35 complex	35	4	4	2
50273	BR41292	UK [England]	2015	carrier	Neisseria meningitidis	NG	B	B	510	28	8	599	30	4	10	48	4	6	3	8	ST-32 complex	7460	NA	NA	1
52548	BR41294	UK [England]	2015	carrier	Neisseria meningitidis	NG	B	B	16	1	4	59	4	4	10	11	18	6	10	12	ST-35 complex	35	4	4	2
43610	BR41298	UK [England]	2015	carrier	Neisseria meningitidis	NG	B	B	1	6	3	100	7	8	10	5	4	6	3	8	ST-32 complex	33	7	1	1
50274	BR41313	UK [England]	2015	carrier	Neisseria meningitidis	W	W	W	22	1	1	1	4	2	3	4	3	8	4	6	ST-11 complex	11	4	4	2
52549	BR41314	UK [England]	2015	carrier	Neisseria meningitidis	NG	NG	NG	13	3	57	209	3	8	25	7	17	21	26	49	ST-1157 complex	1157	3	3	1
43614	BR41315	UK [England]	2015	carrier	Neisseria meningitidis	NG	B	B	16	1	4	59	4	4	10	11	18	6	10	12	ST-35 complex	35	4	4	2
43615	BR41338	UK [England]	2015	carrier	Neisseria meningitidis	NG	B	B	16	1	4	59	4	4	10	11	18	6	10	12	ST-35 complex	35	4	4	2
43616	BR41347	UK [England]	2015	carrier	Neisseria meningitidis	NG	B	B	16	1	4	59	4	4	10	11	18	6	10	12	ST-35 complex	35	4	4	2
52550	BR41378	UK [England]	2015	carrier	Neisseria meningitidis	NG	B	B	525	98	1	457	122	9	6	9	1	8	6	6	ST-41/44 complex	7516	NA	NA	1
50276	BR41389	UK [England]	2015	carrier	Neisseria meningitidis	NG	NG	NG	13	3	57	209	3	8	25	7	17	21	26	49	ST-1157 complex	1157	3	3	1
43623	BR41396	UK [England]	2015	carrier	Neisseria meningitidis	W	W	W	22	1	1	1	4	2	3	4	3	8	4	6	ST-11 complex	11	4	4	2
43624	BR41407	UK [England]	2015	carrier	Neisseria meningitidis	NG	B	B	525	98	1	457	122	9	6	9	1	8	6	6	ST-41/44 complex	7516	NA	NA	1
50277	BR41408	UK [England]	2015	carrier	Neisseria meningitidis	W	W	W	22	1	1	1	4	2	3	4	3	8	4	6	ST-11 complex	11	4	4	2
43626	BR41423	UK [England]	2015	carrier	Neisseria meningitidis	NG	B	B	45	8	77	87	20	7	25	1	13	36	26	15	ST-213 complex	12057	20	4	3
43627	BR41426	UK [England]	2015	carrier	Neisseria meningitidis	B	B	B	16	1	4	681	4	4	10	11	17	6	10	12	ST-35 complex	457	4	4	2
43634	BR41438	UK [England]	2015	carrier	Neisseria meningitidis	NG	B	B	4	2	1	84	2	3	6	9	5	9	6	2	ST-41/44 complex	2314	2	3	1
52552	BR41445	UK [England]	2015	carrier	Neisseria meningitidis	Y	Y	Y	31	9	1	58	16	12	5	18	9	11	9	17	ST-23 complex	1655	16	2	3
52553	BR41449	UK [England]	2015	carrier	Neisseria meningitidis	Y	Y	Y	25	1	1	26	4	12	5	18	9	11	9	17	ST-23 complex	1655	4	4	2
50278	BR41452	UK [England]	2015	carrier	Neisseria meningitidis	Y	Y	Y	25	1	1	26	4	12	5	18	9	11	9	17	ST-23 complex	1655	4	4	2
50279	BR41454	UK [England]	2015	carrier	Neisseria meningitidis	Y	Y	Y	25	1	1	26	4	12	5	18	9	11	9	17	ST-23 complex	1655	4	4	2
52554	BR41456	UK [England]	2015	carrier	Neisseria meningitidis	Y	Y	Y	24	1	1	27	4	2	7	6	17	9	6	8	ST-167 complex	1624	4	4	2
52555	BR41465	UK [England]	2015	carrier	Neisseria meningitidis	W	W	W	22	1	1	1	4	2	3	4	3	8	4	6	ST-11 complex	11	4	4	2
43795	BR41481	UK [England]	2015	carrier	Neisseria meningitidis	NG	cnl	cnl	102	8	10	2	20	16	2	6	25	17	25	22	ST-53 complex	53	20	4	2
43641	BR41483	UK [England]	2015	carrier	Neisseria meningitidis	NG	E	E	12	258	57	266	NA	7	16	55	10	3	56	46	ST-178 complex	178	NA	NA	1
43642	BR41486	UK [England]	2015	carrier	Neisseria meningitidis	NG	E	E	13	3	57	209	3	8	25	7	17	21	26	49	ST-1157 complex	1157	3	3	1
50280	BR41490	UK [England]	2015	carrier	Neisseria meningitidis	Y	Y	Y	25	1	1	26	4	12	5	18	9	11	9	17	ST-23 complex	1655	4	4	2
52556	BR41492	UK [England]	2015	carrier	Neisseria meningitidis	B	B	B	14	1	57	142	13	6	5	1	151	12	11	14		12059	13	3	1
50281	BR41497	UK [England]	2015	carrier	Neisseria meningitidis	W	W	W	22	1	1	1	4	2	3	4	3	8	4	6	ST-11 complex	11	4	4	2
43646	BR41505	UK [England]	2015	carrier	Neisseria meningitidis	NG	Z	Z	25	1	1	26	4	8	4	6	17	5	18	2	ST-103 complex	103	4	4	2
52557	BR41515	UK [England]	2015	carrier	Neisseria meningitidis	NG	Y	Y	25	1	1	26	4	12	5	18	9	11	9	17	ST-23 complex	1655	4	4	2
43796	BR41516	UK [England]	2015	carrier	Neisseria meningitidis	NG	Y	Y	24	1	1	27	4	2	7	6	17	9	6	8	ST-167 complex	1624	4	4	2
43649	BR41517	UK [England]	2015	carrier	Neisseria meningitidis	NG	B	B	510	28	8	599	30	4	10	5	4	6	3	8	ST-32 complex	32	NA	NA	1
43650	BR41524	UK [England]	2015	carrier	Neisseria meningitidis	NG	cnl	cnl	102	8	1	2	20	16	2	6	25	17	25	22	ST-53 complex	53	20	4	2
43651	BR41525	UK [England]	2015	carrier	Neisseria meningitidis	NG	E	E	12	258	57	266	NA	7	16	55	10	3	56	46	ST-178 complex	178	NA	NA	1
43652	BR41532	UK [England]	2015	carrier	Neisseria meningitidis	NG	E	E	13	3	57	209	3	8	25	7	17	21	26	49	ST-1157 complex	1157	3	3	1
43653	BR41552	UK [England]	2015	carrier	Neisseria meningitidis	NG	cnl	cnl	4	1	1	206	4	5	4	17	15	30	7	12	ST-198 complex	823	4	3	1
43654	BR41564	UK [England]	2015	carrier	Neisseria meningitidis	NG	cnl	cnl	16	1	1	60	4	8	10	5	4	5	3	21	ST-32 complex	12060	4	4	2
43655	BR41586	UK [England]	2015	carrier	Neisseria meningitidis	Y	Y	Y	25	1	1	26	4	12	5	18	9	11	9	17	ST-23 complex	1655	4	4	2
52558	BR41628	UK [England]	2015	carrier	Neisseria meningitidis	Y	Y	Y	25	1	1	26	4	10	5	18	9	11	9	17	ST-23 complex	23	4	4	2
50283	BR41664	UK [England]	2015	carrier	Neisseria meningitidis	NG	B	B	45	8	77	87	20	7	5	1	13	36	53	8	ST-213 complex	4224	20	4	3
52559	BR41667	UK [England]	2015	carrier	Neisseria meningitidis	Y	Y	Y	25	1	1	26	4	12	5	18	9	11	9	17	ST-23 complex	1655	4	4	2
52560	BR41688	UK [England]	2015	carrier	Neisseria meningitidis	W	W/Y	discrepancy	25	1	1	26	4	10	5	18	9	11	9	17	ST-23 complex	23	4	4	2
43663	BR41694	UK [England]	2015	carrier	Neisseria meningitidis	B	B	B	202	1	1	35	4	27	6	9	3	9	6	16	ST-41/44 complex	136	4	4	2
43666	BR41712	UK [England]	2015	carrier	Neisseria meningitidis	W	W	W	651	12	100	125	28	8	5	18	17	11	24	21	ST-22 complex	3651	NA	NA	3
50284	BR41719	UK [England]	2015	carrier	Neisseria meningitidis	W	W	W	16	1	1	60	4	11	5	18	154	11	24	21	ST-22 complex	1281	4	4	2
43668	BR41736	UK [England]	2015	carrier	Neisseria meningitidis	NG	cnl	cnl	4	1	1	206	4	5	4	17	15	30	7	12	ST-198 complex	823	4	3	1
43669	BR41744	UK [England]	2015	carrier	Neisseria meningitidis	NG	cnl	cnl	4	1	1	206	4	10	4	17	15	30	7	12	ST-198 complex	6592	4	3	1
43671	BR41778	UK [England]	2015	carrier	Neisseria meningitidis	NG	E	E	13	3	57	209	3	8	25	7	17	21	26	49	ST-1157 complex	1157	3	3	1

43672	BR41780	UK [England]	2015	carrier	Neisseria meningitidis	NG	B	B	19	1	1	83	4	4	10	2	3	3	11	9	ST-269 complex	6672	4	4	2
43674	BR41785	UK [England]	2015	carrier	Neisseria meningitidis	NG	cnl	cnl	4	1	1	206	4	5	535	17	15	30	26	12	ST-198 complex	12143	4	3	1
43676	BR41790	UK [England]	2015	carrier	Neisseria meningitidis	NG	cnl	cnl	102	8	10	2	20	16	2	6	25	17	25	22	ST-53 complex	53	20	4	2
43677	BR41793	UK [England]	2015	carrier	Neisseria meningitidis	NG	E	E	13	3	57	209	3	8	25	7	17	21	26	49	ST-1157 complex	1157	3	3	1
43797	BR41797	UK [England]	2015	carrier	Neisseria meningitidis	NG	cnl	cnl	4	1	1	206	4	5	4	17	15	30	7	12	ST-198 complex	823	4	3	1
50285	BR41810	UK [England]	2015	carrier	Neisseria meningitidis	NG	Y	Y	25	1	1	26	4	10	5	18	9	11	9	17	ST-23 complex	23	4	4	2
43680	BR41811	UK [England]	2015	carrier	Neisseria meningitidis	NG	cnl	cnl	94	NA		56	69	NA	5	4	17	15	14	7	12	ST-198 complex	198	NA	NA	3
61948	Bris001	UK	2014	carrier	Neisseria meningitidis		E	E	13	3	1	209	3	8	25	7	17	21	26	49	ST-1157 complex	1157	3	3	1
61938	Bris002	UK	2014	carrier	Neisseria meningitidis			NA	25	1	1	26	4	12	5	18	9	11	9	17	ST-23 complex	1655	4	4	2
61959	Bris003	UK	2014	carrier	Neisseria meningitidis		Y	Y	25	1	1	26	4	12	5	18	9	11	9	17	ST-23 complex	1655	4	4	2
61945	Bris004	UK	2014	carrier	Neisseria meningitidis		cnl	cnl	102	8	10	2	20	16	2	6	25	17	25	22	ST-53 complex	53	20	4	2
61935	Bris005	UK	2014	carrier	Neisseria meningitidis		B	B	19	1	4	83	4	4	10	161	9	6	21	3		1167	4	4	2
61951	Bris006	UK	2014	carrier	Neisseria meningitidis		B	B	16	1	4	59	4	4	10	11	18	6	10	12	ST-35 complex	35	4	4	2
61962	Bris007	UK	2014	carrier	Neisseria meningitidis		Y	Y	23	1	1	4	4	2	7	159	92	93	6	8		6464	4	4	2
61970	Bris008	UK	2014	carrier	Neisseria meningitidis		B	B	16	1	4	59	4	4	10	11	18	6	10	12	ST-35 complex	35	4	4	2
61974	Bris009	UK	2014	carrier	Neisseria meningitidis		W	W	16	1	1	60	4	11	5	18	154	11	24	21	ST-22 complex	1281	4	4	2
61958	Bris010	UK	2014	carrier	Neisseria meningitidis		Y	Y	25	1	1	26	4	10	5	18	9	11	9	17	ST-23 complex	23	4	4	2
61980	Bris011	UK	2014	carrier	Neisseria meningitidis			NA	25	1	1	26	4	12	5	18	9	11	9	17	ST-23 complex	1655	4	4	2
61971	Bris012	UK	2014	carrier	Neisseria meningitidis		cnl	cnl	94	NA		56	69	NA	5	4	17	15	14	7	12	ST-198 complex	198	NA	NA	3
61937	Bris013	UK	2014	carrier	Neisseria meningitidis		B	B	13	3	57	209	3	8	25	7	17	21	26	3	ST-1157 complex	12055	3	3	1
61942	Bris014	UK	2014	carrier	Neisseria meningitidis		cnl	cnl	4	1	1	206	4	5	4	17	15	30	7	12	ST-198 complex	823	4	3	1
61976	Bris015	UK	2014	carrier	Neisseria meningitidis		B	B	525	98	1	457	122	9	6	9	1	8	6	6	ST-41/44 complex	7516	NA	NA	1
61961	Bris016	UK	2014	carrier	Neisseria meningitidis		B	B	1	6	3	100	7	8	10	5	4	6	3	8	ST-32 complex	33	7	1	1
61952	Bris017	UK	2014	carrier	Neisseria meningitidis		B	B	45	8	77	87	20	7	5	1	13	36	53	15	ST-213 complex	213	20	4	3
61981	Bris018	UK	2014	carrier	Neisseria meningitidis		E	E	13	3	57	209	3	8	25	7	17	21	26	49	ST-1157 complex	1157	3	3	1
61940	Bris019	UK	2014	carrier	Neisseria meningitidis		Y	Y	25	1	1	26	4	12	5	18	9	11	9	17	ST-23 complex	1655	4	4	2
61964	Bris020	UK	2014	carrier	Neisseria meningitidis		B	B	47	1	57	90	15	12	5	12	35	60	22	17	ST-461 complex	461	15	4	3
61946	Bris021	UK	2014	carrier	Neisseria meningitidis		W	W	22	1	1	1	4	2	3	4	3	8	4	6	ST-11 complex	11	4	4	2
61944	Bris022	UK	2014	carrier	Neisseria meningitidis		NG	NG	13	3	57	209	3	8	25	7	17	21	26	49	ST-1157 complex	1157	3	3	1
61982	Bris023A	UK	2014	carrier	Neisseria meningitidis		NG	NG	13	3	57	209	3	8	25	7	17	21	26	49	ST-1157 complex	1157	3	3	1
61966	Bris023B	UK	2014	carrier	Neisseria meningitidis		NG	NG	13	3	57	209	3	8	25	7	17	21	26	49	ST-1157 complex	1157	3	3	1
61954	Bris024	UK	2014	carrier	Neisseria meningitidis		cnl	cnl	102	8	10	2	20	16	2	6	25	17	25	22	ST-53 complex	53	20	4	2
61957	Bris025	UK	2014	carrier	Neisseria meningitidis		B	B	16	1	4	59	4	4	10	11	18	6	10	12	ST-35 complex	35	4	4	2
61972	Bris026	UK	2014	carrier	Neisseria meningitidis		cnl	cnl	4	1	1	206	4	5	4	17	15	30	7	12	ST-198 complex	823	4	3	1
61963	Bris027	UK	2014	carrier	Neisseria meningitidis		cnl	cnl	4	1	1	206	4	5	4	17	15	30	7	12	ST-198 complex	823	4	3	1
61950	Bris028	UK	2014	carrier	Neisseria meningitidis		B	B	45	8	77	87	20	7	5	1	13	36	53	15	ST-213 complex	213	20	4	3
61965	Bris029	UK	2014	carrier	Neisseria meningitidis		NG	NG	13	3	57	209	3	8	25	7	17	21	26	49	ST-1157 complex	1157	3	3	1
61979	Bris030	UK	2014	carrier	Neisseria meningitidis		cnl	cnl	102	8	10	2	20	16	2	6	25	17	25	22	ST-53 complex	53	20	4	2
61953	Bris031	UK	2014	carrier	Neisseria meningitidis		NG	NG	683	NA		2	176	NA	4	10	15	9	8	11	9	ST-269 complex	269	NA	NA	2
61943	Bris032	UK	2014	carrier	Neisseria meningitidis		cnl	cnl	4	1	1	206	4	5	4	17	15	30	7	12	ST-198 complex	823	4	3	1
61969	Bris033	UK	2014	carrier	Neisseria meningitidis		W	W	651	12	100	125	28	8	5	18	17	11	24	21	ST-22 complex	3651	NA	NA	3
61973	Bris034	UK	2014	carrier	Neisseria meningitidis		cnl	cnl	102	8	10	2	20	16	2	6	25	17	25	22	ST-53 complex	53	20	4	2
61939	Bris035	UK	2014	carrier	Neisseria meningitidis		cnl	cnl	4	1	1	206	4	5	4	17	15	30	7	12	ST-198 complex	823	4	3	1
61960	Bris036	UK	2014	carrier	Neisseria meningitidis		cnl	cnl	4	1	1	206	4	5	4	17	15	30	7	12	ST-198 complex	823	4	3	1
61968	Bris037	UK	2014	carrier	Neisseria meningitidis		B	B	21	1	1	7	4	1	5	13	53	26	41	3	ST-162 complex	162	4	4	2
61936	Bris038	UK	2014	carrier	Neisseria meningitidis		cnl	cnl	102	8	10	2	20	16	2	6	25	17	25	22	ST-53 complex	53	20	4	2
61947	Bris039	UK	2014	carrier	Neisseria meningitidis		cnl	cnl	102	8	10	2	20	16	2	6	25	17	25	22	ST-53 complex	53	20	4	2
61977	Bris040	UK	2014	carrier	Neisseria meningitidis		NG	NG	13	3	57	209	3	8	25	7	17	21	26	49	ST-1157 complex	1157	3	3	1
61955	Bris041	UK	2014	carrier	Neisseria meningitidis		cnl	cnl	16	1	1	60	4	8	10	5	4	5	3	21	ST-32 complex	12060	4	4	2
61975	Bris042	UK	2014	carrier	Neisseria meningitidis		B	B	16	1	4	681	4	4	10	11	17	6	10	12	ST-35 complex	457	4	4	2
61941	Bris043	UK	2014	carrier	Neisseria meningitidis		W	W	22	1	1	1	4	2	3	4	3	8	4	6	ST-11 complex	11	4	4	2
61949	Bris044	UK	2014	carrier	Neisseria meningitidis		B	B	19	1	4	83	4	4	10	161	9	6	21	3		1167	4	4	2
61967	Bris045	UK	2014	carrier	Neisseria meningitidis		E	E	13	3	57	209	3	8	25	7	17	21	26	49	ST-1157 complex	1157	3	3	1
61978	Bris046	UK	2014	carrier	Neisseria meningitidis		W	W	651	12	100	125	28	8	5	18	17	11	24	21	ST-22 complex	3651	NA	NA	3
61956	Bris047	UK	2014	carrier	Neisseria meningitidis			NA	13	3	1	145	3	175	5	484	17	3	4	124		6798	3	3	1
49273	CA40003	UK [Wales]	2015	carrier	Neisseria meningitidis	Y	Y	Y	25	1	1	26	4	12	5	18	9	11	9	17	ST-23 complex	1655	4	4	2
49376	CA40020	UK [Wales]	2015	carrier	Neisseria meningitidis	B	B	B	45	8	77	87	20	7	5	1	13	36	53	15	ST-213 complex	213	20	4	3
49377	CA40030	UK [Wales]	2015	carrier	Neisseria meningitidis	Y	Y	Y	25	1	1	26	4	12	5	18	9	11	9	17	ST-23 complex	1655	4	4	2
49378	CA40036	UK [Wales]	2015	carrier	Neisseria meningitidis	Y	Y	Y	25	1	1	26	4	12	5	18	9	11	9	17	ST-23 complex	1655	4	4	2
49379	CA40041	UK [Wales]	2015	carrier	Neisseria meningitidis	NG	cnl	cnl	14	7	1	142	6	3	6	9	5	9	6	9	ST-41/44 complex	41	6	2	1
49380	CA40046	UK [Wales]	2015	carrier	Neisseria meningitidis	NG	B	B	22	1	77	1	4	7	5	1	13	36	53	15	ST-213 complex	213	4	4	2
49381	CA40049	UK [Wales]	2015	carrier	Neisseria meningitidis	NG	cnl	cnl	4	1	1	206	4	5	4	17	15	30	7	12	ST-198 complex	823	4	3	1
49382	CA40055	UK [Wales]	2015	carrier	Neisseria meningitidis	B	B	B	21	1	1	7	4	1	5	13	53	26	41	3	ST-162 complex	162	4	4	2
49383	CA40059	UK [Wales]	2015	carrier	Neisseria meningitidis	B	B	B	24	1	63	25	4	27	6	9	17	9	6	16	ST-41/44 complex	1770	4	4	2
49384	CA40063	UK [Wales]	2015	carrier	Neisseria meningitidis	NG	E	E	13	3	57	209	3	8	25	7	17	21	26	49	ST-1157 complex	1157	3	3	1
49385	CA40067	UK [Wales]	2015	carrier	Neisseria meningitidis	NG	cnl	cnl	94	58	56	89	NA	5	4	38	15	22	40	13	ST-1136 complex	1136	NA	NA	3
49386	CA40069	UK [Wales]	2015	carrier	Neisseria meningitidis	NG	cnl	cnl	499	15	5	134	58	5	4	17	15	30	7	12	ST-198 complex	823	NA	NA	3
49387	CA40074	UK [Wales]	2015	carrier	Neisseria meningitidis	NG	B	B	187	3	57	136	3	7	5	1	13	36	53	15	ST-213 complex	213	3	4	3
49388	CA40075	UK [Wales]	2015	carrier	Neisseria meningitidis	Y	Y	Y	25	1	1	26	4	12	5	18	9	11	9	17	ST-23 complex	1655	4	4	2
52561	CA40083	UK [Wales]	2015	carrier	Neisseria meningitidis	NG	Y	Y	104	1	57	10	4	10	5	18	59	11	9	17	ST-23 complex	183	4	4	2
49390	CA40097	UK [Wales]	2015	carrier	Neisseria meningitidis	B	B	B	24	1	63	25	4	27	6	9	3	9	6	16	ST-41/44 complex	136	4	4	2
49391	CA40098	UK [Wales]	2015	carrier	Neisseria meningitidis	NG	W	W	22	1	1	1	4	2	3	4	3	8	4	6	ST-11 complex	11	4	4	2
49392	CA40111	UK [Wales]	2015	carrier	Neisseria meningitidis	NG	B	B	19	1	77	65	4	7	5	1	13	36	53	15	ST-213 complex	213	4	4	2
49393	CA40114	UK [Wales]	2015	carrier	Neisseria meningitidis	B	B	B	45	8	1	87	20	7	395	1	25	36	53	15	ST-213 complex	12570	20	4	3
49394	CA40117	UK [Wales]	2015	carrier	Neisseria meningitidis	B	B	B	19	1	1	65	4	9	6	9	9	9	6	9	ST-41/44 complex	44	4	4	2
49395	CA40118	UK [Wales]	2015	carrier	Neisseria meningitidis	B	B	B	14	1	1	166	4	3	6	9	5	9	6	74	ST-41/44 complex	7088	4	3	1
50287	CA40145	UK [Wales]	2015	carrier	Neisseria meningitidis	NG	cnl	cnl	14	7	1	142	6	3	6	9	5	9	6	9	ST-41/44 complex	41	6	2	1
49398	CA40155	UK [Wales]	2015	carrier	Neisseria meningitidis	NG	cnl	cnl	499	15	5	134	58	5	4	17	15	30	7	12	ST-198 complex	823	NA	NA	3
52562	CA40160	UK [Wales]	2015	carrier	Neisseria meningitidis	NG	cnl	cnl	94	157	56	69	NA	5	4	17	15	14	7	12	ST-198 complex	198	NA	NA	3
49400	CA40172	UK [Wales]	2015	carrier	Neisseria meningitidis	NG	E	E	13	3	1	145	3	17	5	19	17	3	26	2	ST-60 complex	60	3	3	1
52563	CA40177	UK [Wales]	2015	carrier	Neisseria meningitidis	B	B	B	19	1	2	65	4	4	10	15	9	8	11	9	ST-269 complex	269	4	4	2
52564	CA40191	UK [Wales]	2015	carrier	Neisseria meningitidis	NG	B	B	268	3	57	528	3	8	25	7	17	21	26	49	ST-1157 complex	1157	3	3	1
49404	CA40197	UK [Wales]	2015	carrier	Neisseria meningitidis	NG	B	B	19	1	1	65	4	9	6	9	9	9	64	9	ST-41/44 complex	409	4	4	2
49405	CA40198	UK [Wales]	2015	carrier	Neisseria meningitidis	NG	cnl	cnl	4	1	1	206	4	5	4	17	15	30	7	12	ST-198 complex	823	4	3	1
49406	CA40206	UK [Wales]	2015	carrier	Neisseria meningitidis	NG	cnl	cnl	94	58	56	89	NA	5	4	38	15	22	40	13	ST-1136 complex	1136	NA	NA	3
49407	CA40208	UK [Wales]	2015	carrier	Neisseria meningitidis	Y	Y	Y	25	1	1	26	4	10	5	314	9	11	9	17	ST-23 complex	3582	4	4	2
49409	CA40221	UK [Wales]	2015	carrier	Neisseria meningitidis	NG	cnl	cnl	14	7	1	142	6	3	6	9	5	9	6	9	ST-41/44 complex	41	6	2	1
46674	CA40230	UK [Wales]	2015	carrier	Neisseria meningitidis	NG	cnl	cnl	21	1	1	7	4	16	2	159	92	77	25	112	ST-1117 complex	1117	4	4	2
49411	CA40259	UK [Wales]	2015	carrier	Neisseria meningitidis	NG	E	E	13	3	1	145	3	17	4	19	17	3	26	2	ST-60 complex	4146	3	3	1
49412	CA40303	UK [Wales]	2015	carrier	Neisseria meningitidis	NG	cnl	cnl	21	1	1	7	4	16	2	159	92	77	25	112	ST-1117 complex	1117	4	4	2
49413	CA40316	UK [Wales]	2015	carrier	Neisseria meningitidis	NG	E	E	13	3	57	209	3	8	25	7	17	21	26	49	ST-1157 complex	1157	3	3	1
46590	CA40338	UK [Wales]	2015	carrier	Neisseria meningitidis	NG	cnl	cnl	19	1	1	65	4	3	6	9	5	9	24	9	ST-41/44 complex	2578	4	4	2
49415	CA40352	UK [Wales]	2015	carrier	Neisseria meningitidis	NG	cnl	cnl	102	8	10	2	20	16	2	18	25	17	25	22	ST-53 complex	2441	20	4	2
49416	CA40356	UK [Wales]	2015	carrier	Neisseria meningitidis	B	B	B	1022	1	1	789	4	4	10	34	5	3	11	9	ST-269 complex	7835	4	4	2
49417	CA40360	UK [Wales]	2015	carrier	Neisseria meningitidis	NG	B	B	19	1	1	65	4	3	6	9	5	9	6	70	ST-41/44 complex	414	4	4	2
46584	CA40402	UK [Wales]	2015	carrier	Neisseria meningitidis	NG	cnl	cnl	102	8	10	2	20	16	2	6	25	17	25	22	ST-53 complex	53	20	4	2
49419	CA40406	UK [Wales]	2015	carrier	Neisseria meningitidis	NG	cnl	cnl	102	8	10	2	20	16	2	6	25	17	25	22	ST-53 complex	53	20	4	2
49420	CA40412	UK [Wales]	2015	carrier	Neisseria meningitidis	Y	Y	Y	25	1	1	26	4	12	5	18	9	11	9	17	ST-23 complex	1655	4	4	2
49421	CA40432	UK [Wales]	2015	carrier	Neisseria meningitidis	NG	H	H	14	1	1	142	13	8	5	4	17	8	21	22	ST-865 complex	3134	13	3	1
49422	CA40476	UK [Wales]	2015	carrier	Neisseria meningitidis	B	B	B	13	3	57	145	3	4	10	34	5	38	11	9	ST-269 complex	1161	3	3	1
46598	CA40483	UK [Wales]	2015	carrier	Neisseria meningitidis	NG	E	E	13	3	57	209	3	8	25	7	17	21	26	49	ST-1157 complex	1157	3	3	1
49423	CA40489	UK [Wales]	2015	carrier	Neisseria meningitidis	Y	Y	Y	25	1	1	26	4	12	5	18	9	11	9	17	ST-23 complex	1655	4	4	2
50288	CA40498	UK [Wales]	2015	carrier	Neisseria meningitidis	W	W	W	22	1	1	1	4	2	3	4	3	8	4	6	ST-11 complex	11	4	4	2
49424	CA40507	UK [Wales]	2015	carrier	Neisseria meningitidis	NG	Z	Z	25	1	1	26	4	8	4	6	17	5	18	2	ST-103 complex	103	4	4	2
46586	CA40517	UK [Wales]	2015	carrier	Neisseria meningitidis	Y	Y	Y	25	1	1	26	4	12	5	18	9	11	9	17	ST-23 complex	1655	4	4	2
49425	CA40524	UK [Wales]	2015	carrier	Neisseria meningitidis	Y	Y	Y	25	1	1	26	4	12	5	18	9	11	9	17	ST-23 complex	1655	4	4	2
49426	CA40526	UK [Wales]	2015	carrier	Neisseria meningitidis	B	B	B	13	3	57	145	3	4	10	34	5	38	11	9	ST-269 complex	1161	3	3	1
49427	CA40533	UK [Wales]	2015	carrier	Neisseria meningitidis	B	B	B	45	8	77	87	20	7	5	1	13	36	53	15	ST-213 complex	213	20	4	3
49428	CA40541	UK [Wales]	2015	carrier	Neisseria meningitidis	Y	Y	Y	25	1	1	26	4	10	5	18	9	11	9	17	ST-23 complex	23	4	4	2
49429	CA40552	UK [Wales]	2015	carrier	Neisseria meningitidis	B	B	B	45	8	77	87	20	7	5	1	13	36	53	15	ST-213 complex	213	20	4	3
49430	CA40553	UK [Wales]	2015	carrier	Neisseria meningitidis	NG	B	B	45	8	77	87	20	7	5	1	13	36	53	15	ST-213 complex	213	20	4	3
49432	CA40583	UK [Wales]	2015	carrier	Neisseria meningitidis	Y	Y	Y	24	1	1	27	4	2	7	6	17	9	6	8	ST-167 complex	1624	4	4	2
49433	CA40584	UK [Wales]	2015	carrier	Neisseria meningitidis	Y	Y	Y	25	1	1	26	4	12	5	18	9	11	9	17	ST-23 complex	1655	4	4	2
49435	CA40596	UK [Wales]	2015	carrier	Neisseria meningitidis	NG	cnl	cnl	102	8	10	2	20	16	2	6	25	17	25	22	ST-53 complex	53	20	4	2
49436	CA40605	UK [Wales]	2015	carrier	Neisseria meningitidis	NG	cnl	cnl	4	1	1	206	4	5	4	17	15	30	7	12	ST-198 complex	823	4	3	1
49438	CA40656	UK [Wales]	2015	carrier	Neisseria meningitidis	B	B	B	14	1	1	142	13	3	6	108	9	9	6	9	ST-41/44 complex	2364	13	3	1
46722	CA40690	UK [Wales]	2015	carrier	Neisseria meningitidis	Y	Y	Y	25	1	1	26	4	12	5	18	9	11	9	17	ST-23 complex	1655	4	4	2
50289	CA40699	UK [Wales]	2015	carrier	Neisseria meningitidis	B	B	B	45	8	77	87	20	7	5	1	13	36	53	15	ST-213 complex	213	20	4	3
49440	CA40706	UK [Wales]	2015	carrier	Neisseria meningitidis	NG	cnl	cnl	102	8	10	2	20	16	2	6	25	17	25	22	ST-53 complex	53	20	4	2
46583	CA40738	UK [Wales]	2015	carrier	Neisseria meningitidis	NG	E	E	13	3	1	145	3	12	5	9	17	3	172	124		3207	3	3	1
49441	CA40742	UK [Wales]	2015	carrier	Neisseria meningitidis	NG	cnl	cnl	21	1	1	7	4	16	2	159	92	77	25	112	ST-1117 complex	1117	4	4	2
49442	CA40746	UK [Wales]	2015	carrier	Neisseria meningitidis	NG	cnl	cnl	4	1	1	206	4	5	4	17	15	30	7	12	ST-198 complex	823	4	3	1
49443	CA40748	UK [Wales]	2015	carrier	Neisseria meningitidis	Y	Y	Y	25	1	1	26	4	12	5	18	9	11	9	17	ST-23 complex	1655	4	4	2
49444	CA40755	UK [Wales]	2015	carrier	Neisseria meningitidis	NG	cnl	cnl	102	79	10	2	72	16	2	6	25	17	25	22	ST-53 complex	53	NA	NA	2
49445	CA40757	UK [Wales]	2015	carrier	Neisseria meningitidis	NG	B	B	106	1	2	146	4	4	10	2	161	8	11	20	ST-269 complex	2369	4	4	2
49446	CA40759	UK [Wales]	2015	carrier	Neisseria meningitidis	NG	cnl	cnl	4	1	1	206	4	453	4	17	15	30	818	12	ST-198 complex	12624	4	3	1
49447	CA40769	UK [Wales]	2015	carrier	Neisseria meningitidis	Y	Y	Y	25	1	1	26	4	10	5	18	9	11	9	17	ST-23 complex	23	4	4	2
49448	CA40795	UK [Wales]	2015	carrier	Neisseria meningitidis	Y	Y	Y	25	1	1	26	4	10	5	18	9	11	9	17	ST-23 complex	23	4	4	2
49449	CA40796	UK [Wales]	2015	carrier	Neisseria meningitidis	NG	E	E	13	3	57	209	3	8	25	7	17	21	26	49	ST-1157 complex	1157	3	3	1
49450	CA40800	UK [Wales]	2015	carrier	Neisseria meningitidis	B	B	B	45	8	77	87	20	7	5	1	13	36	53	15	ST-213 complex	213	20	4	3
49451	CA40808	UK [Wales]	2015	carrier	Neisseria meningitidis	NG	E	E	13	3	57	209	3	8	25	7	17	21	26	49	ST-1157 complex	1157	3	3	1
46587	CA40819	UK [Wales]	2015	carrier	Neisseria meningitidis	Y	Y	Y	25	1	1	26	4	12	5	18	9	11	9	17	ST-23 complex	1655	4	4	2
49452	CA40820	UK [Wales]	2015	carrier	Neisseria meningitidis	NG	cnl	cnl	102	8	1	2	20	16	2	6	25	17	25	22	ST-53 complex	53	20	4	2
49453	CA40833	UK [Wales]	2015	carrier	Neisseria meningitidis	Y	Y	Y	24	1	1	27	4	2	7	6	17	9	6	8	ST-167 complex	1624	4	4	2
49456	CA40848	UK [Wales]	2015	carrier	Neisseria meningitidis	B	B	B	4	2	1	84	2	3	6	9	5	9	6	9	ST-41/44 complex	41	2	3	1
49457	CA40849	UK [Wales]	2015	carrier	Neisseria meningitidis	NG	E	E	13	3	57	209	3	8	25	7	17	21	26	49	ST-1157 complex	1157	3	3	1
52566	CA40856	UK [Wales]	2015	carrier	Neisseria meningitidis	Y	Y	Y	25	1	1	26	4	12	5	18	9	11	9	17	ST-23 complex	1655	4	4	2
49460	CA40862	UK [Wales]	2015	carrier	Neisseria meningitidis	NG	Y	Y	119	1	1	98	4	6	3	4	9	8	13	169		1768	4	4	2

49461	CA40868	UK [Wales]	2015	carrier	Neisseria meningitidis	NG	cnl	cnl	4	1	1	206	4	5	4	17	17	30	7	12	ST-198 complex	2384	4	3	1
49462	CA40875	UK [Wales]	2015	carrier	Neisseria meningitidis	Y	Y	Y	104	1	57	10	4	10	5	18	59	11	9	17	ST-23 complex	183	4	4	2
49463	CA40881	UK [Wales]	2015	carrier	Neisseria meningitidis	NG	E	E	13	3	57	209	3	8	25	7	17	21	26	49	ST-1157 complex	1157	3	3	1
49464	CA40883	UK [Wales]	2015	carrier	Neisseria meningitidis	NG	E	E	13	3	57	209	3	8	25	7	17	21	26	49	ST-1157 complex	1157	3	3	1
49466	CA40895	UK [Wales]	2015	carrier	Neisseria meningitidis	B	B	B	21	1	1	7	4	4	10	5	26	6	3	8	ST-32 complex	1096	4	4	2
46605	CA40943	UK [Wales]	2015	carrier	Neisseria meningitidis	NG	Z	Z	25	1	1	26	4	8	4	6	17	5	18	2	ST-103 complex	103	4	4	2
49467	CA40946	UK [Wales]	2015	carrier	Neisseria meningitidis	Y	Y	Y	25	1	1	26	4	12	5	18	9	11	9	17	ST-23 complex	1655	4	4	2
49468	CA40947	UK [Wales]	2015	carrier	Neisseria meningitidis	Y	Y	Y	25	1	1	26	4	12	5	18	9	11	9	17	ST-23 complex	1655	4	4	2
52568	CA40952	UK [Wales]	2015	carrier	Neisseria meningitidis	Y	Y	Y	119	1	1	98	4	6	3	4	9	8	13	169		1768	4	4	2
52569	CA40954	UK [Wales]	2015	carrier	Neisseria meningitidis	NG	NG	NG	21	1	1	7	4	4	10	15	9	8	11	13	ST-269 complex	467	4	4	2
49471	CA40956	UK [Wales]	2015	carrier	Neisseria meningitidis	NG	cnl	cnl	985	8	10	787	20	16	2	6	25	17	25	22	ST-53 complex	53	20	4	2
52570	CA40963	UK [Wales]	2015	carrier	Neisseria meningitidis	NG	B	B	102	199	10	2	NA	7	5	1	13	36	53	15	ST-213 complex	213	NA	NA	2
49472	CA40974	UK [Wales]	2015	carrier	Neisseria meningitidis	Y	Y	Y	25	1	1	26	4	12	5	18	9	11	9	17	ST-23 complex	1655	4	4	2
49473	CA40979	UK [Wales]	2015	carrier	Neisseria meningitidis	Y	Y	Y	25	1	1	26	4	12	5	18	9	11	9	17	ST-23 complex	1655	4	4	2
49474	CA41013	UK [Wales]	2015	carrier	Neisseria meningitidis	NG	cnl	cnl	4	1	1	206	4	5	4	17	15	30	7	12	ST-198 complex	823	4	3	1
49476	CA41016	UK [Wales]	2015	carrier	Neisseria meningitidis	NG	cnl	cnl	21	1	1	7	4	16	2	159	92	77	25	112	ST-1117 complex	1117	4	4	2
49477	CA41029	UK [Wales]	2015	carrier	Neisseria meningitidis	NG	cnl	cnl	4	1	1	206	4	5	4	17	15	30	7	12	ST-198 complex	823	4	3	1
49479	CA41041	UK [Wales]	2015	carrier	Neisseria meningitidis	B	B	B	713	1	57	344	13	12	10	10	72	8	21	139		1111	13	3	1
49480	CA41127	UK [Wales]	2015	carrier	Neisseria meningitidis	NG	B	B	19	112	1	65	106	4	10	15	7	8	11	1	ST-269 complex	13	NA	NA	2
49481	CA41144	UK [Wales]	2015	carrier	Neisseria meningitidis	Y	Y	Y	24	1	1	27	4	2	7	6	17	9	6	8	ST-167 complex	1624	4	4	2
49482	CA41147	UK [Wales]	2015	carrier	Neisseria meningitidis	NG	cnl	cnl	4	1	1	206	4	5	4	17	15	30	7	12	ST-198 complex	823	4	3	1
49483	CA41154	UK [Wales]	2015	carrier	Neisseria meningitidis	NG	cnl	cnl	21	1	1	7	4	16	2	159	92	77	25	112	ST-1117 complex	1117	4	4	2
49484	CA41161	UK [Wales]	2015	carrier	Neisseria meningitidis	W	W	W	22	1	1	1	4	2	3	4	3	8	4	6	ST-11 complex	11	4	4	2
49485	CA41166	UK [Wales]	2015	carrier	Neisseria meningitidis	NG	B	B	16	1	2	60	4	222	3	58	261	263	5	255	ST-4821 complex	3200	4	4	2
49486	CA41174	UK [Wales]	2015	carrier	Neisseria meningitidis	NG	cnl	cnl	102	8	10	2	20	16	2	6	25	17	25	22	ST-53 complex	53	20	4	2
49487	CA41187	UK [Wales]	2015	carrier	Neisseria meningitidis	NG	cnl	cnl	4	1	1	206	4	5	4	17	15	30	7	12	ST-198 complex	823	4	3	1
49488	CA41188	UK [Wales]	2015	carrier	Neisseria meningitidis	NG	B	B	24	1	1	25	4	6	6	9	9	9	6	17	ST-41/44 complex	833	4	4	2
49489	CA41220	UK [Wales]	2015	carrier	Neisseria meningitidis	NG	E	E	13	3	57	209	3	8	25	7	17	21	26	49	ST-1157 complex	1157	3	3	1
49490	CA41229	UK [Wales]	2015	carrier	Neisseria meningitidis	NG	Z	Z	25	1	1	26	4	8	4	6	17	5	18	2	ST-103 complex	103	4	4	2
49491	CA41259	UK [Wales]	2015	carrier	Neisseria meningitidis	NG	cnl	cnl	102	8	10	2	20	16	2	6	25	17	25	22	ST-53 complex	53	20	4	2
49492	CA41277	UK [Wales]	2015	carrier	Neisseria meningitidis	W	W	W	22	1	1	1	4	2	3	4	3	8	4	6	ST-11 complex	11	4	4	2
49493	CA41280	UK [Wales]	2015	carrier	Neisseria meningitidis	NG	B	B	19	1	1	65	4	9	6	9	9	9	64	9	ST-41/44 complex	409	4	4	2
52571	CA41303	UK [Wales]	2015	carrier	Neisseria meningitidis	NG	Y	Y	4	2	57	84	2	10	5	18	59	11	41	17	ST-23 complex	2902	2	3	1
49495	CA41316	UK [Wales]	2015	carrier	Neisseria meningitidis	NG	cnl	cnl	22	1	1	1	4	16	2	6	25	17	25	22	ST-53 complex	53	4	4	2
49496	CA41329	UK [Wales]	2015	carrier	Neisseria meningitidis	Y	Y	Y	25	1	1	26	4	12	5	18	9	11	9	17	ST-23 complex	1655	4	4	2
49497	CA41346	UK [Wales]	2015	carrier	Neisseria meningitidis	B	B	B	13	3	1	145	3	7	5	1	13	82	53	15	ST-213 complex	575	3	3	1
49498	CA41347	UK [Wales]	2015	carrier	Neisseria meningitidis	NG	E	E	13	3	57	209	3	8	25	7	17	21	25	49	ST-1157 complex	1669	3	3	1
49499	CA41369	UK [Wales]	2015	carrier	Neisseria meningitidis	NG	Z	Z	16	56	79	60	NA	6	29	2	17	26	65	53		12571	NA	NA	2
49500	CA41382	UK [Wales]	2015	carrier	Neisseria meningitidis	NG	cnl	cnl	499	15	5	134	58	5	4	17	15	30	7	12	ST-198 complex	823	NA	NA	3
49501	CA41412	UK [Wales]	2015	carrier	Neisseria meningitidis	NG	E	E	13	3	1	145	3	17	5	19	10	3	26	2	ST-60 complex	1383	3	3	1
49502	CA41429	UK [Wales]	2015	carrier	Neisseria meningitidis	NG	cnl	cnl	102	8	1	2	20	16	2	6	25	17	25	22	ST-53 complex	53	20	4	2
49503	CA41451	UK [Wales]	2015	carrier	Neisseria meningitidis	Y	Y	Y	25	1	1	26	4	12	5	18	9	11	9	17	ST-23 complex	1655	4	4	2
49504	CA41453	UK [Wales]	2015	carrier	Neisseria meningitidis	B	B	B	47	1	57	90	15	12	5	12	35	192	22	17	ST-461 complex	1946	15	4	3
49505	CA41456	UK [Wales]	2015	carrier	Neisseria meningitidis	NG	cnl	cnl	499	15	5	134	58	5	4	17	15	30	7	12	ST-198 complex	823	NA	NA	3
49506	CA41472	UK [Wales]	2015	carrier	Neisseria meningitidis	NG	E	E	13	3	57	209	3	8	25	7	17	21	26	49	ST-1157 complex	1157	3	3	1
49507	CA41475	UK [Wales]	2015	carrier	Neisseria meningitidis	NG	E	E	13	3	57	209	3	8	25	7	17	21	26	49	ST-1157 complex	1157	3	3	1
49508	CA41506	UK [Wales]	2015	carrier	Neisseria meningitidis	Y	Y	Y	25	1	1	26	4	12	5	18	9	11	9	17	ST-23 complex	1655	4	4	2
49509	CA41510	UK [Wales]	2015	carrier	Neisseria meningitidis	Y	Y	Y	19	1	1	93	13	2	16	6	17	9	18	8	ST-167 complex	168	13	4	2
52572	CA41519	UK [Wales]	2015	carrier	Neisseria meningitidis	NG	NG	NG	321	111	1	598	NA	6	7	4	56	26	18	8	ST-175 complex	175	NA	NA	1
49511	CA41520	UK [Wales]	2015	carrier	Neisseria meningitidis	NG	X	X	13	3	57	209	3	8	5	7	13	21	26	2	ST-1157 complex	12572	3	3	1
49512	CA41573	UK [Wales]	2015	carrier	Neisseria meningitidis	NG	E	E	13	3	57	209	3	8	25	7	17	21	25	49	ST-1157 complex	1669	3	3	1
49513	CA41583	UK [Wales]	2015	carrier	Neisseria meningitidis	NG	Y	Y	25	1	1	26	4	12	5	18	9	11	9	17	ST-23 complex	1655	4	4	2
49514	CA41592	UK [Wales]	2015	carrier	Neisseria meningitidis	B	B	B	592	1	57	338	13	12	582	12	35	192	22	17	ST-461 complex	12625	13	3	1
49515	CA41615	UK [Wales]	2015	carrier	Neisseria meningitidis	Y	Y	Y	25	1	1	26	4	12	5	18	9	11	9	17	ST-23 complex	1655	4	4	2
49516	CA41620	UK [Wales]	2015	carrier	Neisseria meningitidis	NG	cnl	cnl	102	8	10	2	20	16	2	6	25	17	25	22	ST-53 complex	53	20	4	2
49517	CA41628	UK [Wales]	2015	carrier	Neisseria meningitidis	NG	cnl	cnl	102	8	10	2	20	16	2	18	25	17	25	22	ST-53 complex	2441	20	4	2
49518	CA41632	UK [Wales]	2015	carrier	Neisseria meningitidis	NG	NG	NG	525	98	1	457	122	285	6	9	1	9	6	9	ST-41/44 complex	4184	NA	NA	1
49519	CA41636	UK [Wales]	2015	carrier	Neisseria meningitidis	Y	Y	Y	25	1	1	26	4	10	5	18	9	11	9	17	ST-23 complex	23	4	4	2
49520	CA41653	UK [Wales]	2015	carrier	Neisseria meningitidis	NG	Y	Y	25	1	1	26	4	12	5	18	9	11	9	17	ST-23 complex	1655	4	4	2
49521	CA41689	UK [Wales]	2015	carrier	Neisseria meningitidis	B	B	B	13	3	57	145	3	4	10	34	5	38	11	9	ST-269 complex	1161	3	3	1
49523	CA41704	UK [Wales]	2015	carrier	Neisseria meningitidis	NG	E	E	19	1	1	65	4	8	25	7	17	21	26	49	ST-1157 complex	1157	4	4	2
49525	CA41725	UK [Wales]	2015	carrier	Neisseria meningitidis	NG	cnl	cnl	102	8	10	2	20	16	2	6	25	17	25	22	ST-53 complex	53	20	4	2
49526	CA41727	UK [Wales]	2015	carrier	Neisseria meningitidis	NG	E	E	13	3	57	209	3	8	25	7	17	21	26	49	ST-1157 complex	1157	3	3	1
49527	CA41748	UK [Wales]	2015	carrier	Neisseria meningitidis	NG	E	E	13	3	57	209	3	8	25	7	17	21	26	49	ST-1157 complex	1157	3	3	1
52574	CA41762	UK [Wales]	2015	carrier	Neisseria meningitidis	NG	B	B	30	17	11	54	37	12	10	10	822	8	21	139		12573	NA	NA	3
49529	CA41772	UK [Wales]	2015	carrier	Neisseria meningitidis	B	B	B	24	1	63	25	4	27	7	9	3	9	20	16		5364	4	4	2
49530	CA41789	UK [Wales]	2015	carrier	Neisseria meningitidis	Y	Y	Y	25	1	1	26	4	12	5	18	9	11	9	17	ST-23 complex	1655	4	4	2
49531	CA41792	UK [Wales]	2015	carrier	Neisseria meningitidis	NG	cnl	cnl	985	8	10	787	20	16	2	6	25	17	25	22	ST-53 complex	53	20	4	2
52575	CA41819	UK [Wales]	2015	carrier	Neisseria meningitidis	NG	B	B	106	1	1	146	4	4	5	2	9	21	11	20		336	4	4	2
49534	CA41834	UK [Wales]	2015	carrier	Neisseria meningitidis	W	W	W	16	1	1	60	4	11	5	18	8	78	24	21	ST-22 complex	1224	4	4	2
49535	CA41844	UK [Wales]	2015	carrier	Neisseria meningitidis	NG	E	E	13	3	57	209	3	8	25	7	17	21	26	49	ST-1157 complex	1157	3	3	1
49536	CA41850	UK [Wales]	2015	carrier	Neisseria meningitidis	NG	W	W	22	1	1	1	4	2	3	4	3	8	4	6	ST-11 complex	11	4	4	2
49537	CA41858	UK [Wales]	2015	carrier	Neisseria meningitidis	NG	E	E	141	1	4	519	4	12	5	19	17	5	24	54		12574	4	4	3
49539	CA41862	UK [Wales]	2015	carrier	Neisseria meningitidis	NG	cnl	cnl	4	1	1	206	4	5	4	17	15	30	7	12	ST-198 complex	823	4	3	1
49540	CA41865	UK [Wales]	2015	carrier	Neisseria meningitidis	W	W	W	22	1	1	1	4	2	3	4	3	8	4	6	ST-11 complex	11	4	4	2
49542	CA41884	UK [Wales]	2015	carrier	Neisseria meningitidis	Y	Y	Y	25	1	1	26	4	10	5	18	9	11	9	17	ST-23 complex	23	4	4	2
49543	CA41893	UK [Wales]	2015	carrier	Neisseria meningitidis	Y	Y	Y	25	1	1	26	4	12	5	18	9	11	9	17	ST-23 complex	1655	4	4	2
49544	CA41900	UK [Wales]	2015	carrier	Neisseria meningitidis	Y	Y	Y	19	1	1	93	13	2	16	6	17	9	18	2	ST-103 complex	5987	13	4	2
49545	CA41902	UK [Wales]	2015	carrier	Neisseria meningitidis	NG	cnl	cnl	4	1	1	206	4	5	4	17	15	30	7	12	ST-198 complex	823	4	3	1
49546	CA41921	UK [Wales]	2015	carrier	Neisseria meningitidis	NG	B	B	45	8	77	87	20	7	5	1	13	36	53	15	ST-213 complex	213	20	4	3
49547	CA41952	UK [Wales]	2015	carrier	Neisseria meningitidis	NG	cnl	cnl	16	1	4	59	4	4	10	11	17	6	10	12	ST-35 complex	457	4	4	2
49548	CA41956	UK [Wales]	2015	carrier	Neisseria meningitidis	Y	Y	Y	25	1	1	26	4	12	5	18	9	11	9	17	ST-23 complex	1655	4	4	2
49550	CA41960	UK [Wales]	2015	carrier	Neisseria meningitidis	NG	E	E	13	3	57	209	3	8	25	7	17	21	26	49	ST-1157 complex	1157	3	3	1
49551	CA41966	UK [Wales]	2015	carrier	Neisseria meningitidis	NG	cnl	cnl	985	8	10	787	20	16	2	6	25	17	25	22	ST-53 complex	53	20	4	2
49552	CA41967	UK [Wales]	2015	carrier	Neisseria meningitidis	NG	Y	Y	19	1	1	93	13	2	16	6	17	9	18	8	ST-167 complex	168	13	4	2
49553	CA41969	UK [Wales]	2015	carrier	Neisseria meningitidis	Y	Y	Y	25	1	1	26	4	12	5	18	9	11	9	17	ST-23 complex	1655	4	4	2
49554	CA41992	UK [Wales]	2015	carrier	Neisseria meningitidis	NG	B	B	45	8	77	87	20	7	5	1	13	36	53	15	ST-213 complex	213	20	4	3
49555	CA41993	UK [Wales]	2015	carrier	Neisseria meningitidis	B	B	B	16	1	4	59	4	4	10	11	18	6	10	12	ST-35 complex	35	4	4	2
49556	CA41997	UK [Wales]	2015	carrier	Neisseria meningitidis	NG	Z	Z	1023	1	79	791	4	13	29	2	17	5	65	53		12627	4	4	2
49557	CA42058	UK [Wales]	2015	carrier	Neisseria meningitidis	NG	cnl	cnl	4	1	1	206	4	5	4	849	15	30	7	12	ST-198 complex	12628	4	3	1
49558	CA42061	UK [Wales]	2015	carrier	Neisseria meningitidis	W	W	W	22	1	1	1	4	2	3	4	3	8	4	6	ST-11 complex	11	4	4	2
49559	CA42071	UK [Wales]	2015	carrier	Neisseria meningitidis	NG	cnl	cnl	102	8	1	2	20	16	2	6	25	17	25	22	ST-53 complex	53	20	4	2
52576	CA42139	UK [Wales]	2015	carrier	Neisseria meningitidis	C	C	C	19	1	1	65	4	12	5	4	9	5	6	9		10909	4	4	2
49561	CA42151	UK [Wales]	2015	carrier	Neisseria meningitidis	NG	cnl	cnl	4	1	1	785	4	5	4	17	15	30	7	12	ST-198 complex	823	4	3	1
49562	CA42157	UK [Wales]	2015	carrier	Neisseria meningitidis	B	B	B	24	1	63	25	4	27	6	9	3	9	6	16	ST-41/44 complex	136	4	4	2
49563	CA42160	UK [Wales]	2015	carrier	Neisseria meningitidis	NG	cnl	cnl	94	184	56	89	NA	5	4	38	15	22	40	13	ST-1136 complex	1136	NA	NA	3
52577	CA42266	UK [Wales]	2015	carrier	Neisseria meningitidis	Y	Y	Y	25	1	1	26	4	10	5	18	9	11	9	17	ST-23 complex	23	4	4	2
49567	CA42333	UK [Wales]	2015	carrier	Neisseria meningitidis	B	B	B	24	1	63	25	4	27	6	9	3	9	6	16	ST-41/44 complex	136	4	4	2
49568	CA42341	UK [Wales]	2015	carrier	Neisseria meningitidis	NG	cnl	cnl	4	1	1	206	4	5	4	849	15	30	7	12	ST-198 complex	12628	4	3	1
49570	CA42425	UK [Wales]	2015	carrier	Neisseria meningitidis	NG	cnl	cnl	4	1	1	206	4	5	4	849	15	30	7	12	ST-198 complex	12628	4	3	1
49572	CA42447	UK [Wales]	2015	carrier	Neisseria meningitidis	NG	cnl	cnl	4	1	1	206	4	5	4	849	15	30	7	12	ST-198 complex	12628	4	3	1
49573	CA42470	UK [Wales]	2015	carrier	Neisseria meningitidis	NG	cnl	cnl	4	1	1	206	4	5	4	17	15	30	7	12	ST-198 complex	823	4	3	1
49574	CA42491	UK [Wales]	2015	carrier	Neisseria meningitidis	Y	Y	Y	25	1	1	26	4	10	5	18	9	11	9	17	ST-23 complex	23	4	4	2
49575	CA42551	UK [Wales]	2015	carrier	Neisseria meningitidis	NG	B	B	16	1	4	59	4	4	10	161	315	6	21	3		12635	4	4	2
49576	CA42560	UK [Wales]	2015	carrier	Neisseria meningitidis	NG	X	X	12	1	1	266	13	2	5	219	827	3	18	7		12636	13	3	1
49577	CA42640	UK [Wales]	2015	carrier	Neisseria meningitidis	B	B	B	24	1	2	25	4	27	6	9	3	9	6	846	ST-41/44 complex	12637	4	4	2
49578	CA42658	UK [Wales]	2015	carrier	Neisseria meningitidis	Y	Y	Y	25	1	1	26	4	10	5	18	9	11	9	17	ST-23 complex	23	4	4	2
49579	CA42665	UK [Wales]	2015	carrier	Neisseria meningitidis	B	B	B	45	8	77	87	20	7	5	1	13	36	53	15	ST-213 complex	213	20	4	3
49580	CA42669	UK [Wales]	2015	carrier	Neisseria meningitidis	NG	B	B	19	1	77	65	4	7	5	1	13	36	53	15	ST-213 complex	213	4	4	2
49581	CA42671	UK [Wales]	2015	carrier	Neisseria meningitidis	B	B	B	713	1	57	344	13	12	10	10	72	8	21	139		1111	13	3	1
49582	CA42672	UK [Wales]	2015	carrier	Neisseria meningitidis	NG	cnl	cnl	4	1	1	206	4	5	4	17	15	30	7	12	ST-198 complex	823	4	3	1
49583	CA42696	UK [Wales]	2015	carrier	Neisseria meningitidis	NG	cnl	cnl	94	NA	56	89	NA	5	4	712	15	22	40	13	ST-1136 complex	10480	NA	NA	3
49584	CA42707	UK [Wales]	2015	carrier	Neisseria meningitidis	NG	Z	Z	25	1	1	26	4	8	4	6	17	5	18	2	ST-103 complex	103	4	4	2
49585	CA42717	UK [Wales]	2015	carrier	Neisseria meningitidis	B	B	B	13	3	57	145	3	4	10	34	5	38	11	9	ST-269 complex	1161	3	3	1
49586	CA42779	UK [Wales]	2015	carrier	Neisseria meningitidis	NG	cnl	cnl	21	1	1	7	4	16	2	159	92	77	25	112	ST-1117 complex	1117	4	4	2
52578	CA42822	UK [Wales]	2015	carrier	Neisseria meningitidis	NG	NG	NG	25	1	1	26	4	10	5	18	9	11	9	17	ST-23 complex	23	4	4	2
49588	CA42862	UK [Wales]	2015	carrier	Neisseria meningitidis	NG	cnl	cnl	14	8	10	788	20	3	6	9	5	9	6	9	ST-41/44 complex	41	20	3	1
49589	CA42865	UK [Wales]	2015	carrier	Neisseria meningitidis	NG	cnl	cnl	985	8	10	787	20	16	2	6	25	17	25	22	ST-53 complex	53	20	4	2
49590	CA42889	UK [Wales]	2015	carrier	Neisseria meningitidis	NG	B	B	16	1	4	59	4	4	124	18	18	6	10	12	ST-35 complex	3093	4	4	2
52579	CA42906	UK [Wales]	2015	carrier	Neisseria meningitidis	B	B	B	24	1	63	25	4	27	6	9	3	9	6	16	ST-41/44 complex	136	4	4	2
49592	CA42914	UK [Wales]	2015	carrier	Neisseria meningitidis	B	B	B	19	1	1	65	4	801	7	9	3	815	6	9		12575	4	4	2
49593	CA42915	UK [Wales]	2015	carrier	Neisseria meningitidis	NG	cnl	cnl	102	8	1	2	20	16	2	6	25	17	25	22	ST-53 complex	53	20	4	2
49594	CA44002	UK [Wales]	2015	carrier	Neisseria meningitidis	B	B	B	13	3	1	209	3	8	25	7	17	21	26	49	ST-1157 complex	1157	3	3	1
49595	CA44003	UK [Wales]	2015	carrier	Neisseria meningitidis	NG	B	B	19	1	1	83	4	4	10	2	5	38	11	9	ST-269 complex	275	4	4	2
49596	CA44009	UK [Wales]	2015	carrier	Neisseria meningitidis	NG	E	E	13	3	57	209	3	8	25	7	17	21	26	49	ST-1157 complex	1157	3	3	1
49599	CA44039	UK [Wales]	2015	carrier	Neisseria meningitidis	Y	Y	Y	25	1	1	26	4	10	5	18	9	11	9	17	ST-23 complex	23	4	4	2
52581	CA44041	UK [Wales]	2015	carrier	Neisseria meningitidis	W	W	W	22	1	1	1	4	2	3	4	3	8	4	6	ST-11 complex	11	4	4	2
49601	CA44068	UK [Wales]	2015	carrier	Neisseria meningitidis	Y	Y	Y	25	1	1	26	4	10	5	18	9	11	9	17	ST-23 complex	23	4	4	2
49602	CA44075	UK [Wales]	2015	carrier	Neisseria meningitidis	NG	E	E	13	3	57	209	3	8	25	7	17	21	26	49	ST-1157 complex	1157	3	3	1
52582	CA44085	UK [Wales]	2015	carrier	Neisseria meningitidis	Y	Y	Y	25	1	1	26	4	10	5	18	9	11	9	17	ST-23 complex	23	4	4	2
49604	CA44086	UK [Wales]	2015	carrier	Neisseria meningitidis	Y	Y	Y	25	1	1	26	4	10	5	18	9	11	9	17	ST-23 complex	23	4	4	2
52583	CA44125	UK [Wales]	2015	carrier	Neisseria meningitidis	NG	X	X	13	3	57	209	3	8	25	7	17	21	26	5	ST-1157 complex	3655	3	3	1
49607	CA44133	UK [Wales]	2015	carrier	Neisseria meningitidis	NG	B	B	13	3	57	145	3	4	10	34	5	198	11	9	ST-269 complex	12577	3	3	1
50292	CA44137	UK [Wales]	2015	carrier	Neisseria meningitidis	NG	cnl	cnl	4	1	1	785	4	5	4	17	15	30	7	12	ST-198 complex	823	4	3	1
49611	CA44168	UK [Wales]	2015	carrier	Neisseria meningitidis	Y	Y	Y	25	1	1	26	4	10	5	18	9	11	9	17	ST-23 complex	23	4	4	2
49612	CA44170	UK [Wales]	2015	carrier	Neisseria meningitidis	NG	E	E	13	3	57	209	3	8	25	12	17	21	26	49	ST-1157 complex	12576	3	3	1
49613	CA44173	UK [Wales]	2015	carrier	Neisseria meningitidis	Y	Y	Y	25	1	1	26	4	12	5	18	9	11	9	17	ST-23 complex	1655	4	4	2
49614	CA44188	UK [Wales]	2015	carrier	Neisseria meningitidis	Y	Y	Y	24	1	1	27	4	2	7	6	17	9	6	8	ST-167 complex	1624	4	4	2

46723	CA44194	UK [Wales]	2015	carrier	Neisseria meningitidis	NG	cnl	cnl	4	1	1	206	4	5	4	17	15	30	7	12	ST-198 complex	823	4	3	1
49615	CA44196	UK [Wales]	2015	carrier	Neisseria meningitidis	Y	Y	Y	25	1	1	26	4	10	5	18	9	11	9	17	ST-23 complex	23	4	4	2
49616	CA44202	UK [Wales]	2015	carrier	Neisseria meningitidis	Y	Y	Y	25	1	1	26	4	12	5	18	9	11	9	17	ST-23 complex	1655	4	4	2
49617	CA44204	UK [Wales]	2015	carrier	Neisseria meningitidis	B	B	B	13	3	57	145	3	4	10	34	5	38	11	9	ST-269 complex	1161	3	3	1
52584	CA44205	UK [Wales]	2015	carrier	Neisseria meningitidis	NG	B	B	45	8	77	87	20	7	5	1	13	36	53	15	ST-213 complex	213	20	4	3
46726	CA44208	UK [Wales]	2015	carrier	Neisseria meningitidis	NG	E	E	13	3	57	209	3	8	25	7	17	21	26	49	ST-1157 complex	1157	3	3	1
49619	CA44230	UK [Wales]	2015	carrier	Neisseria meningitidis	NG	B	B	16	1	4	59	4	4	10	11	18	6	10	12	ST-35 complex	35	4	4	2
49621	CA44254	UK [Wales]	2015	carrier	Neisseria meningitidis	NG	E	E	13	3	1	145	3	175	5	484	17	3	4	124		6798	3	3	1
49622	CA44255	UK [Wales]	2015	carrier	Neisseria meningitidis	Y	Y	Y	25	1	1	26	4	10	5	18	9	11	9	17	ST-23 complex	23	4	4	2
49623	CA44265	UK [Wales]	2015	carrier	Neisseria meningitidis	NG	cnl	cnl	94	157	56	69	NA	5	4	17	15	14	7	12	ST-198 complex	198	NA	NA	3
49624	CA44266	UK [Wales]	2015	carrier	Neisseria meningitidis	B	B	B	13	3	57	145	3	4	10	34	5	198	11	9	ST-269 complex	12577	3	3	1
49625	CA44273	UK [Wales]	2015	carrier	Neisseria meningitidis	NG	W	W	16	1	1	60	4	11	5	18	8	11	4	21	ST-22 complex	184	4	4	2
52585	CA44284	UK [Wales]	2015	carrier	Neisseria meningitidis	Y	Y	Y	25	1	1	26	4	10	5	18	9	11	9	17	ST-23 complex	23	4	4	2
49629	CA44299	UK [Wales]	2015	carrier	Neisseria meningitidis	NG	cnl	cnl	4	1	1	206	4	5	4	17	15	30	7	12	ST-198 complex	823	4	3	1
49631	CA44315	UK [Wales]	2015	carrier	Neisseria meningitidis	Y	Y	Y	25	1	1	26	4	10	5	18	9	11	9	17	ST-23 complex	23	4	4	2
49632	CA44330	UK [Wales]	2015	carrier	Neisseria meningitidis	NG	W	W	22	1	1	1	4	2	3	4	3	8	4	6	ST-11 complex	11	4	4	2
49634	CA44345	UK [Wales]	2015	carrier	Neisseria meningitidis	B	B	B	13	3	57	145	3	4	10	6	5	38	11	9	ST-269 complex	1991	3	3	1
49635	CA44347	UK [Wales]	2015	carrier	Neisseria meningitidis	NG	cnl	cnl	4	1	1	206	4	5	4	17	15	30	7	12	ST-198 complex	823	4	3	1
49636	CA44356	UK [Wales]	2015	carrier	Neisseria meningitidis	NG	cnl	cnl	4	1	1	785	4	5	4	17	15	30	7	12	ST-198 complex	823	4	3	1
49637	CA44357	UK [Wales]	2015	carrier	Neisseria meningitidis	NG	cnl	cnl	4	1	1	785	4	5	4	17	15	30	7	12	ST-198 complex	823	4	3	1
49638	CA44362	UK [Wales]	2015	carrier	Neisseria meningitidis	B	B	B	13	3	57	145	3	4	10	6	5	38	11	9	ST-269 complex	1991	3	3	1
49639	CA44373	UK [Wales]	2015	carrier	Neisseria meningitidis	NG	cnl	cnl	21	1	1	7	4	16	2	159	92	77	25	112	ST-1117 complex	1117	4	4	2
49640	CA44411	UK [Wales]	2015	carrier	Neisseria meningitidis	NG	cnl	cnl	4	1	1	206	4	5	4	17	17	30	7	12	ST-198 complex	2384	4	3	1
49641	CA44414	UK [Wales]	2015	carrier	Neisseria meningitidis	NG	cnl	cnl	4	1	1	206	4	5	4	17	15	30	7	12	ST-198 complex	823	4	3	1
49643	CA44418	UK [Wales]	2015	carrier	Neisseria meningitidis	B	B	B	4	2	1	84	2	3	6	9	5	9	6	9	ST-41/44 complex	41	2	3	1
49644	CA44422	UK [Wales]	2015	carrier	Neisseria meningitidis	NG	Z	Z	16	56	79	60	NA	13	29	2	17	26	65	53		2123	NA	NA	2
49646	CA44497	UK [Wales]	2015	carrier	Neisseria meningitidis	NG	B	B	21	1	1	7	4	1	5	13	53	26	41	3	ST-162 complex	162	4	4	2
49647	CA44553	UK [Wales]	2015	carrier	Neisseria meningitidis	NG	cnl	cnl	985	8	10	787	20	16	2	6	25	17	25	22	ST-53 complex	53	20	4	2
49648	CA44562	UK [Wales]	2015	carrier	Neisseria meningitidis	NG	cnl	cnl	985	8	10	787	20	16	2	6	25	17	25	22	ST-53 complex	53	20	4	2
49649	CA44565	UK [Wales]	2015	carrier	Neisseria meningitidis	Y	Y	Y	25	1	1	26	4	12	5	18	9	11	9	17	ST-23 complex	1655	4	4	2
49650	CA44614	UK [Wales]	2015	carrier	Neisseria meningitidis	Y	Y	Y	25	1	1	26	4	12	5	18	9	11	9	17	ST-23 complex	1655	4	4	2
49651	CA44633	UK [Wales]	2015	carrier	Neisseria meningitidis	Y	Y	Y	24	1	1	27	4	2	7	6	17	9	6	8	ST-167 complex	1624	4	4	2
49652	CA44660	UK [Wales]	2015	carrier	Neisseria meningitidis	Y	Y	Y	25	1	1	26	4	10	5	18	9	11	9	17	ST-23 complex	23	4	4	2
49653	CA44663	UK [Wales]	2015	carrier	Neisseria meningitidis	Y	Y	Y	25	1	1	26	4	10	5	18	9	11	9	17	ST-23 complex	23	4	4	2
49654	CA44695	UK [Wales]	2015	carrier	Neisseria meningitidis	Y	Y	Y	25	1	1	26	4	10	5	18	9	11	9	17	ST-23 complex	23	4	4	2
49655	CA44698	UK [Wales]	2015	carrier	Neisseria meningitidis	Y	Y	Y	25	1	1	26	4	12	5	18	9	11	9	17	ST-23 complex	1655	4	4	2
49657	CA44753	UK [Wales]	2015	carrier	Neisseria meningitidis	Y	Y	Y	24	1	1	27	4	2	4	6	17	817	6	8	ST-167 complex	12641	4	4	2
49658	CA44812	UK [Wales]	2015	carrier	Neisseria meningitidis	NG	E	E	13	3	57	209	3	8	25	7	17	21	26	49	ST-1157 complex	1157	3	3	1
49659	CA44853	UK [Wales]	2015	carrier	Neisseria meningitidis	B	B	B	224	15	1	252	68	4	5	5	4	3	3	8	ST-32 complex	8993	NA	NA	1
49660	CA44881	UK [Wales]	2015	carrier	Neisseria meningitidis	NG	B	B	224	15	1	252	68	4	10	5	4	6	26	8	ST-32 complex	11395	NA	NA	1
49661	CA44925	UK [Wales]	2015	carrier	Neisseria meningitidis	NG	cnl	cnl	94	157	56	69	NA	5	4	17	15	14	7	12	ST-198 complex	198	NA	NA	3
49662	CA44938	UK [Wales]	2015	carrier	Neisseria meningitidis	NG	B	B	202	1	1	35	4	42	583	46	24	819	20	17	ST-282 complex	12642	4	4	2
49663	CA44946	UK [Wales]	2015	carrier	Neisseria meningitidis	B	B	B	45	8	77	87	20	7	5	1	13	36	53	15	ST-213 complex	213	20	4	3
52590	CM40350	UK [England]	2015	carrier	Neisseria meningitidis	B	B	B	14	1	1	142	13	3	6	19	5	83	6	9	ST-41/44 complex	7670	13	3	1
49728	CM40360	UK [England]	2015	carrier	Neisseria meningitidis	NG	E	E	13	3	57	209	3	8	25	7	17	21	26	49	ST-1157 complex	1157	3	3	1
49729	CM40363	UK [England]	2015	carrier	Neisseria meningitidis	NG	Y	Y	23	1	1	4	4	2	7	6	9	16	9	8	ST-167 complex	767	4	4	2
49730	CM40369	UK [England]	2015	carrier	Neisseria meningitidis	NG	Y	Y	25	1	1	26	4	10	5	116	9	11	9	17	ST-23 complex	10294	4	4	2
52592	CM40389	UK [England]	2015	carrier	Neisseria meningitidis	B	B	B	13	3	1	145	3	7	5	1	13	82	53	15	ST-213 complex	575	3	3	1
49733	CM40392	UK [England]	2015	carrier	Neisseria meningitidis	W	W	W	22	1	1	1	4	662	3	4	3	8	4	6	ST-11 complex	10651	4	4	2
52593	CM40400	UK [England]	2015	carrier	Neisseria meningitidis	W	W	W	260	1	1	401	4	11	5	5	8	11	24	21	ST-22 complex	1617	4	3	1
52594	CM40401	UK [England]	2015	carrier	Neisseria meningitidis	NG	Y	Y	25	1	1	26	4	12	5	18	9	11	9	17	ST-23 complex	1655	4	4	2
52595	CM40408	UK [England]	2015	carrier	Neisseria meningitidis	B	B	B	13	3	57	145	3	4	10	34	5	38	11	9	ST-269 complex	1161	3	3	1
49738	CM40410	UK [England]	2015	carrier	Neisseria meningitidis	NG	W	W	22	1	1	1	4	2	3	4	3	8	4	6	ST-11 complex	11	4	4	2
52596	CM40412	UK [England]	2015	carrier	Neisseria meningitidis	NG	Y	Y	25	1	1	26	4	8	3	6	17	29	18	9	ST-103 complex	1418	4	4	2
49740	CM40456	UK [England]	2015	carrier	Neisseria meningitidis	B	B	B	45	8	1	87	20	46	5	9	13	36	53	15	ST-213 complex	12659	20	4	3
52597	CM40462	UK [England]	2015	carrier	Neisseria meningitidis	NG	cnl	cnl	4	1	1	206	4	5	4	17	15	30	7	12	ST-198 complex	823	4	3	1
49742	CM40467	UK [England]	2015	carrier	Neisseria meningitidis	B	cnl	discrepancy	4	1	1	206	4	5	4	17	15	30	7	12	ST-198 complex	823	4	3	1
49744	CM40483	UK [England]	2015	carrier	Neisseria meningitidis	NG	Z	Z	16	1	1	59	4	10	29	2	26	71	3	129		10866	4	4	2
52598	CM40494	UK [England]	2015	carrier	Neisseria meningitidis	NG	B	B	45	8	77	87	20	7	5	1	8	36	53	15	ST-213 complex	2179	20	4	3
52599	CM40500	UK [England]	2015	carrier	Neisseria meningitidis	W	W	W	22	1	1	1	4	2	3	4	3	8	4	6	ST-11 complex	11	4	4	2
49747	CM40508	UK [England]	2015	carrier	Neisseria meningitidis	NG	E	E	13	3	57	209	3	8	25	7	17	21	26	49	ST-1157 complex	1157	3	3	1
49748	CM40509	UK [England]	2015	carrier	Neisseria meningitidis	NG	E	E	13	3	1	145	3	17	5	19	17	3	26	2	ST-60 complex	60	3	3	1
49756	CM40585	UK [England]	2015	carrier	Neisseria meningitidis	B	B	B	13	3	57	145	3	8	105	52	3	9	11	7		1572	3	3	1
49757	CM40605	UK [England]	2015	carrier	Neisseria meningitidis	Y	Y	Y	25	1	1	26	4	10	5	116	9	11	9	17	ST-23 complex	10294	4	4	2
49758	CM40606	UK [England]	2015	carrier	Neisseria meningitidis	NG	E	E	13	3	57	209	3	8	25	7	17	21	26	49	ST-1157 complex	1157	3	3	1
49759	CM40649	UK [England]	2015	carrier	Neisseria meningitidis	NG	Z	Z	16	56	79	60	NA	13	29	2	17	26	65	22		3995	NA	NA	2
49760	CM40658	UK [England]	2015	carrier	Neisseria meningitidis	NG	Y	Y	25	1	1	26	4	10	5	18	9	11	9	17	ST-23 complex	23	4	4	2
50235	CM40689	UK [England]	2015	carrier	Neisseria meningitidis	W	W	W	22	1	1	1	4	2	3	4	3	8	4	6	ST-11 complex	11	4	4	2
49762	CM40705	UK [England]	2015	carrier	Neisseria meningitidis	NG	cnl	cnl	102	8	10	2	20	16	2	6	25	17	25	22	ST-53 complex	53	20	4	2
49764	CM40716	UK [England]	2015	carrier	Neisseria meningitidis	B	B	B	19	1	1	83	4	4	10	1	5	38	11	9	ST-269 complex	12663	4	4	2
49765	CM40717	UK [England]	2015	carrier	Neisseria meningitidis	NG	cnl	cnl	4	1	1	206	4	5	4	17	15	30	7	12	ST-198 complex	823	4	3	1
52601	CM40719	UK [England]	2015	carrier	Neisseria meningitidis	B	B	B	45	8	1	87	20	7	588	1	9	36	53	15	ST-213 complex	12664	20	4	3
49767	CM40734	UK [England]	2015	carrier	Neisseria meningitidis	Y	Y	Y	25	1	1	26	4	10	5	18	9	11	9	17	ST-23 complex	23	4	4	2
49768	CM40740	UK [England]	2015	carrier	Neisseria meningitidis	NG	cnl	cnl	94	58	56	89	NA	5	4	38	15	22	40	13	ST-1136 complex	1136	NA	NA	3
49770	CM40745	UK [England]	2015	carrier	Neisseria meningitidis	NG	E	E	13	3	1	145	3	17	5	19	17	3	26	2	ST-60 complex	60	3	3	1
49771	CM40746	UK [England]	2015	carrier	Neisseria meningitidis	NG	cnl	cnl	94	58	56	89	NA	5	4	38	15	22	40	13	ST-1136 complex	1136	NA	NA	3
49772	CM40767	UK [England]	2015	carrier	Neisseria meningitidis	Y	Y	Y	25	1	1	26	4	12	5	18	9	11	9	17	ST-23 complex	1655	4	4	2
49773	CM40772	UK [England]	2015	carrier	Neisseria meningitidis	NG	Z	Z	16	56	1	60	NA	13	29	2	17	26	65	53		2123	NA	NA	2
49774	CM40805	UK [England]	2015	carrier	Neisseria meningitidis	NG	cnl	cnl	102	8	10	2	20	16	2	6	25	17	25	22	ST-53 complex	53	20	4	2
49775	CM40819	UK [England]	2015	carrier	Neisseria meningitidis	NG	cnl	cnl	102	8	10	2	20	16	2	6	25	17	25	22	ST-53 complex	53	20	4	2
49776	CM40834	UK [England]	2015	carrier	Neisseria meningitidis	NG	Y	Y	23	1	1	4	4	9	7	6	24	11	18	8	ST-167 complex	1760	4	4	2
49777	CM40835	UK [England]	2015	carrier	Neisseria meningitidis	NG	W	W	16	1	1	60	4	11	5	18	8	11	4	21	ST-22 complex	184	4	4	2
49778	CM40843	UK [England]	2015	carrier	Neisseria meningitidis	W	W	W	22	1	1	1	4	2	3	4	3	8	4	6	ST-11 complex	11	4	4	2
49779	CM40847	UK [England]	2015	carrier	Neisseria meningitidis	NG	cnl	cnl	14	8	10	788	20	3	6	9	5	9	6	9	ST-41/44 complex	41	20	3	1
49780	CM40850	UK [England]	2015	carrier	Neisseria meningitidis	Y	Y	Y	25	1	1	26	4	12	5	18	9	11	817	17	ST-23 complex	12666	4	4	2
49781	CM40852	UK [England]	2015	carrier	Neisseria meningitidis	NG	cnl	cnl	102	8	10	2	20	16	2	6	25	17	25	22	ST-53 complex	53	20	4	2
49782	CM40868	UK [England]	2015	carrier	Neisseria meningitidis	NG	E	E	13	NA	1	209	NA	8	25	7	17	21	26	49	ST-1157 complex	1157	NA	NA	1
49783	CM40873	UK [England]	2015	carrier	Neisseria meningitidis	NG	Y	Y	104	1	57	10	4	10	5	18	59	11	9	17	ST-23 complex	183	4	4	2
49784	CM40892	UK [England]	2015	carrier	Neisseria meningitidis	NG	cnl	cnl	102	8	10	2	20	16	2	18	25	17	25	9	ST-53 complex	12667	20	4	2
52602	CM40899	UK [England]	2015	carrier	Neisseria meningitidis	NG	B	B	45	8	77	87	20	7	5	1	13	36	53	195	ST-213 complex	2388	20	4	3
49786	CM40903	UK [England]	2015	carrier	Neisseria meningitidis	NG	E	E	13	3	57	209	3	8	25	7	17	21	26	49	ST-1157 complex	1157	3	3	1
49787	CM40906	UK [England]	2015	carrier	Neisseria meningitidis	NG	cnl	cnl	94	58	56	89	NA	5	4	38	3	22	40	13	ST-1136 complex	4215	NA	NA	3
49788	CM40907	UK [England]	2015	carrier	Neisseria meningitidis	NG	E	E	0	3	57	1074	3	8	25	7	17	21	26	49	ST-1157 complex	1157	3	3	NA
49789	CM40919	UK [England]	2015	carrier	Neisseria meningitidis	Y	Y	Y	25	1	1	26	4	12	5	18	9	11	9	17	ST-23 complex	1655	4	4	2
49790	CM40924	UK [England]	2015	carrier	Neisseria meningitidis	B	B	B	989	1	1	795	4	4	10	2	5	3	11	9	ST-269 complex	1163	4	4	2
49791	CM40933	UK [England]	2015	carrier	Neisseria meningitidis	B	B	B	45	8	77	87	20	7	5	1	13	36	53	15	ST-213 complex	213	20	4	3
49792	CM40940	UK [England]	2015	carrier	Neisseria meningitidis	Y	Y	Y	25	1	1	26	4	10	5	18	9	11	9	17	ST-23 complex	23	4	4	2
49793	CM40955	UK [England]	2015	carrier	Neisseria meningitidis	B	B	B	102	8	10	2	20	7	5	1	13	36	53	15	ST-213 complex	213	20	4	2
49794	CM40987	UK [England]	2015	carrier	Neisseria meningitidis	B	B	B	19	1	1	65	4	8	6	9	9	8	6	9	ST-41/44 complex	1311	4	4	2
49795	CM40995	UK [England]	2015	carrier	Neisseria meningitidis	NG	cnl	cnl	990	8	10	796	20	16	2	6	25	17	25	22	ST-53 complex	53	20	4	2
52603	CM41000	UK [England]	2015	carrier	Neisseria meningitidis	Y	Y	Y	25	1	1	26	4	12	5	18	9	11	9	17	ST-23 complex	1655	4	4	2
49797	CM41001	UK [England]	2015	carrier	Neisseria meningitidis	Y	Y	Y	987	NA	1	797	NA	10	5	18	9	11	9	17	ST-23 complex	23	NA	NA	1
49798	CM41041	UK [England]	2015	carrier	Neisseria meningitidis	NG	cnl	cnl	21	1	1	7	4	16	2	159	9	77	25	112	ST-1117 complex	12668	4	4	2
49799	CM41048	UK [England]	2015	carrier	Neisseria meningitidis	W	W	W	22	1	1	1	4	2	3	4	3	8	4	6	ST-11 complex	11	4	4	2
49800	CM41054	UK [England]	2015	carrier	Neisseria meningitidis	B	B	B	19	1	1	65	4	3	6	9	9	9	6	9	ST-41/44 complex	303	4	4	2
52604	CM41063	UK [England]	2015	carrier	Neisseria meningitidis	NG	Y	Y	25	1	1	26	4	12	5	18	9	11	9	17	ST-23 complex	1655	4	4	2
49802	CM41100	UK [England]	2015	carrier	Neisseria meningitidis	Y	Y	Y	25	1	1	26	4	12	5	18	53	11	9	17	ST-23 complex	11754	4	4	2
49803	CM41101	UK [England]	2015	carrier	Neisseria meningitidis	NG	E	E	13	3	57	209	3	8	25	7	17	21	26	49	ST-1157 complex	1157	3	3	1
52606	CM41104	UK [England]	2015	carrier	Neisseria meningitidis	Y	Y	Y	25	1	1	26	4	12	5	18	9	11	9	17	ST-23 complex	1655	4	4	2
52607	CM41110	UK [England]	2015	carrier	Neisseria meningitidis	B	B	B	47	1	57	90	15	12	5	12	35	192	22	17	ST-461 complex	1946	15	4	3
52608	CM41121	UK [England]	2015	carrier	Neisseria meningitidis	NG	NG	NG	19	1	1	65	4	8	26	9	24	26	20	18	ST-212 complex	212	4	4	2
49808	CM41157	UK [England]	2015	carrier	Neisseria meningitidis	B	B	B	19	1	2	65	4	6	6	63	9	9	11	9	ST-41/44 complex	839	4	4	2
49809	CM41196	UK [England]	2015	carrier	Neisseria meningitidis	Y	Y	Y	25	1	1	26	4	12	5	18	9	11	9	17	ST-23 complex	1655	4	4	2
49810	CM41222	UK [England]	2015	carrier	Neisseria meningitidis	NG	X	X	13	3	1	209	3	8	25	7	17	15	26	49	ST-1157 complex	12073	3	3	1
49811	CM41253	UK [England]	2015	carrier	Neisseria meningitidis	NG	cnl	cnl	334	1	1	527	4	6	41	108	15	9	6	9		845	4	4	2
52609	CM41280	UK [England]	2015	carrier	Neisseria meningitidis	B	B	B	202	1	1	35	4	27	6	9	3	9	6	16	ST-41/44 complex	136	4	4	2
52610	CM41286	UK [England]	2015	carrier	Neisseria meningitidis	NG	NG	NG	16	1	4	59	4	4	586	11	831	6	10	12	ST-35 complex	12670	4	4	2
49814	CM41293	UK [England]	2015	carrier	Neisseria meningitidis	B	B	B	19	1	1	65	4	9	20	9	9	9	6	2	ST-41/44 complex	1097	4	4	2
49815	CM41315	UK [England]	2015	carrier	Neisseria meningitidis	NG	W	W	16	1	1	60	4	11	5	18	8	11	4	21	ST-22 complex	184	4	4	2
49816	CM41316	UK [England]	2015	carrier	Neisseria meningitidis	Y	Y	Y	25	1	1	26	4	12	5	18	9	11	9	17	ST-23 complex	1655	4	4	2
49817	CM41330	UK [England]	2015	carrier	Neisseria meningitidis	Y	Y	Y	104	1	57	10	4	10	5	18	59	11	9	17	ST-23 complex	183	4	4	2
49818	CM41346	UK [England]	2015	carrier	Neisseria meningitidis	NG	B	B	45	91	77	87	172	7	5	1	13	36	53	15	ST-213 complex	213	NA	NA	3
49820	CM41773	UK [England]	2015	carrier	Neisseria meningitidis	NG	C	C	525	98	1	457	122	9	6	9	1	8	820	6		12662	NA	NA	1
49822	CM41807	UK [England]	2015	carrier	Neisseria meningitidis	W	W	W	22	1	1	1	4	2	3	4	3	8	4	6	ST-11 complex	11	4	4	2
49823	CM41829	UK [England]	2015	carrier	Neisseria meningitidis	NG	B	B	911	15	4	798	68	4	10	11	18	6	10	12	ST-35 complex	35	NA	NA	1
52611	CM41832	UK [England]	2015	carrier	Neisseria meningitidis	NG	B	B	24	1	2	25	4	27	6	9	3	9	6	16	ST-41/44 complex	136	4	4	2
52612	CM41841	UK [England]	2015	carrier	Neisseria meningitidis	W	W	W	16	1	1	60	4	11	5	18	8	11	4	21	ST-22 complex	184	4	4	2
49827	CM41853	UK [England]	2015	carrier	Neisseria meningitidis	NG	cnl	cnl	102	8	10	2	20	16	2	6	25	17	25	22	ST-53 complex	53	20	4	2
49828	CM41867	UK [England]	2015	carrier	Neisseria meningitidis	B	B	B	13	3	57	209	3	8	25	7	17	21	26	49	ST-1157 complex	1157	3	3	1
49829	CM41876	UK [England]	2015	carrier	Neisseria meningitidis	NG	B	B	45	8	1	87	20	7	5	1	13	36	53	9	ST-213 complex	3120	20	4	3
49830	CM41889	UK [England]	2015	carrier	Neisseria meningitidis	NG	cnl	cnl	4	1	1	206	4	5	4	17	15	30	650	12	ST-198 complex	10042	4	3	1
52614	CM41908	UK [England]	2015	carrier	Neisseria meningitidis	NG	NG	NG	151	1	1	130	4	6	7	4	56	26	18	8	ST-175 complex	175	4	4	2
49832	CM41947	UK [England]	2015	carrier	Neisseria meningitidis	NG	E	E	13	3	1	145	3	17	5	47	17	3	26	2	ST-60 complex	1430	3	3	1
49833	CM41957	UK [England]	2015	carrier	Neisseria meningitidis	NG	B	B	4	2	1	84	2	3	6	9	5	8	6	9	ST-41/44 complex	485	2	3	1
49834	CM41959	UK [England]	2015	carrier	Neisseria meningitidis	NG	E	E	13	3	57	209	3	8	25	7	17	21	26	49	ST-1157 complex	1157	3	3	1
49835	CM41970	UK [England]	2015	carrier	Neisseria meningitidis	NG	Y	Y	25	1	1	26	4	12	5	18	9	11	9	17	ST-23 complex	1655	4	4	2
49836	CM42002	UK [England]	2015	carrier	Neisseria meningitidis	B	B	B	24	1	63	25	4	27	6	9	3	9	6	16	ST-41/44 complex	136	4	4	2
90	CN100	UK [England]	1941	invasive (unspecified/other)	Neisseria meningitidis	A	A	A	4	37	1	84	125	1	5	1	1	2	16	17		21	NA	NA	1

30246	E84 000091	UK	1984	invasive (unspecified/other)	Neisseria meningitidis	W	W	W	16	1	1	60	4	11	5	18	8	11	24	21	ST-22 complex	22	4	4	2
30247	E84 000967	UK	1984	invasive (unspecified/other)	Neisseria meningitidis	W	W	W	16	1	1	60	4	11	5	18	8	11	24	21	ST-22 complex	22	4	4	2
30239	F85 0000001	UK	1985	invasive (unspecified/other)	Neisseria meningitidis	C	C	C	4	2	1	84	2	2	3	4	3	8	4	6	ST-11 complex	11	2	3	1
30240	F85 0000045	UK	1985	invasive (unspecified/other)	Neisseria meningitidis	C	C	C	22	1	1	1	4	2	3	4	3	8	4	6	ST-11 complex	11	4	4	2
30241	F85 0000076	UK	1985	invasive (unspecified/other)	Neisseria meningitidis	C	C	C	22	1	1	1	4	2	3	4	3	8	4	6	ST-11 complex	11	4	4	2
30242	F85 0000171	UK	1985	invasive (unspecified/other)	Neisseria meningitidis	C	C	C	22	1	1	1	4	2	3	4	3	8	4	6	ST-11 complex	11	4	4	2
30243	F85 0000671	UK	1985	invasive (unspecified/other)	Neisseria meningitidis	C	C	C	22	1	1	1	4	2	3	4	3	8	4	6	ST-11 complex	11	4	4	2
30244	F85 0000704	UK	1985	invasive (unspecified/other)	Neisseria meningitidis	C	C	C	22	1	1	1	4	2	3	4	3	8	4	6	ST-11 complex	11	4	4	2
30245	F85 0000746	UK	1985	invasive (unspecified/other)	Neisseria meningitidis	C	C	C	22	1	1	1	4	2	3	4	3	8	4	6	ST-11 complex	11	4	4	2
30248	F85 000311	UK	1985	invasive (unspecified/other)	Neisseria meningitidis	W	W	W	21	1	1	7	4	12	16	34	17	5	38	17		1251	4	4	2
30249	F85 001122	UK	1985	invasive (unspecified/other)	Neisseria meningitidis	W	W	W	21	1	1	7	4	12	5	34	17	5	38	17	ST-174 complex	185	4	4	2
638	G2136	UK [England]	1986	invasive (unspecified/other)	Neisseria meningitidis	B	B	B	16	1	4	59	4	NA	NA	NA	NA	NA	NA	NA		NA	4	4	2
30250	G86 000031	UK	1986	invasive (unspecified/other)	Neisseria meningitidis	W	Y	discrepancy	16	1	1	60	4	11	5	18	17	11	24	21	ST-22 complex	114	4	4	2
46544	GL40048	UK [Scotland]	2014	carrier	Neisseria meningitidis	NG	cnl	cnl	94	58	56	89	NA	5	4	38	15	22	40	13	ST-1136 complex	1136	NA	NA	3
50237	GL40059	UK [Scotland]	2014	carrier	Neisseria meningitidis	NG	B	B	45	8	77	87	20	7	5	1	13	36	53	15	ST-213 complex	213	20	4	3
46545	GL40066	UK [Scotland]	2014	carrier	Neisseria meningitidis	Y	Y	Y	25	1	1	26	4	12	5	18	9	11	9	17	ST-23 complex	1655	4	4	2
46546	GL40077	UK [Scotland]	2014	carrier	Neisseria meningitidis	NG	cnl	cnl	4	1	1	206	4	5	4	17	15	30	7	12	ST-198 complex	823	4	3	1
49664	GL40093	UK [Scotland]	2014	carrier	Neisseria meningitidis	Y	Y	Y	25	1	1	26	4	12	5	18	9	11	9	17	ST-23 complex	1655	4	4	2
46548	GL40098	UK [Scotland]	2014	carrier	Neisseria meningitidis	NG	cnl	cnl	94	58	56	89	NA	5	4	38	15	22	40	13	ST-1136 complex	1136	NA	NA	3
46549	GL40107	UK [Scotland]	2014	carrier	Neisseria meningitidis	NG	cnl	cnl	94	157	56	69	NA	5	4	17	15	14	7	12	ST-198 complex	198	NA	NA	3
46550	GL40125	UK [Scotland]	2014	carrier	Neisseria meningitidis	NG	B	B	45	8	77	87	20	788	5	1	13	36	53	15	ST-213 complex	12403	20	4	3
49665	GL40131	UK [Scotland]	2014	carrier	Neisseria meningitidis	NG	cnl	cnl	102	8	10	2	20	16	2	6	25	17	25	22	ST-53 complex	53	20	4	2
44229	GL40142	UK [Scotland]	2014	carrier	Neisseria meningitidis	B	B	B	24	1	4	25	4	4	10	47	17	6	2	12	ST-35 complex	278	4	4	2
46553	GL40151	UK [Scotland]	2014	carrier	Neisseria meningitidis	NG	cnl	cnl	94	58	56	89	NA	5	4	38	15	22	40	13	ST-1136 complex	1136	NA	NA	3
46554	GL40172	UK [Scotland]	2014	carrier	Neisseria meningitidis	Y	Y	Y	25	1	1	26	4	12	5	18	9	11	9	17	ST-23 complex	1655	4	4	2
46555	GL40182	UK [Scotland]	2014	carrier	Neisseria meningitidis	Y	Y	Y	25	1	1	26	4	12	5	18	9	11	9	17	ST-23 complex	1655	4	4	2
46556	GL40215	UK [Scotland]	2014	carrier	Neisseria meningitidis	NG	cnl	cnl	102	8	10	2	20	16	2	6	25	17	25	22	ST-53 complex	53	20	4	2
46559	GL40301	UK [Scotland]	2014	carrier	Neisseria meningitidis	NG	cnl	cnl	94	157	56	69	NA	5	4	17	15	14	7	12	ST-198 complex	198	NA	NA	3
46560	GL40386	UK [Scotland]	2014	carrier	Neisseria meningitidis	NG	Y	Y	25	1	1	26	4	12	5	18	9	11	9	17	ST-23 complex	1655	4	4	2
46561	GL40393	UK [Scotland]	2014	carrier	Neisseria meningitidis	NG	E	E	13	3	1	209	3	8	25	7	17	21	26	49	ST-1157 complex	1157	3	3	1
46562	GL40407	UK [Scotland]	2014	carrier	Neisseria meningitidis	NG	E	E	13	3	57	209	3	789	25	7	801	21	26	49	ST-1157 complex	12420	3	3	1
46566	GL40545	UK [Scotland]	2014	carrier	Neisseria meningitidis	Y	Y	Y	25	1	1	26	4	10	5	18	9	11	9	17	ST-23 complex	23	4	4	2
46567	GL40559	UK [Scotland]	2014	carrier	Neisseria meningitidis	NG	E	E	13	3	1	145	3	17	5	19	17	3	26	2	ST-60 complex	60	3	3	1
46568	GL40560	UK [Scotland]	2014	carrier	Neisseria meningitidis	NG	X	X	13	3	57	209	3	8	5	7	17	21	26	2	ST-1157 complex	3135	3	3	1
46569	GL40564	UK [Scotland]	2014	carrier	Neisseria meningitidis	NG	cnl	cnl	94	157	56	746	NA	5	4	17	15	14	7	12	ST-198 complex	198	NA	NA	3
46570	GL40565	UK [Scotland]	2014	carrier	Neisseria meningitidis	B	B	B	19	1	1	748	4	3	6	9	24	9	38	9	ST-41/44 complex	1414	4	4	2
46571	GL40575	UK [Scotland]	2014	carrier	Neisseria meningitidis	NG	E	E	13	3	1	145	3	17	5	19	17	3	26	33	ST-60 complex	5022	3	3	1
49666	GL40577	UK [Scotland]	2014	carrier	Neisseria meningitidis	NG	E	E	13	3	57	209	3	8	25	7	17	21	26	49	ST-1157 complex	1157	3	3	1
46573	GL40580	UK [Scotland]	2014	carrier	Neisseria meningitidis	NG	E	E	13	3	57	209	3	8	25	7	17	21	26	49	ST-1157 complex	1157	3	3	1
46575	GL40585	UK [Scotland]	2014	carrier	Neisseria meningitidis	Y	Y	Y	25	1	1	26	4	12	5	18	9	11	9	17	ST-23 complex	1655	4	4	2
46576	GL40586	UK [Scotland]	2014	carrier	Neisseria meningitidis	Y	Y	Y	25	1	1	26	4	12	5	18	9	11	9	17	ST-23 complex	1655	4	4	2
52616	GL40616	UK [Scotland]	2014	carrier	Neisseria meningitidis	NG	B	B	16	1	4	59	4	4	10	161	9	6	21	3		1167	4	4	2
46578	GL40632	UK [Scotland]	2014	carrier	Neisseria meningitidis	NG	E	E	13	3	57	209	3	8	25	7	17	21	26	49	ST-1157 complex	1157	3	3	1
50293	GL40646	UK [Scotland]	2014	carrier	Neisseria meningitidis	NG	Y	Y	25	1	1	26	4	12	5	18	9	11	9	17	ST-23 complex	1655	4	4	2
46581	GL40674	UK [Scotland]	2014	carrier	Neisseria meningitidis	NG	cnl	cnl	102	8	10	2	20	16	2	6	25	17	25	22	ST-53 complex	53	20	4	2
49668	GL40744	UK [Scotland]	2014	carrier	Neisseria meningitidis	NG	cnl	cnl	4	1	1	206	4	5	210	17	15	30	7	12	ST-198 complex	4247	4	3	1
49669	GL40798	UK [Scotland]	2014	carrier	Neisseria meningitidis	B	B	B	24	1	4	27	4	4	10	72	9	6	10	16	ST-35 complex	12644	4	4	2
46585	GL40812	UK [Scotland]	2014	carrier	Neisseria meningitidis	NG	cnl	cnl	21	1	1	7	4	16	2	159	92	77	25	112	ST-1117 complex	1117	4	4	2
49670	GL40827	UK [Scotland]	2014	carrier	Neisseria meningitidis	B	B	B	19	1	1	65	4	4	6	15	9	8	11	9	ST-269 complex	1284	4	4	2
50294	GL40845	UK [Scotland]	2014	carrier	Neisseria meningitidis	NG	cnl	cnl	21	1	1	7	4	16	2	159	92	77	25	112	ST-1117 complex	1117	4	4	2
49672	GL40854	UK [Scotland]	2014	carrier	Neisseria meningitidis	NG	NG	NG	125	1	57	268	13	7	16	55	5	3	56	7	ST-178 complex	12846	13	3	1
46589	GL40868	UK [Scotland]	2014	carrier	Neisseria meningitidis	NG	cnl	cnl	21	1	1	7	4	16	2	159	92	77	25	112	ST-1117 complex	1117	4	4	2
49673	GL40874	UK [Scotland]	2014	carrier	Neisseria meningitidis	Y	Y	Y	25	1	1	26	4	12	5	18	9	11	9	17	ST-23 complex	1655	4	4	2
46591	GL40886	UK [Scotland]	2014	carrier	Neisseria meningitidis	B	B	B	19	1	2	65	4	4	10	15	9	8	11	9	ST-269 complex	269	4	4	2
46592	GL40903	UK [Scotland]	2014	carrier	Neisseria meningitidis	NG	cnl	cnl	94	58	56	89	NA	5	4	38	15	22	40	13	ST-1136 complex	1136	NA	NA	3
46593	GL40915	UK [Scotland]	2014	carrier	Neisseria meningitidis	Y	Y	Y	25	1	1	26	4	10	5	18	9	11	9	17	ST-23 complex	23	4	4	2
46594	GL40916	UK [Scotland]	2014	carrier	Neisseria meningitidis	NG	cnl	cnl	102	8	1	2	20	16	2	6	25	17	25	22	ST-53 complex	53	20	4	2
46595	GL40926	UK [Scotland]	2014	carrier	Neisseria meningitidis	Y	Y	Y	25	1	1	26	4	10	5	18	9	11	9	17	ST-23 complex	23	4	4	2
46596	GL40928	UK [Scotland]	2014	carrier	Neisseria meningitidis	Y	Y	Y	25	1	1	26	4	12	5	18	9	11	9	17	ST-23 complex	1655	4	4	2
46597	GL40965	UK [Scotland]	2014	carrier	Neisseria meningitidis	NG	cnl	cnl	94	58	56	89	NA	5	4	38	15	22	40	13	ST-1136 complex	1136	NA	NA	3
49674	GL40966	UK [Scotland]	2014	carrier	Neisseria meningitidis	Y	Y	Y	25	1	1	26	4	12	5	18	9	11	9	17	ST-23 complex	1655	4	4	2
46599	GL41006	UK [Scotland]	2014	carrier	Neisseria meningitidis	NG	Z	Z	16	56	NA	60	NA	13	29	2	17	26	65	53		2123	NA	NA	2
52618	GL41017	UK [Scotland]	2014	carrier	Neisseria meningitidis	B	B	B	19	1	1	65	4	3	6	9	60	9	20	9	ST-41/44 complex	2012	4	4	2
46602	GL41053	UK [Scotland]	2014	carrier	Neisseria meningitidis	NG	E	E	13	3	57	145	3	2	16	12	11	3	60	7	ST-254 complex	254	3	3	1
44217	GL41064	UK [Scotland]	2014	carrier	Neisseria meningitidis	NG	cnl	cnl	102	8	1	2	20	16	2	6	25	17	25	22	ST-53 complex	53	20	4	2
46603	GL41107	UK [Scotland]	2014	carrier	Neisseria meningitidis	B	B	B	19	1	1	83	4	4	10	15	5	NA	NA	NA		NA	4	4	2
46604	GL41113	UK [Scotland]	2014	carrier	Neisseria meningitidis	B	B	B	19	1	1	83	4	4	10	15	5	3	11	9	ST-269 complex	2561	4	4	2
49676	GL41128	UK [Scotland]	2014	carrier	Neisseria meningitidis	Y	Y	Y	25	1	1	26	4	12	5	18	9	11	9	17	ST-23 complex	1655	4	4	2
44216	GL41142	UK [Scotland]	2014	carrier	Neisseria meningitidis	Y	Y	Y	25	1	1	26	4	12	5	18	9	11	9	17	ST-23 complex	1655	4	4	2
44215	GL41152	UK [Scotland]	2014	carrier	Neisseria meningitidis	NG	E	E	13	3	57	209	3	8	25	7	17	21	26	49	ST-1157 complex	1157	3	3	1
52619	GL41176	UK [Scotland]	2014	carrier	Neisseria meningitidis	NG	Y	Y	104	1	57	10	4	10	5	18	59	11	9	17	ST-23 complex	183	4	4	2
46607	GL41183	UK [Scotland]	2014	carrier	Neisseria meningitidis	Y	Y	Y	23	1	1	4	4	9	7	6	17	11	18	8	ST-167 complex	884	4	4	2
46608	GL41200	UK [Scotland]	2014	carrier	Neisseria meningitidis	NG	C	C	208	1	1	701	4	46	29	186	176	67	175	96		1975	4	3	1
46609	GL41208	UK [Scotland]	2014	carrier	Neisseria meningitidis	NG	cnl	cnl	102	8	10	2	20	790	2	6	25	17	25	22	ST-53 complex	12415	20	4	2
46610	GL41212	UK [Scotland]	2014	carrier	Neisseria meningitidis	NG	cnl	cnl	102	8	10	2	20	790	2	6	25	17	25	22	ST-53 complex	12415	20	4	2
46611	GL41235	UK [Scotland]	2014	carrier	Neisseria meningitidis	Y	Y	Y	25	1	1	26	4	12	5	18	332	11	9	17	ST-23 complex	11280	4	4	2
46612	GL41239	UK [Scotland]	2014	carrier	Neisseria meningitidis	B	B	B	19	1	1	83	4	4	10	15	5	3	11	9	ST-269 complex	2561	4	4	2
46613	GL41245	UK [Scotland]	2014	carrier	Neisseria meningitidis	Y	Y	Y	25	1	1	26	4	12	5	18	9	11	9	17	ST-23 complex	1655	4	4	2
46614	GL41254	UK [Scotland]	2014	carrier	Neisseria meningitidis	NG	E	E	13	3	1	145	3	17	5	19	17	62	26	2	ST-60 complex	10454	3	3	1
46615	GL41261	UK [Scotland]	2014	carrier	Neisseria meningitidis	NG	Y	Y	27	1	1	749	4	2	7	6	17	9	18	8	ST-167 complex	766	4	4	2
46616	GL41316	UK [Scotland]	2014	carrier	Neisseria meningitidis	NG	Z	Z	25	1	1	26	4	2	4	34	17	5	18	2	ST-103 complex	12407	4	4	2
46617	GL41339	UK [Scotland]	2014	carrier	Neisseria meningitidis	NG	cnl	cnl	4	1	1	206	4	5	4	17	15	30	7	12	ST-198 complex	823	4	3	1
46618	GL41342	UK [Scotland]	2014	carrier	Neisseria meningitidis	NG	Z	Z	16	56	79	60	NA	13	29	2	17	26	65	53		2123	NA	NA	2
46619	GL41357	UK [Scotland]	2014	carrier	Neisseria meningitidis	NG	cnl	cnl	968	157	56	707	NA	5	4	17	15	14	7	12	ST-198 complex	198	NA	NA	3
46620	GL41367	UK [Scotland]	2014	carrier	Neisseria meningitidis	W	W	W	22	1	1	1	4	2	3	4	3	8	4	6	ST-11 complex	11	4	4	2
46621	GL41379	UK [Scotland]	2014	carrier	Neisseria meningitidis	Y	Y	Y	25	1	1	26	4	12	5	18	9	11	9	17	ST-23 complex	1655	4	4	2
46623	GL41392	UK [Scotland]	2014	carrier	Neisseria meningitidis	NG	Z	Z	16	56	79	60	NA	13	29	2	17	26	65	53		2123	NA	NA	2
46624	GL41410	UK [Scotland]	2014	carrier	Neisseria meningitidis	NG	Y	Y	104	1	57	10	4	10	5	18	59	11	9	17	ST-23 complex	183	4	4	2
46625	GL41483	UK [Scotland]	2014	carrier	Neisseria meningitidis	NG	B	B	16	1	2	60	4	222	3	58	261	263	5	255	ST-4821 complex	3200	4	4	2
46626	GL41514	UK [Scotland]	2014	carrier	Neisseria meningitidis	NG	C	C	15	NA	2	193	NA	4	10	15	9	8	11	13	ST-269 complex	467	NA	NA	1
46627	GL41521	UK [Scotland]	2014	carrier	Neisseria meningitidis	NG	cnl	cnl	94	58	56	89	NA	5	4	38	15	22	40	13	ST-1136 complex	1136	NA	NA	3
46628	GL41537	UK [Scotland]	2014	carrier	Neisseria meningitidis	Y	Y	Y	25	1	1	26	4	10	5	18	9	11	9	17	ST-23 complex	23	4	4	2
50296	GL41538	UK [Scotland]	2014	carrier	Neisseria meningitidis	NG	X	X	16	1	1	59	4	8	3	19	17	62	21	206		12228	4	4	2
46630	GL41574	UK [Scotland]	2014	carrier	Neisseria meningitidis	B	B	B	106	1	1	146	4	4	10	4	8	8	11	8	ST-269 complex	2187	4	4	2
46631	GL41577	UK [Scotland]	2014	carrier	Neisseria meningitidis	NG	Z	Z	13	3	57	209	3	4	25	7	17	21	26	49	ST-1157 complex	12410	3	3	1
46632	GL41581	UK [Scotland]	2014	carrier	Neisseria meningitidis	NG	cnl	cnl	102	8	10	2	20	16	2	6	25	17	25	22	ST-53 complex	53	20	4	2
46634	GL41685	UK [Scotland]	2014	carrier	Neisseria meningitidis	Y	Y	Y	25	1	1	26	4	12	5	18	9	11	9	17	ST-23 complex	1655	4	4	2
46635	GL41705	UK [Scotland]	2014	carrier	Neisseria meningitidis	NG	B	B	16	1	4	59	4	4	10	11	18	6	10	12	ST-35 complex	35	4	4	2
46637	GL41768	UK [Scotland]	2015	carrier	Neisseria meningitidis	Y	Y	Y	25	1	1	26	4	12	5	18	9	11	9	17	ST-23 complex	1655	4	4	2
46638	GL41769	UK [Scotland]	2015	carrier	Neisseria meningitidis	Y	Y	Y	25	1	1	26	4	12	5	18	9	11	9	17	ST-23 complex	1655	4	4	2
46639	GL41776	UK [Scotland]	2015	carrier	Neisseria meningitidis	NG	B	B	13	3	57	209	3	7	5	1	277	36	53	15	ST-213 complex	3633	3	3	1
46641	GL41793	UK [Scotland]	2015	carrier	Neisseria meningitidis	W	W	W	16	1	1	60	4	11	5	18	8	11	24	21	ST-22 complex	22	4	4	2
46642	GL41802	UK [Scotland]	2015	carrier	Neisseria meningitidis	B	B	B	19	1	2	65	4	6	6	63	9	9	11	9	ST-41/44 complex	839	4	4	2
46643	GL41804	UK [Scotland]	2015	carrier	Neisseria meningitidis	Y	Y	Y	16	1	1	60	4	11	5	18	17	11	24	21	ST-22 complex	114	4	4	2
46644	GL41807	UK [Scotland]	2015	carrier	Neisseria meningitidis	Y	Y	Y	16	1	1	60	4	11	5	18	17	11	24	21	ST-22 complex	114	4	4	2
46645	GL41827	UK [Scotland]	2015	carrier	Neisseria meningitidis	W	W	W	16	1	1	60	4	11	5	18	8	11	24	21	ST-22 complex	22	4	4	2
46646	GL41845	UK [Scotland]	2015	carrier	Neisseria meningitidis	NG	B	B	19	1	1	65	4	3	6	9	9	9	6	9	ST-41/44 complex	303	4	4	2
46647	GL41846	UK [Scotland]	2015	carrier	Neisseria meningitidis	NG	E	E	13	3	57	209	3	8	25	7	542	21	26	826	ST-1157 complex	12430	3	3	1
46648	GL41861	UK [Scotland]	2015	carrier	Neisseria meningitidis	NG	cnl	cnl	1021	8	1	751	20	16	2	6	25	17	25	22	ST-53 complex	53	20	4	2
46649	GL41866	UK [Scotland]	2015	carrier	Neisseria meningitidis	W	W	W	16	1	1	60	4	11	5	18	8	11	24	21	ST-22 complex	22	4	4	2
46650	GL41886	UK [Scotland]	2015	carrier	Neisseria meningitidis	B	B	B	19	1	1	65	4	3	6	9	9	9	6	9	ST-41/44 complex	303	4	4	2
50297	GL41889	UK [Scotland]	2015	carrier	Neisseria meningitidis	Y	Y	Y	16	1	1	60	4	11	5	18	17	11	24	21	ST-22 complex	114	4	4	2
46652	GL41898	UK [Scotland]	2015	carrier	Neisseria meningitidis	Y	Y	Y	25	1	1	26	4	12	5	18	9	11	9	17	ST-23 complex	1655	4	4	2
46653	GL41900	UK [Scotland]	2015	carrier	Neisseria meningitidis	Y	Y	Y	25	1	1	26	4	12	5	18	9	11	9	17	ST-23 complex	1655	4	4	2
46656	GL41924	UK [Scotland]	2015	carrier	Neisseria meningitidis	NG	B	B	23	250	1	431	NA	8	5	15	17	8	21	2	ST-865 complex	865	NA	NA	2
46657	GL41940	UK [Scotland]	2015	carrier	Neisseria meningitidis	Y	Y	Y	25	1	1	26	4	12	5	18	9	11	9	17	ST-23 complex	1655	4	4	2
46659	GL41944	UK [Scotland]	2015	carrier	Neisseria meningitidis	Y	Y	Y	25	1	1	26	4	12	5	18	9	11	9	17	ST-23 complex	1655	4	4	2
46660	GL41970	UK [Scotland]	2015	carrier	Neisseria meningitidis	NG	cnl	cnl	4	1	1	206	4	5	4	17	15	30	7	12	ST-198 complex	823	4	3	1
46661	GL41990	UK [Scotland]	2015	carrier	Neisseria meningitidis	Y	Y	Y	27	1	1	749	4	2	7	6	17	9	18	8	ST-167 complex	766	4	4	2
46662	GL42005	UK [Scotland]	2015	carrier	Neisseria meningitidis	Y	Y	Y	25	1	1	26	4	12	5	18	9	11	9	17	ST-23 complex	1655	4	4	2
46663	GL42015	UK [Scotland]	2015	carrier	Neisseria meningitidis	NG	cnl	cnl	21	1	1	7	4	16	2	159	92	182	25	112	ST-1117 complex	2445	4	4	2
49678	GL42028	UK [Scotland]	2015	carrier	Neisseria meningitidis	B	B	B	45	8	77	87	20	7	5	1	13	36	53	15	ST-213 complex	213	20	4	3
46664	GL42029	UK [Scotland]	2015	carrier	Neisseria meningitidis	NG	B	B	45	8	77	87	20	7	5	1	13	36	53	15	ST-213 complex	213	20	4	3
46665	GL42034	UK [Scotland]	2015	carrier	Neisseria meningitidis	NG	B	B	226	66	4	228	38	4	10	11	18	6	10	12	ST-35 complex	35	NA	NA	1
46666	GL42044	UK [Scotland]	2015	carrier	Neisseria meningitidis	NG	cnl	cnl	94	58	56	89	NA	5	4	38	15	22	40	13	ST-1136 complex	1136	NA	NA	3
46667	GL42049	UK [Scotland]	2015	carrier	Neisseria meningitidis	NG	cnl	cnl	21	1	4	7	4	132	3	19	17	62	25	2		12412	4	4	2
50298	GL42097	UK [Scotland]	2015	carrier	Neisseria meningitidis	NG	W	W	16	1	1	60	4	11	5	18	8	11	4	21	ST-22 complex	184	4	4	2
46669	GL42112	UK [Scotland]	2015	carrier	Neisseria meningitidis	NG	cnl	cnl	102	8	10	2	20	16	2	6	25	799	25	22	ST-53 complex	12440	20	4	2
52623	GL42143	UK [Scotland]	2015	carrier	Neisseria meningitidis	B	B	B	19	1	1	65	4	8	6	9	9	9	6	9	ST-41/44 complex	146	4	4	2
50299	GL42166	UK [Scotland]	2015	carrier	Neisseria meningitidis	NG	E	E	13	3	57	209	3	8	25	7	17	21	26	49	ST-1157 complex	1157	3	3	1
52624	GL42194	UK [Scotland]	2015	carrier	Neisseria meningitidis	NG	B	B	106	1	1	146	4	4	10	4	8	8	11	8	ST-269 complex	2187	4	4	2
46675	GL42200	UK [Scotland]	2015	carrier	Neisseria meningitidis	NG	cnl	cnl	102	8	10	2	20	16	2	6	25	17	25	22	ST-53 complex	53	20	4	2
46676	GL42220	UK [Scotland]	2015	carrier	Neisseria meningitidis	NG	X	X	25	1	1	26	4	9	4	6	17	5	18	2	ST-103 complex	12413	4	4	2
46677	GL42226	UK [Scotland]	2015	carrier	Neisseria meningitidis	NG	X	X	13	3	57	209	3	8	25	7	17	21	26	49	ST-1157 complex	1157	3	3	1
46678	GL42229	UK [Scotland]	2015	carrier	Neisseria meningitidis	NG	Y	Y	21	1	1	7	4	6	5	173	13	5	24	17	ST-174 complex	1466	4	4	2
46679	GL42248	UK [Scotland]	2015	carrier	Neisseria meningitidis	NG	X	X	25	1	1	26	4	9	4	6	17	5	18	2	ST-103 complex	12413	4	4	2
52625	GL42501	UK [Scotland]	2015	carrier	Neisseria meningitidis	NG	B	B	106	1	1	146	4	4	10	4	8	8	11	8	ST-269 complex	2187	4	4	2
46681	GL42505	UK [Scotland]	2015	carrier	Neisseria meningitidis	W	W	W	22	1	1	1	4	2	3	4	3	8	4	6	ST-11 complex	11	4	4	2

46682	GL42509	UK [Scotland]	2015	carrier	Neisseria meningitidis	Y	Y	Y	25	1	1	26	4	12	5	18	9	11	9	17	ST-23 complex	1655	4	4	2
46684	GL42536	UK [Scotland]	2015	carrier	Neisseria meningitidis	NG	cnl	cnl	94	58	56	89	NA	5	558	38	15	22	40	13	ST-1136 complex	12402	NA	NA	3
50300	GL42557	UK [Scotland]	2015	carrier	Neisseria meningitidis	NG	Y	Y	25	1	1	26	4	11	5	18	8	11	4	21	ST-22 complex	184	4	4	2
50301	GL42561	UK [Scotland]	2015	carrier	Neisseria meningitidis	NG	Y	Y	25	1	1	26	4	10	5	18	9	11	9	17	ST-23 complex	23	4	4	2
46688	GL42572	UK [Scotland]	2015	carrier	Neisseria meningitidis	NG	cnl	cnl	102	8	10	2	20	16	2	6	25	17	25	22	ST-53 complex	53	20	4	2
46689	GL42575	UK [Scotland]	2015	carrier	Neisseria meningitidis	NG	cnl	cnl	94	58	56	89	NA	5	4	38	15	22	40	13	ST-1136 complex	1136	NA	NA	3
46690	GL42583	UK [Scotland]	2015	carrier	Neisseria meningitidis	Y	Y	Y	25	1	1	26	4	10	5	18	9	11	9	17	ST-23 complex	23	4	4	2
52626	GL42588	UK [Scotland]	2015	carrier	Neisseria meningitidis	Y	Y	Y	25	1	1	26	4	12	5	18	9	11	801	17	ST-23 complex	12414	4	4	2
50302	GL42616	UK [Scotland]	2015	carrier	Neisseria meningitidis	NG	Y	Y	25	1	1	26	4	12	5	18	9	11	9	17	ST-23 complex	1655	4	4	2
46693	GL42618	UK [Scotland]	2015	carrier	Neisseria meningitidis	NG	cnl	cnl	102	8	10	2	20	16	2	6	25	17	25	22	ST-53 complex	53	20	4	2
46694	GL42620	UK [Scotland]	2015	carrier	Neisseria meningitidis	NG	B	B	25	1	1	26	4	8	5	15	56	9	21	2	ST-865 complex	4237	4	4	2
50303	GL42621	UK [Scotland]	2015	carrier	Neisseria meningitidis	Y	Y	Y	25	1	1	26	4	12	5	18	9	11	9	17	ST-23 complex	1655	4	4	2
46696	GL42629	UK [Scotland]	2015	carrier	Neisseria meningitidis	Y	Y	Y	13	3	57	209	3	12	5	18	332	11	9	17	ST-23 complex	11280	3	3	1
50304	GL42643	UK [Scotland]	2015	carrier	Neisseria meningitidis	Y	Y	Y	25	1	1	26	4	12	5	18	9	11	9	17	ST-23 complex	1655	4	4	2
50305	GL42673	UK [Scotland]	2015	carrier	Neisseria meningitidis	Y	Y	Y	25	1	1	26	4	10	5	18	9	11	9	17	ST-23 complex	23	4	4	2
50306	GL42674	UK [Scotland]	2015	carrier	Neisseria meningitidis	NG	Y	Y	25	1	1	26	4	10	5	18	9	11	9	17	ST-23 complex	23	4	4	2
46700	GL42680	UK [Scotland]	2015	carrier	Neisseria meningitidis	NG	B	B	19	1	1	65	4	9	6	9	9	9	64	9	ST-41/44 complex	409	4	4	2
46701	GL42688	UK [Scotland]	2015	carrier	Neisseria meningitidis	NG	X	X	13	3	57	209	3	8	5	7	17	21	26	2	ST-1157 complex	3135	3	3	1
52628	GL42697	UK [Scotland]	2015	carrier	Neisseria meningitidis	NG	B	B	45	8	77	87	20	7	5	771	13	36	53	15	ST-213 complex	11221	20	4	3
46704	GL42705	UK [Scotland]	2015	carrier	Neisseria meningitidis	NG	X	X	13	3	57	209	3	8	5	7	17	21	26	2	ST-1157 complex	3135	3	3	1
50307	GL42707	UK [Scotland]	2015	carrier	Neisseria meningitidis	B	B	B	202	1	1	35	4	42	26	46	24	615	20	17	ST-282 complex	11521	4	4	2
52629	GL42743	UK [Scotland]	2015	carrier	Neisseria meningitidis	B	B	B	14	30	1	142	71	3	6	9	5	111	6	9	ST-41/44 complex	7385	NA	NA	1
52630	GL42747	UK [Scotland]	2015	carrier	Neisseria meningitidis	B	B	B	208	1	1	701	4	46	29	186	176	67	175	96		1975	4	3	1
46708	GL42762	UK [Scotland]	2015	carrier	Neisseria meningitidis	NG	E	E	13	3	57	145	3	2	16	12	11	123	22	6	ST-254 complex	1827	3	3	1
50308	GL42770	UK [Scotland]	2015	carrier	Neisseria meningitidis	NG	Y	Y	456	73	1	243	56	12	5	18	9	11	9	17	ST-23 complex	1655	NA	NA	1
46710	GL42773	UK [Scotland]	2015	carrier	Neisseria meningitidis	NG	E	E	584	38	1	108	42	6	5	9	17	5	24	8	ST-174 complex	11445	NA	NA	2
49681	GL42775	UK [Scotland]	2015	carrier	Neisseria meningitidis	B	B	B	45	8	77	87	20	7	5	771	13	36	53	15	ST-213 complex	11221	20	4	3
50309	GL42781	UK [Scotland]	2015	carrier	Neisseria meningitidis	Y	Y	Y	25	1	1	26	4	10	5	18	9	11	9	17	ST-23 complex	23	4	4	2
46713	GL42830	UK [Scotland]	2015	carrier	Neisseria meningitidis	NG	E	E	13	3	57	209	3	8	25	7	17	21	26	49	ST-1157 complex	1157	3	3	1
46716	GL42849	UK [Scotland]	2015	carrier	Neisseria meningitidis	NG	cnl	cnl	4	1	1	206	4	5	4	17	15	30	7	12	ST-198 complex	823	4	3	1
52631	GL42872	UK [Scotland]	2015	carrier	Neisseria meningitidis	B	B	B	45	8	77	87	20	7	5	1	13	36	53	15	ST-213 complex	213	20	4	3
46718	GL42874	UK [Scotland]	2015	carrier	Neisseria meningitidis	NG	cnl	cnl	102	8	10	2	20	16	2	6	25	17	802	22	ST-53 complex	12418	20	4	2
46719	GL42890	UK [Scotland]	2015	carrier	Neisseria meningitidis	NG	E	E	13	3	1	209	3	8	25	7	17	21	26	49	ST-1157 complex	1157	3	3	1
52632	GL42894	UK [Scotland]	2015	carrier	Neisseria meningitidis	B	B	B	24	1	2	25	4	27	6	9	10	9	6	16	ST-41/44 complex	2009	4	4	2
210	H1964	UK	1987	invasive (unspecified/other)	Neisseria meningitidis	A	A	A	5	37	1	81	125	1	1	2	1	3	2	3	ST-5 complex	5	NA	NA	1
30251	H87 000098	UK	1987	invasive (unspecified/other)	Neisseria meningitidis	W	W	W	19	1	1	65	4	2	3	4	3	8	4	6	ST-11 complex	11	4	4	2
30252	H87 002642	UK	1987	invasive (unspecified/other)	Neisseria meningitidis	W	W	W	16	1	1	60	4	11	5	18	8	11	24	21	ST-22 complex	22	4	4	2
30253	K89 000575	UK	1989	invasive (unspecified/other)	Neisseria meningitidis	W	W	W	277	163	1	300	NA	2	3	4	3	8	4	6	ST-11 complex	11	NA	NA	1
30254	L90 001901	UK	1990	invasive (unspecified/other)	Neisseria meningitidis	W	W	W	22	1	1	1	4	2	3	4	3	8	4	6	ST-11 complex	11	4	4	2
30255	L90 002191	UK	1990	invasive (unspecified/other)	Neisseria meningitidis	W	W	W	19	1	1	65	4	2	3	4	3	8	4	6	ST-11 complex	11	4	4	2
30256	L91 000268	UK	1991	invasive (unspecified/other)	Neisseria meningitidis	W	W	W	16	1	1	60	4	11	5	18	8	11	24	9	ST-22 complex	6622	4	4	2
644	L93/4286	UK [England]	1993	invasive (unspecified/other)	Neisseria meningitidis	C	C	C	95	5	1	32	11	2	3	4	3	8	4	6	ST-11 complex	11	11	2	2
49683	LO40030	UK [England]	2014	carrier	Neisseria meningitidis	B	B	B	47	1	57	90	15	12	5	12	35	192	22	17	ST-461 complex	1946	15	4	3
49684	LO40043	UK [England]	2014	carrier	Neisseria meningitidis	NG	B	B	19	1	1	65	4	9	6	9	9	249	6	9	ST-41/44 complex	3346	4	4	2
52634	LO40075	UK [England]	2014	carrier	Neisseria meningitidis	Y	Y	Y	25	1	1	26	4	10	5	18	9	11	9	17	ST-23 complex	23	4	4	2
49686	LO40121	UK [England]	2014	carrier	Neisseria meningitidis	Y	Y	Y	987	NA	1	797	NA	10	5	18	9	11	9	17	ST-23 complex	23	NA	NA	1
49687	LO40132	UK [England]	2014	carrier	Neisseria meningitidis	Y	Y	Y	987	NA	1	797	NA	10	5	18	9	11	9	17	ST-23 complex	23	NA	NA	1
49688	LO40200	UK [England]	2014	carrier	Neisseria meningitidis	W	W	W	22	1	1	1	4	2	3	4	3	8	4	6	ST-11 complex	11	4	4	2
49689	LO40217	UK [England]	2014	carrier	Neisseria meningitidis	Y	Y	Y	25	1	1	26	4	12	5	18	9	11	9	17	ST-23 complex	1655	4	4	2
49690	LO40226	UK [England]	2014	carrier	Neisseria meningitidis	NG	cnl	cnl	14	7	1	142	6	3	6	9	5	9	6	9	ST-41/44 complex	41	6	2	1
49691	LO40256	UK [England]	2014	carrier	Neisseria meningitidis	NG	cnl	cnl	26	1	1	39	4	47	3	58	31	3	59	49		12646	4	4	2
49692	LO40580	UK [England]	2014	carrier	Neisseria meningitidis	NG	E	E	13	3	1	145	3	17	5	19	10	3	26	2	ST-60 complex	1383	3	3	1
49696	LO40728	UK [England]	2015	carrier	Neisseria meningitidis	NG	Y	Y	21	1	1	7	4	6	5	173	13	5	24	17	ST-174 complex	1466	4	4	2
49697	LO40776	UK [England]	2015	carrier	Neisseria meningitidis	NG	cnl	cnl	21	1	1	7	4	16	2	159	92	77	25	112	ST-1117 complex	1117	4	4	2
49699	LO40810	UK [England]	2015	carrier	Neisseria meningitidis	NG	Y	Y	25	1	1	26	4	10	5	18	9	11	9	17	ST-23 complex	23	4	4	2
49700	LO40812	UK [England]	2015	carrier	Neisseria meningitidis	Y	Y	Y	25	1	1	26	4	12	5	18	9	11	9	17	ST-23 complex	1655	4	4	2
49701	LO40814	UK [England]	2015	carrier	Neisseria meningitidis	Y	Y	Y	25	1	1	26	4	10	5	18	9	11	9	17	ST-23 complex	23	4	4	2
49702	LO40829	UK [England]	2015	carrier	Neisseria meningitidis	W	W	W	22	1	1	1	4	2	3	4	3	8	4	6	ST-11 complex	11	4	4	2
49703	LO40999	UK [England]	2015	carrier	Neisseria meningitidis	NG	cnl	cnl	4	1	1	206	4	5	4	17	15	30	7	12	ST-198 complex	823	4	3	1
49704	LO41002	UK [England]	2015	carrier	Neisseria meningitidis	NG	E	E	13	3	1	145	3	17	584	19	17	3	26	2	ST-60 complex	12650	3	3	1
49705	LO41005	UK [England]	2015	carrier	Neisseria meningitidis	NG	cnl	cnl	14	7	1	142	6	3	6	9	5	9	6	9	ST-41/44 complex	41	6	2	1
49706	LO41008	UK [England]	2015	carrier	Neisseria meningitidis	NG	cnl	cnl	21	1	1	7	4	16	2	159	92	77	25	112	ST-1117 complex	1117	4	4	2
49707	LO41012	UK [England]	2015	carrier	Neisseria meningitidis	NG	cnl	cnl	4	1	1	206	4	5	4	17	15	30	7	12	ST-198 complex	823	4	3	1
49708	LO41014	UK [England]	2015	carrier	Neisseria meningitidis	NG	Y	Y	23	1	1	4	4	808	7	6	17	1	18	8	ST-167 complex	12651	4	4	2
49709	LO41015	UK [England]	2015	carrier	Neisseria meningitidis	NG	cnl	cnl	4	1	1	206	4	5	4	17	15	30	7	12	ST-198 complex	823	4	3	1
49710	LO41016	UK [England]	2015	carrier	Neisseria meningitidis	NG	cnl	cnl	21	1	1	7	4	16	2	159	92	77	25	112	ST-1117 complex	1117	4	4	2
49711	LO41021	UK [England]	2015	carrier	Neisseria meningitidis	NG	cnl	cnl	21	1	1	7	4	16	2	159	92	77	25	112	ST-1117 complex	1117	4	4	2
49713	LO41350	UK [England]	2015	carrier	Neisseria meningitidis	NG	cnl	cnl	102	8	10	2	20	16	2	6	25	17	25	22	ST-53 complex	53	20	4	2
49714	LO41426	UK [England]	2015	carrier	Neisseria meningitidis	NG	cnl	cnl	14	7	1	142	6	3	6	9	5	9	6	9	ST-41/44 complex	41	6	2	1
49717	LO41494	UK [England]	2015	carrier	Neisseria meningitidis	NG	cnl	cnl	102	8	10	2	20	16	2	6	25	17	25	22	ST-53 complex	53	20	4	2
49719	LO41652	UK [England]	2015	carrier	Neisseria meningitidis	Y	Y	Y	456	73	80	243	56	12	5	18	9	11	9	17	ST-23 complex	1655	NA	NA	1
29832	M00 240186	UK	2000	invasive (unspecified/other)	Neisseria meningitidis	C	C	C	4	2	1	84	2	2	3	4	3	8	4	6	ST-11 complex	11	2	3	1
29833	M00 240320	UK	2000	invasive (unspecified/other)	Neisseria meningitidis	C	C	C	453	5	1	182	11	2	3	4	3	8	4	6	ST-11 complex	11	11	2	1
29988	M00 240440	UK	2000	invasive (unspecified/other)	Neisseria meningitidis	B	B	B	453	5	1	182	11	2	3	4	3	8	4	6	ST-11 complex	11	11	2	1
29989	M00 240519	UK	2000	invasive (unspecified/other)	Neisseria meningitidis	B	B	B	78	5	1	245	11	2	3	4	3	8	4	101	ST-11 complex	1278	11	2	1
29834	M00 240543	UK	2000	invasive (unspecified/other)	Neisseria meningitidis	C	C	C	4	2	1	84	2	2	3	4	3	8	4	6	ST-11 complex	11	2	3	1
29835	M00 240680	UK	2000	invasive (unspecified/other)	Neisseria meningitidis	C	C	C	1	6	29	100	7	2	3	4	3	8	24	6	ST-11 complex	7128	7	1	1
29990	M00 240815	UK	2000	invasive (unspecified/other)	Neisseria meningitidis	B	B	B	462	5	1	325	11	2	3	4	3	8	4	6	ST-11 complex	11	11	2	1
29836	M00 240845	UK	2000	invasive (unspecified/other)	Neisseria meningitidis	C	C	C	2	5	1	280	11	2	3	4	3	8	4	6	ST-11 complex	11	11	2	1
29837	M00 240963	UK	2000	invasive (unspecified/other)	Neisseria meningitidis	C	C	C	95	5	1	32	11	2	3	4	3	48	4	6	ST-11 complex	214	11	2	2
29838	M00 241306	UK	2000	invasive (unspecified/other)	Neisseria meningitidis	C	C	C	774	5	1	324	11	2	3	4	3	8	4	6	ST-11 complex	11	11	2	1
19957	M00 241317	UK	2000	invasive (unspecified/other)	Neisseria meningitidis	W	W	W	9	1	1	187	47	2	3	NA	NA	NA	NA	NA		NA	NA	NA	1
29928	M00 241341	UK	2000	invasive (unspecified/other)	Neisseria meningitidis	W	W	W	9	1	1	187	47	2	3	4	3	8	4	6	ST-11 complex	11	NA	NA	1
29929	M00 241352	UK	2000	invasive (unspecified/other)	Neisseria meningitidis	W	W	W	9	1	1	187	47	2	3	4	3	8	4	6	ST-11 complex	11	NA	NA	1
29930	M00 241357	UK	2000	invasive (unspecified/other)	Neisseria meningitidis	W	W	W	9	1	1	187	47	2	3	4	3	8	4	6	ST-11 complex	11	NA	NA	1
29931	M00 241362	UK	2000	invasive (unspecified/other)	Neisseria meningitidis	W	W	W	9	1	1	187	47	2	3	4	3	8	4	6	ST-11 complex	11	NA	NA	1
29932	M00 241380	UK	2000	invasive (unspecified/other)	Neisseria meningitidis	W	W	W	9	1	1	187	47	2	3	4	3	8	4	6	ST-11 complex	11	NA	NA	1
29775	M00 241395	UK	2000	invasive (unspecified/other)	Neisseria meningitidis	W	W	W	9	1	1	187	47	2	3	4	3	8	4	6	ST-11 complex	11	NA	NA	1
29933	M00 241396	UK	2000	invasive (unspecified/other)	Neisseria meningitidis	W	W	W	9	1	1	187	47	2	3	4	3	8	4	6	ST-11 complex	11	NA	NA	1
29934	M00 241401	UK	2000	invasive (unspecified/other)	Neisseria meningitidis	W	W	W	9	1	1	187	47	2	3	4	3	8	4	6	ST-11 complex	11	NA	NA	1
29935	M00 241408	UK	2000	invasive (unspecified/other)	Neisseria meningitidis	W	W	W	9	1	1	187	47	2	3	4	3	8	4	6	ST-11 complex	11	NA	NA	1
29936	M00 241420	UK	2000	invasive (unspecified/other)	Neisseria meningitidis	W	W	W	9	1	1	187	47	2	3	4	3	8	4	6	ST-11 complex	11	NA	NA	1
29937	M00 241430	UK	2000	invasive (unspecified/other)	Neisseria meningitidis	W	W	W	9	1	1	187	47	2	3	4	3	8	4	6	ST-11 complex	11	NA	NA	1
29938	M00 241431	UK	2000	invasive (unspecified/other)	Neisseria meningitidis	W	W	W	9	1	1	187	47	2	3	4	3	8	4	6	ST-11 complex	11	NA	NA	1
29939	M00 241432	UK	2000	invasive (unspecified/other)	Neisseria meningitidis	W	W	W	9	1	1	187	47	2	3	4	3	8	4	6	ST-11 complex	11	NA	NA	1
29940	M00 241433	UK	2000	invasive (unspecified/other)	Neisseria meningitidis	W	W	W	9	1	1	187	47	2	3	4	3	8	4	6	ST-11 complex	11	NA	NA	1
30122	M00 241434	UK	2000	invasive (unspecified/other)	Neisseria meningitidis	W	W	W	9	1	1	187	47	2	3	4	3	8	4	6	ST-11 complex	11	NA	NA	1
29941	M00 241435	UK	2000	invasive (unspecified/other)	Neisseria meningitidis	W	W	W	9	1	1	187	47	2	3	4	3	8	4	6	ST-11 complex	11	NA	NA	1
29942	M00 241445	UK	2000	invasive (unspecified/other)	Neisseria meningitidis	W	W	W	9	1	1	187	47	2	3	4	3	8	4	6	ST-11 complex	11	NA	NA	1
29943	M00 241447	UK	2000	invasive (unspecified/other)	Neisseria meningitidis	W	W	W	9	1	1	187	47	2	3	4	3	8	4	6	ST-11 complex	11	NA	NA	1
29945	M00 241510	UK	2000	invasive (unspecified/other)	Neisseria meningitidis	W	W	W	9	1	1	187	47	2	3	4	3	8	4	6	ST-11 complex	11	NA	NA	1
29946	M00 241511	UK	2000	invasive (unspecified/other)	Neisseria meningitidis	W	W	W	9	1	1	187	47	2	3	4	3	8	4	6	ST-11 complex	11	NA	NA	1
29947	M00 241557	UK	2000	invasive (unspecified/other)	Neisseria meningitidis	W	W	W	9	1	1	187	47	2	3	4	3	8	4	6	ST-11 complex	11	NA	NA	1
29948	M00 241578	UK	2000	invasive (unspecified/other)	Neisseria meningitidis	W	W	W	9	1	1	187	47	2	3	4	3	8	4	6	ST-11 complex	11	NA	NA	1
29949	M00 241620	UK	2000	invasive (unspecified/other)	Neisseria meningitidis	W	W	W	9	1	1	187	47	2	3	4	3	8	4	6	ST-11 complex	11	NA	NA	1
29950	M00 241645	UK	2000	invasive (unspecified/other)	Neisseria meningitidis	W	W	W	9	1	1	187	47	2	3	4	3	8	4	6	ST-11 complex	11	NA	NA	1
29951	M00 241655	UK	2000	invasive (unspecified/other)	Neisseria meningitidis	W	W	W	9	1	1	187	47	2	3	4	3	8	4	6	ST-11 complex	11	NA	NA	1
29953	M00 241674	UK	2000	invasive (unspecified/other)	Neisseria meningitidis	W	W	W	9	1	1	187	47	2	3	4	3	8	4	6	ST-11 complex	11	NA	NA	1
29954	M00 241675	UK	2000	invasive (unspecified/other)	Neisseria meningitidis	W	W	W	9	1	1	187	47	2	3	4	3	8	4	6	ST-11 complex	11	NA	NA	1
29955	M00 241704	UK	2000	invasive (unspecified/other)	Neisseria meningitidis	W	W	W	9	1	1	187	47	2	3	4	3	8	4	6	ST-11 complex	11	NA	NA	1
29956	M00 241759	UK	2000	invasive (unspecified/other)	Neisseria meningitidis	W	W	W	9	1	1	187	47	2	3	4	3	8	4	6	ST-11 complex	11	NA	NA	1
29957	M00 241760	UK	2000	invasive (unspecified/other)	Neisseria meningitidis	W	W	W	9	1	1	187	47	2	3	4	3	8	4	6	ST-11 complex	11	NA	NA	1
29840	M00 241761	UK	2000	invasive (unspecified/other)	Neisseria meningitidis	C	C	C	22	1	1	1	4	2	3	4	3	8	4	6	ST-11 complex	11	4	4	2
29958	M00 241800	UK	2000	invasive (unspecified/other)	Neisseria meningitidis	W	W	W	9	1	1	187	47	2	3	4	3	8	4	6	ST-11 complex	11	NA	NA	1
29959	M00 241833	UK	2000	invasive (unspecified/other)	Neisseria meningitidis	W	W	W	9	1	1	187	47	2	3	4	3	8	4	6	ST-11 complex	11	NA	NA	1
29960	M00 241894	UK	2000	invasive (unspecified/other)	Neisseria meningitidis	W	W	W	9	1	1	187	47	2	3	4	3	8	4	6	ST-11 complex	11	NA	NA	1
29961	M00 241957	UK	2000	invasive (unspecified/other)	Neisseria meningitidis	W	W	W	9	1	1	187	47	2	3	4	3	8	4	6	ST-11 complex	11	NA	NA	1
29962	M00 241978	UK	2000	invasive (unspecified/other)	Neisseria meningitidis	W	W	W	9	1	1	187	47	2	3	4	3	8	4	6	ST-11 complex	11	NA	NA	1
30123	M00 241979	UK	2000	invasive (unspecified/other)	Neisseria meningitidis	W	W	W	9	1	1	187	47	2	3	4	3	8	4	6	ST-11 complex	11	NA	NA	1
29963	M00 241986	UK	2000	invasive (unspecified/other)	Neisseria meningitidis	W	W	W	9	1	1	187	47	2	3	4	3	8	4	6	ST-11 complex	11	NA	NA	1
29964	M00 242029	UK	2000	invasive (unspecified/other)	Neisseria meningitidis	W	W	W	9	1	1	187	47	2	3	4	3	8	4	6	ST-11 complex	11	NA	NA	1
29841	M00 242236	UK	2000	invasive (unspecified/other)	Neisseria meningitidis	C	C	C	157	166	30	353	204	2	3	4	3	8	4	6	ST-11 complex	11	NA	NA	1
29965	M00 242295	UK	2000	invasive (unspecified/other)	Neisseria meningitidis	W	W	W	9	1	1	187	47	2	3	4	3	8	4	6	ST-11 complex	11	NA	NA	1
29966	M00 242531	UK	2000	invasive (unspecified/other)	Neisseria meningitidis	W	W	W	9	1	1	187	47	2	3	4	3	8	4	6	ST-11 complex	11	NA	NA	1
29967	M00 242667	UK	2000	invasive (unspecified/other)	Neisseria meningitidis	W	W	W	9	1	1	187	47	2	3	4	3	8	4	6	ST-11 complex	11	NA	NA	1
29968	M00 242770	UK	2000	invasive (unspecified/other)	Neisseria meningitidis	W	W	W	9	1	1	187	47	2	3	4	3	8	4	6	ST-11 complex	11	NA	NA	1
29969	M00 242916	UK	2000	invasive (unspecified/other)	Neisseria meningitidis	W	W	W	9	1	1	187	47	2	3	4	3	8	4	6	ST-11 complex	11	NA	NA	1
30277	M00 242922	UK	2000	invasive (unspecified/other)	Neisseria meningitidis	B	B	B	4	2	1	84	2	3	6	9	5	9	6	9	ST-41/44 complex	41	2	3	1
29970	M00 242974	UK	2000	invasive (unspecified/other)	Neisseria meningitidis	W	W	W	9	1	NA	187	47	2	3	4	3	8	4	6	ST-11 complex	11	NA	NA	1
29843	M00 243016	UK	2000	invasive (unspecified/other)	Neisseria meningitidis	C	C	C	4	2	1	84	2	2	3	4	3	8	4	6	ST-11 complex	11	2	3	1
29991	M00 243175	UK	2000	invasive (unspecified/other)	Neisseria meningitidis	W	W	W	9	1	1	187	47	2	3	4	3	8	4	6	ST-11 complex	11	NA	NA	1
34541	M00 243289	UK	2000	invasive (unspecified/other)	Neisseria meningitidis	C	C	C	NA	5	1	917	11	2	3	4	3	8	4	6	ST-11 complex	11	11	2	NA
29992	M00 243297	UK	2000	invasive (unspecified/other)	Neisseria meningitidis	W	W	W	9	1	1	187	47	2	3	4	3	8	4	6	ST-11 complex	11	NA	NA	1
38130	M01 240007	UK	2001	invasive (unspecified/other)	Neisseria meningitidis	B	B	B	15	4	2	193	1	4	10	15	9	8	11	9	ST-269 complex	269	1	1	1
19364	M01-240013	UK	2001	invasive (unspecified/other)	Neisseria meningitidis		B	B	19	1	1	83	4	4	10	2	5	38	11	1		1159	4	4	2
29993	M01 240025	UK	2001	invasive (unspecified/other)	Neisseria meningitidis	B	B	B	118	5	1	73	11	2	3	4	3	8	4	6	ST-11 complex	11	11	2	2
29994	M01 240028	UK	2001	invasive (unspecified/other)	Neisseria meningitidis	W	W	W	9	1	1	187	47	2	3	4	3	8	4	6	ST-11 complex	11	NA	NA	1
34543	M01 240070	UK	2001	invasive (unspecified/other)	Neisseria meningitidis	W	W	W	16	1	1	60	4	11	5	18	8	11	4	21	ST-22 complex	184	4	4	2

35829	M01 240100	UK	2001	invasive (unspecified/other)	Neisseria meningitidis	B	B	B	15	4	1	193	1	4	10	15	9	5	11	17	ST-269 complex	1791	1	1	1
30273	M01 240101	UK	2001	invasive (unspecified/other)	Neisseria meningitidis	B	B	B	15	4	2	193	1	4	10	15	17	8	11	9	ST-269 complex	1049	1	1	1
29995	M01 240168	UK	2001	invasive (unspecified/other)	Neisseria meningitidis	W	W	W	9	1	1	187	47	2	3	4	3	8	4	6	ST-11 complex	11	NA	NA	1
30274	M01 240185	UK	2001	invasive (unspecified/other)	Neisseria meningitidis	B	B	B	10	5	1	199	11	2	3	4	3	8	4	6	ST-11 complex	11	11	2	1
29996	M01 240240	UK	2001	invasive (unspecified/other)	Neisseria meningitidis	W	W	W	9	1	1	187	47	2	3	4	3	8	4	6	ST-11 complex	11	NA	NA	1
29997	M01 240241	UK	2001	invasive (unspecified/other)	Neisseria meningitidis	W	W	W	9	1	1	187	47	2	3	4	3	8	4	6	ST-11 complex	11	NA	NA	1
29999	M01 240354	UK	2001	invasive (unspecified/other)	Neisseria meningitidis	W	W	W	9	1	1	187	47	2	3	4	3	8	4	6	ST-11 complex	11	NA	NA	1
30276	M01 240364	UK	2001	invasive (unspecified/other)	Neisseria meningitidis	B	B	B	31	9	4	58	16	2	3	4	3	8	4	6	ST-11 complex	11	16	2	3
29846	M01 240426	UK	2001	invasive (unspecified/other)	Neisseria meningitidis	C	C	C	303	5	1	184	11	672	3	4	3	8	4	6	ST-11 complex	11149	11	2	1
29850	M01 240514	UK	2001	invasive (unspecified/other)	Neisseria meningitidis	C	C	C	4	2	1	84	2	2	10	4	3	8	4	6	ST-11 complex	5069	2	3	1
29847	M01 240526	UK	2001	invasive (unspecified/other)	Neisseria meningitidis	C	C	C	259	5	1	215	11	2	5	4	3	8	4	6	ST-11 complex	3463	11	2	1
38131	M01 240601	UK	2001	invasive (unspecified/other)	Neisseria meningitidis	B	B	B	15	4	1	193	1	4	10	15	9	5	11	17	ST-269 complex	1791	1	1	1
30000	M01 240622	UK	2001	invasive (unspecified/other)	Neisseria meningitidis	B	B	B	462	5	1	325	11	2	3	4	3	8	4	6	ST-11 complex	11	11	2	1
30001	M01 240634	UK	2001	invasive (unspecified/other)	Neisseria meningitidis	W	W	W	9	1	1	187	47	2	3	4	3	8	4	6	ST-11 complex	11	NA	NA	1
30002	M01 240659	UK	2001	invasive (unspecified/other)	Neisseria meningitidis	W	W	W	9	1	1	187	47	2	3	4	3	8	4	6	ST-11 complex	11	NA	NA	1
30003	M01 240663	UK	2001	invasive (unspecified/other)	Neisseria meningitidis	B	B	B	10	5	1	199	11	2	3	4	3	8	4	6	ST-11 complex	11	11	2	1
30004	M01 240759	UK	2001	invasive (unspecified/other)	Neisseria meningitidis	W	W	W	9	1	1	187	47	2	3	4	3	8	4	6	ST-11 complex	11	NA	NA	1
30005	M01 240760	UK	2001	invasive (unspecified/other)	Neisseria meningitidis	W	W	W	9	1	1	187	47	2	3	4	3	8	4	6	ST-11 complex	11	NA	NA	1
30006	M01 240801	UK	2001	invasive (unspecified/other)	Neisseria meningitidis	W	W	W	9	1	1	187	47	2	3	4	3	8	4	6	ST-11 complex	11	NA	NA	1
30007	M01 240807	UK	2001	invasive (unspecified/other)	Neisseria meningitidis	W	W	W	9	1	1	187	47	2	3	4	3	8	4	6	ST-11 complex	11	NA	NA	1
30008	M01 240808	UK	2001	invasive (unspecified/other)	Neisseria meningitidis	W	W	W	9	1	1	187	47	2	3	4	3	8	4	6	ST-11 complex	11	NA	NA	1
30009	M01 240839	UK	2001	invasive (unspecified/other)	Neisseria meningitidis	W	W	W	9	1	1	187	47	2	3	4	3	8	4	6	ST-11 complex	11	NA	NA	1
30010	M01 240840	UK	2001	invasive (unspecified/other)	Neisseria meningitidis	W	W	W	9	1	1	187	47	2	3	4	3	8	4	6	ST-11 complex	11	NA	NA	1
29851	M01 240850	UK	2001	invasive (unspecified/other)	Neisseria meningitidis	C	C	C	10	5	1	199	11	2	3	4	3	8	4	6	ST-11 complex	11	11	2	1
30011	M01 240859	UK	2001	invasive (unspecified/other)	Neisseria meningitidis	W	W	W	9	1	1	187	47	2	3	4	3	8	4	6	ST-11 complex	11	NA	NA	1
30012	M01 240887	UK	2001	invasive (unspecified/other)	Neisseria meningitidis	W	W	W	9	1	1	187	47	2	3	4	3	8	4	6	ST-11 complex	11	NA	NA	1
30013	M01 240900	UK	2001	invasive (unspecified/other)	Neisseria meningitidis	W		W	9	1	1	187	47	2	3	4	3	8	4	6	ST-11 complex	11	NA	NA	1
30014	M01 240902	UK	2001	invasive (unspecified/other)	Neisseria meningitidis	W	W	W	9	1	1	187	47	2	3	4	3	8	4	6	ST-11 complex	11	NA	NA	1
30015	M01 240913	UK	2001	invasive (unspecified/other)	Neisseria meningitidis	W	W	W	9	1	1	187	47	2	3	4	3	8	4	6	ST-11 complex	11	NA	NA	1
30016	M01 240915	UK	2001	invasive (unspecified/other)	Neisseria meningitidis	W	W	W	9	1	1	187	47	2	3	4	3	8	4	6	ST-11 complex	11	NA	NA	1
30017	M01 240916	UK	2001	invasive (unspecified/other)	Neisseria meningitidis	W	W	W	9	1	1	187	47	2	3	4	3	8	4	6	ST-11 complex	11	NA	NA	1
30018	M01 240921	UK	2001	invasive (unspecified/other)	Neisseria meningitidis	W	W	W	9	1	1	187	47	2	3	4	3	8	4	6	ST-11 complex	11	NA	NA	1
30019	M01 240949	UK	2001	invasive (unspecified/other)	Neisseria meningitidis	W	W	W	9	1	1	187	47	2	3	4	3	8	4	6	ST-11 complex	11	NA	NA	1
30020	M01 240953	UK	2001	invasive (unspecified/other)	Neisseria meningitidis	W	W	W	9	1	1	187	47	2	3	4	3	8	4	6	ST-11 complex	11	NA	NA	1
30021	M01 240956	UK	2001	invasive (unspecified/other)	Neisseria meningitidis	W	W	W	9	1	1	187	47	2	3	4	3	8	4	6	ST-11 complex	11	NA	NA	1
30022	M01 240959	UK	2001	invasive (unspecified/other)	Neisseria meningitidis	W	W	W	9	1	1	187	47	2	3	4	3	8	4	6	ST-11 complex	11	NA	NA	1
30023	M01 240967	UK	2001	invasive (unspecified/other)	Neisseria meningitidis	W	W	W	9	1	1	187	47	2	3	4	3	8	4	6	ST-11 complex	11	NA	NA	1
30024	M01 240978	UK	2001	invasive (unspecified/other)	Neisseria meningitidis	W	W	W	9	1	1	187	47	2	3	4	3	8	4	6	ST-11 complex	11	NA	NA	1
30025	M01 240979	UK	2001	invasive (unspecified/other)	Neisseria meningitidis	W	W	W	9	1	1	187	47	2	3	4	3	8	4	6	ST-11 complex	11	NA	NA	1
30026	M01 240989	UK	2001	invasive (unspecified/other)	Neisseria meningitidis	W	W	W	9	1	1	187	47	2	3	4	3	8	4	6	ST-11 complex	11	NA	NA	1
30027	M01 240990	UK	2001	invasive (unspecified/other)	Neisseria meningitidis	W	W	W	9	1	1	187	47	2	3	4	3	8	4	6	ST-11 complex	11	NA	NA	1
30028	M01 241031	UK	2001	invasive (unspecified/other)	Neisseria meningitidis	W	W	W	9	1	1	187	47	2	3	4	3	8	4	6	ST-11 complex	11	NA	NA	1
30029	M01 241052	UK	2001	invasive (unspecified/other)	Neisseria meningitidis	W	W	W	9	1	1	187	47	2	3	4	3	8	4	6	ST-11 complex	11	NA	NA	1
30030	M01 241064	UK	2001	invasive (unspecified/other)	Neisseria meningitidis	W	W	W	9	1	1	187	47	2	3	4	3	8	4	6	ST-11 complex	11	NA	NA	1
30031	M01 241093	UK	2001	invasive (unspecified/other)	Neisseria meningitidis	W	W	W	9	1	1	187	47	2	3	4	3	8	4	6	ST-11 complex	11	NA	NA	1
30032	M01 241097	UK	2001	invasive (unspecified/other)	Neisseria meningitidis	W	W	W	9	1	1	187	47	2	3	4	3	8	4	6	ST-11 complex	11	NA	NA	1
30033	M01 241098	UK	2001	invasive (unspecified/other)	Neisseria meningitidis	W	W	W	9	1	1	187	47	2	3	4	3	8	4	6	ST-11 complex	11	NA	NA	1
30034	M01 241133	UK	2001	invasive (unspecified/other)	Neisseria meningitidis	W	W	W	9	1	1	187	47	2	3	4	3	8	4	6	ST-11 complex	11	NA	NA	1
30035	M01 241213	UK	2001	invasive (unspecified/other)	Neisseria meningitidis	W	W	W	9	1	1	187	47	2	3	4	3	8	4	6	ST-11 complex	11	NA	NA	1
30036	M01 241214	UK	2001	invasive (unspecified/other)	Neisseria meningitidis	W	W	W	9	1	1	187	47	2	3	4	3	8	4	6	ST-11 complex	11	NA	NA	1
38132	M01 241271	UK	2001	invasive (unspecified/other)	Neisseria meningitidis	B	B	B	4	NA	1	84	NA	3	6	9	5	9	6	9	ST-41/44 complex	41	NA	NA	1
29853	M01 241276	UK	2001	invasive (unspecified/other)	Neisseria meningitidis	C	C	C	95	5	1	40	11	2	3	4	3	8	4	6	ST-11 complex	11	11	2	2
38133	M01 241299	UK	2001	invasive (unspecified/other)	Neisseria meningitidis	B	B	B	19	1	1	65	4	9	6	9	9	58	6	16	ST-41/44 complex	571	4	4	2
30037	M01 241368	UK	2001	invasive (unspecified/other)	Neisseria meningitidis	W	W	W	9	1	1	187	47	2	3	4	3	8	4	6	ST-11 complex	11	NA	NA	1
30038	M01 241396	UK	2001	invasive (unspecified/other)	Neisseria meningitidis	W	W	W	9	1	1	187	47	2	3	4	3	8	4	6	ST-11 complex	11	NA	NA	1
38134	M01 241451	UK	2001	invasive (unspecified/other)	Neisseria meningitidis	B	B	B	19	1	1	65	4	12	6	9	9	9	6	9	ST-41/44 complex	43	4	4	2
30039	M01 241454	UK	2001	invasive (unspecified/other)	Neisseria meningitidis	W	W	W	9	1	1	187	47	2	3	4	3	8	4	6	ST-11 complex	11	NA	NA	1
30040	M01 241494	UK	2001	invasive (unspecified/other)	Neisseria meningitidis	W	W	W	9	1	1	187	47	2	3	4	3	8	4	6	ST-11 complex	11	NA	NA	1
38135	M01 241601	UK	2001	invasive (unspecified/other)	Neisseria meningitidis	B	B	B	14	7	1	142	6	10	6	9	5	9	6	9	ST-41/44 complex	42	6	2	1
29855	M01 241612	UK	2001	invasive (unspecified/other)	Neisseria meningitidis	C	C	C	10	5	1	199	11	2	3	4	3	8	4	6	ST-11 complex	11	11	2	1
29856	M01 241620	UK	2001	invasive (unspecified/other)	Neisseria meningitidis	C	C	C	259	5	1	215	11	2	3	4	3	8	4	6	ST-11 complex	11	11	2	1
30041	M01 241950	UK	2001	invasive (unspecified/other)	Neisseria meningitidis	W	W	W	9	1	1	187	47	2	3	4	3	8	4	6	ST-11 complex	11	NA	NA	1
30042	M01 242003	UK	2001	carrier	Neisseria meningitidis	B	B	B	NA	5	1	917	11	2	3	4	3	8	4	6	ST-11 complex	11	11	2	NA
30043	M01 242084	UK	2001	invasive (unspecified/other)	Neisseria meningitidis	W	W	W	9	1	1	187	47	2	3	4	3	8	4	6	ST-11 complex	11	NA	NA	1
30265	M01 242103	UK	2001	invasive (unspecified/other)	Neisseria meningitidis	W	W	W	9	1	1	187	47	2	3	4	3	8	4	6	ST-11 complex	11	NA	NA	1
29857	M01 242315	UK	2001	invasive (unspecified/other)	Neisseria meningitidis	C	C	C	10	5	1	199	11	2	3	4	3	8	4	6	ST-11 complex	11	11	2	1
30266	M01 242502	UK	2001	invasive (unspecified/other)	Neisseria meningitidis	W	W	W	9	1	1	187	47	2	3	4	3	8	4	6	ST-11 complex	11	NA	NA	1
30267	M01 242503	UK	2001	invasive (unspecified/other)	Neisseria meningitidis	W	W	W	9	1	1	187	47	2	3	4	3	8	4	6	ST-11 complex	11	NA	NA	1
30044	M01 242511	UK	2001	invasive (unspecified/other)	Neisseria meningitidis	B	B	B	259	5	1	215	11	150	3	4	3	8	4	6	ST-11 complex	1789	11	2	1
30045	M01 242572	UK	2001	invasive (unspecified/other)	Neisseria meningitidis	B	B	B	4	2	6	84	2	3	6	9	5	9	6	9	ST-41/44 complex	41	2	3	1
29680	M01 242591	UK	2001	invasive (unspecified/other)	Neisseria meningitidis	W	W	W	9	1	1	187	47	2	3	4	3	8	4	6	ST-11 complex	11	NA	NA	1
29677	M01 242608	UK	2001	invasive (unspecified/other)	Neisseria meningitidis	W	W	W	9	1	1	187	47	2	3	4	3	8	4	6	ST-11 complex	11	NA	NA	1
29679	M01 242679	UK	2001	invasive (unspecified/other)	Neisseria meningitidis	W	W	W	22	1	1	1	4	2	3	4	3	8	4	6	ST-11 complex	11	4	4	2
29681	M01 242700	UK	2001	invasive (unspecified/other)	Neisseria meningitidis	W	W	W	9	1	1	187	47	2	3	4	3	8	4	6	ST-11 complex	11	NA	NA	1
29684	M01 242717	UK	2001	invasive (unspecified/other)	Neisseria meningitidis	W	W	W	9	1	1	187	47	2	3	4	3	8	4	6	ST-11 complex	11	NA	NA	1
29859	M01 242752	UK	2001	invasive (unspecified/other)	Neisseria meningitidis	C	C	C	118	5	1	73	11	2	3	4	3	8	4	6	ST-11 complex	11	11	2	2
29890	M10 240001	UK	2010	invasive (unspecified/other)	Neisseria meningitidis	C	C	C	306	5	1	217	11	2	3	4	3	8	4	6	ST-11 complex	11	11	2	1
29714	M10 240005	UK	2010	invasive (unspecified/other)	Neisseria meningitidis	W	W	W	22	1	1	1	4	2	3	4	3	8	4	6	ST-11 complex	11	4	4	2
29892	M10 240014	UK	2010	invasive (unspecified/other)	Neisseria meningitidis	C	C	C	1046	5	1	358	11	2	3	4	3	8	4	6	ST-11 complex	11	11	2	1
20809	M10 240015	UK	2010	invasive (unspecified/other)	Neisseria meningitidis		B	B	4	2	1	84	2	8	6	9	5	9	6	9	ST-41/44 complex	1788	2	3	1
20781	M10 240050	UK	2010	invasive (unspecified/other)	Neisseria meningitidis		B	B	4	2	1	84	2	3	6	9	5	9	6	9	ST-41/44 complex	41	2	3	1
20782	M10 240052	UK	2010	invasive (unspecified/other)	Neisseria meningitidis		B	B	4	2	1	84	2	3	6	9	5	11	6	9	ST-41/44 complex	154	2	3	1
20785	M10 240080	UK	2010	invasive (unspecified/other)	Neisseria meningitidis		B	B	4	2	1	84	2	10	6	9	499	526	6	9	ST-41/44 complex	8229	2	3	1
20788	M10 240115	UK	2010	invasive (unspecified/other)	Neisseria meningitidis		B	B	14	30	1	142	71	3	6	9	5	9	6	9	ST-41/44 complex	41	NA	NA	1
20792	M10 240137	UK	2010	invasive (unspecified/other)	Neisseria meningitidis		B	B	4	2	4	84	2	3	5	9	5	9	6	9	ST-41/44 complex	3754	2	3	1
20795	M10 240168	UK	2010	invasive (unspecified/other)	Neisseria meningitidis		B	B	35	3	1	167	3	3	6	9	9	9	6	9	ST-41/44 complex	303	3	3	1
20798	M10 240184	UK	2010	invasive (unspecified/other)	Neisseria meningitidis		B	B	4	2	1	84	2	3	6	9	5	11	6	9	ST-41/44 complex	154	2	3	1
20800	M10 240219	UK	2010	invasive (unspecified/other)	Neisseria meningitidis		B	B	14	30	1	142	71	3	6	9	5	111	6	9	ST-41/44 complex	7385	NA	NA	1
29891	M10 240229	UK	2010	invasive (unspecified/other)	Neisseria meningitidis	C	C	C	22	1	1	1	4	2	3	4	3	8	4	6	ST-11 complex	11	4	4	2
20802	M10 240300	UK	2010	invasive (unspecified/other)	Neisseria meningitidis		B	B	14	5	1	204	11	3	6	19	5	3	6	9	ST-41/44 complex	340	11	2	1
29718	M10 240389	UK	2010	invasive (unspecified/other)	Neisseria meningitidis	W	W	W	22	1	1	1	4	2	3	4	3	8	4	6	ST-11 complex	11	4	4	2
29719	M10 240452	UK	2010	invasive (unspecified/other)	Neisseria meningitidis	W	W	W	22	1	1	1	4	2	3	4	3	8	4	6	ST-11 complex	11	4	4	2
19023	M10 240473	UK [England]	2010	invasive (unspecified/other)	Neisseria meningitidis	B	B	B	13	3	57	145	3	4	10	34	5	38	11	9	ST-269 complex	1161	3	3	1
18968	M10 240474	UK [England]	2010	invasive (unspecified/other)	Neisseria meningitidis	B	B	B	15	4	2	193	1	4	10	15	9	8	11	9	ST-269 complex	269	1	1	1
19958	M10 240476	UK [England]	2010	invasive (unspecified/other)	Neisseria meningitidis	B	B	B	110	32	3	171	5	7	5	18	13	36	53	15	ST-213 complex	9812	NA	NA	1
18969	M10 240478	UK [England]	2010	invasive (unspecified/other)	Neisseria meningitidis	B	B	B	4	2	1	84	2	3	6	34	5	11	6	9	ST-41/44 complex	1194	2	3	1
19024	M10 240480	UK [England]	2010	invasive (unspecified/other)	Neisseria meningitidis	B	B	B	4	2	1	84	2	3	6	34	5	11	6	9	ST-41/44 complex	1194	2	3	1
19025	M10 240481	UK [England]	2010	invasive (unspecified/other)	Neisseria meningitidis	B	B	B	19	1	1	83	4	4	4	2	5	38	11	9		1831	4	4	2
19026	M10 240482	UK [England]	2010	invasive (unspecified/other)	Neisseria meningitidis	B	B	B	4	2	4	84	2	3	5	9	5	9	6	9	ST-41/44 complex	3754	2	3	1
19027	M10 240484	UK [England]	2010	invasive (unspecified/other)	Neisseria meningitidis	B	B	B	19	1	1	65	4	9	20	9	9	9	6	2	ST-41/44 complex	1097	4	4	2
19028	M10 240485	UK [England]	2010	invasive (unspecified/other)	Neisseria meningitidis	B	B	B	13	3	57	145	3	4	10	2	5	38	11	9	ST-269 complex	275	3	3	1
19029	M10 240487	UK [England]	2010	invasive (unspecified/other)	Neisseria meningitidis	B	B	B	15	4	2	193	1	4	10	15	17	8	11	9	ST-269 complex	1049	1	1	1
19030	M10 240489	UK [England]	2010	invasive (unspecified/other)	Neisseria meningitidis	B	B	B	15	4	57	193	1	12	5	12	35	60	22	17	ST-461 complex	461	1	1	1
19031	M10 240490	UK [England]	2010	invasive (unspecified/other)	Neisseria meningitidis	B	B	B	13	3	57	145	3	4	10	34	5	38	11	9	ST-269 complex	1161	3	3	1
19032	M10 240498	UK [Wales]	2010	invasive (unspecified/other)	Neisseria meningitidis	B	B	B	4	2	79	84	2	3	6	9	5	9	6	9	ST-41/44 complex	41	2	3	1
19959	M10 240499	UK [England]	2010	invasive (unspecified/other)	Neisseria meningitidis	B	B	B	19	1	1	83	4	4	10	2	5	38	11	9	ST-269 complex	275	4	4	2
19960	M10 240500	UK [England]	2010	invasive (unspecified/other)	Neisseria meningitidis	B	B	B	14	7	1	142	6	3	6	9	NA	9	6	9		NA	6	2	1
19961	M10 240502	UK [England]	2010	invasive (unspecified/other)	Neisseria meningitidis	B	B	B	14	6	3	202	7	3	6	19	5	3	6	9	ST-41/44 complex	340	7	1	1
19962	M10 240503	UK [England]	2010	invasive (unspecified/other)	Neisseria meningitidis	Y	Y	Y	25	1	1	26	4	10	5	18	9	11	9	17	ST-23 complex	23	4	4	2
19963	M10 240505	UK [England]	2010	invasive (unspecified/other)	Neisseria meningitidis	Y	Y	Y	25	1	1	26	4	12	5	18	9	11	9	17	ST-23 complex	1655	4	4	2
19964	M10 240507	UK [England]	2010	invasive (unspecified/other)	Neisseria meningitidis	Y	Y	Y	104	1	57	10	4	10	5	18	59	11	9	17	ST-23 complex	183	4	4	2
19965	M10 240508	UK [England]	2010	invasive (unspecified/other)	Neisseria meningitidis	B	B	B	291	20	4	203	8	4	10	47	17	6	2	9		1575	NA	NA	1
19966	M10 240511	UK [England]	2010	invasive (unspecified/other)	Neisseria meningitidis	B	B	B	13	3	57	145	3	4	10	34	5	38	11	330		4713	3	3	1
19967	M10 240512	UK [England]	2010	invasive (unspecified/other)	Neisseria meningitidis	B	B	B	4	2	1	84	2	3	6	9	5	9	6	9	ST-41/44 complex	41	2	3	1
19968	M10 240514	UK [England]	2010	invasive (unspecified/other)	Neisseria meningitidis	W	W	W	22	1	1	1	4	2	3	4	3	8	4	6	ST-11 complex	11	4	4	2
19969	M10 240515	UK [England]	2010	invasive (unspecified/other)	Neisseria meningitidis	B	B	B	15	4	NA	193	1	4	10	15	9	8	11	9	ST-269 complex	269	1	1	1
19970	M10 240520	UK [England]	2010	invasive (unspecified/other)	Neisseria meningitidis	Y	Y	Y	25	1	1	26	4	12	5	18	9	11	9	17	ST-23 complex	1655	4	4	2
19971	M10 240521	UK [England]	2010	invasive (unspecified/other)	Neisseria meningitidis	B	B	B	595	59	5	86	9	3	6	18	5	3	6	9	ST-41/44 complex	3802	NA	NA	3
19972	M10 240522	UK [England]	2010	invasive (unspecified/other)	Neisseria meningitidis	B	B	B	4	2	1	84	2	3	6	9	5	9	6	9	ST-41/44 complex	41	2	3	1
19973	M10 240525	UK [England]	2010	invasive (unspecified/other)	Neisseria meningitidis	B	B	B	1	6	3	100	7	4	5	5	4	6	3	8	ST-32 complex	2931	7	1	1
19974	M10 240527	UK [England]	2010	invasive (unspecified/other)	Neisseria meningitidis	B	B	B	15	4	2	193	1	4	10	15	9	8	11	9	ST-269 complex	269	1	1	1
19975	M10 240528	UK [England]	2010	invasive (unspecified/other)	Neisseria meningitidis	B	B	B	13	3	57	145	3	4	10	15	5	38	11	9	ST-269 complex	7833	3	3	1
19976	M10 240530	UK [England]	2010	invasive (unspecified/other)	Neisseria meningitidis	Y	Y	Y	104	1	57	10	4	10	5	216	17	11	9	17	ST-23 complex	9813	4	4	2
19977	M10 240531	UK [England]	2010	invasive (unspecified/other)	Neisseria meningitidis	Y	Y	Y	25	1	1	26	4	12	5	18	9	11	9	17	ST-23 complex	1655	4	4	2
19978	M10 240532	UK [England]	2010	invasive (unspecified/other)	Neisseria meningitidis	B	B	B	4	2	1	84	2	3	6	9	5	9	6	9	ST-41/44 complex	41	2	3	1
19979	M10 240534	UK [England]	2010	invasive (unspecified/other)	Neisseria meningitidis	Y	Y	Y	25	1	1	26	4	12	5	18	9	11	9	17	ST-23 complex	1655	4	4	2
19980	M10 240536	UK [England]	2010	invasive (unspecified/other)	Neisseria meningitidis	Y	Y	Y	25	1	1	26	4	12	5	18	9	11	9	17	ST-23 complex	1655	4	4	2
19981	M10 240540	UK [England]	2010	invasive (unspecified/other)	Neisseria meningitidis	Y	Y	Y	25	1	1	26	4	345	5	18	9	11	9	17	ST-23 complex	9814	4	4	2
19982	M10 240546	UK [England]	2010	invasive (unspecified/other)	Neisseria meningitidis	B	B	B	45	11	77	87	10	7	5	1	NA	36	53	15		NA	NA	NA	3
19983	M10 240547	UK [England]	2010	invasive (unspecified/other)	Neisseria meningitidis	B	B	B	596	6	3	34	7	8	10	77	4	6	3	8	ST-32 complex	749	7	1	3
19984	M10 240548	UK [England]	2010	invasive (unspecified/other)	Neisseria meningitidis	B	B	B	14	5	1	204	11	3	6	19	5	3	6	9	ST-41/44 complex	340	11	2	1
19985	M10 240549	UK [Wales]	2010	invasive (unspecified/other)	Neisseria meningitidis	B	B	B	4	2	1	84	2	3	6	9	5	9	6	9	ST-41/44 complex	41	2	3	1
19986	M10 240550	UK [England]	2010	invasive (unspecified/other)	Neisseria meningitidis	B	B	B	597	60	14	88	12	4	10	2	5	3	11	9	ST-269 complex	1163	NA	NA	2
19987	M10 240553	UK [Wales]	2010	invasive (unspecified/other)	Neisseria meningitidis	B	B	B	13	3	57	145	3	4	10	34	5	38	11	9	ST-269 complex	1161	3	3	1
19988	M10 240556	UK [England]	2010	invasive (unspecified/other)	Neisseria meningitidis	B	B	B	14	1	1	142	13	3	6	108	5	636	6	9	ST-41/44 complex	9815	13	3	1
19989	M10 240558	UK [Wales]	2010	invasive (unspecified/other)	Neisseria meningitidis	B	B	B	14	1	1	142	13	3	6	19	193	3	6	9	ST-41/44 complex	1992	13	3	1
19990	M10 240559	UK [Wales]	2010	invasive (unspecified/other)	Neisseria meningitidis	B	B	B	1	6	3	100	7	1	5	13	53	26	41	3	ST-162 complex	162	7	1	1
19991	M10 240566	UK [England]	2010	invasive (unspecified/other)	Neisseria meningitidis	B	B	B	45	33	1	87	14	7	5	1	13	36	53	15	ST-213 complex	213	NA	NA	3

19992	M10 240568	UK [England]	2010	invasive (unspecified/other)	Neisseria meningitidis	B	B	B	15	4	9	193	1	7	5	1	13	36	53	15	ST-213 complex	213	1	1	1
19993	M10 240569	UK [England]	2010	invasive (unspecified/other)	Neisseria meningitidis	B	B	B	37	1	1	408	4	7	8	10	19	10	1	2	ST-18 complex	18	4	3	1
19994	M10 240570	UK [England]	2010	invasive (unspecified/other)	Neisseria meningitidis	B	B	B	4	2	79	84	2	3	206	9	5	9	6	9	ST-41/44 complex	5357	2	3	1
19995	M10 240572	UK [England]	2010	invasive (unspecified/other)	Neisseria meningitidis	B	B	B	13	3	57	145	3	4	10	34	5	38	11	9	ST-269 complex	1161	3	3	1
19996	M10 240578	UK [England]	2010	invasive (unspecified/other)	Neisseria meningitidis	B	B	B	598	6	3	205	7	8	10	5	4	5	3	8	ST-32 complex	34	7	1	1
19997	M10 240579	UK [England]	2010	invasive (unspecified/other)	Neisseria meningitidis	B	B	B	4	1	57	206	4	47	3	58	31	24	87	13		3687	4	3	1
19998	M10 240580	UK [England]	2010	invasive (unspecified/other)	Neisseria meningitidis	Y	Y	Y	25	1	1	26	4	12	5	18	9	11	9	17	ST-23 complex	1655	4	4	2
19999	M10 240581	UK [Northern Ireland]	2010	invasive (unspecified/other)	Neisseria meningitidis	NG	cnl	cnl	94	58	56	89	NA	5	4	38	15	22	40	13	ST-1136 complex	1136	NA	NA	3
20000	M10 240582	UK [England]	2010	invasive (unspecified/other)	Neisseria meningitidis	B	B	B	598	6	3	205	7	8	10	5	4	5	3	8	ST-32 complex	34	7	1	1
20001	M10 240583	UK [England]	2010	invasive (unspecified/other)	Neisseria meningitidis	B	B	B	13	3	57	145	3	4	10	2	5	38	11	46		9816	3	3	1
20002	M10 240585	UK [England]	2010	invasive (unspecified/other)	Neisseria meningitidis	B	B	B	4	2	1	84	2	3	6	9	5	3	6	9	ST-41/44 complex	46	2	3	1
20003	M10 240586	UK [England]	2010	invasive (unspecified/other)	Neisseria meningitidis	B	B	B	4	2	1	84	2	3	6	9	5	8	6	9	ST-41/44 complex	485	2	3	1
20004	M10 240587	UK [England]	2010	invasive (unspecified/other)	Neisseria meningitidis	B	B	B	24	1	63	25	4	27	6	9	626	9	6	16	ST-41/44 complex	9879	4	4	2
20005	M10 240589	UK [England]	2010	invasive (unspecified/other)	Neisseria meningitidis	B	B	B	14	7	1	142	6	3	6	9	5	9	22	9	ST-41/44 complex	40	6	2	1
20006	M10 240590	UK [England]	2010	invasive (unspecified/other)	Neisseria meningitidis	Y	Y	Y	25	1	1	26	4	10	5	18	9	11	9	17	ST-23 complex	23	4	4	2
20007	M10 240591	UK [England]	2010	invasive (unspecified/other)	Neisseria meningitidis	B	B	B	47	1	57	90	15	12	5	12	35	60	22	17	ST-461 complex	461	15	4	3
20008	M10 240592	UK [England]	2010	invasive (unspecified/other)	Neisseria meningitidis	B	B	B	14	7	1	142	6	3	7	9	5	17	22	9	ST-41/44 complex	6782	6	2	1
20009	M10 240595	UK [England]	2010	invasive (unspecified/other)	Neisseria meningitidis	B	B	B	31	9	15	58	16	7	5	1	13	36	53	15	ST-213 complex	213	16	2	3
20010	M10 240597	UK [England]	2010	invasive (unspecified/other)	Neisseria meningitidis	B	B	B	4	2	79	84	2	3	6	9	5	9	6	9	ST-41/44 complex	41	2	3	1
20011	M10 240598	UK [England]	2010	invasive (unspecified/other)	Neisseria meningitidis	B	B	B	14	7	1	142	6	3	6	9	5	9	6	9	ST-41/44 complex	41	6	2	1
20012	M10 240602	UK [England]	2010	invasive (unspecified/other)	Neisseria meningitidis	B	B	B	13	3	57	145	3	4	10	34	5	38	11	9	ST-269 complex	1161	3	3	1
20013	M10 240604	UK [Northern Ireland]	2010	invasive (unspecified/other)	Neisseria meningitidis	B	B	B	4	2	1	84	2	3	6	34	5	17	6	9	ST-41/44 complex	8384	2	3	1
20014	M10 240605	UK [England]	2010	invasive (unspecified/other)	Neisseria meningitidis	B	B	B	14	1	1	166	4	3	6	9	5	9	6	9	ST-41/44 complex	41	4	3	1
20015	M10 240606	UK [England]	2010	invasive (unspecified/other)	Neisseria meningitidis	B	B	B	621	1	77	91	4	7	5	1	13	36	53	15	ST-213 complex	213	4	4	3
20016	M10 240607	UK [England]	2010	invasive (unspecified/other)	Neisseria meningitidis	C	C	C	19	1	2	65	4	9	4	9	17	5	6	2	ST-103 complex	5133	4	4	2
20017	M10 240611	UK [England]	2010	invasive (unspecified/other)	Neisseria meningitidis	B	B	B	1	6	3	100	7	8	10	77	4	6	3	8	ST-32 complex	749	7	1	1
20018	M10 240612	UK [England]	2010	invasive (unspecified/other)	Neisseria meningitidis	B	B	B	15	10	2	193	17	4	10	15	9	8	11	9	ST-269 complex	269	NA	NA	1
20019	M10 240613	UK [England]	2010	invasive (unspecified/other)	Neisseria meningitidis	B	B	B	13	3	57	145	3	4	10	34	5	38	11	9	ST-269 complex	1161	3	3	1
20020	M10 240614	UK [England]	2010	invasive (unspecified/other)	Neisseria meningitidis	B	B	B	256	34	1	207	18	42	5	15	5	21	18	6		9817	NA	NA	1
20021	M10 240616	UK [England]	2010	invasive (unspecified/other)	Neisseria meningitidis	W	W	W	16	1	1	60	4	11	5	18	8	78	24	21	ST-22 complex	1224	4	4	2
20022	M10 240617	UK [England]	2010	invasive (unspecified/other)	Neisseria meningitidis	B	B	B	31	9	5	58	16	7	5	1	13	36	53	15	ST-213 complex	213	16	2	3
20023	M10 240618	UK [England]	2010	invasive (unspecified/other)	Neisseria meningitidis	E	E	E	13	3	1	145	3	12	5	19	17	3	172	124	ST-60 complex	2435	3	3	1
20024	M10 240619	UK [England]	2010	invasive (unspecified/other)	Neisseria meningitidis	B	B	B	4	2	1	84	2	7	5	1	13	36	53	15	ST-213 complex	213	2	3	1
20025	M10 240621	UK [Wales]	2010	invasive (unspecified/other)	Neisseria meningitidis	B	B	B	31	9	5	58	16	7	5	1	13	36	53	15	ST-213 complex	213	16	2	3
20026	M10 240622	UK [England]	2010	invasive (unspecified/other)	Neisseria meningitidis	B	B	B	13	3	57	145	3	4	422	18	5	38	11	9		9880	3	3	1
20027	M10 240623	UK [England]	2010	invasive (unspecified/other)	Neisseria meningitidis	B	B	B	357	4	12	208	1	4	10	34	5	38	11	9	ST-269 complex	1161	1	1	1
20028	M10 240624	UK [England]	2010	invasive (unspecified/other)	Neisseria meningitidis	B	B	B	13	3	57	145	3	4	10	2	5	38	11	9	ST-269 complex	275	3	3	1
20029	M10 240626	UK [England]	2010	invasive (unspecified/other)	Neisseria meningitidis	B	B	B	13	3	1	145	3	4	10	2	8	38	11	9	ST-269 complex	4401	3	3	1
20030	M10 240627	UK [England]	2010	invasive (unspecified/other)	Neisseria meningitidis	B	B	B	1	6	7	100	7	4	10	5	4	6	416	8	ST-32 complex	6083	7	1	1
20031	M10 240631	UK [England]	2010	invasive (unspecified/other)	Neisseria meningitidis	C	C	C	15	4	2	193	1	4	10	15	9	8	11	13	ST-269 complex	467	1	1	1
20032	M10 240632	UK [England]	2010	invasive (unspecified/other)	Neisseria meningitidis	Y	Y	Y	25	1	1	26	4	12	5	18	9	11	9	17	ST-23 complex	1655	4	4	2
20033	M10 240633	UK [England]	2010	invasive (unspecified/other)	Neisseria meningitidis	Y	Y	Y	25	1	1	26	4	12	5	18	9	11	9	17	ST-23 complex	1655	4	4	2
20034	M10 240634	UK [Northern Ireland]	2010	invasive (unspecified/other)	Neisseria meningitidis	B	B	B	14	7	1	142	6	10	6	9	5	9	6	9	ST-41/44 complex	42	6	2	1
20035	M10 240636	UK [England]	2010	invasive (unspecified/other)	Neisseria meningitidis	B	B	B	13	3	57	209	3	8	25	7	17	21	26	49	ST-1157 complex	1157	3	3	1
20036	M10 240638	UK [England]	2010	invasive (unspecified/other)	Neisseria meningitidis	B	B	B	19	1	1	83	4	4	10	2	5	38	11	9	ST-269 complex	275	4	4	2
20037	M10 240639	UK [England]	2010	invasive (unspecified/other)	Neisseria meningitidis	B	B	B	238	21	5	210	19	20	6	63	9	9	11	2		284	NA	NA	1
20038	M10 240640	UK [England]	2010	invasive (unspecified/other)	Neisseria meningitidis	B	B	B	19	1	1	83	4	4	4	2	5	38	11	9		1831	4	4	2
20039	M10 240641	UK [England]	2010	invasive (unspecified/other)	Neisseria meningitidis	W	W	W	16	1	1	60	4	11	5	18	8	11	4	21	ST-22 complex	184	4	4	2
20040	M10 240642	UK [England]	2010	invasive (unspecified/other)	Neisseria meningitidis	B	B	B	13	3	57	145	3	4	10	34	5	38	6	9		5335	3	3	1
20041	M10 240643	UK [England]	2010	invasive (unspecified/other)	Neisseria meningitidis	Y	Y	Y	25	1	1	26	4	10	5	18	9	11	9	17	ST-23 complex	23	4	4	2
20042	M10 240644	UK [England]	2010	invasive (unspecified/other)	Neisseria meningitidis	Y	Y	Y	15	4	2	193	1	8	5	18	17	11	24	21	ST-22 complex	3651	1	1	1
20043	M10 240645	UK [England]	2010	invasive (unspecified/other)	Neisseria meningitidis	B	B	B	15	4	2	193	1	4	10	15	9	8	11	17	ST-269 complex	283	1	1	1
20044	M10 240649	UK [England]	2010	invasive (unspecified/other)	Neisseria meningitidis	B	B	B	19	1	1	83	4	4	10	2	5	38	11	9	ST-269 complex	275	4	4	2
20045	M10 240650	UK [England]	2010	invasive (unspecified/other)	Neisseria meningitidis	B	B	B	16	56	79	60	NA	13	29	2	17	26	65	53		2123	NA	NA	2
20046	M10 240651	UK [England]	2010	invasive (unspecified/other)	Neisseria meningitidis	B	B	B	45	8	1	87	20	7	5	1	627	36	53	15	ST-213 complex	9881	20	4	3
20047	M10 240652	UK [England]	2010	invasive (unspecified/other)	Neisseria meningitidis	B	B	B	19	1	1	83	4	4	10	2	5	38	11	9	ST-269 complex	275	4	4	2
20048	M10 240657	UK [England]	2010	invasive (unspecified/other)	Neisseria meningitidis	B	B	B	599	4	4	92	1	4	10	11	18	6	10	12	ST-35 complex	35	1	1	2
20049	M10 240659	UK [England]	2010	invasive (unspecified/other)	Neisseria meningitidis	B	B	B	16	1	1	59	4	3	6	9	5	9	6	9	ST-41/44 complex	41	4	4	2
20050	M10 240661	UK [England]	2010	invasive (unspecified/other)	Neisseria meningitidis	B	B	B	13	3	57	145	3	4	10	34	5	38	11	9	ST-269 complex	1161	3	3	1
20051	M10 240662	UK [England]	2010	invasive (unspecified/other)	Neisseria meningitidis	B	B	B	4	2	1	84	2	3	6	9	628	9	6	9	ST-41/44 complex	9882	2	3	1
20052	M10 240665	UK [England]	2010	invasive (unspecified/other)	Neisseria meningitidis	B	B	B	45	11	77	87	10	7	5	1	13	36	53	15	ST-213 complex	213	NA	NA	3
20053	M10 240666	UK [England]	2010	invasive (unspecified/other)	Neisseria meningitidis	W/Y	W/Y	W/Y	21	1	1	7	4	6	5	173	13	5	24	17	ST-174 complex	1466	4	4	2
20054	M10 240668	UK [England]	2010	invasive (unspecified/other)	Neisseria meningitidis	X	X	X	19	1	1	83	4	4	10	2	13	3	11	295		9839	4	4	2
20055	M10 240669	UK [England]	2010	invasive (unspecified/other)	Neisseria meningitidis	B	B	B	14	1	1	142	13	3	6	19	193	3	6	9	ST-41/44 complex	1992	13	3	1
20056	M10 240670	UK [England]	2010	invasive (unspecified/other)	Neisseria meningitidis	B	B	B	15	4	2	193	1	4	10	15	9	8	11	9	ST-269 complex	269	1	1	1
20057	M10 240671	UK [England]	2010	invasive (unspecified/other)	Neisseria meningitidis	W	W	W	22	1	1	1	4	2	3	4	3	8	4	6	ST-11 complex	11	4	4	2
20058	M10 240674	UK [England]	2010	invasive (unspecified/other)	Neisseria meningitidis	B	B	B	4	2	1	84	2	3	6	9	5	9	6	9	ST-41/44 complex	41	2	3	1
20059	M10 240675	UK [England]	2010	invasive (unspecified/other)	Neisseria meningitidis	Y	Y	Y	19	1	1	93	13	2	16	6	17	9	18	8	ST-167 complex	168	13	4	2
20060	M10 240676	UK [England]	2010	invasive (unspecified/other)	Neisseria meningitidis	B	B	B	170	9	1	94	21	4	16	11	9	3	10	7		9828	NA	NA	3
20061	M10 240677	UK [England]	2010	invasive (unspecified/other)	Neisseria meningitidis	B	B	B	291	20	4	203	8	4	10	47	17	6	2	9		1575	NA	NA	1
20062	M10 240678	UK [England]	2010	invasive (unspecified/other)	Neisseria meningitidis	B	B	B	14	1	1	142	13	3	6	19	5	3	6	9	ST-41/44 complex	340	13	3	1
20063	M10 240681	UK [England]	2010	invasive (unspecified/other)	Neisseria meningitidis	B	B	B	14	7	1	142	6	3	6	9	5	650	6	8	ST-41/44 complex	9883	6	2	1
20064	M10 240682	UK [Northern Ireland]	2010	invasive (unspecified/other)	Neisseria meningitidis	B	B	B	600	4	2	211	1	4	10	15	9	8	11	9	ST-269 complex	269	1	1	1
20065	M10 240683	UK [Wales]	2010	invasive (unspecified/other)	Neisseria meningitidis	B	B	B	37	1	1	408	4	7	8	10	19	10	1	2	ST-18 complex	18	4	3	1
20066	M10 240684	UK [England]	2010	invasive (unspecified/other)	Neisseria meningitidis	B	B	B	601	5	1	212	11	2	3	4	3	8	4	6	ST-11 complex	11	11	2	1
20067	M10 240685	UK [England]	2010	invasive (unspecified/other)	Neisseria meningitidis	Y	Y	Y	25	1	1	26	4	12	5	18	9	11	9	17	ST-23 complex	1655	4	4	2
20068	M10 240687	UK [England]	2010	invasive (unspecified/other)	Neisseria meningitidis	B	B	B	13	3	1	145	3	17	5	19	17	3	26	2	ST-60 complex	60	3	3	1
20069	M10 240689	UK [England]	2010	invasive (unspecified/other)	Neisseria meningitidis	B	B	B	4	2	1	84	2	3	6	9	5	11	6	9	ST-41/44 complex	154	2	3	1
20070	M10 240690	UK [Wales]	2010	invasive (unspecified/other)	Neisseria meningitidis	B	B	B	4	2	1	84	2	3	6	153	5	9	6	9	ST-41/44 complex	1090	2	3	1
20071	M10 240693	UK [England]	2010	invasive (unspecified/other)	Neisseria meningitidis	B	B	B	13	3	1	145	3	17	5	47	17	3	26	2	ST-60 complex	1430	3	3	1
20072	M10 240694	UK [England]	2010	invasive (unspecified/other)	Neisseria meningitidis	Y	Y	Y	21	1	1	7	4	6	5	173	13	5	24	17	ST-174 complex	1466	4	4	2
20073	M10 240698	UK [England]	2010	invasive (unspecified/other)	Neisseria meningitidis	B	B	B	84	13	1	70	22	42	26	46	24	2	20	17	ST-282 complex	282	NA	NA	3
20074	M10 240700	UK [England]	2010	invasive (unspecified/other)	Neisseria meningitidis	Y	Y	Y	25	1	1	26	4	12	286	18	9	11	9	17	ST-23 complex	6463	4	4	2
20075	M10 240701	UK [England]	2010	invasive (unspecified/other)	Neisseria meningitidis	B	B	B	89	5	5	213	23	7	5	1	13	36	53	15	ST-213 complex	213	NA	NA	1
20076	M10 240702	UK [England]	2010	invasive (unspecified/other)	Neisseria meningitidis	B	B	B	4	2	1	84	2	3	6	9	5	11	6	9	ST-41/44 complex	154	2	3	1
20077	M10 240703	UK [England]	2010	invasive (unspecified/other)	Neisseria meningitidis	B	B	B	59	9	77	75	16	7	5	1	13	36	53	664	ST-213 complex	9884	16	2	3
20078	M10 240704	UK [England]	2010	invasive (unspecified/other)	Neisseria meningitidis	B	B	B	15	10	2	193	17	4	10	15	9	8	11	9	ST-269 complex	269	NA	NA	1
20079	M10 240705	UK [England]	2010	invasive (unspecified/other)	Neisseria meningitidis	C	C	C	19	1	2	65	4	9	4	9	17	5	6	2	ST-103 complex	5133	4	4	2
20080	M10 240707	UK [England]	2010	invasive (unspecified/other)	Neisseria meningitidis	B	B	B	4	2	1	84	2	3	6	34	5	11	6	9	ST-41/44 complex	1194	2	3	1
20081	M10 240708	UK [England]	2010	invasive (unspecified/other)	Neisseria meningitidis	B	B	B	15	4	2	193	1	4	10	15	9	8	5	9	ST-269 complex	1195	1	1	1
20082	M10 240709	UK [England]	2010	invasive (unspecified/other)	Neisseria meningitidis	B	B	B	15	4	2	193	1	4	10	15	17	8	11	17	ST-269 complex	479	1	1	1
20083	M10 240711	UK [England]	2010	invasive (unspecified/other)	Neisseria meningitidis	B	B	B	19	1	1	83	4	4	10	2	5	38	11	9	ST-269 complex	275	4	4	2
20084	M10 240712	UK [England]	2010	invasive (unspecified/other)	Neisseria meningitidis	B	B	B	45	8	77	87	20	7	5	1	13	36	53	15	ST-213 complex	213	20	4	3
20085	M10 240713	UK [England]	2010	invasive (unspecified/other)	Neisseria meningitidis	B	B	B	13	3	57	145	3	4	10	2	4	38	11	9	ST-269 complex	9829	3	3	1
20086	M10 240716	UK [England]	2010	invasive (unspecified/other)	Neisseria meningitidis	B	B	B	47	1	57	90	15	12	5	12	35	60	22	17	ST-461 complex	461	15	4	3
20087	M10 240717	UK [England]	2010	invasive (unspecified/other)	Neisseria meningitidis	B	B	B	45	61	77	87	NA	7	5	1	13	619	53	15	ST-213 complex	9830	NA	NA	3
20088	M10 240718	UK [England]	2010	invasive (unspecified/other)	Neisseria meningitidis	B	B	B	13	3	1	145	3	608	5	19	17	3	26	2	ST-60 complex	9885	3	3	1
20089	M10 240719	UK [England]	2010	invasive (unspecified/other)	Neisseria meningitidis	B	B	B	1	6	3	100	7	8	10	5	4	5	3	8	ST-32 complex	34	7	1	1
20090	M10 240720	UK [England]	2010	invasive (unspecified/other)	Neisseria meningitidis	B	B	B	15	4	2	193	1	4	10	15	17	8	11	17	ST-269 complex	479	1	1	1
20091	M10 240721	UK [England]	2010	invasive (unspecified/other)	Neisseria meningitidis	B	B	B	14	1	1	142	13	9	6	9	5	9	6	9	ST-41/44 complex	318	13	3	1
20092	M10 240722	UK [England]	2010	invasive (unspecified/other)	Neisseria meningitidis	B	B	B	14	7	1	142	6	3	6	9	5	9	6	9	ST-41/44 complex	41	6	2	1
20093	M10 240723	UK [Wales]	2010	invasive (unspecified/other)	Neisseria meningitidis	B	B	B	4	2	1	84	2	3	6	9	5	9	6	9	ST-41/44 complex	41	2	3	1
20094	M10 240724	UK [England]	2010	invasive (unspecified/other)	Neisseria meningitidis	B	B	B	4	2	1	84	2	3	6	34	5	11	6	9	ST-41/44 complex	1194	2	3	1
20095	M10 240728	UK [England]	2010	invasive (unspecified/other)	Neisseria meningitidis	B	B	B	622	4	2	190	1	4	10	15	9	8	239	9	ST-269 complex	9840	1	1	1
20096	M10 240729	UK [England]	2010	invasive (unspecified/other)	Neisseria meningitidis	B	B	B	15	4	2	193	1	4	10	15	17	8	11	9	ST-269 complex	1049	1	1	1
20097	M10 240730	UK [England]	2010	invasive (unspecified/other)	Neisseria meningitidis	B	B	B	23	1	1	4	4	10	6	9	5	9	641	12	ST-41/44 complex	9886	4	4	2
20098	M10 240731	UK [England]	2010	invasive (unspecified/other)	Neisseria meningitidis	B	B	B	19	1	1	83	4	4	423	2	5	38	11	9		9887	4	4	2
20099	M10 240732	UK [England]	2010	invasive (unspecified/other)	Neisseria meningitidis	Y	Y	Y	25	1	1	26	4	10	5	18	9	11	9	17	ST-23 complex	23	4	4	2
20100	M10 240733	UK [England]	2010	invasive (unspecified/other)	Neisseria meningitidis	B	B	B	602	26	5	216	25	4	10	2	5	3	11	2		3934	NA	NA	1
20101	M10 240737	UK [England]	2010	invasive (unspecified/other)	Neisseria meningitidis	W	W	W	16	1	1	60	4	11	5	18	154	11	24	21	ST-22 complex	1281	4	4	2
20102	M10 240742	UK [England]	2010	invasive (unspecified/other)	Neisseria meningitidis	B	B	B	603	27	16	95	26	7	5	13	13	36	53	15	ST-213 complex	8955	NA	NA	3
20103	M10 240743	UK [England]	2010	invasive (unspecified/other)	Neisseria meningitidis	B	B	B	15	4	2	193	1	4	10	15	9	8	11	9	ST-269 complex	269	1	1	1
20104	M10 240745	UK [England]	2010	invasive (unspecified/other)	Neisseria meningitidis	Y	Y	Y	25	1	1	26	4	12	5	18	9	11	9	17	ST-23 complex	1655	4	4	2
20105	M10 240746	UK [England]	2010	invasive (unspecified/other)	Neisseria meningitidis	B	B	B	4	2	1	84	2	3	6	9	13	9	6	9	ST-41/44 complex	9841	2	3	1
20106	M10 240747	UK [England]	2010	invasive (unspecified/other)	Neisseria meningitidis	B	B	B	19	1	1	65	4	9	20	9	9	9	6	2	ST-41/44 complex	1097	4	4	2
20107	M10 240748	UK [England]	2010	invasive (unspecified/other)	Neisseria meningitidis	B	B	B	4	2	1	84	2	3	6	9	5	8	6	9	ST-41/44 complex	485	2	3	1
20108	M10 240749	UK [England]	2010	invasive (unspecified/other)	Neisseria meningitidis	B	B	B	4	2	1	84	2	3	6	9	5	8	6	9	ST-41/44 complex	485	2	3	1
20109	M10 240750	UK [England]	2010	invasive (unspecified/other)	Neisseria meningitidis	B	B	B	14	7	1	142	6	3	6	9	5	9	7	9	ST-41/44 complex	8203	6	2	1
20110	M10 240751	UK [England]	2010	invasive (unspecified/other)	Neisseria meningitidis	B	B	B	45	8	77	87	20	7	5	1	13	36	53	15	ST-213 complex	213	20	4	3
20111	M10 240752	UK [England]	2010	invasive (unspecified/other)	Neisseria meningitidis	B	B	B	13	3	57	145	3	4	10	34	5	38	11	9	ST-269 complex	1161	3	3	1
20112	M10 240753	UK [England]	2010	invasive (unspecified/other)	Neisseria meningitidis	B	B	B	1	6	3	100	7	8	10	5	4	5	3	8	ST-32 complex	34	7	1	1
20113	M10 240754	UK [England]	2010	invasive (unspecified/other)	Neisseria meningitidis	B	B	B	4	2	1	84	2	3	6	9	5	3	6	9	ST-41/44 complex	46	2	3	1
20114	M10 240756	UK [England]	2010	invasive (unspecified/other)	Neisseria meningitidis	W	W	W	16	1	1	60	4	11	5	5	9	11	24	21	ST-22 complex	2114	4	4	2
20115	M10 240759	UK [England]	2010	invasive (unspecified/other)	Neisseria meningitidis	Y	Y	Y	21	1	1	7	4	6	5	173	13	5	24	17	ST-174 complex	1466	4	4	2
20116	M10 240761	UK [England]	2010	invasive (unspecified/other)	Neisseria meningitidis	B	B	B	4	2	1	84	2	3	6	9	5	9	56	9	ST-41/44 complex	1938	2	3	1
20117	M10 240762	UK [England]	2010	invasive (unspecified/other)	Neisseria meningitidis	B	B	B	4	2	1	84	2	3	6	9	5	9	6	9	ST-41/44 complex	41	2	3	1
20118	M10 240763	UK [England]	2010	invasive (unspecified/other)	Neisseria meningitidis	B	B	B	4	2	1	84	2	6	3	6	17	26	21	8		4051	2	3	1
20119	M10 240766	UK [England]	2010	invasive (unspecified/other)	Neisseria meningitidis	B	B	B	506	5	2	11	11	4	10	15	17	8	11	17	ST-269 complex	479	11	2	2
20120	M10 240767	UK [Wales]	2010	invasive (unspecified/other)	Neisseria meningitidis	B	B	B	1	6	3	100	7	8	10	5	4	5	3	8	ST-32 complex	34	7	1	1
20121	M10 240768	UK [England]	2010	invasive (unspecified/other)	Neisseria meningitidis	B	B	B	160	10	2	96	27	4	10	15	9	8	11	22	ST-269 complex	2873	NA	NA	3
20122	M10 240770	UK [England]	2010	invasive (unspecified/other)	Neisseria meningitidis	B	B	B	15	4	2	193	1	4	10	15	9	8	5	9	ST-269 complex	1195	1	1	1
20123	M10 240771	UK [England]	2010	invasive (unspecified/other)	Neisseria meningitidis	B	B	B	84	13	1	70	22	42	26	46	24	5	20	17	ST-282 complex	8068	NA	NA	3
20124	M10 240772	UK [England]	2010	invasive (unspecified/other)	Neisseria meningitidis	B	B	B	13	3	57	145	3	4	10	34	5	38	11	9	ST-269 complex	1161	3	3	1
20125	M10 240773	UK [England]	2010	invasive (unspecified/other)	Neisseria meningitidis	B	B	B	4	2	1	84	2	3	6	9	13	9	6	9	ST-41/44 complex	9841	2	3	1
20126	M10 240774	UK [Wales]	2010	invasive (unspecified/other)	Neisseria meningitidis	B	B	B	13	3	57	145	3	4	10	34	5	38	11	9	ST-269 complex	1161	3	3	1
20127	M10 240775	UK [England]	2010	invasive (unspecified/other)	Neisseria meningitidis	B	B	B	4	2	1	84	2	3	6	9	5	9	6	9	ST-41/44 complex	41	2	3	1
20128	M10 240776	UK [England]	2010	invasive (unspecified/other)	Neisseria meningitidis	Y	Y	Y	25	1	1	26	4	12	5	18	9	11	9	17	ST-23 complex	1655	4	4	2
20129	M10 240777	UK [England]	2010	invasive (unspecified/other)	Neisseria meningitidis	Y	Y	Y	25	1	1	26	4	10	5	18	9	11	9	17	ST-23 complex	23	4	4	2

20130	M10 240778	UK [Wales]	2010	invasive (unspecified/other)	Neisseria meningitidis	B	B	B		494	12	17	53	28	566	5	1	13	36	53	15	ST-213 complex	9187	NA	NA	3
20131	M10 240779	UK [England]	2010	invasive (unspecified/other)	Neisseria meningitidis	B	B	B		15	4	77	193	1	4	10	15	9	8	11	9	ST-269 complex	269	1	1	1
20132	M10 240781	UK [England]	2010	invasive (unspecified/other)	Neisseria meningitidis	Y	Y	Y		25	1	1	26	4	8	5	18	17	11	24	21	ST-22 complex	3651	4	4	2
20133	M10 240783	UK [England]	2010	invasive (unspecified/other)	Neisseria meningitidis	B	B	B		13	3	57	145	3	4	10	34	5	38	11	9	ST-269 complex	1161	3	3	1
20134	M10 240785	UK [England]	2010	invasive (unspecified/other)	Neisseria meningitidis	B	B	B		14	7	1	142	6	17	6	9	5	9	6	9	ST-41/44 complex	3447	6	2	1
20135	M10 240786	UK [England]	2010	invasive (unspecified/other)	Neisseria meningitidis	Y	Y	Y		25	1	1	26	4	12	5	18	9	30	9	17	ST-23 complex	9831	4	4	2
20136	M10 240787	UK [England]	2010	invasive (unspecified/other)	Neisseria meningitidis	Y	Y	Y		25	1	1	26	4	12	5	18	9	11	9	17	ST-23 complex	1655	4	4	2
20137	M10 240788	UK [Wales]	2010	invasive (unspecified/other)	Neisseria meningitidis	B	B	B		4	2	79	84	2	3	16	325	5	9	6	9	ST-41/44 complex	5981	2	3	1
20138	M10 240789	UK [Wales]	2010	invasive (unspecified/other)	Neisseria meningitidis	B	B	B		15	4	2	193	1	4	10	15	9	8	11	17	ST-269 complex	283	1	1	1
20139	M10 240790	UK [England]	2010	invasive (unspecified/other)	Neisseria meningitidis	B	B	B		1	6	3	100	7	4	10	5	40	6	3	8	ST-32 complex	259	7	1	1
20140	M10 240793	UK [England]	2010	invasive (unspecified/other)	Neisseria meningitidis	B	B	B		14	7	1	142	6	3	7	9	5	17	22	9	ST-41/44 complex	6782	6	2	1
20141	M10 240794	UK [England]	2010	invasive (unspecified/other)	Neisseria meningitidis	B	B	B		1	6	3	100	7	4	10	5	4	6	3	8	ST-32 complex	32	7	1	1
20142	M10 240796	UK [England]	2010	invasive (unspecified/other)	Neisseria meningitidis	B	B	B		202	1	1	35	4	27	6	9	3	9	6	16	ST-41/44 complex	136	4	4	2
20143	M10 240798	UK [England]	2010	invasive (unspecified/other)	Neisseria meningitidis	Y	Y	Y		25	1	1	26	4	12	5	18	9	11	9	17	ST-23 complex	1655	4	4	2
20144	M10 240799	UK [England]	2010	invasive (unspecified/other)	Neisseria meningitidis	B	B	B		45	62	77	87	29	7	5	1	13	36	53	15	ST-213 complex	213	NA	NA	3
20145	M10 240803	UK [England]	2010	invasive (unspecified/other)	Neisseria meningitidis	B	B	B		4	2	1	84	2	3	6	34	5	11	6	9	ST-41/44 complex	1194	2	3	1
20146	M10 240804	UK [England]	2010	invasive (unspecified/other)	Neisseria meningitidis	Y	Y	Y		25	1	1	26	4	12	5	18	9	11	9	17	ST-23 complex	1655	4	4	2
20147	M10 240805	UK [England]	2010	invasive (unspecified/other)	Neisseria meningitidis	B	B	B		13	3	57	145	3	4	10	34	NA	38	11	330		NA	3	3	1
20148	M10 240807	UK [England]	2010	invasive (unspecified/other)	Neisseria meningitidis	B	B	B		4	2	1	84	2	3	6	9	5	9	6	9	ST-41/44 complex	41	2	3	1
20149	M10 240808	UK [England]	2010	invasive (unspecified/other)	Neisseria meningitidis	B	B	B		15	4	2	193	1	4	10	6	9	8	11	9	ST-269 complex	1092	1	1	1
20150	M10 240809	UK [England]	2010	invasive (unspecified/other)	Neisseria meningitidis	B	B	B		4	2	1	84	2	3	6	9	5	9	6	9	ST-41/44 complex	41	2	3	1
20151	M10 240810	UK [England]	2010	invasive (unspecified/other)	Neisseria meningitidis	B	B	B		21	1	1	7	4	1	5	13	53	26	41	3	ST-162 complex	162	4	4	2
20152	M10 240811	UK [England]	2010	invasive (unspecified/other)	Neisseria meningitidis	B	B	B		13	3	57	209	3	8	424	7	17	21	26	49	ST-1157 complex	9888	3	3	1
20153	M10 240814	UK [Wales]	2010	invasive (unspecified/other)	Neisseria meningitidis	B	B	B		4	2	1	84	2	4	10	34	5	38	11	9	ST-269 complex	1161	2	3	1
20154	M10 240817	UK [England]	2010	invasive (unspecified/other)	Neisseria meningitidis	W	W	W		22	1	1	1	4	2	3	4	3	8	4	6	ST-11 complex	11	4	4	2
20155	M10 240818	UK [England]	2010	invasive (unspecified/other)	Neisseria meningitidis	C	C	C		306	5	1	217	11	2	3	4	3	8	4	6	ST-11 complex	11	11	2	1
20156	M10 240819	UK [England]	2010	invasive (unspecified/other)	Neisseria meningitidis	B	B	B		19	1	1	83	4	4	10	2	5	3	11	9	ST-269 complex	1163	4	4	2
20157	M10 240820	UK [England]	2010	invasive (unspecified/other)	Neisseria meningitidis	B	B	B		4	2	6	84	2	3	6	9	5	9	6	9	ST-41/44 complex	41	2	3	1
20158	M10 240821	UK [England]	2010	invasive (unspecified/other)	Neisseria meningitidis	W	W	W		22	1	1	1	4	2	3	4	3	8	4	6	ST-11 complex	11	4	4	2
20159	M10 240822	UK [England]	2010	invasive (unspecified/other)	Neisseria meningitidis	W	W	W		16	1	1	60	4	11	5	18	8	78	24	21	ST-22 complex	1224	4	4	2
20160	M10 240823	UK [England]	2010	invasive (unspecified/other)	Neisseria meningitidis	B	B	B		19	1	1	65	4	9	20	9	9	9	6	2	ST-41/44 complex	1097	4	4	2
20161	M10 240824	UK [England]	2010	invasive (unspecified/other)	Neisseria meningitidis	B	B	B		1	6	7	100	7	4	10	5	4	6	3	8	ST-32 complex	32	7	1	1
20162	M10 240825	UK [England]	2010	invasive (unspecified/other)	Neisseria meningitidis	B	B	B		13	3	77	145	3	7	5	19	17	3	26	2	ST-60 complex	9818	3	3	1
20163	M10 240826	UK [England]	2010	invasive (unspecified/other)	Neisseria meningitidis	B	B	B		13	3	57	145	3	4	10	34	5	6	11	9	ST-269 complex	7789	3	3	1
30072	M11 240099 b	UK	2011	invasive (unspecified/other)	Neisseria meningitidis	W		W		22	1	1	1	4	2	3	4	3	8	4	6	ST-11 complex	11	4	4	2
20164	M11 240000	UK [England]	2011	invasive (unspecified/other)	Neisseria meningitidis	Y	Y	Y		14	1	1	142	13	10	5	18	9	11	9	17	ST-23 complex	23	13	3	1
20165	M11 240001	UK [England]	2011	invasive (unspecified/other)	Neisseria meningitidis	B	B	B		13	3	57	209	3	12	5	12	35	192	22	17	ST-461 complex	1946	3	3	1
20166	M11 240002	UK [England]	2011	invasive (unspecified/other)	Neisseria meningitidis	B	B	B		14	1	57	142	13	6	5	105	12	6	13	17		4954	13	3	1
20167	M11 240003	UK [England]	2011	invasive (unspecified/other)	Neisseria meningitidis	B	B	B		8	21	1	218	19	9	20	9	629	9	6	2	ST-41/44 complex	9889	NA	NA	1
20168	M11 240004	UK [Wales]	2011	invasive (unspecified/other)	Neisseria meningitidis	B	B	B		13	3	57	145	3	4	10	34	5	38	11	9	ST-269 complex	1161	3	3	1
20169	M11 240005	UK [England]	2011	invasive (unspecified/other)	Neisseria meningitidis	Y	Y	Y		25	1	1	26	4	10	5	18	9	11	9	17	ST-23 complex	23	4	4	2
20170	M11 240006	UK [Wales]	2011	invasive (unspecified/other)	Neisseria meningitidis	B	B	B		1	6	3	100	7	8	10	5	4	6	3	15	ST-32 complex	8049	7	1	1
20171	M11 240007	UK [England]	2011	invasive (unspecified/other)	Neisseria meningitidis	Y	Y	Y		25	1	NA	26	4	12	5	18	9	NA	9	17		NA	4	4	2
20172	M11 240008	UK [England]	2011	invasive (unspecified/other)	Neisseria meningitidis	B	B	B		605	6	1	219	7	3	6	19	5	3	6	8	ST-41/44 complex	2783	7	1	1
20173	M11 240010	UK [England]	2011	invasive (unspecified/other)	Neisseria meningitidis	B	B	B		15	4	2	193	1	4	10	15	9	8	11	9	ST-269 complex	269	1	1	1
20174	M11 240011	UK [England]	2011	invasive (unspecified/other)	Neisseria meningitidis	B	B	B		14	7	1	142	6	10	6	9	5	9	6	9	ST-41/44 complex	42	6	2	1
20175	M11 240012	UK [England]	2011	invasive (unspecified/other)	Neisseria meningitidis	B	B	B		4	2	1	84	2	3	6	9	5	11	6	9	ST-41/44 complex	154	2	3	1
20176	M11 240013	UK [England]	2011	invasive (unspecified/other)	Neisseria meningitidis	B	B	B		510	28	8	599	30	4	10	5	630	6	3	8	ST-32 complex	9890	NA	NA	1
20177	M11 240014	UK [England]	2011	invasive (unspecified/other)	Neisseria meningitidis	B	B	B		47	1	57	90	15	12	5	6	35	17	642	17	ST-461 complex	9891	15	4	3
20178	M11 240015	UK [England]	2011	invasive (unspecified/other)	Neisseria meningitidis	B	B	B		14	1	1	142	13	4	10	2	5	38	11	1		1159	13	3	1
20179	M11 240016	UK [Wales]	2011	invasive (unspecified/other)	Neisseria meningitidis	B	B	B		62	35	18	221	31	4	10	5	26	6	3	8	ST-32 complex	1096	NA	NA	1
20180	M11 240017	UK [Northern Ireland]	2011	invasive (unspecified/other)	Neisseria meningitidis	B	B	B		14	5	1	204	11	3	6	494	5	3	6	9	ST-41/44 complex	9819	11	2	1
20181	M11 240018	UK [England]	2011	invasive (unspecified/other)	Neisseria meningitidis	B	B	B		4	2	1	84	2	11	6	9	5	9	375	9	ST-41/44 complex	9820	2	3	1
20182	M11 240019	UK [England]	2011	invasive (unspecified/other)	Neisseria meningitidis	B	B	B		16	1	4	59	4	4	10	11	53	6	10	12	ST-35 complex	8063	4	4	2
20183	M11 240021	UK [England]	2011	invasive (unspecified/other)	Neisseria meningitidis	B	B	B		1	6	3	100	7	4	10	5	40	6	3	8	ST-32 complex	259	7	1	1
20184	M11 240022	UK [Wales]	2011	invasive (unspecified/other)	Neisseria meningitidis	B	B	B		13	3	57	145	3	4	10	34	5	38	11	9	ST-269 complex	1161	3	3	1
20185	M11 240023	UK [Wales]	2011	invasive (unspecified/other)	Neisseria meningitidis	B	B	B		4	2	1	84	2	3	6	9	5	9	6	9	ST-41/44 complex	41	2	3	1
20186	M11 240024	UK [Wales]	2011	invasive (unspecified/other)	Neisseria meningitidis	B	B	B		19	1	1	65	4	9	20	9	9	9	6	2	ST-41/44 complex	1097	4	4	2
20187	M11 240025	UK [England]	2011	invasive (unspecified/other)	Neisseria meningitidis	B	B	B		15	4	2	193	1	4	10	15	17	8	11	9	ST-269 complex	1049	1	1	1
20188	M11 240026	UK [England]	2011	invasive (unspecified/other)	Neisseria meningitidis	B	B	B		15	4	2	193	1	4	10	15	17	8	11	9	ST-269 complex	1049	1	1	1
20189	M11 240027	UK [England]	2011	invasive (unspecified/other)	Neisseria meningitidis	B	B	B		13	3	1	145	3	17	5	19	17	3	26	2	ST-60 complex	60	3	3	1
20190	M11 240028	UK [England]	2011	invasive (unspecified/other)	Neisseria meningitidis	B	B	B		13	3	1	145	3	17	5	19	17	3	26	2	ST-60 complex	60	3	3	1
20191	M11 240029	UK [England]	2011	invasive (unspecified/other)	Neisseria meningitidis	W	W	W		16	22	1	97	32	11	5	168	8	11	4	21	ST-22 complex	1286	NA	NA	2
20192	M11 240030	UK [England]	2011	invasive (unspecified/other)	Neisseria meningitidis	B	B	B		15	4	2	193	1	4	10	15	9	8	11	9	ST-269 complex	269	1	1	1
20193	M11 240031	UK [England]	2011	invasive (unspecified/other)	Neisseria meningitidis	Y	Y	Y		25	1	1	26	4	12	5	18	9	11	9	17	ST-23 complex	1655	4	4	2
20194	M11 240032	UK [England]	2011	invasive (unspecified/other)	Neisseria meningitidis	B	B	B		13	3	57	145	3	4	5	34	5	38	11	9		5849	3	3	1
20195	M11 240034	UK [England]	2011	invasive (unspecified/other)	Neisseria meningitidis	B	B	B		86	4	2	222	1	3	6	19	7	3	6	9	ST-41/44 complex	5861	1	1	1
20196	M11 240035	UK [Wales]	2011	invasive (unspecified/other)	Neisseria meningitidis	W	W	W		22	1	1	1	4	2	3	4	3	8	4	6	ST-11 complex	11	4	4	2
20197	M11 240036	UK [Wales]	2011	invasive (unspecified/other)	Neisseria meningitidis	Y	Y	Y		25	1	1	26	4	12	5	18	9	11	9	17	ST-23 complex	1655	4	4	2
20198	M11 240037	UK [England]	2011	invasive (unspecified/other)	Neisseria meningitidis	B	B	B		1	6	3	100	7	8	10	5	4	5	3	8	ST-32 complex	34	7	1	1
20199	M11 240038	UK [England]	2011	invasive (unspecified/other)	Neisseria meningitidis	Y	Y	Y		19	1	1	93	13	2	16	6	17	9	18	8	ST-167 complex	168	13	4	2
20200	M11 240039	UK [Wales]	2011	invasive (unspecified/other)	Neisseria meningitidis	B	B	B		7	63	75	223	33	42	26	46	24	6	20	17	ST-282 complex	1802	NA	NA	1
20201	M11 240040	UK [England]	2011	invasive (unspecified/other)	Neisseria meningitidis	B	B	B		4	2	1	84	2	16	6	34	5	11	6	9	ST-41/44 complex	1196	2	3	1
20202	M11 240041	UK [England]	2011	invasive (unspecified/other)	Neisseria meningitidis	B	B	B		4	2	1	84	2	3	6	6	5	9	6	9	ST-41/44 complex	477	2	3	1
20203	M11 240042	UK [England]	2011	invasive (unspecified/other)	Neisseria meningitidis	B	B	B		47	1	57	90	15	12	5	12	35	192	22	17	ST-461 complex	1946	15	4	3
20204	M11 240043	UK [England]	2011	invasive (unspecified/other)	Neisseria meningitidis	Y	Y	Y		25	1	1	26	4	12	5	18	25	11	9	17	ST-23 complex	9842	4	4	2
20205	M11 240044	UK [Wales]	2011	invasive (unspecified/other)	Neisseria meningitidis	B	B	B		15	4	2	193	1	4	10	12	9	8	5	9	ST-269 complex	9843	1	1	1
20206	M11 240045	UK [England]	2011	invasive (unspecified/other)	Neisseria meningitidis	B	B	B		37	1	1	408	4	7	8	10	26	10	18	16	ST-18 complex	858	4	3	1
20207	M11 240046	UK [England]	2011	invasive (unspecified/other)	Neisseria meningitidis	B	B	B		275	64	77	224	34	7	5	1	13	36	53	15	ST-213 complex	213	NA	NA	1
20208	M11 240047	UK [England]	2011	invasive (unspecified/other)	Neisseria meningitidis	B	B	B		4	2	79	84	2	3	6	9	5	9	6	9	ST-41/44 complex	41	2	3	1
20209	M11 240048	UK [England]	2011	invasive (unspecified/other)	Neisseria meningitidis	B	B	B		13	3	1	145	3	17	5	19	17	3	26	2	ST-60 complex	60	3	3	1
20210	M11 240050	UK [England]	2011	invasive (unspecified/other)	Neisseria meningitidis	B	B	B		45	8	77	87	20	7	5	1	13	36	53	15	ST-213 complex	213	20	4	3
20211	M11 240052	UK [Wales]	2011	invasive (unspecified/other)	Neisseria meningitidis	B	B	B		47	1	1	90	15	12	5	12	35	60	22	17	ST-461 complex	461	15	4	3
20212	M11 240053	UK [England]	2011	invasive (unspecified/other)	Neisseria meningitidis	B	B	B		14	7	1	142	6	3	6	9	5	9	6	9	ST-41/44 complex	41	6	2	1
20213	M11 240054	UK [Wales]	2011	invasive (unspecified/other)	Neisseria meningitidis	B	B	B		4	2	1	84	2	3	6	9	5	9	6	9	ST-41/44 complex	41	2	3	1
20214	M11 240055	UK [Wales]	2011	invasive (unspecified/other)	Neisseria meningitidis	B	B	B		4	2	1	84	2	3	6	9	5	9	6	9	ST-41/44 complex	41	2	3	1
20215	M11 240056	UK [England]	2011	invasive (unspecified/other)	Neisseria meningitidis	B	B	B		606	5	1	916	11	2	3	4	3	8	4	6	ST-11 complex	11	11	2	1
20216	M11 240057	UK [England]	2011	invasive (unspecified/other)	Neisseria meningitidis	W	W	W		22	1	1	1	4	2	3	4	3	8	4	6	ST-11 complex	11	4	4	2
20217	M11 240058	UK [England]	2011	invasive (unspecified/other)	Neisseria meningitidis	B	B	B		13	3	10	145	3	4	10	2	5	38	11	9	ST-269 complex	275	3	3	1
20218	M11 240059	UK [England]	2011	invasive (unspecified/other)	Neisseria meningitidis	B	B	B		4	2	1	84	2	5	4	9	5	9	6	9	ST-41/44 complex	9832	2	3	1
20219	M11 240060	UK [England]	2011	invasive (unspecified/other)	Neisseria meningitidis	B	B	B		95	5	1	32	11	4	10	72	9	6	10	12	ST-35 complex	2380	11	2	2
20220	M11 240061	UK [England]	2011	invasive (unspecified/other)	Neisseria meningitidis	B	B	B		318	36	5	226	35	7	5	1	13	3	53	271	ST-213 complex	9833	NA	NA	1
20221	M11 240062	UK [England]	2011	invasive (unspecified/other)	Neisseria meningitidis	B	B	B		19	1	1	83	4	4	10	2	5	38	11	9	ST-269 complex	275	4	4	2
20222	M11 240063	UK [England]	2011	invasive (unspecified/other)	Neisseria meningitidis	B	B	B		4	2	1	84	2	3	6	9	5	9	6	9	ST-41/44 complex	41	2	3	1
20223	M11 240064	UK [England]	2011	invasive (unspecified/other)	Neisseria meningitidis	Y	Y	Y		16	1	1	60	4	219	25	275	17	11	8	21		7786	4	4	2
20224	M11 240065	UK [England]	2011	invasive (unspecified/other)	Neisseria meningitidis	Y	Y	Y		25	1	1	26	4	12	5	18	9	11	9	17	ST-23 complex	1655	4	4	2
20225	M11 240066	UK [England]	2011	invasive (unspecified/other)	Neisseria meningitidis	B	B	B		13	3	57	145	3	4	10	2	5	38	11	9	ST-269 complex	275	3	3	1
20226	M11 240067	UK [England]	2011	invasive (unspecified/other)	Neisseria meningitidis	W	W	W		22	1	1	1	4	2	3	4	3	8	4	6	ST-11 complex	11	4	4	2
20227	M11 240068	UK [England]	2011	invasive (unspecified/other)	Neisseria meningitidis	NG	Y	Y		119	1	1	98	4	6	3	4	9	21	13	169		9844	4	4	2
20228	M11 240069	UK [England]	2011	invasive (unspecified/other)	Neisseria meningitidis	B	B	B		4	2	1	84	2	4	10	11	18	6	10	12	ST-35 complex	35	2	3	1
20229	M11 240070	UK [England]	2011	invasive (unspecified/other)	Neisseria meningitidis	B	B	B		15	4	2	193	1	4	10	15	9	8	11	9	ST-269 complex	269	1	1	1
20230	M11 240071	UK [England]	2011	invasive (unspecified/other)	Neisseria meningitidis	Y	Y	Y		23	1	1	4	4	2	7	6	9	16	9	8	ST-167 complex	767	4	4	2
20231	M11 240072	UK [England]	2011	invasive (unspecified/other)	Neisseria meningitidis	B	B	B		19	1	1	83	4	4	10	2	5	38	11	9	ST-269 complex	275	4	4	2
20232	M11 240073	UK [England]	2011	invasive (unspecified/other)	Neisseria meningitidis	Y	Y	Y		21	1	1	7	4	6	5	173	13	5	24	17	ST-174 complex	1466	4	4	2
20233	M11 240074	UK [Wales]	2011	invasive (unspecified/other)	Neisseria meningitidis	B	B	B		45	8	1	87	20	7	5	1	13	36	53	15	ST-213 complex	213	20	4	3
20234	M11 240076	UK [England]	2011	invasive (unspecified/other)	Neisseria meningitidis	C	C	C		22	1	1	1	4	2	3	4	3	8	4	6	ST-11 complex	11	4	4	2
20235	M11 240077	UK [England]	2011	invasive (unspecified/other)	Neisseria meningitidis	B	B	B		15	4	2	193	1	4	10	15	9	8	11	9	ST-269 complex	269	1	1	1
20236	M11 240079	UK [England]	2011	invasive (unspecified/other)	Neisseria meningitidis	B	B	B		4	2	79	84	2	3	6	9	5	9	6	9	ST-41/44 complex	41	2	3	1
20237	M11 240081	UK [England]	2011	invasive (unspecified/other)	Neisseria meningitidis	B	B	B		4	2	1	84	2	3	6	34	5	11	6	9	ST-41/44 complex	1194	2	3	1
20238	M11 240082	UK [England]	2011	invasive (unspecified/other)	Neisseria meningitidis	B	B	B		1	6	3	100	7	8	3	5	4	1	3	8	ST-32 complex	290	7	1	1
20239	M11 240083	UK [England]	2011	invasive (unspecified/other)	Neisseria meningitidis	B	B	B		47	1	1	90	15	12	5	12	35	60	22	17	ST-461 complex	461	15	4	3
20240	M11 240084	UK [Wales]	2011	invasive (unspecified/other)	Neisseria meningitidis	B	B	B		37	65	1	408	36	7	8	10	5	10	1	2	ST-18 complex	4997	NA	NA	1
20241	M11 240085	UK [England]	2011	invasive (unspecified/other)	Neisseria meningitidis	B	B	B		14	1	1	142	13	3	6	19	5	3	6	9	ST-41/44 complex	340	13	3	1
20242	M11 240086	UK [England]	2011	invasive (unspecified/other)	Neisseria meningitidis	B	B	B		143	4	2	99	1	4	10	15	9	14	11	17	ST-269 complex	1774	1	1	3
20243	M11 240088	UK [England]	2011	invasive (unspecified/other)	Neisseria meningitidis	B	B	B		4	2	1	84	2	3	6	9	5	8	6	9	ST-41/44 complex	485	2	3	1
20244	M11 240094	UK [Wales]	2011	invasive (unspecified/other)	Neisseria meningitidis	C	C	C		19	1	2	65	4	9	4	9	17	5	6	2	ST-103 complex	5133	4	4	2
20245	M11 240096	UK [England]	2011	invasive (unspecified/other)	Neisseria meningitidis	Y	Y	Y		25	1	1	26	4	12	5	18	9	11	9	17	ST-23 complex	1655	4	4	2
20246	M11 240097	UK [England]	2011	invasive (unspecified/other)	Neisseria meningitidis	B	B	B		4	2	1	84	2	3	6	9	25	9	6	9	ST-41/44 complex	2799	2	3	1
20247	M11 240099	UK [England]	2011	invasive (unspecified/other)	Neisseria meningitidis	W	W	W		22	1	1	1	4	2	3	4	3	8	4	6	ST-11 complex	11	4	4	2
20248	M11 240100	UK [England]	2011	invasive (unspecified/other)	Neisseria meningitidis	B	B	B		25	1	1	26	4	8	4	1	17	5	18	2	ST-103 complex	9239	4	4	2
20249	M11 240108	UK [England]	2011	invasive (unspecified/other)	Neisseria meningitidis	B	B	B		14	5	1	204	11	3	6	19	5	3	6	2	ST-41/44 complex	2632	11	2	1
20250	M11 240109	UK [England]	2011	invasive (unspecified/other)	Neisseria meningitidis	B	B	B	NA		1	57	NA	4	4	5	5	4	6	3	8	ST-32 complex	2931	4	4	NA
20251	M11 240110	UK [England]	2011	invasive (unspecified/other)	Neisseria meningitidis	B	B	B		4	2	1	84	2	3	6	34	5	11	6	9	ST-41/44 complex	1194	2	3	1
20252	M11 240111	UK [England]	2011	invasive (unspecified/other)	Neisseria meningitidis	B	B	B		30	17	77	54	37	7	5	1	13	36	53	15	ST-213 complex	213	NA	NA	3
20253	M11 240112	UK [England]	2011	invasive (unspecified/other)	Neisseria meningitidis	B	B	B		14	7	1	142	6	3	6	9	8	9	22	9	ST-41/44 complex	8052	6	2	1
20254	M11 240113	UK [England]	2011	invasive (unspecified/other)	Neisseria meningitidis	B	B	B		45	8	77	87	20	7	5	1	13	36	53	6	ST-213 complex	2666	20	4	3
20255	M11 240115	UK [Wales]	2011	invasive (unspecified/other)	Neisseria meningitidis	B	B	B		4	2	79	84	2	3	16	325	5	9	6	9	ST-41/44 complex	5981	2	3	1
20256	M11 240116	UK [England]	2011	invasive (unspecified/other)	Neisseria meningitidis	Y	Y	Y		25	1	1	26	4	12	5	18	9	11	9	17	ST-23 complex	1655	4	4	2
20257	M11 240117	UK [England]	2011	invasive (unspecified/other)	Neisseria meningitidis	B	B	B		118	5	1	73	11	17	5	19	17	3	53	2	ST-60 complex	9821	11	2	2
20470	M11 240118	UK [England]	2011	invasive (unspecified/other)	Neisseria meningitidis	B	B	B		4	2	1	84	2	3	6	9	5	9	6	9	ST-41/44 complex	41	2	3	1
20258	M11 240119	UK [England]	2011	invasive (unspecified/other)	Neisseria meningitidis	B	B	B		30	17	11	54	37	9	6	9	17	9	6	9	ST-41/44 complex	437	NA	NA	3
20259	M11 240121	UK [England]	2011	invasive (unspecified/other)	Neisseria meningitidis	B	B	B		31	9	5	58	16	7	5	1	13	36	53	15	ST-213 complex	213	16	2	3
20260	M11 240122	UK [England]	2011	invasive (unspecified/other)	Neisseria meningitidis	B	B	B		13	3	57	209	3	4	10	34	5	38	11	330		4713	3	3	1
20261	M11 240123	UK [England]	2011	invasive (unspecified/other)	Neisseria meningitidis	B	B	B		22	1	1	1	4	2	3	4	256	8	4	6	ST-11 complex	3537	4	4	2
20263	M11 240125	UK [England]	2011	invasive (unspecified/other)	Neisseria meningitidis	B	B	B		4	2	1	84	2	3	6	9	3	11	6	9	ST-41/44 complex	3818	2	3	1
20264	M11 240126	UK [England]	2011	invasive (unspecified/other)	Neisseria meningitidis	Y	Y	Y		25	1	1	26	4	10	5	18	9	11	9	17	ST-23 complex	23	4	4	2
20265	M11 240128	UK [England]	2011	invasive (unspecified/other)	Neisseria meningitidis	B	B	B		13	3	57	145	3	4	10	34	5	38	11	330		4713	3	3	1
20266	M11 240129	UK [England]	2011	invasive (unspecified/other)	Neisseria meningitidis	B	B	B		84	13	1	70	22	42	26	46	24	2	20	17	ST-282 complex	282	NA	NA	3

20267	M11 240131	UK [England]	2011	invasive (unspecified/other)	Neisseria meningitidis	B	B	B	21	1	1	7	4	1	5	13	53	26	41	3	ST-162 complex	162	4	4	2
20268	M11 240132	UK [England]	2011	invasive (unspecified/other)	Neisseria meningitidis	B	B	B	15	4	77	193	1	4	10	15	9	8	11	9	ST-269 complex	269	1	1	1
20269	M11 240133	UK [Wales]	2011	invasive (unspecified/other)	Neisseria meningitidis	B	B	B	37	1	1	408	4	7	8	10	19	10	1	2	ST-18 complex	18	4	3	1
20270	M11 240136	UK [England]	2011	invasive (unspecified/other)	Neisseria meningitidis	B	B	B	45	8	1	87	20	7	5	1	13	36	53	15	ST-213 complex	213	20	4	3
20271	M11 240137	UK [England]	2011	invasive (unspecified/other)	Neisseria meningitidis	B	B	B	45	33	77	87	14	7	5	1	13	36	53	15	ST-213 complex	213	NA	NA	3
20272	M11 240139	UK [England]	2011	invasive (unspecified/other)	Neisseria meningitidis	B	B	B	13	3	57	145	3	4	10	34	5	38	11	9	ST-269 complex	1161	3	3	1
20273	M11 240145	UK [England]	2011	invasive (unspecified/other)	Neisseria meningitidis	B	B	B	19	1	57	65	4	3	6	9	5	9	18	9	ST-41/44 complex	2080	4	4	2
20274	M11 240146	UK [England]	2011	invasive (unspecified/other)	Neisseria meningitidis	B	B	B	19	1	1	83	4	4	10	2	5	38	11	9	ST-269 complex	275	4	4	2
20275	M11 240147	UK [England]	2011	invasive (unspecified/other)	Neisseria meningitidis	B	B	B	45	8	77	87	20	7	5	1	13	36	53	15	ST-213 complex	213	20	4	3
20276	M11 240148	UK [England]	2011	invasive (unspecified/other)	Neisseria meningitidis	B	B	B	4	2	79	84	2	3	6	9	5	9	6	9	ST-41/44 complex	41	2	3	1
20277	M11 240149	UK [England]	2011	invasive (unspecified/other)	Neisseria meningitidis	B	B	B	4	2	1	84	2	3	6	9	5	9	6	2	ST-41/44 complex	2314	2	3	1
20278	M11 240150	UK [Wales]	2011	invasive (unspecified/other)	Neisseria meningitidis	B	B	B	15	4	2	193	1	4	10	15	9	3	11	9	ST-269 complex	7939	1	1	1
20279	M11 240151	UK [England]	2011	invasive (unspecified/other)	Neisseria meningitidis	B	B	B	15	4	2	193	1	4	10	15	17	8	11	17	ST-269 complex	479	1	1	1
20280	M11 240152	UK [England]	2011	invasive (unspecified/other)	Neisseria meningitidis	B	B	B	14	1	1	142	13	3	6	108	5	9	6	9	ST-41/44 complex	1475	13	3	1
20281	M11 240157	UK [England]	2011	invasive (unspecified/other)	Neisseria meningitidis	Y	Y	Y	25	1	1	26	4	515	5	18	NA	11	9	17		NA	4	4	2
20282	M11 240161	UK [England]	2011	invasive (unspecified/other)	Neisseria meningitidis	Y	Y	Y	226	66	1	228	38	6	5	173	13	5	24	17	ST-174 complex	1466	NA	NA	1
20283	M11 240163	UK [England]	2011	invasive (unspecified/other)	Neisseria meningitidis	B	B	B	19	1	1	65	4	11	6	9	9	9	6	9	ST-41/44 complex	327	4	4	2
20284	M11 240164	UK [England]	2011	invasive (unspecified/other)	Neisseria meningitidis	B	B	B	86	4	2	222	1	3	6	19	7	3	6	9	ST-41/44 complex	5861	1	1	1
20285	M11 240165	UK [England]	2011	invasive (unspecified/other)	Neisseria meningitidis	Y	Y	Y	254	1	1	229	4	6	5	173	13	5	24	17	ST-174 complex	1466	4	3	1
20286	M11 240166	UK [England]	2011	invasive (unspecified/other)	Neisseria meningitidis	Y	Y	Y	25	1	1	26	4	10	5	18	9	11	9	17	ST-23 complex	23	4	4	2
20287	M11 240167	UK [England]	2011	invasive (unspecified/other)	Neisseria meningitidis	B	B	B	13	3	1	145	3	563	5	19	90	3	26	2	ST-60 complex	9327	3	3	1
20288	M11 240168	UK [England]	2011	invasive (unspecified/other)	Neisseria meningitidis	W	W	W	22	1	1	1	4	2	3	4	3	8	4	6	ST-11 complex	11	4	4	2
20289	M11 240170	UK [England]	2011	invasive (unspecified/other)	Neisseria meningitidis	B	B	B	14	5	1	204	11	3	6	19	NA	3	6	665		NA	11	2	1
20290	M11 240173	UK [England]	2011	invasive (unspecified/other)	Neisseria meningitidis	B	B	B	4	2	79	84	2	3	16	325	5	9	6	9	ST-41/44 complex	5981	2	3	1
20291	M11 240174	UK [England]	2011	invasive (unspecified/other)	Neisseria meningitidis	B	B	B	16	1	4	59	4	4	10	11	18	6	10	12	ST-35 complex	35	4	4	2
20292	M11 240175	UK [England]	2011	invasive (unspecified/other)	Neisseria meningitidis	B	B	B	1	6	3	100	7	8	10	5	4	6	3	8	ST-32 complex	33	7	1	1
20293	M11 240176	UK [England]	2011	invasive (unspecified/other)	Neisseria meningitidis	B	B	B	607	67	77	230	39	8	5	19	17	5	24	54		3138	NA	NA	1
20294	M11 240180	UK [England]	2011	invasive (unspecified/other)	Neisseria meningitidis	B	B	B	15	4	2	193	1	4	10	15	17	8	11	9	ST-269 complex	1049	1	1	1
20295	M11 240181	UK [England]	2011	invasive (unspecified/other)	Neisseria meningitidis	B	B	B	187	5	77	101	11	7	5	1	13	36	53	15	ST-213 complex	213	11	2	3
20296	M11 240182	UK [Wales]	2011	invasive (unspecified/other)	Neisseria meningitidis	B	B	B	1	6	3	100	7	8	35	5	162	6	3	15		9834	7	1	1
20297	M11 240183	UK [England]	2011	invasive (unspecified/other)	Neisseria meningitidis	B	B	B	49	1	57	102	13	4	10	5	4	6	3	8	ST-32 complex	32	13	4	2
20298	M11 240185	UK [England]	2011	invasive (unspecified/other)	Neisseria meningitidis	B	B	B	15	4	2	231	1	4	10	15	17	8	11	9	ST-269 complex	1049	1	1	1
20299	M11 240188	UK [England]	2011	invasive (unspecified/other)	Neisseria meningitidis	B	B	B	4	2	1	84	2	3	6	9	5	9	6	9	ST-41/44 complex	41	2	3	1
20300	M11 240189	UK [England]	2011	invasive (unspecified/other)	Neisseria meningitidis	B	B	B	84	13	1	70	22	42	26	46	24	5	20	17	ST-282 complex	8068	NA	NA	3
20301	M11 240191	UK [England]	2011	invasive (unspecified/other)	Neisseria meningitidis	B	B	B	4	2	1	84	2	3	6	9	5	8	6	666	ST-41/44 complex	9892	2	3	1
20302	M11 240192	UK [Wales]	2011	invasive (unspecified/other)	Neisseria meningitidis	Y	Y	Y	25	1	1	26	4	12	5	18	9	11	9	17	ST-23 complex	1655	4	4	2
20303	M11 240193	UK [England]	2011	invasive (unspecified/other)	Neisseria meningitidis	B	B	B	486	4	2	103	1	9	20	9	9	9	6	2	ST-41/44 complex	1097	1	1	2
20304	M11 240195	UK [England]	2011	invasive (unspecified/other)	Neisseria meningitidis	B	B	B	14	1	1	142	13	3	6	108	5	9	6	9	ST-41/44 complex	1475	13	3	1
20305	M11 240203	UK [Northern Ireland]	2011	invasive (unspecified/other)	Neisseria meningitidis	B	B	B	14	1	1	142	13	3	6	19	5	3	6	9	ST-41/44 complex	340	13	3	1
20306	M11 240206	UK [England]	2011	invasive (unspecified/other)	Neisseria meningitidis	B	B	B	47	1	1	90	15	12	5	12	35	60	22	17	ST-461 complex	461	15	4	3
20307	M11 240207	UK [England]	2011	invasive (unspecified/other)	Neisseria meningitidis	C	C	C	19	1	2	65	4	6	6	63	9	9	11	9	ST-41/44 complex	839	4	4	2
20308	M11 240209	UK [England]	2011	invasive (unspecified/other)	Neisseria meningitidis	C	Y	Y	21	1	1	7	4	6	5	173	13	5	24	17	ST-174 complex	1466	4	4	2
20309	M11 240210	UK [England]	2011	invasive (unspecified/other)	Neisseria meningitidis	Y	Y	Y	25	1	1	26	4	10	5	18	9	11	9	17	ST-23 complex	23	4	4	2
20310	M11 240211	UK [Northern Ireland]	2011	invasive (unspecified/other)	Neisseria meningitidis	Y	Y	Y	21	1	1	7	4	6	30	173	631	5	24	17	ST-174 complex	9893	4	4	2
20311	M11 240212	UK [England]	2011	invasive (unspecified/other)	Neisseria meningitidis	B	B	B	19	68	1	104	40	4	10	2	5	38	11	9	ST-269 complex	275	NA	NA	2
20312	M11 240213	UK [England]	2011	invasive (unspecified/other)	Neisseria meningitidis	B	B	B	4	2	1	84	2	3	6	34	5	11	6	9	ST-41/44 complex	1194	2	3	1
20313	M11 240214	UK [England]	2011	invasive (unspecified/other)	Neisseria meningitidis	B	B	B	47	1	57	90	15	12	5	12	35	192	22	17	ST-461 complex	1946	15	4	3
20314	M11 240215	UK [England]	2011	invasive (unspecified/other)	Neisseria meningitidis	Y	Y	Y	25	1	1	26	4	12	5	18	9	11	9	17	ST-23 complex	1655	4	4	2
20315	M11 240216	UK [England]	2011	invasive (unspecified/other)	Neisseria meningitidis	Y	Y	Y	25	1	1	26	4	12	5	18	9	11	9	17	ST-23 complex	1655	4	4	2
20316	M11 240226	UK [England]	2011	invasive (unspecified/other)	Neisseria meningitidis	B	B	B	15	4	2	193	1	4	10	15	9	8	11	9	ST-269 complex	269	1	1	1
20317	M11 240227	UK [England]	2011	invasive (unspecified/other)	Neisseria meningitidis	Y	Y	Y	25	1	1	26	4	12	286	18	9	11	9	17	ST-23 complex	6463	4	4	2
20318	M11 240231	UK [England]	2011	invasive (unspecified/other)	Neisseria meningitidis	W	W	W	16	1	1	60	4	11	5	18	8	11	4	21	ST-22 complex	184	4	4	2
20319	M11 240232	UK [England]	2011	invasive (unspecified/other)	Neisseria meningitidis	B	B	B	4	2	1	84	2	4	10	11	18	6	10	12	ST-35 complex	35	2	3	1
20320	M11 240233	UK [England]	2011	invasive (unspecified/other)	Neisseria meningitidis	NG	NG	NG	609	1	1	105	4	12	27	6	7	8	18	9	ST-226 complex	9835	4	4	2
20321	M11 240234	UK [England]	2011	invasive (unspecified/other)	Neisseria meningitidis	C	C	C	1	6	77	100	7	6	5	393	17	21	24	2		5238	7	1	1
20322	M11 240236	UK [England]	2011	invasive (unspecified/other)	Neisseria meningitidis	B	B	B	13	3	57	145	3	4	10	34	5	38	11	9	ST-269 complex	1161	3	3	1
20323	M11 240237	UK [England]	2011	invasive (unspecified/other)	Neisseria meningitidis	B	B	B	19	1	1	65	4	20	3	13	93	6	155	77		3482	4	4	2
20324	M11 240241	UK [England]	2011	invasive (unspecified/other)	Neisseria meningitidis	B	B	B	4	2	1	84	2	3	6	34	5	11	6	9	ST-41/44 complex	1194	2	3	1
20325	M11 240242	UK [England]	2011	invasive (unspecified/other)	Neisseria meningitidis	B	B	B	4	2	1	84	2	3	6	34	5	11	6	9	ST-41/44 complex	1194	2	3	1
20326	M11 240243	UK [England]	2011	invasive (unspecified/other)	Neisseria meningitidis	B	B	B	13	3	57	145	3	4	10	2	8	38	19	9		9845	3	3	1
20327	M11 240244	UK [England]	2011	invasive (unspecified/other)	Neisseria meningitidis	B	B	B	14	7	1	142	6	3	116	9	5	9	22	9	ST-41/44 complex	1960	6	2	1
20328	M11 240246	UK [England]	2011	invasive (unspecified/other)	Neisseria meningitidis	NG	E	E	13	3	1	145	3	17	5	19	17	3	26	2	ST-60 complex	60	3	3	1
20329	M11 240247	UK [England]	2011	invasive (unspecified/other)	Neisseria meningitidis	C	C	C	0	5	1	917	11	2	3	4	3	8	4	6	ST-11 complex	11	11	2	NA
20330	M11 240248	UK [England]	2011	invasive (unspecified/other)	Neisseria meningitidis	B	B	B	4	2	1	84	2	3	6	34	5	11	6	9	ST-41/44 complex	1194	2	3	1
20331	M11 240249	UK [England]	2011	invasive (unspecified/other)	Neisseria meningitidis	B	B	B	16	1	1	60	4	43	5	6	60	3	19	15		8944	4	4	2
20332	M11 240251	UK [England]	2011	invasive (unspecified/other)	Neisseria meningitidis	B	B	B	15	4	2	193	1	4	10	15	9	8	11	9	ST-269 complex	269	1	1	1
20333	M11 240255	UK [England]	2011	invasive (unspecified/other)	Neisseria meningitidis	B	B	B	4	2	1	84	2	3	6	153	5	9	6	9	ST-41/44 complex	1090	2	3	1
20334	M11 240256	UK [England]	2011	invasive (unspecified/other)	Neisseria meningitidis	B	B	B	4	2	1	84	2	3	6	9	5	36	6	9	ST-41/44 complex	9822	2	3	1
20335	M11 240258	UK [England]	2011	invasive (unspecified/other)	Neisseria meningitidis	Y	Y	Y	25	1	1	26	4	10	5	18	9	11	9	17	ST-23 complex	23	4	4	2
20336	M11 240259	UK [England]	2011	invasive (unspecified/other)	Neisseria meningitidis	B	B	B	4	2	1	84	2	3	6	9	5	9	6	9	ST-41/44 complex	41	2	3	1
20337	M11 240261	UK [England]	2011	invasive (unspecified/other)	Neisseria meningitidis	W	W	W	16	1	1	60	4	11	5	18	8	11	4	21	ST-22 complex	184	4	4	2
20338	M11 240262	UK [England]	2011	invasive (unspecified/other)	Neisseria meningitidis	A	A	A	94	37	1	106	198	1	1	2	1	3	334	19	ST-5 complex	4789	NA	NA	3
20339	M11 240263	UK [England]	2011	invasive (unspecified/other)	Neisseria meningitidis	Y	Y	Y	25	1	1	26	4	12	5	18	9	11	9	17	ST-23 complex	1655	4	4	2
20340	M11 240265	UK [England]	2011	invasive (unspecified/other)	Neisseria meningitidis	B	B	B	15	4	2	193	1	4	10	300	9	8	11	9	ST-269 complex	9836	1	1	1
20341	M11 240266	UK [England]	2011	invasive (unspecified/other)	Neisseria meningitidis	B	B	B	611	9	5	107	21	7	5	1	13	36	53	15	ST-213 complex	213	NA	NA	3
20342	M11 240268	UK [England]	2011	invasive (unspecified/other)	Neisseria meningitidis	B	B	B	4	2	1	84	2	7	5	1	13	36	53	15	ST-213 complex	213	2	3	1
20343	M11 240269	UK [England]	2011	invasive (unspecified/other)	Neisseria meningitidis	B	B	B	4	2	1	84	2	3	6	153	5	9	6	9	ST-41/44 complex	1090	2	3	1
20344	M11 240275	UK [England]	2011	invasive (unspecified/other)	Neisseria meningitidis	B	B	B	15	4	2	193	1	4	10	15	9	8	11	9	ST-269 complex	269	1	1	1
20345	M11 240276	UK [England]	2011	invasive (unspecified/other)	Neisseria meningitidis	B	B	B	4	2	6	84	2	3	6	9	5	9	6	9	ST-41/44 complex	41	2	3	1
20346	M11 240277	UK [England]	2011	invasive (unspecified/other)	Neisseria meningitidis	Y	Y	Y	25	1	1	26	4	12	5	18	NA	11	9	17		NA	4	4	2
20347	M11 240278	UK [England]	2011	invasive (unspecified/other)	Neisseria meningitidis	Y	Y	Y	25	1	1	26	4	12	5	18	9	11	9	17	ST-23 complex	1655	4	4	2
20348	M11 240280	UK [Northern Ireland]	2011	invasive (unspecified/other)	Neisseria meningitidis	B	B	B	24	1	4	25	4	4	10	47	17	6	2	12	ST-35 complex	278	4	4	2
20349	M11 240281	UK [England]	2011	invasive (unspecified/other)	Neisseria meningitidis	B	B	B	15	4	2	193	1	4	10	2	5	38	11	9	ST-269 complex	275	1	1	1
20350	M11 240282	UK [England]	2011	invasive (unspecified/other)	Neisseria meningitidis	B	B	B	31	69	5	58	41	7	5	1	13	36	53	15	ST-213 complex	213	NA	NA	3
20351	M11 240283	UK [England]	2011	invasive (unspecified/other)	Neisseria meningitidis	Y	Y	Y	25	1	1	26	4	10	5	18	9	11	9	17	ST-23 complex	23	4	4	2
20352	M11 240284	UK [England]	2011	invasive (unspecified/other)	Neisseria meningitidis	B	B	B	13	3	57	145	3	4	10	34	5	38	11	9	ST-269 complex	1161	3	3	1
20353	M11 240285	UK [England]	2011	invasive (unspecified/other)	Neisseria meningitidis	B	B	B	144	1	4	232	4	8	10	5	4	6	3	8	ST-32 complex	33	4	3	1
20354	M11 240286	UK [England]	2011	invasive (unspecified/other)	Neisseria meningitidis	B	B	B	4	2	1	84	2	6	3	675	17	26	21	667		9894	2	3	1
20355	M11 240287	UK [Northern Ireland]	2011	invasive (unspecified/other)	Neisseria meningitidis	B	B	B	584	38	1	108	42	12	5	339	17	8	21	8	ST-865 complex	9846	NA	NA	2
20356	M11 240289	UK [Northern Ireland]	2011	invasive (unspecified/other)	Neisseria meningitidis	B	B	B	14	5	1	204	11	3	6	19	5	3	6	9	ST-41/44 complex	340	11	2	1
20357	M11 240290	UK [England]	2011	invasive (unspecified/other)	Neisseria meningitidis	B	B	B	94	8	77	89	43	609	5	1	13	36	53	15	ST-213 complex	9895	NA	NA	3
20358	M11 240291	UK [England]	2011	invasive (unspecified/other)	Neisseria meningitidis	B	B	B	4	2	1	84	2	3	6	9	5	17	6	9	ST-41/44 complex	2363	2	3	1
20359	M11 240294	UK [England]	2011	invasive (unspecified/other)	Neisseria meningitidis	C	C	C	306	5	1	217	11	2	3	4	3	8	4	6	ST-11 complex	11	11	2	1
20360	M11 240295	UK [England]	2011	invasive (unspecified/other)	Neisseria meningitidis	B	B	B	4	2	1	84	2	3	6	9	9	9	6	9	ST-41/44 complex	303	2	3	1
20361	M11 240296	UK [England]	2011	invasive (unspecified/other)	Neisseria meningitidis	B	B	B	1	6	3	100	7	8	10	5	4	5	3	8	ST-32 complex	34	7	1	1
20362	M11 240297	UK [England]	2011	invasive (unspecified/other)	Neisseria meningitidis	B	B	B	45	8	77	87	20	7	5	1	13	36	53	15	ST-213 complex	213	20	4	3
20363	M11 240298	UK [England]	2011	invasive (unspecified/other)	Neisseria meningitidis	Y	Y	Y	25	1	1	26	4	12	5	18	9	11	9	17	ST-23 complex	1655	4	4	2
20364	M11 240299	UK [England]	2011	invasive (unspecified/other)	Neisseria meningitidis	B	B	B	4	2	1	84	2	3	6	9	5	9	6	2	ST-41/44 complex	2314	2	3	1
20365	M11 240302	UK [England]	2011	invasive (unspecified/other)	Neisseria meningitidis	W	W	W	16	1	1	60	4	11	5	18	8	11	4	21	ST-22 complex	184	4	4	2
20366	M11 240303	UK [England]	2011	invasive (unspecified/other)	Neisseria meningitidis	B	B	B	19	1	1	83	4	4	10	2	5	38	11	9	ST-269 complex	275	4	4	2
20367	M11 240304	UK [England]	2011	invasive (unspecified/other)	Neisseria meningitidis	B	B	B	160	70	1	96	44	46	5	2	7	26	20	2		2888	NA	NA	3
20368	M11 240305	UK [England]	2011	invasive (unspecified/other)	Neisseria meningitidis	W	W	W	22	1	1	1	4	2	3	4	3	8	4	6	ST-11 complex	11	4	4	2
20369	M11 240306	UK [England]	2011	invasive (unspecified/other)	Neisseria meningitidis	B	B	B	4	2	1	84	2	3	6	9	5	9	6	9	ST-41/44 complex	41	2	3	1
20370	M11 240309	UK [England]	2011	invasive (unspecified/other)	Neisseria meningitidis	B	B	B	19	1	1	83	4	4	10	2	17	3	11	22		9837	4	4	2
20371	M11 240311	UK [England]	2011	invasive (unspecified/other)	Neisseria meningitidis	B	B	B	4	2	79	84	2	3	6	9	632	9	6	9	ST-41/44 complex	9896	2	3	1
20372	M11 240312	UK [England]	2011	invasive (unspecified/other)	Neisseria meningitidis	Y	Y	Y	25	1	1	26	4	10	5	18	9	11	9	17	ST-23 complex	23	4	4	2
20373	M11 240313	UK [England]	2011	invasive (unspecified/other)	Neisseria meningitidis	Y	Y	Y	25	1	1	26	4	12	5	18	9	11	9	17	ST-23 complex	1655	4	4	2
20374	M11 240314	UK [England]	2011	invasive (unspecified/other)	Neisseria meningitidis	B	B	B	13	3	57	145	3	4	10	34	5	38	11	9	ST-269 complex	1161	3	3	1
20375	M11 240315	UK [England]	2011	invasive (unspecified/other)	Neisseria meningitidis	B	B	B	13	3	57	145	3	4	10	34	5	38	11	9	ST-269 complex	1161	3	3	1
20376	M11 240316	UK [England]	2011	invasive (unspecified/other)	Neisseria meningitidis	B	B	B	506	5	2	11	11	4	10	15	17	8	11	17	ST-269 complex	479	11	2	2
20377	M11 240317	UK [England]	2011	invasive (unspecified/other)	Neisseria meningitidis	B	B	B	15	4	2	193	1	4	10	15	9	8	11	9	ST-269 complex	269	1	1	1
20378	M11 240318	UK [Northern Ireland]	2011	invasive (unspecified/other)	Neisseria meningitidis	B	B	B	13	3	57	145	3	4	10	2	5	21	11	9	ST-269 complex	9004	3	3	1
20379	M11 240319	UK [England]	2011	invasive (unspecified/other)	Neisseria meningitidis	Y	Y	Y	25	1	1	26	4	12	5	18	9	11	9	17	ST-23 complex	1655	4	4	2
20380	M11 240322	UK [England]	2011	invasive (unspecified/other)	Neisseria meningitidis	Y	Y	Y	25	1	1	26	4	12	5	18	9	11	9	17	ST-23 complex	1655	4	4	2
20381	M11 240323	UK [England]	2011	invasive (unspecified/other)	Neisseria meningitidis	B	B	B	13	3	1	145	3	12	5	19	15	3	10	124		9847	3	3	1
20382	M11 240324	UK [England]	2011	invasive (unspecified/other)	Neisseria meningitidis	C	C	C	1	6	7	100	7	4	10	5	4	6	3	8	ST-32 complex	32	7	1	1
20383	M11 240326	UK [England]	2011	invasive (unspecified/other)	Neisseria meningitidis	Y	Y	Y	25	1	1	26	4	12	5	18	9	11	9	17	ST-23 complex	1655	4	4	2
20384	M11 240328	UK [England]	2011	invasive (unspecified/other)	Neisseria meningitidis	B	B	B	4	2	1	84	2	3	6	34	NA	11	6	9		NA	2	3	1
20385	M11 240330	UK [England]	2011	invasive (unspecified/other)	Neisseria meningitidis	B	B	B	13	3	57	145	3	4	10	34	5	38	11	9	ST-269 complex	1161	3	3	1
20386	M11 240333	UK [England]	2011	invasive (unspecified/other)	Neisseria meningitidis	B	B	B	24	1	1	25	4	6	6	9	9	9	6	17	ST-41/44 complex	833	4	4	2
20387	M11 240334	UK [England]	2011	invasive (unspecified/other)	Neisseria meningitidis	B	B	B	13	3	57	145	3	4	10	34	13	38	11	9	ST-269 complex	6604	3	3	1
20388	M11 240335	UK [England]	2011	invasive (unspecified/other)	Neisseria meningitidis	B	B	B	21	1	1	7	4	1	5	13	53	26	41	3	ST-162 complex	162	4	4	2
20389	M11 240337	UK [England]	2011	invasive (unspecified/other)	Neisseria meningitidis	B	B	B	494	12	20	53	28	7	5	1	13	36	53	15	ST-213 complex	213	NA	NA	3
20390	M11 240338	UK [England]	2011	invasive (unspecified/other)	Neisseria meningitidis	B	B	B	13	3	57	209	3	8	25	7	17	21	6	49	ST-1157 complex	3737	3	3	1
20391	M11 240339	UK [England]	2011	invasive (unspecified/other)	Neisseria meningitidis	B	B	B	13	3	1	145	3	17	5	19	17	3	26	2	ST-60 complex	60	3	3	1
20392	M11 240341	UK [England]	2011	invasive (unspecified/other)	Neisseria meningitidis	B	B	B	4	2	1	84	2	3	6	9	5	8	6	9	ST-41/44 complex	485	2	3	1
20393	M11 240342	UK [England]	2011	invasive (unspecified/other)	Neisseria meningitidis	B	B	B	1	6	7	100	7	4	10	5	4	6	3	8	ST-32 complex	32	7	1	1
20394	M11 240343	UK [England]	2011	invasive (unspecified/other)	Neisseria meningitidis	B	B	B	4	2	1	84	2	10	6	9	5	79	6	9	ST-41/44 complex	1200	2	3	1
20395	M11 240344	UK [England]	2011	invasive (unspecified/other)	Neisseria meningitidis	B	B	B	45	8	77	87	20	7	5	1	13	36	53	15	ST-213 complex	213	20	4	3
20396	M11 240346	UK [England]	2011	invasive (unspecified/other)	Neisseria meningitidis	Y	Y	Y	19	1	1	93	13	2	16	6	17	9	18	8	ST-167 complex	168	13	4	2
20397	M11 240347	UK [England]	2011	invasive (unspecified/other)	Neisseria meningitidis	C	C	C	19	1	2	65	4	9	4	9	17	5	6	2	ST-103 complex	5133	4	4	2
20398	M11 240348	UK [England]	2011	invasive (unspecified/other)	Neisseria meningitidis	B	B	B	45	14	77	87	45	7	5	1	13	36	53	15	ST-213 complex	213	NA	NA	3
20399	M11 240349	UK [England]	2011	invasive (unspecified/other)	Neisseria meningitidis	W	W	W	84	12	1	70	28	11	5	18	8	11	24	21	ST-22 complex	22	NA	NA	3
20400	M11 240350	UK [England]	2011	invasive (unspecified/other)	Neisseria meningitidis	B	B	B	14	1	1	142	13	3	6	9	17	9	6	15	ST-41/44 complex	9838	13	3	1
20401	M11 240352	UK [England]	2011	invasive (unspecified/other)	Neisseria meningitidis	B	B	B	13	3	57	145	3	4	10	34	5	38	11	9	ST-269 complex	1161	3	3	1
20402	M11 240353	UK [England]	2011	invasive (unspecified/other)	Neisseria meningitidis	B	B	B	19	1	1	83	4	4	10	2	5	38	11	9	ST-269 complex	275	4	4	2
20403	M11 240356	UK [England]	2011	invasive (unspecified/other)	Neisseria meningitidis	B	B	B	1	6	7	100	7	4	10	5	4	6	3	8	ST-32 complex	32	7	1	1
20404	M11 240358	UK [England]	2011	invasive (unspecified/other)	Neisseria meningitidis	W/Y	W/Y	W/Y	25	1	1	26	4	10	5	18	9	11	9	17	ST-23 complex	23	4	4	2

20405	M11 240360	UK [England]	2011	invasive (unspecified/other)	Neisseria meningitidis	B	B	B	19	1	1	65	4	9	6	9	9	9	6	2	ST-41/44 complex	180	4	4	2
20406	M11 240363	UK [England]	2011	invasive (unspecified/other)	Neisseria meningitidis	C	C	C	19	1	2	65	4	9	4	9	17	5	6	360		5315	4	4	2
20407	M11 240364	UK [England]	2011	invasive (unspecified/other)	Neisseria meningitidis	B	B	B	4	2	1	84	2	3	6	9	5	11	6	9	ST-41/44 complex	154	2	3	1
20408	M11 240365	UK [England]	2011	invasive (unspecified/other)	Neisseria meningitidis	B	B	B	4	2	1	84	2	3	6	9	5	9	6	9	ST-41/44 complex	41	2	3	1
20409	M11 240366	UK [Northern Ireland]	2011	invasive (unspecified/other)	Neisseria meningitidis	B	B	B	144	1	4	232	4	8	10	5	4	6	3	8	ST-32 complex	33	4	3	1
20410	M11 240367	UK [England]	2011	invasive (unspecified/other)	Neisseria meningitidis	B	B	B	4	2	1	84	2	3	6	9	5	3	6	9	ST-41/44 complex	46	2	3	1
20411	M11 240368	UK [England]	2011	invasive (unspecified/other)	Neisseria meningitidis	B	B	B	15	4	2	193	1	4	10	15	9	8	11	9	ST-269 complex	269	1	1	1
20412	M11 240369	UK [England]	2011	invasive (unspecified/other)	Neisseria meningitidis	B	B	B	4	2	1	84	2	3	6	9	5	9	6	9	ST-41/44 complex	41	2	3	1
20413	M11 240371	UK [England]	2011	invasive (unspecified/other)	Neisseria meningitidis	B	B	B	21	71	56	7	46	1	5	13	53	26	41	3	ST-162 complex	162	NA	NA	2
20414	M11 240372	UK [England]	2011	invasive (unspecified/other)	Neisseria meningitidis	NG	Y	Y	25	1	1	26	4	12	5	18	9	11	9	17	ST-23 complex	1655	4	4	2
20415	M11 240373	UK [England]	2011	invasive (unspecified/other)	Neisseria meningitidis	B	B	B	4	2	1	84	2	3	6	34	5	11	6	9	ST-41/44 complex	1194	2	3	1
20416	M11 240375	UK [England]	2011	invasive (unspecified/other)	Neisseria meningitidis	B	B	B	15	4	2	193	1	4	10	15	9	8	11	9	ST-269 complex	269	1	1	1
20417	M11 240376	UK [England]	2011	invasive (unspecified/other)	Neisseria meningitidis	B	B	B	16	4	2	110	1	4	10	15	9	8	11	9	ST-269 complex	269	1	1	2
20418	M11 240381	UK [England]	2011	invasive (unspecified/other)	Neisseria meningitidis	B	B	B	21	1	1	7	4	1	5	13	53	26	41	3	ST-162 complex	162	4	4	2
20419	M11 240382	UK [England]	2011	invasive (unspecified/other)	Neisseria meningitidis	B	B	B	4	2	1	84	2	3	6	9	5	8	6	9	ST-41/44 complex	485	2	3	1
20420	M11 240383	UK [England]	2011	invasive (unspecified/other)	Neisseria meningitidis	B	B	B	119	1	1	111	4	4	10	12	9	6	10	12	ST-35 complex	3077	4	4	2
20421	M11 240386	UK [England]	2011	invasive (unspecified/other)	Neisseria meningitidis	B	B	B	4	2	1	84	2	3	6	9	5	9	6	9	ST-41/44 complex	41	2	3	1
20422	M11 240387	UK [England]	2011	invasive (unspecified/other)	Neisseria meningitidis	W	W	W	138	5	1	112	11	11	5	18	8	11	4	21	ST-22 complex	184	11	2	2
20423	M11 240388	UK [England]	2011	invasive (unspecified/other)	Neisseria meningitidis	B	B	B	15	4	56	193	1	4	10	15	9	8	11	79	ST-269 complex	9823	1	1	1
20424	M11 240389	UK [England]	2011	invasive (unspecified/other)	Neisseria meningitidis	W	W	W	9	1	1	187	47	2	3	4	3	8	4	6	ST-11 complex	11	NA	NA	1
20425	M11 240390	UK [England]	2011	invasive (unspecified/other)	Neisseria meningitidis	B	B	B	19	1	1	83	4	4	10	2	5	38	11	1		1159	4	4	2
20426	M11 240391	UK [England]	2011	invasive (unspecified/other)	Neisseria meningitidis	B	B	B	4	2	21	84	2	3	4	9	5	9	6	2	ST-41/44 complex	9824	2	3	1
20427	M11 240392	UK [England]	2011	invasive (unspecified/other)	Neisseria meningitidis	B	B	B	1	6	3	100	7	8	3	5	4	1	3	8	ST-32 complex	290	7	1	1
20428	M11 240394	UK [England]	2011	invasive (unspecified/other)	Neisseria meningitidis	B	B	B	13	3	1	145	3	17	5	19	3	3	26	2	ST-60 complex	5103	3	3	1
20429	M11 240395	UK [England]	2011	invasive (unspecified/other)	Neisseria meningitidis	B	B	B	152	39	1	113	48	4	10	2	5	3	11	9	ST-269 complex	1163	NA	NA	3
20430	M11 240396	UK [England]	2011	invasive (unspecified/other)	Neisseria meningitidis	Y	Y	Y	25	1	1	26	4	12	5	18	9	11	9	17	ST-23 complex	1655	4	4	2
20431	M11 240397	UK [England]	2011	invasive (unspecified/other)	Neisseria meningitidis	B	B	B	13	3	57	145	3	4	10	34	5	38	11	9	ST-269 complex	1161	3	3	1
20432	M11 240398	UK [Wales]	2011	invasive (unspecified/other)	Neisseria meningitidis	B	B	B	4	2	1	84	2	3	6	9	5	9	6	9	ST-41/44 complex	41	2	3	1
20433	M11 240399	UK [England]	2011	invasive (unspecified/other)	Neisseria meningitidis	Y	Y	Y	25	1	1	26	4	12	5	18	9	11	9	17	ST-23 complex	1655	4	4	2
20434	M11 240401	UK [England]	2011	invasive (unspecified/other)	Neisseria meningitidis	B	B	B	13	3	1	145	3	4	10	34	5	38	11	9	ST-269 complex	1161	3	3	1
20435	M11 240402	UK [England]	2011	invasive (unspecified/other)	Neisseria meningitidis	B	B	B	0	NA	1	0	NA	43	5	9	60	5	19	9		9825	NA	NA	NA
20436	M11 240403	UK [England]	2011	invasive (unspecified/other)	Neisseria meningitidis	W	W	W	22	1	1	1	4	2	3	4	3	8	4	6	ST-11 complex	11	4	4	2
20437	M11 240405	UK [England]	2011	invasive (unspecified/other)	Neisseria meningitidis	B	B	B	13	3	57	145	3	4	10	34	5	38	11	9	ST-269 complex	1161	3	3	1
20438	M11 240406	UK [England]	2011	invasive (unspecified/other)	Neisseria meningitidis	B	B	B	1	6	7	100	7	4	10	5	4	6	3	8	ST-32 complex	32	7	1	1
20439	M11 240409	UK [England]	2011	invasive (unspecified/other)	Neisseria meningitidis	B	B	B	15	10	2	193	17	4	10	15	9	8	11	9	ST-269 complex	269	NA	NA	1
20440	M11 240411	UK [England]	2011	invasive (unspecified/other)	Neisseria meningitidis	Y	Y	Y	25	1	1	26	4	10	5	18	9	11	9	12	ST-23 complex	4183	4	4	2
20441	M11 240412	UK [England]	2011	invasive (unspecified/other)	Neisseria meningitidis	B	B	B	13	3	1	145	3	17	5	47	17	3	26	2	ST-60 complex	1430	3	3	1
20442	M11 240413	UK [England]	2011	invasive (unspecified/other)	Neisseria meningitidis	B	B	B	13	3	57	145	3	4	10	9	9	38	11	9	ST-269 complex	9826	3	3	1
20443	M11 240414	UK [England]	2011	invasive (unspecified/other)	Neisseria meningitidis	B	B	B	14	1	1	142	13	3	6	108	5	9	25	9	ST-41/44 complex	5551	13	3	1
20444	M11 240417	UK [England]	2011	invasive (unspecified/other)	Neisseria meningitidis	W	W	W	22	1	1	1	4	2	3	4	3	8	4	6	ST-11 complex	11	4	4	2
20445	M11 240420	UK [England]	2011	invasive (unspecified/other)	Neisseria meningitidis	B	B	B	4	2	1	84	2	3	6	9	5	9	6	9	ST-41/44 complex	41	2	3	1
20446	M11 240422	UK [England]	2011	invasive (unspecified/other)	Neisseria meningitidis	B	B	B	15	4	2	193	1	4	10	15	9	8	11	13	ST-269 complex	467	1	1	1
20447	M11 240424	UK [England]	2011	invasive (unspecified/other)	Neisseria meningitidis	B	B	B	24	1	63	25	4	5	6	9	3	9	6	16	ST-41/44 complex	3101	4	4	2
20448	M11 240425	UK [England]	2011	invasive (unspecified/other)	Neisseria meningitidis	B	B	B	4	2	1	84	2	3	6	9	5	9	6	9	ST-41/44 complex	41	2	3	1
20449	M11 240427	UK [England]	2011	invasive (unspecified/other)	Neisseria meningitidis	W	W	W	22	1	1	1	4	2	3	4	3	8	4	6	ST-11 complex	11	4	4	2
20450	M11 240428	UK [England]	2011	invasive (unspecified/other)	Neisseria meningitidis	B	B	B	13	3	57	145	3	4	10	2	5	38	11	9	ST-269 complex	275	3	3	1
20451	M11 240430	UK [England]	2011	invasive (unspecified/other)	Neisseria meningitidis	Y	Y	Y	293	23	1	114	49	10	5	18	9	11	9	17	ST-23 complex	23	NA	NA	3
20452	M11 240431	UK [England]	2011	invasive (unspecified/other)	Neisseria meningitidis	B	B	B	19	40	1	83	50	4	6	63	244	9	11	9		9827	NA	NA	2
20453	M11 240434	UK [England]	2011	invasive (unspecified/other)	Neisseria meningitidis	B	B	B	4	2	1	84	2	3	6	34	5	11	6	9	ST-41/44 complex	1194	2	3	1
20454	M11 240435	UK [England]	2011	invasive (unspecified/other)	Neisseria meningitidis	Y	Y	Y	25	1	1	26	4	12	5	18	9	11	9	17	ST-23 complex	1655	4	4	2
20455	M11 240436	UK [England]	2011	invasive (unspecified/other)	Neisseria meningitidis	B	B	B	13	3	57	145	3	4	10	34	5	38	11	9	ST-269 complex	1161	3	3	1
20456	M11 240437	UK [Northern Ireland]	2011	invasive (unspecified/other)	Neisseria meningitidis	Y	Y	Y	21	1	1	7	4	3	7	4	37	8	18	120	ST-92 complex	784	4	4	2
20457	M11 240440	UK [England]	2011	invasive (unspecified/other)	Neisseria meningitidis	B	B	B	13	3	1	145	3	17	5	19	17	3	26	2	ST-60 complex	60	3	3	1
20458	M11 240441	UK [England]	2011	invasive (unspecified/other)	Neisseria meningitidis	W	W	W	421	41	1	12	51	6	148	15	17	5	24	17	ST-174 complex	2977	NA	NA	2
20459	M11 240442	UK [England]	2011	invasive (unspecified/other)	Neisseria meningitidis	Y	Y	Y	25	1	1	26	4	10	5	18	9	11	9	17	ST-23 complex	23	4	4	2
21092	M11 240443	UK [England]	2011	invasive (unspecified/other)	Neisseria meningitidis	B	B	B	90	72	85	242	52	12	5	12	35	60	22	17	ST-461 complex	461	NA	NA	1
21093	M11 240445	UK [England]	2011	invasive (unspecified/other)	Neisseria meningitidis	B	B	B	13	3	57	145	3	4	10	34	5	38	11	9	ST-269 complex	1161	3	3	1
21094	M11 240446	UK [England]	2011	invasive (unspecified/other)	Neisseria meningitidis	Y	Y	Y	25	1	1	26	4	12	5	18	9	11	9	17	ST-23 complex	1655	4	4	2
21095	M11 240447	UK [England]	2011	invasive (unspecified/other)	Neisseria meningitidis	B	B	B	14	7	1	142	6	3	116	9	5	9	22	9	ST-41/44 complex	1960	6	2	1
21096	M11 240448	UK [Wales]	2011	invasive (unspecified/other)	Neisseria meningitidis	B	B	B	1	6	3	100	7	8	10	5	4	6	3	15	ST-32 complex	8049	7	1	1
21097	M11 240450	UK [England]	2011	invasive (unspecified/other)	Neisseria meningitidis	B	B	B	13	3	57	145	3	4	10	34	5	38	11	9	ST-269 complex	1161	3	3	1
21098	M11 240451	UK [England]	2011	invasive (unspecified/other)	Neisseria meningitidis	B	B	B	4	2	1	84	2	3	6	34	5	11	6	9	ST-41/44 complex	1194	2	3	1
21099	M11 240452	UK [England]	2011	invasive (unspecified/other)	Neisseria meningitidis	B	B	B	45	42	77	87	53	7	5	1	13	36	53	15	ST-213 complex	213	NA	NA	3
21100	M11 240453	UK [England]	2011	invasive (unspecified/other)	Neisseria meningitidis	B	B	B	19	1	1	83	4	4	10	2	5	38	11	9	ST-269 complex	275	4	4	2
21101	M11 240456	UK [England]	2011	invasive (unspecified/other)	Neisseria meningitidis	B	B	B	645	29	5	120	54	7	5	1	17	36	53	15	ST-213 complex	2391	NA	NA	3
21102	M11 240457	UK [England]	2011	invasive (unspecified/other)	Neisseria meningitidis	B	B	B	302	24	1	121	55	9	20	9	9	9	6	9	ST-41/44 complex	9200	NA	NA	2
21103	M11 240458	UK [England]	2011	invasive (unspecified/other)	Neisseria meningitidis	B	B	B	456	73	1	243	56	4	10	2	5	3	11	9	ST-269 complex	1163	NA	NA	1
21104	M11 240459	UK [Wales]	2011	invasive (unspecified/other)	Neisseria meningitidis	B	B	B	19	1	1	83	4	4	10	2	5	11	11	9	ST-269 complex	6428	4	4	2
21105	M11 240461	UK [England]	2011	invasive (unspecified/other)	Neisseria meningitidis	Y	Y	Y	25	1	1	26	4	10	5	18	9	11	9	17	ST-23 complex	23	4	4	2
21106	M11 240463	UK [England]	2011	invasive (unspecified/other)	Neisseria meningitidis	B	B	B	59	9	57	75	16	4	10	34	5	38	11	9	ST-269 complex	1161	16	2	3
21107	M11 240465	UK [England]	2011	invasive (unspecified/other)	Neisseria meningitidis	Y	Y	Y	25	1	1	26	4	12	5	18	9	11	9	17	ST-23 complex	1655	4	4	2
21108	M11 240466	UK [England]	2011	invasive (unspecified/other)	Neisseria meningitidis	Y	Y	Y	293	23	1	114	49	10	5	18	9	521	9	17	ST-23 complex	8096	NA	NA	3
21109	M11 240467	UK [England]	2011	invasive (unspecified/other)	Neisseria meningitidis	B	B	B	309	1	1	244	4	1	5	13	53	26	41	3	ST-162 complex	162	4	3	1
21110	M11 240469	UK [England]	2011	invasive (unspecified/other)	Neisseria meningitidis	B	B	B	100	74	5	191	57	2	3	4	3	8	4	683	ST-11 complex	10260	NA	NA	1
21111	M11 240470	UK [England]	2011	invasive (unspecified/other)	Neisseria meningitidis	B	B	B	646	15	77	122	58	7	5	1	13	36	53	15	ST-213 complex	213	NA	NA	3
21112	M11 240471	UK [England]	2011	invasive (unspecified/other)	Neisseria meningitidis	B	B	B	13	3	1	145	3	17	5	19	17	3	26	2	ST-60 complex	60	3	3	1
21113	M11 240472	UK [England]	2011	invasive (unspecified/other)	Neisseria meningitidis	B	B	B	24	1	1	25	4	27	6	9	10	9	6	16	ST-41/44 complex	2009	4	4	2
21114	M11 240473	UK [England]	2011	invasive (unspecified/other)	Neisseria meningitidis	B	B	B	4	2	1	84	2	3	6	9	5	8	6	9	ST-41/44 complex	485	2	3	1
21115	M11 240474	UK [Northern Ireland]	2011	invasive (unspecified/other)	Neisseria meningitidis	C	C	C	647	43	1	123	59	206	6	470	13	249	6	9		10261	NA	NA	3
21116	M11 240475	UK [England]	2011	invasive (unspecified/other)	Neisseria meningitidis	B	B	B	4	2	1	84	2	6	3	6	17	26	21	8		4051	2	3	1
21117	M11 240476	UK [England]	2011	invasive (unspecified/other)	Neisseria meningitidis	B	B	B	19	1	1	65	4	9	20	9	9	9	6	2	ST-41/44 complex	1097	4	4	2
21118	M11 240477	UK [England]	2011	invasive (unspecified/other)	Neisseria meningitidis	B	B	B	15	4	2	193	1	4	10	15	9	8	11	13	ST-269 complex	467	1	1	1
21119	M11 240479	UK [England]	2011	invasive (unspecified/other)	Neisseria meningitidis	B	B	B	21	1	1	7	4	1	5	13	53	26	41	3	ST-162 complex	162	4	4	2
21120	M11 240480	UK [England]	2011	invasive (unspecified/other)	Neisseria meningitidis	B	B	B	15	4	2	193	1	4	10	15	9	8	11	9	ST-269 complex	269	1	1	1
21121	M11 240484	UK [England]	2011	invasive (unspecified/other)	Neisseria meningitidis	B	B	B	215	5	5	233	60	4	10	2	5	3	11	9	ST-269 complex	1163	NA	NA	1
21122	M11 240485	UK [England]	2011	invasive (unspecified/other)	Neisseria meningitidis	B	B	B	4	2	57	84	2	3	6	9	25	9	6	9	ST-41/44 complex	2799	2	3	1
21123	M11 240486	UK [England]	2011	invasive (unspecified/other)	Neisseria meningitidis	W	W	W	22	1	1	1	4	2	3	4	3	21	4	6	ST-11 complex	1860	4	4	2
21124	M11 240487	UK [England]	2011	invasive (unspecified/other)	Neisseria meningitidis	B	B	B	16	1	1	59	4	12	5	2	24	5	662	3		10262	4	4	2
21125	M11 240488	UK [England]	2011	invasive (unspecified/other)	Neisseria meningitidis	B	B	B	19	1	57	93	13	8	5	15	56	9	21	2	ST-865 complex	4237	13	4	2
21126	M11 240489	UK [England]	2011	invasive (unspecified/other)	Neisseria meningitidis	B	B	B	19	1	1	65	4	7	5	1	13	36	53	15	ST-213 complex	213	4	4	2
21127	M11 240491	UK [England]	2011	invasive (unspecified/other)	Neisseria meningitidis	B	B	B	4	2	1	84	2	3	6	9	5	9	6	46	ST-41/44 complex	3050	2	3	1
21128	M11 240492	UK [England]	2011	invasive (unspecified/other)	Neisseria meningitidis	B	B	B	4	2	1	84	2	3	6	9	5	36	6	9	ST-41/44 complex	9822	2	3	1
21129	M11 240493	UK [England]	2011	invasive (unspecified/other)	Neisseria meningitidis	B	B	B	4	2	1	84	2	3	6	9	5	11	6	9	ST-41/44 complex	154	2	3	1
21130	M11 240494	UK [England]	2011	invasive (unspecified/other)	Neisseria meningitidis	Y	Y	Y	25	1	1	26	4	12	5	18	9	11	9	17	ST-23 complex	1655	4	4	2
21131	M11 240495	UK [England]	2011	invasive (unspecified/other)	Neisseria meningitidis	B	B	B	1	6	3	100	7	4	10	5	40	6	3	8	ST-32 complex	259	7	1	1
21132	M11 240497	UK [England]	2011	invasive (unspecified/other)	Neisseria meningitidis	W	W	W	16	1	1	60	4	11	5	18	154	11	24	21	ST-22 complex	1281	4	4	2
21133	M11 240501	UK [England]	2011	invasive (unspecified/other)	Neisseria meningitidis	B	B	B	19	1	1	83	4	4	10	2	5	3	11	12		10263	4	4	2
21134	M11 240502	UK [England]	2011	invasive (unspecified/other)	Neisseria meningitidis	C	C	C	78	5	1	245	11	2	3	4	3	8	4	6	ST-11 complex	11	11	2	1
21137	M11 240506	UK [England]	2011	invasive (unspecified/other)	Neisseria meningitidis	B	B	B	86	4	2	222	1	3	6	19	7	3	6	5		8988	1	1	1
21138	M11 240507	UK [England]	2011	invasive (unspecified/other)	Neisseria meningitidis	Y	Y	Y	25	1	1	26	4	12	5	18	9	11	9	17	ST-23 complex	1655	4	4	2
21139	M11 240508	UK [England]	2011	invasive (unspecified/other)	Neisseria meningitidis	W	W	W	16	1	1	60	4	11	5	18	8	11	4	21	ST-22 complex	184	4	4	2
21140	M11 240509	UK [England]	2011	invasive (unspecified/other)	Neisseria meningitidis	B	B	B	19	1	1	83	4	4	10	2	5	3	11	9	ST-269 complex	1163	4	4	2
21141	M11 240510	UK [England]	2011	invasive (unspecified/other)	Neisseria meningitidis	B	B	B	215	5	77	233	60	7	5	1	13	36	53	15	ST-213 complex	213	NA	NA	1
21142	M11 240592	UK [England]	2011	invasive (unspecified/other)	Neisseria meningitidis	B	B	B	4	2	1	84	2	3	6	9	5	9	6	9	ST-41/44 complex	41	2	3	1
21143	M11 240593	UK [England]	2011	invasive (unspecified/other)	Neisseria meningitidis	B	B	B	15	4	2	193	1	4	10	15	9	8	42	13	ST-269 complex	10264	1	1	1
21144	M11 240594	UK [England]	2011	invasive (unspecified/other)	Neisseria meningitidis	B	B	B	4	2	1	84	2	3	6	9	5	9	6	9	ST-41/44 complex	41	2	3	1
21145	M11 240595	UK [England]	2011	invasive (unspecified/other)	Neisseria meningitidis	B	B	B	45	75	22	87	61	7	5	1	13	36	53	15	ST-213 complex	213	NA	NA	3
21146	M11 240597	UK [England]	2011	invasive (unspecified/other)	Neisseria meningitidis	B	B	B	13	3	57	145	3	4	10	34	5	38	11	9	ST-269 complex	1161	3	3	1
21147	M11 240598	UK [England]	2011	invasive (unspecified/other)	Neisseria meningitidis	B	B	B	45	8	77	87	20	7	5	1	13	36	53	15	ST-213 complex	213	20	4	3
21148	M11 240599	UK [England]	2011	invasive (unspecified/other)	Neisseria meningitidis	Y	Y	Y	25	1	1	26	4	12	5	18	9	11	9	17	ST-23 complex	1655	4	4	2
21151	M11 240657	UK [England]	2011	invasive (unspecified/other)	Neisseria meningitidis	Y	Y	Y	25	1	1	26	4	10	5	18	9	11	9	17	ST-23 complex	23	4	4	2
21152	M11 240707	UK [England]	2011	invasive (unspecified/other)	Neisseria meningitidis	B	B	B	19	1	1	65	4	9	20	9	9	9	6	2	ST-41/44 complex	1097	4	4	2
21153	M11 240710	UK [England]	2011	invasive (unspecified/other)	Neisseria meningitidis	Y	Y	Y	25	1	1	26	4	12	5	18	9	11	9	17	ST-23 complex	1655	4	4	2
21154	M11 240712	UK [Wales]	2011	invasive (unspecified/other)	Neisseria meningitidis	B	B	B	19	1	1	65	4	9	6	9	11	9	6	9	ST-41/44 complex	5906	4	4	2
21155	M11 240713	UK [England]	2011	invasive (unspecified/other)	Neisseria meningitidis	B	B	B	13	3	57	145	3	4	10	34	5	38	11	3		6781	3	3	1
21156	M11 240716	UK [England]	2011	invasive (unspecified/other)	Neisseria meningitidis	W	W	W	16	1	1	60	4	11	5	18	17	11	4	21	ST-22 complex	3795	4	4	2
21157	M11 240717	UK [Northern Ireland]	2011	invasive (unspecified/other)	Neisseria meningitidis	Y	Y	Y	25	1	1	26	4	12	5	18	9	11	9	17	ST-23 complex	1655	4	4	2
21158	M11 240719	UK [England]	2011	invasive (unspecified/other)	Neisseria meningitidis	Y	Y	Y	25	1	1	26	4	10	5	314	9	11	9	17	ST-23 complex	3582	4	4	2
21159	M11 240721	UK [England]	2011	invasive (unspecified/other)	Neisseria meningitidis	B	B	B	4	2	1	84	2	3	6	9	5	9	6	9	ST-41/44 complex	41	2	3	1
21160	M11 240723	UK [England]	2011	invasive (unspecified/other)	Neisseria meningitidis	B	B	B	14	1	57	142	13	6	5	12	12	12	13	14		1345	13	3	1
21161	M11 240724	UK [England]	2011	invasive (unspecified/other)	Neisseria meningitidis	B	B	B	4	2	79	84	2	3	206	9	5	9	6	9	ST-41/44 complex	5357	2	3	1
21162	M11 240725	UK [England]	2011	invasive (unspecified/other)	Neisseria meningitidis	B	B	B	15	4	2	193	1	4	10	2	5	38	11	9	ST-269 complex	275	1	1	1
21163	M11 240726	UK [England]	2011	invasive (unspecified/other)	Neisseria meningitidis	W	W	W	22	1	1	1	4	2	3	4	3	8	4	6	ST-11 complex	11	4	4	2
21164	M11 240727	UK [England]	2011	invasive (unspecified/other)	Neisseria meningitidis	B	B	B	102	8	10	2	20	7	5	1	13	36	53	15	ST-213 complex	213	20	4	2
21165	M11 240728	UK [England]	2011	invasive (unspecified/other)	Neisseria meningitidis	B	B	B	8	21	1	218	19	9	20	9	629	9	6	2	ST-41/44 complex	9889	NA	NA	1
21167	M11 240731	UK [England]	2011	invasive (unspecified/other)	Neisseria meningitidis	Y	Y	Y	25	1	1	26	4	12	5	18	9	11	9	17	ST-23 complex	1655	4	4	2
21168	M11 240733	UK [England]	2011	invasive (unspecified/other)	Neisseria meningitidis	B	B	B	13	3	57	145	3	4	10	34	5	38	11	9	ST-269 complex	1161	3	3	1
21169	M11 240734	UK [England]	2011	invasive (unspecified/other)	Neisseria meningitidis	Y	Y	Y	25	1	1	26	4	10	5	18	9	11	9	17	ST-23 complex	23	4	4	2
21170	M11 240735	UK [England]	2011	invasive (unspecified/other)	Neisseria meningitidis	B	B	B	13	3	57	145	3	4	10	34	13	38	11	9	ST-269 complex	6604	3	3	1
21171	M11 240736	UK [England]	2011	invasive (unspecified/other)	Neisseria meningitidis	B	B	B	19	1	1	65	4	9	20	9	9	9	6	2	ST-41/44 complex	1097	4	4	2
21172	M11 240737	UK [Wales]	2011	invasive (unspecified/other)	Neisseria meningitidis	Y	Y	Y	25	1	1	26	4	12	5	18	9	11	9	17	ST-23 complex	1655	4	4	2
21173	M11 240738	UK [England]	2011	invasive (unspecified/other)	Neisseria meningitidis	B	B	B	648	9	4	246	16	7	5	1	13	36	53	15	ST-213 complex	213	16	2	1
21174	M11 240740	UK [England]	2011	invasive (unspecified/other)	Neisseria meningitidis	B	B	B	31	9	1	124	16	4	10	2	17	38	11	1		10265	16	2	3
21175	M11 240741	UK [England]	2011	invasive (unspecified/other)	Neisseria meningitidis	W	W	W	16	22	1	97	32	11	5	168	8	11	4	21	ST-22 complex	1286	NA	NA	2
21176	M11 240742	UK [England]	2011	invasive (unspecified/other)	Neisseria meningitidis	B	B	B	599	4	4	92	1	4	10	11	18	6	10	12	ST-35 complex	35	1	1	2
21177	M11 240743	UK [England]	2011	invasive (unspecified/other)	Neisseria meningitidis	B	B	B	21	1	1	7	4	1	5	13	53	26	41	3	ST-162 complex	162	4	4	2
21178	M11 240745	UK [England]	2011	invasive (unspecified/other)	Neisseria meningitidis	Y	Y	Y	25	1	1	26	4	10	339	18	9	11	9	17	ST-23 complex	8411	4	4	2
21179	M11 240749	UK [England]	2011	invasive (unspecified/other)	Neisseria meningitidis	B	B	B	1	6	3	100	7	8	10	77	4	6	3	8	ST-32 complex	749	7	1	1

21180	M11 240750	UK [England]	2011	invasive (unspecified/other)	Neisseria meningitidis	B	B	B	15	4	2	193	1	4	10	15	9	8	26	9	ST-269 complex	1942	1	1	1
21181	M11 240762	UK [England]	2011	invasive (unspecified/other)	Neisseria meningitidis	B	B	B	19	1	1	83	4	4	10	2	5	38	11	9	ST-269 complex	275	4	4	2
21182	M11 240763	UK [England]	2011	invasive (unspecified/other)	Neisseria meningitidis	B	B	B	16	1	4	59	4	132	3	19	9	216	21	2		10266	4	4	2
21183	M11 240765	UK [England]	2011	invasive (unspecified/other)	Neisseria meningitidis	Y	Y	Y	25	1	1	26	4	12	5	18	9	11	9	17	ST-23 complex	1655	4	4	2
21184	M11 240766	UK [England]	2011	invasive (unspecified/other)	Neisseria meningitidis	B	B	B	14	7	1	142	6	3	6	9	5	26	6	9	ST-41/44 complex	8054	6	2	1
21186	M11 240771	UK [England]	2011	invasive (unspecified/other)	Neisseria meningitidis	B	B	B	37	1	1	408	4	7	8	10	19	10	1	2	ST-18 complex	18	4	3	1
21187	M11 240772	UK [England]	2011	invasive (unspecified/other)	Neisseria meningitidis	Y	Y	Y	25	1	1	26	4	12	5	18	9	11	9	17	ST-23 complex	1655	4	4	2
21188	M11 240773	UK [England]	2011	invasive (unspecified/other)	Neisseria meningitidis	B	B	B	14	7	1	142	6	3	6	9	5	9	6	9	ST-41/44 complex	41	6	2	1
21189	M11 240774	UK [England]	2011	invasive (unspecified/other)	Neisseria meningitidis	B	B	B	15	4	2	193	1	4	10	15	9	8	11	9	ST-269 complex	269	1	1	1
21190	M11 240775	UK [England]	2011	invasive (unspecified/other)	Neisseria meningitidis	B	B	B	15	4	2	193	1	4	10	15	9	8	11	17	ST-269 complex	283	1	1	1
21191	M11 240776	UK [England]	2011	invasive (unspecified/other)	Neisseria meningitidis	B	B	B	13	3	57	145	3	4	10	34	5	38	11	9	ST-269 complex	1161	3	3	1
21192	M11 240779	UK [England]	2011	invasive (unspecified/other)	Neisseria meningitidis	Y	Y	Y	25	1	1	26	4	12	286	18	9	11	9	17	ST-23 complex	6463	4	4	2
21193	M11 240780	UK [England]	2011	invasive (unspecified/other)	Neisseria meningitidis	W/Y	W/Y	W/Y	25	1	1	26	4	8	5	18	17	11	24	21	ST-22 complex	3651	4	4	2
21194	M11 240781	UK [England]	2011	invasive (unspecified/other)	Neisseria meningitidis	B	B	B	15	4	2	193	1	4	10	15	9	8	11	9	ST-269 complex	269	1	1	1
21195	M11 240783	UK [Northern Ireland]	2011	invasive (unspecified/other)	Neisseria meningitidis	B	B	B	4	2	1	84	2	10	6	34	5	11	6	9	ST-41/44 complex	4496	2	3	1
21196	M11 240785	UK [England]	2011	invasive (unspecified/other)	Neisseria meningitidis	C	C	C	22	1	1	1	4	2	3	4	3	8	4	6	ST-11 complex	11	4	4	2
21197	M11 240787	UK [England]	2011	invasive (unspecified/other)	Neisseria meningitidis	B	B	B	649	1	59	247	4	20	5	75	58	1	21	20		939	4	3	1
21198	M11 240788	UK [England]	2011	invasive (unspecified/other)	Neisseria meningitidis	Y	Y	Y	25	1	1	26	4	12	5	18	9	11	9	17	ST-23 complex	1655	4	4	2
21199	M11 240789	UK [England]	2011	invasive (unspecified/other)	Neisseria meningitidis	B	B	B	25	1	1	26	4	8	4	6	17	5	18	2	ST-103 complex	103	4	4	2
21200	M11 240790	UK [England]	2011	invasive (unspecified/other)	Neisseria meningitidis	B	B	B	147	44	13	36	62	27	6	9	3	9	6	16	ST-41/44 complex	136	NA	NA	2
21201	M11 240793	UK [England]	2011	invasive (unspecified/other)	Neisseria meningitidis	Y	Y	Y	25	1	1	26	4	10	5	18	9	11	9	17	ST-23 complex	23	4	4	2
21202	M11 240796	UK [England]	2011	invasive (unspecified/other)	Neisseria meningitidis	B	B	B	15	10	2	193	17	4	10	15	9	8	11	9	ST-269 complex	269	NA	NA	1
21203	M11 240798	UK [Wales]	2011	invasive (unspecified/other)	Neisseria meningitidis	W	W	W	22	1	1	1	4	2	3	4	3	8	4	6	ST-11 complex	11	4	4	2
21204	M11 240799	UK [England]	2011	invasive (unspecified/other)	Neisseria meningitidis	B	B	B	1	6	3	100	7	8	10	5	4	6	3	8	ST-32 complex	33	7	1	1
21205	M11 240801	UK [England]	2011	invasive (unspecified/other)	Neisseria meningitidis	Y	Y	Y	25	1	1	26	4	10	5	18	9	11	9	17	ST-23 complex	23	4	4	2
21206	M11 240802	UK [England]	2011	invasive (unspecified/other)	Neisseria meningitidis	W	W	W	22	1	1	1	4	2	3	4	3	8	4	6	ST-11 complex	11	4	4	2
21207	M11 240803	UK [Northern Ireland]	2011	invasive (unspecified/other)	Neisseria meningitidis	B	B	B	19	1	1	83	4	4	10	2	5	3	11	9	ST-269 complex	1163	4	4	2
21208	M11 240941	UK [England]	2011	invasive (unspecified/other)	Neisseria meningitidis	C	C	C	13	16	57	145	63	2	3	4	3	8	4	6	ST-11 complex	11	NA	NA	1
21209	M11 240942	UK [England]	2011	invasive (unspecified/other)	Neisseria meningitidis	B	B	B	19	1	1	83	4	4	10	2	5	38	11	9	ST-269 complex	275	4	4	2
21210	M11 240945	UK [England]	2011	invasive (unspecified/other)	Neisseria meningitidis	B	B	B	14	1	1	142	13	3	6	19	5	3	6	9	ST-41/44 complex	340	13	3	1
21211	M11 240946	UK [England]	2011	invasive (unspecified/other)	Neisseria meningitidis	B	B	B	4	1	1	206	4	4	10	34	5	38	11	9	ST-269 complex	1161	4	3	1
21212	M11 240948	UK [England]	2011	invasive (unspecified/other)	Neisseria meningitidis	B	B	B	45	8	77	87	20	7	5	1	13	36	53	15	ST-213 complex	213	20	4	3
21213	M11 240949	UK [England]	2011	invasive (unspecified/other)	Neisseria meningitidis	B	B	B	4	2	1	84	2	3	6	9	5	9	6	9	ST-41/44 complex	41	2	3	1
21214	M11 240953	UK [Wales]	2011	invasive (unspecified/other)	Neisseria meningitidis	W	W	W	22	1	1	1	4	2	3	4	3	8	4	6	ST-11 complex	11	4	4	2
21215	M11 240954	UK [England]	2011	invasive (unspecified/other)	Neisseria meningitidis	B	B	B	13	3	57	145	3	4	10	34	5	38	11	9	ST-269 complex	1161	3	3	1
21216	M11 240975	UK [England]	2011	invasive (unspecified/other)	Neisseria meningitidis	W	W	W	22	1	1	1	4	2	3	4	3	8	4	6	ST-11 complex	11	4	4	2
21217	M11 240976	UK [England]	2011	invasive (unspecified/other)	Neisseria meningitidis	B	B	B	45	14	77	87	45	7	5	1	13	36	53	15	ST-213 complex	213	NA	NA	3
21218	M11 240977	UK [England]	2011	invasive (unspecified/other)	Neisseria meningitidis	B	B	B	45	8	77	87	20	7	5	1	NA	36	53	15		NA	20	4	3
21219	M11 240978	UK [England]	2011	invasive (unspecified/other)	Neisseria meningitidis	B	B	B	19	1	1	65	4	4	10	15	7	8	11	1	ST-269 complex	13	4	4	2
21220	M11 240979	UK [England]	2011	invasive (unspecified/other)	Neisseria meningitidis	B	B	B	13	3	57	145	3	4	10	2	5	38	11	9	ST-269 complex	275	3	3	1
21221	M11 240980	UK [Wales]	2011	invasive (unspecified/other)	Neisseria meningitidis	B	B	B	45	8	NA	87	20	7	5	1	17	180	53	15	ST-213 complex	10267	20	4	3
21222	M11 240981	UK [England]	2011	invasive (unspecified/other)	Neisseria meningitidis	B	B	B	4	2	1	84	2	3	6	9	5	9	6	9	ST-41/44 complex	41	2	3	1
21223	M11 240982	UK [Northern Ireland]	2011	invasive (unspecified/other)	Neisseria meningitidis	Y	Y	Y	25	1	1	26	4	12	5	18	9	11	9	17	ST-23 complex	1655	4	4	2
21224	M11 240983	UK [England]	2011	invasive (unspecified/other)	Neisseria meningitidis	B	B	B	14	1	57	142	13	4	10	2	5	38	11	1		1159	13	3	1
21225	M11 240984	UK [England]	2011	invasive (unspecified/other)	Neisseria meningitidis	B	B	B	69	45	1	248	64	4	10	5	4	6	3	8	ST-32 complex	32	NA	NA	1
21226	M11 240986	UK [England]	2011	invasive (unspecified/other)	Neisseria meningitidis	B	B	B	13	3	57	145	3	4	10	34	5	38	11	9	ST-269 complex	1161	3	3	1
21227	M11 240987	UK [England]	2011	invasive (unspecified/other)	Neisseria meningitidis	Y	Y	Y	25	1	1	26	4	10	5	18	9	11	9	17	ST-23 complex	23	4	4	2
21228	M11 240988	UK [England]	2011	invasive (unspecified/other)	Neisseria meningitidis	B	B	B	47	1	1	90	15	12	5	12	35	60	22	17	ST-461 complex	461	15	4	3
21229	M11 240991	UK [England]	2011	invasive (unspecified/other)	Neisseria meningitidis	Y	Y	Y	25	1	1	26	4	12	5	18	9	11	9	17	ST-23 complex	1655	4	4	2
21230	M11 240992	UK [England]	2011	invasive (unspecified/other)	Neisseria meningitidis	B	B	B	4	2	1	84	2	3	6	34	5	11	6	9	ST-41/44 complex	1194	2	3	1
21231	M11 240993	UK [England]	2011	invasive (unspecified/other)	Neisseria meningitidis	B	B	B	15	4	2	193	1	4	10	15	9	8	20	9	ST-269 complex	7226	1	1	1
21232	M11 240994	UK [England]	2011	invasive (unspecified/other)	Neisseria meningitidis	C	C	C	650	5	1	249	65	2	3	4	3	8	4	6	ST-11 complex	11	NA	NA	1
21233	M11 240995	UK [England]	2011	invasive (unspecified/other)	Neisseria meningitidis	B	B	B	13	3	57	145	3	4	10	34	5	38	11	9	ST-269 complex	1161	3	3	1
21234	M11 241013	UK [England]	2011	invasive (unspecified/other)	Neisseria meningitidis	B	B	B	651	76	1	125	66	3	6	9	5	9	6	9	ST-41/44 complex	41	NA	NA	3
21235	M11 241014	UK [England]	2011	invasive (unspecified/other)	Neisseria meningitidis	B	B	B	4	2	79	84	2	3	6	9	5	9	6	9	ST-41/44 complex	41	2	3	1
21236	M11 241015	UK [England]	2011	invasive (unspecified/other)	Neisseria meningitidis	B	B	B	4	2	1	84	2	3	6	9	5	9	6	9	ST-41/44 complex	41	2	3	1
21237	M11 241016	UK [England]	2011	invasive (unspecified/other)	Neisseria meningitidis	B	B	B	0	5	1	917	11	2	3	4	3	8	4	6	ST-11 complex	11	11	2	NA
21238	M11 241018	UK [Wales]	2011	invasive (unspecified/other)	Neisseria meningitidis	B	B	B	4	2	1	84	2	3	6	34	5	11	6	9	ST-41/44 complex	1194	2	3	1
21239	M11 241019	UK [England]	2011	invasive (unspecified/other)	Neisseria meningitidis	B	B	B	1	77	3	100	67	8	10	5	4	6	3	8	ST-32 complex	33	NA	NA	1
21240	M11 241023	UK [England]	2011	invasive (unspecified/other)	Neisseria meningitidis	B	B	B	539	1	1	421	4	7	8	10	19	5	1	2	ST-18 complex	5529	4	3	1
21241	M11 241024	UK [England]	2011	invasive (unspecified/other)	Neisseria meningitidis	B	B	B	15	4	NA	193	1	4	10	15	9	8	11	9	ST-269 complex	269	1	1	1
21242	M11 241025	UK [England]	2011	invasive (unspecified/other)	Neisseria meningitidis	B	B	B	24	1	4	27	4	4	10	72	9	3	10	12	ST-35 complex	6605	4	4	2
21243	M11 241026	UK [England]	2011	invasive (unspecified/other)	Neisseria meningitidis	B	B	B	13	3	57	209	3	626	25	1	17	21	26	49	ST-1157 complex	10268	3	3	1
21244	M11 241027	UK [England]	2011	invasive (unspecified/other)	Neisseria meningitidis	B	B	B	13	3	57	145	3	4	10	34	5	38	11	9	ST-269 complex	1161	3	3	1
21245	M11 241028	UK [England]	2011	invasive (unspecified/other)	Neisseria meningitidis	B	B	B	4	2	1	84	2	3	6	9	5	9	6	9	ST-41/44 complex	41	2	3	1
21246	M11 241031	UK [England]	2011	invasive (unspecified/other)	Neisseria meningitidis	B	B	B	47	1	1	90	15	12	5	12	35	60	22	17	ST-461 complex	461	15	4	3
21247	M11 241032	UK [England]	2011	invasive (unspecified/other)	Neisseria meningitidis	B	B	B	4	2	1	84	2	3	6	9	5	8	6	9	ST-41/44 complex	485	2	3	1
21248	M11 241033	UK [England]	2011	invasive (unspecified/other)	Neisseria meningitidis	B	B	B	15	4	2	193	1	4	10	15	9	8	11	9	ST-269 complex	269	1	1	1
21249	M11 241034	UK [England]	2011	invasive (unspecified/other)	Neisseria meningitidis	B	B	B	202	1	1	35	4	42	26	46	24	2	20	17	ST-282 complex	282	4	4	2
21250	M11 241035	UK [England]	2011	invasive (unspecified/other)	Neisseria meningitidis	Y	Y	Y	25	1	1	26	4	12	5	18	9	11	9	17	ST-23 complex	1655	4	4	2
21251	M11 241036	UK [Wales]	2011	invasive (unspecified/other)	Neisseria meningitidis	B	B	B	15	4	2	193	1	4	10	15	9	8	11	9	ST-269 complex	269	1	1	1
21252	M11 241037	UK [England]	2011	invasive (unspecified/other)	Neisseria meningitidis	B	B	B	13	3	57	209	3	8	25	7	17	21	26	49	ST-1157 complex	1157	3	3	1
21253	M11 241039	UK [England]	2011	invasive (unspecified/other)	Neisseria meningitidis	C	C	C	13	5	1	251	11	2	3	4	3	8	4	6	ST-11 complex	11	11	2	1
21254	M11 241040	UK [England]	2011	invasive (unspecified/other)	Neisseria meningitidis	B	B	B	25	1	1	28	4	8	6	9	5	106	18	9	ST-41/44 complex	10269	4	4	2
21255	M11 241042	UK [England]	2011	invasive (unspecified/other)	Neisseria meningitidis	Y	Y	Y	25	1	1	26	4	12	5	18	9	11	9	17	ST-23 complex	1655	4	4	2
21256	M11 241043	UK [England]	2011	invasive (unspecified/other)	Neisseria meningitidis	C	C	C	13	16	57	145	63	2	3	4	3	8	4	6	ST-11 complex	11	NA	NA	1
21257	M11 241044	UK [England]	2011	invasive (unspecified/other)	Neisseria meningitidis	B	B	B	4	2	1	84	2	3	6	34	5	11	6	9	ST-41/44 complex	1194	2	3	1
21258	M11 241046	UK [Northern Ireland]	2011	invasive (unspecified/other)	Neisseria meningitidis	B	B	B	4	2	1	84	2	3	6	1	5	11	6	9	ST-41/44 complex	10270	2	3	1
21259	M11 241047	UK [England]	2011	invasive (unspecified/other)	Neisseria meningitidis	Y	Y	Y	25	1	1	26	4	12	5	18	9	11	9	17	ST-23 complex	1655	4	4	2
21260	M11 241048	UK [England]	2011	invasive (unspecified/other)	Neisseria meningitidis	B	B	B	19	1	1	83	4	4	10	2	5	38	11	9	ST-269 complex	275	4	4	2
21261	M11 241050	UK [England]	2011	invasive (unspecified/other)	Neisseria meningitidis	B	B	B	4	2	1	84	2	3	6	34	5	11	6	9	ST-41/44 complex	1194	2	3	1
21262	M11 241051	UK [England]	2011	invasive (unspecified/other)	Neisseria meningitidis	Y	Y	Y	25	1	1	26	4	10	5	18	9	11	9	17	ST-23 complex	23	4	4	2
21263	M11 241054	UK [England]	2011	invasive (unspecified/other)	Neisseria meningitidis	B	B	B	4	2	1	84	2	3	6	34	5	11	6	9	ST-41/44 complex	1194	2	3	1
21264	M11 241055	UK [England]	2011	invasive (unspecified/other)	Neisseria meningitidis	B	B	B	4	2	79	84	2	3	6	9	5	9	6	9	ST-41/44 complex	41	2	3	1
21265	M11 241057	UK [England]	2011	invasive (unspecified/other)	Neisseria meningitidis	B	B	B	224	15	1	252	68	4	10	5	4	6	3	8	ST-32 complex	32	NA	NA	1
21266	M11 241058	UK [England]	2011	invasive (unspecified/other)	Neisseria meningitidis	B	B	B	108	25	3	239	69	1	5	13	53	26	41	3	ST-162 complex	162	NA	NA	1
21267	M11 241059	UK [England]	2011	invasive (unspecified/other)	Neisseria meningitidis	B	B	B	13	3	57	209	3	8	25	1	17	11	26	49	ST-1157 complex	10271	3	3	1
21268	M11 241060	UK [Northern Ireland]	2011	invasive (unspecified/other)	Neisseria meningitidis	B	B	B	13	3	1	145	3	7	5	1	13	82	53	15	ST-213 complex	575	3	3	1
21269	M11 241061	UK [England]	2011	invasive (unspecified/other)	Neisseria meningitidis	B	B	B	15	4	2	193	1	4	10	15	17	8	11	9	ST-269 complex	1049	1	1	1
21270	M11 241063	UK [England]	2011	invasive (unspecified/other)	Neisseria meningitidis	B	B	B	4	2	1	84	2	3	6	9	5	9	6	9	ST-41/44 complex	41	2	3	1
21271	M11 241064	UK [England]	2011	invasive (unspecified/other)	Neisseria meningitidis	Y	Y	Y	25	1	1	26	4	8	5	18	17	11	24	21	ST-22 complex	3651	4	4	2
21272	M11 241065	UK [England]	2011	invasive (unspecified/other)	Neisseria meningitidis	Y	Y	Y	25	1	1	26	4	12	5	18	9	11	9	17	ST-23 complex	1655	4	4	2
21273	M11 241066	UK [England]	2011	invasive (unspecified/other)	Neisseria meningitidis	B	B	B	4	NA		6	84	144	3	6	9	5	9	6	9	ST-41/44 complex	41	NA	NA	1
21274	M11 241067	UK [England]	2011	invasive (unspecified/other)	Neisseria meningitidis	Y	Y	Y	25	1	1	26	4	12	5	18	9	11	9	17	ST-23 complex	1655	4	4	2
21275	M11 241068	UK [England]	2011	invasive (unspecified/other)	Neisseria meningitidis	B	B	B	45	8	77	87	20	7	5	1	13	36	53	15	ST-213 complex	213	20	4	3
21276	M11 241069	UK [England]	2011	invasive (unspecified/other)	Neisseria meningitidis	Y	Y	Y	25	1	1	26	4	10	5	18	9	11	9	17	ST-23 complex	23	4	4	2
21277	M11 241072	UK [England]	2011	invasive (unspecified/other)	Neisseria meningitidis	B	B	B	86	4	2	222	1	3	6	19	7	3	6	5		8988	1	1	1
21278	M11 241073	UK [England]	2011	invasive (unspecified/other)	Neisseria meningitidis	B	B	B	4	2	1	84	2	3	6	9	NA	9	6	9		NA	2	3	1
21279	M11 241074	UK [England]	2011	invasive (unspecified/other)	Neisseria meningitidis	B	B	B	19	1	1	83	4	4	10	2	5	3	11	9	ST-269 complex	1163	4	4	2
21280	M11 241075	UK [Wales]	2011	invasive (unspecified/other)	Neisseria meningitidis	B	B	B	13	3	1	145	3	17	5	19	17	3	26	2	ST-60 complex	60	3	3	1
21281	M11 241076	UK [England]	2011	invasive (unspecified/other)	Neisseria meningitidis	B	B	B	45	78	77	87	70	627	5	1	13	36	53	15	ST-213 complex	10272	NA	NA	3
21282	M11 241077	UK [England]	2011	invasive (unspecified/other)	Neisseria meningitidis	B	B	B	13	3	57	145	3	4	10	34	5	38	11	9	ST-269 complex	1161	3	3	1
21283	M11 241078	UK [England]	2011	invasive (unspecified/other)	Neisseria meningitidis	Y	Y	Y	25	1	1	26	4	12	5	18	9	11	9	17	ST-23 complex	1655	4	4	2
30069	M12 240156 b	UK	2012	invasive (unspecified/other)	Neisseria meningitidis	W	W	W	22	1	1	1	4	2	3	4	3	8	4	6	ST-11 complex	11	4	4	2
30070	M12 240160 b	UK	2012	invasive (unspecified/other)	Neisseria meningitidis	W	W	W	22	1	1	1	4	2	3	4	3	8	4	6	ST-11 complex	11	4	4	2
30071	M12 240196 b	UK	2012	carrier	Neisseria meningitidis	W	W	W	22	1	1	1	4	2	3	4	3	8	4	6	ST-11 complex	11	4	4	2
21284	M12 240000	UK [England]	2012	invasive (unspecified/other)	Neisseria meningitidis	B	B	B	19	1	62	65	4	7	5	1	13	36	53	15	ST-213 complex	213	4	4	2
21285	M12 240001	UK [England]	2012	invasive (unspecified/other)	Neisseria meningitidis	B	B	B	13	3	57	145	3	4	10	34	5	38	11	9	ST-269 complex	1161	3	3	1
21286	M12 240002	UK [England]	2012	invasive (unspecified/other)	Neisseria meningitidis	B	B	B	14	30	1	142	71	3	6	9	5	111	6	9	ST-41/44 complex	7385	NA	NA	1
21287	M12 240003	UK [England]	2012	invasive (unspecified/other)	Neisseria meningitidis	B	B	B	13	3	57	145	3	4	10	34	5	38	11	9	ST-269 complex	1161	3	3	1
21288	M12 240004	UK [Wales]	2012	invasive (unspecified/other)	Neisseria meningitidis	W	W	W	22	1	1	1	4	2	3	4	3	8	4	6	ST-11 complex	11	4	4	2
21289	M12 240006	UK [England]	2012	invasive (unspecified/other)	Neisseria meningitidis	B	B	B	13	3	57	145	3	4	10	34	5	38	11	9	ST-269 complex	1161	3	3	1
21290	M12 240008	UK [England]	2012	invasive (unspecified/other)	Neisseria meningitidis	W	W	W	16	1	1	60	4	11	5	18	8	11	4	21	ST-22 complex	184	4	4	2
21291	M12 240009	UK [England]	2012	invasive (unspecified/other)	Neisseria meningitidis	B	B	B	14	7	1	142	6	3	6	9	5	9	6	9	ST-41/44 complex	41	6	2	1
21292	M12 240010	UK [England]	2012	invasive (unspecified/other)	Neisseria meningitidis	B	B	B	4	2	1	84	2	3	6	9	5	11	6	9	ST-41/44 complex	154	2	3	1
21293	M12 240011	UK [England]	2012	invasive (unspecified/other)	Neisseria meningitidis	Y	Y	Y	25	1	1	26	4	10	5	18	9	11	9	17	ST-23 complex	23	4	4	2
21294	M12 240012	UK [England]	2012	invasive (unspecified/other)	Neisseria meningitidis	Y	Y	Y	25	1	1	26	4	12	5	18	9	11	9	17	ST-23 complex	1655	4	4	2
21295	M12 240013	UK [England]	2012	invasive (unspecified/other)	Neisseria meningitidis	Y	Y	Y	25	1	1	26	4	12	5	18	9	11	9	17	ST-23 complex	1655	4	4	2
21296	M12 240014	UK [England]	2012	invasive (unspecified/other)	Neisseria meningitidis	C	C	C	19	1	1	65	4	9	6	9	9	58	126	9	ST-41/44 complex	877	4	4	2
21297	M12 240015	UK [England]	2012	invasive (unspecified/other)	Neisseria meningitidis	NG	B	B	15	4	2	193	1	4	10	15	9	8	11	9	ST-269 complex	269	1	1	1
21298	M12 240016	UK [England]	2012	invasive (unspecified/other)	Neisseria meningitidis	W	W	W	22	1	1	1	4	2	3	4	3	8	4	6	ST-11 complex	11	4	4	2
21299	M12 240018	UK [England]	2012	invasive (unspecified/other)	Neisseria meningitidis	B	B	B	4	2	1	84	2	3	6	9	5	11	6	9	ST-41/44 complex	154	2	3	1
21300	M12 240019	UK [England]	2012	invasive (unspecified/other)	Neisseria meningitidis	B	B	B	47	1	57	90	15	12	5	12	35	60	22	17	ST-461 complex	461	15	4	3
21301	M12 240020	UK [Wales]	2012	invasive (unspecified/other)	Neisseria meningitidis	B	B	B	4	2	4	84	2	3	5	9	5	9	6	9	ST-41/44 complex	3754	2	3	1
21302	M12 240021	UK [England]	2012	invasive (unspecified/other)	Neisseria meningitidis	W	W	W	22	1	1	1	4	2	3	4	3	8	4	6	ST-11 complex	11	4	4	2
21303	M12 240022	UK [England]	2012	invasive (unspecified/other)	Neisseria meningitidis	B	B	B	45	8	77	87	20	7	5	1	13	36	53	15	ST-213 complex	213	20	4	3
21304	M12 240024	UK [England]	2012	invasive (unspecified/other)	Neisseria meningitidis	Y	Y	Y	25	1	1	26	4	12	5	18	9	11	9	17	ST-23 complex	1655	4	4	2
21305	M12 240027	UK [England]	2012	invasive (unspecified/other)	Neisseria meningitidis	W	W	W	22	1	1	1	4	2	3	4	3	8	4	6	ST-11 complex	11	4	4	2
21306	M12 240028	UK [England]	2012	invasive (unspecified/other)	Neisseria meningitidis	B	B	B	45	79	77	87	72	7	5	1	13	36	53	15	ST-213 complex	213	NA	NA	3
21307	M12 240029	UK [Wales]	2012	invasive (unspecified/other)	Neisseria meningitidis	B	B	B	13	3	57	145	3	4	10	34	5	38	11	9	ST-269 complex	1161	3	3	1
21308	M12 240030	UK [England]	2012	invasive (unspecified/other)	Neisseria meningitidis	B	B	B	19	1	1	83	4	4	10	2	5	38	11	9	ST-269 complex	275	4	4	2
21309	M12 240031	UK [England]	2012	invasive (unspecified/other)	Neisseria meningitidis	B	B	B	29	46	118	49	73	1	5	13	53	26	41	3	ST-162 complex	162	NA	NA	3
21310	M12 240032	UK [England]	2012	invasive (unspecified/other)	Neisseria meningitidis	B	B	B	4	2	79	84	2	3	6	9	5	9	6	9	ST-41/44 complex	41	2	3	1
21311	M12 240033	UK [Wales]	2012	invasive (unspecified/other)	Neisseria meningitidis	B	B	B	430	5	1	253	11	2	3	4	3	8	4	6	ST-11 complex	11	11	2	1
21312	M12 240034	UK [England]	2012	invasive (unspecified/other)	Neisseria meningitidis	B	B	B	143	4	2	99	1	4	10	15	9	14	11	17	ST-269 complex	1774	1	1	3
21313	M12 240035	UK [England]	2012	invasive (unspecified/other)	Neisseria meningitidis	B	B	B	4	2	1	84	2	3	6	34	5	11	6	9	ST-41/44 complex	1194	2	3	1
21314	M12 240036	UK [England]	2012	invasive (unspecified/other)	Neisseria meningitidis	B	B	B	4	2	1	84	2	3	6	9	5	9	6	9	ST-41/44 complex	41	2	3	1
21315	M12 240038	UK [England]	2012	invasive (unspecified/other)	Neisseria meningitidis	B	B	B	309	1	1	244	4	1	5	13	53	26	41	3	ST-162 complex	162	4	3	1

21316	M12 240039	UK [England]	2012	invasive (unspecified/other)	Neisseria meningitidis	B	B	B	4	2	1	84	2	3	6	9	5	11	6	9	ST-41/44 complex	154	2	3	1
21317	M12 240040	UK [England]	2012	invasive (unspecified/other)	Neisseria meningitidis	B	B	B	15	4	2	193	1	4	10	15	17	8	11	9	ST-269 complex	1049	1	1	1
21318	M12 240041	UK [Wales]	2012	invasive (unspecified/other)	Neisseria meningitidis	B	B	B	4	2	4	84	2	3	5	9	5	9	6	9	ST-41/44 complex	3754	2	3	1
21319	M12 240042	UK [Wales]	2012	invasive (unspecified/other)	Neisseria meningitidis	B	B	B	4	2	79	84	2	3	6	9	5	9	6	9	ST-41/44 complex	41	2	3	1
21320	M12 240044	UK [England]	2012	invasive (unspecified/other)	Neisseria meningitidis	B	B	B	37	1	1	408	4	628	8	10	26	10	18	16		10273	4	3	1
21321	M12 240045	UK [England]	2012	invasive (unspecified/other)	Neisseria meningitidis	B	B	B	14	7	1	142	6	3	6	9	5	9	6	9	ST-41/44 complex	41	6	2	1
21322	M12 240046	UK [England]	2012	invasive (unspecified/other)	Neisseria meningitidis	B	B	B	13	3	57	145	3	4	10	34	5	38	11	9	ST-269 complex	1161	3	3	1
21323	M12 240047	UK [England]	2012	invasive (unspecified/other)	Neisseria meningitidis	B	B	B	45	14	77	87	45	629	5	1	13	36	53	15	ST-213 complex	10274	NA	NA	3
21324	M12 240048	UK [England]	2012	invasive (unspecified/other)	Neisseria meningitidis	B	B	B	19	1	1	65	4	6	5	4	17	21	13	17		10275	4	4	2
21325	M12 240051	UK [England]	2012	invasive (unspecified/other)	Neisseria meningitidis	B	B	B	108	25	3	239	69	1	5	13	53	26	41	3	ST-162 complex	162	NA	NA	1
21326	M12 240054	UK [England]	2012	invasive (unspecified/other)	Neisseria meningitidis	B	B	B	2	5	57	254	11	4	10	5	4	6	3	8	ST-32 complex	32	11	2	1
21327	M12 240057	UK [England]	2012	invasive (unspecified/other)	Neisseria meningitidis	B	B	B	15	4	2	193	1	4	10	15	9	8	11	9	ST-269 complex	269	1	1	1
21328	M12 240058	UK [England]	2012	invasive (unspecified/other)	Neisseria meningitidis	B	B	B	2	5	57	254	11	4	10	5	4	6	3	8	ST-32 complex	32	11	2	1
21329	M12 240059	UK [England]	2012	invasive (unspecified/other)	Neisseria meningitidis	B	B	B	13	3	57	145	3	4	10	34	5	38	11	9	ST-269 complex	1161	3	3	1
21330	M12 240061	UK [Wales]	2012	invasive (unspecified/other)	Neisseria meningitidis	B	B	B	306	5	1	217	11	2	3	4	3	8	4	6	ST-11 complex	11	11	2	1
21331	M12 240062	UK [England]	2012	invasive (unspecified/other)	Neisseria meningitidis	B	B	B	37	1	1	408	4	7	8	10	19	10	663	2	ST-18 complex	10276	4	3	1
21332	M12 240063	UK [England]	2012	invasive (unspecified/other)	Neisseria meningitidis	B	B	B	45	14	77	87	45	7	5	1	13	9	53	15	ST-213 complex	3635	NA	NA	3
21333	M12 240066	UK [England]	2012	invasive (unspecified/other)	Neisseria meningitidis	B	B	B	15	10	2	193	17	4	10	15	9	8	11	9	ST-269 complex	269	NA	NA	1
21334	M12 240067	UK [England]	2012	invasive (unspecified/other)	Neisseria meningitidis	W	W	W	22	1	1	1	4	2	3	4	3	8	4	6	ST-11 complex	11	4	4	2
21335	M12 240069	UK [England]	2012	invasive (unspecified/other)	Neisseria meningitidis	B	B	B	22	1	1	1	4	2	3	4	3	8	4	6	ST-11 complex	11	4	4	2
21336	M12 240070	UK [England]	2012	invasive (unspecified/other)	Neisseria meningitidis	B	B	B	0	NA	1	0	NA	43	5	9	60	11	25	15		10277	NA	NA	NA
21337	M12 240071	UK [England]	2012	invasive (unspecified/other)	Neisseria meningitidis	B	B	B	167	8	1	126	20	3	6	696	5	17	22	9	ST-41/44 complex	10278	20	4	3
21338	M12 240072	UK [England]	2012	invasive (unspecified/other)	Neisseria meningitidis	Y	Y	Y	25	1	1	26	4	12	5	18	9	11	9	17	ST-23 complex	1655	4	4	2
21339	M12 240076	UK [England]	2012	invasive (unspecified/other)	Neisseria meningitidis	Y	Y	Y	21	1	1	7	4	6	5	173	13	5	24	17	ST-174 complex	1466	4	4	2
85623	M12 240077	UK [England]	2012	invasive (unspecified/other)	Neisseria meningitidis	B	B	B	14	7	1	142	6	3	6	9	5	9	6	9	ST-41/44 complex	41	6	2	1
21341	M12 240078	UK [England]	2012	invasive (unspecified/other)	Neisseria meningitidis	B	B	B	4	2	1	84	2	3	6	34	5	11	6	9	ST-41/44 complex	1194	2	3	1
21342	M12 240079	UK [England]	2012	invasive (unspecified/other)	Neisseria meningitidis	B	B	B	13	3	57	145	3	4	10	34	5	38	11	9	ST-269 complex	1161	3	3	1
21343	M12 240080	UK [England]	2012	invasive (unspecified/other)	Neisseria meningitidis	Y	Y	Y	25	1	1	26	4	12	5	18	9	11	9	17	ST-23 complex	1655	4	4	2
21344	M12 240081	UK [England]	2012	invasive (unspecified/other)	Neisseria meningitidis	B	B	B	14	1	1	142	13	3	5	656	5	3	6	9	ST-41/44 complex	9352	13	3	1
21345	M12 240083	UK [England]	2012	invasive (unspecified/other)	Neisseria meningitidis	B	B	B	13	3	1	145	3	7	5	1	13	82	661	15	ST-213 complex	10279	3	3	1
21346	M12 240084	UK [England]	2012	invasive (unspecified/other)	Neisseria meningitidis	Y	Y	Y	25	1	1	26	4	12	5	18	9	11	9	17	ST-23 complex	1655	4	4	2
21347	M12 240085	UK [England]	2012	invasive (unspecified/other)	Neisseria meningitidis	B	B	B	19	1	1	65	4	630	6	9	5	79	6	9	ST-41/44 complex	10280	4	4	2
21348	M12 240086	UK [England]	2012	invasive (unspecified/other)	Neisseria meningitidis	B	B	B	15	4	2	193	1	4	10	15	17	8	11	9	ST-269 complex	1049	1	1	1
21349	M12 240088	UK [England]	2012	invasive (unspecified/other)	Neisseria meningitidis	W	W	W	16	1	1	60	4	11	5	18	8	78	24	21	ST-22 complex	1224	4	4	2
21350	M12 240090	UK [England]	2012	invasive (unspecified/other)	Neisseria meningitidis	B	B	B	19	1	1	83	4	4	10	2	5	3	11	9	ST-269 complex	1163	4	4	2
21351	M12 240092	UK [England]	2012	invasive (unspecified/other)	Neisseria meningitidis	B	B	B	1	6	77	100	7	7	5	1	3	36	53	15	ST-213 complex	3496	7	1	1
21352	M12 240093	UK [England]	2012	invasive (unspecified/other)	Neisseria meningitidis	E	E	E	13	3	1	145	3	17	4	19	17	3	26	2	ST-60 complex	4146	3	3	1
21353	M12 240094	UK [England]	2012	invasive (unspecified/other)	Neisseria meningitidis	B	B	B	4	2	1	84	2	3	6	9	5	9	6	9	ST-41/44 complex	41	2	3	1
21354	M12 240095	UK [England]	2012	invasive (unspecified/other)	Neisseria meningitidis	W	W	W	22	1	1	1	4	2	3	4	3	8	4	6	ST-11 complex	11	4	4	2
21355	M12 240097	UK [England]	2012	invasive (unspecified/other)	Neisseria meningitidis	B	B	B	14	30	1	142	71	3	6	9	5	9	6	9	ST-41/44 complex	41	NA	NA	1
21356	M12 240098	UK [England]	2012	invasive (unspecified/other)	Neisseria meningitidis	Y	Y	Y	25	1	1	26	4	12	5	18	9	11	9	17	ST-23 complex	1655	4	4	2
21357	M12 240099	UK [England]	2012	invasive (unspecified/other)	Neisseria meningitidis	B	B	B	19	1	1	83	4	4	10	2	5	3	11	9	ST-269 complex	1163	4	4	2
21358	M12 240100	UK [England]	2012	invasive (unspecified/other)	Neisseria meningitidis	B	B	B	15	4	2	193	1	4	10	15	9	8	11	9	ST-269 complex	269	1	1	1
21359	M12 240101	UK [England]	2012	invasive (unspecified/other)	Neisseria meningitidis	C	C	C	306	5	1	217	11	2	3	4	3	8	4	6	ST-11 complex	11	11	2	1
21360	M12 240103	UK [England]	2012	invasive (unspecified/other)	Neisseria meningitidis	B	B	B	19	1	1	65	4	9	3	9	9	9	64	9	ST-41/44 complex	6058	4	4	2
21361	M12 240104	UK [England]	2012	invasive (unspecified/other)	Neisseria meningitidis	NG	B	B	0	5	1	917	11	2	3	4	3	8	4	6	ST-11 complex	11	11	2	NA
21362	M12 240105	UK [England]	2012	invasive (unspecified/other)	Neisseria meningitidis	B	B	B	13	3	57	145	3	4	10	34	5	38	11	9	ST-269 complex	1161	3	3	1
21363	M12 240107	UK [England]	2012	invasive (unspecified/other)	Neisseria meningitidis	B	B	B	187	5	1	101	11	7	25	1	13	9	53	49	ST-213 complex	10281	11	2	3
21364	M12 240111	UK [England]	2012	invasive (unspecified/other)	Neisseria meningitidis	C	C	C	306	5	1	217	11	2	3	4	3	8	4	6	ST-11 complex	11	11	2	1
21365	M12 240114	UK [England]	2012	invasive (unspecified/other)	Neisseria meningitidis	B	B	B	4	2	1	84	2	7	5	1	15	36	53	15	ST-213 complex	7309	2	3	1
21366	M12 240115	UK [England]	2012	invasive (unspecified/other)	Neisseria meningitidis	Y	Y	Y	25	1	1	26	4	12	5	18	9	11	9	17	ST-23 complex	1655	4	4	2
21367	M12 240116	UK [England]	2012	invasive (unspecified/other)	Neisseria meningitidis	B	B	B	15	4	2	193	1	4	10	15	9	8	5	9	ST-269 complex	1195	1	1	1
21368	M12 240117	UK [Wales]	2012	invasive (unspecified/other)	Neisseria meningitidis	B	B	B	14	1	1	142	13	3	6	19	5	3	6	9	ST-41/44 complex	340	13	3	1
21369	M12 240118	UK [Wales]	2012	invasive (unspecified/other)	Neisseria meningitidis	B	B	B	4	2	1	84	2	3	10	9	5	9	10	9	ST-41/44 complex	10282	2	3	1
21370	M12 240120	UK [Northern Ireland]	2012	invasive (unspecified/other)	Neisseria meningitidis	B	B	B	19	1	1	83	4	4	10	2	25	17	11	22		10283	4	4	2
21371	M12 240121	UK [England]	2012	invasive (unspecified/other)	Neisseria meningitidis	W	W	W	16	1	1	60	4	11	5	18	8	11	4	21	ST-22 complex	184	4	4	2
21372	M12 240122	UK [England]	2012	invasive (unspecified/other)	Neisseria meningitidis	Y	Y	Y	25	1	1	26	4	12	5	18	9	11	9	17	ST-23 complex	1655	4	4	2
21373	M12 240123	UK [England]	2012	invasive (unspecified/other)	Neisseria meningitidis	Y	Y	Y	25	1	1	26	4	12	5	18	9	11	9	17	ST-23 complex	1655	4	4	2
21374	M12 240124	UK [Wales]	2012	invasive (unspecified/other)	Neisseria meningitidis	B	B	B	4	2	79	84	2	3	6	9	5	9	6	9	ST-41/44 complex	41	2	3	1
21375	M12 240125	UK [England]	2012	invasive (unspecified/other)	Neisseria meningitidis	W	W	W	22	1	1	1	4	2	3	4	3	8	4	6	ST-11 complex	11	4	4	2
21376	M12 240126	UK [England]	2012	invasive (unspecified/other)	Neisseria meningitidis	B	B	B	4	2	1	84	2	3	6	34	5	11	6	9	ST-41/44 complex	1194	2	3	1
21377	M12 240127	UK [England]	2012	invasive (unspecified/other)	Neisseria meningitidis	W	W	W	22	1	1	1	4	2	3	4	3	8	4	6	ST-11 complex	11	4	4	2
21378	M12 240128	UK [England]	2012	invasive (unspecified/other)	Neisseria meningitidis	B	B	B	4	2	1	84	2	3	6	9	5	3	6	9	ST-41/44 complex	46	2	3	1
21379	M12 240131	UK [England]	2012	invasive (unspecified/other)	Neisseria meningitidis	NG	B	B	4	2	1	84	2	3	6	9	5	9	6	9	ST-41/44 complex	41	2	3	1
21380	M12 240132	UK [England]	2012	invasive (unspecified/other)	Neisseria meningitidis	W	W	W	16	1	1	60	4	11	5	18	8	11	4	21	ST-22 complex	184	4	4	2
21381	M12 240133	UK [England]	2012	invasive (unspecified/other)	Neisseria meningitidis	W	W	W	22	1	1	1	4	2	3	4	3	8	4	6	ST-11 complex	11	4	4	2
21382	M12 240134	UK [England]	2012	invasive (unspecified/other)	Neisseria meningitidis	B	B	B	4	2	1	84	2	429	6	9	5	9	6	9	ST-41/44 complex	9171	2	3	1
21383	M12 240135	UK [Wales]	2012	invasive (unspecified/other)	Neisseria meningitidis	B	B	B	13	3	57	209	3	8	25	7	17	21	26	49	ST-1157 complex	1157	3	3	1
21384	M12 240138	UK [England]	2012	invasive (unspecified/other)	Neisseria meningitidis	B	B	B	19	1	1	65	4	9	20	9	9	9	6	2	ST-41/44 complex	1097	4	4	2
21385	M12 240142	UK [England]	2012	invasive (unspecified/other)	Neisseria meningitidis	B	B	B	4	2	1	84	2	7	5	1	13	36	53	15	ST-213 complex	213	2	3	1
21386	M12 240144	UK [England]	2012	invasive (unspecified/other)	Neisseria meningitidis	W	W	W	22	1	1	1	4	631	3	4	3	8	4	6	ST-11 complex	10284	4	4	2
21387	M12 240145	UK [England]	2012	invasive (unspecified/other)	Neisseria meningitidis	B	B	B	29	39	77	49	74	7	5	1	13	36	53	15	ST-213 complex	213	NA	NA	3
21388	M12 240146	UK [England]	2012	invasive (unspecified/other)	Neisseria meningitidis	B	B	B	19	1	1	65	4	4	10	5	24	6	3	9	ST-32 complex	10285	4	4	2
21390	M12 240149	UK [England]	2012	invasive (unspecified/other)	Neisseria meningitidis	B	B	B	224	80	1	252	75	4	10	5	8	6	3	8	ST-32 complex	10286	NA	NA	1
21391	M12 240150	UK [England]	2012	invasive (unspecified/other)	Neisseria meningitidis	B	B	B	102	8	1	2	20	4	5	2	5	38	11	9		2307	20	4	2
21392	M12 240151	UK [England]	2012	invasive (unspecified/other)	Neisseria meningitidis	B	B	B	45	8	77	87	20	7	5	1	13	36	53	15	ST-213 complex	213	20	4	3
21393	M12 240153	UK [England]	2012	invasive (unspecified/other)	Neisseria meningitidis	B	B	B	15	4	2	193	1	4	10	15	9	8	11	9	ST-269 complex	269	1	1	1
21394	M12 240155	UK [England]	2012	invasive (unspecified/other)	Neisseria meningitidis	B	B	B	4	2	79	84	2	3	6	9	5	9	6	9	ST-41/44 complex	41	2	3	1
20460	M12 240156	UK [England]	2012	invasive (unspecified/other)	Neisseria meningitidis	W	W	W	22	1	1	1	4	2	3	4	3	8	4	6	ST-11 complex	11	4	4	2
21396	M12 240158	UK [England]	2012	invasive (unspecified/other)	Neisseria meningitidis	B	B	B	13	3	1	145	3	17	5	19	17	6	26	2	ST-60 complex	10287	3	3	1
20461	M12 240160	UK [England]	2012	invasive (unspecified/other)	Neisseria meningitidis	W	W	W	22	1	1	1	4	2	3	4	3	8	4	6	ST-11 complex	11	4	4	2
21398	M12 240161	UK [England]	2012	invasive (unspecified/other)	Neisseria meningitidis	B	B	B	4	2	1	84	2	3	6	9	5	9	6	9	ST-41/44 complex	41	2	3	1
21399	M12 240166	UK [England]	2012	invasive (unspecified/other)	Neisseria meningitidis	Y	Y	Y	25	1	1	26	4	10	5	18	NA	11	9	17		NA	4	4	2
21400	M12 240167	UK [England]	2012	invasive (unspecified/other)	Neisseria meningitidis	B	B	B	4	2	1	84	2	3	6	34	5	11	6	9	ST-41/44 complex	1194	2	3	1
21401	M12 240168	UK [England]	2012	invasive (unspecified/other)	Neisseria meningitidis	B	B	B	19	1	NA	65	4	12	6	9	9	9	6	9	ST-41/44 complex	43	4	4	2
21402	M12 240169	UK [England]	2012	invasive (unspecified/other)	Neisseria meningitidis	NG	B	B	19	1	1	65	4	3	6	9	5	9	6	70	ST-41/44 complex	414	4	4	2
21403	M12 240171	UK [England]	2012	invasive (unspecified/other)	Neisseria meningitidis	Y	Y	Y	25	1	1	26	4	12	5	18	9	11	9	17	ST-23 complex	1655	4	4	2
21404	M12 240174	UK [England]	2012	invasive (unspecified/other)	Neisseria meningitidis	Y	Y	Y	25	1	1	26	4	10	5	18	9	11	9	17	ST-23 complex	23	4	4	2
21405	M12 240175	UK [England]	2012	invasive (unspecified/other)	Neisseria meningitidis	B	B	B	19	1	1	83	4	4	10	2	5	3	11	9	ST-269 complex	1163	4	4	2
21406	M12 240176	UK [Wales]	2012	invasive (unspecified/other)	Neisseria meningitidis	B	B	B	13	3	57	145	3	4	5	18	5	8	11	9	ST-269 complex	10288	3	3	1
21407	M12 240177	UK [England]	2012	invasive (unspecified/other)	Neisseria meningitidis	C	C	C	306	5	1	217	11	2	3	4	3	8	4	6	ST-11 complex	11	11	2	1
21408	M12 240180	UK [England]	2012	invasive (unspecified/other)	Neisseria meningitidis	Y	Y	Y	25	1	1	26	4	12	5	18	9	11	9	17	ST-23 complex	1655	4	4	2
21409	M12 240181	UK [England]	2012	invasive (unspecified/other)	Neisseria meningitidis	Y	Y	Y	24	1	1	27	4	2	7	6	17	9	6	8	ST-167 complex	1624	4	4	2
21410	M12 240184	UK [Wales]	2012	invasive (unspecified/other)	Neisseria meningitidis	Y	Y	Y	25	1	1	26	4	10	5	18	9	11	9	17	ST-23 complex	23	4	4	2
21411	M12 240186	UK [England]	2012	invasive (unspecified/other)	Neisseria meningitidis	B	B	B	14	7	1	142	6	3	7	9	5	17	22	9	ST-41/44 complex	6782	6	2	1
21412	M12 240187	UK [England]	2012	invasive (unspecified/other)	Neisseria meningitidis	B	B	B	4	2	1	84	2	3	6	9	5	9	6	2	ST-41/44 complex	2314	2	3	1
21413	M12 240188	UK [England]	2012	invasive (unspecified/other)	Neisseria meningitidis	B	B	B	37	1	1	408	4	2	3	10	19	10	664	2	ST-18 complex	10289	4	3	1
21414	M12 240191	UK [England]	2012	invasive (unspecified/other)	Neisseria meningitidis	B	B	B	19	1	1	65	4	9	3	9	9	9	64	9	ST-41/44 complex	6058	4	4	2
95898	M12 240192	UK [England]	2012	invasive (unspecified/other)	Neisseria meningitidis	Y	Y	Y	16	1	1	60	4	11	5	18	17	11	24	21	ST-22 complex	114	4	4	2
21415	M12 240194	UK [England]	2012	invasive (unspecified/other)	Neisseria meningitidis	B	B	B	1	6	3	100	7	8	10	77	25	6	3	22		5151	7	1	1
20462	M12 240196	UK	2012	invasive (unspecified/other)	Neisseria meningitidis	W	W	W	22	1	1	1	4	2	3	4	3	8	4	6	ST-11 complex	11	4	4	2
21416	M12 240197	UK [England]	2012	invasive (unspecified/other)	Neisseria meningitidis	B	B	B	21	1	1	7	4	1	5	13	53	26	41	3	ST-162 complex	162	4	4	2
21417	M12 240200	UK [England]	2012	invasive (unspecified/other)	Neisseria meningitidis	C	C	C	19	1	2	65	4	9	4	9	17	5	6	2	ST-103 complex	5133	4	4	2
21418	M12 240201	UK [England]	2012	invasive (unspecified/other)	Neisseria meningitidis	B	B	B	15	10	2	193	17	4	10	15	9	8	11	9	ST-269 complex	269	NA	NA	1
21419	M12 240202	UK [England]	2012	invasive (unspecified/other)	Neisseria meningitidis	C	C	C	13	16	57	145	63	2	3	4	3	8	4	6	ST-11 complex	11	NA	NA	1
21420	M12 240203	UK [England]	2012	invasive (unspecified/other)	Neisseria meningitidis	B	B	B	1	6	3	100	7	1	5	13	53	26	41	3	ST-162 complex	162	7	1	1
21421	M12 240205	UK [England]	2012	invasive (unspecified/other)	Neisseria meningitidis	Y	Y	Y	21	1	1	7	4	6	5	173	13	5	24	17	ST-174 complex	1466	4	4	2
21422	M12 240206	UK [England]	2012	invasive (unspecified/other)	Neisseria meningitidis	B	B	B	13	3	57	145	3	4	10	34	5	38	11	9	ST-269 complex	1161	3	3	1
21423	M12 240207	UK [England]	2012	invasive (unspecified/other)	Neisseria meningitidis	B	B	B	4	2	1	84	2	3	6	9	5	11	6	9	ST-41/44 complex	154	2	3	1
21424	M12 240208	UK [England]	2012	invasive (unspecified/other)	Neisseria meningitidis	B	B	B	59	9	77	75	16	7	5	1	13	36	53	664	ST-213 complex	9884	16	2	3
95899	M12 240209	UK [England]	2012	invasive (unspecified/other)	Neisseria meningitidis	B	B	B	4	2	1	84	2	3	5	34	5	11	6	9	ST-41/44 complex	5986	2	3	1
21425	M12 240211	UK [England]	2012	invasive (unspecified/other)	Neisseria meningitidis	B	B	B	15	10	2	193	17	4	10	15	9	8	11	9	ST-269 complex	269	NA	NA	1
21426	M12 240213	UK [England]	2012	invasive (unspecified/other)	Neisseria meningitidis	B	B	B	4	2	1	84	2	3	6	9	5	9	6	9	ST-41/44 complex	41	2	3	1
21427	M12 240214	UK [England]	2012	invasive (unspecified/other)	Neisseria meningitidis	B	B	B	13	3	57	145	3	4	132	34	13	38	11	9		10290	3	3	1
21428	M12 240215	UK [England]	2012	invasive (unspecified/other)	Neisseria meningitidis	B	B	B	1	6	77	100	7	7	5	1	3	36	53	15	ST-213 complex	3496	7	1	1
21429	M12 240216	UK [England]	2012	invasive (unspecified/other)	Neisseria meningitidis	B	B	B	13	3	57	145	3	4	10	34	5	38	11	330		4713	3	3	1
95900	M12 240217	UK [England]	2012	invasive (unspecified/other)	Neisseria meningitidis	B	B	B	14	1	1	142	13	3	6	19	5	3	6	9	ST-41/44 complex	340	13	3	1
21430	M12 240219	UK [Northern Ireland]	2012	invasive (unspecified/other)	Neisseria meningitidis	C	C	C	15	4	2	193	1	12	10	15	9	8	11	13	ST-269 complex	10291	1	1	1
21431	M12 240220	UK [England]	2012	invasive (unspecified/other)	Neisseria meningitidis	B	B	B	4	2	1	84	2	3	6	9	5	8	6	9	ST-41/44 complex	485	2	3	1
21432	M12 240222	UK [England]	2012	invasive (unspecified/other)	Neisseria meningitidis	B	B	B	13	3	1	145	3	17	5	47	17	3	665	2	ST-60 complex	10292	3	3	1
21433	M12 240223	UK [England]	2012	invasive (unspecified/other)	Neisseria meningitidis	B	B	B	13	3	57	145	3	4	10	34	5	38	11	9	ST-269 complex	1161	3	3	1
21434	M12 240224	UK [England]	2012	invasive (unspecified/other)	Neisseria meningitidis	B	B	B	4	2	79	84	2	3	6	9	5	9	6	9	ST-41/44 complex	41	2	3	1
21435	M12 240225	UK [Wales]	2012	invasive (unspecified/other)	Neisseria meningitidis	B	B	B	45	8	77	87	20	7	5	1	13	36	6	15	ST-213 complex	2660	20	4	3
21436	M12 240226	UK [England]	2012	invasive (unspecified/other)	Neisseria meningitidis	B	B	B	29	12	77	49	76	7	5	1	13	36	53	15	ST-213 complex	213	NA	NA	3
21437	M12 240227	UK [England]	2012	invasive (unspecified/other)	Neisseria meningitidis	B	B	B	4	2	1	84	2	3	6	9	5	8	6	9	ST-41/44 complex	485	2	3	1
21438	M12 240228	UK [England]	2012	invasive (unspecified/other)	Neisseria meningitidis	B	B	B	45	8	77	87	20	7	5	1	13	36	53	15	ST-213 complex	213	20	4	3
21439	M12 240229	UK [Wales]	2012	invasive (unspecified/other)	Neisseria meningitidis	B	B	B	13	3	57	145	3	4	10	2	5	38	11	9	ST-269 complex	275	3	3	1
21440	M12 240230	UK [England]	2012	invasive (unspecified/other)	Neisseria meningitidis	Y	Y	Y	25	1	1	26	4	10	5	18	9	11	9	17	ST-23 complex	23	4	4	2
21441	M12 240231	UK [England]	2012	invasive (unspecified/other)	Neisseria meningitidis	B	B	B	1	6	3	100	7	8	10	77	4	6	3	8	ST-32 complex	749	7	1	1
21442	M12 240232	UK [England]	2012	invasive (unspecified/other)	Neisseria meningitidis	B	B	B	110	32	1	255	5	4	10	2	17	3	11	21		9311	NA	NA	1
21443	M12 240237	UK [England]	2012	invasive (unspecified/other)	Neisseria meningitidis	Y	Y	Y	25	1	1	26	4	12	5	18	9	11	9	17	ST-23 complex	1655	4	4	2
21444	M12 240238	UK [England]	2012	invasive (unspecified/other)	Neisseria meningitidis	B	B	B	13	3	57	145	3	12	10	2	5	38	11	9		7143	3	3	1
21445	M12 240239	UK [Northern Ireland]	2012	invasive (unspecified/other)	Neisseria meningitidis	B	B	B	15	4	2	193	1	4	10	15	9	8	11	9	ST-269 complex	269	1	1	1
21446	M12 240240	UK [England]	2012	invasive (unspecified/other)	Neisseria meningitidis	W	W	W	22	1	1	1	4	2	3	4	3	8	4	6	ST-11 complex	11	4	4	2
21447	M12 240241	UK [England]	2012	invasive (unspecified/other)	Neisseria meningitidis	Y	Y	Y	25	1	1	26	4	10	5	18	9	11	9	17	ST-23 complex	23	4	4	2
21448	M12 240242	UK [England]	2012	invasive (unspecified/other)	Neisseria meningitidis	Y	Y	Y	25	1	1	26	4	12	5	18	NA	11	9	17		NA	4	4	2
21449	M12 240243	UK [England]	2012	invasive (unspecified/other)	Neisseria meningitidis	B	B	B	15	10	2	193	17	4	10	15	9	8	11	9	ST-269 complex	269	NA	NA	1
21450	M12 240245	UK [England]	2012	invasive (unspecified/other)	Neisseria meningitidis	B	B	B	652	4	2	256	77	3	6	19	5	3	6	9	ST-41/44 complex	340	NA	NA	1

21451	M12 240248	UK [England]	2012	invasive (unspecified/other)	Neisseria meningitidis	Y	Y	Y	25	1	1	26	4	12	286	18	9	11	9	17	ST-23 complex	6463	4	4	2
21452	M12 240249	UK [England]	2012	invasive (unspecified/other)	Neisseria meningitidis	B	B	B	4	2	6	84	2	3	6	9	5	9	6	9	ST-41/44 complex	41	2	3	1
21453	M12 240250	UK [England]	2012	invasive (unspecified/other)	Neisseria meningitidis	B	B	B	541	41	2	74	51	12	17	4	39	9	22	8		2766	NA	NA	2
21454	M12 240251	UK [England]	2012	invasive (unspecified/other)	Neisseria meningitidis	W	W	W	16	1	1	60	4	11	5	18	154	11	24	21	ST-22 complex	1281	4	4	2
21455	M12 240252	UK [England]	2012	invasive (unspecified/other)	Neisseria meningitidis	B	B	B	14	7	1	257	6	10	6	9	5	11	6	9	ST-41/44 complex	2203	6	2	1
21456	M12 240253	UK [England]	2012	invasive (unspecified/other)	Neisseria meningitidis	B	B	B	185	4	77	127	1	7	5	1	13	36	53	15	ST-213 complex	213	1	1	3
21457	M12 240254	UK [England]	2012	invasive (unspecified/other)	Neisseria meningitidis	B	B	B	185	4	77	127	1	7	5	1	13	36	53	15	ST-213 complex	213	1	1	3
21458	M12 240255	UK [England]	2012	invasive (unspecified/other)	Neisseria meningitidis	C	C	C	22	1	1	1	4	2	3	4	3	8	4	6	ST-11 complex	11	4	4	2
21460	M12 240257	UK [England]	2012	invasive (unspecified/other)	Neisseria meningitidis	B	B	B	19	1	1	65	4	9	6	9	9	58	6	16	ST-41/44 complex	571	4	4	2
21461	M12 240259	UK [England]	2012	invasive (unspecified/other)	Neisseria meningitidis	B	B	B	0	NA	1	0	NA	43	5	9	60	3	19	21		7395	NA	NA	NA
21462	M12 240261	UK [England]	2012	invasive (unspecified/other)	Neisseria meningitidis	B	B	B	1	6	3	100	7	8	10	5	4	5	3	8	ST-32 complex	34	7	1	1
21463	M12 240263	UK [England]	2012	invasive (unspecified/other)	Neisseria meningitidis	B	B	B	653	17	1	128	37	3	6	298	5	9	18	9	ST-41/44 complex	3619	NA	NA	3
21464	M12 240264	UK [England]	2012	invasive (unspecified/other)	Neisseria meningitidis	B	B	B	4	2	79	84	2	3	6	9	5	9	6	9	ST-41/44 complex	41	2	3	1
21465	M12 240267	UK [England]	2012	invasive (unspecified/other)	Neisseria meningitidis	B	B	B	15	4	2	193	1	4	10	15	9	8	11	9	ST-269 complex	269	1	1	1
21466	M12 240272	UK [England]	2012	invasive (unspecified/other)	Neisseria meningitidis	Y	Y	Y	25	1	1	26	4	12	5	18	9	11	9	17	ST-23 complex	1655	4	4	2
21467	M12 240273	UK [England]	2012	invasive (unspecified/other)	Neisseria meningitidis	B	B	B	45	47	77	87	78	7	5	1	13	36	53	15	ST-213 complex	213	NA	NA	3
21468	M12 240274	UK [England]	2012	invasive (unspecified/other)	Neisseria meningitidis	B	B	B	4	2	6	84	2	3	6	9	5	9	6	9	ST-41/44 complex	41	2	3	1
21469	M12 240277	UK [Northern Ireland]	2012	invasive (unspecified/other)	Neisseria meningitidis	B	B	B	90	29	86	258	54	8	10	5	9	6	3	8	ST-32 complex	639	NA	NA	1
21470	M12 240284	UK [England]	2012	invasive (unspecified/other)	Neisseria meningitidis	B	B	B	4	2	1	84	2	3	2	34	5	11	6	9	ST-41/44 complex	10293	2	3	1
21471	M12 240287	UK [Wales]	2012	invasive (unspecified/other)	Neisseria meningitidis	B	B	B	13	3	57	145	3	4	10	34	5	38	11	9	ST-269 complex	1161	3	3	1
21472	M12 240288	UK [England]	2012	invasive (unspecified/other)	Neisseria meningitidis	Y	Y	Y	25	1	1	26	4	10	5	18	9	11	9	17	ST-23 complex	23	4	4	2
21473	M12 240289	UK [England]	2012	invasive (unspecified/other)	Neisseria meningitidis	Y	Y	Y	25	1	1	26	4	10	5	116	9	11	9	17	ST-23 complex	10294	4	4	2
21474	M12 240290	UK [England]	2012	invasive (unspecified/other)	Neisseria meningitidis	B	B	B	14	5	1	204	11	3	6	19	5	3	6	9	ST-41/44 complex	340	11	2	1
21475	M12 240291	UK [England]	2012	invasive (unspecified/other)	Neisseria meningitidis	Y	Y	Y	25	1	1	26	4	10	5	18	9	11	9	17	ST-23 complex	23	4	4	2
21476	M12 240293	UK [England]	2012	invasive (unspecified/other)	Neisseria meningitidis	Y	Y	Y	25	1	1	26	4	12	5	18	9	11	9	17	ST-23 complex	1655	4	4	2
21477	M12 240294	UK [England]	2012	invasive (unspecified/other)	Neisseria meningitidis	B	B	B	15	4	2	193	1	4	10	15	9	8	11	13	ST-269 complex	467	1	1	1
21478	M12 240296	UK [England]	2012	invasive (unspecified/other)	Neisseria meningitidis	B	B	B	19	1	1	65	4	9	6	9	9	58	6	16	ST-41/44 complex	571	4	4	2
21479	M12 240299	UK [England]	2012	invasive (unspecified/other)	Neisseria meningitidis	B	B	B	4	2	1	84	2	3	6	9	3	11	6	9	ST-41/44 complex	3818	2	3	1
21480	M12 240300	UK [England]	2012	invasive (unspecified/other)	Neisseria meningitidis	Y	Y	Y	25	1	1	26	4	12	5	18	9	11	9	17	ST-23 complex	1655	4	4	2
21481	M12 240301	UK [England]	2012	invasive (unspecified/other)	Neisseria meningitidis	B	B	B	4	2	1	84	2	3	6	34	5	11	6	22	ST-41/44 complex	10295	2	3	1
21482	M12 240302	UK [England]	2012	invasive (unspecified/other)	Neisseria meningitidis	B	B	B	19	8	2	129	20	6	430	63	9	9	18	9		10296	20	4	2
21483	M12 240303	UK [England]	2012	invasive (unspecified/other)	Neisseria meningitidis	B	B	B	13	3	57	145	3	4	5	34	5	38	11	9		5849	3	3	1
21484	M12 240305	UK [England]	2012	invasive (unspecified/other)	Neisseria meningitidis	B	B	B	4	2	79	84	2	3	6	9	5	9	6	9	ST-41/44 complex	41	2	3	1
21485	M12 240306	UK [Wales]	2012	invasive (unspecified/other)	Neisseria meningitidis	B	B	B	13	3	57	145	3	4	5	18	5	38	11	9		10297	3	3	1
21486	M12 240307	UK [England]	2012	invasive (unspecified/other)	Neisseria meningitidis	B	B	B	15	4	2	193	1	4	10	15	9	8	5	9	ST-269 complex	1195	1	1	1
21487	M12 240308	UK [England]	2012	invasive (unspecified/other)	Neisseria meningitidis	B	B	B	4	2	1	84	2	3	6	9	5	9	6	9	ST-41/44 complex	41	2	3	1
21488	M12 240309	UK [England]	2012	invasive (unspecified/other)	Neisseria meningitidis	B	B	B	14	1	1	142	13	3	6	9	5	9	21	9	ST-41/44 complex	2266	13	3	1
21489	M12 240310	UK [England]	2012	invasive (unspecified/other)	Neisseria meningitidis	B	B	B	15	4	2	193	1	4	10	15	9	8	11	9	ST-269 complex	269	1	1	1
21490	M12 240314	UK [England]	2012	invasive (unspecified/other)	Neisseria meningitidis	B	B	B	14	5	1	204	11	3	6	19	5	3	6	9	ST-41/44 complex	340	11	2	1
21491	M12 240315	UK [England]	2012	invasive (unspecified/other)	Neisseria meningitidis	B	B	B	14	1	1	142	13	3	5	656	5	3	6	9	ST-41/44 complex	9352	13	3	1
21492	M12 240317	UK [England]	2012	invasive (unspecified/other)	Neisseria meningitidis	W	W	W	22	1	1	1	4	2	3	4	3	8	4	6	ST-11 complex	11	4	4	2
21493	M12 240318	UK [England]	2012	invasive (unspecified/other)	Neisseria meningitidis	Y	Y	Y	25	1	1	26	4	12	5	18	9	11	9	17	ST-23 complex	1655	4	4	2
21494	M12 240319	UK [England]	2012	invasive (unspecified/other)	Neisseria meningitidis	B	B	B	4	2	1	84	2	3	6	9	5	9	6	9	ST-41/44 complex	41	2	3	1
21495	M12 240320	UK [England]	2012	invasive (unspecified/other)	Neisseria meningitidis	B	B	B	510	81	8	599	79	4	10	48	4	6	3	8	ST-32 complex	7460	NA	NA	1
21496	M12 240321	UK [England]	2012	invasive (unspecified/other)	Neisseria meningitidis	B	B	B	14	7	1	142	6	10	6	63	5	9	6	12	ST-41/44 complex	1403	6	2	1
21497	M12 240322	UK [England]	2012	invasive (unspecified/other)	Neisseria meningitidis	Y	Y	Y	25	1	1	26	4	10	5	18	9	11	9	17	ST-23 complex	23	4	4	2
21498	M12 240323	UK [England]	2012	invasive (unspecified/other)	Neisseria meningitidis	C	C	C	15	4	2	193	1	4	10	15	9	8	11	13	ST-269 complex	467	1	1	1
21499	M12 240324	UK [Wales]	2012	invasive (unspecified/other)	Neisseria meningitidis	W	W	W	22	1	1	1	4	2	3	4	3	8	4	6	ST-11 complex	11	4	4	2
21500	M12 240325	UK [England]	2012	invasive (unspecified/other)	Neisseria meningitidis	B	B	B	4	2	79	84	2	3	6	9	5	9	6	9	ST-41/44 complex	41	2	3	1
21501	M12 240326	UK [England]	2012	invasive (unspecified/other)	Neisseria meningitidis	B	B	B	45	14	77	87	45	7	5	1	13	36	53	15	ST-213 complex	213	NA	NA	3
21502	M12 240328	UK [England]	2012	invasive (unspecified/other)	Neisseria meningitidis	Y	Y	Y	25	1	1	26	4	10	5	18	9	11	9	17	ST-23 complex	23	4	4	2
21503	M12 240329	UK [England]	2012	invasive (unspecified/other)	Neisseria meningitidis	B	B	B	12	1	1	259	4	7	5	1	13	82	53	15	ST-213 complex	575	4	3	1
21504	M12 240330	UK [England]	2012	invasive (unspecified/other)	Neisseria meningitidis	Y	Y	Y	628	1	121	260	15	9	7	6	17	11	18	8	ST-167 complex	884	15	3	1
21505	M12 240332	UK [England]	2012	invasive (unspecified/other)	Neisseria meningitidis	Y	Y	Y	19	1	1	93	13	2	16	6	17	9	18	2	ST-103 complex	5987	13	4	2
21506	M12 240333	UK [England]	2012	invasive (unspecified/other)	Neisseria meningitidis	B	B	B	4	2	1	84	2	3	6	9	5	9	6	9	ST-41/44 complex	41	2	3	1
21507	M12 240334	UK [England]	2012	invasive (unspecified/other)	Neisseria meningitidis	B	B	B	14	1	1	142	13	3	6	19	5	3	6	9	ST-41/44 complex	340	13	3	1
21508	M12 240335	UK [England]	2012	invasive (unspecified/other)	Neisseria meningitidis	B	B	B	19	1	1	83	4	4	10	2	5	3	11	9	ST-269 complex	1163	4	4	2
27778	M12 240336	UK [England]	2012	invasive (unspecified/other)	Neisseria meningitidis	B	B	B	14	1	1	142	13	3	6	108	5	9	6	9	ST-41/44 complex	1475	13	3	1
28114	M12 240337	UK [England]	2012	invasive (unspecified/other)	Neisseria meningitidis	W	W	W	22	1	1	1	4	2	3	4	3	8	4	6	ST-11 complex	11	4	4	2
27779	M12 240338	UK [England]	2012	invasive (unspecified/other)	Neisseria meningitidis	B	B	B	95	5	1	32	11	4	10	7	9	6	10	12	ST-35 complex	10692	11	2	2
27780	M12 240340	UK [England]	2012	invasive (unspecified/other)	Neisseria meningitidis	B	B	B	297	15	81	276	58	7	5	1	13	36	53	15	ST-213 complex	213	NA	NA	1
27781	M12 240341	UK [England]	2012	invasive (unspecified/other)	Neisseria meningitidis	B	B	B	13	3	57	145	3	4	10	34	5	608	11	9	ST-269 complex	9180	3	3	1
28165	M12 240342	UK [England]	2012	invasive (unspecified/other)	Neisseria meningitidis	Y	Y	Y	25	1	1	26	4	10	5	18	9	11	9	17	ST-23 complex	23	4	4	2
28166	M12 240343	UK [Northern Ireland]	2012	invasive (unspecified/other)	Neisseria meningitidis	Y	Y	Y	25	1	1	26	4	12	5	18	9	11	9	17	ST-23 complex	1655	4	4	2
28079	M12 240345	UK [England]	2012	invasive (unspecified/other)	Neisseria meningitidis	C	C	C	19	1	2	65	4	9	4	9	17	5	6	2	ST-103 complex	5133	4	4	2
27782	M12 240347	UK [England]	2012	invasive (unspecified/other)	Neisseria meningitidis	B	B	B	47	1	57	90	15	12	5	12	35	60	22	17	ST-461 complex	461	15	4	3
27783	M12 240349	UK [England]	2012	invasive (unspecified/other)	Neisseria meningitidis	B	B	B	19	1	1	65	4	9	20	9	9	9	6	2	ST-41/44 complex	1097	4	4	2
28080	M12 240639	UK [England]	2012	invasive (unspecified/other)	Neisseria meningitidis	C	C	C	22	1	1	1	4	2	3	4	3	8	4	6	ST-11 complex	11	4	4	2
28115	M12 240640	UK [England]	2012	invasive (unspecified/other)	Neisseria meningitidis	W	W	W	22	1	1	1	4	2	3	4	3	8	4	6	ST-11 complex	11	4	4	2
28107	M12 240641	UK [England]	2012	invasive (unspecified/other)	Neisseria meningitidis	NG	E	E	13	3	57	209	3	8	342	7	17	21	26	49	ST-1157 complex	10301	3	3	1
28167	M12 240643	UK [England]	2012	invasive (unspecified/other)	Neisseria meningitidis	Y	Y	Y	25	1	1	26	4	12	5	18	9	11	9	17	ST-23 complex	1655	4	4	2
27784	M12 240644	UK [England]	2012	invasive (unspecified/other)	Neisseria meningitidis	B	B	B	4	2	1	84	2	6	3	6	17	26	21	8		4051	2	3	1
28168	M12 240646	UK [England]	2012	invasive (unspecified/other)	Neisseria meningitidis	Y	Y	Y	25	1	1	26	4	12	5	18	9	11	9	17	ST-23 complex	1655	4	4	2
27785	M12 240647	UK [Wales]	2012	invasive (unspecified/other)	Neisseria meningitidis	B	B	B	476	82	1	118	135	4	10	5	26	6	3	8	ST-32 complex	1096	NA	NA	3
27786	M12 240650	UK [England]	2012	invasive (unspecified/other)	Neisseria meningitidis	B	B	B	4	2	1	84	2	3	6	9	5	9	6	9	ST-41/44 complex	41	2	3	1
27787	M12 240651	UK [England]	2012	invasive (unspecified/other)	Neisseria meningitidis	B	B	B	608	2	1	277	2	3	6	34	5	11	6	9	ST-41/44 complex	1194	2	3	1
27788	M12 240652	UK [England]	2012	invasive (unspecified/other)	Neisseria meningitidis	B	B	B	4	2	1	84	2	3	6	9	5	11	6	9	ST-41/44 complex	154	2	3	1
27789	M12 240653	UK [Wales]	2012	invasive (unspecified/other)	Neisseria meningitidis	B	B	B	13	3	57	145	3	4	10	34	5	38	11	9	ST-269 complex	1161	3	3	1
27790	M12 240654	UK [England]	2012	invasive (unspecified/other)	Neisseria meningitidis	B	B	B	4	2	1	84	2	3	6	9	11	110	6	9	ST-41/44 complex	10693	2	3	1
28081	M12 240655	UK [England]	2012	invasive (unspecified/other)	Neisseria meningitidis	C	C	C	15	1	24	317	4	4	10	15	9	8	11	13	ST-269 complex	467	4	3	1
27791	M12 240656	UK [England]	2012	invasive (unspecified/other)	Neisseria meningitidis	B	B	B	14	7	1	142	6	10	6	9	17	9	6	9	ST-41/44 complex	3695	6	2	1
28116	M12 240657	UK [England]	2012	invasive (unspecified/other)	Neisseria meningitidis	W	W	W	22	1	1	1	4	2	3	4	3	8	4	6	ST-11 complex	11	4	4	2
27792	M12 240658	UK [England]	2012	invasive (unspecified/other)	Neisseria meningitidis	B	B	B	14	7	1	142	6	10	6	9	5	9	6	9	ST-41/44 complex	42	6	2	1
27793	M12 240659	UK [England]	2012	invasive (unspecified/other)	Neisseria meningitidis	B	B	B	16	1	1	60	4	11	5	5	8	11	24	21	ST-22 complex	1617	4	4	2
28169	M12 240661	UK [England]	2012	invasive (unspecified/other)	Neisseria meningitidis	Y	Y	Y	25	1	1	26	4	10	5	18	9	11	9	17	ST-23 complex	23	4	4	2
28117	M12 240663	UK [England]	2012	invasive (unspecified/other)	Neisseria meningitidis	W	W	W	22	1	1	1	4	2	3	4	3	8	4	6	ST-11 complex	11	4	4	2
27794	M12 240664	UK [England]	2012	invasive (unspecified/other)	Neisseria meningitidis	B	B	B	4	2	1	84	2	3	6	9	5	9	6	9	ST-41/44 complex	41	2	3	1
27795	M12 240667	UK [England]	2012	invasive (unspecified/other)	Neisseria meningitidis	B	B	B	4	2	1	84	2	3	6	34	5	11	6	9	ST-41/44 complex	1194	2	3	1
27796	M12 240668	UK [England]	2012	invasive (unspecified/other)	Neisseria meningitidis	B	B	B	4	2	79	84	2	3	6	9	5	9	6	9	ST-41/44 complex	41	2	3	1
27797	M12 240669	UK [England]	2012	invasive (unspecified/other)	Neisseria meningitidis	B	B	B	15	4	1	193	1	4	10	2	5	38	686	9		10694	1	1	1
28118	M12 240671	UK [England]	2012	invasive (unspecified/other)	Neisseria meningitidis	W	W	W	16	1	1	60	4	11	5	18	8	11	4	21	ST-22 complex	184	4	4	2
27798	M12 240672	UK [England]	2012	invasive (unspecified/other)	Neisseria meningitidis	B	B	B	19	1	1	65	4	9	20	9	9	9	6	2	ST-41/44 complex	1097	4	4	2
27799	M12 240674	UK [England]	2012	invasive (unspecified/other)	Neisseria meningitidis	B	B	B	13	3	57	145	3	4	10	34	5	38	11	9	ST-269 complex	1161	3	3	1
27800	M12 240675	UK [England]	2012	invasive (unspecified/other)	Neisseria meningitidis	B	B	B	15	4	2	193	1	4	10	15	9	8	687	9	ST-269 complex	10695	1	1	1
27801	M12 240678	UK [Northern Ireland]	2012	invasive (unspecified/other)	Neisseria meningitidis	B	B	B	15	4	2	193	1	4	10	15	9	8	11	9	ST-269 complex	269	1	1	1
28082	M12 240679	UK [England]	2012	invasive (unspecified/other)	Neisseria meningitidis	C	C	C	13	16	57	145	63	2	3	4	3	8	4	6	ST-11 complex	11	NA	NA	1
27802	M12 240681	UK [England]	2012	invasive (unspecified/other)	Neisseria meningitidis	B	B	B	19	1	1	65	4	9	6	9	9	58	6	16	ST-41/44 complex	571	4	4	2
27803	M12 240682	UK [England]	2012	invasive (unspecified/other)	Neisseria meningitidis	B	B	B	0	5	1	917	11	2	3	4	3	8	4	6	ST-11 complex	11	11	2	NA
27804	M12 240683	UK [England]	2012	invasive (unspecified/other)	Neisseria meningitidis	B	B	B	13	3	57	145	3	4	10	3	5	38	11	9	ST-269 complex	2157	3	3	1
27805	M12 240684	UK [England]	2012	invasive (unspecified/other)	Neisseria meningitidis	B	B	B	19	18	1	83	87	4	10	2	5	612	602	9		9199	NA	NA	2
27806	M12 240685	UK [England]	2012	invasive (unspecified/other)	Neisseria meningitidis	B	B	B	24	1	63	25	4	27	6	9	17	9	24	16		3242	4	4	2
27807	M12 240688	UK [England]	2012	invasive (unspecified/other)	Neisseria meningitidis	B	B	B	13	3	57	145	3	4	10	34	5	38	11	9	ST-269 complex	1161	3	3	1
27808	M12 240691	UK [England]	2012	invasive (unspecified/other)	Neisseria meningitidis	B	B	B	13	3	57	145	3	4	10	34	5	38	11	9	ST-269 complex	1161	3	3	1
27809	M12 240693	UK [England]	2012	invasive (unspecified/other)	Neisseria meningitidis	B	B	B	1	6	3	100	7	8	3	5	4	1	3	8	ST-32 complex	290	7	1	1
27810	M12 240694	UK [England]	2012	invasive (unspecified/other)	Neisseria meningitidis	B	B	B	15	4	2	193	1	4	10	15	9	9	11	9	ST-269 complex	1855	1	1	1
27811	M12 240697	UK [England]	2012	invasive (unspecified/other)	Neisseria meningitidis	B	B	B	4	2	79	84	2	3	6	9	5	9	6	9	ST-41/44 complex	41	2	3	1
28083	M12 240698	UK [England]	2012	invasive (unspecified/other)	Neisseria meningitidis	C	C	C	13	5	1	251	11	2	3	4	3	8	4	6	ST-11 complex	11	11	2	1
27812	M12 240699	UK [England]	2012	invasive (unspecified/other)	Neisseria meningitidis	B	B	B	14	1	1	142	13	3	6	4	670	3	6	9	ST-41/44 complex	10696	13	3	1
28170	M12 240701	UK [England]	2012	invasive (unspecified/other)	Neisseria meningitidis	Y	Y	Y	25	1	1	26	4	10	5	18	9	11	9	17	ST-23 complex	23	4	4	2
28119	M12 240702	UK [England]	2012	invasive (unspecified/other)	Neisseria meningitidis	W	W	W	22	1	1	1	4	2	3	4	3	8	4	6	ST-11 complex	11	4	4	2
27813	M12 240703	UK [England]	2012	invasive (unspecified/other)	Neisseria meningitidis	B	B	B	45	11	77	87	10	7	5	1	13	36	53	15	ST-213 complex	213	NA	NA	3
27814	M12 240704	UK [England]	2012	invasive (unspecified/other)	Neisseria meningitidis	B	B	B	47	1	57	90	15	451	5	12	35	192	22	17	ST-461 complex	7261	15	4	3
27815	M12 240705	UK [Wales]	2012	invasive (unspecified/other)	Neisseria meningitidis	B	B	B	141	1	4	116	4	8	5	1	13	36	53	15	ST-213 complex	10697	4	4	3
27816	M12 240706	UK [England]	2012	invasive (unspecified/other)	Neisseria meningitidis	B	B	B	4	2	1	84	2	3	6	159	5	9	6	9	ST-41/44 complex	10698	2	3	1
27817	M12 240707	UK [England]	2012	invasive (unspecified/other)	Neisseria meningitidis	B	B	B	15	4	2	193	1	4	10	15	9	8	11	9	ST-269 complex	269	1	1	1
27818	M12 240712	UK [England]	2012	invasive (unspecified/other)	Neisseria meningitidis	B	B	B	4	2	1	84	2	3	6	9	5	11	6	9	ST-41/44 complex	154	2	3	1
28171	M12 240713	UK [Wales]	2012	invasive (unspecified/other)	Neisseria meningitidis	Y	Y	Y	25	1	1	26	4	12	5	18	9	11	9	17	ST-23 complex	1655	4	4	2
27819	M12 240714	UK [England]	2012	invasive (unspecified/other)	Neisseria meningitidis	B	B	B	1	6	3	100	7	4	10	5	40	6	3	8	ST-32 complex	259	7	1	1
28172	M12 240715	UK [England]	2012	invasive (unspecified/other)	Neisseria meningitidis	Y	Y	Y	25	1	1	26	4	10	5	18	9	11	9	17	ST-23 complex	23	4	4	2
28084	M12 240716	UK [England]	2012	invasive (unspecified/other)	Neisseria meningitidis	C	C	C	22	1	1	1	4	2	3	4	3	8	4	6	ST-11 complex	11	4	4	2
28173	M12 240717	UK [England]	2012	invasive (unspecified/other)	Neisseria meningitidis	Y	Y	Y	21	1	1	7	4	6	5	173	13	5	24	17	ST-174 complex	1466	4	4	2
27820	M12 240718	UK [England]	2012	invasive (unspecified/other)	Neisseria meningitidis	B	B	B	19	1	1	83	4	4	10	2	5	3	11	9	ST-269 complex	1163	4	4	2
27821	M12 240719	UK [England]	2012	invasive (unspecified/other)	Neisseria meningitidis	B	B	B	13	3	57	145	3	4	10	34	5	38	11	9	ST-269 complex	1161	3	3	1
27822	M12 240720	UK [England]	2012	invasive (unspecified/other)	Neisseria meningitidis	B	B	B	4	2	79	84	2	3	6	9	5	9	6	9	ST-41/44 complex	41	2	3	1
27823	M12 240724	UK [England]	2012	invasive (unspecified/other)	Neisseria meningitidis	B	B	B	45	83	77	87	164	7	5	1	13	36	489	15	ST-213 complex	7676	NA	NA	3
27824	M12 240725	UK [England]	2012	invasive (unspecified/other)	Neisseria meningitidis	B	B	B	16	1	4	59	4	4	10	11	18	6	10	12	ST-35 complex	35	4	4	2
28174	M12 240728	UK [England]	2012	invasive (unspecified/other)	Neisseria meningitidis	Y	Y	Y	14	1	1	142	13	10	5	18	9	11	9	17	ST-23 complex	23	13	3	1
27825	M12 240729	UK [England]	2012	invasive (unspecified/other)	Neisseria meningitidis	B	B	B	13	3	57	145	3	4	10	2	5	38	11	9	ST-269 complex	275	3	3	1
27826	M12 240730	UK [England]	2012	invasive (unspecified/other)	Neisseria meningitidis	B	B	B	1	6	3	100	7	8	10	5	4	5	3	8	ST-32 complex	34	7	1	1
27827	M12 240732	UK [Wales]	2012	invasive (unspecified/other)	Neisseria meningitidis	B	B	B	1	6	3	100	7	8	10	5	4	6	3	15	ST-32 complex	8049	7	1	1
27828	M12 240733	UK [England]	2012	invasive (unspecified/other)	Neisseria meningitidis	B	B	B	450	84	5	278	165	7	5	1	9	36	688	15	ST-213 complex	10699	NA	NA	1
28120	M12 240734	UK [England]	2012	invasive (unspecified/other)	Neisseria meningitidis	W	W	W	16	22	1	97	32	11	5	168	8	11	4	21	ST-22 complex	1286	NA	NA	2
27829	M12 240735	UK [England]	2012	invasive (unspecified/other)	Neisseria meningitidis	B	B	B	1	6	3	100	7	8	10	77	4	6	3	8	ST-32 complex	749	7	1	1
27830	M12 240736	UK [England]	2012	invasive (unspecified/other)	Neisseria meningitidis	B	B	B	15	4	5	193	1	1	5	13	53	26	41	3	ST-162 complex	162	1	1	1
27831	M12 240737	UK [England]	2012	invasive (unspecified/other)	Neisseria meningitidis	B	B	B	15	4	2	193	1	4	10	15	17	8	11	17	ST-269 complex	479	1	1	1
27832	M12 240739	UK [England]	2012	invasive (unspecified/other)	Neisseria meningitidis	B	B	B	700	7	1	279	6	1	5	13	53	26	41	3	ST-162 complex	162	6	2	1
27833	M12 240741	UK [England]	2012	invasive (unspecified/other)	Neisseria meningitidis	B	B	B	4	2	79	84	2	3	6	9	5	26	6	9	ST-41/44 complex	8054	2	3	1
27834	M12 240745	UK [England]	2012	invasive (unspecified/other)	Neisseria meningitidis	B	B	B	143	4	2	99	1	4	10	15	9	14	11	17	ST-269 complex	1774	1	1	3

28175	M12 240746	UK [England]	2012	invasive (unspecified/other)	Neisseria meningitidis	Y	Y	Y	4	2	1	84	2	1	5	18	59	11	9	17	ST-23 complex	3030	2	3	1
28176	M12 240748	UK [England]	2012	invasive (unspecified/other)	Neisseria meningitidis	Y	Y	Y	25	1	1	26	4	8	4	6	17	5	18	2	ST-103 complex	103	4	4	2
27835	M12 240749	UK [England]	2012	invasive (unspecified/other)	Neisseria meningitidis	B	B	B	13	3	57	145	3	4	10	34	5	38	11	330		4713	3	3	1
27836	M12 240750	UK [England]	2012	invasive (unspecified/other)	Neisseria meningitidis	B	B	B	4	2	1	84	2	3	6	9	5	9	6	9	ST-41/44 complex	41	2	3	1
28121	M12 240751	UK [Wales]	2012	invasive (unspecified/other)	Neisseria meningitidis	W	W	W	22	1	1	1	4	662	3	4	3	8	4	6	ST-11 complex	10651	4	4	2
27837	M12 240752	UK [Northern Ireland]	2012	invasive (unspecified/other)	Neisseria meningitidis	B	B	B	4	2	1	84	2	3	6	9	5	11	6	9	ST-41/44 complex	154	2	3	1
28122	M12 240754	UK [England]	2012	invasive (unspecified/other)	Neisseria meningitidis	W	W	W	22	1	1	1	4	2	3	4	3	8	4	6	ST-11 complex	11	4	4	2
28108	M12 240756	UK [England]	2012	invasive (unspecified/other)	Neisseria meningitidis	NG	B	B	13	3	57	145	3	4	10	34	13	38	11	9	ST-269 complex	6604	3	3	1
27838	M12 240758	UK [England]	2012	invasive (unspecified/other)	Neisseria meningitidis	B	B	B	506	5	1	11	11	4	10	5	4	6	6	17	ST-32 complex	10700	11	2	2
28177	M12 240760	UK [England]	2012	invasive (unspecified/other)	Neisseria meningitidis	Y	Y	Y	25	1	1	26	4	12	5	18	9	11	9	17	ST-23 complex	1655	4	4	2
28123	M12 240762	UK [England]	2012	invasive (unspecified/other)	Neisseria meningitidis	W	W	W	16	1	1	60	4	11	5	18	8	11	4	21	ST-22 complex	184	4	4	2
27839	M12 240763	UK [England]	2012	invasive (unspecified/other)	Neisseria meningitidis	B	B	B	47	1	57	90	15	12	5	12	35	192	22	17	ST-461 complex	1946	15	4	3
27840	M12 240765	UK [England]	2012	invasive (unspecified/other)	Neisseria meningitidis	B	B	B	106	1	1	146	4	4	5	15	9	21	11	20	ST-269 complex	2738	4	4	2
28178	M12 240767	UK [England]	2012	invasive (unspecified/other)	Neisseria meningitidis	Y	Y	Y	25	1	1	26	4	12	5	18	9	11	9	17	ST-23 complex	1655	4	4	2
27841	M12 240769	UK [England]	2012	invasive (unspecified/other)	Neisseria meningitidis	B	B	B	13	3	57	145	3	8	105	52	3	9	11	7		1572	3	3	1
28124	M12 240772	UK [England]	2012	invasive (unspecified/other)	Neisseria meningitidis	W	W	W	16	1	1	60	4	11	5	18	8	11	24	21	ST-22 complex	22	4	4	2
27842	M12 240773	UK [England]	2012	invasive (unspecified/other)	Neisseria meningitidis	B	B	B	15	4	2	193	1	4	10	15	17	8	11	9	ST-269 complex	1049	1	1	1
28125	M12 240774	UK [England]	2012	invasive (unspecified/other)	Neisseria meningitidis	W	W	W	22	1	1	1	4	2	3	4	3	8	4	6	ST-11 complex	11	4	4	2
28085	M12 240776	UK [England]	2012	invasive (unspecified/other)	Neisseria meningitidis	C	C	C	306	5	1	217	11	2	3	4	3	8	4	6	ST-11 complex	11	11	2	1
27843	M12 240778	UK [England]	2012	invasive (unspecified/other)	Neisseria meningitidis	B	B	B	47	1	57	90	15	12	5	12	35	192	22	17	ST-461 complex	1946	15	4	3
28179	M12 240779	UK [England]	2012	invasive (unspecified/other)	Neisseria meningitidis	Y	Y	Y	25	1	1	26	4	12	5	18	9	11	9	17	ST-23 complex	1655	4	4	2
27844	M12 240781	UK [England]	2012	invasive (unspecified/other)	Neisseria meningitidis	B	B	B	19	1	1	83	4	4	10	2	5	38	11	9	ST-269 complex	275	4	4	2
27845	M12 240782	UK [Wales]	2012	invasive (unspecified/other)	Neisseria meningitidis	B	B	B	345	48	19	147	92	7	5	1	13	36	53	15	ST-213 complex	213	NA	NA	3
27846	M12 240783	UK [England]	2012	invasive (unspecified/other)	Neisseria meningitidis	B	B	B	4	2	1	84	2	3	6	9	5	9	6	9	ST-41/44 complex	41	2	3	1
28180	M12 240784	UK [England]	2012	invasive (unspecified/other)	Neisseria meningitidis	Y	Y	Y	21	1	1	7	4	6	5	173	13	5	24	17	ST-174 complex	1466	4	4	2
27847	M12 240785	UK [England]	2012	invasive (unspecified/other)	Neisseria meningitidis	B	B	B	4	2	1	84	2	3	6	9	5	9	6	9	ST-41/44 complex	41	2	3	1
27848	M12 240786	UK [England]	2012	invasive (unspecified/other)	Neisseria meningitidis	B	B	B	14	1	1	142	13	6	5	12	12	11	13	14		1359	13	3	1
27849	M12 240787	UK [Wales]	2012	invasive (unspecified/other)	Neisseria meningitidis	B	B	B	47	1	57	90	15	12	5	12	35	192	22	17	ST-461 complex	1946	15	4	3
28181	M12 240788	UK [England]	2012	invasive (unspecified/other)	Neisseria meningitidis	Y	Y	Y	25	1	1	26	4	10	5	116	9	11	9	17	ST-23 complex	10294	4	4	2
28126	M12 240789	UK [Wales]	2012	invasive (unspecified/other)	Neisseria meningitidis	W	W	W	16	1	1	60	4	11	5	18	8	11	4	21	ST-22 complex	184	4	4	2
27850	M12 240790	UK [England]	2012	invasive (unspecified/other)	Neisseria meningitidis	B	B	B	1	6	3	100	7	8	10	5	4	6	3	8	ST-32 complex	33	7	1	1
28182	M12 240791	UK [England]	2012	invasive (unspecified/other)	Neisseria meningitidis	Y	Y	Y	25	1	1	26	4	12	5	18	9	11	9	17	ST-23 complex	1655	4	4	2
27851	M12 240793	UK [England]	2012	invasive (unspecified/other)	Neisseria meningitidis	B	B	B	13	3	57	145	3	4	10	34	5	6	11	9	ST-269 complex	7789	3	3	1
27852	M12 240794	UK [England]	2012	invasive (unspecified/other)	Neisseria meningitidis	B	B	B	27	9	4	148	108	4	10	721	7	9	10	12	ST-35 complex	10701	NA	NA	2
28183	M12 240796	UK [England]	2012	invasive (unspecified/other)	Neisseria meningitidis	Y	Y	Y	23	1	1	4	4	2	7	1	17	1	18	8	ST-167 complex	10727	4	4	2
28184	M12 240797	UK [England]	2012	invasive (unspecified/other)	Neisseria meningitidis	Y	Y	Y	24	1	1	27	4	2	4	6	17	9	6	8	ST-167 complex	10728	4	4	2
28185	M12 240799	UK [England]	2012	invasive (unspecified/other)	Neisseria meningitidis	Y	Y	Y	25	1	1	26	4	10	5	18	9	11	9	17	ST-23 complex	23	4	4	2
28186	M12 240801	UK [England]	2012	invasive (unspecified/other)	Neisseria meningitidis	Y	Y	Y	25	1	1	26	4	12	5	18	9	11	9	17	ST-23 complex	1655	4	4	2
28187	M12 240802	UK [England]	2012	invasive (unspecified/other)	Neisseria meningitidis	Y	Y	Y	15	4	5	193	1	11	5	18	17	11	24	21	ST-22 complex	114	1	1	1
27853	M12 240803	UK [England]	2012	invasive (unspecified/other)	Neisseria meningitidis	B	B	B	1	6	3	100	7	8	10	5	4	5	3	8	ST-32 complex	34	7	1	1
27854	M12 240805	UK [England]	2012	invasive (unspecified/other)	Neisseria meningitidis	B	B	B	701	1	1	149	4	8	105	52	11	3	11	7		4953	4	4	2
27855	M12 240806	UK [Northern Ireland]	2012	invasive (unspecified/other)	Neisseria meningitidis	B	B	B	37	1	1	408	4	2	8	10	19	6	1	2	ST-18 complex	9774	4	3	1
27856	M12 240807	UK [England]	2012	invasive (unspecified/other)	Neisseria meningitidis	B	B	B	14	1	1	142	13	11	6	19	5	3	689	9		10702	13	3	1
27857	M12 240808	UK [England]	2012	invasive (unspecified/other)	Neisseria meningitidis	B	B	B	84	13	1	70	22	42	26	46	24	2	20	17	ST-282 complex	282	NA	NA	3
27858	M12 240809	UK [England]	2012	invasive (unspecified/other)	Neisseria meningitidis	B	B	B	16	1	4	59	4	43	5	6	60	3	19	15		8944	4	4	2
27859	M12 240810	UK [Northern Ireland]	2012	invasive (unspecified/other)	Neisseria meningitidis	B	B	B	15	4	2	193	1	4	10	15	9	8	11	9	ST-269 complex	269	1	1	1
27860	M12 240812	UK [England]	2012	invasive (unspecified/other)	Neisseria meningitidis	B	B	B	2	5	1	280	11	4	10	15	17	8	11	17	ST-269 complex	479	11	2	1
27861	M12 240814	UK [England]	2012	invasive (unspecified/other)	Neisseria meningitidis	B	B	B	19	40	1	83	50	4	10	19	244	38	11	9	ST-269 complex	10703	NA	NA	2
28188	M12 240816	UK [England]	2012	invasive (unspecified/other)	Neisseria meningitidis	Y	Y	Y	25	1	1	26	4	10	5	18	9	11	9	17	ST-23 complex	23	4	4	2
27862	M12 240817	UK [England]	2012	invasive (unspecified/other)	Neisseria meningitidis	B	B	B	1	6	7	100	7	4	10	5	4	6	3	8	ST-32 complex	32	7	1	1
27863	M12 240818	UK [England]	2012	invasive (unspecified/other)	Neisseria meningitidis	B	B	B	1	6	3	100	7	4	10	5	40	6	3	8	ST-32 complex	259	7	1	1
27864	M12 240819	UK [England]	2012	invasive (unspecified/other)	Neisseria meningitidis	B	B	B	47	1	1	90	15	12	5	12	35	60	22	17	ST-461 complex	461	15	4	3
28189	M12 240821	UK [England]	2012	invasive (unspecified/other)	Neisseria meningitidis	Y	Y	Y	23	1	1	4	4	2	16	6	17	9	18	20	ST-167 complex	10729	4	4	2
28190	M12 240822	UK [England]	2012	invasive (unspecified/other)	Neisseria meningitidis	Y	Y	Y	25	1	1	26	4	10	5	18	9	11	9	17	ST-23 complex	23	4	4	2
28127	M12 240823	UK [England]	2012	invasive (unspecified/other)	Neisseria meningitidis	W	W	W	16	1	1	60	4	11	5	168	8	11	4	21	ST-22 complex	1286	4	4	2
27865	M12 240825	UK [England]	2012	invasive (unspecified/other)	Neisseria meningitidis	B	B	B	4	2	1	84	2	3	6	9	5	8	6	9	ST-41/44 complex	485	2	3	1
28128	M12 240826	UK [England]	2012	invasive (unspecified/other)	Neisseria meningitidis	W	W	W	22	1	1	1	4	2	3	4	3	8	4	6	ST-11 complex	11	4	4	2
27866	M12 240827	UK [England]	2012	invasive (unspecified/other)	Neisseria meningitidis	B	B	B	13	3	57	145	3	4	10	34	5	38	11	9	ST-269 complex	1161	3	3	1
27867	M12 240831	UK [England]	2012	invasive (unspecified/other)	Neisseria meningitidis	B	B	B	15	4	2	193	1	4	10	15	17	8	11	9	ST-269 complex	1049	1	1	1
27868	M12 240834	UK [England]	2012	invasive (unspecified/other)	Neisseria meningitidis	B	B	B	2	5	1	280	11	4	10	15	17	8	11	17	ST-269 complex	479	11	2	1
28191	M12 240840	UK [England]	2012	invasive (unspecified/other)	Neisseria meningitidis	Y	Y	Y	25	1	1	26	4	10	5	18	9	11	9	17	ST-23 complex	23	4	4	2
27869	M12 240842	UK [England]	2012	invasive (unspecified/other)	Neisseria meningitidis	B	B	B	13	3	57	145	3	4	10	34	5	38	11	9	ST-269 complex	1161	3	3	1
27870	M12 240843	UK [England]	2012	invasive (unspecified/other)	Neisseria meningitidis	B	B	B	702	31	57	281	140	6	5	2	151	12	11	14		1976	NA	NA	1
27871	M12 240844	UK [England]	2012	invasive (unspecified/other)	Neisseria meningitidis	B	B	B	47	1	57	90	15	12	5	19	35	5	22	17	ST-461 complex	8223	15	4	3
27872	M12 240846	UK [England]	2012	invasive (unspecified/other)	Neisseria meningitidis	B	B	B	4	NA	6	84	144	3	6	9	5	9	6	9	ST-41/44 complex	41	NA	NA	1
27873	M12 240847	UK [England]	2012	invasive (unspecified/other)	Neisseria meningitidis	B	B	B	4	2	6	84	2	11	6	9	5	9	11	9	ST-41/44 complex	3666	2	3	1
27874	M12 240848	UK [England]	2012	invasive (unspecified/other)	Neisseria meningitidis	B	B	B	294	4	2	150	1	7	5	1	13	36	53	15	ST-213 complex	213	1	1	3
27875	M12 240849	UK [England]	2012	invasive (unspecified/other)	Neisseria meningitidis	B	B	B	4	2	1	84	2	3	6	9	5	9	6	9	ST-41/44 complex	41	2	3	1
27876	M12 240850	UK [England]	2012	invasive (unspecified/other)	Neisseria meningitidis	B	B	B	4	2	1	84	2	3	6	34	5	11	6	9	ST-41/44 complex	1194	2	3	1
27877	M12 240851	UK [England]	2012	invasive (unspecified/other)	Neisseria meningitidis	B	B	B	13	3	57	145	3	4	10	2	5	38	11	9	ST-269 complex	275	3	3	1
27878	M12 240852	UK [England]	2012	invasive (unspecified/other)	Neisseria meningitidis	B	B	B	245	10	2	282	17	4	10	15	9	8	11	9	ST-269 complex	269	NA	NA	1
27879	M12 240853	UK [England]	2012	invasive (unspecified/other)	Neisseria meningitidis	B	B	B	15	4	2	193	1	4	10	15	9	8	11	9	ST-269 complex	269	1	1	1
28086	M12 240854	UK [England]	2012	invasive (unspecified/other)	Neisseria meningitidis	C	C	C	13	51	1	251	199	6	5	173	13	5	24	17	ST-174 complex	1466	NA	NA	1
28087	M12 240855	UK [England]	2012	invasive (unspecified/other)	Neisseria meningitidis	C	C	C	13	51	1	251	199	6	5	173	13	5	24	17	ST-174 complex	1466	NA	NA	1
28192	M12 240857	UK [England]	2012	invasive (unspecified/other)	Neisseria meningitidis	Y	Y	Y	25	1	1	26	4	12	5	18	9	11	9	17	ST-23 complex	1655	4	4	2
27880	M12 240858	UK [England]	2012	invasive (unspecified/other)	Neisseria meningitidis	B	B	B	31	49	77	58	83	4	10	15	9	8	11	9	ST-269 complex	269	NA	NA	3
27881	M12 240861	UK [England]	2012	invasive (unspecified/other)	Neisseria meningitidis	B	B	B	16	56	79	60	NA	13	29	2	17	26	65	53		2123	NA	NA	2
28129	M12 240862	UK [England]	2012	invasive (unspecified/other)	Neisseria meningitidis	W	W	W	16	1	1	60	4	11	5	18	154	11	24	21	ST-22 complex	1281	4	4	2
27882	M12 240865	UK [England]	2012	invasive (unspecified/other)	Neisseria meningitidis	B	B	B	13	3	57	145	3	7	8	10	116	10	690	20	ST-18 complex	10704	3	3	1
27883	M12 240866	UK [England]	2012	invasive (unspecified/other)	Neisseria meningitidis	B	B	B	185	4	77	127	1	7	5	1	13	36	53	15	ST-213 complex	213	1	1	3
27884	M12 240867	UK [Wales]	2012	invasive (unspecified/other)	Neisseria meningitidis	B	B	B	15	4	2	193	1	4	10	15	17	8	11	17	ST-269 complex	479	1	1	1
27885	M12 240868	UK [England]	2012	invasive (unspecified/other)	Neisseria meningitidis	B	B	B	4	2	1	84	2	3	6	153	5	9	6	9	ST-41/44 complex	1090	2	3	1
27886	M12 240872	UK [England]	2012	invasive (unspecified/other)	Neisseria meningitidis	B	B	B	25	1	1	26	4	6	5	63	NA	9	11	9		NA	4	4	2
27887	M12 240873	UK [Wales]	2012	invasive (unspecified/other)	Neisseria meningitidis	B	B	B	13	3	1	145	3	12	5	12	17	685	22	17	ST-461 complex	10705	3	3	1
27888	M12 240874	UK [England]	2012	invasive (unspecified/other)	Neisseria meningitidis	B	B	B	1	6	3	100	7	4	10	5	4	5	3	2	ST-32 complex	74	7	1	1
27889	M12 240876	UK [England]	2012	invasive (unspecified/other)	Neisseria meningitidis	B	B	B	703	2	1	283	2	12	6	9	9	9	6	193	ST-41/44 complex	2821	2	3	1
27890	M12 240877	UK [Wales]	2012	invasive (unspecified/other)	Neisseria meningitidis	B	B	B	13	85	57	145	166	4	10	34	5	38	11	9	ST-269 complex	1161	NA	NA	1
27891	M12 240878	UK [England]	2012	invasive (unspecified/other)	Neisseria meningitidis	B	B	B	21	1	1	7	4	1	5	13	53	26	41	3	ST-162 complex	162	4	4	2
27892	M12 240879	UK [England]	2012	invasive (unspecified/other)	Neisseria meningitidis	B	B	B	13	3	57	145	3	4	10	34	5	38	11	9	ST-269 complex	1161	3	3	1
27893	M12 240880	UK [England]	2012	invasive (unspecified/other)	Neisseria meningitidis	B	B	B	19	1	2	65	4	4	10	15	9	8	11	9	ST-269 complex	269	4	4	2
28130	M12 240881	UK [England]	2012	invasive (unspecified/other)	Neisseria meningitidis	W/Y	W/Y	W/Y	25	1	1	26	4	12	5	18	9	11	9	17	ST-23 complex	1655	4	4	2
27894	M12 240884	UK [England]	2012	invasive (unspecified/other)	Neisseria meningitidis	B	B	B	1	6	7	100	7	4	10	5	4	6	3	8	ST-32 complex	32	7	1	1
28193	M12 240885	UK [England]	2012	invasive (unspecified/other)	Neisseria meningitidis	Y	Y	Y	25	1	1	26	4	12	5	18	9	11	9	17	ST-23 complex	1655	4	4	2
28194	M12 240886	UK [England]	2012	invasive (unspecified/other)	Neisseria meningitidis	Y	Y	Y	25	1	1	26	4	10	5	18	9	11	9	17	ST-23 complex	23	4	4	2
27895	M12 240887	UK [England]	2012	invasive (unspecified/other)	Neisseria meningitidis	B	B	B	4	2	1	84	2	3	6	9	5	63	6	9	ST-41/44 complex	1153	2	3	1
28195	M12 240888	UK [England]	2012	invasive (unspecified/other)	Neisseria meningitidis	Y	Y	Y	25	1	1	26	4	10	5	18	9	11	9	17	ST-23 complex	23	4	4	2
27896	M12 240893	UK [England]	2012	invasive (unspecified/other)	Neisseria meningitidis	B	B	B	15	4	2	193	1	4	10	9	9	8	11	9	ST-269 complex	8288	1	1	1
28196	M12 240894	UK [England]	2012	invasive (unspecified/other)	Neisseria meningitidis	Y	Y	Y	25	1	1	26	4	12	5	18	9	11	9	17	ST-23 complex	1655	4	4	2
28131	M12 240895	UK [England]	2012	invasive (unspecified/other)	Neisseria meningitidis	W	W	W	22	1	1	1	4	2	3	4	3	8	4	6	ST-11 complex	11	4	4	2
27897	M12 240896	UK [Wales]	2012	invasive (unspecified/other)	Neisseria meningitidis	B	B	B	74	20	1	284	8	4	10	5	26	6	3	8	ST-32 complex	1096	NA	NA	1
28197	M12 240897	UK [England]	2012	invasive (unspecified/other)	Neisseria meningitidis	Y	Y	Y	104	52	57	10	109	10	5	18	9	11	9	17	ST-23 complex	23	NA	NA	2
28132	M12 240898	UK [England]	2012	invasive (unspecified/other)	Neisseria meningitidis	W	W	W	22	1	1	1	4	2	3	4	3	8	4	6	ST-11 complex	11	4	4	2
27898	M12 240899	UK [England]	2012	invasive (unspecified/other)	Neisseria meningitidis	B	B	B	187	26	5	151	25	7	5	1	13	36	53	15	ST-213 complex	213	NA	NA	3
27899	M12 240900	UK [Wales]	2012	invasive (unspecified/other)	Neisseria meningitidis	B	B	B	15	4	2	193	1	4	10	15	9	8	11	2	ST-269 complex	2592	1	1	1
27900	M12 240901	UK [England]	2012	invasive (unspecified/other)	Neisseria meningitidis	B	B	B	87	4	88	285	1	12	2	9	671	9	6	9	ST-41/44 complex	10706	1	1	1
27901	M12 240902	UK [England]	2012	invasive (unspecified/other)	Neisseria meningitidis	B	B	B	4	2	1	84	2	3	6	9	21	9	6	9	ST-41/44 complex	1233	2	3	1
27902	M13 240000	UK [England]	2013	invasive (unspecified/other)	Neisseria meningitidis	B	B	B	19	1	1	83	4	4	4	2	5	38	11	9		1831	4	4	2
27903	M13 240001	UK [England]	2013	invasive (unspecified/other)	Neisseria meningitidis	B	B	B	4	2	1	84	2	3	6	9	5	9	6	9	ST-41/44 complex	41	2	3	1
28088	M13 240002	UK [England]	2013	invasive (unspecified/other)	Neisseria meningitidis	C	C	C	647	43	1	123	59	206	6	9	13	249	6	8		9777	NA	NA	3
27904	M13 240003	UK [England]	2013	invasive (unspecified/other)	Neisseria meningitidis	B	B	B	15	4	2	193	1	4	10	15	9	8	11	9	ST-269 complex	269	1	1	1
27905	M13 240004	UK [England]	2013	invasive (unspecified/other)	Neisseria meningitidis	B	B	B	506	5	2	11	11	4	10	15	17	8	11	17	ST-269 complex	479	11	2	2
27906	M13 240005	UK [England]	2013	invasive (unspecified/other)	Neisseria meningitidis	B	B	B	15	4	2	193	1	4	10	15	9	8	11	8	ST-269 complex	2166	1	1	1
27907	M13 240006	UK [England]	2013	invasive (unspecified/other)	Neisseria meningitidis	B	B	B	47	1	57	90	15	12	5	12	35	60	22	17	ST-461 complex	461	15	4	3
27908	M13 240007	UK [England]	2013	invasive (unspecified/other)	Neisseria meningitidis	B	B	B	704	2	1	286	2	3	6	34	5	11	6	9	ST-41/44 complex	1194	2	3	1
28089	M13 240008	UK [England]	2013	invasive (unspecified/other)	Neisseria meningitidis	C	C	C	22	1	1	1	4	2	3	4	3	8	4	6	ST-11 complex	11	4	4	2
27909	M13 240009	UK [England]	2013	invasive (unspecified/other)	Neisseria meningitidis	B	B	B	14	7	1	142	6	3	6	9	25	9	6	9	ST-41/44 complex	2799	6	2	1
28198	M13 240011	UK [England]	2013	invasive (unspecified/other)	Neisseria meningitidis	Y	Y	Y	25	1	1	26	4	12	5	18	9	11	9	17	ST-23 complex	1655	4	4	2
27910	M13 240012	UK [England]	2013	invasive (unspecified/other)	Neisseria meningitidis	B	B	B	4	2	1	84	2	3	6	9	5	9	6	9	ST-41/44 complex	41	2	3	1
28133	M13 240013	UK [England]	2013	invasive (unspecified/other)	Neisseria meningitidis	W	W	W	16	22	1	97	32	11	5	168	8	11	4	21	ST-22 complex	1286	NA	NA	2
27911	M13 240014	UK [England]	2013	invasive (unspecified/other)	Neisseria meningitidis	B	B	B	13	3	57	145	3	4	10	34	5	38	11	9	ST-269 complex	1161	3	3	1
28134	M13 240015	UK [England]	2013	invasive (unspecified/other)	Neisseria meningitidis	W	W	W	22	1	1	1	4	2	3	4	3	8	4	6	ST-11 complex	11	4	4	2
27912	M13 240017	UK [Wales]	2013	invasive (unspecified/other)	Neisseria meningitidis	B	B	B	1	6	3	100	7	8	10	5	672	6	3	15	ST-32 complex	10707	7	1	1
27913	M13 240019	UK [England]	2013	invasive (unspecified/other)	Neisseria meningitidis	B	B	B	702	31	1	281	140	3	228	9	5	9	6	70	ST-41/44 complex	7612	NA	NA	1
28199	M13 240020	UK [England]	2013	invasive (unspecified/other)	Neisseria meningitidis	Y	Y	Y	16	1	1	60	4	11	5	18	17	11	24	21	ST-22 complex	114	4	4	2
27914	M13 240021	UK [England]	2013	invasive (unspecified/other)	Neisseria meningitidis	B	B	B	108	25	3	239	69	12	5	12	35	60	22	17	ST-461 complex	461	NA	NA	1
28090	M13 240022	UK [England]	2013	invasive (unspecified/other)	Neisseria meningitidis	C	C	C	0	5	1	917	11	2	3	4	3	8	4	6	ST-11 complex	11	11	2	NA
27915	M13 240023	UK [England]	2013	invasive (unspecified/other)	Neisseria meningitidis	B	B	B	4	2	1	84	2	4	10	11	18	6	10	12	ST-35 complex	35	2	3	1
28135	M13 240025	UK [England]	2013	invasive (unspecified/other)	Neisseria meningitidis	W	W	W	22	1	1	1	4	2	3	4	3	8	4	6	ST-11 complex	11	4	4	2
28091	M13 240026	UK [Northern Ireland]	2013	invasive (unspecified/other)	Neisseria meningitidis	C	C	C	15	4	2	193	1	4	10	15	9	8	11	13	ST-269 complex	467	1	1	1
28136	M13 240028	UK [England]	2013	invasive (unspecified/other)	Neisseria meningitidis	W	W	W	22	1	1	1	4	2	3	4	3	8	4	6	ST-11 complex	11	4	4	2
27916	M13 240029	UK [England]	2013	invasive (unspecified/other)	Neisseria meningitidis	B	B	B	628	1	5	260	15	7	5	1	13	36	53	15	ST-213 complex	213	15	3	1
27917	M13 240030	UK [England]	2013	invasive (unspecified/other)	Neisseria meningitidis	B	B	B	13	3	57	145	3	4	10	34	353	38	11	9	ST-269 complex	10708	3	3	1
27918	M13 240031	UK [England]	2013	invasive (unspecified/other)	Neisseria meningitidis	B	B	B	19	1	1	93	13	12	6	9	17	9	73	9	ST-41/44 complex	337	13	4	2
27919	M13 240032	UK [England]	2013	invasive (unspecified/other)	Neisseria meningitidis	B	B	B	4	2	1	84	2	4	10	11	18	6	10	12	ST-35 complex	35	2	3	1
27920	M13 240033	UK [England]	2013	invasive (unspecified/other)	Neisseria meningitidis	B	B	B	13	3	57	145	3	4	10	34	5	38	11	9	ST-269 complex	1161	3	3	1
28200	M13 240035	UK [England]	2013	invasive (unspecified/other)	Neisseria meningitidis	Y	Y	Y	25	1	1	26	4	12	5	18	9	11	9	17	ST-23 complex	1655	4	4	2
27921	M13 240036	UK [England]	2013	invasive (unspecified/other)	Neisseria meningitidis	B	B	B	13	3	57	145	3	4	10	34	5	38	11	15		10709	3	3	1
27922	M13 240039	UK [England]	2013	invasive (unspecified/other)	Neisseria meningitidis	B	B	B	15	4	2	193	1	4	10	15	17	8	11	17	ST-269 complex	479	1	1	1

27923	M13 240040	UK [England]	2013	invasive (unspecified/other)	Neisseria meningitidis	B	B	B	15	4	2	193	1	4	10	15	17	8	11	17	ST-269 complex	479	1	1	1
27924	M13 240041	UK [England]	2013	invasive (unspecified/other)	Neisseria meningitidis	B	B	B	14	7	1	142	6	3	5	9	5	9	6	21	ST-41/44 complex	10710	6	2	1
27925	M13 240042	UK [England]	2013	invasive (unspecified/other)	Neisseria meningitidis	B	B	B	16	1	87	59	4	12	6	4	32	83	18	20		8006	4	4	2
27926	M13 240043	UK [England]	2013	invasive (unspecified/other)	Neisseria meningitidis	B	B	B	15	4	2	193	1	4	10	15	9	8	5	9	ST-269 complex	1195	1	1	1
28201	M13 240044	UK [Northern Ireland]	2013	invasive (unspecified/other)	Neisseria meningitidis	Y	Y	Y	25	1	1	26	4	12	5	18	9	11	9	17	ST-23 complex	1655	4	4	2
27927	M13 240045	UK [England]	2013	invasive (unspecified/other)	Neisseria meningitidis	B	B	B	13	3	57	209	3	12	5	12	35	192	22	17	ST-461 complex	1946	3	3	1
27928	M13 240046	UK [England]	2013	invasive (unspecified/other)	Neisseria meningitidis	B	B	B	19	1	1	83	4	4	10	2	5	38	11	9	ST-269 complex	275	4	4	2
27929	M13 240047	UK [England]	2013	invasive (unspecified/other)	Neisseria meningitidis	B	B	B	14	7	1	142	6	3	6	9	5	26	6	9	ST-41/44 complex	8054	6	2	1
27930	M13 240048	UK [England]	2013	invasive (unspecified/other)	Neisseria meningitidis	B	B	B	13	3	57	145	3	4	10	34	5	38	11	9	ST-269 complex	1161	3	3	1
27931	M13 240051	UK [England]	2013	invasive (unspecified/other)	Neisseria meningitidis	B	B	B	506	5	2	11	11	4	10	15	17	8	11	17	ST-269 complex	479	11	2	2
28202	M13 240053	UK [England]	2013	invasive (unspecified/other)	Neisseria meningitidis	Y	Y	Y	24	1	1	27	4	12	7	6	17	9	6	8	ST-167 complex	10730	4	4	2
27932	M13 240054	UK [England]	2013	invasive (unspecified/other)	Neisseria meningitidis	B	B	B	143	4	2	99	1	4	10	15	9	14	11	17	ST-269 complex	1774	1	1	3
28203	M13 240055	UK [England]	2013	invasive (unspecified/other)	Neisseria meningitidis	Y	Y	Y	16	1	1	60	4	11	5	18	17	11	24	21	ST-22 complex	114	4	4	2
28137	M13 240056	UK [England]	2013	invasive (unspecified/other)	Neisseria meningitidis	W	W	W	22	1	1	1	4	2	3	4	3	8	4	6	ST-11 complex	11	4	4	2
27933	M13 240057	UK [England]	2013	invasive (unspecified/other)	Neisseria meningitidis	B	B	B	47	1	57	90	15	12	5	12	35	5	22	17	ST-461 complex	5983	15	4	3
28204	M13 240059	UK [England]	2013	invasive (unspecified/other)	Neisseria meningitidis	Y	Y	Y	24	1	1	27	4	2	4	6	17	9	6	8	ST-167 complex	10728	4	4	2
27934	M13 240063	UK [England]	2013	invasive (unspecified/other)	Neisseria meningitidis	B	B	B	108	25	3	239	69	12	5	12	35	60	22	17	ST-461 complex	461	NA	NA	1
28205	M13 240064	UK [England]	2013	invasive (unspecified/other)	Neisseria meningitidis	Y	Y	Y	25	1	1	26	4	12	5	18	9	11	9	17	ST-23 complex	1655	4	4	2
28138	M13 240066	UK [England]	2013	invasive (unspecified/other)	Neisseria meningitidis	W	W	W	22	1	1	1	4	2	3	4	3	8	4	6	ST-11 complex	11	4	4	2
27935	M13 240067	UK [England]	2013	invasive (unspecified/other)	Neisseria meningitidis	B	B	B	15	4	2	193	1	4	10	2	5	38	11	9	ST-269 complex	275	1	1	1
28092	M13 240068	UK [England]	2013	invasive (unspecified/other)	Neisseria meningitidis	C	C	C	22	1	1	1	4	2	3	4	3	8	4	6	ST-11 complex	11	4	4	2
27936	M13 240069	UK [England]	2013	invasive (unspecified/other)	Neisseria meningitidis	B	B	B	13	3	57	145	3	4	10	34	5	38	11	9	ST-269 complex	1161	3	3	1
27937	M13 240072	UK [England]	2013	invasive (unspecified/other)	Neisseria meningitidis	B	B	B	15	10	2	193	17	4	10	15	9	8	11	9	ST-269 complex	269	NA	NA	1
28206	M13 240073	UK [England]	2013	invasive (unspecified/other)	Neisseria meningitidis	Y	Y	Y	25	1	1	26	4	10	5	18	9	11	9	17	ST-23 complex	23	4	4	2
27938	M13 240074	UK [England]	2013	invasive (unspecified/other)	Neisseria meningitidis	B	B	B	187	1	57	152	15	12	5	12	35	60	22	17	ST-461 complex	461	15	4	3
27939	M13 240075	UK [England]	2013	invasive (unspecified/other)	Neisseria meningitidis	B	B	B	19	1	1	83	4	4	10	2	5	15	11	22		7196	4	4	2
27940	M13 240076	UK [England]	2013	invasive (unspecified/other)	Neisseria meningitidis	B	B	B	90	29	2	258	54	222	444	58	261	263	5	13		10711	NA	NA	1
28139	M13 240077	UK [England]	2013	invasive (unspecified/other)	Neisseria meningitidis	W	W	W	22	1	1	1	4	662	3	4	3	8	4	6	ST-11 complex	10651	4	4	2
27941	M13 240078	UK [England]	2013	invasive (unspecified/other)	Neisseria meningitidis	B	B	B	47	1	57	90	15	12	5	12	35	192	22	17	ST-461 complex	1946	15	4	3
27942	M13 240081	UK [England]	2013	invasive (unspecified/other)	Neisseria meningitidis	B	B	B	45	8	1	87	20	7	395	1	13	36	53	15	ST-213 complex	9194	20	4	3
27943	M13 240084	UK [England]	2013	invasive (unspecified/other)	Neisseria meningitidis	B	B	B	14	18	1	142	126	3	6	108	5	9	6	9	ST-41/44 complex	1475	NA	NA	1
27944	M13 240085	UK [England]	2013	invasive (unspecified/other)	Neisseria meningitidis	B	B	B	167	8	1	126	20	3	6	9	5	9	22	9	ST-41/44 complex	40	20	4	3
27945	M13 240086	UK [England]	2013	invasive (unspecified/other)	Neisseria meningitidis	B	B	B	16	1	4	59	4	132	3	19	17	62	21	2		1434	4	4	2
28207	M13 240087	UK [England]	2013	invasive (unspecified/other)	Neisseria meningitidis	Y	Y	Y	25	1	1	26	4	12	5	18	9	11	9	17	ST-23 complex	1655	4	4	2
27946	M13 240088	UK [England]	2013	invasive (unspecified/other)	Neisseria meningitidis	B	B	B	4	2	1	84	2	3	6	9	5	9	6	9	ST-41/44 complex	41	2	3	1
28140	M13 240089	UK [England]	2013	invasive (unspecified/other)	Neisseria meningitidis	W	W	W	16	1	1	60	4	11	5	18	8	11	4	21	ST-22 complex	184	4	4	2
27947	M13 240090	UK [England]	2013	invasive (unspecified/other)	Neisseria meningitidis	B	B	B	14	5	1	204	11	3	6	19	201	3	6	9	ST-41/44 complex	6761	11	2	1
27948	M13 240091	UK [England]	2013	invasive (unspecified/other)	Neisseria meningitidis	B	B	B	21	1	1	7	4	1	5	13	53	26	41	3	ST-162 complex	162	4	4	2
27949	M13 240095	UK [England]	2013	invasive (unspecified/other)	Neisseria meningitidis	B	B	B	15	4	2	193	1	4	10	15	17	8	11	9	ST-269 complex	1049	1	1	1
27950	M13 240097	UK [Wales]	2013	invasive (unspecified/other)	Neisseria meningitidis	B	B	B	16	1	1	60	4	11	20	9	9	9	6	21	ST-41/44 complex	3893	4	4	2
27951	M13 240098	UK [Wales]	2013	invasive (unspecified/other)	Neisseria meningitidis	B	B	B	302	24	1	121	55	9	20	9	9	9	6	2	ST-41/44 complex	1097	NA	NA	2
28208	M13 240099	UK [England]	2013	invasive (unspecified/other)	Neisseria meningitidis	Y	Y	Y	25	1	1	26	4	12	5	18	9	11	9	17	ST-23 complex	1655	4	4	2
28209	M13 240102	UK [England]	2013	invasive (unspecified/other)	Neisseria meningitidis	Y	Y	Y	25	1	1	26	4	12	5	18	9	11	9	17	ST-23 complex	1655	4	4	2
27952	M13 240105	UK [England]	2013	invasive (unspecified/other)	Neisseria meningitidis	B	B	B	13	3	57	145	3	8	105	52	3	9	11	7		1572	3	3	1
27953	M13 240106	UK [England]	2013	invasive (unspecified/other)	Neisseria meningitidis	B	B	B	1	6	7	100	7	4	10	5	4	6	3	8	ST-32 complex	32	7	1	1
28141	M13 240109	UK [England]	2013	invasive (unspecified/other)	Neisseria meningitidis	W	W	W	22	1	1	1	4	2	3	4	3	8	4	6	ST-11 complex	11	4	4	2
27954	M13 240111	UK [England]	2013	invasive (unspecified/other)	Neisseria meningitidis	B	B	B	4	2	1	84	2	3	6	34	5	11	6	9	ST-41/44 complex	1194	2	3	1
27955	M13 240112	UK [England]	2013	invasive (unspecified/other)	Neisseria meningitidis	B	B	B	15	10	2	193	17	4	10	15	9	8	11	9	ST-269 complex	269	NA	NA	1
28142	M13 240114	UK [England]	2013	invasive (unspecified/other)	Neisseria meningitidis	W	W	W	22	1	1	1	4	2	3	4	3	8	4	6	ST-11 complex	11	4	4	2
27956	M13 240115	UK [England]	2013	invasive (unspecified/other)	Neisseria meningitidis	B	B	B	9	50	1	187	132	3	6	34	5	11	6	9	ST-41/44 complex	1194	NA	NA	1
28210	M13 240116	UK [England]	2013	invasive (unspecified/other)	Neisseria meningitidis	Y	Y	Y	25	1	1	26	4	12	5	18	9	11	9	17	ST-23 complex	1655	4	4	2
27957	M13 240118	UK [England]	2013	invasive (unspecified/other)	Neisseria meningitidis	B	B	B	1	6	3	100	7	8	10	5	4	5	3	8	ST-32 complex	34	7	1	1
27958	M13 240120	UK [England]	2013	invasive (unspecified/other)	Neisseria meningitidis	B	B	B	4	2	79	84	2	3	6	9	5	9	6	9	ST-41/44 complex	41	2	3	1
27959	M13 240121	UK [England]	2013	invasive (unspecified/other)	Neisseria meningitidis	B	B	B	13	3	57	145	3	4	10	34	5	38	11	9	ST-269 complex	1161	3	3	1
27960	M13 240123	UK [England]	2013	invasive (unspecified/other)	Neisseria meningitidis	B	B	B	4	2	79	84	2	3	6	9	5	9	6	9	ST-41/44 complex	41	2	3	1
27961	M13 240124	UK [England]	2013	invasive (unspecified/other)	Neisseria meningitidis	B	B	B	13	3	57	145	3	4	10	34	5	38	11	9	ST-269 complex	1161	3	3	1
27962	M13 240125	UK [England]	2013	invasive (unspecified/other)	Neisseria meningitidis	B	B	B	202	1	1	35	4	42	26	46	24	6	20	17	ST-282 complex	1802	4	4	2
28211	M13 240126	UK [Northern Ireland]	2013	invasive (unspecified/other)	Neisseria meningitidis	Y	Y	Y	95	5	1	32	11	2	7	6	8	9	7	8	ST-167 complex	3980	11	2	2
27963	M13 240127	UK [England]	2013	invasive (unspecified/other)	Neisseria meningitidis	B	B	B	187	5	77	101	11	7	5	1	13	36	53	15	ST-213 complex	213	11	2	3
27964	M13 240130	UK [England]	2013	invasive (unspecified/other)	Neisseria meningitidis	B	B	B	1	6	3	100	7	8	10	77	4	6	3	8	ST-32 complex	749	7	1	1
28212	M13 240131	UK [England]	2013	invasive (unspecified/other)	Neisseria meningitidis	Y	Y	Y	25	1	1	26	4	10	5	18	9	11	9	17	ST-23 complex	23	4	4	2
27965	M13 240132	UK [England]	2013	invasive (unspecified/other)	Neisseria meningitidis	B	B	B	45	8	77	87	20	7	5	1	13	36	53	15	ST-213 complex	213	20	4	3
28109	M13 240134	UK [England]	2013	invasive (unspecified/other)	Neisseria meningitidis	NG	cnl	cnl	4	1	1	206	4	5	4	17	15	30	7	12	ST-198 complex	823	4	3	1
27966	M13 240135	UK [England]	2013	invasive (unspecified/other)	Neisseria meningitidis	B	B	B	9	50	1	187	132	3	6	34	5	11	6	9	ST-41/44 complex	1194	NA	NA	1
28213	M13 240136	UK [England]	2013	invasive (unspecified/other)	Neisseria meningitidis	Y	Y	Y	23	1	1	4	4	2	16	6	17	9	18	8	ST-167 complex	168	4	4	2
27967	M13 240142	UK [England]	2013	invasive (unspecified/other)	Neisseria meningitidis	B	B	B	24	1	63	25	4	27	6	9	17	9	6	16	ST-41/44 complex	1770	4	4	2
27968	M13 240143	UK [England]	2013	invasive (unspecified/other)	Neisseria meningitidis	B	B	B	21	1	1	7	4	1	5	13	53	26	41	623	ST-162 complex	9293	4	4	2
28214	M13 240144	UK [England]	2013	invasive (unspecified/other)	Neisseria meningitidis	Y	Y	Y	25	1	1	26	4	12	5	18	9	11	9	17	ST-23 complex	1655	4	4	2
27969	M13 240145	UK [England]	2013	invasive (unspecified/other)	Neisseria meningitidis	B	B	B	84	13	1	70	22	42	26	46	24	2	20	17	ST-282 complex	282	NA	NA	3
28215	M13 240146	UK [England]	2013	invasive (unspecified/other)	Neisseria meningitidis	Y	Y	Y	25	1	1	26	4	12	5	18	9	11	9	17	ST-23 complex	1655	4	4	2
27970	M13 240147	UK [England]	2013	invasive (unspecified/other)	Neisseria meningitidis	B	B	B	15	4	2	193	1	4	10	15	673	8	5	9	ST-269 complex	10712	1	1	1
27971	M13 240148	UK [Northern Ireland]	2013	invasive (unspecified/other)	Neisseria meningitidis	B	B	B	13	3	1	145	3	6	6	470	58	58	11	9		10713	3	3	1
27972	M13 240150	UK [England]	2013	invasive (unspecified/other)	Neisseria meningitidis	B	B	B	19	1	1	83	4	4	10	616	5	38	24	9		10714	4	4	2
27973	M13 240153	UK [England]	2013	invasive (unspecified/other)	Neisseria meningitidis	B	B	B	13	3	1	145	3	563	5	19	90	3	26	2	ST-60 complex	9327	3	3	1
28216	M13 240154	UK [England]	2013	invasive (unspecified/other)	Neisseria meningitidis	Y	Y	Y	25	1	1	26	4	12	5	18	9	11	9	17	ST-23 complex	1655	4	4	2
28093	M13 240155	UK [England]	2013	invasive (unspecified/other)	Neisseria meningitidis	C	C	C	13	5	1	251	11	2	3	4	3	8	4	6	ST-11 complex	11	11	2	1
27974	M13 240157	UK [England]	2013	invasive (unspecified/other)	Neisseria meningitidis	B	B	B	4	2	1	84	2	3	6	9	5	9	6	9	ST-41/44 complex	41	2	3	1
28143	M13 240158	UK [England]	2013	invasive (unspecified/other)	Neisseria meningitidis	W	W	W	22	1	1	1	4	2	3	4	3	8	4	6	ST-11 complex	11	4	4	2
27975	M13 240160	UK [Wales]	2013	invasive (unspecified/other)	Neisseria meningitidis	B	B	B	15	4	2	193	1	4	10	15	9	8	11	9	ST-269 complex	269	1	1	1
27976	M13 240161	UK [England]	2013	invasive (unspecified/other)	Neisseria meningitidis	B	B	B	16	1	4	59	4	4	10	11	18	6	10	12	ST-35 complex	35	4	4	2
28094	M13 240162	UK [England]	2013	invasive (unspecified/other)	Neisseria meningitidis	C	C	C	13	5	1	251	11	2	3	4	3	8	4	6	ST-11 complex	11	11	2	1
27977	M13 240164	UK [England]	2013	invasive (unspecified/other)	Neisseria meningitidis	B	B	B	15	4	2	193	1	4	10	15	9	8	11	9	ST-269 complex	269	1	1	1
28217	M13 240165	UK [England]	2013	invasive (unspecified/other)	Neisseria meningitidis	Y	Y	Y	25	1	1	26	4	12	5	18	9	11	9	17	ST-23 complex	1655	4	4	2
27978	M13 240166	UK [England]	2013	invasive (unspecified/other)	Neisseria meningitidis	B	B	B	4	2	1	84	2	3	6	34	5	11	6	9	ST-41/44 complex	1194	2	3	1
27979	M13 240167	UK [England]	2013	invasive (unspecified/other)	Neisseria meningitidis	B	B	B	15	4	2	193	1	4	10	15	9	8	11	9	ST-269 complex	269	1	1	1
28144	M13 240168	UK [England]	2013	invasive (unspecified/other)	Neisseria meningitidis	W	W	W	22	1	1	1	4	2	3	4	3	8	4	6	ST-11 complex	11	4	4	2
27980	M13 240170	UK [England]	2013	invasive (unspecified/other)	Neisseria meningitidis	B	B	B	13	3	1	145	3	7	5	1	13	82	53	15	ST-213 complex	575	3	3	1
27981	M13 240171	UK [England]	2013	invasive (unspecified/other)	Neisseria meningitidis	B	B	B	4	2	79	84	2	3	206	9	5	9	6	9	ST-41/44 complex	5357	2	3	1
27982	M13 240172	UK [England]	2013	invasive (unspecified/other)	Neisseria meningitidis	B	B	B	19	1	1	83	4	4	10	2	5	38	11	9	ST-269 complex	275	4	4	2
27983	M13 240173	UK [England]	2013	invasive (unspecified/other)	Neisseria meningitidis	B	B	B	66	86	57	287	111	3	6	5	5	9	22	9	ST-41/44 complex	4256	NA	NA	1
27984	M13 240174	UK [England]	2013	invasive (unspecified/other)	Neisseria meningitidis	B	B	B	14	7	1	257	6	10	6	9	5	11	6	9	ST-41/44 complex	2203	6	2	1
27985	M13 240175	UK [England]	2013	invasive (unspecified/other)	Neisseria meningitidis	B	B	B	16	1	1	59	4	3	6	9	5	9	679	9	ST-41/44 complex	10535	4	4	2
28145	M13 240176	UK [England]	2013	invasive (unspecified/other)	Neisseria meningitidis	W	W	W	16	1	1	60	4	11	5	18	8	11	24	21	ST-22 complex	22	4	4	2
27986	M13 240181	UK [England]	2013	invasive (unspecified/other)	Neisseria meningitidis	B	B	B	4	2	79	84	2	3	6	9	5	9	6	9	ST-41/44 complex	41	2	3	1
27987	M13 240183	UK [England]	2013	invasive (unspecified/other)	Neisseria meningitidis	B	B	B	1	6	3	100	7	8	10	77	25	6	3	22		5151	7	1	1
28218	M13 240184	UK [England]	2013	invasive (unspecified/other)	Neisseria meningitidis	Y	Y	Y	25	1	1	26	4	12	5	18	9	11	9	17	ST-23 complex	1655	4	4	2
28146	M13 240185	UK [England]	2013	invasive (unspecified/other)	Neisseria meningitidis	W	W	W	22	1	1	1	4	662	3	4	3	8	4	6	ST-11 complex	10651	4	4	2
27988	M13 240187	UK [England]	2013	invasive (unspecified/other)	Neisseria meningitidis	B	B	B	45	14	77	87	45	7	5	1	13	36	53	15	ST-213 complex	213	NA	NA	3
28219	M13 240188	UK [England]	2013	invasive (unspecified/other)	Neisseria meningitidis	Y	Y	Y	25	1	1	26	4	12	5	18	9	11	9	17	ST-23 complex	1655	4	4	2
28095	M13 240189	UK [England]	2013	invasive (unspecified/other)	Neisseria meningitidis	C	C	C	13	16	57	145	63	2	3	4	3	8	4	6	ST-11 complex	11	NA	NA	1
27989	M13 240191	UK [England]	2013	invasive (unspecified/other)	Neisseria meningitidis	B	B	B	19	1	1	83	4	4	10	2	5	3	11	9	ST-269 complex	1163	4	4	2
27990	M13 240192	UK [England]	2013	invasive (unspecified/other)	Neisseria meningitidis	B	B	B	19	1	1	83	4	4	10	2	5	38	11	9	ST-269 complex	275	4	4	2
28220	M13 240194	UK [England]	2013	invasive (unspecified/other)	Neisseria meningitidis	Y	Y	Y	25	1	1	26	4	12	5	18	9	11	9	17	ST-23 complex	1655	4	4	2
27991	M13 240197	UK [England]	2013	invasive (unspecified/other)	Neisseria meningitidis	B	B	B	13	3	57	145	3	4	10	2	4	38	11	9	ST-269 complex	9829	3	3	1
28110	M13 240198	UK [England]	2013	invasive (unspecified/other)	Neisseria meningitidis	NG	B	B	4	2	6	84	2	3	6	724	5	9	6	9	ST-41/44 complex	10725	2	3	1
27992	M13 240199	UK [Wales]	2013	invasive (unspecified/other)	Neisseria meningitidis	B	B	B	95	5	89	32	11	2	3	4	3	8	4	6	ST-11 complex	11	11	2	2
28221	M13 240200	UK [England]	2013	invasive (unspecified/other)	Neisseria meningitidis	Y	Y	Y	25	1	1	26	4	12	5	18	9	11	9	17	ST-23 complex	1655	4	4	2
28096	M13 240201	UK [England]	2013	invasive (unspecified/other)	Neisseria meningitidis	C	C	C	19	1	2	65	4	9	4	9	17	5	6	360		5315	4	4	2
27993	M13 240202	UK [England]	2013	invasive (unspecified/other)	Neisseria meningitidis	B	B	B	13	3	57	145	3	12	10	2	5	38	11	9		7143	3	3	1
28222	M13 240203	UK [England]	2013	invasive (unspecified/other)	Neisseria meningitidis	Y	Y	Y	25	1	1	26	4	10	5	18	9	11	9	17	ST-23 complex	23	4	4	2
27994	M13 240204	UK [England]	2013	invasive (unspecified/other)	Neisseria meningitidis	B	B	B	608	2	1	277	2	3	6	34	5	11	6	9	ST-41/44 complex	1194	2	3	1
27995	M13 240205	UK [England]	2013	invasive (unspecified/other)	Neisseria meningitidis	B	B	B	4	2	1	84	2	3	6	9	5	8	6	9	ST-41/44 complex	485	2	3	1
27996	M13 240206	UK [England]	2013	invasive (unspecified/other)	Neisseria meningitidis	B	B	B	19	1	1	65	4	28	6	9	9	9	6	9	ST-41/44 complex	207	4	4	2
27997	M13 240207	UK [Wales]	2013	invasive (unspecified/other)	Neisseria meningitidis	B	B	B	13	3	57	145	3	4	10	34	5	38	11	9	ST-269 complex	1161	3	3	1
27998	M13 240210	UK [England]	2013	invasive (unspecified/other)	Neisseria meningitidis	B	B	B	4	NA	6	84	144	3	6	9	5	9	6	9	ST-41/44 complex	41	NA	NA	1
27999	M13 240211	UK [England]	2013	invasive (unspecified/other)	Neisseria meningitidis	B	B	B	13	3	57	145	3	4	5	34	5	38	11	9		5849	3	3	1
28000	M13 240212	UK [England]	2013	invasive (unspecified/other)	Neisseria meningitidis	B	B	B	24	1	63	25	4	5	6	9	3	9	6	16	ST-41/44 complex	3101	4	4	2
28001	M13 240213	UK [Northern Ireland]	2013	invasive (unspecified/other)	Neisseria meningitidis	B	B	B	13	3	57	145	3	4	10	2	5	38	11	9	ST-269 complex	275	3	3	1
28002	M13 240216	UK [Wales]	2013	invasive (unspecified/other)	Neisseria meningitidis	B	B	B	1	6	3	100	7	8	10	5	4	6	3	15	ST-32 complex	8049	7	1	1
28003	M13 240217	UK [England]	2013	invasive (unspecified/other)	Neisseria meningitidis	B	B	B	4	2	1	84	2	3	6	34	5	11	6	9	ST-41/44 complex	1194	2	3	1
28223	M13 240218	UK [England]	2013	invasive (unspecified/other)	Neisseria meningitidis	Y	Y	Y	25	1	1	26	4	12	5	18	9	11	9	17	ST-23 complex	1655	4	4	2
28224	M13 240220	UK [England]	2013	invasive (unspecified/other)	Neisseria meningitidis	Y	Y	Y	106	1	1	146	4	12	5	18	9	11	9	17	ST-23 complex	1655	4	4	2
28147	M13 240223	UK [England]	2013	invasive (unspecified/other)	Neisseria meningitidis	W	W	W	22	1	1	1	4	2	3	4	3	8	4	6	ST-11 complex	11	4	4	2
28004	M13 240224	UK [Wales]	2013	invasive (unspecified/other)	Neisseria meningitidis	B	B	B	13	3	57	145	3	4	10	34	25	38	25	49		10715	3	3	1
28148	M13 240225	UK [Wales]	2013	invasive (unspecified/other)	Neisseria meningitidis	W	W	W	22	1	1	1	4	662	3	4	3	8	4	6	ST-11 complex	10651	4	4	2
28225	M13 240226	UK [England]	2013	invasive (unspecified/other)	Neisseria meningitidis	Y	Y	Y	16	1	1	60	4	219	5	275	17	11	8	21		3015	4	4	2
28005	M13 240227	UK [Wales]	2013	invasive (unspecified/other)	Neisseria meningitidis	B	B	B	13	3	57	145	3	4	10	34	5	38	11	9	ST-269 complex	1161	3	3	1
28006	M13 240228	UK [England]	2013	invasive (unspecified/other)	Neisseria meningitidis	B	B	B	4	2	6	84	2	3	6	9	5	9	6	9	ST-41/44 complex	41	2	3	1
28007	M13 240229	UK [Wales]	2013	invasive (unspecified/other)	Neisseria meningitidis	B	B	B	283	48	19	861	92	4	10	5	26	6	3	8	ST-32 complex	1096	NA	NA	1
28008	M13 240230	UK [Wales]	2013	invasive (unspecified/other)	Neisseria meningitidis	B	B	B	1	6	3	100	7	8	10	5	4	6	3	15	ST-32 complex	8049	7	1	1
28009	M13 240231	UK [Wales]	2013	invasive (unspecified/other)	Neisseria meningitidis	B	B	B	4	2	1	84	2	3	6	15	5	247	6	9	ST-41/44 complex	8539	2	3	1
28010	M13 240232	UK [England]	2013	invasive (unspecified/other)	Neisseria meningitidis	B	B	B	603	27	77	95	26	7	5	1	13	36	53	15	ST-213 complex	213	NA	NA	3
28011	M13 240233	UK [England]	2013	invasive (unspecified/other)	Neisseria meningitidis	B	B	B	19	7	1	153	6	4	10	2	5	38	11	9	ST-269 complex	275	6	2	2
28012	M13 240234	UK [England]	2013	invasive (unspecified/other)	Neisseria meningitidis	B	B	B	4	2	79	84	2	3	6	9	5	9	6	9	ST-41/44 complex	41	2	3	1
28013	M13 240235	UK [Wales]	2013	invasive (unspecified/other)	Neisseria meningitidis	B	B	B	4	2	1	84	2	3	6	9	5	9	6	9	ST-41/44 complex	41	2	3	1
28014	M13 240237	UK [England]	2013	invasive (unspecified/other)	Neisseria meningitidis	B	B	B	4	NA	6	84	144	3	6	9	5	9	6	9	ST-41/44 complex	41	NA	NA	1
28149	M13 240238	UK [England]	2013	invasive (unspecified/other)	Neisseria meningitidis	W	W	W	22	1	1	1	4	2	3	4	3	8	4	6	ST-11 complex	11	4	4	2
28015	M13 240239	UK [Northern Ireland]	2013	invasive (unspecified/other)	Neisseria meningitidis	B	B	B	4	2	79	84	2	3	6	9	5	9	6	9	ST-41/44 complex	41	2	3	1

28111	M13 240240	UK [England]	2013	invasive (unspecified/other)	Neisseria meningitidis	NG	B	B	13	3	57	145	3	7	5	1	13	36	53	15	ST-213 complex	213	3	3	1
28226	M13 240242	UK [England]	2013	invasive (unspecified/other)	Neisseria meningitidis	Y	Y	Y	104	52	57	10	109	10	5	18	9	11	9	17	ST-23 complex	23	NA	NA	2
28227	M13 240244	UK [England]	2013	invasive (unspecified/other)	Neisseria meningitidis	Y	Y	Y	25	1	1	26	4	12	5	18	9	11	9	17	ST-23 complex	1655	4	4	2
28016	M13 240245	UK [England]	2013	invasive (unspecified/other)	Neisseria meningitidis	B	B	B	15	10	2	193	17	4	10	15	9	8	11	9	ST-269 complex	269	NA	NA	1
28150	M13 240246	UK [England]	2013	invasive (unspecified/other)	Neisseria meningitidis	W	W	W	22	1	1	1	4	2	3	4	3	8	4	6	ST-11 complex	11	4	4	2
28151	M13 240247	UK [England]	2013	invasive (unspecified/other)	Neisseria meningitidis	W	W	W	22	1	1	1	4	2	3	4	3	8	4	6	ST-11 complex	11	4	4	2
28017	M13 240248	UK [England]	2013	invasive (unspecified/other)	Neisseria meningitidis	B	B	B	690	1	57	291	15	12	5	12	35	192	22	17	ST-461 complex	1946	15	3	1
28018	M13 240249	UK [Northern Ireland]	2013	invasive (unspecified/other)	Neisseria meningitidis	B	B	B	45	8	77	87	20	7	2	1	13	36	53	15	ST-213 complex	10716	20	4	3
28019	M13 240250	UK [England]	2013	invasive (unspecified/other)	Neisseria meningitidis	B	B	B	4	2	1	84	2	6	7	6	17	26	21	8	ST-167 complex	840	2	3	1
28152	M13 240251	UK [England]	2013	invasive (unspecified/other)	Neisseria meningitidis	W	W	W	22	1	1	1	4	2	3	4	3	8	4	6	ST-11 complex	11	4	4	2
28097	M13 240254	UK [England]	2013	invasive (unspecified/other)	Neisseria meningitidis	C	C	C	13	5	1	251	11	2	3	4	3	8	4	6	ST-11 complex	11	11	2	1
28020	M13 240255	UK [England]	2013	invasive (unspecified/other)	Neisseria meningitidis	B	B	B	13	3	57	145	3	4	10	34	5	38	11	9	ST-269 complex	1161	3	3	1
28021	M13 240256	UK [England]	2013	invasive (unspecified/other)	Neisseria meningitidis	B	B	B	19	1	1	83	4	4	10	2	5	3	11	9	ST-269 complex	1163	4	4	2
28022	M13 240257	UK [England]	2013	invasive (unspecified/other)	Neisseria meningitidis	B	B	B	19	1	1	83	4	4	10	64	5	38	11	9	ST-269 complex	1273	4	4	2
28023	M13 240258	UK [England]	2013	invasive (unspecified/other)	Neisseria meningitidis	B	B	B	185	4	19	154	1	637	5	1	13	36	53	15	ST-213 complex	10338	1	1	3
28024	M13 240261	UK [England]	2013	invasive (unspecified/other)	Neisseria meningitidis	B	B	B	4	2	79	84	2	3	6	9	5	9	6	9	ST-41/44 complex	41	2	3	1
28025	M13 240262	UK [England]	2013	invasive (unspecified/other)	Neisseria meningitidis	B	B	B	316	27	23	155	26	4	10	5	4	6	53	8	ST-32 complex	10717	NA	NA	3
28026	M13 240263	UK [England]	2013	invasive (unspecified/other)	Neisseria meningitidis	B	B	B	4	2	6	84	2	3	6	9	NA	9	6	9		NA	2	3	1
28027	M13 240264	UK [England]	2013	invasive (unspecified/other)	Neisseria meningitidis	B	B	B	13	3	57	209	3	8	25	7	17	21	26	49	ST-1157 complex	1157	3	3	1
28228	M13 240265	UK [England]	2013	invasive (unspecified/other)	Neisseria meningitidis	Y	Y	Y	25	1	1	26	4	12	5	18	9	11	9	17	ST-23 complex	1655	4	4	2
28229	M13 240267	UK [England]	2013	invasive (unspecified/other)	Neisseria meningitidis	Y	Y	Y	25	1	1	26	4	12	5	18	9	11	9	17	ST-23 complex	1655	4	4	2
28230	M13 240268	UK [England]	2013	invasive (unspecified/other)	Neisseria meningitidis	Y	Y	Y	25	1	NA	26	4	10	5	18	9	11	9	22	ST-23 complex	10732	4	4	2
28153	M13 240269	UK [England]	2013	invasive (unspecified/other)	Neisseria meningitidis	W	W	W	22	1	1	1	4	2	3	4	3	8	4	6	ST-11 complex	11	4	4	2
28028	M13 240271	UK [England]	2013	invasive (unspecified/other)	Neisseria meningitidis	B	B	B	494	87	1	53	141	3	6	722	5	9	6	9	ST-41/44 complex	10718	NA	NA	3
28029	M13 240272	UK [England]	2013	invasive (unspecified/other)	Neisseria meningitidis	B	B	B	705	88	5	314	167	1	5	13	53	26	41	3	ST-162 complex	162	NA	NA	1
28030	M13 240273	UK [England]	2013	invasive (unspecified/other)	Neisseria meningitidis	B	B	B	4	2	1	84	2	3	6	34	5	11	6	9	ST-41/44 complex	1194	2	3	1
28031	M13 240274	UK [England]	2013	invasive (unspecified/other)	Neisseria meningitidis	B	B	B	15	4	2	193	1	4	10	15	17	8	11	9	ST-269 complex	1049	1	1	1
28032	M13 240275	UK [England]	2013	invasive (unspecified/other)	Neisseria meningitidis	B	B	B	4	2	1	84	2	3	6	9	5	9	6	9	ST-41/44 complex	41	2	3	1
28033	M13 240277	UK [England]	2013	invasive (unspecified/other)	Neisseria meningitidis	B	B	B	327	1	57	315	4	3	6	9	5	9	6	156	ST-41/44 complex	1823	4	3	1
28098	M13 240280	UK [England]	2013	invasive (unspecified/other)	Neisseria meningitidis	C	C	C	78	5	1	245	11	2	3	4	3	8	4	6	ST-11 complex	11	11	2	1
28034	M13 240281	UK [England]	2013	invasive (unspecified/other)	Neisseria meningitidis	B	B	B	276	26	77	316	100	7	5	1	13	36	691	15	ST-213 complex	10719	NA	NA	1
28099	M13 240282	UK [England]	2013	invasive (unspecified/other)	Neisseria meningitidis	C	C	C	13	5	1	251	11	2	3	4	3	8	4	6	ST-11 complex	11	11	2	1
28154	M13 240283	UK [England]	2013	invasive (unspecified/other)	Neisseria meningitidis	W	W	W	22	1	1	1	4	2	3	4	3	8	4	6	ST-11 complex	11	4	4	2
28035	M13 240289	UK [England]	2013	invasive (unspecified/other)	Neisseria meningitidis	B	B	B	200	1	1	16	4	663	290	15	9	8	11	9	ST-269 complex	10720	4	4	2
28036	M13 240292	UK [England]	2013	invasive (unspecified/other)	Neisseria meningitidis	B	B	B	14	1	1	142	13	8	6	19	5	3	18	9		5330	13	3	1
28037	M13 240294	UK [England]	2013	invasive (unspecified/other)	Neisseria meningitidis	B	B	B	19	18	1	83	87	4	10	2	201	38	11	9	ST-269 complex	6791	NA	NA	2
28100	M13 240295	UK [England]	2013	invasive (unspecified/other)	Neisseria meningitidis	C	C	C	13	5	1	251	11	2	3	4	3	8	4	6	ST-11 complex	11	11	2	1
28231	M13 240381	UK [England]	2013	invasive (unspecified/other)	Neisseria meningitidis	Y	Y	Y	25	1	1	26	4	10	5	18	9	11	9	17	ST-23 complex	23	4	4	2
28232	M13 240383	UK [England]	2013	invasive (unspecified/other)	Neisseria meningitidis	Y	Y	Y	706	1	1	319	4	8	5	6	17	8	31	8	ST-865 complex	3342	4	3	1
28038	M13 240384	UK [England]	2013	invasive (unspecified/other)	Neisseria meningitidis	B	B	B	69	45	1	248	64	4	10	5	4	6	3	8	ST-32 complex	32	NA	NA	1
28039	M13 240385	UK [England]	2013	invasive (unspecified/other)	Neisseria meningitidis	B	B	B	4	2	1	84	2	3	6	9	5	11	6	2	ST-41/44 complex	8176	2	3	1
28040	M13 240386	UK [England]	2013	invasive (unspecified/other)	Neisseria meningitidis	B	B	B	87	4	1	234	1	4	10	2	5	38	11	1		1159	1	1	1
28233	M13 240396	UK [England]	2013	invasive (unspecified/other)	Neisseria meningitidis	Y	Y	Y	25	1	1	26	4	10	208	18	17	317	9	17	ST-23 complex	4446	4	4	2
28041	M13 240397	UK [England]	2013	invasive (unspecified/other)	Neisseria meningitidis	B	B	B	291	20	4	203	8	4	10	47	17	6	2	9		1575	NA	NA	1
28101	M13 240398	UK [England]	2013	invasive (unspecified/other)	Neisseria meningitidis	C	C	C	15	4	2	193	1	9	4	9	17	5	6	2	ST-103 complex	5133	1	1	1
28112	M13 240399	UK [England]	2013	invasive (unspecified/other)	Neisseria meningitidis	NG	B	B	4	2	6	84	2	3	6	9	5	9	6	9	ST-41/44 complex	41	2	3	1
28042	M13 240400	UK [England]	2013	invasive (unspecified/other)	Neisseria meningitidis	B	B	B	15	4	2	193	1	4	10	15	9	8	11	9	ST-269 complex	269	1	1	1
28102	M13 240401	UK [England]	2013	invasive (unspecified/other)	Neisseria meningitidis	C	C	C	22	1	1	1	4	2	3	4	3	8	4	6	ST-11 complex	11	4	4	2
28103	M13 240402	UK [England]	2013	invasive (unspecified/other)	Neisseria meningitidis	C	C	C	13	5	1	251	11	2	3	4	3	8	4	6	ST-11 complex	11	11	2	1
28104	M13 240405	UK [England]	2013	invasive (unspecified/other)	Neisseria meningitidis	C	C	C	208	5	1	318	11	2	3	4	3	8	4	6	ST-11 complex	11	11	2	1
28043	M13 240406	UK [England]	2013	invasive (unspecified/other)	Neisseria meningitidis	B	B	B	47	1	57	90	15	12	5	12	35	192	22	17	ST-461 complex	1946	15	4	3
28234	M13 240407	UK [England]	2013	invasive (unspecified/other)	Neisseria meningitidis	Y	Y	Y	25	1	1	26	4	12	5	18	9	11	9	17	ST-23 complex	1655	4	4	2
28044	M13 240409	UK [England]	2013	invasive (unspecified/other)	Neisseria meningitidis	B	B	B	4	2	1	84	2	3	6	9	5	9	6	9	ST-41/44 complex	41	2	3	1
28113	M13 240410	UK [England]	2013	invasive (unspecified/other)	Neisseria meningitidis	NG	E	E	13	3	57	209	3	8	25	7	17	21	26	49	ST-1157 complex	1157	3	3	1
28045	M13 240411	UK [Wales]	2013	invasive (unspecified/other)	Neisseria meningitidis	B	B	B	15	4	2	193	1	4	10	15	9	8	11	17	ST-269 complex	283	1	1	1
28046	M13 240413	UK [England]	2013	invasive (unspecified/other)	Neisseria meningitidis	B	B	B	4	2	1	84	2	3	6	9	5	9	7	46	ST-41/44 complex	10721	2	3	1
28047	M13 240414	UK [England]	2013	invasive (unspecified/other)	Neisseria meningitidis	B	B	B	21	1	1	7	4	1	5	13	53	26	41	3	ST-162 complex	162	4	4	2
28048	M13 240417	UK [England]	2013	invasive (unspecified/other)	Neisseria meningitidis	B	B	B	13	3	1	145	3	17	5	47	17	3	26	2	ST-60 complex	1430	3	3	1
28049	M13 240418	UK [England]	2013	invasive (unspecified/other)	Neisseria meningitidis	B	B	B	4	2	1	84	2	3	6	9	5	3	6	9	ST-41/44 complex	46	2	3	1
28050	M13 240421	UK [England]	2013	invasive (unspecified/other)	Neisseria meningitidis	B	B	B	15	4	2	193	1	4	10	15	17	8	11	9	ST-269 complex	1049	1	1	1
28051	M13 240422	UK [England]	2013	invasive (unspecified/other)	Neisseria meningitidis	B	B	B	19	1	1	83	4	4	10	2	5	3	11	9	ST-269 complex	1163	4	4	2
28052	M13 240424	UK [England]	2013	invasive (unspecified/other)	Neisseria meningitidis	B	B	B	37	1	1	408	4	7	8	723	26	58	1	16		10722	4	3	1
28053	M13 240426	UK [England]	2013	invasive (unspecified/other)	Neisseria meningitidis	B	B	B	13	3	57	145	3	4	5	34	5	38	11	9		5849	3	3	1
28054	M13 240427	UK [Wales]	2013	invasive (unspecified/other)	Neisseria meningitidis	B	B	B	15	4	5	193	1	7	5	1	13	36	53	15	ST-213 complex	213	1	1	1
28055	M13 240428	UK [England]	2013	invasive (unspecified/other)	Neisseria meningitidis	B	B	B	25	1	1	26	4	4	10	2	5	38	11	9	ST-269 complex	275	4	4	2
28056	M13 240429	UK [England]	2013	invasive (unspecified/other)	Neisseria meningitidis	B	B	B	15	4	2	193	1	4	10	15	9	8	11	9	ST-269 complex	269	1	1	1
28155	M13 240431	UK [England]	2013	invasive (unspecified/other)	Neisseria meningitidis	W	W	W	16	1	1	60	4	11	445	18	8	11	4	21	ST-22 complex	10726	4	4	2
28057	M13 240434	UK [England]	2013	invasive (unspecified/other)	Neisseria meningitidis	B	B	B	13	3	57	145	3	2	16	12	11	3	60	7	ST-254 complex	254	3	3	1
28156	M13 240436	UK [England]	2013	invasive (unspecified/other)	Neisseria meningitidis	W	W	W	22	1	1	1	4	2	3	4	3	8	4	6	ST-11 complex	11	4	4	2
28058	M13 240437	UK [England]	2013	invasive (unspecified/other)	Neisseria meningitidis	B	B	B	45	89	77	87	168	7	5	1	13	36	53	15	ST-213 complex	213	NA	NA	3
28059	M13 240438	UK [England]	2013	invasive (unspecified/other)	Neisseria meningitidis	B	B	B	19	1	1	65	4	9	6	18	9	9	6	9	ST-41/44 complex	7955	4	4	2
28235	M13 240439	UK [England]	2013	invasive (unspecified/other)	Neisseria meningitidis	Y	Y	Y	25	1	1	26	4	12	5	18	9	11	9	17	ST-23 complex	1655	4	4	2
28105	M13 240440	UK [England]	2013	invasive (unspecified/other)	Neisseria meningitidis	C	C	C	13	5	1	251	11	2	3	4	3	8	4	6	ST-11 complex	11	11	2	1
28157	M13 240446	UK [England]	2013	invasive (unspecified/other)	Neisseria meningitidis	W	W	W	22	1	1	1	4	2	3	4	3	8	4	6	ST-11 complex	11	4	4	2
28060	M13 240448	UK [England]	2013	invasive (unspecified/other)	Neisseria meningitidis	B	B	B	25	1	1	26	4	8	4	6	17	5	18	2	ST-103 complex	103	4	4	2
28061	M13 240449	UK [England]	2013	invasive (unspecified/other)	Neisseria meningitidis	B	B	B	4	2	1	84	2	3	6	9	5	11	6	9	ST-41/44 complex	154	2	3	1
28062	M13 240450	UK [England]	2013	invasive (unspecified/other)	Neisseria meningitidis	B	B	B	13	3	57	145	3	4	10	34	5	38	11	9	ST-269 complex	1161	3	3	1
28063	M13 240451	UK [England]	2013	invasive (unspecified/other)	Neisseria meningitidis	B	B	B	357	4	12	208	1	7	5	1	13	36	53	9	ST-213 complex	3120	1	1	1
28064	M13 240452	UK [England]	2013	invasive (unspecified/other)	Neisseria meningitidis	B	B	B	4	2	1	84	2	3	6	9	5	9	6	9	ST-41/44 complex	41	2	3	1
28065	M13 240453	UK [England]	2013	invasive (unspecified/other)	Neisseria meningitidis	B	B	B	15	4	2	193	1	4	10	15	9	8	11	9	ST-269 complex	269	1	1	1
28066	M13 240455	UK [England]	2013	invasive (unspecified/other)	Neisseria meningitidis	B	B	B	19	1	1	83	4	4	10	2	5	38	11	9	ST-269 complex	275	4	4	2
28158	M13 240457	UK [England]	2013	invasive (unspecified/other)	Neisseria meningitidis	W	W	W	22	1	1	1	4	2	3	4	3	8	4	6	ST-11 complex	11	4	4	2
28067	M13 240458	UK [England]	2013	invasive (unspecified/other)	Neisseria meningitidis	B	B	B	25	1	1	26	4	8	4	6	17	5	18	2	ST-103 complex	103	4	4	2
28106	M13 240460	UK [England]	2013	invasive (unspecified/other)	Neisseria meningitidis	C	C	C	22	1	1	1	4	2	3	4	3	8	4	6	ST-11 complex	11	4	4	2
28068	M13 240461	UK [England]	2013	invasive (unspecified/other)	Neisseria meningitidis	B	B	B	14	1	1	142	13	4	10	2	5	38	11	1		1159	13	3	1
28069	M13 240463	UK [England]	2013	invasive (unspecified/other)	Neisseria meningitidis	B	B	B	13	3	57	145	3	4	10	34	5	38	11	9	ST-269 complex	1161	3	3	1
28159	M13 240464	UK [Northern Ireland]	2013	invasive (unspecified/other)	Neisseria meningitidis	W	W	W	22	1	1	1	4	2	3	4	3	8	4	6	ST-11 complex	11	4	4	2
28160	M13 240465	UK [England]	2013	invasive (unspecified/other)	Neisseria meningitidis	W	W	W	16	1	1	60	4	11	5	18	8	11	4	21	ST-22 complex	184	4	4	2
28070	M13 240466	UK [England]	2013	invasive (unspecified/other)	Neisseria meningitidis	B	B	B	13	3	57	145	3	4	10	2	5	38	11	9	ST-269 complex	275	3	3	1
28161	M13 240467	UK [England]	2013	invasive (unspecified/other)	Neisseria meningitidis	W	W	W	22	1	1	1	4	2	3	4	3	8	4	6	ST-11 complex	11	4	4	2
28071	M13 240468	UK [England]	2013	invasive (unspecified/other)	Neisseria meningitidis	B	B	B	203	44	13	17	62	1	5	13	53	26	41	3	ST-162 complex	162	NA	NA	2
28162	M13 240469	UK [England]	2013	invasive (unspecified/other)	Neisseria meningitidis	W	W	W	1	6	1	100	7	2	3	4	3	8	4	6	ST-11 complex	11	7	1	1
28072	M13 240472	UK [England]	2013	invasive (unspecified/other)	Neisseria meningitidis	B	B	B	4	2	1	84	2	3	6	9	5	9	6	9	ST-41/44 complex	41	2	3	1
28163	M13 240473	UK [England]	2013	invasive (unspecified/other)	Neisseria meningitidis	W	W	W	22	1	1	1	4	2	3	4	3	8	4	6	ST-11 complex	11	4	4	2
28073	M13 240475	UK [England]	2013	invasive (unspecified/other)	Neisseria meningitidis	B	B	B	19	1	57	65	4	3	6	9	5	9	18	9	ST-41/44 complex	2080	4	4	2
28074	M13 240476	UK [England]	2013	invasive (unspecified/other)	Neisseria meningitidis	B	B	B	13	3	57	145	3	4	10	34	5	38	11	9	ST-269 complex	1161	3	3	1
28075	M13 240478	UK [England]	2013	invasive (unspecified/other)	Neisseria meningitidis	B	B	B	13	3	57	145	3	4	10	34	5	38	11	9	ST-269 complex	1161	3	3	1
28076	M13 240479	UK [England]	2013	invasive (unspecified/other)	Neisseria meningitidis	B	B	B	4	NA	6	84	144	3	6	9	5	9	6	9	ST-41/44 complex	41	NA	NA	1
28077	M13 240481	UK [England]	2013	invasive (unspecified/other)	Neisseria meningitidis	B	B	B	364	12	1	156	28	664	5	1	13	36	6	15	ST-213 complex	10723	NA	NA	3
28164	M13 240482	UK [England]	2013	invasive (unspecified/other)	Neisseria meningitidis	W	W	W	22	1	1	1	4	2	3	4	3	8	4	6	ST-11 complex	11	4	4	2
28078	M13 240483	UK [England]	2013	invasive (unspecified/other)	Neisseria meningitidis	B	B	B	19	1	1	83	4	4	10	9	5	3	11	9	ST-269 complex	10724	4	4	2
34502	M13 240484	UK [England]	2013	invasive (unspecified/other)	Neisseria meningitidis	Y	Y	Y	25	1	1	26	4	12	5	18	9	11	9	17	ST-23 complex	1655	4	4	2
34503	M13 240485	UK [England]	2013	invasive (unspecified/other)	Neisseria meningitidis	Y		Y	25	1	1	26	4	12	5	18	9	11	9	17	ST-23 complex	1655	4	4	2
34504	M13 240486	UK [England]	2013	invasive (unspecified/other)	Neisseria meningitidis	B	B	B	15	4	2	193	1	4	10	15	9	8	11	9	ST-269 complex	269	1	1	1
34505	M13 240488	UK [England]	2013	invasive (unspecified/other)	Neisseria meningitidis	B	B	B	29	12	118	49	76	4	10	5	4	6	3	2	ST-32 complex	343	NA	NA	3
34506	M13 240490	UK [England]	2013	invasive (unspecified/other)	Neisseria meningitidis	B	B	B	13	3	57	145	3	4	10	2	405	38	11	9	ST-269 complex	5972	3	3	1
29721	M13 240491	UK [England]	2013	invasive (unspecified/other)	Neisseria meningitidis	W	W	W	22	1	1	1	4	2	3	4	3	8	4	6	ST-11 complex	11	4	4	2
34507	M13 240495	UK [England]	2013	invasive (unspecified/other)	Neisseria meningitidis	B	B	B	4	2	1	84	2	3	6	9	5	9	6	9	ST-41/44 complex	41	2	3	1
29895	M13 240496	UK [Northern Ireland]	2013	invasive (unspecified/other)	Neisseria meningitidis	C	C	C	22	1	1	1	4	2	3	4	3	8	4	6	ST-11 complex	11	4	4	2
34508	M13 240497	UK [England]	2013	invasive (unspecified/other)	Neisseria meningitidis	B	B	B	683	4	2	176	1	9	5	9	9	9	6	2	ST-41/44 complex	1423	1	1	2
34509	M13 240499	UK [Wales]	2013	invasive (unspecified/other)	Neisseria meningitidis	B	B	B	15	4	2	193	1	4	10	15	9	9	11	9	ST-269 complex	1855	1	1	1
29897	M13 240500	UK [England]	2013	invasive (unspecified/other)	Neisseria meningitidis	C	C	C	0	5	1	917	11	2	3	4	3	8	4	6	ST-11 complex	11	11	2	NA
34510	M13 240501	UK [England]	2013	invasive (unspecified/other)	Neisseria meningitidis	B	B	B	4	NA	1	84	NA	3	6	34	5	11	6	9	ST-41/44 complex	1194	NA	NA	1
34511	M13 240502	UK [England]	2013	invasive (unspecified/other)	Neisseria meningitidis	B	B	B	1	6	7	100	7	4	10	5	4	6	3	8	ST-32 complex	32	7	1	1
34512	M13 240503	UK [England]	2013	invasive (unspecified/other)	Neisseria meningitidis	W		W	16	1	1	60	4	11	5	18	8	11	4	21	ST-22 complex	184	4	4	2
34513	M13 240507	UK [England]	2013	invasive (unspecified/other)	Neisseria meningitidis	B	B	B	296	90	2	177	171	6	5	7	474	21	24	17	ST-174 complex	9126	NA	NA	2
34514	M13 240508	UK [England]	2013	invasive (unspecified/other)	Neisseria meningitidis	B	B	B	702	31	2	281	140	2	10	15	9	8	11	17	ST-269 complex	1214	NA	NA	1
29731	M13 240510	UK [England]	2013	invasive (unspecified/other)	Neisseria meningitidis	W	W	W	22	1	1	1	4	2	3	4	3	8	4	6	ST-11 complex	11	4	4	2
29898	M13 240514	UK [England]	2013	invasive (unspecified/other)	Neisseria meningitidis	C	C	C	22	1	1	1	4	2	3	4	3	8	4	6	ST-11 complex	11	4	4	2
29735	M13 240515	UK [England]	2013	invasive (unspecified/other)	Neisseria meningitidis	W	W	W	22	1	1	1	4	2	3	4	3	8	4	6	ST-11 complex	11	4	4	2
34515	M13 240516	UK [England]	2013	invasive (unspecified/other)	Neisseria meningitidis	B	B	B	1	6	3	100	7	8	10	77	4	6	3	8	ST-32 complex	749	7	1	1
34516	M13 240519	UK [England]	2013	invasive (unspecified/other)	Neisseria meningitidis	B	B	B	19	1	77	65	4	7	5	1	13	36	53	15	ST-213 complex	213	4	4	2
34517	M13 240525	UK [England]	2013	invasive (unspecified/other)	Neisseria meningitidis	B	B	B	4	2	1	84	2	3	6	9	5	9	6	9	ST-41/44 complex	41	2	3	1
35422	M13 240526	UK [England]	2013	invasive (unspecified/other)	Neisseria meningitidis	B	B	B	16	1	4	59	4	132	3	19	NA	216	21	2		NA	4	4	2
35423	M13 240528	UK [England]	2013	invasive (unspecified/other)	Neisseria meningitidis	W	W	W	16	1	1	60	4	11	5	18	8	11	4	21	ST-22 complex	184	4	4	2
35424	M13 240529	UK [England]	2013	invasive (unspecified/other)	Neisseria meningitidis	Y	Y	Y	25	1	1	26	4	12	5	18	9	11	9	17	ST-23 complex	1655	4	4	2
29722	M13 240530	UK	2013	invasive (unspecified/other)	Neisseria meningitidis	W	W	W	22	1	1	1	4	2	3	4	3	8	4	6	ST-11 complex	11	4	4	2
35425	M13 240531	UK [England]	2013	invasive (unspecified/other)	Neisseria meningitidis	W	W	W	22	1	1	1	4	2	3	4	3	8	4	6	ST-11 complex	11	4	4	2
35426	M13 240532	UK [England]	2013	invasive (unspecified/other)	Neisseria meningitidis	B	B	B	4	2	1	84	2	12	6	18	8	9	6	9	ST-41/44 complex	11225	2	3	1
35427	M13 240534	UK [England]	2013	invasive (unspecified/other)	Neisseria meningitidis	B	B	B	830	97	57	609	138	12	5	12	35	192	22	17	ST-461 complex	1946	NA	NA	1
35428	M13 240535	UK [England]	2013	invasive (unspecified/other)	Neisseria meningitidis	B	B	B	1	6	3	100	7	8	10	5	4	5	3	8	ST-32 complex	34	7	1	1
35429	M13 240536	UK [England]	2013	invasive (unspecified/other)	Neisseria meningitidis	B	B	B	19	1	1	83	4	4	10	2	5	3	11	9	ST-269 complex	1163	4	4	2
29734	M13 240538	UK [England]	2013	invasive (unspecified/other)	Neisseria meningitidis	W	W	W	22	1	1	1	4	2	3	4	3	8	4	6	ST-11 complex	11	4	4	2
35430	M13 240540	UK [England]	2013	invasive (unspecified/other)	Neisseria meningitidis	B	B	B	19	1	1	83	4	4	10	2	5	38	11	9	ST-269 complex	275	4	4	2
35431	M13 240542	UK [Northern Ireland]	2013	invasive (unspecified/other)	Neisseria meningitidis	B	B	B	45	8	77	87	20	2	5	1	13	36	25	15	ST-213 complex	11226	20	4	3
35432	M13 240543	UK [England]	2013	invasive (unspecified/other)	Neisseria meningitidis	Y	Y	Y	25	1	1	26	4	12	5	18	9	11	9	17	ST-23 complex	1655	4	4	2
29727	M13 240545	UK [Wales]	2013	invasive (unspecified/other)	Neisseria meningitidis	W	W	W	22	1	1	1	4	2	3	4	3	8	4	6	ST-11 complex	11	4	4	2
38122	M13 240546	UK [England]	2013	invasive (unspecified/other)	Neisseria meningitidis	B	NG	discrepancy	31	9	1	124	16	7	5	1	13	36	53	15	ST-213 complex	213	16	2	3

35434	M13 240548	UK [England]	2013	invasive (unspecified/other)	Neisseria meningitidis	B	B	B	19	1	1	456	142	3	6	9	9	9	6	9	ST-41/44 complex	303	NA	NA	2
35435	M13 240549	UK [England]	2013	invasive (unspecified/other)	Neisseria meningitidis	Y	Y	Y	25	1	1	26	4	12	5	18	9	11	9	17	ST-23 complex	1655	4	4	2
29737	M13 240553	UK [England]	2013	invasive (unspecified/other)	Neisseria meningitidis	W	W	W	22	1	1	1	4	2	3	4	3	8	4	6	ST-11 complex	11	4	4	2
35436	M13 240554	UK [England]	2013	invasive (unspecified/other)	Neisseria meningitidis	B	B	B	47	1	1	90	15	12	5	12	35	60	22	17	ST-461 complex	461	15	4	3
29723	M13 240555	UK [England]	2013	invasive (unspecified/other)	Neisseria meningitidis	W	W	W	767	4	2	30	1	2	3	4	3	8	4	6	ST-11 complex	11	1	1	2
35421	M13 240557	UK	2013	invasive (unspecified/other)	Neisseria meningitidis	B	B	B	31	9	1	124	16	7	5	1	NA	36	53	15		NA	16	2	3
29728	M13 240558	UK [England]	2013	invasive (unspecified/other)	Neisseria meningitidis	W	W	W	22	1	1	1	4	2	3	4	3	8	4	6	ST-11 complex	11	4	4	2
26732	M13 240559	UK [England]	2013	invasive (unspecified/other)	Neisseria meningitidis	C	C	C	13	16	57	145	63	2	3	4	3	8	4	6	ST-11 complex	11	NA	NA	1
35437	M13 240560	UK [England]	2013	invasive (unspecified/other)	Neisseria meningitidis	B	B	B	525	98	1	457	122	9	6	9	1	8	6	6	ST-41/44 complex	7516	NA	NA	1
35438	M13 240561	UK [England]	2013	invasive (unspecified/other)	Neisseria meningitidis	B	B	B	4	2	1	84	2	3	6	9	5	9	6	49	ST-41/44 complex	11227	2	3	1
35439	M13 240562	UK [England]	2013	invasive (unspecified/other)	Neisseria meningitidis	B	B	B	19	1	1	83	4	4	10	2	5	3	11	9	ST-269 complex	1163	4	4	2
35440	M13 240564	UK [England]	2013	invasive (unspecified/other)	Neisseria meningitidis	B	B	B	4	2	1	84	2	3	6	9	17	721	6	9	ST-41/44 complex	11229	2	3	1
35441	M13 240565	UK [England]	2013	invasive (unspecified/other)	Neisseria meningitidis	B	B	B	15	4	2	193	1	4	10	15	9	8	11	17	ST-269 complex	283	1	1	1
35442	M13 240566	UK [Wales]	2013	invasive (unspecified/other)	Neisseria meningitidis	B	B	B	45	8	77	87	20	7	5	1	13	36	53	15	ST-213 complex	213	20	4	3
35443	M13 240568	UK [England]	2013	invasive (unspecified/other)	Neisseria meningitidis	B	B	B	15	4	2	193	1	4	10	15	9	8	11	9	ST-269 complex	269	1	1	1
35444	M13 240572	UK [England]	2013	invasive (unspecified/other)	Neisseria meningitidis	B	B	B	831	99	1	458	177	4	10	15	9	8	11	249	ST-269 complex	11237	NA	NA	1
35445	M13 240573	UK [England]	2013	invasive (unspecified/other)	Neisseria meningitidis	Y	Y	Y	25	1	1	26	4	12	5	18	25	11	9	17	ST-23 complex	9842	4	4	2
35446	M13 240574	UK [England]	2013	invasive (unspecified/other)	Neisseria meningitidis	Y	Y	Y	25	1	1	26	4	12	5	18	9	11	9	17	ST-23 complex	1655	4	4	2
35447	M13 240579	UK [England]	2013	invasive (unspecified/other)	Neisseria meningitidis	B	B	B	15	4	2	193	1	4	10	6	9	21	732	9	ST-269 complex	11238	1	1	1
35448	M13 240581	UK [England]	2013	invasive (unspecified/other)	Neisseria meningitidis	B	B	B	47	1	57	90	15	12	5	12	35	192	22	17	ST-461 complex	1946	15	4	3
29730	M13 240583	UK [England]	2013	invasive (unspecified/other)	Neisseria meningitidis	W	W	W	22	1	1	1	4	662	3	4	3	8	7	6	ST-11 complex	11161	4	4	2
35449	M13 240588	UK [England]	2013	invasive (unspecified/other)	Neisseria meningitidis	W/Y	W/Y	W/Y	24	1	1	27	4	2	7	6	17	9	6	8	ST-167 complex	1624	4	4	2
35450	M13 240590	UK [England]	2013	invasive (unspecified/other)	Neisseria meningitidis	Y	Y	Y	25	1	1	26	4	12	286	18	NA	11	9	17		NA	4	4	2
29724	M13 240591	UK [England]	2013	invasive (unspecified/other)	Neisseria meningitidis	W	W	W	22	1	1	1	4	2	3	4	3	8	4	6	ST-11 complex	11	4	4	2
35451	M13 240592	UK [Wales]	2013	invasive (unspecified/other)	Neisseria meningitidis	B	B	B	13	3	57	145	3	4	10	768	13	38	11	9	ST-269 complex	11239	3	3	1
35452	M13 240593	UK [England]	2013	invasive (unspecified/other)	Neisseria meningitidis	B	B	B	21	4	1	7	178	1	5	13	53	26	41	3	ST-162 complex	162	NA	NA	2
29729	M13 240594	UK [England]	2013	invasive (unspecified/other)	Neisseria meningitidis	W	W	W	22	1	1	1	4	2	3	4	3	8	4	6	ST-11 complex	11	4	4	2
35453	M13 240595	UK [England]	2013	invasive (unspecified/other)	Neisseria meningitidis	W	W	W	16	1	1	60	4	11	5	18	8	11	4	21	ST-22 complex	184	4	4	2
35454	M13 240596	UK [England]	2013	invasive (unspecified/other)	Neisseria meningitidis	B	B	B	15	4	2	193	1	4	10	15	9	8	11	9	ST-269 complex	269	1	1	1
95906	M13 240598	UK [England]	2013	invasive (unspecified/other)	Neisseria meningitidis	Y	Y	Y	24	1	1	27	4	2	7	6	17	9	6	8	ST-167 complex	1624	4	4	2
35456	M13 240599	UK [Northern Ireland]	2013	invasive (unspecified/other)	Neisseria meningitidis	Y	Y	Y	293	23	1	114	49	10	5	18	9	11	9	17	ST-23 complex	23	NA	NA	3
29736	M13 240600	UK [England]	2013	invasive (unspecified/other)	Neisseria meningitidis	W	W	W	22	1	1	1	4	2	3	4	3	8	4	6	ST-11 complex	11	4	4	2
35457	M13 240601	UK [England]	2013	invasive (unspecified/other)	Neisseria meningitidis	B	B	B	4	37	1	84	125	3	6	9	5	9	6	9	ST-41/44 complex	41	NA	NA	1
29725	M13 240603	UK [England]	2013	invasive (unspecified/other)	Neisseria meningitidis	W	W	W	22	1	1	1	4	2	3	4	3	8	4	6	ST-11 complex	11	4	4	2
29726	M13 240604	UK [England]	2013	invasive (unspecified/other)	Neisseria meningitidis	W	W	W	22	1	1	1	4	662	3	4	3	8	4	6	ST-11 complex	10651	4	4	2
35458	M13 240605	UK [Wales]	2013	invasive (unspecified/other)	Neisseria meningitidis	B	B	B	13	3	57	145	3	4	35	34	5	38	11	9		11328	3	3	1
35459	M13 240608	UK [England]	2013	invasive (unspecified/other)	Neisseria meningitidis	B	B	B	4	2	1	84	2	12	6	9	9	9	6	193	ST-41/44 complex	2821	2	3	1
35460	M13 240610	UK [England]	2013	invasive (unspecified/other)	Neisseria meningitidis	Y	Y	Y	25	1	1	26	4	12	5	18	9	11	9	17	ST-23 complex	1655	4	4	2
35461	M13 240611	UK [England]	2013	invasive (unspecified/other)	Neisseria meningitidis	B	B	B	19	1	1	83	4	4	10	2	5	3	11	9	ST-269 complex	1163	4	4	2
35462	M13 240613	UK [England]	2013	invasive (unspecified/other)	Neisseria meningitidis	B	B	B	4	2	1	84	2	3	6	9	5	9	6	2	ST-41/44 complex	2314	2	3	1
35463	M13 240614	UK [England]	2013	invasive (unspecified/other)	Neisseria meningitidis	B	B	B	4	1	59	84	4	686	6	9	5	9	21	9	ST-41/44 complex	10868	4	3	1
35464	M13 240615	UK [Wales]	2013	invasive (unspecified/other)	Neisseria meningitidis	B	B	B	25	1	1	28	4	8	6	NA	5	106	18	9		NA	4	4	2
35465	M13 240618	UK [England]	2013	invasive (unspecified/other)	Neisseria meningitidis	B	B	B	15	4	2	193	1	2	10	15	NA	NA	11	17		NA	1	1	1
35466	M13 240619	UK [England]	2013	invasive (unspecified/other)	Neisseria meningitidis	B	B	B	89	5	5	213	23	7	5	1	13	36	53	15	ST-213 complex	213	NA	NA	1
35467	M13 240620	UK [England]	2013	invasive (unspecified/other)	Neisseria meningitidis	B	B	B	4	NA	1	84	NA	3	6	34	5	11	6	9	ST-41/44 complex	1194	NA	NA	1
35468	M13 240622	UK [England]	2013	invasive (unspecified/other)	Neisseria meningitidis	Y	Y	Y	25	1	1	26	4	12	5	18	9	11	9	17	ST-23 complex	1655	4	4	2
35469	M13 240623	UK [England]	2013	invasive (unspecified/other)	Neisseria meningitidis	B	B	B	4	2	1	84	2	4	10	11	18	6	10	12	ST-35 complex	35	2	3	1
35470	M13 240624	UK [England]	2013	invasive (unspecified/other)	Neisseria meningitidis	B	B	B	4	2	1	84	2	503	6	9	5	9	6	9	ST-41/44 complex	8380	2	3	1
35471	M13 240626	UK [England]	2013	invasive (unspecified/other)	Neisseria meningitidis	B	B	B	4	2	1	84	2	3	6	9	5	9	6	9	ST-41/44 complex	41	2	3	1
35472	M13 240627	UK [England]	2013	invasive (unspecified/other)	Neisseria meningitidis	Y	Y	Y	24	1	1	27	4	2	7	6	17	9	6	8	ST-167 complex	1624	4	4	2
35473	M13 240632	UK [Wales]	2013	invasive (unspecified/other)	Neisseria meningitidis	B	B	B	187	5	1	101	11	7	25	1	13	9	26	49		11243	11	2	3
29732	M13 240633	UK [England]	2013	invasive (unspecified/other)	Neisseria meningitidis	W	W	W	22	1	1	1	4	2	3	4	3	8	4	6	ST-11 complex	11	4	4	2
29733	M13 240634	UK [England]	2013	invasive (unspecified/other)	Neisseria meningitidis	W	W	W	22	1	1	1	4	2	3	4	3	8	4	6	ST-11 complex	11	4	4	2
30136	M13 240635	UK [England]	2013	invasive (unspecified/other)	Neisseria meningitidis	W	W	W	22	1	1	1	4	2	3	4	3	8	4	6	ST-11 complex	11	4	4	2
35474	M13 240636	UK [England]	2013	invasive (unspecified/other)	Neisseria meningitidis	B	B	B	4	2	6	84	2	3	6	9	5	9	6	9	ST-41/44 complex	41	2	3	1
30137	M13 240637	UK [England]	2013	invasive (unspecified/other)	Neisseria meningitidis	W	W	W	22	1	1	1	4	2	3	4	3	8	4	6	ST-11 complex	11	4	4	2
35475	M13 240638	UK [England]	2013	invasive (unspecified/other)	Neisseria meningitidis	B	B	B	19	1	1	83	4	3	2	2	5	38	11	9		11329	4	4	2
38123	M13 240639	UK [England]	2013	invasive (unspecified/other)	Neisseria meningitidis	B	B	B	45	8	77	87	20	7	5	1	13	36	53	15	ST-213 complex	213	20	4	3
35477	M13 240640	UK [England]	2013	invasive (unspecified/other)	Neisseria meningitidis	B	B	B	13	3	57	145	3	4	10	34	5	608	11	9	ST-269 complex	9180	3	3	1
35478	M13 240641	UK [England]	2013	invasive (unspecified/other)	Neisseria meningitidis	B	B	B	4	NA	6	84	NA	3	6	9	5	9	6	9	ST-41/44 complex	41	NA	NA	1
38124	M13 240642	UK [England]	2013	invasive (unspecified/other)	Neisseria meningitidis	B	B	B	45	8	1	87	20	7	5	1	13	36	53	9	ST-213 complex	3120	20	4	3
35480	M13 240644	UK [England]	2013	invasive (unspecified/other)	Neisseria meningitidis	B	B	B	47	1	57	90	15	12	5	12	35	60	22	17	ST-461 complex	461	15	4	3
35481	M13 240646	UK [England]	2013	invasive (unspecified/other)	Neisseria meningitidis	B	B	B	19	1	1	83	4	4	10	2	NA	3	11	9		NA	4	4	2
35482	M13 240647	UK [England]	2013	invasive (unspecified/other)	Neisseria meningitidis	B	B	B	4	2	79	84	2	3	6	9	5	9	6	9	ST-41/44 complex	41	2	3	1
35483	M13 240648	UK [England]	2013	invasive (unspecified/other)	Neisseria meningitidis	B	B	B	4	2	1	84	2	3	6	9	5	8	6	9	ST-41/44 complex	485	2	3	1
30138	M13 240649	UK [England]	2013	invasive (unspecified/other)	Neisseria meningitidis	W	W	W	22	1	1	1	4	2	3	4	3	8	4	6	ST-11 complex	11	4	4	2
35484	M13 240652	UK [England]	2013	invasive (unspecified/other)	Neisseria meningitidis	Y	Y	Y	25	1	1	26	4	12	5	18	9	11	9	17	ST-23 complex	1655	4	4	2
30139	M13 240655	UK [England]	2013	invasive (unspecified/other)	Neisseria meningitidis	W	W	W	22	1	1	1	4	2	3	4	3	8	4	6	ST-11 complex	11	4	4	2
30140	M13 240657	UK [England]	2013	invasive (unspecified/other)	Neisseria meningitidis	W	W	W	22	1	1	1	4	2	3	4	3	8	4	6	ST-11 complex	11	4	4	2
35485	M13 240658	UK [England]	2013	invasive (unspecified/other)	Neisseria meningitidis	B	B	B	13	3	57	145	3	4	10	34	5	38	11	9	ST-269 complex	1161	3	3	1
35486	M13 240662	UK [England]	2013	invasive (unspecified/other)	Neisseria meningitidis	B	B	B	14	55	57	142	179	6	5	2	4	12	11	8		9174	NA	NA	1
35487	M13 240663	UK [England]	2013	invasive (unspecified/other)	Neisseria meningitidis	Y	Y	Y	25	1	1	26	4	12	5	18	9	11	9	17	ST-23 complex	1655	4	4	2
30141	M13 240664	UK [England]	2013	invasive (unspecified/other)	Neisseria meningitidis	W	W	W	22	1	1	1	4	2	3	4	3	8	4	6	ST-11 complex	11	4	4	2
35488	M13 240665	UK [England]	2013	invasive (unspecified/other)	Neisseria meningitidis	B	B	B	499	24	77	460	115	7	5	1	13	36	53	15	ST-213 complex	213	NA	NA	3
30143	M13 240667	UK [England]	2013	invasive (unspecified/other)	Neisseria meningitidis	W	W	W	22	1	1	1	4	2	3	4	3	8	4	6	ST-11 complex	11	4	4	2
30144	M13 240668	UK [England]	2013	invasive (unspecified/other)	Neisseria meningitidis	W	W	W	22	1	1	1	4	2	3	4	3	8	4	6	ST-11 complex	11	4	4	2
35489	M13 240670	UK [England]	2013	invasive (unspecified/other)	Neisseria meningitidis	B	B	B	4	2	1	84	2	3	6	9	5	8	6	9	ST-41/44 complex	485	2	3	1
38125	M13 240671	UK [Wales]	2013	invasive (unspecified/other)	Neisseria meningitidis	B	B	B	47	1	57	90	15	12	5	12	35	192	22	17	ST-461 complex	1946	15	4	3
30145	M13 240672	UK [England]	2013	invasive (unspecified/other)	Neisseria meningitidis	W	W	W	22	1	1	1	4	2	3	4	3	8	4	6	ST-11 complex	11	4	4	2
35491	M13 240673	UK [England]	2013	invasive (unspecified/other)	Neisseria meningitidis	Y	Y	Y	147	100	1	461	NA	10	5	18	9	11	9	17	ST-23 complex	23	NA	NA	2
35830	M13 240674	UK [England]	2013	invasive (unspecified/other)	Neisseria meningitidis	Y	Y	Y	25	1	1	26	4	12	5	18	9	11	9	17	ST-23 complex	1655	4	4	2
35492	M13 240675	UK [England]	2013	invasive (unspecified/other)	Neisseria meningitidis	B	B	B	14	1	1	142	13	3	6	108	5	9	6	9	ST-41/44 complex	1475	13	3	1
35493	M13 240677	UK [England]	2013	invasive (unspecified/other)	Neisseria meningitidis	B	B	B	19	1	2	65	4	6	6	63	9	9	11	5		11245	4	4	2
30146	M13 240678	UK [England]	2013	invasive (unspecified/other)	Neisseria meningitidis	W	W	W	22	1	1	1	4	2	3	4	3	8	4	6	ST-11 complex	11	4	4	2
30147	M13 240680	UK [England]	2013	invasive (unspecified/other)	Neisseria meningitidis	W	W	W	22	1	1	1	4	2	3	4	3	8	4	6	ST-11 complex	11	4	4	2
30148	M13 240681	UK [England]	2013	invasive (unspecified/other)	Neisseria meningitidis	W	W	W	22	1	1	1	4	2	3	4	3	8	4	6	ST-11 complex	11	4	4	2
35494	M13 240684	UK [England]	2013	invasive (unspecified/other)	Neisseria meningitidis	B	B	B	15	4	2	193	1	4	10	15	17	3	11	2	ST-269 complex	8004	1	1	1
35495	M13 240686	UK [England]	2013	invasive (unspecified/other)	Neisseria meningitidis	B	B	B	827	101	1	462	180	4	10	9	9	8	11	16	ST-269 complex	11246	NA	NA	1
35496	M13 240690	UK [England]	2013	invasive (unspecified/other)	Neisseria meningitidis	B	B	B	4	2	1	84	2	3	6	NA	5	9	6	9		NA	2	3	1
30210	M13 240691	UK [England]	2013	invasive (unspecified/other)	Neisseria meningitidis	C	C	C	13	5	1	251	11	2	3	4	3	8	4	6	ST-11 complex	11	11	2	1
35497	M13 240692	UK [England]	2013	invasive (unspecified/other)	Neisseria meningitidis	B	B	B	4	102	6	84	133	3	6	9	5	9	6	9	ST-41/44 complex	41	NA	NA	1
35498	M13 240693	UK [England]	2013	invasive (unspecified/other)	Neisseria meningitidis	B	B	B	510	28	8	599	30	4	10	5	4	6	3	8	ST-32 complex	32	NA	NA	1
35499	M13 240694	UK [England]	2013	invasive (unspecified/other)	Neisseria meningitidis	Y	Y	Y	25	1	1	26	4	12	5	NA	9	11	25	17		NA	4	4	2
35500	M13 240695	UK [England]	2013	invasive (unspecified/other)	Neisseria meningitidis	B	B	B	785	5	1	311	11	2	3	4	3	8	4	6	ST-11 complex	11	11	2	1
35501	M13 240696	UK [England]	2013	invasive (unspecified/other)	Neisseria meningitidis	Y	Y	Y	23	1	1	4	4	2	7	6	17	1	18	8	ST-167 complex	1627	4	4	2
35502	M13 240697	UK [England]	2013	invasive (unspecified/other)	Neisseria meningitidis	B	B	B	453	5	1	182	11	2	3	4	NA	NA	NA	NA		NA	11	2	1
35503	M13 240698	UK [England]	2013	invasive (unspecified/other)	Neisseria meningitidis	B	B	B	4	2	1	84	2	3	6	9	5	9	6	9	ST-41/44 complex	41	2	3	1
35504	M13 240699	UK [England]	2013	invasive (unspecified/other)	Neisseria meningitidis	B	B	B	278	103	5	910	143	3	6	9	5	9	6	9	ST-41/44 complex	41	NA	NA	1
35505	M13 240702	UK [England]	2013	invasive (unspecified/other)	Neisseria meningitidis	B	B	B	14	1	4	142	13	4	10	11	18	6	10	12	ST-35 complex	35	13	3	1
35506	M13 240703	UK [England]	2013	invasive (unspecified/other)	Neisseria meningitidis	B	B	B	15	4	2	193	1	4	10	15	9	8	11	9	ST-269 complex	269	1	1	1
30149	M13 240704	UK [England]	2013	invasive (unspecified/other)	Neisseria meningitidis	W	W	W	16	1	93	60	4	11	5	5	8	11	24	21	ST-22 complex	1617	4	4	2
35507	M13 240705	UK [England]	2013	invasive (unspecified/other)	Neisseria meningitidis	W	W	W	22	1	1	1	4	2	3	4	3	8	4	6	ST-11 complex	11	4	4	2
35508	M13 240706	UK [England]	2013	invasive (unspecified/other)	Neisseria meningitidis	Y	Y	Y	25	1	1	26	4	10	5	18	9	11	9	17	ST-23 complex	23	4	4	2
35509	M13 240708	UK [England]	2013	invasive (unspecified/other)	Neisseria meningitidis	B	B	B	14	1	1	142	13	3	6	9	5	9	6	9	ST-41/44 complex	41	13	3	1
35510	M13 240709	UK [England]	2013	invasive (unspecified/other)	Neisseria meningitidis	B	B	B	765	9	5	144	21	27	6	9	3	9	6	16	ST-41/44 complex	136	NA	NA	1
35511	M13 240710	UK [England]	2013	invasive (unspecified/other)	Neisseria meningitidis	B	B	B	13	3	1	463	3	17	5	19	17	3	26	2	ST-60 complex	60	3	3	1
35512	M13 240715	UK [England]	2013	invasive (unspecified/other)	Neisseria meningitidis	B	B	B	13	3	1	145	3	7	5	1	13	82	53	15	ST-213 complex	575	3	3	1
35513	M13 240716	UK [England]	2013	invasive (unspecified/other)	Neisseria meningitidis	Y	Y	Y	25	1	1	26	4	10	5	18	9	11	9	17	ST-23 complex	23	4	4	2
35514	M13 240717	UK [England]	2013	invasive (unspecified/other)	Neisseria meningitidis	B	B	B	45	8	77	87	20	7	5	1	13	36	53	15	ST-213 complex	213	20	4	3
35515	M13 240718	UK [England]	2013	invasive (unspecified/other)	Neisseria meningitidis	B	B	B	15	4	2	193	1	4	10	15	9	8	11	9	ST-269 complex	269	1	1	1
35516	M13 240719	UK [England]	2013	invasive (unspecified/other)	Neisseria meningitidis	B	B	B	4	2	1	84	2	7	5	1	13	36	53	15	ST-213 complex	213	2	3	1
35517	M13 240720	UK [England]	2013	invasive (unspecified/other)	Neisseria meningitidis	Y	Y	Y	16	1	1	60	4	11	5	18	17	11	24	21	ST-22 complex	114	4	4	2
35518	M13 240721	UK [England]	2013	invasive (unspecified/other)	Neisseria meningitidis	Y	Y	Y	25	1	1	26	4	12	5	18	9	11	9	17	ST-23 complex	1655	4	4	2
35519	M13 240723	UK [England]	2013	invasive (unspecified/other)	Neisseria meningitidis	B	B	B	31	9	1	58	16	4	10	769	26	6	3	8	ST-32 complex	11250	16	2	3
30150	M13 240724	UK [England]	2013	invasive (unspecified/other)	Neisseria meningitidis	W	W	W	22	1	1	1	4	2	3	4	3	8	4	6	ST-11 complex	11	4	4	2
35520	M13 240725	UK [England]	2013	invasive (unspecified/other)	Neisseria meningitidis	Y	Y	Y	25	1	1	26	4	12	5	18	9	11	9	17	ST-23 complex	1655	4	4	2
30151	M13 240726	UK [England]	2013	invasive (unspecified/other)	Neisseria meningitidis	W	W	W	22	1	1	1	4	2	3	4	3	8	4	6	ST-11 complex	11	4	4	2
35521	M13 240729	UK [England]	2013	invasive (unspecified/other)	Neisseria meningitidis	B	B	B	45	11	1	87	10	7	5	1	13	36	53	15	ST-213 complex	213	NA	NA	3
30152	M13 240730	UK [England]	2013	invasive (unspecified/other)	Neisseria meningitidis	W	W	W	22	1	1	1	4	2	3	4	3	8	4	6	ST-11 complex	11	4	4	2
35522	M13 240731	UK [England]	2013	invasive (unspecified/other)	Neisseria meningitidis	B	B	B	31	9	5	58	16	7	5	1	13	36	53	15	ST-213 complex	213	16	2	3
30153	M13 240732	UK [England]	2013	invasive (unspecified/other)	Neisseria meningitidis	W	W	W	22	1	1	1	4	2	3	4	3	8	4	6	ST-11 complex	11	4	4	2
35523	M13 240733	UK [England]	2013	invasive (unspecified/other)	Neisseria meningitidis	Y	Y	Y	16	1	1	60	4	2	16	6	17	9	733	8	ST-167 complex	11254	4	4	2
35524	M13 240734	UK [England]	2013	invasive (unspecified/other)	Neisseria meningitidis	C	C	C	13	3	1	145	3	17	5	19	176	3	26	2	ST-60 complex	9923	3	3	1
30211	M13 240735	UK [England]	2013	invasive (unspecified/other)	Neisseria meningitidis	C	Y	discrepancy	25	1	1	26	4	10	5	18	9	11	9	17	ST-23 complex	23	4	4	2
35525	M13 240737	UK [England]	2013	invasive (unspecified/other)	Neisseria meningitidis	B	B	B	13	3	57	145	3	4	10	34	5	38	11	9	ST-269 complex	1161	3	3	1
35526	M13 240738	UK [England]	2013	invasive (unspecified/other)	Neisseria meningitidis	B	B	B	4	2	1	84	2	3	6	9	5	8	6	9	ST-41/44 complex	485	2	3	1
35527	M13 240739	UK [England]	2013	invasive (unspecified/other)	Neisseria meningitidis	B	B	B	45	11	77	87	10	7	5	1	13	3	53	15	ST-213 complex	2101	NA	NA	3
35528	M13 240740	UK [England]	2013	invasive (unspecified/other)	Neisseria meningitidis	B	B	B	25	1	1	26	4	8	4	34	17	194	18	2	ST-103 complex	8023	4	4	2
35529	M13 240742	UK [England]	2013	invasive (unspecified/other)	Neisseria meningitidis	B	B	B	13	3	57	145	3	4	5	34	5	38	11	743		11330	3	3	1
35530	M13 240743	UK [England]	2013	invasive (unspecified/other)	Neisseria meningitidis	B	B	B	134	1	1	464	4	8	5	9	17	9	6	21		11259	4	4	2
35531	M13 240744	UK [England]	2013	invasive (unspecified/other)	Neisseria meningitidis	Y	Y	Y	25	1	1	26	4	12	5	NA	9	11	9	17		NA	4	4	2
35532	M13 240745	UK [England]	2013	invasive (unspecified/other)	Neisseria meningitidis	B	B	B	4	2	1	84	2	3	6	9	5	8	6	9	ST-41/44 complex	485	2	3	1
35533	M13 240746	UK [England]	2013	invasive (unspecified/other)	Neisseria meningitidis	B	B	B	4	2	10	84	2	3	6	9	25	9	6	9	ST-41/44 complex	2799	2	3	1
35534	M13 240748	UK [England]	2013	invasive (unspecified/other)	Neisseria meningitidis	B	B	B	19	104	1	83	181	4	10	2	5	607	11	9	ST-269 complex	11261	NA	NA	2
35535	M13 240749	UK [England]	2013	invasive (unspecified/other)	Neisseria meningitidis	Y	Y	Y	25	1	1	26	4	12	5	18	9	11	9	17	ST-23 complex	1655	4	4	2
35536	M13 240750	UK [England]	2013	invasive (unspecified/other)	Neisseria meningitidis	Y	Y	Y	25	1	1	26	4	12	5	18	9	11	9	17	ST-23 complex	1655	4	4	2
35537	M13 240751	UK [England]	2013	invasive (unspecified/other)	Neisseria meningitidis	Y	Y	Y	25	1	1	26	4	12	5	18	9	11	9	17	ST-23 complex	1655	4	4	2
35538	M13 240752	UK [England]	2013	invasive (unspecified/other)	Neisseria meningitidis	Y	Y	Y	25	1	1	26	4	10	5	18	9	11	9	17	ST-23 complex	23	4	4	2

35539	M13 240753	UK [England]	2013	invasive (unspecified/other)	Neisseria meningitidis	B	B	B	4	2	1	84	2	3	6	34	5	11	6	9	ST-41/44 complex	1194	2	3	1
35540	M13 240755	UK [England]	2013	invasive (unspecified/other)	Neisseria meningitidis	B	B	B	13	3	77	209	3	16	5	1	13	36	53	2	ST-213 complex	11262	3	3	1
30154	M14 240001	UK [England]	2014	invasive (unspecified/other)	Neisseria meningitidis	W	W	W	22	1	1	1	4	2	3	4	3	8	4	6	ST-11 complex	11	4	4	2
30155	M14 240002	UK [England]	2014	invasive (unspecified/other)	Neisseria meningitidis	W	W	W	22	1	1	1	4	2	3	4	3	8	4	6	ST-11 complex	11	4	4	2
35541	M14 240003	UK [England]	2014	invasive (unspecified/other)	Neisseria meningitidis	B	B	B	260	1	1	453	4	326	6	770	9	9	6	2	ST-41/44 complex	11263	4	3	1
30212	M14 240004	UK [England]	2014	invasive (unspecified/other)	Neisseria meningitidis	C	C	C	13	5	1	251	11	2	3	4	NA	8	4	6		NA	11	2	1
30156	M14 240007	UK [England]	2014	invasive (unspecified/other)	Neisseria meningitidis	W	W	W	22	1	1	1	4	2	3	4	3	8	4	6	ST-11 complex	11	4	4	2
35542	M14 240008	UK [England]	2014	invasive (unspecified/other)	Neisseria meningitidis	B	B	B	14	5	1	204	11	2	6	9	45	9	6	156	ST-41/44 complex	11265	11	2	1
35543	M14 240010	UK [England]	2014	invasive (unspecified/other)	Neisseria meningitidis	Y	Y	Y	25	1	1	26	4	10	5	18	9	11	9	17	ST-23 complex	23	4	4	2
35544	M14 240011	UK [England]	2014	invasive (unspecified/other)	Neisseria meningitidis	B	B	B	13	3	57	145	3	4	10	34	5	38	11	9	ST-269 complex	1161	3	3	1
35545	M14 240012	UK [England]	2014	invasive (unspecified/other)	Neisseria meningitidis	Y	Y	Y	25	1	1	26	4	12	5	18	9	11	9	17	ST-23 complex	1655	4	4	2
30157	M14 240013	UK [England]	2014	invasive (unspecified/other)	Neisseria meningitidis	W	W	W	22	1	1	1	4	2	3	4	3	8	4	6	ST-11 complex	11	4	4	2
35546	M14 240014	UK [England]	2014	invasive (unspecified/other)	Neisseria meningitidis	B	B	B	1	6	7	100	7	4	10	5	4	6	3	8	ST-32 complex	32	7	1	1
35547	M14 240015	UK [England]	2014	invasive (unspecified/other)	Neisseria meningitidis	Y	Y	Y	24	1	1	27	4	2	7	6	17	9	6	8	ST-167 complex	1624	4	4	2
35548	M14 240016	UK [England]	2014	invasive (unspecified/other)	Neisseria meningitidis	B	B	B	14	1	1	142	13	3	6	19	5	3	6	9	ST-41/44 complex	340	13	3	1
35549	M14 240017	UK [England]	2014	invasive (unspecified/other)	Neisseria meningitidis	B	B	B	4	2	1	84	2	3	6	49	25	9	6	9	ST-41/44 complex	3446	2	3	1
35550	M14 240018	UK [England]	2014	invasive (unspecified/other)	Neisseria meningitidis	B	B	B	14	7	1	142	6	3	7	9	5	17	22	9	ST-41/44 complex	6782	6	2	1
30158	M14 240019	UK [England]	2014	invasive (unspecified/other)	Neisseria meningitidis	W	W	W	762	1	1	296	4	2	3	4	3	8	4	6	ST-11 complex	11	4	3	1
35551	M14 240020	UK [England]	2014	invasive (unspecified/other)	Neisseria meningitidis	C	C	C	19	1	2	65	4	9	4	9	17	5	6	2	ST-103 complex	5133	4	4	2
35552	M14 240021	UK [Wales]	2014	invasive (unspecified/other)	Neisseria meningitidis	B	B	B	4	2	79	84	2	3	16	325	5	9	6	9	ST-41/44 complex	5981	2	3	1
30159	M14 240022	UK [England]	2014	invasive (unspecified/other)	Neisseria meningitidis	W	W	W	22	1	1	1	4	2	3	4	3	573	4	6	ST-11 complex	8621	4	4	2
35553	M14 240023	UK [England]	2014	invasive (unspecified/other)	Neisseria meningitidis	Y	Y	Y	25	1	1	26	4	12	5	NA	9	11	9	17		NA	4	4	2
35554	M14 240024	UK [England]	2014	invasive (unspecified/other)	Neisseria meningitidis	B	B	B	144	1	4	232	4	8	10	5	4	5	3	8	ST-32 complex	34	4	3	1
35555	M14 240025	UK [England]	2014	invasive (unspecified/other)	Neisseria meningitidis	B	B	B	13	3	57	145	3	4	10	34	5	38	11	9	ST-269 complex	1161	3	3	1
30160	M14 240026	UK [England]	2014	invasive (unspecified/other)	Neisseria meningitidis	W	W	W	22	1	1	1	4	2	3	4	3	8	4	6	ST-11 complex	11	4	4	2
35556	M14 240027	UK [England]	2014	invasive (unspecified/other)	Neisseria meningitidis	B	B	B	14	7	1	142	6	10	6	63	5	9	6	12	ST-41/44 complex	1403	6	2	1
35557	M14 240028	UK [England]	2014	invasive (unspecified/other)	Neisseria meningitidis	B	B	B	224	15	1	252	68	4	10	5	4	5	3	8	ST-32 complex	2506	NA	NA	1
30161	M14 240029	UK [England]	2014	invasive (unspecified/other)	Neisseria meningitidis	W	W	W	22	1	1	1	4	2	3	4	3	8	4	6	ST-11 complex	11	4	4	2
35558	M14 240030	UK [England]	2014	invasive (unspecified/other)	Neisseria meningitidis	B	B	B	13	3	1	145	3	7	5	1	13	82	53	15	ST-213 complex	575	3	3	1
30162	M14 240031	UK [England]	2014	invasive (unspecified/other)	Neisseria meningitidis	W	W	W	22	1	1	1	4	2	3	4	3	8	4	6	ST-11 complex	11	4	4	2
35559	M14 240037	UK [England]	2014	invasive (unspecified/other)	Neisseria meningitidis	B	B	B	24	1	4	25	4	4	10	2	17	38	11	9	ST-269 complex	9242	4	4	2
35560	M14 240038	UK [England]	2014	invasive (unspecified/other)	Neisseria meningitidis	W	W	W	16	1	1	60	4	11	5	18	8	11	38	21	ST-22 complex	2638	4	4	2
35561	M14 240039	UK [England]	2014	invasive (unspecified/other)	Neisseria meningitidis	B	B	B	4	2	1	84	2	3	6	NA	5	9	6	9		NA	2	3	1
30163	M14 240042	UK [England]	2014	invasive (unspecified/other)	Neisseria meningitidis	W	W	W	22	1	1	1	4	2	3	4	3	8	4	6	ST-11 complex	11	4	4	2
30164	M14 240043	UK [England]	2014	invasive (unspecified/other)	Neisseria meningitidis	W	W	W	22	18	1	1	87	2	3	4	3	8	4	6	ST-11 complex	11	NA	NA	2
35562	M14 240045	UK [England]	2014	invasive (unspecified/other)	Neisseria meningitidis	B	B	B	4	2	1	84	2	3	6	9	5	9	6	9	ST-41/44 complex	41	2	3	1
35563	M14 240046	UK [Wales]	2014	invasive (unspecified/other)	Neisseria meningitidis	B	B	B	4	2	1	84	2	3	6	9	5	9	6	9	ST-41/44 complex	41	2	3	1
35564	M14 240048	UK [England]	2014	invasive (unspecified/other)	Neisseria meningitidis	B	B	B	1	6	3	100	7	8	10	5	4	5	3	8	ST-32 complex	34	7	1	1
35565	M14 240049	UK [England]	2014	invasive (unspecified/other)	Neisseria meningitidis	Y	Y	Y	25	1	1	26	4	12	5	18	9	11	9	17	ST-23 complex	1655	4	4	2
30165	M14 240052	UK [England]	2014	invasive (unspecified/other)	Neisseria meningitidis	W	W	W	22	1	1	1	4	2	3	4	3	8	4	6	ST-11 complex	11	4	4	2
30166	M14 240053	UK [England]	2014	invasive (unspecified/other)	Neisseria meningitidis	W	W	W	22	1	1	1	4	662	3	4	3	8	4	6	ST-11 complex	10651	4	4	2
30167	M14 240054	UK [England]	2014	invasive (unspecified/other)	Neisseria meningitidis	W	W	W	22	1	1	1	4	2	3	4	3	8	4	6	ST-11 complex	11	4	4	2
35566	M14 240055	UK [England]	2014	invasive (unspecified/other)	Neisseria meningitidis	B	B	B	4	2	1	84	2	3	6	9	5	11	6	9	ST-41/44 complex	154	2	3	1
35567	M14 240056	UK [England]	2014	invasive (unspecified/other)	Neisseria meningitidis	Y	Y	Y	25	1	1	26	4	12	5	18	9	11	9	17	ST-23 complex	1655	4	4	2
35568	M14 240059	UK [England]	2014	invasive (unspecified/other)	Neisseria meningitidis	B	B	B	13	3	57	145	3	4	10	34	5	608	11	9	ST-269 complex	9180	3	3	1
35569	M14 240060	UK [England]	2014	invasive (unspecified/other)	Neisseria meningitidis	Y	Y	Y	25	1	1	26	4	12	5	18	9	11	9	17	ST-23 complex	1655	4	4	2
35570	M14 240061	UK [Wales]	2014	invasive (unspecified/other)	Neisseria meningitidis	B	B	B	15	4	2	193	1	4	10	15	9	8	11	17	ST-269 complex	283	1	1	1
35571	M14 240062	UK [England]	2014	invasive (unspecified/other)	Neisseria meningitidis	B	B	B	1	6	3	100	7	8	10	5	4	5	3	8	ST-32 complex	34	7	1	1
30168	M14 240072	UK [England]	2014	invasive (unspecified/other)	Neisseria meningitidis	W	W	W	22	18	1	1	87	2	3	4	3	8	4	6	ST-11 complex	11	NA	NA	2
35572	M14 240073	UK [England]	2014	invasive (unspecified/other)	Neisseria meningitidis	B	B	B	15	4	2	193	1	4	10	2	5	38	11	9	ST-269 complex	275	1	1	1
35573	M14 240074	UK [England]	2014	invasive (unspecified/other)	Neisseria meningitidis	B	B	B	651	4	98	465	1	3	490	19	5	3	6	9	ST-41/44 complex	11266	1	1	3
35574	M14 240075	UK [England]	2014	invasive (unspecified/other)	Neisseria meningitidis	Y	Y	Y	25	1	1	26	4	12	5	18	9	11	9	17	ST-23 complex	1655	4	4	2
35575	M14 240077	UK [England]	2014	invasive (unspecified/other)	Neisseria meningitidis	B	B	B	1	6	3	100	7	8	10	5	4	5	3	8	ST-32 complex	34	7	1	1
35576	M14 240078	UK [England]	2014	invasive (unspecified/other)	Neisseria meningitidis	B	B	B	45	11	77	87	10	7	5	1	13	36	53	15	ST-213 complex	213	NA	NA	3
30169	M14 240082	UK [Northern Ireland]	2014	invasive (unspecified/other)	Neisseria meningitidis	W	W	W	22	1	1	1	4	2	3	4	3	8	4	6	ST-11 complex	11	4	4	2
35577	M14 240083	UK [Northern Ireland]	2014	invasive (unspecified/other)	Neisseria meningitidis	B	B	B	316	49	57	155	83	12	5	12	35	192	22	788	ST-461 complex	11865	NA	NA	3
35578	M14 240085	UK [Wales]	2014	invasive (unspecified/other)	Neisseria meningitidis	B	B	B	4	2	1	84	2	4	6	9	5	9	6	9	ST-41/44 complex	5098	2	3	1
35579	M14 240087	UK [England]	2014	invasive (unspecified/other)	Neisseria meningitidis	B	B	B	45	8	1	87	20	7	5	1	13	36	53	2	ST-213 complex	3116	20	4	3
35580	M14 240088	UK [England]	2014	invasive (unspecified/other)	Neisseria meningitidis	B	B	B	47	1	57	90	15	12	5	12	35	60	22	17	ST-461 complex	461	15	4	3
35581	M14 240090	UK [England]	2014	invasive (unspecified/other)	Neisseria meningitidis	W	W	W	22	1	1	1	4	2	3	4	3	8	4	6	ST-11 complex	11	4	4	2
35582	M14 240091	UK [England]	2014	invasive (unspecified/other)	Neisseria meningitidis	Y	Y	Y	25	1	1	26	4	12	5	18	9	11	9	17	ST-23 complex	1655	4	4	2
35583	M14 240093	UK [England]	2014	invasive (unspecified/other)	Neisseria meningitidis	B	B	B	37	1	1	408	4	7	8	4	19	10	734	2	ST-18 complex	11272	4	3	1
30213	M14 240094	UK [England]	2014	invasive (unspecified/other)	Neisseria meningitidis	C	C	C	13	5	1	251	11	2	3	4	NA	8	4	6		NA	11	2	1
35584	M14 240095	UK [Northern Ireland]	2014	invasive (unspecified/other)	Neisseria meningitidis	B	B	B	4	2	1	84	2	3	5	34	5	11	6	9	ST-41/44 complex	5986	2	3	1
35585	M14 240096	UK [England]	2014	invasive (unspecified/other)	Neisseria meningitidis	B	B	B	14	7	1	142	6	17	6	9	5	9	6	9	ST-41/44 complex	3447	6	2	1
35586	M14 240098	UK [England]	2014	invasive (unspecified/other)	Neisseria meningitidis	B	B	B	4	2	6	84	2	3	6	9	5	9	6	9	ST-41/44 complex	41	2	3	1
35587	M14 240099	UK [England]	2014	invasive (unspecified/other)	Neisseria meningitidis	B	B	B	19	1	1	83	4	4	10	1	5	3	11	9	ST-269 complex	6789	4	4	2
30214	M14 240100	UK [England]	2014	invasive (unspecified/other)	Neisseria meningitidis	C	C	C	22	1	1	1	4	2	3	4	3	8	4	6	ST-11 complex	11	4	4	2
35588	M14 240101	UK [England]	2014	invasive (unspecified/other)	Neisseria meningitidis	B	B	B	1	6	3	100	7	8	10	77	4	6	3	8	ST-32 complex	749	7	1	1
35589	M14 240103	UK [England]	2014	invasive (unspecified/other)	Neisseria meningitidis	B	B	B	94	105	1	466	136	4	10	5	4	6	3	8	ST-32 complex	32	NA	NA	3
35590	M14 240104	UK [England]	2014	invasive (unspecified/other)	Neisseria meningitidis	Y		Y	25	1	1	26	4	10	5	18	9	11	9	17	ST-23 complex	23	4	4	2
35591	M14 240105	UK [England]	2014	invasive (unspecified/other)	Neisseria meningitidis	Y		Y	25	1	1	26	4	10	5	18	9	11	9	17	ST-23 complex	23	4	4	2
30215	M14 240107	UK [England]	2014	invasive (unspecified/other)	Neisseria meningitidis	C	C	C	22	1	1	1	4	2	3	4	3	8	4	6	ST-11 complex	11	4	4	2
35592	M14 240108	UK [Wales]	2014	invasive (unspecified/other)	Neisseria meningitidis	Y	Y	Y	25	1	1	26	4	12	5	18	9	11	9	17	ST-23 complex	1655	4	4	2
35593	M14 240109	UK [England]	2014	invasive (unspecified/other)	Neisseria meningitidis	C	C	C	15	4	2	193	1	4	10	15	9	8	11	13	ST-269 complex	467	1	1	1
35594	M14 240110	UK [England]	2014	invasive (unspecified/other)	Neisseria meningitidis	B	B	B	14	1	1	142	13	3	6	108	5	9	6	9	ST-41/44 complex	1475	13	3	1
35595	M14 240111	UK [England]	2014	invasive (unspecified/other)	Neisseria meningitidis	Y	Y	Y	25	1	1	26	4	12	5	18	9	11	9	17	ST-23 complex	1655	4	4	2
35596	M14 240112	UK [England]	2014	invasive (unspecified/other)	Neisseria meningitidis	Y	Y	Y	25	1	1	26	4	12	5	18	9	11	9	17	ST-23 complex	1655	4	4	2
35597	M14 240114	UK [England]	2014	invasive (unspecified/other)	Neisseria meningitidis	B	B	B	4	NA	10	84	NA	16	2	9	5	8	6	9	ST-41/44 complex	11460	NA	NA	1
35598	M14 240116	UK [England]	2014	invasive (unspecified/other)	Neisseria meningitidis	W	W	W	22	1	1	1	4	2	3	4	3	8	4	6	ST-11 complex	11	4	4	2
35599	M14 240117	UK [England]	2014	invasive (unspecified/other)	Neisseria meningitidis	Y	Y	Y	25	1	1	26	4	12	5	18	9	11	9	17	ST-23 complex	1655	4	4	2
35600	M14 240118	UK [England]	2014	invasive (unspecified/other)	Neisseria meningitidis	Y	Y	Y	25	1	1	26	4	10	5	18	9	11	9	17	ST-23 complex	23	4	4	2
35601	M14 240119	UK [England]	2014	invasive (unspecified/other)	Neisseria meningitidis	B	B	B	84	13	1	70	22	42	26	46	24	2	20	17	ST-282 complex	282	NA	NA	3
35602	M14 240120	UK [England]	2014	invasive (unspecified/other)	Neisseria meningitidis	Y	Y	Y	19	1	1	93	13	2	16	6	NA	9	18	8		NA	13	4	2
35603	M14 240121	UK [England]	2014	invasive (unspecified/other)	Neisseria meningitidis	B	B	B	102	8	1	2	20	7	5	1	13	36	53	15	ST-213 complex	213	20	4	2
35604	M14 240122	UK [England]	2014	invasive (unspecified/other)	Neisseria meningitidis	B	B	B	1	6	3	100	7	8	10	5	4	6	3	8	ST-32 complex	33	7	1	1
35605	M14 240123	UK [England]	2014	invasive (unspecified/other)	Neisseria meningitidis	B	B	B	13	3	1	145	3	17	5	19	17	3	26	22	ST-60 complex	2455	3	3	1
35606	M14 240124	UK [England]	2014	invasive (unspecified/other)	Neisseria meningitidis	B	B	B	4	2	1	84	2	11	6	9	5	11	6	9	ST-41/44 complex	5480	2	3	1
35607	M14 240125	UK [England]	2014	invasive (unspecified/other)	Neisseria meningitidis	W		W	16	1	1	60	4	11	5	18	17	11	24	21	ST-22 complex	114	4	4	2
35608	M14 240126	UK [England]	2014	invasive (unspecified/other)	Neisseria meningitidis	W	NG	discrepancy	22	1	1	1	4	2	3	4	3	8	4	6	ST-11 complex	11	4	4	2
35609	M14 240127	UK [Northern Ireland]	2014	invasive (unspecified/other)	Neisseria meningitidis	B	B	B	14	5	1	204	11	3	6	494	5	3	6	9	ST-41/44 complex	9819	11	2	1
35610	M14 240129	UK [England]	2014	invasive (unspecified/other)	Neisseria meningitidis	W	W	W	16	1	1	60	4	11	5	5	8	11	24	21	ST-22 complex	1617	4	4	2
35611	M14 240130	UK [England]	2014	invasive (unspecified/other)	Neisseria meningitidis	Y	Y	Y	25	1	1	26	4	12	5	NA	9	11	9	17		NA	4	4	2
35612	M14 240133	UK [Wales]	2014	invasive (unspecified/other)	Neisseria meningitidis	B	B	B	13	3	57	209	3	12	5	12	35	192	22	17	ST-461 complex	1946	3	3	1
35613	M14 240134	UK [England]	2014	invasive (unspecified/other)	Neisseria meningitidis	B	B	B	826	106	1	467	24	3	6	9	5	9	6	9	ST-41/44 complex	41	NA	NA	3
35614	M14 240135	UK [England]	2014	invasive (unspecified/other)	Neisseria meningitidis	W	W	W	22	1	1	1	4	2	3	4	3	8	4	6	ST-11 complex	11	4	4	2
35615	M14 240136	UK [England]	2014	invasive (unspecified/other)	Neisseria meningitidis	Y	Y	Y	25	1	1	26	4	12	5	18	9	11	9	17	ST-23 complex	1655	4	4	2
37662	M14 240138	UK [England]	2014	invasive (unspecified/other)	Neisseria meningitidis	B	B	B	31	9	5	58	16	7	5	1	13	36	53	15	ST-213 complex	213	16	2	3
35616	M14 240140	UK [England]	2014	invasive (unspecified/other)	Neisseria meningitidis	Y	Y	Y	25	1	1	26	4	10	5	18	9	11	9	17	ST-23 complex	23	4	4	2
35617	M14 240141	UK [England]	2014	invasive (unspecified/other)	Neisseria meningitidis	B	B	B	4	2	1	84	2	3	6	9	5	9	NA	9		NA	2	3	1
35618	M14 240142	UK [England]	2014	invasive (unspecified/other)	Neisseria meningitidis	W	W	W	256	15	5	207	58	2	3	4	3	8	4	6	ST-11 complex	11	NA	NA	1
35619	M14 240143	UK [England]	2014	invasive (unspecified/other)	Neisseria meningitidis	B	B	B	1	6	7	100	7	4	10	5	4	722	3	8	ST-32 complex	11278	7	1	1
35620	M14 240144	UK [England]	2014	invasive (unspecified/other)	Neisseria meningitidis	Y	Y	Y	25	1	1	26	4	12	5	18	9	11	9	17	ST-23 complex	1655	4	4	2
35621	M14 240145	UK [England]	2014	invasive (unspecified/other)	Neisseria meningitidis	W	W	W	22	1	1	1	4	2	3	4	3	8	4	6	ST-11 complex	11	4	4	2
35622	M14 240147	UK [England]	2014	invasive (unspecified/other)	Neisseria meningitidis	W	W	W	762	1	1	296	4	2	3	4	3	8	4	6	ST-11 complex	11	4	3	1
37663	M14 240148	UK [Wales]	2014	invasive (unspecified/other)	Neisseria meningitidis	B	B	B	4	2	79	84	2	3	6	9	5	9	6	9	ST-41/44 complex	41	2	3	1
35623	M14 240149	UK [Northern Ireland]	2014	invasive (unspecified/other)	Neisseria meningitidis	B	B	B	13	3	57	145	3	4	10	34	5	38	11	9	ST-269 complex	1161	3	3	1
35624	M14 240150	UK [England]	2014	invasive (unspecified/other)	Neisseria meningitidis	W	W	W	22	1	1	1	4	2	3	4	3	8	4	6	ST-11 complex	11	4	4	2
35625	M14 240153	UK [England]	2014	invasive (unspecified/other)	Neisseria meningitidis	B	B	B	4	2	1	84	2	3	6	9	5	9	6	2	ST-41/44 complex	2314	2	3	1
35626	M14 240154	UK [England]	2014	invasive (unspecified/other)	Neisseria meningitidis	B	B	B	86	4	2	222	1	3	6	19	7	3	6	5		8988	1	1	1
37664	M14 240156	UK [England]	2014	invasive (unspecified/other)	Neisseria meningitidis	Y	Y	Y	25	1	1	26	4	12	5	18	9	11	9	17	ST-23 complex	1655	4	4	2
35627	M14 240157	UK [England]	2014	invasive (unspecified/other)	Neisseria meningitidis	Y		Y	25	1	1	26	4	12	5	18	9	11	9	17	ST-23 complex	1655	4	4	2
37665	M14 240166	UK [England]	2014	invasive (unspecified/other)	Neisseria meningitidis	B	B	B	13	3	57	145	3	4	10	34	5	38	11	9	ST-269 complex	1161	3	3	1
35628	M14 240168	UK [Northern Ireland]	2014	invasive (unspecified/other)	Neisseria meningitidis	C	C	C	22	1	1	1	4	2	3	4	3	8	4	6	ST-11 complex	11	4	4	2
37666	M14 240170	UK [England]	2014	invasive (unspecified/other)	Neisseria meningitidis	Y	Y	Y	25	1	1	26	4	10	5	18	9	11	9	17	ST-23 complex	23	4	4	2
37667	M14 240172	UK [England]	2014	invasive (unspecified/other)	Neisseria meningitidis	Y	Y	Y	25	1	1	26	4	12	5	18	9	11	9	17	ST-23 complex	1655	4	4	2
35629	M14 240174	UK [Wales]	2014	invasive (unspecified/other)	Neisseria meningitidis	B	B	B	4	2	1	84	2	10	6	173	5	79	6	9	ST-41/44 complex	11279	2	3	1
35630	M14 240175	UK [England]	2014	invasive (unspecified/other)	Neisseria meningitidis	B	B	B	15	4	2	193	1	4	10	15	9	8	11	9	ST-269 complex	269	1	1	1
35631	M14 240177	UK [England]	2014	invasive (unspecified/other)	Neisseria meningitidis	B	B	B	13	3	57	145	3	4	10	34	5	38	11	9	ST-269 complex	1161	3	3	1
35632	M14 240178	UK [England]	2014	invasive (unspecified/other)	Neisseria meningitidis	W	W	W	22	1	1	1	4	2	3	4	3	8	4	6	ST-11 complex	11	4	4	2
35633	M14 240179	UK [England]	2014	invasive (unspecified/other)	Neisseria meningitidis	B	B	B	15	4	2	193	1	4	10	15	9	8	11	13	ST-269 complex	467	1	1	1
35634	M14 240180	UK [England]	2014	invasive (unspecified/other)	Neisseria meningitidis	W	W	W	22	1	1	1	4	2	3	4	3	8	4	6	ST-11 complex	11	4	4	2
35635	M14 240199	UK [England]	2014	invasive (unspecified/other)	Neisseria meningitidis	W	W	W	13	1	1	468	4	2	3	4	3	8	4	6	ST-11 complex	11	4	3	1
35636	M14 240201	UK [England]	2014	invasive (unspecified/other)	Neisseria meningitidis	B	B	B	143	4	2	99	1	4	10	15	9	14	11	17	ST-269 complex	1774	1	1	3
35637	M14 240202	UK [England]	2014	invasive (unspecified/other)	Neisseria meningitidis	Y	Y	Y	13	3	57	209	3	12	5	18	332	11	9	17	ST-23 complex	11280	3	3	1
35638	M14 240204	UK [England]	2014	invasive (unspecified/other)	Neisseria meningitidis	W	W	W	22	1	1	1	4	2	3	4	3	8	4	6	ST-11 complex	11	4	4	2
35639	M14 240205	UK [Northern Ireland]	2014	invasive (unspecified/other)	Neisseria meningitidis	B	B	B	828	107	26	469	182	7	5	159	13	36	53	15	ST-213 complex	4145	NA	NA	1
35640	M14 240206	UK [England]	2014	invasive (unspecified/other)	Neisseria meningitidis	B	B	B	13	3	57	145	3	4	10	34	5	38	11	9	ST-269 complex	1161	3	3	1
35641	M14 240208	UK [Wales]	2014	invasive (unspecified/other)	Neisseria meningitidis	NG	E	E	13	3	57	209	3	8	25	7	720	21	26	49	ST-1157 complex	11282	3	3	1
35642	M14 240210	UK [England]	2014	invasive (unspecified/other)	Neisseria meningitidis	Y	Y	Y	25	1	1	26	4	10	5	18	9	11	9	17	ST-23 complex	23	4	4	2
35643	M14 240211	UK [England]	2014	invasive (unspecified/other)	Neisseria meningitidis	W	W	W	22	1	1	1	4	2	3	4	3	8	4	6	ST-11 complex	11	4	4	2
35644	M14 240213	UK [England]	2014	invasive (unspecified/other)	Neisseria meningitidis	B	B	B	13	3	57	209	3	8	25	NA	17	21	26	49		NA	3	3	1
35645	M14 240215	UK [England]	2014	invasive (unspecified/other)	Neisseria meningitidis	B	B	B	45	47	77	87	78	7	5	1	13	36	53	15	ST-213 complex	213	NA	NA	3
35646	M14 240217	UK [England]	2014	invasive (unspecified/other)	Neisseria meningitidis	B	B	B	683	4	2	176	1	9	5	9	9	9	6	2	ST-41/44 complex	1423	1	1	2
35647	M14 240218	UK [England]	2014	invasive (unspecified/other)	Neisseria meningitidis	B	B	B	510	28	8	599	30	4	10	48	4	6	3	8	ST-32 complex	7460	NA	NA	1
35648	M14 240220	UK [Wales]	2014	invasive (unspecified/other)	Neisseria meningitidis	Y	Y	Y	25	1	1	26	4	12	5	NA	9	11	9	17		NA	4	4	2
35649	M14 240221	UK [England]	2014	invasive (unspecified/other)	Neisseria meningitidis	Y	Y	Y	25	1	1	26	4	10	5	NA	9	11	9	17		NA	4	4	2
35650	M14 240222	UK [England]	2014	invasive (unspecified/other)	Neisseria meningitidis	C	C	C	13	5	1	251	11	2	3	4	3	8	4	6	ST-11 complex	11	11	2	1

35651	M14 240223	UK [England]	2014	invasive (unspecified/other)	Neisseria meningitidis	W	W	W	22	1	1	1	4	2	3	4	3	8	4	6	ST-11 complex	11	4	4	2
35652	M14 240224	UK [England]	2014	invasive (unspecified/other)	Neisseria meningitidis	C	C	C	4	2	1	84	2	3	6	NA	5	8	6	9		NA	2	3	1
35653	M14 240225	UK [England]	2014	invasive (unspecified/other)	Neisseria meningitidis	Y		Y	25	1	1	26	4	12	5	18	9	11	9	17	ST-23 complex	1655	4	4	2
35654	M14 240226	UK [England]	2014	invasive (unspecified/other)	Neisseria meningitidis	Y	Y	Y	25	1	1	26	4	12	5	NA	9	11	9	17		NA	4	4	2
35655	M14 240227	UK [Wales]	2014	invasive (unspecified/other)	Neisseria meningitidis	B	B	B	1	6	3	100	7	8	10	5	4	6	3	15	ST-32 complex	8049	7	1	1
37668	M14 240229	UK [England]	2014	invasive (unspecified/other)	Neisseria meningitidis	C	C	C	22	1	1	1	4	2	3	4	3	8	4	6	ST-11 complex	11	4	4	2
37669	M14 240230	UK [England]	2014	invasive (unspecified/other)	Neisseria meningitidis	B	B	B	4	116	1	84	NA	3	6	9	5	9	6	9	ST-41/44 complex	41	NA	NA	1
35656	M14 240232	UK [England]	2014	invasive (unspecified/other)	Neisseria meningitidis	B	B	B	19	1	1	65	4	9	6	9	9	9	64	9	ST-41/44 complex	409	4	4	2
35657	M14 240233	UK [England]	2014	invasive (unspecified/other)	Neisseria meningitidis	B	B	B	186	9	1	470	16	9	6	9	9	9	64	9	ST-41/44 complex	409	16	2	3
35658	M14 240234	UK [England]	2014	invasive (unspecified/other)	Neisseria meningitidis	B	B	B	15	4	77	193	1	4	10	15	9	8	11	738	ST-269 complex	11291	1	1	1
35659	M14 240236	UK [England]	2014	invasive (unspecified/other)	Neisseria meningitidis	C	C	C	0	5	1	917	11	2	3	38	3	8	4	6	ST-11 complex	10482	11	2	NA
35660	M14 240237	UK [Wales]	2014	invasive (unspecified/other)	Neisseria meningitidis	Y	Y	Y	25	1	1	26	4	12	5	18	9	11	9	17	ST-23 complex	1655	4	4	2
35661	M14 240238	UK [England]	2014	invasive (unspecified/other)	Neisseria meningitidis	W	W	W	84	12	1	70	28	11	5	18	8	11	24	21	ST-22 complex	22	NA	NA	3
35662	M14 240241	UK [England]	2014	invasive (unspecified/other)	Neisseria meningitidis	B	B	B	19	1	1	83	4	4	10	2	5	38	11	9	ST-269 complex	275	4	4	2
35663	M14 240242	UK [England]	2014	invasive (unspecified/other)	Neisseria meningitidis	B	B	B	700	7	1	279	6	1	5	13	53	26	41	3	ST-162 complex	162	6	2	1
35664	M14 240245	UK [England]	2014	invasive (unspecified/other)	Neisseria meningitidis	W	W	W	22	1	1	1	4	2	3	4	3	8	735	6	ST-11 complex	11292	4	4	2
35665	M14 240246	UK [England]	2014	invasive (unspecified/other)	Neisseria meningitidis	Y	Y	Y	25	1	1	26	4	10	5	18	32	11	9	17	ST-23 complex	11293	4	4	2
35666	M14 240248	UK [England]	2014	invasive (unspecified/other)	Neisseria meningitidis	B	B	B	1	6	7	100	7	4	10	5	4	6	3	8	ST-32 complex	32	7	1	1
35667	M14 240249	UK [Wales]	2014	invasive (unspecified/other)	Neisseria meningitidis	B	B	B	823	5	1	471	11	2	3	4	3	8	4	6	ST-11 complex	11	11	2	1
35668	M14 240251	UK [England]	2014	invasive (unspecified/other)	Neisseria meningitidis	B	B	B	700	7	1	279	6	1	5	13	53	26	41	3	ST-162 complex	162	6	2	1
35669	M14 240252	UK [England]	2014	invasive (unspecified/other)	Neisseria meningitidis	B	B	B	13	3	57	145	3	4	10	34	5	38	11	9	ST-269 complex	1161	3	3	1
35670	M14 240253	UK [England]	2014	invasive (unspecified/other)	Neisseria meningitidis	W	W	W	22	1	1	1	4	2	3	4	3	8	4	6	ST-11 complex	11	4	4	2
35671	M14 240254	UK [England]	2014	invasive (unspecified/other)	Neisseria meningitidis	B	B	B	14	7	1	142	6	3	6	9	NA	26	6	9		NA	6	2	1
35672	M14 240255	UK [England]	2014	invasive (unspecified/other)	Neisseria meningitidis	C	C	C	22	1	1	1	4	2	3	4	3	8	4	6	ST-11 complex	11	4	4	2
35673	M14 240256	UK [England]	2014	invasive (unspecified/other)	Neisseria meningitidis	B	B	B	608	2	1	277	2	3	6	34	5	11	6	9	ST-41/44 complex	1194	2	3	1
35674	M14 240257	UK [England]	2014	invasive (unspecified/other)	Neisseria meningitidis	W	W	W	22	1	1	1	4	2	3	4	3	8	4	6	ST-11 complex	11	4	4	2
35675	M14 240258	UK [England]	2014	invasive (unspecified/other)	Neisseria meningitidis	W	W	W	22	1	1	1	4	2	3	4	3	8	4	6	ST-11 complex	11	4	4	2
35676	M14 240259	UK [Northern Ireland]	2014	invasive (unspecified/other)	Neisseria meningitidis	B	B	B	21	1	1	7	4	1	5	13	53	26	41	739	ST-162 complex	11295	4	4	2
35677	M14 240261	UK [England]	2014	invasive (unspecified/other)	Neisseria meningitidis	B	B	B	13	3	57	145	3	4	10	34	5	38	11	9	ST-269 complex	1161	3	3	1
35678	M14 240264	UK [England]	2014	invasive (unspecified/other)	Neisseria meningitidis	B	B	B	45	11	77	87	10	7	5	1	NA	36	53	15		NA	NA	NA	3
35679	M14 240265	UK [England]	2014	invasive (unspecified/other)	Neisseria meningitidis	B	B	B	15	4	2	193	1	4	10	15	142	8	11	9	ST-269 complex	1089	1	1	1
35680	M14 240266	UK [England]	2014	invasive (unspecified/other)	Neisseria meningitidis	B	B	B	19	1	1	83	4	4	10	2	5	38	11	9	ST-269 complex	275	4	4	2
35681	M14 240267	UK [England]	2014	invasive (unspecified/other)	Neisseria meningitidis	B	B	B	4	2	79	84	2	3	6	9	5	9	6	9	ST-41/44 complex	41	2	3	1
35682	M14 240271	UK [England]	2014	invasive (unspecified/other)	Neisseria meningitidis	B	B	B	1	6	3	100	7	8	10	5	4	5	3	8	ST-32 complex	34	7	1	1
35683	M14 240273	UK [Wales]	2014	invasive (unspecified/other)	Neisseria meningitidis	B	B	B	62	35	8	221	31	4	10	5	26	6	3	8	ST-32 complex	1096	NA	NA	1
35684	M14 240276	UK [England]	2014	invasive (unspecified/other)	Neisseria meningitidis	Y	Y	Y	25	1	1	26	4	12	5	18	9	11	9	17	ST-23 complex	1655	4	4	2
35685	M14 240277	UK [England]	2014	invasive (unspecified/other)	Neisseria meningitidis	Y	Y	Y	23	1	1	4	4	2	7	6	9	16	9	8	ST-167 complex	767	4	4	2
35686	M14 240278	UK [England]	2014	invasive (unspecified/other)	Neisseria meningitidis	W	W	W	22	1	1	1	4	2	3	4	3	8	4	6	ST-11 complex	11	4	4	2
35687	M14 240279	UK [England]	2014	invasive (unspecified/other)	Neisseria meningitidis	W	W	W	22	1	1	1	4	662	3	4	3	8	4	6	ST-11 complex	10651	4	4	2
35688	M14 240280	UK [England]	2014	invasive (unspecified/other)	Neisseria meningitidis	C	C	C	22	1	1	1	4	2	3	4	3	8	4	6	ST-11 complex	11	4	4	2
35689	M14 240282	UK [England]	2014	invasive (unspecified/other)	Neisseria meningitidis	W	W	W	22	1	1	1	4	2	3	4	3	8	4	6	ST-11 complex	11	4	4	2
35690	M14 240283	UK [England]	2014	invasive (unspecified/other)	Neisseria meningitidis	W	W	W	22	1	1	1	4	2	3	4	3	8	4	6	ST-11 complex	11	4	4	2
35691	M14 240284	UK [England]	2014	invasive (unspecified/other)	Neisseria meningitidis	Y	Y	Y	25	1	1	26	4	12	5	NA	9	11	9	17		NA	4	4	2
35692	M14 240285	UK [England]	2014	invasive (unspecified/other)	Neisseria meningitidis	Y	Y	Y	25	1	1	26	4	12	5	18	9	11	9	17	ST-23 complex	1655	4	4	2
35693	M14 240286	UK [England]	2014	invasive (unspecified/other)	Neisseria meningitidis	C	C	C	0	5	1	917	11	2	3	4	3	8	4	6	ST-11 complex	11	11	2	NA
35694	M14 240287	UK [England]	2014	invasive (unspecified/other)	Neisseria meningitidis	Y	Y	Y	25	1	1	26	4	12	5	18	9	11	9	17	ST-23 complex	1655	4	4	2
35695	M14 240289	UK [England]	2014	invasive (unspecified/other)	Neisseria meningitidis	B	B	B	14	1	1	142	13	3	5	4	5	9	26	156		5391	13	3	1
35696	M14 240291	UK [England]	2014	invasive (unspecified/other)	Neisseria meningitidis	Y	Y	Y	25	1	1	26	4	12	5	18	9	11	9	17	ST-23 complex	1655	4	4	2
35697	M14 240293	UK [England]	2014	invasive (unspecified/other)	Neisseria meningitidis	Y	Y	Y	16	1	1	60	4	11	5	18	17	11	24	21	ST-22 complex	114	4	4	2
35698	M14 240295	UK [England]	2014	invasive (unspecified/other)	Neisseria meningitidis	B	B	B	13	3	1	209	3	8	5	9	17	3	26	2	ST-60 complex	11296	3	3	1
35699	M14 240296	UK [England]	2014	invasive (unspecified/other)	Neisseria meningitidis	B	B	B	144	1	4	232	4	8	10	4	4	6	3	8	ST-32 complex	4786	4	3	1
35700	M14 240299	UK [England]	2014	invasive (unspecified/other)	Neisseria meningitidis	W	W	W	22	1	1	1	4	662	3	4	3	8	4	6	ST-11 complex	10651	4	4	2
35701	M14 240300	UK [England]	2014	invasive (unspecified/other)	Neisseria meningitidis	B	B	B	19	1	1	83	4	4	10	2	5	38	11	9	ST-269 complex	275	4	4	2
35702	M14 240304	UK [England]	2014	invasive (unspecified/other)	Neisseria meningitidis	W	W	W	22	1	1	1	4	2	3	4	3	8	4	6	ST-11 complex	11	4	4	2
35703	M14 240305	UK [England]	2014	invasive (unspecified/other)	Neisseria meningitidis	B	B	B	45	8	77	87	20	7	5	1	13	36	53	15	ST-213 complex	213	20	4	3
35704	M14 240306	UK [England]	2014	invasive (unspecified/other)	Neisseria meningitidis	B	B	B	14	5	1	204	11	3	6	19	5	17	6	9	ST-41/44 complex	8511	11	2	1
35705	M14 240309	UK [England]	2014	invasive (unspecified/other)	Neisseria meningitidis	B	B	B	14	1	1	142	13	12	6	9	5	723	6	9	ST-41/44 complex	11297	13	3	1
35706	M14 240310	UK [England]	2014	invasive (unspecified/other)	Neisseria meningitidis	C	C	C	78	5	1	245	11	2	3	4	3	8	4	6	ST-11 complex	11	11	2	1
35707	M14 240311	UK [England]	2014	invasive (unspecified/other)	Neisseria meningitidis	C	C	C	19	1	1	65	4	13	5	15	9	3	736	18	ST-334 complex	11298	4	4	2
35708	M14 240312	UK [England]	2014	invasive (unspecified/other)	Neisseria meningitidis	B	B	B	19	1	1	65	4	3	6	9	5	14	25	9	ST-41/44 complex	2922	4	4	2
35709	M14 240313	UK [England]	2014	invasive (unspecified/other)	Neisseria meningitidis	W/Y	W/Y	W/Y	25	1	1	26	4	12	5	18	9	11	9	17	ST-23 complex	1655	4	4	2
35710	M14 240314	UK [England]	2014	invasive (unspecified/other)	Neisseria meningitidis	B	B	B	13	3	1	145	3	17	5	19	17	3	26	2	ST-60 complex	60	3	3	1
35711	M14 240315	UK [England]	2014	invasive (unspecified/other)	Neisseria meningitidis	B	B	B	16	1	4	59	4	132	3	19	32	261	21	2		11429	4	4	2
35712	M14 240316	UK [England]	2014	invasive (unspecified/other)	Neisseria meningitidis	B	B	B	45	8	77	87	20	7	5	1	13	36	53	15	ST-213 complex	213	20	4	3
35713	M14 240317	UK [England]	2014	invasive (unspecified/other)	Neisseria meningitidis	B	B	B	47	1	57	90	15	12	5	12	35	192	22	17	ST-461 complex	1946	15	4	3
35714	M14 240319	UK [England]	2014	invasive (unspecified/other)	Neisseria meningitidis	B	B	B	19	108	77	83	183	4	6	2	17	3	11	9		11299	NA	NA	2
35715	M14 240321	UK [England]	2014	invasive (unspecified/other)	Neisseria meningitidis	B	B	B	45	14	77	87	45	7	5	1	13	21	53	15	ST-213 complex	11300	NA	NA	3
35716	M14 240325	UK [England]	2014	invasive (unspecified/other)	Neisseria meningitidis	B	B	B	824	46	57	472	184	4	10	34	5	38	11	9	ST-269 complex	1161	NA	NA	3
35717	M14 240327	UK [England]	2014	invasive (unspecified/other)	Neisseria meningitidis	Y	Y	Y	24	1	59	25	4	11	7	18	9	26	15	9		910	4	4	2
35718	M14 240328	UK [England]	2014	invasive (unspecified/other)	Neisseria meningitidis	B	B	B	30	17	11	54	37	7	5	1	13	36	53	15	ST-213 complex	213	NA	NA	3
31162	M14 240329	UK [England]	2014	invasive (unspecified/other)	Neisseria meningitidis	B	B	B	785	5	1	311	11	2	3	4	3	8	4	6	ST-11 complex	11	11	2	1
35719	M14 240331	UK [England]	2014	invasive (unspecified/other)	Neisseria meningitidis	B	B	B	4	2	79	84	2	3	16	325	5	9	6	9	ST-41/44 complex	5981	2	3	1
35720	M14 240333	UK [England]	2014	invasive (unspecified/other)	Neisseria meningitidis	B	B	B	829	15	4	473	185	25	6	470	58	58	11	9		11301	NA	NA	1
35721	M14 240335	UK [England]	2014	invasive (unspecified/other)	Neisseria meningitidis	Y	Y	Y	25	1	1	26	4	12	5	18	9	6	9	17	ST-23 complex	11331	4	4	2
35722	M14 240337	UK [England]	2014	invasive (unspecified/other)	Neisseria meningitidis	W	W	W	22	1	1	1	4	2	3	4	3	8	4	6	ST-11 complex	11	4	4	2
35723	M14 240338	UK [England]	2014	invasive (unspecified/other)	Neisseria meningitidis	B	B	B	19	109	1	83	186	4	10	2	5	38	11	1		1159	NA	NA	2
35724	M14 240342	UK [England]	2014	invasive (unspecified/other)	Neisseria meningitidis	B	B	B	1	6	3	100	7	8	35	5	4	5	3	8	ST-32 complex	11302	7	1	1
35725	M14 240343	UK [England]	2014	invasive (unspecified/other)	Neisseria meningitidis	W	W	W	22	1	1	1	4	2	3	4	3	8	4	6	ST-11 complex	11	4	4	2
35726	M14 240344	UK [England]	2014	invasive (unspecified/other)	Neisseria meningitidis	B	B	B	19	1	1	83	4	4	10	2	5	38	11	9	ST-269 complex	275	4	4	2
35727	M14 240345	UK [Wales]	2014	invasive (unspecified/other)	Neisseria meningitidis	B	B	B	47	1	57	90	15	12	5	12	35	192	22	17	ST-461 complex	1946	15	4	3
35728	M14 240346	UK [England]	2014	invasive (unspecified/other)	Neisseria meningitidis	W	W	W	22	1	1	1	4	2	3	4	3	8	4	6	ST-11 complex	11	4	4	2
35729	M14 240347	UK [Northern Ireland]	2014	invasive (unspecified/other)	Neisseria meningitidis	B	B	B	19	1	1	65	4	3	6	9	NA	9	6	9		NA	4	4	2
35730	M14 240350	UK [England]	2014	invasive (unspecified/other)	Neisseria meningitidis	B	B	B	1	6	3	100	7	8	10	77	25	6	3	22		5151	7	1	1
35731	M14 240351	UK [Wales]	2014	invasive (unspecified/other)	Neisseria meningitidis	W	W	W	22	1	1	1	4	2	3	4	3	8	4	6	ST-11 complex	11	4	4	2
35732	M14 240353	UK [England]	2014	invasive (unspecified/other)	Neisseria meningitidis	B	B	B	4	2	1	84	2	3	6	34	5	11	6	9	ST-41/44 complex	1194	2	3	1
35733	M14 240354	UK [England]	2014	invasive (unspecified/other)	Neisseria meningitidis	B	B	B	45	8	77	87	20	7	5	1	13	36	53	15	ST-213 complex	213	20	4	3
35734	M14 240356	UK [Wales]	2014	invasive (unspecified/other)	Neisseria meningitidis	B	B	B	47	1	57	90	15	12	5	12	35	60	22	17	ST-461 complex	461	15	4	3
35735	M14 240357	UK [Wales]	2014	invasive (unspecified/other)	Neisseria meningitidis	B	B	B	14	7	1	142	6	3	6	9	5	9	6	9	ST-41/44 complex	41	6	2	1
35736	M14 240359	UK [Wales]	2014	invasive (unspecified/other)	Neisseria meningitidis	B	B	B	31	49	1	124	83	7	5	9	25	17	25	15		11303	NA	NA	3
35737	M14 240360	UK [England]	2014	invasive (unspecified/other)	Neisseria meningitidis	B	B	B	19	1	1	83	4	4	10	2	35	38	11	9	ST-269 complex	11304	4	4	2
35738	M14 240361	UK [England]	2014	invasive (unspecified/other)	Neisseria meningitidis	W	W	W	16	1	1	60	4	11	5	NA	8	11	24	21		NA	4	4	2
35739	M14 240365	UK [England]	2014	invasive (unspecified/other)	Neisseria meningitidis	B	B	B	187	1	57	152	15	12	5	12	35	60	22	17	ST-461 complex	461	15	4	3
35420	M14 240366	UK	2014	invasive (unspecified/other)	Neisseria meningitidis	W		W	16	1	1	60	4	11	5	NA	8	11	24	21		NA	4	4	2
35740	M14 240367	UK [England]	2014	invasive (unspecified/other)	Neisseria meningitidis	B	B	B	15	10	2	193	17	4	10	15	9	8	11	9	ST-269 complex	269	NA	NA	1
35741	M14 240368	UK [England]	2014	invasive (unspecified/other)	Neisseria meningitidis	B	B	B	14	1	1	142	13	3	6	NA	5	83	6	9		NA	13	3	1
35742	M14 240369	UK [England]	2014	invasive (unspecified/other)	Neisseria meningitidis	B	B	B	4	2	1	84	2	6	3	6	17	26	21	8		4051	2	3	1
35743	M14 240372	UK [England]	2014	invasive (unspecified/other)	Neisseria meningitidis	B	B	B	885	115	77	499	149	7	5	1	13	36	53	15	ST-213 complex	213	NA	NA	3
35744	M14 240374	UK [England]	2014	invasive (unspecified/other)	Neisseria meningitidis	B	B	B	366	110	63	474	150	27	6	0	3	9	6	16		NA	NA	NA	3
35745	M14 240377	UK [England]	2014	invasive (unspecified/other)	Neisseria meningitidis	W	W	W	22	1	1	1	4	NA	3	4	3	8	4	6		NA	4	4	2
35746	M14 240378	UK [Wales]	2014	invasive (unspecified/other)	Neisseria meningitidis	Y	Y	Y	25	1	1	26	4	12	5	18	9	11	9	17	ST-23 complex	1655	4	4	2
35747	M14 240379	UK [England]	2014	invasive (unspecified/other)	Neisseria meningitidis	B	B	B	499	24	77	460	115	7	5	1	13	36	53	15	ST-213 complex	213	NA	NA	3
31163	M14 240380	UK [England]	2014	invasive (unspecified/other)	Neisseria meningitidis	B	B	B	4	2	1	84	2	3	6	9	5	8	6	9	ST-41/44 complex	485	2	3	1
38119	M14 240380b	UK [England]	2014	invasive (unspecified/other)	Neisseria meningitidis	B	B	B	4	NA	1	84	NA	3	6	9	5	8	6	9	ST-41/44 complex	485	NA	NA	1
35748	M14 240382	UK [England]	2014	invasive (unspecified/other)	Neisseria meningitidis	B	B	B	19	1	1	83	4	4	10	2	5	38	11	2		8061	4	4	2
35749	M14 240383	UK [England]	2014	invasive (unspecified/other)	Neisseria meningitidis	B	B	B	10	5	1	199	11	8	10	5	4	6	3	8	ST-32 complex	33	11	2	1
35750	M14 240384	UK [England]	2014	invasive (unspecified/other)	Neisseria meningitidis	B	B	B	47	1	57	90	15	12	5	12	35	192	22	17	ST-461 complex	1946	15	4	3
35751	M14 240386	UK [England]	2014	invasive (unspecified/other)	Neisseria meningitidis	B	B	B	119	1	1	372	4	8	3	154	17	26	21	NA		NA	4	4	2
35752	M14 240387	UK [England]	2014	invasive (unspecified/other)	Neisseria meningitidis	B	B	B	14	1	1	142	13	3	6	19	5	3	6	9	ST-41/44 complex	340	13	3	1
35753	M14 240388	UK [England]	2014	invasive (unspecified/other)	Neisseria meningitidis	B	B	B	4	2	1	84	2	3	6	0	5	8	6	9		NA	2	3	1
35754	M14 240389	UK [England]	2014	invasive (unspecified/other)	Neisseria meningitidis	W	W	W	22	1	1	1	4	2	3	4	3	8	4	6	ST-11 complex	11	4	4	2
35755	M14 240390	UK [England]	2014	invasive (unspecified/other)	Neisseria meningitidis	B	B	B	13	3	57	145	3	4	10	34	5	38	11	9	ST-269 complex	1161	3	3	1
35756	M14 240391	UK [England]	2014	invasive (unspecified/other)	Neisseria meningitidis	W		W	22	1	1	1	4	2	3	4	3	8	4	6	ST-11 complex	11	4	4	2
35757	M14 240393	UK [Wales]	2014	invasive (unspecified/other)	Neisseria meningitidis	B	B	B	45	11	77	87	10	7	5	1	13	36	53	15	ST-213 complex	213	NA	NA	3
37670	M14 240394	UK [England]	2014	invasive (unspecified/other)	Neisseria meningitidis	W	W	W	22	1	1	1	4	2	3	4	3	8	4	6	ST-11 complex	11	4	4	2
35758	M14 240396	UK [Wales]	2014	invasive (unspecified/other)	Neisseria meningitidis	B	B	B	19	1	1	65	4	12	6	9	9	9	6	9	ST-41/44 complex	43	4	4	2
35759	M14 240400	UK [England]	2014	invasive (unspecified/other)	Neisseria meningitidis	B	B	B	608	2	1	277	2	3	6	34	5	11	6	9	ST-41/44 complex	1194	2	3	1
35760	M14 240403	UK [England]	2014	invasive (unspecified/other)	Neisseria meningitidis	Y	Y	Y	25	1	1	26	4	12	5	18	NA	11	9	17		NA	4	4	2
35761	M14 240404	UK [England]	2014	invasive (unspecified/other)	Neisseria meningitidis	Y	Y	Y	25	1	1	26	4	10	5	18	9	11	9	17	ST-23 complex	23	4	4	2
35762	M14 240405	UK [Northern Ireland]	2014	invasive (unspecified/other)	Neisseria meningitidis	C	C	C	22	1	1	1	4	2	3	4	3	8	4	6	ST-11 complex	11	4	4	2
35763	M14 240406	UK [England]	2014	invasive (unspecified/other)	Neisseria meningitidis	W	W	W	22	1	1	1	4	2	3	4	3	8	4	6	ST-11 complex	11	4	4	2
35764	M14 240407	UK [England]	2014	invasive (unspecified/other)	Neisseria meningitidis	B	B	B	14	7	1	257	6	10	6	0	5	11	6	9		NA	6	2	1
35765	M14 240408	UK [England]	2014	invasive (unspecified/other)	Neisseria meningitidis	B	B	B	15	4	2	193	1	4	10	15	9	8	11	9	ST-269 complex	269	1	1	1
35766	M14 240410	UK [England]	2014	invasive (unspecified/other)	Neisseria meningitidis	B	B	B	4	2	1	84	2	3	6	9	5	8	6	9	ST-41/44 complex	485	2	3	1
35767	M14 240423	UK [England]	2014	invasive (unspecified/other)	Neisseria meningitidis	Y		Y	25	1	1	26	4	12	5	18	9	11	9	17	ST-23 complex	1655	4	4	2
35768	M14 240425	UK [Wales]	2014	invasive (unspecified/other)	Neisseria meningitidis	B	B	B	4	2	1	84	2	3	5	34	5	11	6	9	ST-41/44 complex	5986	2	3	1
35769	M14 240426	UK [England]	2014	invasive (unspecified/other)	Neisseria meningitidis	B	B	B	4	NA	79	84	NA	3	6	9	5	9	6	9	ST-41/44 complex	41	NA	NA	1
35770	M14 240428	UK [England]	2014	invasive (unspecified/other)	Neisseria meningitidis	B	B	B	13	3	57	145	3	4	10	34	5	38	11	9	ST-269 complex	1161	3	3	1
35771	M14 240429	UK [England]	2014	invasive (unspecified/other)	Neisseria meningitidis	B	B	B	4	2	1	84	2	3	6	9	5	8	6	9	ST-41/44 complex	485	2	3	1
35772	M14 240431	UK [England]	2014	invasive (unspecified/other)	Neisseria meningitidis	Y	Y	Y	25	1	1	26	4	12	5	NA	9	11	9	17		NA	4	4	2
35773	M14 240432	UK [England]	2014	invasive (unspecified/other)	Neisseria meningitidis	Y	Y	Y	25	1	1	26	4	10	5	NA	9	11	9	17		NA	4	4	2
35774	M14 240434	UK [England]	2014	invasive (unspecified/other)	Neisseria meningitidis	B	B	B	15	4	2	193	1	4	10	15	9	8	11	9	ST-269 complex	269	1	1	1
35775	M14 240436	UK [England]	2014	invasive (unspecified/other)	Neisseria meningitidis	C	C	C	22	1	1	1	4	2	3	4	3	8	4	6	ST-11 complex	11	4	4	2
35776	M14 240437	UK [England]	2014	invasive (unspecified/other)	Neisseria meningitidis	B	B	B	13	3	57	209	3	8	25	7	17	21	26	49	ST-1157 complex	1157	3	3	1
37671	M14 240439	UK [Northern Ireland]	2014	invasive (unspecified/other)	Neisseria meningitidis	NG		NG	23	1	1	4	4	2	7	6	9	16	9	8	ST-167 complex	767	4	4	2
35777	M14 240441	UK [England]	2014	invasive (unspecified/other)	Neisseria meningitidis	C	C	C	101	38	1	8	42	25	5	772	32	21	13	20	ST-116 complex	11332	NA	NA	2
31168	M14 240447	UK [England]	2014	invasive (unspecified/other)	Neisseria meningitidis	W	W	W	22	1	1	1	4	2	3	4	3	8	4	6	ST-11 complex	11	4	4	2
38120	M14 240447b	UK [England]	2014	invasive (unspecified/other)	Neisseria meningitidis	W		W	22	1	1	1	4	2	3	4	3	8	4	6	ST-11 complex	11	4	4	2
31169	M14 240448	UK [England]	2014	invasive (unspecified/other)	Neisseria meningitidis	W	W	W	22	1	1	1	4	2	3	4	3	8	4	6	ST-11 complex	11	4	4	2

38121	M14 240448b	UK [England]	2014	invasive (unspecified/other)	Neisseria meningitidis	W		W	22	1	1	1	4	2	3	4	3	8	4	6	ST-11 complex	11	4	4	2
35778	M14 240451	UK [England]	2014	invasive (unspecified/other)	Neisseria meningitidis	B	B	B	19	1	1	83	4	4	10	2	721	38	11	9	ST-269 complex	11305	4	4	2
35779	M14 240452	UK [Northern Ireland]	2014	invasive (unspecified/other)	Neisseria meningitidis	B	B	B	15	4	2	193	1	2	10	15	9	8	11	17	ST-269 complex	1214	1	1	1
35780	M14 240455	UK [England]	2014	invasive (unspecified/other)	Neisseria meningitidis	B	B	B	4	2	1	84	2	3	6	9	5	8	6	9	ST-41/44 complex	485	2	3	1
35781	M14 240456	UK [England]	2014	invasive (unspecified/other)	Neisseria meningitidis	B	B	B	13	3	57	145	3	12	5	12	35	192	22	17	ST-461 complex	1946	3	3	1
35782	M14 240463	UK [England]	2014	invasive (unspecified/other)	Neisseria meningitidis	W	W	W	22	1	1	1	4	2	3	4	3	8	4	6	ST-11 complex	11	4	4	2
35783	M14 240465	UK [England]	2014	invasive (unspecified/other)	Neisseria meningitidis	B	B	B	13	3	57	145	3	4	10	34	5	38	737	9		11306	3	3	1
35784	M14 240466	UK [England]	2014	invasive (unspecified/other)	Neisseria meningitidis	B	B	B	15	4	2	193	1	4	491	2	5	38	11	9		11307	1	1	1
35785	M14 240467	UK [Wales]	2014	invasive (unspecified/other)	Neisseria meningitidis	B	B	B	1	6	3	100	7	8	10	5	4	6	3	15	ST-32 complex	8049	7	1	1
35786	M14 240468	UK [England]	2014	invasive (unspecified/other)	Neisseria meningitidis	W	W	W	22	1	1	1	4	2	3	4	3	8	4	6	ST-11 complex	11	4	4	2
35787	M14 240469	UK [England]	2014	invasive (unspecified/other)	Neisseria meningitidis	B	B	B	825	4	99	475	1	4	10	393	9	8	5	9	ST-269 complex	11308	1	1	1
35788	M14 240470	UK [England]	2014	invasive (unspecified/other)	Neisseria meningitidis	W	W	W	22	1	1	1	4	2	3	4	3	8	4	6	ST-11 complex	11	4	4	2
35789	M14 240471	UK [England]	2014	invasive (unspecified/other)	Neisseria meningitidis	B	B	B	14	7	1	142	6	3	6	9	5	26	6	9	ST-41/44 complex	8054	6	2	1
35790	M14 240472	UK [England]	2014	invasive (unspecified/other)	Neisseria meningitidis	B	B	B	14	7	1	142	6	3	6	9	5	26	6	9	ST-41/44 complex	8054	6	2	1
35791	M14 240473	UK [England]	2014	invasive (unspecified/other)	Neisseria meningitidis	W	W	W	16	1	1	60	4	11	5	18	8	11	24	21	ST-22 complex	22	4	4	2
35792	M14 240474	UK [England]	2014	invasive (unspecified/other)	Neisseria meningitidis	W	W	W	22	1	1	1	4	2	3	4	3	8	4	6	ST-11 complex	11	4	4	2
35793	M14 240475	UK [England]	2014	invasive (unspecified/other)	Neisseria meningitidis	B	B	B	822	36	2	476	35	716	6	9	9	9	6	9	ST-41/44 complex	11309	NA	NA	1
35794	M14 240476	UK [England]	2014	invasive (unspecified/other)	Neisseria meningitidis	B	B	B	14	5	1	204	11	3	6	19	5	3	6	9	ST-41/44 complex	340	11	2	1
35795	M14 240477	UK [England]	2014	invasive (unspecified/other)	Neisseria meningitidis	B	B	B	4	2	1	84	2	3	6	9	5	8	6	9	ST-41/44 complex	485	2	3	1
35796	M14 240478	UK [England]	2014	invasive (unspecified/other)	Neisseria meningitidis	W	W	W	22	1	1	1	4	2	3	4	3	8	4	6	ST-11 complex	11	4	4	2
35797	M14 240480	UK [England]	2014	invasive (unspecified/other)	Neisseria meningitidis	B	B	B	1	6	3	100	7	8	35	5	4	5	3	8	ST-32 complex	11302	7	1	1
35798	M14 240481	UK [England]	2014	invasive (unspecified/other)	Neisseria meningitidis	B	B	B	21	1	1	7	4	1	5	13	53	26	41	3	ST-162 complex	162	4	4	2
35799	M14 240482	UK [England]	2014	invasive (unspecified/other)	Neisseria meningitidis	W	W	W	22	1	1	1	4	2	3	4	3	8	4	6	ST-11 complex	11	4	4	2
35800	M14 240485	UK [England]	2014	invasive (unspecified/other)	Neisseria meningitidis	W	W	W	22	1	1	1	4	2	3	4	3	8	4	6	ST-11 complex	11	4	4	2
35801	M14 240486	UK [England]	2014	invasive (unspecified/other)	Neisseria meningitidis	W	W	W	22	1	1	1	4	2	3	4	3	8	4	6	ST-11 complex	11	4	4	2
35802	M14 240487	UK [England]	2014	invasive (unspecified/other)	Neisseria meningitidis	Y	Y	Y	25	1	1	26	4	12	5	18	9	11	9	17	ST-23 complex	1655	4	4	2
35803	M14 240488	UK [England]	2014	invasive (unspecified/other)	Neisseria meningitidis	B	B	B	683	4	2	176	1	9	5	9	9	9	6	2	ST-41/44 complex	1423	1	1	2
35804	M14 240489	UK [England]	2014	invasive (unspecified/other)	Neisseria meningitidis	Y	Y	Y	25	1	1	26	4	12	5	18	9	11	9	17	ST-23 complex	1655	4	4	2
35805	M14 240490	UK [England]	2014	invasive (unspecified/other)	Neisseria meningitidis	B	B	B	14	5	1	204	11	3	6	19	5	3	6	9	ST-41/44 complex	340	11	2	1
35806	M14 240491	UK [England]	2014	invasive (unspecified/other)	Neisseria meningitidis	W	W	W	22	1	1	1	4	662	3	4	3	8	4	6	ST-11 complex	10651	4	4	2
35807	M14 240492	UK [England]	2014	invasive (unspecified/other)	Neisseria meningitidis	W	W	W	22	1	1	1	4	662	3	4	3	8	4	6	ST-11 complex	10651	4	4	2
35808	M14 240494	UK [England]	2014	invasive (unspecified/other)	Neisseria meningitidis	W	W	W	22	1	1	1	4	2	3	4	3	8	4	6	ST-11 complex	11	4	4	2
35809	M14 240495	UK [England]	2014	invasive (unspecified/other)	Neisseria meningitidis	W	W	W	22	1	1	1	4	2	3	4	3	8	4	6	ST-11 complex	11	4	4	2
35810	M14 240496	UK [England]	2014	invasive (unspecified/other)	Neisseria meningitidis	W	W	W	22	1	1	1	4	2	3	4	3	8	4	6	ST-11 complex	11	4	4	2
35811	M14 240500	UK [England]	2014	invasive (unspecified/other)	Neisseria meningitidis	W	W	W	22	1	1	1	4	2	3	4	3	8	4	6	ST-11 complex	11	4	4	2
37672	M14 240501	UK [England]	2014	invasive (unspecified/other)	Neisseria meningitidis	Y	NG	discrepancy	25	1	1	26	4	12	5	18	9	11	9	17	ST-23 complex	1655	4	4	2
37673	M14 240502	UK [England]	2014	invasive (unspecified/other)	Neisseria meningitidis	W		W	22	1	1	1	4	662	3	4	3	8	4	6	ST-11 complex	10651	4	4	2
37674	M14 240503	UK [England]	2014	invasive (unspecified/other)	Neisseria meningitidis	B	B	B	19	1	1	83	4	4	10	2	5	38	11	9	ST-269 complex	275	4	4	2
37675	M14 240504	UK [England]	2014	invasive (unspecified/other)	Neisseria meningitidis	W		W	22	1	1	1	4	662	3	4	3	8	4	6	ST-11 complex	10651	4	4	2
37676	M14 240506	UK [England]	2014	invasive (unspecified/other)	Neisseria meningitidis	Y		Y	24	1	1	27	4	2	7	6	17	9	6	8	ST-167 complex	1624	4	4	2
37677	M14 240507	UK [England]	2014	invasive (unspecified/other)	Neisseria meningitidis	B	B	B	19	1	1	83	4	4	10	2	5	38	11	9	ST-269 complex	275	4	4	2
37678	M14 240508	UK [England]	2014	invasive (unspecified/other)	Neisseria meningitidis	B	B	B	19	1	1	65	4	9	20	9	9	9	6	2	ST-41/44 complex	1097	4	4	2
37679	M14 240509	UK [England]	2014	invasive (unspecified/other)	Neisseria meningitidis	C	C	C	13	5	1	251	11	2	3	4	3	8	4	6	ST-11 complex	11	11	2	1
37680	M14 240510	UK [England]	2014	invasive (unspecified/other)	Neisseria meningitidis	B	B	B	85	9	77	509	21	7	5	5	13	8	53	15	ST-213 complex	11465	NA	NA	3
37681	M14 240511	UK [England]	2014	invasive (unspecified/other)	Neisseria meningitidis	W		W	22	1	1	1	4	2	3	4	3	8	4	6	ST-11 complex	11	4	4	2
37682	M14 240513	UK [England]	2014	invasive (unspecified/other)	Neisseria meningitidis	B	B	B	224	15	1	252	68	4	10	5	4	5	3	8	ST-32 complex	2506	NA	NA	1
37683	M14 240514	UK [England]	2014	invasive (unspecified/other)	Neisseria meningitidis	B	B	B	309	1	1	244	4	1	5	13	53	26	41	22	ST-162 complex	10817	4	3	1
38127	M14 240515	UK	2014	invasive (unspecified/other)	Neisseria meningitidis	W	W	W	22	1	1	1	4	2	3	4	3	8	4	6	ST-11 complex	11	4	4	2
37684	M14 240516	UK [England]	2014	invasive (unspecified/other)	Neisseria meningitidis	B	B	B	4	2	1	84	2	3	6	34	5	11	6	9	ST-41/44 complex	1194	2	3	1
37685	M14 240517	UK [England]	2014	invasive (unspecified/other)	Neisseria meningitidis	W	W	W	886	24	1	510	115	2	3	4	3	8	4	6	ST-11 complex	11	NA	NA	2
37686	M14 240518	UK [England]	2014	invasive (unspecified/other)	Neisseria meningitidis	B	B	B	4	2	4	84	2	3	5	9	5	9	6	9	ST-41/44 complex	3754	2	3	1
37687	M14 240519	UK [Wales]	2014	invasive (unspecified/other)	Neisseria meningitidis	C	C	C	1	6	7	100	7	4	10	5	4	6	3	8	ST-32 complex	32	7	1	1
37688	M14 240520	UK [England]	2014	invasive (unspecified/other)	Neisseria meningitidis	B	B	B	61	5	102	237	90	4	10	2	5	3	11	9	ST-269 complex	1163	NA	NA	1
37689	M14 240521	UK [England]	2014	invasive (unspecified/other)	Neisseria meningitidis	Y	Y	Y	25	1	1	26	4	12	5	18	9	11	9	786	ST-23 complex	11866	4	4	2
37690	M14 240522	UK [England]	2014	invasive (unspecified/other)	Neisseria meningitidis	W	W	W	22	1	1	1	4	2	3	4	3	8	4	6	ST-11 complex	11	4	4	2
37691	M14 240523	UK [England]	2014	invasive (unspecified/other)	Neisseria meningitidis	Y		Y	25	1	1	26	4	10	5	18	9	11	9	17	ST-23 complex	23	4	4	2
38118	M14 240523b	UK [England]	2014	invasive (unspecified/other)	Neisseria meningitidis	Y		Y	25	1	1	26	4	10	5	18	9	11	9	17	ST-23 complex	23	4	4	2
37692	M14 240524	UK [England]	2014	invasive (unspecified/other)	Neisseria meningitidis	W	W	W	22	1	1	1	4	2	3	4	3	8	4	6	ST-11 complex	11	4	4	2
37693	M14 240525	UK [England]	2014	invasive (unspecified/other)	Neisseria meningitidis	W	W	W	22	1	1	1	4	2	3	4	3	8	4	6	ST-11 complex	11	4	4	2
37694	M14 240526	UK [England]	2014	invasive (unspecified/other)	Neisseria meningitidis	Y	Y	Y	25	1	1	26	4	12	5	18	9	11	9	17	ST-23 complex	1655	4	4	2
37695	M14 240527	UK [England]	2014	invasive (unspecified/other)	Neisseria meningitidis	B	B	B	1	6	3	100	7	8	10	5	4	5	3	8	ST-32 complex	34	7	1	1
37696	M14 240528	UK [England]	2014	invasive (unspecified/other)	Neisseria meningitidis	B	B	B	1	6	7	100	7	4	10	5	4	6	3	8	ST-32 complex	32	7	1	1
37697	M14 240530	UK [England]	2014	invasive (unspecified/other)	Neisseria meningitidis	Y	Y	Y	25	1	1	26	4	10	5	314	9	11	9	17	ST-23 complex	3582	4	4	2
37698	M14 240531	UK [England]	2014	invasive (unspecified/other)	Neisseria meningitidis	B	B	B	15	10	2	193	17	4	10	15	9	8	11	9	ST-269 complex	269	NA	NA	1
37699	M14 240532	UK [England]	2014	invasive (unspecified/other)	Neisseria meningitidis	W		W	22	1	1	1	4	2	3	4	3	8	4	6	ST-11 complex	11	4	4	2
37700	M14 240533	UK [England]	2014	invasive (unspecified/other)	Neisseria meningitidis	B	B	B	4	2	4	84	2	3	5	9	5	9	6	9	ST-41/44 complex	3754	2	3	1
37701	M14 240534	UK [England]	2014	invasive (unspecified/other)	Neisseria meningitidis	W		W	22	1	1	1	4	2	3	4	3	8	4	6	ST-11 complex	11	4	4	2
37702	M14 240535	UK [England]	2014	invasive (unspecified/other)	Neisseria meningitidis	B	B	B	24	1	63	25	4	27	6	9	3	9	6	16	ST-41/44 complex	136	4	4	2
37703	M14 240536	UK [England]	2014	invasive (unspecified/other)	Neisseria meningitidis	Y	Y	Y	25	1	1	26	4	12	5	18	9	11	9	17	ST-23 complex	1655	4	4	2
37704	M14 240539	UK [England]	2014	invasive (unspecified/other)	Neisseria meningitidis	B	B	B	61	5	57	237	90	4	10	34	5	38	11	9	ST-269 complex	1161	NA	NA	1
37705	M14 240541	UK [England]	2014	invasive (unspecified/other)	Neisseria meningitidis	B	B	B	14	1	57	142	13	12	10	10	72	8	21	139		1111	13	3	1
37706	M14 240542	UK [England]	2014	invasive (unspecified/other)	Neisseria meningitidis	B	B	B	15	4	2	193	1	4	4	15	17	8	11	9	ST-269 complex	11466	1	1	1
37707	M14 240543	UK [England]	2014	invasive (unspecified/other)	Neisseria meningitidis	B	B	B	4	2	1	84	2	3	6	9	5	11	6	9	ST-41/44 complex	154	2	3	1
37708	M14 240545	UK [England]	2014	invasive (unspecified/other)	Neisseria meningitidis	W	W	W	22	1	1	1	4	2	3	4	3	8	4	6	ST-11 complex	11	4	4	2
37709	M14 240546	UK [England]	2014	invasive (unspecified/other)	Neisseria meningitidis	C	C	C	22	1	1	1	4	2	3	4	3	8	4	6	ST-11 complex	11	4	4	2
37710	M14 240548	UK [England]	2014	invasive (unspecified/other)	Neisseria meningitidis	W	W	W	22	1	1	1	4	2	3	4	3	8	4	6	ST-11 complex	11	4	4	2
37711	M14 240550	UK [England]	2014	invasive (unspecified/other)	Neisseria meningitidis	W	W	W	22	1	1	1	4	2	3	4	3	8	4	6	ST-11 complex	11	4	4	2
37712	M14 240551	UK [England]	2014	invasive (unspecified/other)	Neisseria meningitidis	B	B	B	4	2	1	84	2	3	6	34	26	11	6	9	ST-41/44 complex	11467	2	3	1
37713	M14 240552	UK [England]	2014	invasive (unspecified/other)	Neisseria meningitidis	Y		Y	13	3	1	145	3	2	7	6	NA	9	6	8		NA	3	3	1
37714	M14 240553	UK [England]	2014	invasive (unspecified/other)	Neisseria meningitidis	Y		Y	25	1	1	26	4	12	5	18	9	11	9	17	ST-23 complex	1655	4	4	2
37715	M14 240554	UK [England]	2014	invasive (unspecified/other)	Neisseria meningitidis	B	B	B	25	1	1	28	4	12	5	19	17	3	10	124	ST-60 complex	1754	4	4	2
37716	M14 240555	UK [England]	2014	invasive (unspecified/other)	Neisseria meningitidis	W	W	W	22	1	1	1	4	2	3	4	3	8	4	6	ST-11 complex	11	4	4	2
37717	M14 240556	UK [England]	2014	invasive (unspecified/other)	Neisseria meningitidis	W		W	22	1	1	1	4	2	3	4	3	8	4	6	ST-11 complex	11	4	4	2
37718	M14 240557	UK [England]	2014	invasive (unspecified/other)	Neisseria meningitidis	B	B	B	4	NA	1	84	NA	3	6	9	5	3	6	9	ST-41/44 complex	46	NA	NA	1
37719	M14 240558	UK [England]	2014	invasive (unspecified/other)	Neisseria meningitidis	B	B	B	19	1	1	65	4	9	20	9	9	9	6	2	ST-41/44 complex	1097	4	4	2
37720	M14 240560	UK [England]	2014	invasive (unspecified/other)	Neisseria meningitidis	B	B	B	1	6	3	100	7	8	10	77	25	6	3	22		5151	7	1	1
37721	M14 240561	UK [Wales]	2014	invasive (unspecified/other)	Neisseria meningitidis	B	B	B	13	3	57	209	3	12	5	12	35	192	22	651	ST-461 complex	11468	3	3	1
37722	M14 240562	UK [England]	2014	invasive (unspecified/other)	Neisseria meningitidis	W	W	W	22	1	1	1	4	2	3	4	3	8	4	6	ST-11 complex	11	4	4	2
37723	M14 240563	UK [England]	2014	invasive (unspecified/other)	Neisseria meningitidis	B	B	B	13	3	1	145	3	17	5	19	17	3	26	2	ST-60 complex	60	3	3	1
37724	M14 240564	UK [England]	2014	invasive (unspecified/other)	Neisseria meningitidis	B	B	B	14	7	1	142	6	3	6	9	5	9	6	9	ST-41/44 complex	41	6	2	1
37725	M14 240565	UK [England]	2014	invasive (unspecified/other)	Neisseria meningitidis	B	B	B	4	2	1	84	2	3	6	9	5	9	6	9	ST-41/44 complex	41	2	3	1
37726	M14 240566	UK [England]	2014	invasive (unspecified/other)	Neisseria meningitidis	B	B	B	13	3	77	145	3	7	5	1	13	9	53	15	ST-213 complex	3635	3	3	1
37727	M14 240567	UK [England]	2014	invasive (unspecified/other)	Neisseria meningitidis	B	B	B	887	18	1	511	87	9	3	9	9	9	64	9	ST-41/44 complex	6058	NA	NA	1
37728	M14 240568	UK [England]	2014	invasive (unspecified/other)	Neisseria meningitidis	C	C	C	22	1	1	1	4	2	3	4	3	8	4	6	ST-11 complex	11	4	4	2
37729	M14 240571	UK [England]	2014	invasive (unspecified/other)	Neisseria meningitidis	Y	Y	Y	21	1	1	7	4	6	5	173	13	5	24	17	ST-174 complex	1466	4	4	2
37730	M14 240572	UK [Northern Ireland]	2014	invasive (unspecified/other)	Neisseria meningitidis	W	W	W	22	1	1	1	4	2	3	4	3	8	4	6	ST-11 complex	11	4	4	2
37731	M14 240573	UK [England]	2014	invasive (unspecified/other)	Neisseria meningitidis	W	W	W	22	1	1	1	4	2	3	4	3	8	4	6	ST-11 complex	11	4	4	2
37732	M14 240574	UK [England]	2014	invasive (unspecified/other)	Neisseria meningitidis	W	W	W	22	1	1	1	4	2	3	4	3	8	4	6	ST-11 complex	11	4	4	2
37733	M14 240575	UK [England]	2014	invasive (unspecified/other)	Neisseria meningitidis	B	B	B	45	8	1	87	20	7	5	1	627	36	53	15	ST-213 complex	9881	20	4	3
37734	M14 240576	UK [England]	2014	invasive (unspecified/other)	Neisseria meningitidis	B	B	B	1	6	7	100	7	4	10	5	4	6	3	8	ST-32 complex	32	7	1	1
37735	M14 240580	UK [England]	2014	invasive (unspecified/other)	Neisseria meningitidis	B	B	B	316	27	2	155	26	222	3	58	261	263	5	255	ST-4821 complex	3200	NA	NA	3
37736	M14 240581	UK [England]	2014	invasive (unspecified/other)	Neisseria meningitidis	W	W	W	22	1	1	1	4	2	3	4	3	8	4	6	ST-11 complex	11	4	4	2
37737	M14 240582	UK [England]	2014	invasive (unspecified/other)	Neisseria meningitidis	B	B	B	19	1	1	65	4	9	6	9	9	9	64	9	ST-41/44 complex	409	4	4	2
37738	M14 240585	UK [England]	2014	invasive (unspecified/other)	Neisseria meningitidis	W	W	W	22	1	1	1	4	662	3	4	3	8	4	6	ST-11 complex	10651	4	4	2
37739	M14 240587	UK [England]	2014	invasive (unspecified/other)	Neisseria meningitidis	W	W	W	22	1	1	1	4	2	3	4	3	8	4	6	ST-11 complex	11	4	4	2
37740	M14 240590	UK [England]	2014	invasive (unspecified/other)	Neisseria meningitidis	W		W	16	1	1	60	4	11	5	18	8	11	4	21	ST-22 complex	184	4	4	2
37741	M14 240591	UK [England]	2014	invasive (unspecified/other)	Neisseria meningitidis	B	B	B	1	6	3	100	7	8	10	5	4	5	6	8	ST-32 complex	5342	7	1	1
37742	M14 240593	UK [England]	2014	invasive (unspecified/other)	Neisseria meningitidis	W		W	762	1	1	296	4	2	3	4	3	8	4	6	ST-11 complex	11	4	3	1
37743	M14 240595	UK [England]	2014	invasive (unspecified/other)	Neisseria meningitidis	B	B	B	4	2	1	84	2	3	6	9	5	9	6	9	ST-41/44 complex	41	2	3	1
37744	M14 240596	UK [England]	2014	invasive (unspecified/other)	Neisseria meningitidis	B	B	B	19	1	1	83	4	4	10	2	5	3	4	9		11469	4	4	2
37745	M14 240598	UK [England]	2014	invasive (unspecified/other)	Neisseria meningitidis	B	B	B	888	6	3	512	7	8	10	77	4	6	3	8	ST-32 complex	749	7	1	3
37746	M14 240601	UK [England]	2014	invasive (unspecified/other)	Neisseria meningitidis	W		W	22	1	1	1	4	2	3	4	756	8	4	6	ST-11 complex	11867	4	4	2
37747	M14 240602	UK [England]	2014	invasive (unspecified/other)	Neisseria meningitidis	W	W	W	22	1	1	1	4	2	3	4	3	8	4	6	ST-11 complex	11	4	4	2
37748	M14 240604	UK [England]	2014	invasive (unspecified/other)	Neisseria meningitidis	W	W	W	22	1	1	1	4	2	3	4	3	8	4	6	ST-11 complex	11	4	4	2
37749	M14 240605	UK [England]	2014	invasive (unspecified/other)	Neisseria meningitidis	W		W	22	1	1	1	4	662	3	4	3	8	4	6	ST-11 complex	10651	4	4	2
37750	M14 240606	UK [England]	2014	invasive (unspecified/other)	Neisseria meningitidis	B	NG	discrepancy	1	6	3	100	7	8	3	5	4	1	3	8	ST-32 complex	290	7	1	1
37751	M14 240607	UK [England]	2014	invasive (unspecified/other)	Neisseria meningitidis	Y		Y	25	1	1	26	4	10	5	18	9	11	9	17	ST-23 complex	23	4	4	2
37752	M14 240616	UK [England]	2014	invasive (unspecified/other)	Neisseria meningitidis	C	C	C	13	5	1	251	11	2	3	4	3	8	4	6	ST-11 complex	11	11	2	1
37753	M14 240617	UK [England]	2014	invasive (unspecified/other)	Neisseria meningitidis	B	B	B	15	4	2	193	1	2	10	15	9	8	11	17	ST-269 complex	1214	1	1	1
37754	M14 240618	UK [England]	2014	invasive (unspecified/other)	Neisseria meningitidis	W	W	W	22	1	1	1	4	2	3	4	3	8	4	6	ST-11 complex	11	4	4	2
37755	M14 240619	UK [England]	2014	invasive (unspecified/other)	Neisseria meningitidis	B	B	B	19	1	1	83	4	4	10	2	5	3	11	9	ST-269 complex	1163	4	4	2
37756	M14 240620	UK [England]	2014	invasive (unspecified/other)	Neisseria meningitidis	B	B	B	224	15	1	252	68	4	10	5	4	6	3	8	ST-32 complex	32	NA	NA	1
37757	M14 240621	UK [England]	2014	invasive (unspecified/other)	Neisseria meningitidis	W	W	W	22	1	1	1	4	2	3	4	3	8	4	6	ST-11 complex	11	4	4	2
37758	M14 240622	UK [England]	2014	invasive (unspecified/other)	Neisseria meningitidis	C	C	C	13	5	1	251	11	2	3	4	3	8	4	6	ST-11 complex	11	11	2	1
37759	M14 240623	UK [England]	2014	invasive (unspecified/other)	Neisseria meningitidis	B	B	B	1	6	3	100	7	8	10	5	9	6	3	8	ST-32 complex	639	7	1	1
37760	M14 240624	UK [England]	2014	invasive (unspecified/other)	Neisseria meningitidis	W	W	W	22	1	1	1	4	2	3	4	3	8	4	6	ST-11 complex	11	4	4	2
37761	M14 240625	UK [England]	2014	invasive (unspecified/other)	Neisseria meningitidis	B	B	B	4	2	1	84	2	3	6	9	5	9	6	9	ST-41/44 complex	41	2	3	1
37762	M14 240626	UK [England]	2014	invasive (unspecified/other)	Neisseria meningitidis	B	NG	discrepancy	15	4	2	193	1	4	10	15	8	8	11	17	ST-269 complex	11470	1	1	1
37763	M14 240627	UK [England]	2014	invasive (unspecified/other)	Neisseria meningitidis	C	C	C	13	5	1	251	11	2	3	4	3	8	4	6	ST-11 complex	11	11	2	1
37764	M14 240628	UK [Northern Ireland]	2014	invasive (unspecified/other)	Neisseria meningitidis	W		W	22	1	1	1	4	2	3	4	3	8	4	6	ST-11 complex	11	4	4	2
37765	M14 240629	UK [England]	2014	invasive (unspecified/other)	Neisseria meningitidis	Y		Y	25	1	1	26	4	10	5	314	9	11	9	787	ST-23 complex	11868	4	4	2
37766	M14 240630	UK [England]	2014	invasive (unspecified/other)	Neisseria meningitidis	B	B	B	170	9	10	94	21	4	10	3	5	3	11	9	ST-269 complex	11471	NA	NA	3
37767	M14 240631	UK [England]	2014	invasive (unspecified/other)	Neisseria meningitidis	B	B	B	4	2	1	84	2	503	6	9	5	9	6	9	ST-41/44 complex	8380	2	3	1
37768	M14 240632	UK [England]	2014	invasive (unspecified/other)	Neisseria meningitidis	Y		Y	25	1	1	26	4	12	5	18	9	9	9	17	ST-23 complex	11472	4	4	2
37769	M14 240633	UK [England]	2014	invasive (unspecified/other)	Neisseria meningitidis	W		W	22	1	1	1	4	2	3	4	3	8	4	6	ST-11 complex	11	4	4	2
37770	M14 240634	UK [England]	2014	invasive (unspecified/other)	Neisseria meningitidis	W	W	W	22	1	1	1	4	2	3	4	3	8	4	6	ST-11 complex	11	4	4	2
37771	M14 240635	UK [England]	2014	invasive (unspecified/other)	Neisseria meningitidis	Y	Y	Y	25	1	1	26	4	12	5	18	9	11	9	17	ST-23 complex	1655	4	4	2
37772	M14 240636	UK [England]	2014	invasive (unspecified/other)	Neisseria meningitidis	B	B	B	4	2	1	84	2	3	6	9	5	8	6	9	ST-41/44 complex	485	2	3	1

37773	M14 240637	UK [England]	2014	invasive (unspecified/other)	Neisseria meningitidis	B	B	B	37	1	1	408	4	7	8	10	19	10	1	2	ST-18 complex	18	4	3	1
37774	M14 240638	UK [England]	2014	invasive (unspecified/other)	Neisseria meningitidis	W	W	W	22	1	1	1	4	2	3	4	3	8	4	6	ST-11 complex	11	4	4	2
37775	M14 240639	UK [England]	2014	invasive (unspecified/other)	Neisseria meningitidis	B	B	B	4	2	6	84	2	3	6	9	5	9	6	9	ST-41/44 complex	41	2	3	1
37776	M14 240640	UK [England]	2014	invasive (unspecified/other)	Neisseria meningitidis	B	B	B	31	9	1	124	16	4	10	2	4	3	11	9	ST-269 complex	11473	16	2	3
37777	M14 240641	UK [England]	2014	invasive (unspecified/other)	Neisseria meningitidis	B	B	B	13	3	57	209	3	8	25	7	17	21	26	49	ST-1157 complex	1157	3	3	1
37779	M14 240643	UK [England]	2014	invasive (unspecified/other)	Neisseria meningitidis	B	B	B	889	2	1	513	2	748	6	9	5	9	6	9	ST-41/44 complex	11869	2	3	1
37780	M14 240645	UK [England]	2014	invasive (unspecified/other)	Neisseria meningitidis	B	B	B	47	1	57	90	15	12	5	12	35	192	22	17	ST-461 complex	1946	15	4	3
37781	M14 240646	UK [England]	2014	invasive (unspecified/other)	Neisseria meningitidis	B	B	B	13	3	57	145	3	4	10	34	5	38	11	9	ST-269 complex	1161	3	3	1
37782	M14 240647	UK [England]	2014	invasive (unspecified/other)	Neisseria meningitidis	B	B	B	13	3	57	145	3	4	10	2	5	38	11	9	ST-269 complex	275	3	3	1
37783	M14 240648	UK [England]	2014	invasive (unspecified/other)	Neisseria meningitidis	B	B	B	24	1	4	25	4	4	10	47	17	6	2	12	ST-35 complex	278	4	4	2
37784	M14 240649	UK [England]	2014	invasive (unspecified/other)	Neisseria meningitidis	W		W	22	1	1	1	4	2	3	4	3	8	4	6	ST-11 complex	11	4	4	2
37785	M14 240650	UK [England]	2014	invasive (unspecified/other)	Neisseria meningitidis	B	B	B	555	113	5	514	131	27	5	9	3	9	6	16		3989	NA	NA	3
37786	M14 240651	UK [England]	2014	invasive (unspecified/other)	Neisseria meningitidis	Y		Y	24	1	1	27	4	2	7	6	17	9	6	8	ST-167 complex	1624	4	4	2
37787	M14 240652	UK [England]	2014	invasive (unspecified/other)	Neisseria meningitidis	C	C	C	22	1	1	1	4	2	3	4	3	8	4	6	ST-11 complex	11	4	4	2
37788	M14 240653	UK [England]	2014	invasive (unspecified/other)	Neisseria meningitidis	B	B	B	45	61	77	87	151	7	5	1	13	36	53	15	ST-213 complex	213	NA	NA	3
37789	M15 240001	UK [England]	2015	invasive (unspecified/other)	Neisseria meningitidis	B	B	B	1	6	3	100	7	8	10	5	4	5	3	8	ST-32 complex	34	7	1	1
37790	M15 240002	UK [England]	2015	invasive (unspecified/other)	Neisseria meningitidis	B	B	B	13	3	57	145	3	4	10	34	5	38	11	9	ST-269 complex	1161	3	3	1
37791	M15 240003	UK [England]	2015	invasive (unspecified/other)	Neisseria meningitidis	B	B	B	19	1	2	65	4	4	10	15	9	8	11	20	ST-269 complex	1354	4	4	2
37792	M15 240004	UK [England]	2015	invasive (unspecified/other)	Neisseria meningitidis	B	B	B	19	1	1	65	4	8	25	7	17	21	26	49	ST-1157 complex	1157	4	4	2
37793	M15 240005	UK [England]	2015	invasive (unspecified/other)	Neisseria meningitidis	W	W	W	22	1	1	1	4	2	3	4	3	8	4	6	ST-11 complex	11	4	4	2
37794	M15 240006	UK [England]	2015	invasive (unspecified/other)	Neisseria meningitidis	Y	NG	discrepancy	25	1	1	26	4	12	5	18	9	11	9	17	ST-23 complex	1655	4	4	2
37795	M15 240007	UK [Wales]	2015	invasive (unspecified/other)	Neisseria meningitidis	Y	Y	Y	25	1	1	26	4	10	5	18	9	11	9	17	ST-23 complex	23	4	4	2
37796	M15 240009	UK [England]	2015	invasive (unspecified/other)	Neisseria meningitidis	Y		Y	25	1	1	26	4	10	5	18	9	11	9	17	ST-23 complex	23	4	4	2
37797	M15 240010	UK [England]	2015	invasive (unspecified/other)	Neisseria meningitidis	W		W	22	1	1	1	4	2	3	4	3	8	4	6	ST-11 complex	11	4	4	2
37798	M15 240011	UK [England]	2015	invasive (unspecified/other)	Neisseria meningitidis	W		W	22	1	1	1	4	2	3	4	3	8	4	6	ST-11 complex	11	4	4	2
37799	M15 240012	UK [England]	2015	invasive (unspecified/other)	Neisseria meningitidis	W		W	22	1	1	1	4	2	3	4	3	8	4	6	ST-11 complex	11	4	4	2
37800	M15 240014	UK [England]	2015	invasive (unspecified/other)	Neisseria meningitidis	B	B	B	13	3	57	145	3	4	10	34	5	38	11	9	ST-269 complex	1161	3	3	1
37801	M15 240015	UK [England]	2015	invasive (unspecified/other)	Neisseria meningitidis	W	W	W	22	1	1	1	4	2	3	4	3	8	4	6	ST-11 complex	11	4	4	2
37802	M15 240016	UK [England]	2015	invasive (unspecified/other)	Neisseria meningitidis	B	B	B	4	NA	1	84	NA	3	6	9	5	8	6	9	ST-41/44 complex	485	NA	NA	1
37803	M15 240017	UK [England]	2015	invasive (unspecified/other)	Neisseria meningitidis	B	B	B	4	NA	1	84	NA	3	6	9	5	8	6	9	ST-41/44 complex	485	NA	NA	1
37804	M15 240020	UK [England]	2015	invasive (unspecified/other)	Neisseria meningitidis	B	B	B	1	6	7	100	7	NA	10	5	4	6	3	8		NA	7	1	1
37805	M15 240021	UK [England]	2015	invasive (unspecified/other)	Neisseria meningitidis	W		W	13	1	1	468	4	2	3	4	3	8	4	6	ST-11 complex	11	4	3	1
37806	M15 240022	UK [England]	2015	invasive (unspecified/other)	Neisseria meningitidis	W	W	W	22	1	1	1	4	2	3	4	3	8	4	6	ST-11 complex	11	4	4	2
37807	M15 240024	UK [England]	2015	invasive (unspecified/other)	Neisseria meningitidis	B	B	B	21	1	1	7	4	1	5	13	53	26	41	3	ST-162 complex	162	4	4	2
37808	M15 240025	UK [England]	2015	invasive (unspecified/other)	Neisseria meningitidis	Y		Y	24	1	1	27	4	2	7	6	17	9	6	12	ST-167 complex	11474	4	4	2
37809	M15 240027	UK [England]	2015	invasive (unspecified/other)	Neisseria meningitidis	W		W	22	1	1	1	4	2	3	4	3	8	4	6	ST-11 complex	11	4	4	2
37810	M15 240028	UK [England]	2015	invasive (unspecified/other)	Neisseria meningitidis	Y	Y	Y	19	1	1	93	13	2	16	6	17	9	18	8	ST-167 complex	168	13	4	2
37811	M15 240029	UK [England]	2015	invasive (unspecified/other)	Neisseria meningitidis	W		W	22	1	1	1	4	2	3	4	3	8	4	6	ST-11 complex	11	4	4	2
37812	M15 240030	UK [England]	2015	invasive (unspecified/other)	Neisseria meningitidis	Y		Y	25	1	1	26	4	12	5	18	9	11	9	17	ST-23 complex	1655	4	4	2
37813	M15 240031	UK [England]	2015	invasive (unspecified/other)	Neisseria meningitidis	Y	Y	Y	24	1	1	27	4	2	7	6	17	9	6	8	ST-167 complex	1624	4	4	2
38128	M15 240032	UK [England]	2015	invasive (unspecified/other)	Neisseria meningitidis	B	B	B	19	1	1	65	4	9	6	9	9	9	64	9	ST-41/44 complex	409	4	4	2
38129	M15 240033	UK [England]	2015	invasive (unspecified/other)	Neisseria meningitidis	Y		Y	21	1	1	7	4	6	5	173	13	5	24	17	ST-174 complex	1466	4	4	2
37814	M15 240034	UK [England]	2015	invasive (unspecified/other)	Neisseria meningitidis	B	B	B	4	2	1	84	2	3	6	9	5	8	6	9	ST-41/44 complex	485	2	3	1
37815	M15 240035	UK [England]	2015	invasive (unspecified/other)	Neisseria meningitidis	C	C	C	22	1	1	1	4	2	3	4	3	8	4	6	ST-11 complex	11	4	4	2
37816	M15 240036	UK [England]	2015	invasive (unspecified/other)	Neisseria meningitidis	C	C	C	13	5	1	251	11	2	3	4	3	8	4	6	ST-11 complex	11	11	2	1
37817	M15 240037	UK [England]	2015	invasive (unspecified/other)	Neisseria meningitidis	B	B	B	890	5	1	515	23	7	4	10	26	10	18	12		11475	NA	NA	1
37818	M15 240038	UK [England]	2015	invasive (unspecified/other)	Neisseria meningitidis	B	B	B	650	60	77	390	152	7	5	1	13	36	53	15	ST-213 complex	213	NA	NA	1
37819	M15 240039	UK [England]	2015	invasive (unspecified/other)	Neisseria meningitidis	B	B	B	143	4	2	99	1	4	10	15	9	14	11	17	ST-269 complex	1774	1	1	3
37820	M15 240040	UK [England]	2015	invasive (unspecified/other)	Neisseria meningitidis	Y	Y	Y	25	1	1	26	4	12	5	18	9	11	9	17	ST-23 complex	1655	4	4	2
37821	M15 240041	UK [England]	2015	invasive (unspecified/other)	Neisseria meningitidis	W		W	22	1	1	1	4	2	3	4	3	8	4	6	ST-11 complex	11	4	4	2
37822	M15 240042	UK [England]	2015	invasive (unspecified/other)	Neisseria meningitidis	W		W	22	1	1	1	4	2	3	4	3	8	4	6	ST-11 complex	11	4	4	2
37823	M15 240043	UK [England]	2015	invasive (unspecified/other)	Neisseria meningitidis	B	B	B	1	6	3	100	7	8	35	5	4	5	3	8	ST-32 complex	11302	7	1	1
37824	M15 240044	UK [England]	2015	invasive (unspecified/other)	Neisseria meningitidis	B	B	B	45	67	77	87	39	7	5	640	13	36	53	15	ST-213 complex	9176	NA	NA	3
37825	M15 240045	UK [England]	2015	invasive (unspecified/other)	Neisseria meningitidis	Y	Y	Y	25	1	1	26	4	12	5	18	9	11	NA	17		NA	4	4	2
37826	M15 240046	UK [England]	2015	invasive (unspecified/other)	Neisseria meningitidis	B	B	B	226	66	5	228	38	7	5	1	13	36	53	195	ST-213 complex	2388	NA	NA	1
37827	M15 240047	UK [England]	2015	invasive (unspecified/other)	Neisseria meningitidis	Y		Y	14	1	1	142	13	10	5	18	9	11	9	17	ST-23 complex	23	13	3	1
37828	M15 240048	UK [England]	2015	invasive (unspecified/other)	Neisseria meningitidis	W	W	W	22	1	1	1	4	2	3	4	3	8	4	6	ST-11 complex	11	4	4	2
37829	M15 240049	UK [Northern Ireland]	2015	invasive (unspecified/other)	Neisseria meningitidis	B	B	B	19	1	1	65	4	4	10	15	9	759	6	9	ST-269 complex	11870	4	4	2
37830	M15 240050	UK [England]	2015	invasive (unspecified/other)	Neisseria meningitidis	B	B	B	30	17	1	54	37	4	10	2	5	3	11	9	ST-269 complex	1163	NA	NA	3
37831	M15 240051	UK [England]	2015	invasive (unspecified/other)	Neisseria meningitidis	B	B	B	249	84	1	517	121	1	5	13	53	26	41	3	ST-162 complex	162	NA	NA	1
37832	M15 240053	UK [England]	2015	invasive (unspecified/other)	Neisseria meningitidis	B	B	B	25	1	1	26	4	8	4	6	17	5	18	2	ST-103 complex	103	4	4	2
37833	M15 240054	UK [England]	2015	invasive (unspecified/other)	Neisseria meningitidis	Y		Y	25	1	1	26	4	12	5	18	9	11	9	17	ST-23 complex	1655	4	4	2
37834	M15 240055	UK [England]	2015	invasive (unspecified/other)	Neisseria meningitidis	W	W	W	22	1	1	1	4	2	3	4	3	8	4	6	ST-11 complex	11	4	4	2
37835	M15 240056	UK [England]	2015	invasive (unspecified/other)	Neisseria meningitidis	B	B	B	19	1	1	83	4	4	10	2	5	3	11	9	ST-269 complex	1163	4	4	2
37836	M15 240057	UK [England]	2015	invasive (unspecified/other)	Neisseria meningitidis	Y	Y	Y	25	1	1	26	4	12	5	18	9	11	9	17	ST-23 complex	1655	4	4	2
37837	M15 240058	UK [Wales]	2015	invasive (unspecified/other)	Neisseria meningitidis	B	B	B	13	3	57	145	3	4	10	34	5	38	11	9	ST-269 complex	1161	3	3	1
37838	M15 240059	UK [England]	2015	invasive (unspecified/other)	Neisseria meningitidis	Y		Y	25	1	1	26	4	12	5	18	9	11	9	17	ST-23 complex	1655	4	4	2
37839	M15 240060	UK [England]	2015	invasive (unspecified/other)	Neisseria meningitidis	B	B	B	13	3	10	145	3	2	5	19	135	58	25	2		11476	3	3	1
37840	M15 240061	UK [England]	2015	invasive (unspecified/other)	Neisseria meningitidis	W	W	W	16	1	1	60	4	8	5	18	8	11	4	21	ST-22 complex	3849	4	4	2
37841	M15 240062	UK [England]	2015	invasive (unspecified/other)	Neisseria meningitidis	Y		Y	25	1	1	26	4	10	5	18	9	11	9	17	ST-23 complex	23	4	4	2
37842	M15 240063	UK [England]	2015	invasive (unspecified/other)	Neisseria meningitidis	B	B	B	14	7	1	142	6	3	6	9	5	9	6	9	ST-41/44 complex	41	6	2	1
37843	M15 240064	UK [England]	2015	invasive (unspecified/other)	Neisseria meningitidis	Y	Y	Y	25	1	1	26	4	12	5	18	9	11	9	17	ST-23 complex	1655	4	4	2
37844	M15 240065	UK [England]	2015	invasive (unspecified/other)	Neisseria meningitidis	B	B	B	21	1	1	7	4	4	10	5	26	6	3	8	ST-32 complex	1096	4	4	2
37845	M15 240066	UK [Wales]	2015	invasive (unspecified/other)	Neisseria meningitidis	W		W	22	1	1	1	4	2	3	4	3	8	4	6	ST-11 complex	11	4	4	2
37846	M15 240067	UK [England]	2015	invasive (unspecified/other)	Neisseria meningitidis	B	B	B	4	2	1	84	2	7	5	1	13	36	53	15	ST-213 complex	213	2	3	1
37847	M15 240068	UK [England]	2015	invasive (unspecified/other)	Neisseria meningitidis	Y	Y	Y	25	1	1	26	4	10	5	18	9	11	9	17	ST-23 complex	23	4	4	2
37848	M15 240069	UK [England]	2015	invasive (unspecified/other)	Neisseria meningitidis	B	NG	discrepancy	13	3	1	145	3	4	10	5	26	6	3	8	ST-32 complex	1096	3	3	1
37849	M15 240071	UK [England]	2015	invasive (unspecified/other)	Neisseria meningitidis	B	B	B	13	3	1	518	3	3	6	19	5	38	6	9	ST-41/44 complex	11477	3	3	1
37850	M15 240072	UK [England]	2015	invasive (unspecified/other)	Neisseria meningitidis	Y		Y	25	1	1	26	4	10	5	18	9	11	9	17	ST-23 complex	23	4	4	2
37851	M15 240073	UK [England]	2015	invasive (unspecified/other)	Neisseria meningitidis	Y		Y	24	1	1	27	4	2	7	6	17	9	6	8	ST-167 complex	1624	4	4	2
37852	M15 240074	UK [Wales]	2015	invasive (unspecified/other)	Neisseria meningitidis	B	B	B	4	NA	1	84	NA	3	6	34	5	11	6	9	ST-41/44 complex	1194	NA	NA	1
38041	M15 240075	UK [England]	2015	invasive (unspecified/other)	Neisseria meningitidis	B	B	B	4	2	1	84	2	3	6	9	5	9	6	9	ST-41/44 complex	41	2	3	1
38042	M15 240076	UK [Northern Ireland]	2015	invasive (unspecified/other)	Neisseria meningitidis	B	B	B	727	4	2	161	1	4	10	2	5	3	11	9	ST-269 complex	1163	1	1	2
37853	M15 240077	UK [England]	2015	invasive (unspecified/other)	Neisseria meningitidis	Y		Y	25	1	1	26	4	10	5	18	9	11	9	17	ST-23 complex	23	4	4	2
37854	M15 240078	UK [England]	2015	invasive (unspecified/other)	Neisseria meningitidis	W	W	W	22	1	1	1	4	2	3	4	3	8	4	6	ST-11 complex	11	4	4	2
37855	M15 240079	UK [England]	2015	invasive (unspecified/other)	Neisseria meningitidis	W	W	W	22	1	1	1	4	NA	3	4	3	8	4	6		NA	4	4	2
37856	M15 240080	UK [England]	2015	invasive (unspecified/other)	Neisseria meningitidis	W	NG	discrepancy	22	1	1	1	4	2	3	4	3	8	4	6	ST-11 complex	11	4	4	2
37857	M15 240081	UK [England]	2015	invasive (unspecified/other)	Neisseria meningitidis	W		W	22	1	1	1	4	2	3	4	3	8	4	6	ST-11 complex	11	4	4	2
37858	M15 240082	UK [England]	2015	invasive (unspecified/other)	Neisseria meningitidis	Y		Y	25	1	1	26	4	12	5	18	9	11	9	17	ST-23 complex	1655	4	4	2
37859	M15 240083	UK [England]	2015	invasive (unspecified/other)	Neisseria meningitidis	Y		Y	25	1	1	26	4	12	5	18	9	11	9	17	ST-23 complex	1655	4	4	2
38043	M15 240084	UK [England]	2015	invasive (unspecified/other)	Neisseria meningitidis	B	B	B	13	3	57	145	3	4	10	34	5	38	11	9	ST-269 complex	1161	3	3	1
37860	M15 240085	UK [England]	2015	invasive (unspecified/other)	Neisseria meningitidis	W		W	22	1	1	1	4	2	3	4	3	8	4	6	ST-11 complex	11	4	4	2
38044	M15 240087	UK [England]	2015	invasive (unspecified/other)	Neisseria meningitidis	B	B	B	4	2	1	84	2	3	6	9	5	8	6	9	ST-41/44 complex	485	2	3	1
38045	M15 240088	UK [England]	2015	invasive (unspecified/other)	Neisseria meningitidis	B	B	B	15	4	2	193	1	4	10	15	9	8	11	9	ST-269 complex	269	1	1	1
37861	M15 240089	UK [England]	2015	invasive (unspecified/other)	Neisseria meningitidis	W		W	22	1	1	1	4	2	3	4	3	8	4	6	ST-11 complex	11	4	4	2
38046	M15 240090	UK [England]	2015	invasive (unspecified/other)	Neisseria meningitidis	B	B	B	13	3	57	145	3	4	10	34	5	38	11	9	ST-269 complex	1161	3	3	1
37862	M15 240091	UK [England]	2015	invasive (unspecified/other)	Neisseria meningitidis	W		W	256	15	5	207	58	2	3	4	3	8	4	6	ST-11 complex	11	NA	NA	1
38047	M15 240092	UK [England]	2015	invasive (unspecified/other)	Neisseria meningitidis	B	B	B	45	8	77	87	20	7	5	1	13	36	72	15	ST-213 complex	11488	20	4	3
37863	M15 240094	UK [England]	2015	invasive (unspecified/other)	Neisseria meningitidis	Y		Y	21	1	1	7	4	6	5	173	13	5	24	17	ST-174 complex	1466	4	4	2
37864	M15 240095	UK [Wales]	2015	invasive (unspecified/other)	Neisseria meningitidis	W	W	W	16	1	1	60	4	11	5	18	8	11	4	21	ST-22 complex	184	4	4	2
37865	M15 240096	UK [England]	2015	invasive (unspecified/other)	Neisseria meningitidis	B	NG	discrepancy	4	NA	1	84	NA	3	6	9	5	9	6	9	ST-41/44 complex	41	NA	NA	1
37866	M15 240097	UK [Wales]	2015	invasive (unspecified/other)	Neisseria meningitidis	W		W	22	1	1	1	4	2	3	4	3	8	4	6	ST-11 complex	11	4	4	2
37867	M15 240098	UK [England]	2015	invasive (unspecified/other)	Neisseria meningitidis	Y		Y	25	1	1	26	4	10	5	18	9	11	9	17	ST-23 complex	23	4	4	2
37868	M15 240099	UK [England]	2015	invasive (unspecified/other)	Neisseria meningitidis	W		W	22	1	1	1	4	2	3	4	3	8	4	6	ST-11 complex	11	4	4	2
37869	M15 240100	UK [England]	2015	invasive (unspecified/other)	Neisseria meningitidis	Y		Y	25	1	1	26	4	12	5	18	9	11	9	17	ST-23 complex	1655	4	4	2
37870	M15 240101	UK [England]	2015	invasive (unspecified/other)	Neisseria meningitidis	W		W	22	1	1	1	4	2	3	4	3	8	4	6	ST-11 complex	11	4	4	2
37871	M15 240102	UK [Northern Ireland]	2015	invasive (unspecified/other)	Neisseria meningitidis	B	B	B	15	4	2	193	1	4	10	15	9	8	11	9	ST-269 complex	269	1	1	1
37872	M15 240103	UK [England]	2015	invasive (unspecified/other)	Neisseria meningitidis	B	B	B	47	1	57	90	15	12	5	12	35	192	22	17	ST-461 complex	1946	15	4	3
37873	M15 240105	UK [England]	2015	invasive (unspecified/other)	Neisseria meningitidis	W		W	22	1	1	1	4	2	3	4	3	8	4	6	ST-11 complex	11	4	4	2
37874	M15 240106	UK [England]	2015	invasive (unspecified/other)	Neisseria meningitidis	B	B	B	4	2	1	84	2	3	6	9	5	9	6	9	ST-41/44 complex	41	2	3	1
37875	M15 240107	UK [England]	2015	invasive (unspecified/other)	Neisseria meningitidis	W		W	22	1	1	1	4	2	3	4	3	8	4	6	ST-11 complex	11	4	4	2
37876	M15 240109	UK [England]	2015	invasive (unspecified/other)	Neisseria meningitidis	B	B	B	15	10	2	193	17	4	10	15	9	8	11	9	ST-269 complex	269	NA	NA	1
37877	M15 240110	UK [England]	2015	invasive (unspecified/other)	Neisseria meningitidis	B	B	B	21	1	1	7	4	1	5	13	19	26	41	3	ST-162 complex	11478	4	4	2
37878	M15 240111	UK [England]	2015	invasive (unspecified/other)	Neisseria meningitidis	B	B	B	256	15	5	207	58	7	5	1	13	36	20	15	ST-213 complex	11479	NA	NA	1
37879	M15 240112	UK [England]	2015	invasive (unspecified/other)	Neisseria meningitidis	W	W	W	22	1	1	1	4	2	3	4	3	8	4	6	ST-11 complex	11	4	4	2
37880	M15 240113	UK [England]	2015	invasive (unspecified/other)	Neisseria meningitidis	B	B	B	1	6	3	100	7	8	10	5	4	6	583	8	ST-32 complex	8956	7	1	1
37881	M15 240114	UK [England]	2015	invasive (unspecified/other)	Neisseria meningitidis	W	W	W	22	1	1	1	4	2	3	4	3	8	4	6	ST-11 complex	11	4	4	2
37882	M15 240115	UK [England]	2015	invasive (unspecified/other)	Neisseria meningitidis	W		W	22	1	1	1	4	2	3	4	3	8	4	6	ST-11 complex	11	4	4	2
37883	M15 240116	UK [Wales]	2015	invasive (unspecified/other)	Neisseria meningitidis	W		W	22	1	1	1	4	2	3	4	3	8	4	6	ST-11 complex	11	4	4	2
37884	M15 240118	UK [England]	2015	invasive (unspecified/other)	Neisseria meningitidis	B	B	B	4	NA	6	84	NA	3	6	9	5	9	6	9	ST-41/44 complex	41	NA	NA	1
37885	M15 240119	UK [England]	2015	invasive (unspecified/other)	Neisseria meningitidis	Y	Y	Y	25	1	1	26	4	12	5	18	9	11	9	17	ST-23 complex	1655	4	4	2
37886	M15 240120	UK [England]	2015	invasive (unspecified/other)	Neisseria meningitidis	B	B	B	45	61	77	87	153	7	5	1	13	9	53	9	ST-213 complex	2100	NA	NA	3
37887	M15 240121	UK [England]	2015	invasive (unspecified/other)	Neisseria meningitidis	B	B	B	141	52	4	519	109	8	5	19	17	11	24	54		1052	NA	NA	3
37888	M15 240122	UK [England]	2015	invasive (unspecified/other)	Neisseria meningitidis	B	B	B	15	10	2	193	17	4	10	15	9	8	11	9	ST-269 complex	269	NA	NA	1
37889	M15 240126	UK [England]	2015	invasive (unspecified/other)	Neisseria meningitidis	Y		Y	25	1	1	26	4	12	5	18	9	11	9	17	ST-23 complex	1655	4	4	2
37890	M15 240127	UK [England]	2015	invasive (unspecified/other)	Neisseria meningitidis	W		W	22	1	1	1	4	2	3	4	3	8	4	6	ST-11 complex	11	4	4	2
37891	M15 240128	UK [England]	2015	invasive (unspecified/other)	Neisseria meningitidis	W		W	22	1	1	1	4	2	3	4	3	8	4	6	ST-11 complex	11	4	4	2
37892	M15 240129	UK [England]	2015	invasive (unspecified/other)	Neisseria meningitidis	NG	NG	NG	25	1	1	26	4	10	5	18	9	11	9	17	ST-23 complex	23	4	4	2
37893	M15 240130	UK [England]	2015	invasive (unspecified/other)	Neisseria meningitidis	W		W	22	1	1	1	4	2	3	4	3	8	4	6	ST-11 complex	11	4	4	2
37894	M15 240131	UK [England]	2015	invasive (unspecified/other)	Neisseria meningitidis	C	C	C	13	5	1	251	11	2	3	4	3	8	4	6	ST-11 complex	11	11	2	1
37895	M15 240132	UK [England]	2015	invasive (unspecified/other)	Neisseria meningitidis	Y		Y	25	1	1	26	4	12	5	18	9	11	9	17	ST-23 complex	1655	4	4	2
37896	M15 240133	UK [England]	2015	invasive (unspecified/other)	Neisseria meningitidis	B	B	B	13	3	1	145	3	17	5	19	17	3	26	2	ST-60 complex	60	3	3	1
37897	M15 240134	UK [England]	2015	invasive (unspecified/other)	Neisseria meningitidis	Y		Y	25	1	1	26	4	12	5	18	9	11	9	17	ST-23 complex	1655	4	4	2
37898	M15 240135	UK [England]	2015	invasive (unspecified/other)	Neisseria meningitidis	B	B	B	24	1	63	25	4	16	6	9	3	9	6	16	ST-41/44 complex	6135	4	4	2
37899	M15 240136	UK [England]	2015	invasive (unspecified/other)	Neisseria meningitidis	Y	Y	Y	25	1	1	26	4	12	5	18	9	11	9	17	ST-23 complex	1655	4	4	2
37900	M15 240139	UK [England]	2015	invasive (unspecified/other)	Neisseria meningitidis	B	B	B	456	1	1	243	142	8	5	2	17	9	6	21		863	NA	NA	1
37901	M15 240140	UK [England]	2015	invasive (unspecified/other)	Neisseria meningitidis	B	B	B	45	14	77	87	45	7	5	1	13	36	53	15	ST-213 complex	213	NA	NA	3
37902	M15 240141	UK [England]	2015	invasive (unspecified/other)	Neisseria meningitidis	W		W	22	1	1	1	4	662	3	4	3	8	4	6	ST-11 complex	10651	4	4	2

37903	M15 240142	UK [England]	2015	invasive (unspecified/other)	Neisseria meningitidis	B	B	B	1	6	3	100	7	8	10	77	4	6	3	8	ST-32 complex	749	7	1	1
37904	M15 240144	UK [England]	2015	invasive (unspecified/other)	Neisseria meningitidis	W		W	22	1	1	1	4	2	3	4	3	8	4	6	ST-11 complex	11	4	4	2
37905	M15 240145	UK [England]	2015	invasive (unspecified/other)	Neisseria meningitidis	B	B	B	45	33	77	87	14	7	5	1	13	36	53	15	ST-213 complex	213	NA	NA	3
37906	M15 240147	UK [England]	2015	invasive (unspecified/other)	Neisseria meningitidis	B	B	B	14	1	1	142	13	3	5	656	5	3	6	9	ST-41/44 complex	9352	13	3	1
37907	M15 240148	UK [England]	2015	invasive (unspecified/other)	Neisseria meningitidis	W		W	22	1	1	1	4	2	3	4	3	8	4	6	ST-11 complex	11	4	4	2
37908	M15 240149	UK [England]	2015	invasive (unspecified/other)	Neisseria meningitidis	B	B	B	19	1	1	83	4	NA	10	2	5	3	11	9		NA	4	4	2
37909	M15 240150	UK [England]	2015	invasive (unspecified/other)	Neisseria meningitidis	B	B	B	45	8	77	87	20	210	5	1	13	36	53	15	ST-213 complex	3282	20	4	3
37910	M15 240151	UK [England]	2015	invasive (unspecified/other)	Neisseria meningitidis	W		W	22	1	1	1	4	2	3	4	3	8	4	6	ST-11 complex	11	4	4	2
37911	M15 240152	UK [England]	2015	invasive (unspecified/other)	Neisseria meningitidis	Y		Y	25	1	1	26	4	12	5	18	9	11	9	17	ST-23 complex	1655	4	4	2
37912	M15 240154	UK [England]	2015	invasive (unspecified/other)	Neisseria meningitidis	B	B	B	220	92	1	449	139	3	10	15	17	6	10	12	ST-35 complex	1764	NA	NA	1
37913	M15 240155	UK [England]	2015	invasive (unspecified/other)	Neisseria meningitidis	B	B	B	4	2	1	84	2	3	6	9	5	9	6	9	ST-41/44 complex	41	2	3	1
37914	M15 240157	UK [England]	2015	invasive (unspecified/other)	Neisseria meningitidis	W		W	25	1	1	26	4	12	5	18	9	11	9	17	ST-23 complex	1655	4	4	2
37915	M15 240158	UK [England]	2015	invasive (unspecified/other)	Neisseria meningitidis	W	W	W	22	1	1	1	4	2	3	4	3	8	4	6	ST-11 complex	11	4	4	2
37916	M15 240159	UK [England]	2015	invasive (unspecified/other)	Neisseria meningitidis	B	B	B	4	2	1	84	2	3	6	9	32	9	6	9	ST-41/44 complex	4971	2	3	1
37917	M15 240160	UK [England]	2015	invasive (unspecified/other)	Neisseria meningitidis	Y		Y	25	1	1	26	4	12	5	18	9	11	9	17	ST-23 complex	1655	4	4	2
37918	M15 240161	UK [Northern Ireland]	2015	invasive (unspecified/other)	Neisseria meningitidis	C	C	C	674	5	1	375	11	2	3	4	3	8	4	6	ST-11 complex	11	11	2	2
37919	M15 240168	UK [England]	2015	invasive (unspecified/other)	Neisseria meningitidis	B	B	B	215	5	121	233	60	4	5	2	423	21	11	20		11480	NA	NA	1
37920	M15 240169	UK [England]	2015	invasive (unspecified/other)	Neisseria meningitidis	B	B	B	30	17	2	54	37	2	10	15	9	8	11	17	ST-269 complex	1214	NA	NA	3
37921	M15 240171	UK [England]	2015	invasive (unspecified/other)	Neisseria meningitidis	B	B	B	14	5	1	204	11	3	6	19	5	3	6	9	ST-41/44 complex	340	11	2	1
37922	M15 240172	UK [England]	2015	invasive (unspecified/other)	Neisseria meningitidis	W		W	22	1	1	1	4	662	3	4	3	8	4	6	ST-11 complex	10651	4	4	2
37923	M15 240174	UK [England]	2015	invasive (unspecified/other)	Neisseria meningitidis	Y		Y	25	1	1	26	4	12	5	18	9	11	9	17	ST-23 complex	1655	4	4	2
37924	M15 240175	UK [England]	2015	invasive (unspecified/other)	Neisseria meningitidis	B	B	B	13	3	57	145	3	4	10	2	5	38	11	9	ST-269 complex	275	3	3	1
37925	M15 240176	UK [England]	2015	invasive (unspecified/other)	Neisseria meningitidis	B	B	B	4	2	1	84	2	3	6	9	5	9	6	9	ST-41/44 complex	41	2	3	1
37926	M15 240177	UK [England]	2015	invasive (unspecified/other)	Neisseria meningitidis	Y	Y	Y	25	1	1	26	4	12	5	18	9	11	25	17	ST-23 complex	10458	4	4	2
37927	M15 240178	UK [England]	2015	invasive (unspecified/other)	Neisseria meningitidis	B	B	B	24	1	4	27	4	4	10	1	9	3	10	12	ST-35 complex	11481	4	4	2
37928	M15 240179	UK [Northern Ireland]	2015	invasive (unspecified/other)	Neisseria meningitidis	B	B	B	15	10	2	193	17	4	10	15	9	17	11	9	ST-269 complex	2693	NA	NA	1
37929	M15 240180	UK [England]	2015	invasive (unspecified/other)	Neisseria meningitidis	B	B	B	700	7	1	279	6	1	5	13	53	26	41	3	ST-162 complex	162	6	2	1
37930	M15 240181	UK [England]	2015	invasive (unspecified/other)	Neisseria meningitidis	W		W	22	1	1	1	4	2	3	4	3	8	4	6	ST-11 complex	11	4	4	2
37931	M15 240182	UK [England]	2015	invasive (unspecified/other)	Neisseria meningitidis	B	B	B	15	4	2	193	1	4	10	15	9	8	11	13	ST-269 complex	467	1	1	1
37932	M15 240183	UK [England]	2015	invasive (unspecified/other)	Neisseria meningitidis	B	B	B	14	1	1	142	13	3	6	19	5	3	6	9	ST-41/44 complex	340	13	3	1
37933	M15 240185	UK [England]	2015	invasive (unspecified/other)	Neisseria meningitidis	W		W	22	1	1	1	4	2	3	4	3	8	4	6	ST-11 complex	11	4	4	2
37934	M15 240187	UK [Wales]	2015	invasive (unspecified/other)	Neisseria meningitidis	W		W	22	1	1	1	4	2	3	4	3	8	4	6	ST-11 complex	11	4	4	2
37935	M15 240188	UK [Wales]	2015	invasive (unspecified/other)	Neisseria meningitidis	B	B	B	30	17	1	54	37	7	5	1	13	36	53	15	ST-213 complex	213	NA	NA	3
37936	M15 240189	UK [England]	2015	invasive (unspecified/other)	Neisseria meningitidis	W		W	22	1	1	1	4	2	3	4	3	8	4	6	ST-11 complex	11	4	4	2
37937	M15 240190	UK [England]	2015	invasive (unspecified/other)	Neisseria meningitidis	Y		Y	22	1	1	1	4	2	3	4	3	8	4	6	ST-11 complex	11	4	4	2
37938	M15 240191	UK [England]	2015	invasive (unspecified/other)	Neisseria meningitidis	Y		Y	25	1	1	26	4	10	5	18	9	11	9	17	ST-23 complex	23	4	4	2
37939	M15 240192	UK [England]	2015	invasive (unspecified/other)	Neisseria meningitidis	Y	Y	Y	25	1	1	26	4	12	5	18	9	11	9	17	ST-23 complex	1655	4	4	2
37940	M15 240193	UK [England]	2015	invasive (unspecified/other)	Neisseria meningitidis	W	W	W	22	1	1	1	4	2	3	4	3	8	4	6	ST-11 complex	11	4	4	2
37941	M15 240194	UK [England]	2015	invasive (unspecified/other)	Neisseria meningitidis	Y		Y	25	1	1	26	4	12	5	18	9	11	9	17	ST-23 complex	1655	4	4	2
37942	M15 240195	UK [England]	2015	invasive (unspecified/other)	Neisseria meningitidis	W		W	22	1	1	1	4	2	3	4	3	8	4	6	ST-11 complex	11	4	4	2
37943	M15 240196	UK [England]	2015	invasive (unspecified/other)	Neisseria meningitidis	B	B	B	15	4	2	193	1	4	10	15	9	8	11	9	ST-269 complex	269	1	1	1
37944	M15 240199	UK [England]	2015	invasive (unspecified/other)	Neisseria meningitidis	Y		Y	25	1	1	26	4	12	5	18	9	11	9	17	ST-23 complex	1655	4	4	2
37945	M15 240201	UK [England]	2015	invasive (unspecified/other)	Neisseria meningitidis	B	B	B	4	NA	1	84	NA	3	6	9	5	11	6	9	ST-41/44 complex	154	NA	NA	1
37946	M15 240202	UK [England]	2015	invasive (unspecified/other)	Neisseria meningitidis	NG	Z	Z	16	56	79	60	NA	13	29	2	17	26	65	53		2123	NA	NA	2
37947	M15 240203	UK [England]	2015	invasive (unspecified/other)	Neisseria meningitidis	B	B	B	101	1	1	13	4	13	3	52	13	3	11	53	ST-364 complex	11482	4	4	2
37948	M15 240204	UK [England]	2015	invasive (unspecified/other)	Neisseria meningitidis	C	C	C	15	4	2	193	1	4	10	15	9	8	11	7	ST-269 complex	11024	1	1	1
37949	M15 240205	UK [England]	2015	invasive (unspecified/other)	Neisseria meningitidis	C	C	C	22	1	1	1	4	2	3	4	3	8	4	6	ST-11 complex	11	4	4	2
37950	M15 240206	UK [England]	2015	invasive (unspecified/other)	Neisseria meningitidis	B	NG	discrepancy	19	1	1	83	4	7	5	1	13	36	53	15	ST-213 complex	213	4	4	2
37951	M15 240207	UK [England]	2015	invasive (unspecified/other)	Neisseria meningitidis	W		W	22	1	1	1	4	2	3	4	3	8	4	6	ST-11 complex	11	4	4	2
37952	M15 240208	UK [England]	2015	invasive (unspecified/other)	Neisseria meningitidis	B	B	B	13	3	57	145	3	4	10	6	5	38	11	9	ST-269 complex	1991	3	3	1
37953	M15 240209	UK [England]	2015	invasive (unspecified/other)	Neisseria meningitidis	B	B	B	4	2	1	84	2	3	6	9	5	8	6	9	ST-41/44 complex	485	2	3	1
37954	M15 240211	UK [Northern Ireland]	2015	invasive (unspecified/other)	Neisseria meningitidis	B	B	B	19	1	1	83	4	4	10	2	5	3	11	9	ST-269 complex	1163	4	4	2
37955	M15 240212	UK [England]	2015	invasive (unspecified/other)	Neisseria meningitidis	Y	Y	Y	25	1	1	26	4	12	5	18	9	11	9	17	ST-23 complex	1655	4	4	2
37956	M15 240213	UK [England]	2015	invasive (unspecified/other)	Neisseria meningitidis	B	B	B	45	94	77	87	154	7	5	1	13	36	53	15	ST-213 complex	213	NA	NA	3
37957	M15 240215	UK [England]	2015	invasive (unspecified/other)	Neisseria meningitidis	B	B	B	354	93	77	41	155	7	5	1	13	36	53	15	ST-213 complex	213	NA	NA	3
37958	M15 240216	UK [England]	2015	invasive (unspecified/other)	Neisseria meningitidis	W	W	W	22	1	1	1	4	2	3	4	3	8	4	6	ST-11 complex	11	4	4	2
37959	M15 240219	UK [England]	2015	invasive (unspecified/other)	Neisseria meningitidis	B	B	B	47	1	57	90	15	12	5	12	35	192	22	17	ST-461 complex	1946	15	4	3
37960	M15 240220	UK [England]	2015	invasive (unspecified/other)	Neisseria meningitidis	B	B	B	4	NA	1	84	NA	3	6	9	5	9	6	9	ST-41/44 complex	41	NA	NA	1
37961	M15 240221	UK [England]	2015	invasive (unspecified/other)	Neisseria meningitidis	W		W	22	1	1	1	4	2	3	4	3	8	4	6	ST-11 complex	11	4	4	2
37962	M15 240222	UK [England]	2015	invasive (unspecified/other)	Neisseria meningitidis	W		W	22	1	1	1	4	2	3	4	3	8	NA	6		NA	4	4	2
37963	M15 240223	UK [England]	2015	invasive (unspecified/other)	Neisseria meningitidis	Y		Y	25	1	1	26	4	10	5	18	9	11	9	17	ST-23 complex	23	4	4	2
37964	M15 240224	UK [England]	2015	invasive (unspecified/other)	Neisseria meningitidis	W	W	W	321	111	1	598	NA	8	6	6	17	8	31	8		9316	NA	NA	1
37965	M15 240225	UK [Northern Ireland]	2015	invasive (unspecified/other)	Neisseria meningitidis	B	B	B	4	2	1	84	2	3	6	49	5	9	6	9	ST-41/44 complex	274	2	3	1
37966	M15 240226	UK [England]	2015	invasive (unspecified/other)	Neisseria meningitidis	B	B	B	16	52	4	59	109	4	10	11	18	6	10	12	ST-35 complex	35	NA	NA	2
37967	M15 240227	UK [England]	2015	invasive (unspecified/other)	Neisseria meningitidis	C	C	C	22	1	1	1	4	2	3	4	3	8	4	6	ST-11 complex	11	4	4	2
37968	M15 240229	UK [England]	2015	invasive (unspecified/other)	Neisseria meningitidis	B	B	B	16	1	4	59	4	4	10	11	18	6	24	12	ST-35 complex	5815	4	4	2
37969	M15 240231	UK [England]	2015	invasive (unspecified/other)	Neisseria meningitidis	B	B	B	59	9	77	75	16	7	5	1	757	36	53	664	ST-213 complex	11871	16	2	3
37970	M15 240232	UK [England]	2015	invasive (unspecified/other)	Neisseria meningitidis	B	B	B	4	2	1	84	2	3	6	9	5	9	6	9	ST-41/44 complex	41	2	3	1
37971	M15 240233	UK [England]	2015	invasive (unspecified/other)	Neisseria meningitidis	B	B	B	1	6	3	100	7	8	10	77	4	6	3	8	ST-32 complex	749	7	1	1
37972	M15 240235	UK [Northern Ireland]	2015	invasive (unspecified/other)	Neisseria meningitidis	B	B	B	13	3	57	145	3	4	10	34	5	38	11	9	ST-269 complex	1161	3	3	1
37973	M15 240236	UK [England]	2015	invasive (unspecified/other)	Neisseria meningitidis	B	B	B	891	1	1	520	4	4	10	11	NA	6	10	12		NA	4	4	NA
38048	M15 240237	UK [England]	2015	invasive (unspecified/other)	Neisseria meningitidis	NG	cnl	cnl	4	1	1	206	4	5	4	17	15	30	7	12	ST-198 complex	823	4	3	1
37974	M15 240239	UK [England]	2015	invasive (unspecified/other)	Neisseria meningitidis	C	C	C	15	4	2	193	1	4	10	15	9	8	11	7	ST-269 complex	11024	1	1	1
37975	M15 240240	UK [England]	2015	invasive (unspecified/other)	Neisseria meningitidis	B	B	B	13	3	57	145	3	4	10	34	5	38	11	9	ST-269 complex	1161	3	3	1
37976	M15 240241	UK [England]	2015	invasive (unspecified/other)	Neisseria meningitidis	B	B	B	15	4	2	193	1	4	10	15	9	8	11	9	ST-269 complex	269	1	1	1
37977	M15 240242	UK [England]	2015	invasive (unspecified/other)	Neisseria meningitidis	W	W	W	22	1	1	1	4	2	3	4	3	8	4	6	ST-11 complex	11	4	4	2
37978	M15 240244	UK [England]	2015	invasive (unspecified/other)	Neisseria meningitidis	W	W	W	22	1	1	1	4	2	3	4	3	8	4	6	ST-11 complex	11	4	4	2
37979	M15 240246	UK [England]	2015	invasive (unspecified/other)	Neisseria meningitidis	Y	Y	Y	25	1	1	26	4	10	5	18	9	11	9	12	ST-23 complex	4183	4	4	2
37980	M15 240247	UK [England]	2015	invasive (unspecified/other)	Neisseria meningitidis	Y		Y	25	1	1	26	4	12	5	18	9	11	9	17	ST-23 complex	1655	4	4	2
37981	M15 240248	UK [England]	2015	invasive (unspecified/other)	Neisseria meningitidis	W		W	456	73	80	243	56	2	3	4	3	8	4	6	ST-11 complex	11	NA	NA	1
37982	M15 240250	UK [England]	2015	invasive (unspecified/other)	Neisseria meningitidis	Y		Y	25	1	1	26	4	12	5	18	9	11	9	17	ST-23 complex	1655	4	4	2
37983	M15 240251	UK [England]	2015	invasive (unspecified/other)	Neisseria meningitidis	W	W	W	22	1	1	1	4	2	3	4	3	8	4	6	ST-11 complex	11	4	4	2
37984	M15 240252	UK [England]	2015	invasive (unspecified/other)	Neisseria meningitidis	B	B	B	16	1	4	59	4	4	10	11	758	36	10	12	ST-35 complex	11872	4	4	2
37985	M15 240253	UK [England]	2015	invasive (unspecified/other)	Neisseria meningitidis	B	B	B	13	3	57	145	3	4	10	34	5	38	11	9	ST-269 complex	1161	3	3	1
37986	M15 240256	UK [Northern Ireland]	2015	invasive (unspecified/other)	Neisseria meningitidis	W	W	W	22	1	1	1	4	2	3	4	3	8	4	6	ST-11 complex	11	4	4	2
37987	M15 240257	UK [England]	2015	invasive (unspecified/other)	Neisseria meningitidis	W		W	22	1	1	1	4	2	3	4	3	8	4	6	ST-11 complex	11	4	4	2
37988	M15 240258	UK [England]	2015	invasive (unspecified/other)	Neisseria meningitidis	W		W	22	1	1	1	4	2	3	4	3	8	4	6	ST-11 complex	11	4	4	2
37989	M15 240259	UK [England]	2015	invasive (unspecified/other)	Neisseria meningitidis	B	B	B	2	5	1	280	11	4	10	15	17	8	11	17	ST-269 complex	479	11	2	1
37990	M15 240260	UK [England]	2015	invasive (unspecified/other)	Neisseria meningitidis	W	W	W	22	1	1	1	4	2	3	4	3	8	4	6	ST-11 complex	11	4	4	2
37991	M15 240262	UK [England]	2015	invasive (unspecified/other)	Neisseria meningitidis	B	B	B	4	2	79	84	2	3	6	9	5	9	6	9	ST-41/44 complex	41	2	3	1
37992	M15 240263	UK [England]	2015	invasive (unspecified/other)	Neisseria meningitidis	W		W	22	1	1	1	4	2	3	4	3	8	4	6	ST-11 complex	11	4	4	2
37993	M15 240265	UK [England]	2015	invasive (unspecified/other)	Neisseria meningitidis	Y	Y	Y	25	1	1	26	4	12	5	18	9	11	9	17	ST-23 complex	1655	4	4	2
37994	M15 240270	UK [England]	2015	invasive (unspecified/other)	Neisseria meningitidis	B	B	B	13	3	57	145	3	12	5	34	17	3	172	124		11483	3	3	1
37995	M15 240271	UK [England]	2015	invasive (unspecified/other)	Neisseria meningitidis	W	W	W	22	1	1	1	4	2	3	4	3	8	4	6	ST-11 complex	11	4	4	2
37996	M15 240273	UK [England]	2015	invasive (unspecified/other)	Neisseria meningitidis	B	B	B	318	36	1	226	35	NA	5	2	423	21	6	20		NA	NA	NA	1
37997	M15 240276	UK [England]	2015	invasive (unspecified/other)	Neisseria meningitidis	B	B	B	499	15	77	134	58	7	5	1	13	36	53	15	ST-213 complex	213	NA	NA	3
37998	M15 240277	UK [England]	2015	invasive (unspecified/other)	Neisseria meningitidis	W	W	W	22	1	1	1	4	2	3	4	3	8	4	6	ST-11 complex	11	4	4	2
37999	M15 240278	UK [England]	2015	invasive (unspecified/other)	Neisseria meningitidis	W		W	22	1	1	1	4	2	3	4	3	8	4	6	ST-11 complex	11	4	4	2
38000	M15 240279	UK [England]	2015	invasive (unspecified/other)	Neisseria meningitidis	B	B	B	1	6	3	100	7	8	10	77	4	6	3	8	ST-32 complex	749	7	1	1
38001	M15 240280	UK [England]	2015	invasive (unspecified/other)	Neisseria meningitidis	W	W	W	22	1	1	1	4	662	3	4	3	8	4	6	ST-11 complex	10651	4	4	2
38002	M15 240281	UK [England]	2015	invasive (unspecified/other)	Neisseria meningitidis	B	B	B	13	3	57	145	3	2	29	514	459	26	481	53		7622	3	3	1
38003	M15 240282	UK [England]	2015	invasive (unspecified/other)	Neisseria meningitidis	W	W	W	22	1	1	1	4	2	3	4	3	8	4	6	ST-11 complex	11	4	4	2
38004	M15 240284	UK [England]	2015	invasive (unspecified/other)	Neisseria meningitidis	W	W	W	22	1	1	1	4	2	3	4	3	8	4	6	ST-11 complex	11	4	4	2
38005	M15 240285	UK [England]	2015	invasive (unspecified/other)	Neisseria meningitidis	Y		Y	25	1	1	26	4	12	5	18	9	11	9	17	ST-23 complex	1655	4	4	2
38006	M15 240286	UK [England]	2015	invasive (unspecified/other)	Neisseria meningitidis	B	B	B	13	3	57	145	3	4	10	34	5	38	11	9	ST-269 complex	1161	3	3	1
38007	M15 240287	UK [England]	2015	invasive (unspecified/other)	Neisseria meningitidis	W		W	22	1	1	1	4	2	3	4	3	8	4	6	ST-11 complex	11	4	4	2
38008	M15 240288	UK [England]	2015	invasive (unspecified/other)	Neisseria meningitidis	W	W	W	892	1	1	521	4	2	3	4	3	8	4	6	ST-11 complex	11	4	4	2
38009	M15 240289	UK [England]	2015	invasive (unspecified/other)	Neisseria meningitidis	B	B	B	14	5	1	204	11	3	6	19	5	3	6	9	ST-41/44 complex	340	11	2	1
38010	M15 240290	UK [England]	2015	invasive (unspecified/other)	Neisseria meningitidis	Y		Y	31	9	1	58	16	12	5	18	9	11	9	17	ST-23 complex	1655	16	2	3
38011	M15 240291	UK [England]	2015	invasive (unspecified/other)	Neisseria meningitidis	W	W	W	22	1	1	1	4	2	3	4	3	8	4	6	ST-11 complex	11	4	4	2
38012	M15 240292	UK [England]	2015	invasive (unspecified/other)	Neisseria meningitidis	Y		Y	25	1	1	26	4	10	5	18	9	11	9	17	ST-23 complex	23	4	4	2
38013	M15 240294	UK [England]	2015	invasive (unspecified/other)	Neisseria meningitidis	Y		Y	25	1	1	26	4	12	5	18	9	11	9	17	ST-23 complex	1655	4	4	2
38014	M15 240295	UK [England]	2015	invasive (unspecified/other)	Neisseria meningitidis	Y		Y	25	1	1	26	4	12	5	18	9	11	9	17	ST-23 complex	1655	4	4	2
38015	M15 240296	UK [England]	2015	invasive (unspecified/other)	Neisseria meningitidis	B	B	B	14	1	57	142	13	6	5	12	12	12	13	14		1345	13	3	1
38016	M15 240297	UK [England]	2015	invasive (unspecified/other)	Neisseria meningitidis	B	B	B	327	1	57	522	4	4	10	29	5	38	11	9	ST-269 complex	11484	4	3	1
38017	M15 240300	UK [England]	2015	invasive (unspecified/other)	Neisseria meningitidis	W		W	22	1	1	1	4	2	3	4	3	8	4	6	ST-11 complex	11	4	4	2
38018	M15 240301	UK [Northern Ireland]	2015	invasive (unspecified/other)	Neisseria meningitidis	W	W	W	22	1	1	1	4	2	3	4	3	8	4	6	ST-11 complex	11	4	4	2
38019	M15 240302	UK [England]	2015	invasive (unspecified/other)	Neisseria meningitidis	B	B	B	125	1	57	268	13	10	5	1	13	8	53	15	ST-213 complex	11485	13	3	1
38020	M15 240304	UK [England]	2015	invasive (unspecified/other)	Neisseria meningitidis	B	B	B	1	6	3	100	7	8	10	77	25	6	3	22		5151	7	1	1
38021	M15 240305	UK [England]	2015	invasive (unspecified/other)	Neisseria meningitidis	Y		Y	25	1	1	26	4	12	5	18	9	11	9	17	ST-23 complex	1655	4	4	2
38022	M15 240307	UK [England]	2015	invasive (unspecified/other)	Neisseria meningitidis	B	B	B	37	1	1	408	4	1	20	10	26	10	593	355		11486	4	3	1
38023	M15 240309	UK [England]	2015	invasive (unspecified/other)	Neisseria meningitidis	B	B	B	4	117	1	84	134	3	6	9	5	21	6	9	ST-41/44 complex	11487	NA	NA	1
38024	M15 240310	UK [England]	2015	invasive (unspecified/other)	Neisseria meningitidis	W		W	22	1	1	1	4	2	3	4	3	8	4	6	ST-11 complex	11	4	4	2
38025	M15 240312	UK [England]	2015	invasive (unspecified/other)	Neisseria meningitidis	B	B	B	19	1	1	65	4	9	6	9	759	9	64	9	ST-41/44 complex	11873	4	4	2
38026	M15 240313	UK [England]	2015	invasive (unspecified/other)	Neisseria meningitidis	B	B	B	14	105	1	142	145	3	6	4	670	3	6	9	ST-41/44 complex	10696	NA	NA	1
38027	M15 240316	UK [England]	2015	invasive (unspecified/other)	Neisseria meningitidis	W	W	W	22	1	1	1	4	2	3	4	3	8	4	6	ST-11 complex	11	4	4	2
38028	M15 240319	UK [England]	2015	invasive (unspecified/other)	Neisseria meningitidis	Y	Y	Y	25	1	1	26	4	12	5	18	9	11	9	17	ST-23 complex	1655	4	4	2
38029	M15 240320	UK [England]	2015	invasive (unspecified/other)	Neisseria meningitidis	W	W	W	22	1	1	1	4	NA	3	4	3	8	4	6		NA	4	4	2
38030	M15 240321	UK [England]	2015	invasive (unspecified/other)	Neisseria meningitidis	B	B	B	110	21	5	171	19	4	10	2	5	563	11	9	ST-269 complex	8616	NA	NA	1
38031	M15 240322	UK [England]	2015	invasive (unspecified/other)	Neisseria meningitidis	B	B	B	4	2	1	84	2	3	6	9	5	8	6	9	ST-41/44 complex	485	2	3	1
38032	M15 240323	UK [England]	2015	invasive (unspecified/other)	Neisseria meningitidis	B	B	B	95	5	1	32	11	NA	10	72	9	6	10	12		NA	11	2	2
38033	M15 240325	UK [England]	2015	invasive (unspecified/other)	Neisseria meningitidis	B	B	B	13	3	57	145	3	4	10	34	5	38	11	9	ST-269 complex	1161	3	3	1
38034	M15 240326	UK [England]	2015	invasive (unspecified/other)	Neisseria meningitidis	W	W	W	22	1	1	1	4	2	3	4	3	8	4	6	ST-11 complex	11	4	4	2
38035	M15 240327	UK [England]	2015	invasive (unspecified/other)	Neisseria meningitidis	W	W	W	22	1	1	1	4	2	3	4	3	8	NA	6		NA	4	4	2
38036	M15 240328	UK [England]	2015	invasive (unspecified/other)	Neisseria meningitidis	B	B	B	14	5	1	204	11	3	6	19	5	17	6	9	ST-41/44 complex	8511	11	2	1
38037	M15 240329	UK [England]	2015	invasive (unspecified/other)	Neisseria meningitidis	W	W	W	22	1	1	1	4	2	3	4	3	8	4	6	ST-11 complex	11	4	4	2
38038	M15 240332	UK [England]	2015	invasive (unspecified/other)	Neisseria meningitidis	W	W	W	22	1	1	1	4	2	3	4	3	8	4	6	ST-11 complex	11	4	4	2
38039	M15 240333	UK [England]	2015	invasive (unspecified/other)	Neisseria meningitidis	B	B	B	4	2	1	84	2	3	6	796	5	9	18	9	ST-41/44 complex	11874	2	3	1

38049	M15 240334	UK [England]	2015	invasive (unspecified/other)	Neisseria meningitidis	W		W	22	1	1	1	4	2	3	4	3	8	4	6	ST-11 complex	11	4	4	2
38050	M15 240335	UK [England]	2015	invasive (unspecified/other)	Neisseria meningitidis	B	B	B	31	9	77	58	16	7	5	9	13	36	239	195	ST-213 complex	11489	16	2	3
38051	M15 240336	UK [England]	2015	invasive (unspecified/other)	Neisseria meningitidis	B	B	B	47	1	1	90	15	12	5	12	35	60	22	17	ST-461 complex	461	15	4	3
38052	M15 240337	UK [England]	2015	invasive (unspecified/other)	Neisseria meningitidis	B	B	B	4	2	1	84	2	3	6	9	5	11	6	9	ST-41/44 complex	154	2	3	1
38053	M15 240352	UK [England]	2015	invasive (unspecified/other)	Neisseria meningitidis	Y		Y	25	1	1	26	4	10	5	18	9	11	774	17	ST-23 complex	11875	4	4	2
38054	M15 240354	UK [England]	2015	invasive (unspecified/other)	Neisseria meningitidis	Y	Y	Y	25	1	1	26	4	12	5	18	NA	11	25	17		NA	4	4	2
38055	M15 240355	UK [England]	2015	invasive (unspecified/other)	Neisseria meningitidis	C	C	C	1	6	7	100	7	4	10	5	4	6	188	8	ST-32 complex	2000	7	1	1
38056	M15 240356	UK [England]	2015	invasive (unspecified/other)	Neisseria meningitidis	B	B	B	14	7	1	142	6	3	6	9	5	9	6	9	ST-41/44 complex	41	6	2	1
38057	M15 240357	UK [England]	2015	invasive (unspecified/other)	Neisseria meningitidis	B	B	B	4	2	1	84	2	3	6	9	5	9	6	NA		NA	2	3	1
38058	M15 240373	UK [England]	2015	invasive (unspecified/other)	Neisseria meningitidis	B	B	B	13	3	57	145	3	4	10	34	5	38	11	9	ST-269 complex	1161	3	3	1
38059	M15 240375	UK [England]	2015	invasive (unspecified/other)	Neisseria meningitidis	W	W	W	22	1	1	1	4	662	3	4	3	8	4	6	ST-11 complex	10651	4	4	2
38060	M15 240376	UK [England]	2015	invasive (unspecified/other)	Neisseria meningitidis	W		W	22	1	1	1	4	2	3	4	3	8	4	6	ST-11 complex	11	4	4	2
38061	M15 240378	UK [England]	2015	invasive (unspecified/other)	Neisseria meningitidis	W		W	16	1	1	60	4	11	5	5	8	11	24	21	ST-22 complex	1617	4	4	2
38062	M15 240379	UK [England]	2015	invasive (unspecified/other)	Neisseria meningitidis	B	B	B	291	20	4	203	8	4	10	47	17	6	2	9		1575	NA	NA	1
38063	M15 240380	UK [England]	2015	invasive (unspecified/other)	Neisseria meningitidis	W	W	W	22	1	1	1	4	2	3	4	3	8	4	6	ST-11 complex	11	4	4	2
38064	M15 240381	UK [England]	2015	invasive (unspecified/other)	Neisseria meningitidis	W		W	22	1	1	1	4	2	3	4	3	8	4	6	ST-11 complex	11	4	4	2
38065	M15 240382	UK [England]	2015	invasive (unspecified/other)	Neisseria meningitidis	W	W	W	22	1	1	1	4	2	3	4	3	8	4	6	ST-11 complex	11	4	4	2
38066	M15 240383	UK [England]	2015	invasive (unspecified/other)	Neisseria meningitidis	W	W	W	22	1	1	1	4	2	3	4	3	8	4	6	ST-11 complex	11	4	4	2
38067	M15 240384	UK [England]	2015	invasive (unspecified/other)	Neisseria meningitidis	W		W	22	1	1	1	4	2	3	4	3	8	4	6	ST-11 complex	11	4	4	2
38068	M15 240385	UK [England]	2015	invasive (unspecified/other)	Neisseria meningitidis	B	B	B	15	4	2	193	1	4	10	15	9	8	25	9	ST-269 complex	3529	1	1	1
38069	M15 240386	UK [England]	2015	invasive (unspecified/other)	Neisseria meningitidis	B	B	B	47	1	57	90	15	12	5	12	35	192	22	17	ST-461 complex	1946	15	4	3
38070	M15 240388	UK [England]	2015	invasive (unspecified/other)	Neisseria meningitidis	B	B	B	47	1	57	90	15	12	5	12	760	192	22	17	ST-461 complex	11876	15	4	3
38071	M15 240422	UK [England]	2015	invasive (unspecified/other)	Neisseria meningitidis	W/Y	W/Y	W/Y	16	1	1	60	4	219	5	275	17	11	8	21		3015	4	4	2
38072	M15 240423	UK [England]	2015	invasive (unspecified/other)	Neisseria meningitidis	B	B	B	536	118	5	523	156	1	5	13	53	26	41	3	ST-162 complex	162	NA	NA	3
38073	M15 240424	UK [England]	2015	invasive (unspecified/other)	Neisseria meningitidis	B	B	B	15	4	2	193	1	4	10	15	9	8	11	9	ST-269 complex	269	1	1	1
38074	M15 240425	UK [England]	2015	invasive (unspecified/other)	Neisseria meningitidis	B	B	B	16	1	4	59	4	8	10	11	4	6	3	8	ST-32 complex	8758	4	4	2
38075	M15 240437	UK [England]	2015	invasive (unspecified/other)	Neisseria meningitidis	B	B	B	13	3	57	145	3	4	10	34	5	38	11	9	ST-269 complex	1161	3	3	1
38076	M15 240438	UK [England]	2015	invasive (unspecified/other)	Neisseria meningitidis	W	W	W	22	1	1	1	4	2	3	4	3	8	4	6	ST-11 complex	11	4	4	2
38077	M15 240439	UK [England]	2015	invasive (unspecified/other)	Neisseria meningitidis	B	B	B	4	2	6	84	2	3	6	9	5	9	6	9	ST-41/44 complex	41	2	3	1
38078	M15 240440	UK [England]	2015	invasive (unspecified/other)	Neisseria meningitidis	Y		Y	25	1	1	26	4	12	5	18	9	11	9	17	ST-23 complex	1655	4	4	2
38079	M15 240441	UK [England]	2015	invasive (unspecified/other)	Neisseria meningitidis	W	W	W	22	1	1	1	4	2	3	4	3	8	4	6	ST-11 complex	11	4	4	2
38080	M15 240443	UK [England]	2015	invasive (unspecified/other)	Neisseria meningitidis	B	B	B	13	3	1	524	3	46	3	79	25	6	22	12		3192	3	3	1
38081	M15 240445	UK [Wales]	2015	invasive (unspecified/other)	Neisseria meningitidis	B	B	B	4	2	79	84	2	3	6	9	5	9	6	9	ST-41/44 complex	41	2	3	1
38082	M15 240446	UK [England]	2015	invasive (unspecified/other)	Neisseria meningitidis	B	B	B	16	1	4	59	4	4	10	11	18	6	10	12	ST-35 complex	35	4	4	2
38083	M15 240459	UK [England]	2015	invasive (unspecified/other)	Neisseria meningitidis	B	B	B	45	8	77	87	20	7	5	1	13	36	53	15	ST-213 complex	213	20	4	3
38084	M15 240460	UK [England]	2015	invasive (unspecified/other)	Neisseria meningitidis	B	B	B	45	8	77	87	20	7	5	1	13	36	53	15	ST-213 complex	213	20	4	3
38085	M15 240461	UK [England]	2015	invasive (unspecified/other)	Neisseria meningitidis	W		W	22	1	1	1	4	2	3	4	3	8	4	6	ST-11 complex	11	4	4	2
38086	M15 240463	UK [England]	2015	invasive (unspecified/other)	Neisseria meningitidis	B	B	B	47	1	57	90	15	12	5	12	35	192	22	17	ST-461 complex	1946	15	4	3
38087	M15 240464	UK [England]	2015	invasive (unspecified/other)	Neisseria meningitidis	Y	Y	Y	25	1	1	26	4	10	25	18	9	11	9	12	ST-23 complex	11490	4	4	2
38088	M15 240465	UK [England]	2015	invasive (unspecified/other)	Neisseria meningitidis	W		W	22	1	1	1	4	2	3	4	3	8	4	6	ST-11 complex	11	4	4	2
38089	M15 240466	UK [England]	2015	invasive (unspecified/other)	Neisseria meningitidis	B	B	B	622	4	2	190	1	4	10	15	9	5	11	17	ST-269 complex	1791	1	1	1
38090	M15 240468	UK [England]	2015	invasive (unspecified/other)	Neisseria meningitidis	B	B	B	13	3	57	145	3	4	10	34	5	38	11	330		4713	3	3	1
38091	M15 240469	UK [England]	2015	invasive (unspecified/other)	Neisseria meningitidis	W		W	16	22	1	97	32	11	5	168	8	11	4	21	ST-22 complex	1286	NA	NA	2
38092	M15 240472	UK [England]	2015	invasive (unspecified/other)	Neisseria meningitidis	B	B	B	19	1	1	65	4	9	6	9	9	3	6	16	ST-41/44 complex	7746	4	4	2
38093	M15 240473	UK [England]	2015	invasive (unspecified/other)	Neisseria meningitidis	Y		Y	25	1	1	26	4	12	5	18	NA	11	9	17		NA	4	4	2
38094	M15 240474	UK [England]	2015	invasive (unspecified/other)	Neisseria meningitidis	B	B	B	14	7	1	257	6	10	6	9	5	11	6	9	ST-41/44 complex	2203	6	2	1
38095	M15 240476	UK [England]	2015	invasive (unspecified/other)	Neisseria meningitidis	B	B	B	13	3	57	145	3	4	10	2	5	38	11	9	ST-269 complex	275	3	3	1
38096	M15 240477	UK [England]	2015	invasive (unspecified/other)	Neisseria meningitidis	W	W	W	22	1	1	1	4	2	3	4	72	8	4	6	ST-11 complex	11491	4	4	2
38097	M15 240496	UK [Wales]	2015	invasive (unspecified/other)	Neisseria meningitidis	B	B	B	893	9	102	911	16	4	10	5	26	6	3	8	ST-32 complex	1096	16	2	1
38098	M15 240497	UK [England]	2015	invasive (unspecified/other)	Neisseria meningitidis	B	B	B	4	2	1	84	2	3	6	9	5	8	6	9	ST-41/44 complex	485	2	3	1
38099	M15 240498	UK [England]	2015	invasive (unspecified/other)	Neisseria meningitidis	Y		Y	25	1	1	26	4	12	5	18	9	11	9	17	ST-23 complex	1655	4	4	2
38100	M15 240499	UK [England]	2015	invasive (unspecified/other)	Neisseria meningitidis	B	B	B	4	2	1	84	2	3	6	9	5	9	6	49	ST-41/44 complex	11227	2	3	1
38101	M15 240511	UK [England]	2015	invasive (unspecified/other)	Neisseria meningitidis	W		W	22	1	1	1	4	2	3	4	3	8	4	6	ST-11 complex	11	4	4	2
38102	M15 240513	UK [England]	2015	invasive (unspecified/other)	Neisseria meningitidis	W	W	W	22	1	1	1	4	2	3	4	3	8	NA	6		NA	4	4	2
38103	M15 240514	UK [England]	2015	invasive (unspecified/other)	Neisseria meningitidis	B	B	B	4	2	1	84	2	3	6	34	5	11	6	9	ST-41/44 complex	1194	2	3	1
38104	M15 240525	UK [England]	2015	invasive (unspecified/other)	Neisseria meningitidis	Y	Y	Y	25	1	1	26	4	12	5	18	9	11	9	17	ST-23 complex	1655	4	4	2
38105	M15 240541	UK [England]	2015	invasive (unspecified/other)	Neisseria meningitidis	B	B	B	13	3	57	145	3	4	10	34	5	38	11	9	ST-269 complex	1161	3	3	1
38148	M15 240541B	UK	2015	invasive (unspecified/other)	Neisseria meningitidis	B	B	B	13	3	57	145	3	4	10	34	5	38	11	9	ST-269 complex	1161	3	3	1
38149	M15 240541C	UK	2015	invasive (unspecified/other)	Neisseria meningitidis	B	B	B	13	3	57	145	3	4	10	34	5	38	11	9	ST-269 complex	1161	3	3	1
38150	M15 240541D	UK	2015	invasive (unspecified/other)	Neisseria meningitidis	B	B	B	13	3	57	145	3	4	10	34	5	38	11	9	ST-269 complex	1161	3	3	1
39002	M15 240552	UK [England]	2015	invasive (unspecified/other)	Neisseria meningitidis	B	B	B	4	2	1	84	2	3	6	9	5	9	6	9	ST-41/44 complex	41	2	3	1
38040	M15 240553	UK [England]	2015	invasive (unspecified/other)	Neisseria meningitidis	C	C	C	1	6	7	100	7	4	10	5	4	6	3	8	ST-32 complex	32	7	1	1
38106	M15 240554	UK [England]	2015	invasive (unspecified/other)	Neisseria meningitidis	W	W	W	22	1	1	1	4	2	3	4	3	8	4	6	ST-11 complex	11	4	4	2
38107	M15 240555	UK [England]	2015	invasive (unspecified/other)	Neisseria meningitidis	B	B	B	13	3	57	145	3	4	10	34	5	38	11	9	ST-269 complex	1161	3	3	1
38108	M15 240556	UK [England]	2015	invasive (unspecified/other)	Neisseria meningitidis	W	W	W	22	1	1	1	4	2	3	4	3	8	4	6	ST-11 complex	11	4	4	2
38109	M15 240557	UK [England]	2015	invasive (unspecified/other)	Neisseria meningitidis	W	W	W	886	24	1	510	115	2	3	4	3	8	4	6	ST-11 complex	11	NA	NA	2
38110	M15 240570	UK [England]	2015	invasive (unspecified/other)	Neisseria meningitidis	B	B	B	15	10	2	193	17	4	10	15	9	8	4	9	ST-269 complex	11492	NA	NA	1
38111	M15 240572	UK [England]	2015	invasive (unspecified/other)	Neisseria meningitidis	NG	cnl	cnl	4	1	1	206	4	5	4	17	15	30	7	12	ST-198 complex	823	4	3	1
38112	M15 240573	UK [England]	2015	invasive (unspecified/other)	Neisseria meningitidis	B	B	B	4	2	1	84	2	3	6	9	5	9	6	9	ST-41/44 complex	41	2	3	1
38113	M15 240574	UK [England]	2015	invasive (unspecified/other)	Neisseria meningitidis	B	B	B	1	6	3	100	7	8	10	77	25	6	3	22		5151	7	1	1
38114	M15 240575	UK [England]	2015	invasive (unspecified/other)	Neisseria meningitidis	W/Y	W/Y	W/Y	714	1	1	345	4	2	7	6	17	16	18	8	ST-167 complex	167	4	3	1
38115	M15 240576	UK [England]	2015	invasive (unspecified/other)	Neisseria meningitidis	W	W	W	22	1	1	1	4	2	3	4	3	8	4	6	ST-11 complex	11	4	4	2
38116	M15 240600	UK [England]	2015	invasive (unspecified/other)	Neisseria meningitidis	Y	Y	Y	25	1	1	26	4	12	5	18	9	11	9	17	ST-23 complex	1655	4	4	2
38117	M15 240602	UK [England]	2015	invasive (unspecified/other)	Neisseria meningitidis	B	B	B	894	9	1	525	16	1	5	13	53	26	41	3	ST-162 complex	162	16	2	3
38151	M15 240605	UK [Wales]	2015	invasive (unspecified/other)	Neisseria meningitidis	W	W	W	22	1	1	1	4	2	3	4	3	8	355	6	ST-11 complex	5121	4	4	2
39318	M15 240625	UK [England]	2015	invasive (unspecified/other)	Neisseria meningitidis	B	B	B	4	2	1	84	2	6	3	6	17	26	21	2		11752	2	3	1
39319	M15 240626	UK [England]	2015	invasive (unspecified/other)	Neisseria meningitidis	C	C	C	13	5	1	251	11	2	3	4	3	8	4	6	ST-11 complex	11	11	2	1
39320	M15 240629	UK [England]	2015	invasive (unspecified/other)	Neisseria meningitidis	W	W	W	22	1	1	1	4	2	3	4	3	8	4	6	ST-11 complex	11	4	4	2
39321	M15 240631	UK [England]	2015	invasive (unspecified/other)	Neisseria meningitidis	W/Y	W/Y	W/Y	25	1	1	26	4	12	5	18	9	11	9	17	ST-23 complex	1655	4	4	2
39322	M15 240634	UK [England]	2015	invasive (unspecified/other)	Neisseria meningitidis	B	B	B	15	4	2	193	1	4	10	15	9	8	11	9	ST-269 complex	269	1	1	1
39323	M15 240635	UK [England]	2015	invasive (unspecified/other)	Neisseria meningitidis	Y	Y	Y	25	1	1	26	4	12	5	18	NA	11	9	17		NA	4	4	2
39324	M15 240636	UK [England]	2015	invasive (unspecified/other)	Neisseria meningitidis	B	B	B	19	1	1	65	4	9	6	9	32	9	6	17	ST-41/44 complex	11753	4	4	2
39325	M15 240637	UK [England]	2015	invasive (unspecified/other)	Neisseria meningitidis	B	B	B	4	2	1	84	2	3	6	9	NA	9	6	9		NA	2	3	1
39326	M15 240638	UK [Wales]	2015	invasive (unspecified/other)	Neisseria meningitidis	W	W	W	22	1	1	1	4	2	3	4	3	8	4	6	ST-11 complex	11	4	4	2
39327	M15 240639	UK [England]	2015	invasive (unspecified/other)	Neisseria meningitidis	W	W	W	928	247	1	918	NA	11	5	18	8	11	4	21	ST-22 complex	184	NA	NA	1
39328	M15 240640	UK [England]	2015	invasive (unspecified/other)	Neisseria meningitidis	W	W	W	22	1	1	1	4	2	3	4	3	8	4	6	ST-11 complex	11	4	4	2
39329	M15 240641	UK [England]	2015	invasive (unspecified/other)	Neisseria meningitidis	B	B	B	13	3	57	145	3	4	10	34	5	38	11	330		4713	3	3	1
39330	M15 240642	UK [England]	2015	invasive (unspecified/other)	Neisseria meningitidis	Y	Y	Y	25	1	1	26	4	10	5	18	9	11	9	17	ST-23 complex	23	4	4	2
39331	M15 240643	UK [England]	2015	invasive (unspecified/other)	Neisseria meningitidis	W	W	W	22	1	1	1	4	2	3	4	3	8	4	6	ST-11 complex	11	4	4	2
39332	M15 240644	UK [Wales]	2015	invasive (unspecified/other)	Neisseria meningitidis	Y	Y	Y	25	1	1	26	4	12	5	18	9	11	9	17	ST-23 complex	1655	4	4	2
39333	M15 240645	UK [England]	2015	invasive (unspecified/other)	Neisseria meningitidis	W	W	W	16	1	1	60	4	11	5	18	NA	11	4	21		NA	4	4	2
39334	M15 240646	UK [England]	2015	invasive (unspecified/other)	Neisseria meningitidis	Y	Y	Y	929	119	1	548	NA	12	5	18	NA	11	9	17		NA	NA	NA	1
39335	M15 240648	UK [England]	2015	invasive (unspecified/other)	Neisseria meningitidis	W	W	W	22	1	1	1	4	2	3	4	3	8	4	6	ST-11 complex	11	4	4	2
39336	M15 240650	UK [England]	2015	invasive (unspecified/other)	Neisseria meningitidis	B	B	B	15	4	2	193	1	4	10	15	17	8	11	9	ST-269 complex	1049	1	1	1
39337	M15 240651	UK [England]	2015	invasive (unspecified/other)	Neisseria meningitidis	W	W	W	22	1	1	1	4	2	3	4	3	8	4	6	ST-11 complex	11	4	4	2
39338	M15 240652	UK [England]	2015	invasive (unspecified/other)	Neisseria meningitidis	B	B	B	4	2	79	84	2	3	206	9	NA	9	6	9		NA	2	3	1
39339	M15 240653	UK [England]	2015	invasive (unspecified/other)	Neisseria meningitidis	Y		Y	165	120	57	549	NA	10	5	23	9	11	9	17	ST-23 complex	9253	NA	NA	3
39340	M15 240657	UK [England]	2015	invasive (unspecified/other)	Neisseria meningitidis	W	W	W	22	1	1	1	4	2	3	4	3	8	4	6	ST-11 complex	11	4	4	2
39341	M15 240658	UK [England]	2015	invasive (unspecified/other)	Neisseria meningitidis	W	W	W	22	1	1	1	4	2	3	4	3	8	4	6	ST-11 complex	11	4	4	2
39342	M15 240659	UK [England]	2015	invasive (unspecified/other)	Neisseria meningitidis	W	W	W	22	1	1	1	4	2	3	4	3	8	4	6	ST-11 complex	11	4	4	2
39343	M15 240660	UK [England]	2015	invasive (unspecified/other)	Neisseria meningitidis	Y	Y	Y	25	1	1	26	4	12	5	NA	9	11	9	17		NA	4	4	2
39344	M15 240661	UK [England]	2015	invasive (unspecified/other)	Neisseria meningitidis	W	W	W	22	1	1	1	4	2	3	4	3	8	4	6	ST-11 complex	11	4	4	2
39345	M15 240662	UK [England]	2015	invasive (unspecified/other)	Neisseria meningitidis	W		W	22	1	1	1	4	2	3	4	3	8	4	6	ST-11 complex	11	4	4	2
39346	M15 240664	UK [England]	2015	invasive (unspecified/other)	Neisseria meningitidis	W	W	W	22	1	1	1	4	2	3	4	3	8	4	6	ST-11 complex	11	4	4	2
39347	M15 240697	UK [England]	2015	invasive (unspecified/other)	Neisseria meningitidis	Y	Y	Y	25	1	1	26	4	12	5	18	53	11	9	17	ST-23 complex	11754	4	4	2
39348	M15 240698	UK [England]	2015	invasive (unspecified/other)	Neisseria meningitidis	B	B	B	13	3	57	145	3	4	10	34	5	38	11	9	ST-269 complex	1161	3	3	1
39349	M15 240699	UK [England]	2015	invasive (unspecified/other)	Neisseria meningitidis	W	W	W	22	1	1	1	4	2	3	4	NA	8	4	6		NA	4	4	2
39350	M15 240701	UK [England]	2015	invasive (unspecified/other)	Neisseria meningitidis	B	B	B	15	4	2	193	1	4	10	15	9	8	11	9	ST-269 complex	269	1	1	1
39351	M15 240703	UK [England]	2015	invasive (unspecified/other)	Neisseria meningitidis	B	B	B	19	1	1	83	4	4	10	11	17	38	11	9	ST-269 complex	11755	4	4	2
39352	M15 240704	UK [England]	2015	invasive (unspecified/other)	Neisseria meningitidis	W	W	W	22	1	1	1	4	2	3	4	3	8	4	6	ST-11 complex	11	4	4	2
39353	M15 240705	UK [England]	2015	invasive (unspecified/other)	Neisseria meningitidis	C	C	C	13	5	1	251	11	2	3	4	3	8	4	6	ST-11 complex	11	11	2	1
39354	M15 240716	UK [Northern Ireland]	2015	invasive (unspecified/other)	Neisseria meningitidis	NG	E	E	30	17	57	54	37	8	25	7	17	21	26	49	ST-1157 complex	1157	NA	NA	3
39355	M15 240717	UK [England]	2015	invasive (unspecified/other)	Neisseria meningitidis	B	B	B	1	6	3	100	7	8	10	77	25	6	3	22		5151	7	1	1
39356	M15 240718	UK [England]	2015	invasive (unspecified/other)	Neisseria meningitidis	W	W	W	22	1	1	1	4	2	3	4	3	8	4	6	ST-11 complex	11	4	4	2
39357	M15 240719	UK [England]	2015	invasive (unspecified/other)	Neisseria meningitidis	B	B	B	1	6	3	NA	157	8	10	0	4	6	3	8		NA	NA	NA	1
39358	M15 240720	UK [England]	2015	invasive (unspecified/other)	Neisseria meningitidis	W	W	W	22	1	1	1	4	2	3	4	3	8	4	6	ST-11 complex	11	4	4	2
38520	M15 240722	UK [England]	2015	invasive (unspecified/other)	Neisseria meningitidis	W	W	W	22	1	1	1	4	2	3	4	3	8	4	6	ST-11 complex	11	4	4	2
39441	M15 240722b	UK	2015	invasive (unspecified/other)	Neisseria meningitidis	W	W	W	22	1	1	1	4	2	3	4	3	8	4	6	ST-11 complex	11	4	4	2
39359	M15 240723	UK [England]	2015	invasive (unspecified/other)	Neisseria meningitidis	Y	Y	Y	25	1	1	26	4	10	5	18	9	11	9	17	ST-23 complex	23	4	4	2
39360	M15 240724	UK [England]	2015	invasive (unspecified/other)	Neisseria meningitidis	B	B	B	19	1	1	93	13	12	6	9	17	9	73	9	ST-41/44 complex	337	13	4	2
39361	M15 240725	UK [England]	2015	invasive (unspecified/other)	Neisseria meningitidis	C	C	C	13	5	1	251	11	2	3	4	3	8	4	6	ST-11 complex	11	11	2	1
39362	M15 240727	UK [England]	2015	invasive (unspecified/other)	Neisseria meningitidis	B	B	B	4	2	6	84	2	3	6	9	9	9	6	9	ST-41/44 complex	303	2	3	1
39363	M15 240728	UK [England]	2015	invasive (unspecified/other)	Neisseria meningitidis	Y	Y	Y	25	1	1	26	4	12	5	18	9	11	9	17	ST-23 complex	1655	4	4	2
39364	M15 240729	UK [England]	2015	invasive (unspecified/other)	Neisseria meningitidis	B	B	B	4	2	1	84	2	8	6	9	5	9	6	9	ST-41/44 complex	1788	2	3	1
39365	M15 240730	UK [England]	2015	invasive (unspecified/other)	Neisseria meningitidis	W	W	W	22	1	1	1	4	2	3	4	3	8	4	6	ST-11 complex	11	4	4	2
39366	M15 240737	UK [England]	2015	invasive (unspecified/other)	Neisseria meningitidis	B	B	B	4	2	1	84	2	3	6	9	5	8	6	9	ST-41/44 complex	485	2	3	1
39367	M15 240738	UK [England]	2015	invasive (unspecified/other)	Neisseria meningitidis	B	B	B	47	1	57	90	15	12	5	12	35	192	22	17	ST-461 complex	1946	15	4	3
39368	M15 240740	UK [England]	2015	invasive (unspecified/other)	Neisseria meningitidis	W	W	W	22	1	1	1	4	2	3	4	3	8	4	6	ST-11 complex	11	4	4	2
39369	M15 240742	UK [England]	2015	invasive (unspecified/other)	Neisseria meningitidis	W	W	W	22	1	1	1	4	2	3	4	3	8	4	6	ST-11 complex	11	4	4	2
39370	M15 240743	UK [England]	2015	invasive (unspecified/other)	Neisseria meningitidis	B	B	B	4	2	1	84	2	3	6	9	5	9	6	9	ST-41/44 complex	41	2	3	1
39371	M15 240744	UK [England]	2015	invasive (unspecified/other)	Neisseria meningitidis	B	B	B	2	5	1	280	11	4	10	15	17	8	11	17	ST-269 complex	479	11	2	1
39372	M15 240745	UK [England]	2015	invasive (unspecified/other)	Neisseria meningitidis	B	B	B	4	2	1	84	2	8	5	9	5	9	6	9	ST-41/44 complex	9544	2	3	1
39373	M15 240746	UK [England]	2015	invasive (unspecified/other)	Neisseria meningitidis	W	W	W	13	3	1	145	3	2	3	4	3	8	4	6	ST-11 complex	11	3	3	1
39374	M15 240747	UK [England]	2015	invasive (unspecified/other)	Neisseria meningitidis	B	B	B	1	6	3	100	7	8	10	NA	4	6	3	8		NA	7	1	1
39375	M15 240748	UK [England]	2015	invasive (unspecified/other)	Neisseria meningitidis	B	B	B	4	2	1	84	2	3	6	9	5	9	6	9	ST-41/44 complex	41	2	3	1
39376	M15 240749	UK [England]	2015	invasive (unspecified/other)	Neisseria meningitidis	Y	Y	Y	25	1	1	26	4	12	5	18	9	11	9	17	ST-23 complex	1655	4	4	2
39377	M15 240750	UK [England]	2015	invasive (unspecified/other)	Neisseria meningitidis	Y	Y	Y	21	1	1	7	4	6	5	173	13	5	24	17	ST-174 complex	1466	4	4	2
39378	M15 240751	UK [Northern Ireland]	2015	invasive (unspecified/other)	Neisseria meningitidis	C	C	C	22	1	1	1	4	2	3	4	3	8	4	6	ST-11 complex	11	4	4	2

39379	M15 240752	UK [England]	2015	invasive (unspecified/other)	Neisseria meningitidis	Y	Y	Y	25	1	1	26	4	12	5	18	9	11	9	17	ST-23 complex	1655	4	4	2
41451	M15 240753	UK [England]	2015	invasive (unspecified/other)	Neisseria meningitidis	W	W	W	13	3	1	145	3	2	3	4	3	8	4	6	ST-11 complex	11	3	3	1
39380	M15 240755	UK [England]	2015	invasive (unspecified/other)	Neisseria meningitidis	NG	B	B	31	3	1	58	NA	1	5	13	53	26	775	3	ST-162 complex	11877	NA	NA	3
39381	M15 240757	UK [England]	2015	invasive (unspecified/other)	Neisseria meningitidis	B	B	B	16	1	1	60	4	11	5	5	8	11	24	21	ST-22 complex	1617	4	4	2
39382	M15 240758	UK [England]	2015	invasive (unspecified/other)	Neisseria meningitidis	B	B	B	14	121	57	142	158	6	5	12	12	12	13	14		1345	NA	NA	1
39383	M15 240759	UK [England]	2015	invasive (unspecified/other)	Neisseria meningitidis	NG	B	B	12	1	2	551	4	3	6	9	5	26	6	9	ST-41/44 complex	8054	4	3	1
39384	M15 240760	UK [England]	2015	invasive (unspecified/other)	Neisseria meningitidis	B	B	B	15	4	2	193	1	4	10	6	9	21	732	9	ST-269 complex	11238	1	1	1
39385	M15 240762	UK [England]	2015	invasive (unspecified/other)	Neisseria meningitidis	W	W	W	22	1	1	1	4	2	3	4	3	8	4	6	ST-11 complex	11	4	4	2
39386	M15 240763	UK [England]	2015	invasive (unspecified/other)	Neisseria meningitidis	B	B	B	16	1	4	59	4	132	3	19	17	62	53	2		9088	4	4	2
39387	M15 240765	UK [England]	2015	invasive (unspecified/other)	Neisseria meningitidis	W	W	W	16	22	104	97	32	11	5	168	8	11	4	21	ST-22 complex	1286	NA	NA	2
39388	M15 240766	UK [England]	2015	invasive (unspecified/other)	Neisseria meningitidis	C	C	C	13	5	1	251	11	2	3	4	3	8	4	6	ST-11 complex	11	11	2	1
39389	M15 240767	UK [England]	2015	invasive (unspecified/other)	Neisseria meningitidis	B	B	B	21	1	1	7	4	4	10	159	4	6	3	8	ST-32 complex	11756	4	4	2
39391	M15 240770	UK [England]	2015	invasive (unspecified/other)	Neisseria meningitidis	B	B	B	510	28	8	599	30	4	10	5	4	6	3	8	ST-32 complex	32	NA	NA	1
39392	M15 240772	UK [England]	2015	invasive (unspecified/other)	Neisseria meningitidis	B	B	B	740	5	77	552	60	7	5	13	13	36	53	15	ST-213 complex	8955	NA	NA	3
39393	M15 240773	UK [England]	2015	invasive (unspecified/other)	Neisseria meningitidis	B	B	B	15	10	2	193	17	4	10	15	9	NA	11	9		NA	NA	NA	1
39394	M15 240775	UK [England]	2015	invasive (unspecified/other)	Neisseria meningitidis	B	B	B	4	2	1	84	2	3	6	34	5	11	6	9	ST-41/44 complex	1194	2	3	1
39395	M15 240778	UK [England]	2015	invasive (unspecified/other)	Neisseria meningitidis	W	W	W	22	1	1	1	4	2	3	4	3	8	4	6	ST-11 complex	11	4	4	2
39396	M15 240779	UK [England]	2015	invasive (unspecified/other)	Neisseria meningitidis	W	W	W	22	1	1	1	4	2	3	4	3	8	4	6	ST-11 complex	11	4	4	2
39397	M15 240781	UK [England]	2015	invasive (unspecified/other)	Neisseria meningitidis	B	B	B	1	6	7	100	7	12	5	5	17	3	10	124		11757	7	1	1
39398	M15 240782	UK [England]	2015	invasive (unspecified/other)	Neisseria meningitidis	W	W	W	22	1	1	1	4	2	3	4	3	8	4	6	ST-11 complex	11	4	4	2
39399	M15 240783	UK [England]	2015	invasive (unspecified/other)	Neisseria meningitidis	B	B	B	13	3	57	145	3	4	10	34	5	38	11	9	ST-269 complex	1161	3	3	1
39400	M15 240784	UK [England]	2015	invasive (unspecified/other)	Neisseria meningitidis	B	B	B	4	2	1	84	2	3	6	34	5	11	6	9	ST-41/44 complex	1194	2	3	1
39401	M15 240785	UK [England]	2015	invasive (unspecified/other)	Neisseria meningitidis	W	W	W	22	1	1	1	4	2	3	4	3	8	4	6	ST-11 complex	11	4	4	2
39402	M15 240786	UK [England]	2015	invasive (unspecified/other)	Neisseria meningitidis	B	B	B	25	1	1	28	4	8	6	9	5	106	18	9	ST-41/44 complex	10269	4	4	2
39403	M15 240787	UK [England]	2015	invasive (unspecified/other)	Neisseria meningitidis	C	C	C	129	5	1	553	11	2	3	4	3	8	4	6	ST-11 complex	11	11	2	1
39404	M15 240788	UK [England]	2015	invasive (unspecified/other)	Neisseria meningitidis	W	W	W	22	1	1	1	4	2	3	4	3	8	4	6	ST-11 complex	11	4	4	2
39405	M15 240789	UK [Northern Ireland]	2015	invasive (unspecified/other)	Neisseria meningitidis	B	B	B	13	3	57	209	3	8	25	7	17	21	26	49	ST-1157 complex	1157	3	3	1
39406	M15 240790	UK [England]	2015	invasive (unspecified/other)	Neisseria meningitidis	B	B	B	13	3	57	145	3	4	10	34	5	38	11	9	ST-269 complex	1161	3	3	1
39407	M15 240793	UK [England]	2015	invasive (unspecified/other)	Neisseria meningitidis	B	B	B	19	1	1	65	4	9	6	9	9	9	64	9	ST-41/44 complex	409	4	4	2
39408	M15 240794	UK [England]	2015	invasive (unspecified/other)	Neisseria meningitidis	W	W	W	22	1	1	1	4	2	3	4	3	8	4	6	ST-11 complex	11	4	4	2
39409	M15 240795	UK [England]	2015	invasive (unspecified/other)	Neisseria meningitidis	C	C	C	13	5	1	251	11	2	3	4	3	8	4	6	ST-11 complex	11	11	2	1
39410	M15 240796	UK [England]	2015	invasive (unspecified/other)	Neisseria meningitidis	W	W	W	22	1	1	1	4	2	3	4	3	8	4	6	ST-11 complex	11	4	4	2
39411	M15 240797	UK [England]	2015	invasive (unspecified/other)	Neisseria meningitidis	W	W	W	13	3	1	145	3	2	3	4	3	8	4	6	ST-11 complex	11	3	3	1
39412	M15 240798	UK [England]	2015	invasive (unspecified/other)	Neisseria meningitidis	W	W	W	22	1	1	1	4	2	3	4	3	8	4	6	ST-11 complex	11	4	4	2
39413	M15 240799	UK [England]	2015	invasive (unspecified/other)	Neisseria meningitidis	B	B	B	4	2	1	84	2	6	3	6	17	26	21	8		4051	2	3	1
39414	M15 240801	UK [Wales]	2015	invasive (unspecified/other)	Neisseria meningitidis	B	B	B	147	122	1	554	159	8	4	6	17	5	18	2	ST-103 complex	103	NA	NA	2
39415	M15 240802	UK [England]	2015	invasive (unspecified/other)	Neisseria meningitidis	B	B	B	1	6	3	100	7	8	10	5	4	5	3	8	ST-32 complex	34	7	1	1
39416	M15 240803	UK [England]	2015	invasive (unspecified/other)	Neisseria meningitidis	W	W	W	16	1	1	60	4	11	5	18	154	11	24	21	ST-22 complex	1281	4	4	2
39417	M15 240805	UK [England]	2015	invasive (unspecified/other)	Neisseria meningitidis	B	B	B	185	4	77	127	1	7	5	1	13	36	53	15	ST-213 complex	213	1	1	3
39418	M15 240806	UK [England]	2015	invasive (unspecified/other)	Neisseria meningitidis	B	B	B	245	123	1	186	93	20	4	6	21	41	11	2		11758	NA	NA	1
39419	M15 240807	UK [England]	2015	invasive (unspecified/other)	Neisseria meningitidis	Y	Y	Y	25	1	1	26	4	12	5	18	9	11	9	17	ST-23 complex	1655	4	4	2
39420	M15 240808	UK [England]	2015	invasive (unspecified/other)	Neisseria meningitidis	Y	Y	Y	25	1	1	26	4	12	5	18	9	11	9	17	ST-23 complex	1655	4	4	2
39421	M15 240816	UK [England]	2015	invasive (unspecified/other)	Neisseria meningitidis	W	W	W	22	1	1	1	4	2	3	4	3	8	4	6	ST-11 complex	11	4	4	2
39422	M15 240818	UK [Wales]	2015	invasive (unspecified/other)	Neisseria meningitidis	W	W	W	22	1	1	1	4	2	3	4	3	8	4	6	ST-11 complex	11	4	4	2
39423	M15 240819	UK [England]	2015	invasive (unspecified/other)	Neisseria meningitidis	W	W	W	22	1	1	1	4	2	3	4	3	8	4	6	ST-11 complex	11	4	4	2
39424	M15 240820	UK [England]	2015	invasive (unspecified/other)	Neisseria meningitidis	W	W	W	321	111	1	598	NA	8	6	6	17	8	31	8		9316	NA	NA	1
39425	M15 240821	UK [England]	2015	invasive (unspecified/other)	Neisseria meningitidis	B	B	B	47	1	57	90	15	12	5	12	35	192	22	17	ST-461 complex	1946	15	4	3
39426	M15 240822	UK [Wales]	2015	invasive (unspecified/other)	Neisseria meningitidis	Y	Y	Y	25	1	1	26	4	12	5	18	53	11	9	17	ST-23 complex	11754	4	4	2
39427	M15 240826	UK [England]	2015	invasive (unspecified/other)	Neisseria meningitidis	W	W	W	22	1	1	1	4	2	3	4	3	8	4	6	ST-11 complex	11	4	4	2
39429	M15 240829	UK [England]	2015	invasive (unspecified/other)	Neisseria meningitidis	W	W	W	22	1	1	1	4	2	3	4	3	8	4	6	ST-11 complex	11	4	4	2
39430	M15 240830	UK [England]	2015	invasive (unspecified/other)	Neisseria meningitidis	W	W	W	22	1	1	1	4	2	3	4	3	8	4	6	ST-11 complex	11	4	4	2
39431	M15 240831	UK [England]	2015	invasive (unspecified/other)	Neisseria meningitidis	W	W	W	22	1	1	1	4	2	3	4	3	8	4	6	ST-11 complex	11	4	4	2
39432	M15 240832	UK [England]	2015	invasive (unspecified/other)	Neisseria meningitidis	Y	Y	Y	25	1	1	26	4	12	5	18	9	11	9	17	ST-23 complex	1655	4	4	2
39433	M15 240833	UK [England]	2015	invasive (unspecified/other)	Neisseria meningitidis	W	W	W	22	1	1	1	4	2	3	4	3	8	4	6	ST-11 complex	11	4	4	2
39434	M15 240835	UK [England]	2015	invasive (unspecified/other)	Neisseria meningitidis	B	B	B	15	4	2	193	1	4	10	15	9	8	11	9	ST-269 complex	269	1	1	1
39435	M15 240844	UK [England]	2015	invasive (unspecified/other)	Neisseria meningitidis	W	W	W	22	1	1	1	4	2	3	4	3	8	4	6	ST-11 complex	11	4	4	2
39436	M15 240845	UK [England]	2015	invasive (unspecified/other)	Neisseria meningitidis	W	W	W	22	1	1	1	4	2	3	4	3	8	4	6	ST-11 complex	11	4	4	2
41452	M15 240846	UK [England]	2015	invasive (unspecified/other)	Neisseria meningitidis	B	B	B	21	1	1	7	4	4	10	5	26	6	3	8	ST-32 complex	1096	4	4	2
39437	M15 240847	UK [England]	2015	invasive (unspecified/other)	Neisseria meningitidis	Y	Y	Y	25	1	1	26	4	12	5	18	9	11	9	17	ST-23 complex	1655	4	4	2
39438	M15 240848	UK [England]	2015	invasive (unspecified/other)	Neisseria meningitidis	W	W	W	22	1	1	1	4	2	3	4	3	8	4	6	ST-11 complex	11	4	4	2
39439	M15 240849	UK [England]	2015	invasive (unspecified/other)	Neisseria meningitidis	B	B	B	4	2	1	84	2	3	6	9	5	9	6	22	ST-41/44 complex	5440	2	3	1
39440	M15 240850	UK [England]	2015	invasive (unspecified/other)	Neisseria meningitidis	Y	Y	Y	25	1	1	26	4	12	5	18	9	11	9	17	ST-23 complex	1655	4	4	2
40321	M15 240851	UK [Wales]	2015	invasive (unspecified/other)	Neisseria meningitidis	B	B	B	680	26	77	912	25	7	5	1	13	212	53	15	ST-213 complex	3872	NA	NA	1
40322	M15 240852	UK [England]	2015	invasive (unspecified/other)	Neisseria meningitidis	W	W	W	22	1	1	1	4	2	3	4	3	8	4	6	ST-11 complex	11	4	4	2
41453	M15 240853	UK [England]	2015	invasive (unspecified/other)	Neisseria meningitidis	B	B	B	45	11	77	87	10	7	395	1	13	36	53	15	ST-213 complex	9194	NA	NA	3
40324	M15 240854	UK [England]	2015	invasive (unspecified/other)	Neisseria meningitidis	B	B	B	13	3	1	145	3	7	5	798	13	82	53	15	ST-213 complex	11880	3	3	1
41454	M15 240855	UK [England]	2015	invasive (unspecified/other)	Neisseria meningitidis	B	B	B	1	6	3	100	7	8	10	77	4	6	3	8	ST-32 complex	749	7	1	1
41455	M15 240856	UK [England]	2015	invasive (unspecified/other)	Neisseria meningitidis	W	W	W	22	1	1	1	4	2	3	4	3	8	4	6	ST-11 complex	11	4	4	2
41456	M15 240857	UK [England]	2015	invasive (unspecified/other)	Neisseria meningitidis	B	B	B	316	27	40	155	26	12	5	12	35	192	22	17	ST-461 complex	1946	NA	NA	3
41457	M15 240859	UK [England]	2015	invasive (unspecified/other)	Neisseria meningitidis	B	B	B	13	3	57	145	3	4	10	2	5	38	11	9	ST-269 complex	275	3	3	1
41458	M15 240860	UK [Wales]	2015	invasive (unspecified/other)	Neisseria meningitidis	W	W	W	22	1	1	1	4	2	3	4	3	8	4	6	ST-11 complex	11	4	4	2
41459	M15 240861	UK [England]	2015	invasive (unspecified/other)	Neisseria meningitidis	B	B	B	22	1	1	1	4	662	3	4	3	8	4	6	ST-11 complex	10651	4	4	2
41460	M15 240862	UK [England]	2015	invasive (unspecified/other)	Neisseria meningitidis	W	W	W	16	22	1	97	32	11	5	NA	8	11	4	21		NA	NA	NA	2
41461	M15 240863	UK [England]	2015	invasive (unspecified/other)	Neisseria meningitidis	B	B	B	16	1	4	59	4	4	10	11	17	6	10	12	ST-35 complex	457	4	4	2
41462	M15 240864	UK [England]	2015	invasive (unspecified/other)	Neisseria meningitidis	W	W	W	22	1	1	1	4	2	3	4	3	8	4	6	ST-11 complex	11	4	4	2
41463	M15 240866	UK [England]	2015	invasive (unspecified/other)	Neisseria meningitidis	W	W	W	22	1	1	1	4	2	3	4	3	8	4	6	ST-11 complex	11	4	4	2
41464	M15 240868	UK [England]	2015	invasive (unspecified/other)	Neisseria meningitidis	B	B	B	4	37	1	84	125	3	6	9	9	9	6	9	ST-41/44 complex	303	NA	NA	1
41465	M15 240869	UK [England]	2015	invasive (unspecified/other)	Neisseria meningitidis	B	B	B	15	4	2	193	1	4	10	15	9	8	11	9	ST-269 complex	269	1	1	1
41466	M15 240870	UK [England]	2015	invasive (unspecified/other)	Neisseria meningitidis	Y	Y	Y	25	1	1	26	4	12	5	18	9	11	9	17	ST-23 complex	1655	4	4	2
41467	M15 240871	UK [England]	2015	invasive (unspecified/other)	Neisseria meningitidis	W	W	W	22	1	1	1	4	2	3	4	3	8	4	6	ST-11 complex	11	4	4	2
41468	M15 240872	UK [England]	2015	invasive (unspecified/other)	Neisseria meningitidis	C	C	C	22	1	1	1	4	2	3	4	3	8	4	6	ST-11 complex	11	4	4	2
41469	M15 240873	UK [England]	2015	invasive (unspecified/other)	Neisseria meningitidis	W	W	W	22	1	1	1	4	2	3	4	3	8	4	6	ST-11 complex	11	4	4	2
41470	M15 240876	UK [England]	2015	invasive (unspecified/other)	Neisseria meningitidis	W	W	W	13	3	1	145	3	2	3	4	3	8	4	6	ST-11 complex	11	3	3	1
41471	M15 240877	UK [England]	2015	invasive (unspecified/other)	Neisseria meningitidis	B	B	B	4	2	1	84	2	3	6	9	5	8	6	9	ST-41/44 complex	485	2	3	1
41472	M15 240878	UK [England]	2015	invasive (unspecified/other)	Neisseria meningitidis	C	C	C	129	5	1	553	11	2	3	4	NA	8	4	6		NA	11	2	1
41473	M15 240879	UK [England]	2015	invasive (unspecified/other)	Neisseria meningitidis	W	W	W	22	1	1	1	4	2	3	4	3	8	4	6	ST-11 complex	11	4	4	2
41475	M15 240881	UK [England]	2015	invasive (unspecified/other)	Neisseria meningitidis	B	B	B	19	1	1	65	4	9	3	9	9	9	64	9	ST-41/44 complex	6058	4	4	2
41476	M15 240882	UK [England]	2015	invasive (unspecified/other)	Neisseria meningitidis	Y	Y	Y	25	1	1	26	4	10	5	18	9	11	9	17	ST-23 complex	23	4	4	2
41477	M15 240883	UK [England]	2015	invasive (unspecified/other)	Neisseria meningitidis	B	B	B	4	2	1	84	2	3	6	9	5	8	6	9	ST-41/44 complex	485	2	3	1
41478	M15 240885	UK [England]	2015	invasive (unspecified/other)	Neisseria meningitidis	W	W	W	22	1	1	1	4	2	3	4	3	8	4	6	ST-11 complex	11	4	4	2
41479	M15 240886	UK [England]	2015	invasive (unspecified/other)	Neisseria meningitidis	B	B	B	13	3	57	145	3	4	10	34	5	38	11	330		4713	3	3	1
41480	M15 240887	UK [England]	2015	invasive (unspecified/other)	Neisseria meningitidis	Y	Y	Y	25	1	1	26	4	12	5	18	9	11	9	17	ST-23 complex	1655	4	4	2
41481	M15 240888	UK [England]	2015	invasive (unspecified/other)	Neisseria meningitidis	W	W	W	22	1	1	1	4	2	3	4	3	8	4	6	ST-11 complex	11	4	4	2
41482	M15 240892	UK [England]	2015	invasive (unspecified/other)	Neisseria meningitidis	B	B	B	401	124	1	645	137	4	450	5	4	9	3	17	ST-32 complex	11917	NA	NA	3
41483	M15 240893	UK [England]	2015	invasive (unspecified/other)	Neisseria meningitidis	NG	W	W	22	1	1	1	4	2	3	4	3	8	4	6	ST-11 complex	11	4	4	2
41484	M15 240894	UK [England]	2015	invasive (unspecified/other)	Neisseria meningitidis	C	C	C	138	5	1	112	11	NA	3	4	3	8	4	6		NA	11	2	2
41485	M15 240895	UK [Northern Ireland]	2015	invasive (unspecified/other)	Neisseria meningitidis	B	B	B	19	18	1	83	87	4	10	2	5	563	11	9	ST-269 complex	8616	NA	NA	2
41486	M15 240896	UK [England]	2015	invasive (unspecified/other)	Neisseria meningitidis	W	W	W	22	1	1	1	4	2	3	4	3	8	4	6	ST-11 complex	11	4	4	2
41487	M15 240897	UK [England]	2015	invasive (unspecified/other)	Neisseria meningitidis	B	B	B	13	3	57	145	3	4	10	34	5	38	11	9	ST-269 complex	1161	3	3	1
41488	M15 240899	UK [England]	2015	invasive (unspecified/other)	Neisseria meningitidis	B	B	B	45	8	77	87	20	7	5	1	13	36	53	8	ST-213 complex	4224	20	4	3
41489	M15 240900	UK [England]	2015	invasive (unspecified/other)	Neisseria meningitidis	W	W	W	22	1	1	1	4	2	3	4	3	8	NA	6		NA	4	4	2
41490	M15 240901	UK [England]	2015	invasive (unspecified/other)	Neisseria meningitidis	B	B	B	13	3	57	145	3	4	5	34	5	38	11	9		5849	3	3	1
41491	M15 240902	UK [England]	2015	invasive (unspecified/other)	Neisseria meningitidis	B	B	B	4	2	79	84	2	3	6	9	5	9	6	9	ST-41/44 complex	41	2	3	1
41492	M15 240903	UK [England]	2015	invasive (unspecified/other)	Neisseria meningitidis	B	B	B	19	1	1	65	4	9	5	9	9	3	6	9	ST-41/44 complex	11918	4	4	2
41493	M15 240904	UK [England]	2015	invasive (unspecified/other)	Neisseria meningitidis	C	C	C	0	5	1	917	11	2	3	4	3	8	4	6	ST-11 complex	11	11	2	NA
41494	M15 240905	UK [England]	2015	invasive (unspecified/other)	Neisseria meningitidis	W	W	W	22	1	1	1	4	2	3	4	3	8	4	6	ST-11 complex	11	4	4	2
40316	M15 240907	UK	2015	carrier	Neisseria meningitidis	W		W	22	1	1	1	4	2	3	4	3	8	4	6	ST-11 complex	11	4	4	2
40317	M15 240908	UK	2015	carrier	Neisseria meningitidis	C	C	C	13	3	57	209	3	8	25	7	17	21	26	49	ST-1157 complex	1157	3	3	1
40318	M15 240909	UK	2015	carrier	Neisseria meningitidis	NG	W	W	22	1	1	1	4	2	3	4	3	8	4	6	ST-11 complex	11	4	4	2
40319	M15 240910	UK	2015	carrier	Neisseria meningitidis	C	C	C	13	3	57	209	3	8	25	7	17	21	26	49	ST-1157 complex	1157	3	3	1
41495	M15 240911	UK [England]	2015	invasive (unspecified/other)	Neisseria meningitidis	Y	Y	Y	25	1	1	26	4	12	5	18	9	11	9	17	ST-23 complex	1655	4	4	2
41496	M15 240912	UK [England]	2015	invasive (unspecified/other)	Neisseria meningitidis	B	B	B	13	3	57	145	3	4	10	34	5	38	11	9	ST-269 complex	1161	3	3	1
41497	M15 240913	UK [England]	2015	invasive (unspecified/other)	Neisseria meningitidis	Y	Y	Y	25	1	1	26	4	10	5	18	9	11	9	17	ST-23 complex	23	4	4	2
41498	M15 240914	UK [Wales]	2015	invasive (unspecified/other)	Neisseria meningitidis	Y	Y	Y	25	1	1	26	4	12	5	18	9	11	9	17	ST-23 complex	1655	4	4	2
41499	M15 240915	UK [England]	2015	invasive (unspecified/other)	Neisseria meningitidis	Y		Y	25	1	1	26	4	12	5	18	9	11	9	17	ST-23 complex	1655	4	4	2
41500	M15 240917	UK [England]	2015	invasive (unspecified/other)	Neisseria meningitidis	B	B	B	45	33	77	87	14	7	5	1	13	36	53	15	ST-213 complex	213	NA	NA	3
41501	M15 240918	UK [England]	2015	invasive (unspecified/other)	Neisseria meningitidis	W	W	W	22	1	1	1	4	2	3	4	3	8	4	6	ST-11 complex	11	4	4	2
41502	M15 240919	UK [England]	2015	invasive (unspecified/other)	Neisseria meningitidis	W	W	W	22	1	1	1	4	2	3	4	3	8	4	6	ST-11 complex	11	4	4	2
41503	M15 240920	UK [England]	2015	invasive (unspecified/other)	Neisseria meningitidis	B	B	B	110	21	121	171	19	3	6	219	5	9	6	9	ST-41/44 complex	2713	NA	NA	1
41504	M15 240922	UK [England]	2015	invasive (unspecified/other)	Neisseria meningitidis	Y	Y	Y	25	1	1	26	4	10	5	18	NA	11	9	17		NA	4	4	2
41505	M15 240923	UK [England]	2015	invasive (unspecified/other)	Neisseria meningitidis	B	B	B	4	2	1	84	2	3	6	34	5	11	6	9	ST-41/44 complex	1194	2	3	1
41506	M15 240924	UK [England]	2015	invasive (unspecified/other)	Neisseria meningitidis	B	B	B	19	1	1	83	4	4	10	11	17	38	11	9	ST-269 complex	11755	4	4	2
41507	M15 240925	UK [England]	2015	invasive (unspecified/other)	Neisseria meningitidis	B	B	B	14	1	1	142	13	3	6	9	17	9	6	9	ST-41/44 complex	482	13	3	1
41508	M15 240926	UK [England]	2015	invasive (unspecified/other)	Neisseria meningitidis	Y	Y	Y	25	1	1	26	4	12	5	18	9	11	9	17	ST-23 complex	1655	4	4	2
41509	M15 240929	UK [England]	2015	invasive (unspecified/other)	Neisseria meningitidis	B	B	B	510	28	8	599	30	4	10	48	4	6	3	8	ST-32 complex	7460	NA	NA	1
41510	M15 240930	UK [England]	2015	invasive (unspecified/other)	Neisseria meningitidis	B	B	B	510	28	8	599	30	4	10	48	4	6	3	8	ST-32 complex	7460	NA	NA	1
41511	M15 240931	UK [England]	2015	invasive (unspecified/other)	Neisseria meningitidis	B	B	B	339	4	81	913	1	9	3	9	9	9	64	9	ST-41/44 complex	6058	1	1	3
41512	M15 240933	UK [England]	2015	invasive (unspecified/other)	Neisseria meningitidis	B	B	B	100	125	2	381	147	12	6	9	9	9	363	9	ST-41/44 complex	6359	NA	NA	1
41513	M15 240934	UK [England]	2015	invasive (unspecified/other)	Neisseria meningitidis	B	B	B	15	4	1	193	1	4	10	15	17	8	11	9	ST-269 complex	1049	1	1	1
41514	M15 240935	UK [England]	2015	invasive (unspecified/other)	Neisseria meningitidis	Y	Y	Y	25	1	1	26	4	12	5	18	9	11	9	17	ST-23 complex	1655	4	4	2
41515	M15 240936	UK [England]	2015	invasive (unspecified/other)	Neisseria meningitidis	W	W	W	9	1	1	187	47	2	3	4	3	8	4	6	ST-11 complex	11	NA	NA	1
41516	M15 240937	UK [England]	2015	invasive (unspecified/other)	Neisseria meningitidis	W	W	W	22	1	1	1	4	2	3	4	3	8	4	6	ST-11 complex	11	4	4	2
41517	M15 240938	UK [Wales]	2015	invasive (unspecified/other)	Neisseria meningitidis	Y	Y	Y	25	1	1	26	4	12	5	18	9	11	9	17	ST-23 complex	1655	4	4	2
41518	M15 240939	UK [England]	2015	invasive (unspecified/other)	Neisseria meningitidis	B	B	B	4	2	1	84	2	3	6	9	5	9	6	6	ST-41/44 complex	5534	2	3	1
41519	M15 240940	UK [England]	2015	invasive (unspecified/other)	Neisseria meningitidis	W		W	22	1	1	1	4	2	3	4	3	8	4	6	ST-11 complex	11	4	4	2
41520	M15 240941	UK [England]	2015	invasive (unspecified/other)	Neisseria meningitidis	Y	Y	Y	25	1	1	26	4	12	5	18	NA	11	9	17		NA	4	4	2
41521	M15 240945	UK [England]	2015	invasive (unspecified/other)	Neisseria meningitidis	Y	Y	Y	25	1	1	26	4	12	5	18	9	11	9	17	ST-23 complex	1655	4	4	2
41522	M15 240946	UK [England]	2015	invasive (unspecified/other)	Neisseria meningitidis	W	W	W	22	1	1	1	4	2	3	4	3	8	4	6	ST-11 complex	11	4	4	2

41523	M15 240947	UK [England]	2015	invasive (unspecified/other)	Neisseria meningitidis	B	B	B		47	1	57	90	15	12	5	NA	35	60	22	17		NA	15	4	3
41524	M15 240949	UK [England]	2015	invasive (unspecified/other)	Neisseria meningitidis	W	W	W		25	1	1	26	4	10	5	NA	NA	11	NA	17		NA	4	4	2
91759	M15 240951	UK [England]	2015	invasive (unspecified/other)	Neisseria meningitidis	W		W		22	1	1	1131	4	2	3	4	3	8	4	6	ST-11 complex	11	4	4	2
41525	M15 240952	UK [England]	2015	invasive (unspecified/other)	Neisseria meningitidis	W	W	W		22	1	1	1	4	2	3	4	3	8	4	6	ST-11 complex	11	4	4	2
41526	M15 240953	UK [England]	2015	invasive (unspecified/other)	Neisseria meningitidis	NG	NG	NG		151	1	1	130	4	6	7	4	56	26	18	8	ST-175 complex	175	4	4	2
41527	M15 240954	UK [England]	2015	invasive (unspecified/other)	Neisseria meningitidis	B	B	B		45	8	77	87	20	7	5	1	13	36	53	15	ST-213 complex	213	20	4	3
41529	M15 240957	UK [England]	2015	invasive (unspecified/other)	Neisseria meningitidis	Y	Y	Y		25	1	1	26	4	10	5	18	9	11	9	17	ST-23 complex	23	4	4	2
41530	M15 240958	UK [England]	2015	invasive (unspecified/other)	Neisseria meningitidis	W	W	W		22	1	1	1	4	2	3	4	3	8	4	6	ST-11 complex	11	4	4	2
41531	M15 240960	UK [England]	2015	invasive (unspecified/other)	Neisseria meningitidis	W	W	W		22	1	1	1	4	2	3	4	3	8	4	6	ST-11 complex	11	4	4	2
41532	M15 240961	UK [Wales]	2015	invasive (unspecified/other)	Neisseria meningitidis	B	B	B		13	3	57	145	3	4	10	34	NA	38	11	9		NA	3	3	1
41533	M15 240963	UK [England]	2015	invasive (unspecified/other)	Neisseria meningitidis	W	W	W		22	1	1	1	4	662	3	4	3	8	4	6	ST-11 complex	10651	4	4	2
41534	M15 240965	UK [England]	2015	invasive (unspecified/other)	Neisseria meningitidis	Y	Y	Y		25	1	1	26	4	12	5	18	9	11	9	17	ST-23 complex	1655	4	4	2
41535	M15 240967	UK [England]	2015	invasive (unspecified/other)	Neisseria meningitidis	Y	Y	Y		25	1	1	26	4	10	5	18	9	11	9	17	ST-23 complex	23	4	4	2
41536	M15 240968	UK [England]	2015	invasive (unspecified/other)	Neisseria meningitidis	W	W	W		22	1	1	1	4	2	3	4	NA	8	4	6		NA	4	4	2
41537	M15 240969	UK [England]	2015	invasive (unspecified/other)	Neisseria meningitidis	B	B	B		4	2	1	84	2	3	6	9	5	8	6	9	ST-41/44 complex	485	2	3	1
95901	M15 240970	UK [England]	2015	invasive (unspecified/other)	Neisseria meningitidis	W	W	W		22	1	1	1	4	2	3	4	3	8	4	6	ST-11 complex	11	4	4	2
41538	M15 240973	UK [England]	2015	invasive (unspecified/other)	Neisseria meningitidis	B	B	B		4	2	1	84	2	3	6	9	5	11	6	9	ST-41/44 complex	154	2	3	1
41539	M15 240974	UK [England]	2015	invasive (unspecified/other)	Neisseria meningitidis	W	W	W		16	1	1	60	4	11	5	18	154	11	24	21	ST-22 complex	1281	4	4	2
41540	M15 240975	UK [Wales]	2015	invasive (unspecified/other)	Neisseria meningitidis	B	B	B		13	3	57	145	3	4	10	34	5	38	11	9	ST-269 complex	1161	3	3	1
41542	M15 240977	UK [England]	2015	invasive (unspecified/other)	Neisseria meningitidis	B	B	B		930	75	77	646	161	7	5	1	13	3	NA	15		NA	NA	NA	3
41543	M15 240978	UK [England]	2015	invasive (unspecified/other)	Neisseria meningitidis	W	W	W		22	1	1	1	4	2	3	4	3	8	4	6	ST-11 complex	11	4	4	2
41544	M15 240979	UK [England]	2015	invasive (unspecified/other)	Neisseria meningitidis	B	B	B		4	2	1	84	2	3	6	0	5	8	6	9		NA	2	3	1
41545	M15 240980	UK [England]	2015	invasive (unspecified/other)	Neisseria meningitidis	B	B	B		21	1	1	7	4	1	5	13	NA	26	41	3		NA	4	4	2
41546	M15 240981	UK [Northern Ireland]	2015	invasive (unspecified/other)	Neisseria meningitidis	W	W	W		22	1	1	1	4	2	3	4	3	8	4	6	ST-11 complex	11	4	4	2
41560	M15 240982	UK	2015	invasive (unspecified/other)	Neisseria meningitidis	W	W	W		22	1	1	1	4	2	3	4	3	8	4	6	ST-11 complex	11	4	4	2
41547	M15 240983	UK [England]	2015	invasive (unspecified/other)	Neisseria meningitidis	W	W	W		22	1	1	1	4	2	3	4	3	8	4	6	ST-11 complex	11	4	4	2
41548	M15 240984	UK [England]	2015	invasive (unspecified/other)	Neisseria meningitidis	B	B	B		13	3	1	145	3	17	5	19	17	3	25	86	ST-60 complex	11919	3	3	1
41549	M15 240985	UK [England]	2015	invasive (unspecified/other)	Neisseria meningitidis	W	W	W		22	1	1	1	4	2	3	4	3	8	4	6	ST-11 complex	11	4	4	2
41550	M15 240986	UK [England]	2015	invasive (unspecified/other)	Neisseria meningitidis	B	B	B		4	2	1	84	2	3	6	9	5	9	6	9	ST-41/44 complex	41	2	3	1
41551	M15 240987	UK [England]	2015	invasive (unspecified/other)	Neisseria meningitidis	Y	Y	Y		21	1	1	7	4	6	5	173	13	5	24	17	ST-174 complex	1466	4	4	2
41552	M15 240988	UK [England]	2015	invasive (unspecified/other)	Neisseria meningitidis	W	W	W		22	1	1	1	4	662	3	4	3	8	4	6	ST-11 complex	10651	4	4	2
41553	M15 240989	UK [England]	2015	invasive (unspecified/other)	Neisseria meningitidis	Y	Y	Y		25	1	1	26	4	12	5	18	9	11	9	17	ST-23 complex	1655	4	4	2
41554	M15 240990	UK [England]	2015	invasive (unspecified/other)	Neisseria meningitidis	B	B	B		4	2	1	84	2	3	6	9	5	8	6	9	ST-41/44 complex	485	2	3	1
41555	M15 240991	UK [England]	2015	invasive (unspecified/other)	Neisseria meningitidis	B	B	B		160	126	1	115	162	17	5	19	17	3	26	2	ST-60 complex	60	NA	NA	3
41556	M15 240992	UK [England]	2015	invasive (unspecified/other)	Neisseria meningitidis	Y	Y	Y		25	1	1	26	4	10	5	18	9	11	9	17	ST-23 complex	23	4	4	2
41561	M15 240993	UK	2015	invasive (unspecified/other)	Neisseria meningitidis	B	B	B		510	28	8	599	30	4	10	5	4	6	3	8	ST-32 complex	32	NA	NA	1
41557	M15 240994	UK [England]	2015	invasive (unspecified/other)	Neisseria meningitidis	Y	Y	Y		25	1	1	26	4	12	5	18	9	11	9	17	ST-23 complex	1655	4	4	2
41558	M15 240995	UK [England]	2015	invasive (unspecified/other)	Neisseria meningitidis	W	W	W		22	1	1	1	4	2	3	4	3	8	4	6	ST-11 complex	11	4	4	2
41559	M15 240996	UK [England]	2015	invasive (unspecified/other)	Neisseria meningitidis	B	B	B		4	2	1	647	2	3	6	9	5	8	6	9	ST-41/44 complex	485	2	3	1
29860	M02 240005	UK	2002	invasive (unspecified/other)	Neisseria meningitidis	C	C	C		786	5	1	326	11	2	3	4	3	8	4	6	ST-11 complex	11	11	2	1
29682	M02 240026	UK	2002	invasive (unspecified/other)	Neisseria meningitidis	W	W	W		22	1	4	1	4	11	5	18	8	11	24	21	ST-22 complex	22	4	4	2
30046	M02 240038	UK	2002	invasive (unspecified/other)	Neisseria meningitidis	B	B	B		259	5	1	215	11	150	3	4	3	8	4	6	ST-11 complex	1789	11	2	1
38136	M02 240039	UK	2002	invasive (unspecified/other)	Neisseria meningitidis	B	B	B		13	3	57	145	3	4	10	6	5	38	11	9	ST-269 complex	1991	3	3	1
29683	M02 240124	UK	2002	invasive (unspecified/other)	Neisseria meningitidis	W	W	W		9	1	1	187	47	2	3	4	3	8	4	6	ST-11 complex	11	NA	NA	1
29861	M02 240131	UK	2002	invasive (unspecified/other)	Neisseria meningitidis	C	C	C		95	5	1	32	11	2	3	4	3	8	4	6	ST-11 complex	11	11	2	2
29865	M02 240206	UK	2002	invasive (unspecified/other)	Neisseria meningitidis	C	C	C		22	1	1	1	4	2	3	4	3	8	4	6	ST-11 complex	11	4	4	2
38137	M02 240210	UK	2002	invasive (unspecified/other)	Neisseria meningitidis	B	B	B		15	4	2	193	1	4	10	15	9	8	5	9	ST-269 complex	1195	1	1	1
29686	M02 240227	UK	2002	invasive (unspecified/other)	Neisseria meningitidis	W	W	W		9	1	1	187	47	2	3	4	3	8	4	6	ST-11 complex	11	NA	NA	1
29687	M02 240235	UK	2002	invasive (unspecified/other)	Neisseria meningitidis	W		W		16	1	1	60	4	11	5	18	15	11	24	21	ST-22 complex	1158	4	4	2
29864	M02 240281	UK	2002	invasive (unspecified/other)	Neisseria meningitidis	C	C	C		45	8	77	87	20	2	3	4	3	8	4	6	ST-11 complex	11	20	4	3
29862	M02 240335	UK	2002	invasive (unspecified/other)	Neisseria meningitidis	C	C	C	NA		5	1	917	11	2	3	4	3	8	4	6	ST-11 complex	11	11	2	NA
29688	M02 240422	UK	2002	invasive (unspecified/other)	Neisseria meningitidis	W	W	W		9	1	1	187	47	2	3	4	3	8	4	6	ST-11 complex	11	NA	NA	1
29689	M02 240440	UK	2002	invasive (unspecified/other)	Neisseria meningitidis	W		W		9	1	1	187	47	2	3	4	3	8	4	6	ST-11 complex	11	NA	NA	1
29866	M02 240525	UK	2002	invasive (unspecified/other)	Neisseria meningitidis	C	C	C		259	5	1	215	11	2	3	4	3	8	4	6	ST-11 complex	11	11	2	1
29690	M02 240609	UK	2002	invasive (unspecified/other)	Neisseria meningitidis	W	W	W		9	1	1	187	47	2	3	4	3	8	4	6	ST-11 complex	11	NA	NA	1
30172	M02 240630	UK	2002	invasive (unspecified/other)	Neisseria meningitidis	B	B	B		59	9	1	75	16	43	5	9	60	11	19	15		1867	16	2	3
30047	M02 240690	UK	2002	invasive (unspecified/other)	Neisseria meningitidis	B	B	B		89	5	1	213	23	2	3	4	3	8	4	6	ST-11 complex	11	NA	NA	1
29692	M02 240839	UK	2002	invasive (unspecified/other)	Neisseria meningitidis	W	W	W		9	1	NA	187	47	2	3	4	3	8	4	6	ST-11 complex	11	NA	NA	1
29867	M02 240843	UK	2002	invasive (unspecified/other)	Neisseria meningitidis	C	C	C		1049	5	92	354	11	2	3	4	3	8	4	6	ST-11 complex	11	11	2	1
29868	M02 240945	UK	2002	invasive (unspecified/other)	Neisseria meningitidis	C	C	C		780	5	1	327	11	2	3	4	3	8	4	6	ST-11 complex	11	11	2	1
29869	M02 240972	UK	2002	invasive (unspecified/other)	Neisseria meningitidis	C	C	C		87	4	1	355	1	2	3	4	3	8	4	6	ST-11 complex	11	1	1	1
30048	M02 241095	UK	2002	invasive (unspecified/other)	Neisseria meningitidis	B	B	B		10	5	1	199	11	2	3	4	3	8	4	6	ST-11 complex	11	11	2	1
29870	M02 241124	UK	2002	invasive (unspecified/other)	Neisseria meningitidis	C	C	C		786	5	1	326	11	2	3	4	3	8	4	6	ST-11 complex	11	11	2	1
29694	M02 241321	UK	2002	invasive (unspecified/other)	Neisseria meningitidis	W	W	W		9	1	1	187	47	2	3	4	3	8	4	6	ST-11 complex	11	NA	NA	1
29695	M02 241603	UK	2002	invasive (unspecified/other)	Neisseria meningitidis	W	W	W		9	1	1	187	47	2	3	4	3	8	4	6	ST-11 complex	11	NA	NA	1
29871	M02 241652	UK	2002	invasive (unspecified/other)	Neisseria meningitidis	C	C	C		4	2	1	84	2	2	3	4	3	8	4	6	ST-11 complex	11	2	3	1
29696	M02 241666	UK	2002	invasive (unspecified/other)	Neisseria meningitidis	W	W	W		9	1	1	187	47	2	3	4	3	8	4	6	ST-11 complex	11	NA	NA	1
30049	M02 241729	UK	2002	invasive (unspecified/other)	Neisseria meningitidis	B	B	B		812	5	1	607	11	2	3	4	3	8	4	6	ST-11 complex	11	11	2	1
29697	M02 241746	UK	2002	invasive (unspecified/other)	Neisseria meningitidis	W	W	W		9	1	1	187	47	2	3	4	3	8	4	6	ST-11 complex	11	NA	NA	1
29872	M02 241949	UK	2002	invasive (unspecified/other)	Neisseria meningitidis	C	C	C		259	5	1	215	11	2	3	4	3	8	4	6	ST-11 complex	11	11	2	1
29698	M02 242123	UK	2002	invasive (unspecified/other)	Neisseria meningitidis	W		W		16	1	1	60	4	11	5	18	8	11	24	21	ST-22 complex	22	4	4	2
29873	M02 242246	UK	2002	invasive (unspecified/other)	Neisseria meningitidis	C	C	C		187	5	1	101	11	2	3	4	3	8	4	6	ST-11 complex	11	11	2	3
29971	M03 240109	UK	2003	invasive (unspecified/other)	Neisseria meningitidis	C	C	C		14	5	1	293	170	2	3	4	3	8	4	6	ST-11 complex	11	NA	NA	1
38138	M03 240151	UK	2003	invasive (unspecified/other)	Neisseria meningitidis	B	B	B		19	1	1	83	4	4	10	2	5	38	11	9	ST-269 complex	275	4	4	2
29875	M03 240453	UK	2003	invasive (unspecified/other)	Neisseria meningitidis	C	C	C		118	5	1	73	11	2	3	4	3	8	4	6	ST-11 complex	11	11	2	2
29972	M03 240483	UK	2003	invasive (unspecified/other)	Neisseria meningitidis	C	C	C		13	5	1	220	11	2	3	4	3	8	4	6	ST-11 complex	11	11	2	1
29973	M03 240588	UK	2003	invasive (unspecified/other)	Neisseria meningitidis	C	C	C		37	5	1	428	11	2	3	4	3	8	4	6	ST-11 complex	11	11	2	1
30224	M03 240641	UK	2003	invasive (unspecified/other)	Neisseria meningitidis	C	C	C		4	2	1	84	2	2	625	4	3	8	4	6	ST-11 complex	13244	2	3	1
29974	M03 240642	UK	2003	invasive (unspecified/other)	Neisseria meningitidis	C	C	C		11	5	1	201	11	2	3	4	3	8	4	6	ST-11 complex	11	11	2	1
29874	M03 240651	UK	2003	invasive (unspecified/other)	Neisseria meningitidis	C	C	C		259	5	1	215	11	2	3	4	3	8	4	6	ST-11 complex	11	11	2	1
29699	M03 240692	UK	2003	invasive (unspecified/other)	Neisseria meningitidis	W	W	W		9	1	1	187	47	2	3	4	3	8	4	6	ST-11 complex	11	NA	NA	1
29700	M03 240696	UK	2003	invasive (unspecified/other)	Neisseria meningitidis	W	W	W		9	1	1	187	47	2	3	4	3	8	4	6	ST-11 complex	11	NA	NA	1
30050	M03 240750	UK	2003	invasive (unspecified/other)	Neisseria meningitidis	B	B	B		10	5	1	199	11	2	3	4	3	8	4	6	ST-11 complex	11	11	2	1
38139	M03 240774	UK	2003	invasive (unspecified/other)	Neisseria meningitidis	B	B	B		84	87	1	70	141	12	6	9	9	9	200	9	ST-41/44 complex	2691	NA	NA	3
30225	M03 240792	UK	2003	invasive (unspecified/other)	Neisseria meningitidis	C	C	C		15	4	2	193	1	4	10	15	9	8	11	13	ST-269 complex	467	1	1	1
38140	M03 240823	UK	2003	invasive (unspecified/other)	Neisseria meningitidis	B	B	B		15	4	2	193	1	4	6	15	9	8	11	9	ST-269 complex	1284	1	1	1
29975	M03 240862	UK	2003	invasive (unspecified/other)	Neisseria meningitidis	C	C	C		259	5	1	215	11	2	3	4	NA	8	4	6		NA	11	2	1
29876	M03 240982	UK	2003	invasive (unspecified/other)	Neisseria meningitidis	C	C	C		14	5	1	293	170	2	3	4	3	8	4	6	ST-11 complex	11	NA	NA	1
29976	M03 241118	UK	2003	invasive (unspecified/other)	Neisseria meningitidis	C	C	C		776	5	1	NA	11	2	3	4	3	8	6	6	ST-11 complex	1055	11	2	NA
30226	M03 241190	UK	2003	invasive (unspecified/other)	Neisseria meningitidis	C	C	C		275	171	1	299	NA	2	3	4	3	8	4	6	ST-11 complex	11	NA	NA	1
29977	M03 241273	UK	2003	invasive (unspecified/other)	Neisseria meningitidis	C	C	C		10	5	1	199	11	2	3	4	3	8	4	6	ST-11 complex	11	11	2	1
30227	M03 241322	UK	2003	invasive (unspecified/other)	Neisseria meningitidis	C	C	C		1106	5	1	608	11	2	3	4	3	8	4	6	ST-11 complex	11	11	2	1
29978	M03 241465	UK	2003	invasive (unspecified/other)	Neisseria meningitidis	C	C	C		15	4	1	193	1	2	3	4	3	8	4	6	ST-11 complex	11	1	1	1
30229	M03 241630	UK	2003	invasive (unspecified/other)	Neisseria meningitidis	C	C	C		1043	5	1	366	11	2	3	4	3	8	4	6	ST-11 complex	11	11	2	1
30051	M03 241710	UK	2003	invasive (unspecified/other)	Neisseria meningitidis	B	B	B		14	5	1	293	170	2	3	4	3	8	4	6	ST-11 complex	11	NA	NA	1
29979	M03 241792	UK	2003	invasive (unspecified/other)	Neisseria meningitidis	C	C	C		4	2	1	84	2	2	3	4	3	8	4	6	ST-11 complex	11	2	3	1
29572	M04 240003	UK	2004	invasive (unspecified/other)	Neisseria meningitidis	C	C	C		11	5	1	201	11	2	3	4	3	8	4	6	ST-11 complex	11	11	2	1
29573	M04 240019	UK	2004	invasive (unspecified/other)	Neisseria meningitidis	C	C	C		10	5	1	199	11	672	3	4	3	8	4	6	ST-11 complex	11149	11	2	1
29574	M04 240020	UK	2004	invasive (unspecified/other)	Neisseria meningitidis	C	C	C		10	5	1	199	11	2	3	4	3	8	4	6	ST-11 complex	11	11	2	1
29575	M04 240029	UK	2004	invasive (unspecified/other)	Neisseria meningitidis	C	C	C		129	5	1	197	11	2	3	4	3	8	4	6	ST-11 complex	11	11	2	1
29576	M04 240065	UK	2004	invasive (unspecified/other)	Neisseria meningitidis	C	C	C		37	5	1	428	11	2	3	4	3	8	4	6	ST-11 complex	11	11	2	1
29577	M04 240078	UK	2004	invasive (unspecified/other)	Neisseria meningitidis	C	C	C	NA		5	1	917	11	2	3	4	3	8	4	6	ST-11 complex	11	11	2	NA
29578	M04 240129	UK	2004	invasive (unspecified/other)	Neisseria meningitidis	C	C	C		22	1	1	1	4	2	3	4	3	8	4	6	ST-11 complex	11	4	4	2
29580	M04 240235	UK	2004	invasive (unspecified/other)	Neisseria meningitidis	C	C	C		10	5	1	199	11	2	3	4	3	8	4	6	ST-11 complex	11	11	2	1
30052	M04 240245	UK	2004	invasive (unspecified/other)	Neisseria meningitidis	B	B	B		10	5	1	199	11	2	3	4	3	8	4	6	ST-11 complex	11	11	2	1
29581	M04 240259	UK	2004	invasive (unspecified/other)	Neisseria meningitidis	C	C	C		10	5	1	199	11	2	3	4	3	8	4	6	ST-11 complex	11	11	2	1
29582	M04 240323	UK	2004	invasive (unspecified/other)	Neisseria meningitidis	C	C	C		10	5	1	199	11	2	3	4	3	8	4	6	ST-11 complex	11	11	2	1
29583	M04 240335	UK	2004	invasive (unspecified/other)	Neisseria meningitidis	C	C	C		259	5	1	215	11	2	3	4	3	8	4	6	ST-11 complex	11	11	2	1
29584	M04 240348	UK	2004	invasive (unspecified/other)	Neisseria meningitidis	C	C	C		13	5	1	220	11	2	3	4	3	8	4	6	ST-11 complex	11	11	2	1
29702	M04 240429	UK	2004	invasive (unspecified/other)	Neisseria meningitidis	W	W	W		9	23	1	187	203	2	3	4	3	8	4	6	ST-11 complex	11	NA	NA	1
38141	M04 240588	UK	2004	invasive (unspecified/other)	Neisseria meningitidis	B	B	B		45	8	77	87	20	7	5	1	13	9	53	15	ST-213 complex	3635	20	4	3
29585	M04 240594	UK	2004	invasive (unspecified/other)	Neisseria meningitidis	C	C	C		259	5	1	215	11	2	3	4	3	8	4	6	ST-11 complex	11	11	2	1
38142	M04 240731	UK	2004	invasive (unspecified/other)	Neisseria meningitidis	B	B	B		123	1	1	382	13	3	6	19	5	3	6	9	ST-41/44 complex	340	13	3	1
29586	M04 240752	UK	2004	invasive (unspecified/other)	Neisseria meningitidis	C	C	C		259	5	1	215	11	2	3	4	3	8	4	6	ST-11 complex	11	11	2	1
29587	M04 240931	UK	2004	invasive (unspecified/other)	Neisseria meningitidis	C	C	C		778	5	1	225	11	2	3	4	3	8	4	6	ST-11 complex	11	11	2	1
29571	M04 240932	UK	2004	invasive (unspecified/other)	Neisseria meningitidis	C	C	C		118	5	1	73	11	2	3	4	3	8	4	6	ST-11 complex	11	11	2	2
30053	M04 241052	UK	2004	invasive (unspecified/other)	Neisseria meningitidis	B	B	B		10	5	1	199	11	2	3	4	3	8	4	6	ST-11 complex	11	11	2	1
29589	M04 241156	UK	2004	invasive (unspecified/other)	Neisseria meningitidis	C	C	C	NA		5	1	917	11	2	3	4	3	8	4	6	ST-11 complex	11	11	2	NA
29590	M04 241209	UK	2004	invasive (unspecified/other)	Neisseria meningitidis	C	C	C		118	5	1	73	11	2	3	4	3	8	4	6	ST-11 complex	11	11	2	2
38143	M04 241215	UK	2004	invasive (unspecified/other)	Neisseria meningitidis	B	B	B		4	2	1	84	2	3	6	9	5	9	6	9	ST-41/44 complex	41	2	3	1
38144	M04 241245	UK	2004	invasive (unspecified/other)	Neisseria meningitidis	B	B	B		19	1	1	65	4	8	26	9	24	26	20	18	ST-212 complex	212	4	4	2
29591	M04 241276	UK	2004	invasive (unspecified/other)	Neisseria meningitidis	C	C	C		269	5	1	227	11	2	3	4	923	8	4	6	ST-11 complex	13253	11	2	1
30231	M04 241279	UK	2004	invasive (unspecified/other)	Neisseria meningitidis	C	C	C		15	4	2	193	1	4	10	15	9	8	11	6	ST-269 complex	1095	1	1	1
29592	M04 241501	UK	2004	invasive (unspecified/other)	Neisseria meningitidis	C	C	C		95	5	1	32	11	2	3	4	3	8	4	6	ST-11 complex	11	11	2	2
29593	M04 241542	UK	2004	invasive (unspecified/other)	Neisseria meningitidis	C	C	C		770	5	1	241	201	2	3	4	3	8	25	22	ST-11 complex	11157	NA	NA	1
29594	M04 241591	UK	2004	invasive (unspecified/other)	Neisseria meningitidis	C	C	C		259	5	1	215	11	2	3	4	3	8	4	6	ST-11 complex	11	11	2	1
29595	M04 241722	UK	2004	invasive (unspecified/other)	Neisseria meningitidis	C	C	C		11	5	1	201	11	2	3	4	3	8	4	6	ST-11 complex	11	11	2	1
29596	M05 240080	UK	2005	invasive (unspecified/other)	Neisseria meningitidis	C	C	C		22	1	1	1	4	2	3	4	3	8	4	6	ST-11 complex	11	4	4	2
29597	M05 240211	UK	2005	invasive (unspecified/other)	Neisseria meningitidis	C	C	C		15	4	121	193	1	2	3	4	3	8	4	6	ST-11 complex	11	1	1	1
38145	M05 240300	UK	2005	invasive (unspecified/other)	Neisseria meningitidis	B	B	B		45	61	77	87	187	7	2	6	13	82	53	15	ST-213 complex	7299	NA	NA	3
29599	M05 240314	UK	2005	invasive (unspecified/other)	Neisseria meningitidis	C	C	C		11	5	1	201	11	2	3	4	3	8	4	6	ST-11 complex	11	11	2	1
29600	M05 240316	UK	2005	invasive (unspecified/other)	Neisseria meningitidis	C	C	C		129	5	1	250	11	2	3	4	3	8	4	6	ST-11 complex	11	11	2	1
29601	M05 240490	UK	2005	invasive (unspecified/other)	Neisseria meningitidis	C	C	C		13	5	1	263	11	2	3	4	3	8	4	6	ST-11 complex	11	11	2	1
30054	M05 240518	UK	2005	invasive (unspecified/other)	Neisseria meningitidis	B	B	B		813	5	1	364	11	2	3	4	3	8	4	6	ST-11 complex	11	11	2	1
29602	M05 240553	UK	2005	invasive (unspecified/other)	Neisseria meningitidis	C	C	C		11	5	1	201	11	2	3	4	3	8	4	6	ST-11 complex	11	11	2	1
29604	M05 240747	UK	2005	invasive (unspecified/other)	Neisseria meningitidis	C	C	C		10	5	1	199	11	2	3	4	3	8	4	6	ST-11 complex	11	11	2	1
29606	M05 240972	UK	2005	invasive (unspecified/other)	Neisseria meningitidis	C	C	C	NA		5	1	917	11	2	3	4	3	8	4	6	ST-11 complex	11	11	2	NA
29704	M05 241067	UK	2005	invasive (unspecified/other)	Neisseria meningitidis	W	W	W		151	1	1	130	4	2	3	4	3	8	4	6	ST-11 complex	11	4	4	2

29608	M05 241164	UK	2005	invasive (unspecified/other)	Neisseria meningitidis	C	C	C	NA	5	1	NA	11	2	3	4	3	8	4	6	ST-11 complex	11	11	2	NA
29610	M05 241361	UK	2005	invasive (unspecified/other)	Neisseria meningitidis	C	C	C	22	1	1	1	4	2	3	4	3	8	4	6	ST-11 complex	11	4	4	2
29611	M06 240019	UK	2006	invasive (unspecified/other)	Neisseria meningitidis	C	C	C	22	1	1	1	4	2	3	4	3	8	4	6	ST-11 complex	11	4	4	2
29612	M06 240067	UK	2006	invasive (unspecified/other)	Neisseria meningitidis	C	C	C	22	1	1	1	4	2	3	4	3	8	4	6	ST-11 complex	11	4	4	2
29613	M06 240167	UK	2006	invasive (unspecified/other)	Neisseria meningitidis	C	C	C	NA	5	1	917	11	2	3	4	3	8	4	6	ST-11 complex	11	11	2	NA
29624	M06 240168	UK	2006	invasive (unspecified/other)	Neisseria meningitidis	C	C	C	252	5	1	274	11	2	3	4	3	8	4	6	ST-11 complex	11	11	2	1
29615	M06 240183	UK	2006	invasive (unspecified/other)	Neisseria meningitidis	C	C	C	118	5	1	73	11	2	3	4	3	8	4	6	ST-11 complex	11	11	2	2
29617	M06 240375	UK	2006	invasive (unspecified/other)	Neisseria meningitidis	C	C	C	107	5	1	NA	11	2	3	4	3	8	4	6	ST-11 complex	11	11	2	1
29705	M06 240459	UK	2006	invasive (unspecified/other)	Neisseria meningitidis	W	W	W	22	1	1	1	4	2	3	4	3	8	4	6	ST-11 complex	11	4	4	2
29619	M06 240536	UK	2006	invasive (unspecified/other)	Neisseria meningitidis	C	C	C	10	5	1	199	11	2	3	4	3	8	4	6	ST-11 complex	11	11	2	1
29620	M06 240537	UK	2006	invasive (unspecified/other)	Neisseria meningitidis	C	C	C	45	5	1	157	11	2	3	4	3	8	4	6	ST-11 complex	11	11	2	3
29621	M06 240625	UK	2006	invasive (unspecified/other)	Neisseria meningitidis	C	C	C	760	5	1	273	11	2	3	4	3	8	4	6	ST-11 complex	11	11	2	1
29622	M06 240671	UK	2006	invasive (unspecified/other)	Neisseria meningitidis	C	C	C	328	5	1	351	11	2	3	4	3	8	4	6	ST-11 complex	11	11	2	1
30055	M06 240679	UK	2006	invasive (unspecified/other)	Neisseria meningitidis	B	B	B	299	5	1	321	11	2	3	4	NA	8	4	6		NA	11	2	1
29614	M06 240854	UK	2006	invasive (unspecified/other)	Neisseria meningitidis	C	C	C	760	5	1	273	11	2	3	4	3	8	4	6	ST-11 complex	11	11	2	1
38146	M06 240928	UK	2006	invasive (unspecified/other)	Neisseria meningitidis	B	B	B	19	1	2	65	4	6	10	15	9	8	874	9	ST-269 complex	13323	4	4	2
29623	M06 241012	UK	2006	invasive (unspecified/other)	Neisseria meningitidis	C	C	C	NA	5	1	917	11	2	3	4	3	8	4	6	ST-11 complex	11	11	2	NA
38147	M06 241112	UK	2006	invasive (unspecified/other)	Neisseria meningitidis	B	B	B	15	4	2	193	1	4	10	15	9	8	11	9	ST-269 complex	269	1	1	1
29706	M07 240001	UK	2007	invasive (unspecified/other)	Neisseria meningitidis	W	W	W	16	1	1	60	4	420	5	173	90	459	24	21		10683	4	4	2
29625	M07 240006	UK	2007	invasive (unspecified/other)	Neisseria meningitidis	C	C	C	768	1	1	275	4	2	3	4	3	8	4	6	ST-11 complex	11	4	3	1
29707	M07 240118	UK	2007	invasive (unspecified/other)	Neisseria meningitidis	W	W	W	9	1	1	187	47	2	3	4	3	8	4	6	ST-11 complex	11	NA	NA	1
29632	M07 240172	UK	2007	invasive (unspecified/other)	Neisseria meningitidis	C	C	C	1040	5	1	352	11	2	3	4	3	8	4	6	ST-11 complex	11	11	2	1
29633	M07 240210	UK	2007	invasive (unspecified/other)	Neisseria meningitidis	C	C	C	165	164	100	158	202	2	3	4	3	8	356	6	ST-11 complex	5149	NA	NA	3
30056	M07 240239	UK	2007	invasive (unspecified/other)	Neisseria meningitidis	B	B	B	814	5	1	365	11	2	3	4	3	8	4	6	ST-11 complex	11	11	2	3
29634	M07 240276	UK	2007	invasive (unspecified/other)	Neisseria meningitidis	C	C	C	NA	5	1	917	11	2	3	4	3	8	4	5	ST-11 complex	11158	11	2	NA
29635	M07 240351	UK	2007	invasive (unspecified/other)	Neisseria meningitidis	C	C	C	779	5	28	320	11	2	3	4	3	8	4	6	ST-11 complex	11	11	2	1
29711	M07 240489	UK	2007	invasive (unspecified/other)	Neisseria meningitidis	W	W	W	16	1	1	60	4	11	5	18	8	11	24	21	ST-22 complex	22	4	4	2
29636	M07 240541	UK	2007	invasive (unspecified/other)	Neisseria meningitidis	C	C	C	11	5	1	201	11	2	3	4	3	8	4	6	ST-11 complex	11	11	2	1
29637	M07 240593	UK	2007	invasive (unspecified/other)	Neisseria meningitidis	C	C	C	11	5	1	201	11	2	3	4	3	8	4	6	ST-11 complex	11	11	2	1
39442	M07 240646	UK	2007	invasive (unspecified/other)	Neisseria meningitidis	B	B	B	19	1	1	83	4	4	10	2	5	38	11	9	ST-269 complex	275	4	4	2
39443	M07 240657	UK	2007	invasive (unspecified/other)	Neisseria meningitidis	B	B	B	294	196	105	555	160	7	5	1	13	36	53	15	ST-213 complex	213	NA	NA	3
39444	M07 240669	UK	2007	invasive (unspecified/other)	Neisseria meningitidis	B	B	B	19	1	1	65	4	9	20	9	9	9	6	2	ST-41/44 complex	1097	4	4	2
39445	M07 240680	UK	2007	invasive (unspecified/other)	Neisseria meningitidis	B	B	B	19	1	1	65	4	28	6	9	9	9	6	9	ST-41/44 complex	207	4	4	2
29626	M07 240686	UK	2007	invasive (unspecified/other)	Neisseria meningitidis	C	C	C	22	1	1	1	4	2	3	4	3	8	494	6	ST-11 complex	7779	4	4	2
29627	M07 240723	UK	2007	invasive (unspecified/other)	Neisseria meningitidis	C	C	C	306	5	1	217	11	2	3	4	3	8	4	6	ST-11 complex	11	11	2	1
29709	M07 240774	UK	2007	invasive (unspecified/other)	Neisseria meningitidis	W	W	W	22	1	1	1	4	2	3	4	5	8	4	6	ST-11 complex	247	4	4	2
39446	M07 240789	UK	2007	invasive (unspecified/other)	Neisseria meningitidis	B	B	B	202	1	1	35	4	42	26	46	24	2	20	17	ST-282 complex	282	4	4	2
35404	M07 240834	UK	2007	invasive (unspecified/other)	Neisseria meningitidis	B	B	B	15	4	2	193	1	4	10	15	9	8	11	9	ST-269 complex	269	1	1	1
35410	M07 240836	UK	2007	invasive (unspecified/other)	Neisseria meningitidis	B	B	B	13	3	57	145	3	4	10	34	0	38	11	9		NA	3	3	1
39447	M07 240850	UK	2007	invasive (unspecified/other)	Neisseria meningitidis	B	B	B	16	1	1	59	4	12	6	4	32	83	18	20		8006	4	4	2
39448	M07 240889	UK	2007	invasive (unspecified/other)	Neisseria meningitidis	B	B	B	19	1	1	83	4	4	10	2	5	38	11	9	ST-269 complex	275	4	4	2
35411	M07 240904	UK	2007	invasive (unspecified/other)	Neisseria meningitidis	B	B	B	13	3	57	145	3	4	10	34	5	38	11	9	ST-269 complex	1161	3	3	1
39449	M07 240909	UK	2007	invasive (unspecified/other)	Neisseria meningitidis	B	B	B	19	1	106	65	4	7	5	0	13	36	53	15		NA	4	4	2
29710	M07 240922	UK	2007	invasive (unspecified/other)	Neisseria meningitidis	W	W	W	37	1	1	408	4	2	3	4	5	8	4	6	ST-11 complex	247	4	3	1
29628	M07 240924	UK	2007	invasive (unspecified/other)	Neisseria meningitidis	C	C	C	10	5	1	199	11	2	3	4	3	9	4	6	ST-11 complex	491	11	2	1
39450	M07 240949	UK	2007	invasive (unspecified/other)	Neisseria meningitidis	B	B	B	19	1	1	65	4	9	20	9	9	9	6	2	ST-41/44 complex	1097	4	4	2
34540	M07 240954	UK	2007	invasive (unspecified/other)	Neisseria meningitidis	C	C	C	10	5	1	199	11	2	3	4	3	9	4	6	ST-11 complex	491	11	2	1
35412	M07 240980	UK	2007	invasive (unspecified/other)	Neisseria meningitidis	B	B	B	13	3	57	145	3	4	10	34	5	38	11	9	ST-269 complex	1161	3	3	1
35405	M07 241002	UK	2007	invasive (unspecified/other)	Neisseria meningitidis	B	B	B	15	4	2	193	1	4	10	15	9	8	11	9	ST-269 complex	269	1	1	1
39451	M07 241052	UK	2007	invasive (unspecified/other)	Neisseria meningitidis	B	B	B	19	1	1	83	4	4	5	2	5	38	11	1		1879	4	4	2
39452	M07 241084	UK	2007	invasive (unspecified/other)	Neisseria meningitidis	B	B	B	302	24	1	121	55	9	20	9	9	9	6	2	ST-41/44 complex	1097	NA	NA	2
29631	M07 241093	UK	2007	invasive (unspecified/other)	Neisseria meningitidis	C	C	C	NA	5	1	917	11	2	3	4	3	8	4	6	ST-11 complex	11	11	2	NA
39453	M07 241109	UK	2007	invasive (unspecified/other)	Neisseria meningitidis	B	B	B	34	41	1	3	51	13	3	16	1	3	11	46	ST-364 complex	7743	NA	NA	2
39454	M07 241117	UK	2007	invasive (unspecified/other)	Neisseria meningitidis	B	B	B	19	1	1	83	4	4	10	2	5	38	11	9	ST-269 complex	275	4	4	2
35406	M07 241143	UK	2007	invasive (unspecified/other)	Neisseria meningitidis	B	B	B	15	4	2	193	1	4	10	15	9	8	11	9	ST-269 complex	269	1	1	1
35413	M08 240017	UK	2008	invasive (unspecified/other)	Neisseria meningitidis	B	B	B	13	3	57	145	3	4	10	34	5	38	11	9	ST-269 complex	1161	3	3	1
29638	M08 240026	UK	2008	invasive (unspecified/other)	Neisseria meningitidis	C	C	C	22	1	1	1	4	2	3	4	3	8	4	6	ST-11 complex	11	4	4	2
30057	M08 240033	UK	2008	invasive (unspecified/other)	Neisseria meningitidis	B	B	B	25	1	1	26	4	15	3	133	13	8	19	15		1162	4	4	2
39455	M08 240035	UK	2008	invasive (unspecified/other)	Neisseria meningitidis	B	B	B	19	1	1	83	4	4	10	2	5	38	11	9	ST-269 complex	275	4	4	2
35414	M08 240056	UK	2008	invasive (unspecified/other)	Neisseria meningitidis	B	B	B	13	3	57	145	3	4	10	34	5	38	11	9	ST-269 complex	1161	3	3	1
29879	M08 240142	UK	2008	invasive (unspecified/other)	Neisseria meningitidis	C	C	C	303	5	1	184	11	2	3	4	3	8	4	6	ST-11 complex	11	11	2	1
39456	M08 240169	UK	2008	invasive (unspecified/other)	Neisseria meningitidis	NG	B	B	16	56	79	60	NA	2	5	2	9	17	65	112	ST-750 complex	11394	NA	NA	2
35407	M08 240183	UK	2008	invasive (unspecified/other)	Neisseria meningitidis	B	B	B	15	4	2	193	1	4	10	15	9	8	11	9	ST-269 complex	269	1	1	1
29639	M08 240185	UK	2008	invasive (unspecified/other)	Neisseria meningitidis	C	C	C	306	5	1	217	11	2	3	4	3	8	4	6	ST-11 complex	11	11	2	1
29640	M08 240201	UK	2008	invasive (unspecified/other)	Neisseria meningitidis	C	C	C	10	5	1	199	11	2	3	4	3	8	4	6	ST-11 complex	11	11	2	1
39457	M08 240220	UK	2008	invasive (unspecified/other)	Neisseria meningitidis	B	B	B	19	1	1	65	4	9	6	9	9	9	6	2	ST-41/44 complex	180	4	4	2
39458	M08 240222	UK	2008	invasive (unspecified/other)	Neisseria meningitidis	B	B	B	19	1	1	65	4	9	6	9	9	9	6	2	ST-41/44 complex	180	4	4	2
30058	M08 240227	UK	2008	invasive (unspecified/other)	Neisseria meningitidis	B	B	B	23	5	1	19	11	2	3	4	3	8	4	6	ST-11 complex	11	11	2	2
29641	M08 240231	UK	2008	invasive (unspecified/other)	Neisseria meningitidis	C	C	C	16	5	1	159	11	2	3	4	3	8	500	21	ST-11 complex	7979	11	2	2
35415	M08 240234	UK	2008	invasive (unspecified/other)	Neisseria meningitidis	B	B	B	13	3	57	145	3	4	10	34	5	38	11	9	ST-269 complex	1161	3	3	1
39459	M08 240255	UK	2008	invasive (unspecified/other)	Neisseria meningitidis	B	B	B	19	1	1	65	4	9	20	9	9	9	6	2	ST-41/44 complex	1097	4	4	2
29642	M08 240270	UK	2008	invasive (unspecified/other)	Neisseria meningitidis	C	C	C	NA	5	1	917	11	NA	3	4	3	8	4	6		NA	11	2	NA
39460	M08 240276	UK	2008	invasive (unspecified/other)	Neisseria meningitidis	B	B	B	45	8	77	87	20	7	5	1	13	36	53	15	ST-213 complex	213	20	4	3
29878	M08 240284	UK	2008	invasive (unspecified/other)	Neisseria meningitidis	C	C	C	10	5	1	199	11	2	3	4	3	9	4	6	ST-11 complex	491	11	2	1
39461	M08 240293	UK	2008	invasive (unspecified/other)	Neisseria meningitidis	B	B	B	16	1	4	59	4	4	10	2	5	38	11	9	ST-269 complex	275	4	4	2
39462	M08 240302	UK	2008	invasive (unspecified/other)	Neisseria meningitidis	B	B	B	102	8	1	2	20	4	10	2	5	38	11	9	ST-269 complex	275	20	4	2
35409	M08 240363	UK	2008	invasive (unspecified/other)	Neisseria meningitidis	B	B	B	15	4	2	193	1	4	10	15	NA	8	11	9		NA	1	1	1
29882	M08 240409	UK	2008	invasive (unspecified/other)	Neisseria meningitidis	C	C	C	NA	5	1	917	11	2	3	4	3	8	4	6	ST-11 complex	11	11	2	NA
35408	M08 240430	UK	2008	invasive (unspecified/other)	Neisseria meningitidis	B	B	B	15	4	2	193	1	4	10	15	9	8	11	17	ST-269 complex	283	1	1	1
29880	M08 240459	UK	2008	invasive (unspecified/other)	Neisseria meningitidis	C	C	C	10	5	1	199	11	2	3	4	3	9	4	6	ST-11 complex	491	11	2	1
39463	M08 240498	UK	2008	invasive (unspecified/other)	Neisseria meningitidis	B	B	B	84	13	1	70	22	42	26	46	24	5	20	17	ST-282 complex	8068	NA	NA	3
29881	M08 240682	UK	2008	invasive (unspecified/other)	Neisseria meningitidis	C	C	C	10	5	1	199	11	2	3	4	NA	9	4	6		NA	11	2	1
29887	M08 240767	UK	2008	invasive (unspecified/other)	Neisseria meningitidis	C	C	C	10	5	1	199	11	2	3	4	3	8	4	6	ST-11 complex	11	11	2	1
30059	M08 240819	UK	2008	invasive (unspecified/other)	Neisseria meningitidis	B	B	B	128	5	1	NA	11	2	3	4	3	8	4	6	ST-11 complex	11	11	2	1
29883	M08 240875	UK	2008	invasive (unspecified/other)	Neisseria meningitidis	C	C	C	10	5	1	199	11	2	3	4	3	9	4	6	ST-11 complex	491	11	2	1
29886	M08 240954	UK	2008	invasive (unspecified/other)	Neisseria meningitidis	C	C	C	760	5	1	273	11	2	3	4	3	8	4	6	ST-11 complex	11	11	2	1
30060	M09 240026	UK	2009	invasive (unspecified/other)	Neisseria meningitidis	B	B	B	775	5	1	294	11	2	3	4	3	8	4	6	ST-11 complex	11	11	2	1
20738	M09 240039	UK	2009	invasive (unspecified/other)	Neisseria meningitidis		B	B	4	2	1	84	2	3	6	34	5	11	6	9	ST-41/44 complex	1194	2	3	1
20742	M09 240078	UK	2009	invasive (unspecified/other)	Neisseria meningitidis		B	B	4	2	79	84	2	3	6	9	5	9	6	9	ST-41/44 complex	41	2	3	1
29884	M09 240132	UK	2009	invasive (unspecified/other)	Neisseria meningitidis	C	C	C	499	5	1	356	11	2	3	4	3	8	4	6	ST-11 complex	11	11	2	3
20748	M09 240139	UK	2009	invasive (unspecified/other)	Neisseria meningitidis		B	B	4	2	79	84	2	3	6	9	5	9	6	9	ST-41/44 complex	41	2	3	1
29885	M09 240171	UK	2009	invasive (unspecified/other)	Neisseria meningitidis	C	C	C	1041	5	1	357	11	2	3	4	3	8	4	6	ST-11 complex	11	11	2	1
20805	M09 240174	UK	2009	invasive (unspecified/other)	Neisseria meningitidis		B	B	14	5	1	204	11	3	6	19	201	3	6	9	ST-41/44 complex	6761	11	2	1
29888	M09 240249	UK	2009	invasive (unspecified/other)	Neisseria meningitidis	C	C	C	NA	5	1	NA	11	2	3	4	3	8	4	6	ST-11 complex	11	11	2	NA
20755	M09 240256	UK	2009	invasive (unspecified/other)	Neisseria meningitidis		B	B	4	2	79	84	2	3	6	9	5	9	6	9	ST-41/44 complex	41	2	3	1
20757	M09 240281	UK	2009	invasive (unspecified/other)	Neisseria meningitidis		B	B	4	2	1	84	2	3	6	9	15	11	6	9	ST-41/44 complex	4100	2	3	1
20758	M09 240283	UK	2009	invasive (unspecified/other)	Neisseria meningitidis		B	B	14	1	1	142	13	9	6	9	5	9	6	9	ST-41/44 complex	318	13	3	1
20760	M09 240330	UK	2009	invasive (unspecified/other)	Neisseria meningitidis		B	B	4	2	1	84	2	3	6	79	5	11	6	9	ST-41/44 complex	9160	2	3	1
29712	M09 240368	UK	2009	invasive (unspecified/other)	Neisseria meningitidis	W	W	W	16	1	1	60	4	420	5	173	90	11	24	21	ST-22 complex	11160	4	4	2
20762	M09 240439	UK	2009	invasive (unspecified/other)	Neisseria meningitidis		B	B	4	2	1	84	2	3	6	9	5	9	6	9	ST-41/44 complex	41	2	3	1
20764	M09 240463	UK	2009	invasive (unspecified/other)	Neisseria meningitidis		B	B	14	1	1	142	13	3	6	19	5	3	6	9	ST-41/44 complex	340	13	3	1
20766	M09 240506	UK	2009	invasive (unspecified/other)	Neisseria meningitidis		B	B	19	1	2	65	4	6	6	63	9	9	11	9	ST-41/44 complex	839	4	4	2
20770	M09 240669	UK	2009	invasive (unspecified/other)	Neisseria meningitidis		B	B	4	2	1	84	2	3	6	9	5	9	6	9	ST-41/44 complex	41	2	3	1
29893	M09 240796	UK	2009	invasive (unspecified/other)	Neisseria meningitidis	C	C	C	NA	5	1	917	11	2	3	4	3	8	4	6	ST-11 complex	11	11	2	NA
29894	M09 240826	UK	2009	invasive (unspecified/other)	Neisseria meningitidis	C	C	C	259	5	1	215	11	2	3	4	3	8	4	6	ST-11 complex	11	11	2	1
20773	M09 240831	UK	2009	invasive (unspecified/other)	Neisseria meningitidis		B	B	4	2	1	84	2	3	10	34	5	11	6	9	ST-41/44 complex	9238	2	3	1
29715	M09 240862	UK	2009	invasive (unspecified/other)	Neisseria meningitidis	W	W	W	22	1	1	1	4	2	3	4	3	8	4	6	ST-11 complex	11	4	4	2
29716	M09 240900	UK	2009	invasive (unspecified/other)	Neisseria meningitidis	W	W	W	22	1	1	1	4	2	3	4	3	8	4	6	ST-11 complex	11	4	4	2
30234	M96 252316	UK	1996	invasive (unspecified/other)	Neisseria meningitidis	W		W	22	1	1	1	4	2	3	4	3	8	4	6	ST-11 complex	11	4	4	2
30232	M96 252550	UK	1996	invasive (unspecified/other)	Neisseria meningitidis	C	NG	discrepancy	13	5	1	251	11	2	3	4	3	8	4	6	ST-11 complex	11	11	2	1
30233	M96 252591	UK	1996	invasive (unspecified/other)	Neisseria meningitidis	C	C	C	95	5	1	32	11	2	3	4	3	8	4	71	ST-11 complex	4044	11	2	2
30235	M96 253521	UK	1996	invasive (unspecified/other)	Neisseria meningitidis	W	W	W	151	1	1	130	4	2	3	4	3	8	4	6	ST-11 complex	11	4	4	2
30236	M96 255203	UK	1996	invasive (unspecified/other)	Neisseria meningitidis	W	W	W	151	1	1	130	4	2	3	4	3	8	4	6	ST-11 complex	11	4	4	2
30237	M96 255688	UK	1996	invasive (unspecified/other)	Neisseria meningitidis	W	W	W	22	1	1	1	4	2	3	4	3	8	4	6	ST-11 complex	11	4	4	2
30185	M96 255770	UK	1996	invasive (unspecified/other)	Neisseria meningitidis	C	C	C	128	5	1	NA	11	2	3	4	3	8	4	6	ST-11 complex	11	11	2	1
30186	M96 255772	UK	1996	invasive (unspecified/other)	Neisseria meningitidis	C	C	C	128	5	1	NA	11	2	3	4	3	8	4	6	ST-11 complex	11	11	2	1
30187	M96 255773	UK	1996	invasive (unspecified/other)	Neisseria meningitidis	C	C	C	128	5	1	NA	11	2	3	4	3	8	4	6	ST-11 complex	11	11	2	1
30188	M96 255774	UK	1996	invasive (unspecified/other)	Neisseria meningitidis	C	C	C	128	5	1	NA	11	2	3	4	3	8	4	6	ST-11 complex	11	11	2	1
30189	M96 255775	UK	1996	invasive (unspecified/other)	Neisseria meningitidis	C	C	C	128	5	1	NA	11	2	3	4	3	8	4	6	ST-11 complex	11	11	2	1
30190	M96 255863	UK	1996	carrier	Neisseria meningitidis	C	C	C	NA	5	1	917	11	2	3	4	3	8	4	6	ST-11 complex	11	11	2	NA
30191	M96 255864	UK	1996	carrier	Neisseria meningitidis	C	C	C	NA	5	1	917	11	2	3	4	3	8	4	6	ST-11 complex	11	11	2	NA
30192	M96 255871	UK	1996	carrier	Neisseria meningitidis	C	C	C	201	1	1	23	4	2	3	4	3	8	4	6	ST-11 complex	11	4	4	2
30193	M96 255976	UK	1996	carrier	Neisseria meningitidis	C	C	C	201	1	1	23	4	2	3	4	3	8	4	6	ST-11 complex	11	4	4	2
30238	M96 256246	UK	1996	invasive (unspecified/other)	Neisseria meningitidis	W	W	W	151	1	NA	130	4	2	3	4	3	8	4	6	ST-11 complex	11	4	4	2
30174	M97 250455	UK	1997	invasive (unspecified/other)	Neisseria meningitidis	W	W	W	151	1	1	130	4	2	3	4	3	8	4	6	ST-11 complex	11	4	4	2
30175	M97 250650	UK	1997	invasive (unspecified/other)	Neisseria meningitidis	W	W	W	22	1	1	1	4	2	3	4	NA	8	4	6		NA	4	4	2
30176	M97 251635	UK	1997	invasive (unspecified/other)	Neisseria meningitidis	W	W	W	151	1	1	130	4	2	3	4	3	8	4	6	ST-11 complex	11	4	4	2
30177	M97 252137	UK	1997	invasive (unspecified/other)	Neisseria meningitidis	W	Y	discrepancy	260	1	4	298	4	25	7	6	17	NA	18	8		NA	4	3	1
29980	M97 253146	UK	1997	carrier	Neisseria meningitidis	B	B	B	462	5	1	325	11	2	3	4	NA	8	4	6		NA	11	2	1
30178	M98 250031	UK	1998	invasive (unspecified/other)	Neisseria meningitidis	W	W	W	22	1	1	1	4	2	3	4	15	8	4	6	ST-11 complex	1237	4	4	2
29899	M98 250423	UK	1998	invasive (unspecified/other)	Neisseria meningitidis	C	C	C	4	2	1	84	2	2	3	4	3	8	4	6	ST-11 complex	11	2	3	1
29900	M98 250842	UK	1998	invasive (unspecified/other)	Neisseria meningitidis	C	C	C	462	5	1	325	11	2	3	4	3	8	25	6	ST-11 complex	3035	11	2	1
29901	M98 250863	UK	1998	invasive (unspecified/other)	Neisseria meningitidis	C	C	C	22	1	1	1	4	2	3	4	3	8	4	6	ST-11 complex	11	4	4	2
29902	M98 251032	UK	1998	invasive (unspecified/other)	Neisseria meningitidis	C	C	C	NA	5	1	917	11	2	3	4	3	8	4	6	ST-11 complex	11	11	2	NA
30179	M98 251039	UK	1998	invasive (unspecified/other)	Neisseria meningitidis	W	W	W	151	1	1	130	4	2	3	4	3	8	4	6	ST-11 complex	11	4	4	2
29981	M98 251264	UK	1998	invasive (unspecified/other)	Neisseria meningitidis	B	B	B	128	5	1	NA	11	2	3	4	3	8	4	6	ST-11 complex	11	11	2	1
29982	M98 251316	UK	1998	carrier	Neisseria meningitidis	B	C	discrepancy	NA	5	1	917	11	2	3	4	3	8	4	6	ST-11 complex	11	11	2	NA
29903	M98 251459	UK	1998	invasive (unspecified/other)	Neisseria meningitidis	C	C	C	10	5	1	199	11	2	3	4	3	8	4	6	ST-11 complex	11	11	2	1

30180	M98 251534	UK	1998	invasive (unspecified/other)	Neisseria meningitidis	W	W	W		9	1	1	187	47	2	3	4	3	8	4	6	ST-11 complex	11	NA	NA	1
29983	M98 251614	UK	1998	carrier	Neisseria meningitidis	B	C	discrepancy	NA		5	1	917	11	2	3	4	3	8	4	6	ST-11 complex	11	11	2	NA
30171	M98 252111	UK	1998	invasive (unspecified/other)	Neisseria meningitidis	C	C	C		131	5	1	297	11	2	3	4	3	8	4	6	ST-11 complex	11	11	2	1
29905	M98 252366	UK	1998	invasive (unspecified/other)	Neisseria meningitidis	C	C	C		1050	5	1	359	11	2	3	4	3	8	4	6	ST-11 complex	11	11	2	2
29906	M98 252388	UK	1998	invasive (unspecified/other)	Neisseria meningitidis	C	C	C		10	5	1	199	11	2	3	4	3	8	4	6	ST-11 complex	11	11	2	1
29907	M98 252898	UK	1998	invasive (unspecified/other)	Neisseria meningitidis	C	C	C		22	1	1	1	4	2	3	4	3	8	4	6	ST-11 complex	11	4	4	2
29908	M98 252918	UK	1998	invasive (unspecified/other)	Neisseria meningitidis	C	C	C		454	5	1	214	11	2	3	4	3	8	6	6	ST-11 complex	1055	11	2	1
29909	M98 253307	UK	1998	invasive (unspecified/other)	Neisseria meningitidis	C	C	C		462	5	1	325	11	2	3	4	3	8	4	6	ST-11 complex	11	11	2	1
30181	M98 253527	UK	1998	invasive (unspecified/other)	Neisseria meningitidis	W	W	W		151	1	1	130	4	2	3	4	3	8	4	6	ST-11 complex	11	4	4	2
29910	M98 253743	UK	1998	invasive (unspecified/other)	Neisseria meningitidis	C	C	C		2	5	1	270	11	2	3	4	3	8	4	6	ST-11 complex	11	11	2	1
29911	M98 253745	UK	1998	invasive (unspecified/other)	Neisseria meningitidis	C	C	C		236	168	1	360	NA	2	3	4	3	8	4	6	ST-11 complex	11	NA	NA	1
29912	M98 253765	UK	1998	invasive (unspecified/other)	Neisseria meningitidis	C	C	C		78	5	1	245	11	2	3	4	3	8	4	6	ST-11 complex	11	11	2	1
29984	M99 240124	UK	1999	invasive (unspecified/other)	Neisseria meningitidis	B	B	B		781	5	1	292	11	2	3	4	3	8	4	6	ST-11 complex	11	11	2	1
29913	M99 240197	UK	1999	invasive (unspecified/other)	Neisseria meningitidis	C	C	C		10	5	1	199	11	2	3	4	3	8	4	6	ST-11 complex	11	11	2	1
29985	M99 240362	UK	1999	invasive (unspecified/other)	Neisseria meningitidis	B	B	B		118	5	1	73	11	2	3	4	3	8	4	6	ST-11 complex	11	11	2	2
29914	M99 240413	UK	1999	invasive (unspecified/other)	Neisseria meningitidis	C	C	C		784	5	1	272	11	2	3	274	3	8	4	6	ST-11 complex	3455	11	2	1
29915	M99 240568	UK	1999	invasive (unspecified/other)	Neisseria meningitidis	C	C	C		11	5	1	201	11	2	3	4	3	8	4	6	ST-11 complex	11	11	2	1
30194	M99 240591	UK	1999	invasive (unspecified/other)	Neisseria meningitidis	C	C	C		10	5	1	199	11	2	3	4	3	8	4	6	ST-11 complex	11	11	2	1
30195	M99 240592	UK	1999	invasive (unspecified/other)	Neisseria meningitidis	C	C	C		10	5	1	199	11	2	3	4	3	8	4	6	ST-11 complex	11	11	2	1
1207	M99 240593	UK [Wales]	1999	invasive (unspecified/other)	Neisseria meningitidis	C	C	C		19	1	1	65	4	8	26	9	24	26	20	18	ST-212 complex	212	4	4	2
30196	M99 240595	UK	1999	invasive (unspecified/other)	Neisseria meningitidis	C	C	C		16	1	1	60	4	9	18	18	17	327	24	16		4095	4	4	2
30197	M99 240706	UK	1999	invasive (unspecified/other)	Neisseria meningitidis	C	C	C		10	5	1	199	11	2	3	4	3	8	4	6	ST-11 complex	11	11	2	1
30198	M99 240707	UK	1999	invasive (unspecified/other)	Neisseria meningitidis	C	C	C		10	5	1	199	11	2	3	4	3	8	4	6	ST-11 complex	11	11	2	1
30200	M99 240718	UK	1999	invasive (unspecified/other)	Neisseria meningitidis	C	C	C		10	5	1	199	11	2	3	4	3	8	4	6	ST-11 complex	11	11	2	1
30201	M99 240719	UK	1999	invasive (unspecified/other)	Neisseria meningitidis	C	C	C		10	5	1	199	11	2	3	4	3	8	4	6	ST-11 complex	11	11	2	1
29916	M99 240746	UK	1999	invasive (unspecified/other)	Neisseria meningitidis	C	C	C		27	5	1	361	97	2	3	4	3	8	4	6	ST-11 complex	11	NA	NA	2
30202	M99 240747	UK	1999	invasive (unspecified/other)	Neisseria meningitidis	C	C	C		4	2	1	84	2	2	3	4	3	8	4	6	ST-11 complex	11	2	3	1
30203	M99 240748	UK	1999	invasive (unspecified/other)	Neisseria meningitidis	C	C	C		4	2	1	84	2	672	3	4	3	8	4	6	ST-11 complex	11149	2	3	1
30204	M99 240770	UK	1999	carrier	Neisseria meningitidis	C	C	C		10	5	1	199	11	2	3	4	3	8	4	6	ST-11 complex	11	11	2	1
30205	M99 240771	UK	1999	carrier	Neisseria meningitidis	C	C	C		10	5	1	199	11	2	3	4	3	8	4	6	ST-11 complex	11	11	2	1
30206	M99 240772	UK	1999	carrier	Neisseria meningitidis	C	C	C		10	5	1	199	11	2	3	4	3	8	4	6	ST-11 complex	11	11	2	1
30207	M99 240773	UK	1999	carrier	Neisseria meningitidis	C	C	C		10	5	1	199	11	2	3	4	3	8	4	6	ST-11 complex	11	11	2	1
30208	M99 240776	UK	1999	carrier	Neisseria meningitidis	C	C	C		10	5	1	199	11	2	3	4	3	8	4	6	ST-11 complex	11	11	2	1
30182	M99 240896	UK	1999	invasive (unspecified/other)	Neisseria meningitidis	W	W	W		16	1	1	60	4	11	5	18	8	11	4	21	ST-22 complex	184	4	4	2
29917	M99 241004	UK	1999	invasive (unspecified/other)	Neisseria meningitidis	C	C	C		10	5	1	199	11	2	3	4	3	8	4	6	ST-11 complex	11	11	2	1
29919	M99 241431	UK	1999	invasive (unspecified/other)	Neisseria meningitidis	C	C	C		10	5	1	199	11	2	3	4	3	8	4	6	ST-11 complex	11	11	2	1
29986	M99 241503	UK	1999	invasive (unspecified/other)	Neisseria meningitidis	B	B	B		78	5	1	363	11	2	3	4	3	8	4	6	ST-11 complex	11	11	2	1
29920	M99 242207	UK	1999	invasive (unspecified/other)	Neisseria meningitidis	C	C	C		289	5	1	290	11	2	3	4	3	8	4	6	ST-11 complex	11	11	2	1
29921	M99 242251	UK	1999	invasive (unspecified/other)	Neisseria meningitidis	C	C	C		10	5	1	199	11	2	3	4	3	8	4	6	ST-11 complex	11	11	2	1
29922	M99 242522	UK	1999	invasive (unspecified/other)	Neisseria meningitidis	C	C	C		78	5	1	245	11	2	3	4	3	8	4	6	ST-11 complex	11	11	2	1
29923	M99 242630	UK	1999	invasive (unspecified/other)	Neisseria meningitidis	C	C	C		299	5	1	362	11	2	3	4	3	8	4	6	ST-11 complex	11	11	2	1
29987	M99 243153	UK	1999	invasive (unspecified/other)	Neisseria meningitidis	B	B	B		45	5	1	157	11	7	5	1	13	36	53	15	ST-213 complex	213	11	2	3
30269	M99 243594	UK	1999	invasive (unspecified/other)	Neisseria meningitidis	A	A	A		5	37	1	81	125	1	1	2	1	3	2	3	ST-5 complex	5	NA	NA	1
29925	M99 243601	UK	1999	invasive (unspecified/other)	Neisseria meningitidis	C	C	C		259	5	1	215	11	2	3	4	3	8	4	6	ST-11 complex	11	11	2	1
29926	M99 243956	UK	1999	invasive (unspecified/other)	Neisseria meningitidis	C	C	C		16	1	4	59	4	2	3	7	2	8	5	2	ST-8 complex	8	4	4	2
29927	M99 243969	UK	1999	invasive (unspecified/other)	Neisseria meningitidis	C	C	C		10	5	1	199	11	2	3	4	3	8	4	6	ST-11 complex	11	11	2	1
56414	Men20A1	UK	2011	invasive (unspecified/other)	Neisseria meningitidis	A	A	A		94	37	1	106	NA	1	1	2	1	3	334	19	ST-5 complex	4789	NA	NA	3
50888	MT40031	UK [England]	2015	carrier	Neisseria meningitidis	Y	Y	Y		25	1	1	26	4	10	5	18	9	11	9	17	ST-23 complex	23	4	4	2
50890	MT40038	UK [England]	2015	carrier	Neisseria meningitidis	NG	Y	Y		19	1	1	93	13	2	16	6	17	9	18	8	ST-167 complex	168	13	4	2
50892	MT40049	UK [England]	2015	carrier	Neisseria meningitidis	NG	NG	NG		13	3	57	209	3	8	4	7	17	21	26	49	ST-1157 complex	1951	3	3	1
50893	MT40051	UK [England]	2015	carrier	Neisseria meningitidis	B	B	B		47	1	57	90	15	12	5	12	35	60	22	17	ST-461 complex	461	15	4	3
50903	MT40144	UK [England]	2015	carrier	Neisseria meningitidis	NG	B	B		19	1	1	65	4	9	6	9	5	9	64	9	ST-41/44 complex	6075	4	4	2
60961	MT40165	UK [England]	2015	carrier	Neisseria meningitidis	NG	E	E		13	3	57	209	3	8	25	7	17	21	26	49	ST-1157 complex	1157	3	3	1
50905	MT40169	UK [England]	2015	carrier	Neisseria meningitidis	NG	NG	NG		15	4	2	193	1	4	10	15	9	8	11	13	ST-269 complex	467	1	1	1
50911	MT40243	UK [England]	2015	carrier	Neisseria meningitidis	NG	cnl	cnl		4	1	1	206	4	5	4	17	15	30	7	12	ST-198 complex	823	4	3	1
50912	MT40258	UK [England]	2015	carrier	Neisseria meningitidis	NG	cnl	cnl		4	1	1	206	4	5	4	17	15	30	7	12	ST-198 complex	823	4	3	1
50913	MT40286	UK [England]	2015	carrier	Neisseria meningitidis	NG	cnl	cnl		4	1	1	206	4	5	4	17	15	30	7	12	ST-198 complex	823	4	3	1
50914	MT40317	UK [England]	2015	carrier	Neisseria meningitidis	Y	Y	Y		25	1	1	26	4	10	5	18	9	11	9	17	ST-23 complex	23	4	4	2
50915	MT40353	UK [England]	2015	carrier	Neisseria meningitidis	NG	B	B		23	250	1	431	NA	8	5	15	17	8	21	2	ST-865 complex	865	NA	NA	2
50918	MT40391	UK [England]	2015	carrier	Neisseria meningitidis	NG	cnl	cnl		21	1	1	7	4	16	2	159	92	77	25	112	ST-1117 complex	1117	4	4	2
50919	MT40393	UK [England]	2015	carrier	Neisseria meningitidis	NG	B	B		23	250	1	431	NA	8	5	15	17	8	21	2	ST-865 complex	865	NA	NA	2
60966	MT40397	UK [England]	2015	carrier	Neisseria meningitidis	NG	E	E		13	3	57	209	3	8	25	7	17	21	26	49	ST-1157 complex	1157	3	3	1
50925	MT40493	UK [England]	2015	carrier	Neisseria meningitidis	NG	cnl	cnl		4	112	1	206	106	5	4	17	15	30	7	12	ST-198 complex	823	NA	NA	1
50931	MT40543	UK [England]	2015	carrier	Neisseria meningitidis	Y	Y	Y		25	1	1	26	4	10	5	18	9	11	9	17	ST-23 complex	23	4	4	2
54457	N115	UK [England]	2008	carrier	Neisseria meningitidis		E	E		13	3	1	145	3	17	5	19	10	3	26	2	ST-60 complex	1383	3	3	1
54458	N120	UK [England]	2008	carrier	Neisseria meningitidis		E	E		13	3	1	145	3	17	5	19	10	3	26	2	ST-60 complex	1383	3	3	1
53976	N137.1	UK [England]	2008	carrier	Neisseria meningitidis		E	E		13	3	1	145	3	17	5	19	10	3	26	2	ST-60 complex	1383	3	3	1
54459	N145	UK [England]	2008	carrier	Neisseria meningitidis		B	B		13	3	1	145	3	17	5	19	10	3	26	2	ST-60 complex	1383	3	3	1
53930	N222.2	UK [England]	2008	carrier	Neisseria meningitidis		Y	Y		25	1	1	26	4	12	5	18	9	11	9	17	ST-23 complex	1655	4	4	2
53931	N222.3	UK [England]	2008	carrier	Neisseria meningitidis		Y	Y		25	1	1	26	4	12	5	18	9	11	9	17	ST-23 complex	1655	4	4	2
53932	N222.4	UK [England]	2008	carrier	Neisseria meningitidis		Y	Y		25	1	1	26	4	12	5	18	9	11	9	17	ST-23 complex	1655	4	4	2
53933	N222.5	UK [England]	2008	carrier	Neisseria meningitidis		Y	Y		25	1	1	26	4	12	5	18	9	11	9	17	ST-23 complex	1655	4	4	2
53934	N222.6	UK [England]	2008	carrier	Neisseria meningitidis		Y	Y		25	1	1	26	4	12	5	18	9	11	9	17	ST-23 complex	1655	4	4	2
53894	N236.1	UK [England]	2008	carrier	Neisseria meningitidis		Y	Y		13	3	57	172	3	6	5	173	13	5	24	17	ST-174 complex	1466	3	3	1
53895	N236.2	UK [England]	2008	carrier	Neisseria meningitidis		Y	Y		13	3	57	172	3	6	5	173	13	5	24	17	ST-174 complex	1466	3	3	1
53896	N236.3	UK [England]	2008	carrier	Neisseria meningitidis		Y	Y		13	3	57	172	3	6	5	173	13	5	24	17	ST-174 complex	1466	3	3	1
53897	N236.4	UK [England]	2008	carrier	Neisseria meningitidis		Y	Y		13	3	57	172	3	6	5	173	13	5	24	17	ST-174 complex	1466	3	3	1
53898	N236.5	UK [England]	2008	carrier	Neisseria meningitidis		Y	Y		13	3	57	172	3	6	5	173	13	5	24	17	ST-174 complex	1466	3	3	1
53899	N236.6	UK [England]	2008	carrier	Neisseria meningitidis		Y	Y		13	3	57	172	3	6	5	173	13	5	24	17	ST-174 complex	1466	3	3	1
53970	N244.2	UK [England]	2008	carrier	Neisseria meningitidis		B	B		13	3	57	145	3	4	10	34	13	38	11	9	ST-269 complex	6604	3	3	1
53911	N258.2	UK [England]	2008	carrier	Neisseria meningitidis		Y	Y		25	1	1	26	4	12	5	18	9	11	9	17	ST-23 complex	1655	4	4	2
53912	N258.3	UK [England]	2008	carrier	Neisseria meningitidis		Y	Y		25	1	1	26	4	12	5	18	9	11	9	17	ST-23 complex	1655	4	4	2
53913	N258.4	UK [England]	2008	carrier	Neisseria meningitidis		Y	Y		25	1	1	26	4	12	5	18	9	11	9	17	ST-23 complex	1655	4	4	2
53914	N258.5	UK [England]	2008	carrier	Neisseria meningitidis		Y	Y		25	1	1	26	4	12	5	18	9	11	9	17	ST-23 complex	1655	4	4	2
53915	N258.6	UK [England]	2008	carrier	Neisseria meningitidis		Y	Y		25	1	1	26	4	12	5	18	9	11	9	17	ST-23 complex	1655	4	4	2
53952	N259.2	UK [England]	2008	carrier	Neisseria meningitidis		Y	Y		25	1	1	26	4	12	5	18	9	11	9	17	ST-23 complex	1655	4	4	2
53953	N259.3	UK [England]	2008	carrier	Neisseria meningitidis		Y	Y		25	1	1	26	4	12	5	18	9	11	9	17	ST-23 complex	1655	4	4	2
53954	N259.4	UK [England]	2008	carrier	Neisseria meningitidis		Y	Y		25	1	1	26	4	12	5	18	9	11	9	17	ST-23 complex	1655	4	4	2
53955	N259.5	UK [England]	2008	carrier	Neisseria meningitidis		Y	Y		25	1	1	26	4	12	5	18	9	11	9	17	ST-23 complex	1655	4	4	2
53956	N259.6	UK [England]	2008	carrier	Neisseria meningitidis		Y	Y		25	1	1	26	4	12	5	18	9	11	9	17	ST-23 complex	1655	4	4	2
53921	N264.2	UK [England]	2008	carrier	Neisseria meningitidis		Y	Y		25	1	1	26	4	12	5	18	9	11	9	17	ST-23 complex	1655	4	4	2
53922	N264.3	UK [England]	2008	carrier	Neisseria meningitidis		Y	Y		25	1	1	26	4	12	5	18	9	11	9	17	ST-23 complex	1655	4	4	2
53923	N264.4	UK [England]	2008	carrier	Neisseria meningitidis		Y	Y		25	1	1	26	4	12	5	18	9	11	9	17	ST-23 complex	1655	4	4	2
53924	N264.5	UK [England]	2008	carrier	Neisseria meningitidis		Y	Y		25	1	1	26	4	12	5	18	9	11	9	17	ST-23 complex	1655	4	4	2
53925	N264.6	UK [England]	2008	carrier	Neisseria meningitidis		Y	Y		25	1	1	26	4	12	5	18	9	11	9	17	ST-23 complex	1655	4	4	2
53973	N292.1	UK [England]	2008	carrier	Neisseria meningitidis		cnl	cnl		4	1	1	206	4	5	4	17	15	30	7	12	ST-198 complex	823	4	3	1
53935	N309.1	UK [England]	2008	carrier	Neisseria meningitidis		Y	Y		25	1	1	26	4	12	5	18	9	11	9	17	ST-23 complex	1655	4	4	2
53936	N309.2	UK [England]	2008	carrier	Neisseria meningitidis		Y	Y		25	1	1	26	4	12	5	18	9	11	9	17	ST-23 complex	1655	4	4	2
53937	N309.3	UK [England]	2008	carrier	Neisseria meningitidis		Y	Y		25	1	1	26	4	12	5	18	9	11	9	17	ST-23 complex	1655	4	4	2
53938	N309.4	UK [England]	2008	carrier	Neisseria meningitidis		Y	Y		25	1	1	26	4	12	5	18	9	11	9	17	ST-23 complex	1655	4	4	2
53939	N309.5	UK [England]	2008	carrier	Neisseria meningitidis		Y	Y		25	1	1	26	4	12	5	18	9	11	9	17	ST-23 complex	1655	4	4	2
53940	N309.6	UK [England]	2008	carrier	Neisseria meningitidis		Y	Y		25	1	1	26	4	12	5	18	9	11	9	17	ST-23 complex	1655	4	4	2
53974	N311.1	UK [England]	2008	carrier	Neisseria meningitidis		B	B		297	15	81	276	58	7	5	1	13	36	53	15	ST-213 complex	213	NA	NA	1
54460	N320	UK [England]	2008	carrier	Neisseria meningitidis		E	E		25	1	1	26	4	12	5	18	9	11	9	17	ST-23 complex	1655	4	4	2
54461	N329	UK [England]	2009	carrier	Neisseria meningitidis		Y	Y		13	3	57	172	3	6	5	173	13	5	24	17	ST-174 complex	1466	3	3	1
53977	N337.1	UK [England]	2009	carrier	Neisseria meningitidis		B	B		14	1	1	142	13	3	6	19	5	17	6	9	ST-41/44 complex	8511	13	3	1
53900	N354.1	UK [England]	2009	carrier	Neisseria meningitidis		Y	Y		13	3	57	172	3	6	5	173	13	5	24	17	ST-174 complex	1466	3	3	1
53901	N354.2	UK [England]	2009	carrier	Neisseria meningitidis		Y	Y		13	3	57	172	3	6	5	173	13	5	24	17	ST-174 complex	1466	3	3	1
53902	N354.3	UK [England]	2009	carrier	Neisseria meningitidis		Y	Y		13	3	57	172	3	6	5	173	13	5	24	17	ST-174 complex	1466	3	3	1
53903	N354.4	UK [England]	2009	carrier	Neisseria meningitidis		Y	Y		13	3	57	172	3	6	5	173	13	5	24	17	ST-174 complex	1466	3	3	1
53904	N354.5	UK [England]	2009	carrier	Neisseria meningitidis		Y	Y		13	3	57	172	3	6	5	173	13	5	24	17	ST-174 complex	1466	3	3	1
53905	N354.6	UK [England]	2009	carrier	Neisseria meningitidis		Y	Y		13	3	57	172	3	6	5	173	13	5	24	17	ST-174 complex	1466	3	3	1
53926	N359.2	UK [England]	2009	carrier	Neisseria meningitidis		Y	Y		25	1	1	26	4	12	5	18	9	11	9	17	ST-23 complex	1655	4	4	2
53927	N359.3	UK [England]	2009	carrier	Neisseria meningitidis		Y	Y		25	1	1	26	4	12	5	18	9	11	9	17	ST-23 complex	1655	4	4	2
53928	N359.4	UK [England]	2009	carrier	Neisseria meningitidis		Y	Y		25	1	1	26	4	12	5	18	9	11	9	17	ST-23 complex	1655	4	4	2
53929	N359.6	UK [England]	2009	carrier	Neisseria meningitidis		Y	Y		25	1	1	26	4	12	5	18	9	11	9	17	ST-23 complex	1655	4	4	2
53957	N370.1	UK [England]	2009	carrier	Neisseria meningitidis		Y	Y		25	1	1	26	4	12	5	18	9	11	9	17	ST-23 complex	1655	4	4	2
53958	N370.2	UK [England]	2009	carrier	Neisseria meningitidis		Y	Y		25	1	1	26	4	12	5	18	9	11	9	17	ST-23 complex	1655	4	4	2
53959	N370.3	UK [England]	2009	carrier	Neisseria meningitidis		Y	Y		25	1	1	26	4	12	5	18	9	11	9	17	ST-23 complex	1655	4	4	2
53960	N370.4	UK [England]	2009	carrier	Neisseria meningitidis		Y	Y		25	1	1	26	4	12	5	18	9	11	9	17	ST-23 complex	1655	4	4	2
53961	N370.5	UK [England]	2009	carrier	Neisseria meningitidis		Y	Y		25	1	1	26	4	12	5	18	9	11	9	17	ST-23 complex	1655	4	4	2
53962	N370.6	UK [England]	2009	carrier	Neisseria meningitidis		Y	Y		25	1	1	26	4	12	5	18	9	11	9	17	ST-23 complex	1655	4	4	2
53941	N391.1	UK [England]	2009	carrier	Neisseria meningitidis		Y	Y		25	1	1	26	4	12	5	18	9	11	9	17	ST-23 complex	1655	4	4	2
53942	N391.2	UK [England]	2009	carrier	Neisseria meningitidis		Y	Y		25	1	1	26	4	12	5	18	9	11	9	17	ST-23 complex	1655	4	4	2
53943	N391.3	UK [England]	2009	carrier	Neisseria meningitidis		Y	Y		25	1	1	26	4	12	5	18	9	11	9	17	ST-23 complex	1655	4	4	2
53944	N391.4	UK [England]	2009	carrier	Neisseria meningitidis		Y	Y		25	1	1	26	4	12	5	18	9	11	9	17	ST-23 complex	1655	4	4	2
53945	N391.5	UK [England]	2009	carrier	Neisseria meningitidis		Y	Y		25	1	1	26	4	12	5	18	9	11	9	17	ST-23 complex	1655	4	4	2
53946	N391.6	UK [England]	2009	carrier	Neisseria meningitidis		Y	Y		25	1	1	26	4	12	5	18	9	11	9	17	ST-23 complex	1655	4	4	2
54462	N418	UK [England]	2009	carrier	Neisseria meningitidis		E	E		13	3	1	145	3	17	5	19	10	3	26	2	ST-60 complex	1383	3	3	1
54463	N421	UK [England]	2009	carrier	Neisseria meningitidis		Y	Y		23	1	1	4	4	2	7	6	9	16	9	8	ST-167 complex	767	4	4	2
53906	N424.2	UK [England]	2009	carrier	Neisseria meningitidis		Y	Y		13	3	57	172	3	6	5	173	13	5	24	17	ST-174 complex	1466	3	3	1
53907	N424.3	UK [England]	2009	carrier	Neisseria meningitidis		Y	Y		13	3	57	172	3	6	5	173	13	5	24	17	ST-174 complex	1466	3	3	1
53908	N424.4	UK [England]	2009	carrier	Neisseria meningitidis		Y	Y		13	3	57	172	3	6	5	173	13	5	24	17	ST-174 complex	1466	3	3	1
53909	N424.5	UK [England]	2009	carrier	Neisseria meningitidis		Y	Y		13	3	57	172	3	6	5	173	13	5	24	17	ST-174 complex	1466	3	3	1
53910	N424.6	UK [England]	2009	carrier	Neisseria meningitidis		Y	Y		13	3	57	172	3	6	5	173	13	5	24	17	ST-174 complex	1466	3	3	1
53968	N428.1	UK [England]	2009	carrier	Neisseria meningitidis		W	W		16	254	1	60	NA	11	5	18	15	11	24	21	ST-22 complex	1158	NA	NA	2
53916	N431.2	UK [England]	2009	carrier	Neisseria meningitidis		Y	Y		25	1	1	26	4	12	5	18	9	11	9	17	ST-23 complex	1655	4	4	2
53917	N431.3	UK [England]	2009	carrier	Neisseria meningitidis		Y	Y		25	1	1	26	4	12	5	18	9	11	9	17	ST-23 complex	1655	4	4	2

53918	N431.4	UK [England]	2009	carrier	Neisseria meningitidis		Y	Y		25	1	1	26	4	12	5	18	9	11	9	17	ST-23 complex	1655	4	4	2
53919	N431.5	UK [England]	2009	carrier	Neisseria meningitidis		Y	Y		25	1	1	26	4	12	5	18	9	11	9	17	ST-23 complex	1655	4	4	2
53920	N431.6	UK [England]	2009	carrier	Neisseria meningitidis		Y	Y		25	1	1	26	4	12	5	18	9	11	9	17	ST-23 complex	1655	4	4	2
53963	N445.2	UK [England]	2009	carrier	Neisseria meningitidis		Y	Y		25	1	1	26	4	12	5	18	9	11	9	17	ST-23 complex	1655	4	4	2
53964	N445.3	UK [England]	2009	carrier	Neisseria meningitidis		Y	Y		25	1	1	26	4	12	5	18	9	11	9	17	ST-23 complex	1655	4	4	2
53965	N445.4	UK [England]	2009	carrier	Neisseria meningitidis		Y	Y		25	1	1	26	4	12	5	18	9	11	9	17	ST-23 complex	1655	4	4	2
53966	N445.5	UK [England]	2009	carrier	Neisseria meningitidis		Y	Y		25	1	1	26	4	12	5	18	9	11	9	17	ST-23 complex	1655	4	4	2
53967	N445.6	UK [England]	2009	carrier	Neisseria meningitidis		Y	Y		25	1	1	26	4	12	5	18	9	11	9	17	ST-23 complex	1655	4	4	2
53971	N458.1	UK [England]	2009	carrier	Neisseria meningitidis		W	W		16	1	1	60	4	11	5	18	8	11	38	21	ST-22 complex	2638	4	4	2
53947	N459.2	UK [England]	2009	carrier	Neisseria meningitidis		Y	Y		25	1	1	26	4	12	5	18	9	11	9	17	ST-23 complex	1655	4	4	2
53948	N459.3	UK [England]	2009	carrier	Neisseria meningitidis		Y	Y		25	1	1	26	4	12	5	18	9	11	9	17	ST-23 complex	1655	4	4	2
53949	N459.4	UK [England]	2009	carrier	Neisseria meningitidis		Y	Y		25	1	1	26	4	12	5	18	9	11	9	17	ST-23 complex	1655	4	4	2
53950	N459.5	UK [England]	2009	carrier	Neisseria meningitidis		Y	Y		25	1	1	26	4	12	5	18	9	11	9	17	ST-23 complex	1655	4	4	2
53951	N459.6	UK [England]	2009	carrier	Neisseria meningitidis		Y	Y		25	1	1	26	4	12	5	18	9	11	9	17	ST-23 complex	1655	4	4	2
53975	N467.1	UK [England]	2009	carrier	Neisseria meningitidis		cnl	cnl		4	1	1	206	4	5	4	17	15	30	7	12	ST-198 complex	823	4	3	1
53889	N51.2	UK [England]	2008	carrier	Neisseria meningitidis		Y	Y		13	3	57	172	3	6	5	173	13	5	24	17	ST-174 complex	1466	3	3	1
53890	N51.3	UK [England]	2008	carrier	Neisseria meningitidis		Y	Y		13	3	57	172	3	6	5	173	13	5	24	17	ST-174 complex	1466	3	3	1
53891	N51.4	UK [England]	2008	carrier	Neisseria meningitidis		Y	Y		13	3	57	172	3	6	5	173	13	5	24	17	ST-174 complex	1466	3	3	1
53892	N51.5	UK [England]	2008	carrier	Neisseria meningitidis		Y	Y		13	3	57	172	3	6	5	173	13	5	24	17	ST-174 complex	1466	3	3	1
53893	N51.6	UK [England]	2008	carrier	Neisseria meningitidis		Y	Y		13	3	57	172	3	6	5	173	13	5	24	17	ST-174 complex	1466	3	3	1
53969	N63.1.1	UK [England]	2008	carrier	Neisseria meningitidis		Y	Y		21	1	1	7	4	6	5	173	515	5	24	17	ST-174 complex	8510	4	4	2
53972	N82.1	UK [England]	2008	carrier	Neisseria meningitidis		Y	Y		23	1	1	4	4	2	7	6	9	16	9	8	ST-167 complex	767	4	4	2
31199	NM10052	UK [England]	2013	invasive (unspecified/other)	Neisseria meningitidis	B	B	B		13	3	57	145	3	4	10	34	5	38	11	9	ST-269 complex	1161	3	3	1
31200	NM10053	UK [England]	2013	invasive (unspecified/other)	Neisseria meningitidis	B	B	B		13	3	57	145	3	4	10	34	5	38	11	9	ST-269 complex	1161	3	3	1
31201	NM10313	UK [England]	2013	invasive (unspecified/other)	Neisseria meningitidis	Y	Y	Y		25	1	1	26	4	10	5	18	9	11	9	22	ST-23 complex	10732	4	4	2
31208	NM10853	UK [England]	2013	carrier	Neisseria meningitidis		cnl	cnl		102	8	1	2	20	16	2	6	25	17	25	22	ST-53 complex	53	20	4	2
31209	NM10863	UK [England]	2013	invasive (unspecified/other)	Neisseria meningitidis	B	B	B		4	1	59	84	4	686	6	9	5	9	21	9	ST-41/44 complex	10868	4	3	1
31210	NM10864	UK [England]	2013	invasive (unspecified/other)	Neisseria meningitidis	B	B	B		4	2	1	84	2	3	6	9	5	9	6	2	ST-41/44 complex	2314	2	3	1
31212	NM11067	UK [England]	2013	invasive (unspecified/other)	Neisseria meningitidis	B	B	B		765	9	5	144	21	27	6	9	3	9	6	16	ST-41/44 complex	136	NA	NA	1
31213	NM11099	UK [England]	2013	invasive (unspecified/other)	Neisseria meningitidis	B	B	B		31	9	5	58	16	7	5	1	13	36	53	15	ST-213 complex	213	16	2	3
31176	NM8250	UK [England]	2011	invasive (unspecified/other)	Neisseria meningitidis	B	B	B		14	7	1	142	6	3	6	9	8	9	22	9	ST-41/44 complex	8052	6	2	1
31190	NM9062	UK [England]	2012	invasive (unspecified/other)	Neisseria meningitidis	B	B	B		14	7	1	142	6	3	6	9	5	9	6	9	ST-41/44 complex	41	6	2	1
31197	NM9905	UK [England]	2012	invasive (unspecified/other)	Neisseria meningitidis	B	B	B		702	31	57	281	140	6	5	2	151	12	11	14		1976	NA	NA	1
31198	NM9954	UK [England]	2012	invasive (unspecified/other)	Neisseria meningitidis	C	C	C	NA		5	1	917	11	2	3	4	3	8	4	6	ST-11 complex	11	11	2	NA
2813	OX0050004	UK [England]	2000	carrier	Neisseria meningitidis	NG	B	B		19	1	1	65	4	27	6	9	5	9	6	9	ST-41/44 complex	577	4	4	2
28899	OX0050007	UK [England]	2000	carrier	Neisseria meningitidis	B	B	B		19	1	1	65	4	9	20	9	9	9	6	2	ST-41/44 complex	1097	4	4	2
35899	OX0050049	UK [England]	2000	carrier	Neisseria meningitidis		B	B		19	1	1	83	4	4	10	2	5	38	11	9	ST-269 complex	275	4	4	2
28900	OX0050057	UK [England]	2000	carrier	Neisseria meningitidis	B	B	B		19	1	1	65	4	9	6	9	9	9	64	9	ST-41/44 complex	409	4	4	2
35900	OX0050081	UK [England]	2000	carrier	Neisseria meningitidis		cnl	cnl		94	184	56	89	NA	5	4	38	15	22	40	13	ST-1136 complex	1136	NA	NA	3
35901	OX0050092	UK [England]	2000	carrier	Neisseria meningitidis		W	W		16	1	1	60	4	11	5	18	8	153	4	21	ST-22 complex	1426	4	4	2
35902	OX0050158	UK [England]	2000	carrier	Neisseria meningitidis		B	B		21	1	1	7	4	1	5	13	53	26	41	3	ST-162 complex	162	4	4	2
35903	OX0050185	UK [England]	2000	carrier	Neisseria meningitidis		X	X		13	3	57	145	3	8	4	6	17	11	18	2	ST-103 complex	862	3	3	1
28901	OX0050209	UK [England]	2000	carrier	Neisseria meningitidis	NG	cnl	cnl		14	7	1	142	6	3	6	9	5	9	6	9	ST-41/44 complex	41	6	2	1
28902	OX0050225	UK [England]	2000	carrier	Neisseria meningitidis	B	B	B		4	1	1	206	4	3	6	9	9	9	6	9	ST-41/44 complex	303	4	3	1
35904	OX0050256	UK [England]	2000	carrier	Neisseria meningitidis		B	B		16	1	4	59	4	4	10	11	18	6	10	12	ST-35 complex	35	4	4	2
35905	OX0050268	UK [England]	2000	carrier	Neisseria meningitidis		E	E		13	3	57	145	3	2	16	12	11	3	60	7	ST-254 complex	254	3	3	1
28903	OX0050304	UK [England]	2000	carrier	Neisseria meningitidis	NG	B	B		338	7	1	NA	6	3	6	9	5	9	6	9	ST-41/44 complex	41	6	2	1
35906	OX0050325	UK [England]	2000	carrier	Neisseria meningitidis		B	B		45	8	77	87	20	7	5	1	13	36	53	15	ST-213 complex	213	20	4	3
35907	OX0050360	UK [England]	2000	carrier	Neisseria meningitidis		B	B		1	6	3	100	7	4	10	5	4	5	3	2	ST-32 complex	74	7	1	1
35908	OX0050377	UK [England]	2000	carrier	Neisseria meningitidis		W	W		22	1	1	1	4	11	5	9	17	11	24	21	ST-22 complex	1221	4	4	2
35909	OX0050393	UK [England]	2000	carrier	Neisseria meningitidis		B	B		15	4	2	193	1	4	10	15	9	8	11	9	ST-269 complex	269	1	1	1
2818	OX0050408	UK [England]	2000	carrier	Neisseria meningitidis	B	B	B		19	1	1	65	4	9	6	9	5	9	24	9	ST-41/44 complex	1875	4	4	2
2819	OX0050459	UK [England]	2000	carrier	Neisseria meningitidis	B	B	B		19	1	1	65	4	9	6	9	9	120	6	9	ST-41/44 complex	1876	4	4	2
35910	OX0050468	UK [England]	2000	carrier	Neisseria meningitidis		E	E		13	3	1	209	3	8	25	7	17	21	26	2	ST-1157 complex	1421	3	3	1
28904	OX0050570	UK [England]	2000	carrier	Neisseria meningitidis	B	B	B		4	2	1	84	2	3	6	9	5	9	6	9	ST-41/44 complex	41	2	3	1
35911	OX0050574	UK [England]	2000	carrier	Neisseria meningitidis		W	W		16	1	1	60	4	NA	5	18	8	174	24	21		NA	4	4	2
35912	OX0050607	UK [England]	2000	carrier	Neisseria meningitidis		B	B		15	4	2	193	1	4	10	15	9	NA	11	9		NA	1	1	1
2913	OX0050673	UK [England]	2000	carrier	Neisseria meningitidis	NG	cnl	cnl		94	NA	56	69	NA	5	4	17	15	14	181	12	ST-198 complex	1956	NA	NA	3
28905	OX0050677	UK [England]	2000	carrier	Neisseria meningitidis	NG	B	B		19	160	1	65	200	27	6	9	5	9	6	9	ST-41/44 complex	577	NA	NA	2
35913	OX0050776	UK [England]	2000	carrier	Neisseria meningitidis		W	W		16	1	1	477	NA	11	5	18	8	11	24	21	ST-22 complex	22	NA	NA	2
35914	OX0050781	UK [England]	2000	carrier	Neisseria meningitidis		W	W		16	1	NA	60	4	11	5	18	8	11	38	21	ST-22 complex	2638	4	4	2
35915	OX0050852	UK [England]	2000	carrier	Neisseria meningitidis		E	E		13	3	1	145	3	17	5	19	17	3	26	2	ST-60 complex	60	3	3	1
35916	OX0050858	UK [England]	2000	carrier	Neisseria meningitidis		E	E		13	3	57	209	3	8	25	7	17	21	26	49	ST-1157 complex	1157	3	3	1
28906	OX0050872	UK [England]	2000	carrier	Neisseria meningitidis	B	B	B		4	2	1	84	2	3	6	9	5	9	6	9	ST-41/44 complex	41	2	3	1
28907	OX0050873	UK [England]	2000	carrier	Neisseria meningitidis	B	B	B		4	2	1	84	2	3	6	9	5	9	6	9	ST-41/44 complex	41	2	3	1
28908	OX0050898	UK [England]	2000	carrier	Neisseria meningitidis	NG	B	B		19	1	1	335	4	9	6	9	9	9	6	9	ST-41/44 complex	44	4	4	2
4259	OX0050901	UK [England]	2000	carrier	Neisseria meningitidis	NG	cnl	cnl		119	1	1	98	4	6	3	4	17	250	13	169		3104	4	4	2
35917	OX0050902	UK [England]	2000	carrier	Neisseria meningitidis		B	B		558	8	77	478	20	7	5	1	13	36	53	15	ST-213 complex	213	20	4	3
28909	OX0050926	UK [England]	2000	carrier	Neisseria meningitidis	NG	B	B		19	1	1	335	4	9	6	9	9	9	6	9	ST-41/44 complex	44	4	4	2
36212	OX0050940	UK [England]	2000	carrier	Neisseria meningitidis	B	B	B		4	2	79	84	2	3	6	9	5	9	6	9	ST-41/44 complex	41	2	3	1
28911	OX0050941	UK [England]	2000	carrier	Neisseria meningitidis	B	B	B		19	1	1	65	4	9	20	9	9	9	6	2	ST-41/44 complex	1097	4	4	2
35918	OX0050946	UK [England]	2000	carrier	Neisseria meningitidis		W	W		138	5	1	112	11	11	5	18	8	11	24	21	ST-22 complex	22	11	2	2
35919	OX0050965	UK [England]	2000	carrier	Neisseria meningitidis		W	W		138	5	1	112	11	11	5	18	8	11	24	21	ST-22 complex	22	11	2	2
3106	OX0050971	UK [England]	2000	carrier	Neisseria meningitidis	NG	cnl	cnl		285	1	1	5	4	12	91	6	181	177	186	8		2121	4	4	2
28912	OX0051021	UK [England]	2000	carrier	Neisseria meningitidis	B	cnl	discrepancy		102	8	1	2	20	16	2	6	25	17	25	22	ST-53 complex	53	20	4	2
35920	OX0051080	UK [England]	2000	carrier	Neisseria meningitidis		cnl	cnl		102	8	1	2	20	16	2	6	25	17	25	22	ST-53 complex	53	20	4	2
3101	OX0051091	UK [England]	2000	carrier	Neisseria meningitidis	B	B	B		15	4	2	193	1	4	10	15	9	5	11	9	ST-269 complex	2091	1	1	1
35921	OX0051099	UK [England]	2000	carrier	Neisseria meningitidis		B	B		45	8	77	87	20	7	5	1	13	36	53	15	ST-213 complex	213	20	4	3
35922	OX0051106	UK [England]	2000	carrier	Neisseria meningitidis		cnl	cnl		297	15	1	276	58	16	2	6	25	17	25	22	ST-53 complex	53	NA	NA	1
35923	OX0051114	UK [England]	2000	carrier	Neisseria meningitidis		B	B		187	1	62	77	4	7	5	4	13	36	6	15	ST-213 complex	1644	4	4	3
28913	OX0051136	UK [England]	2000	carrier	Neisseria meningitidis	B	B	B		19	1	1	65	4	9	6	9	9	9	6	2	ST-41/44 complex	180	4	4	2
3103	OX0051143	UK [England]	2000	carrier	Neisseria meningitidis	NG	cnl	cnl		4	1	1	206	4	5	4	17	15	30	7	12	ST-198 complex	823	4	3	1
35925	OX0051153	UK [England]	2000	carrier	Neisseria meningitidis		cnl	cnl		94	138	56	69	NA	5	4	17	15	14	7	12	ST-198 complex	198	NA	NA	3
35926	OX0051189	UK [England]	2000	carrier	Neisseria meningitidis		B	B		47	1	57	90	15	12	5	12	35	60	22	17	ST-461 complex	461	15	4	3
35927	OX0051290	UK [England]	2000	carrier	Neisseria meningitidis		B	B		21	1	1	7	4	1	5	13	53	26	41	3	ST-162 complex	162	4	4	2
35928	OX0051317	UK [England]	2000	carrier	Neisseria meningitidis		B	B		21	1	1	7	4	1	5	13	17	26	41	3	ST-162 complex	755	4	4	2
2914	OX0051332	UK [England]	2000	carrier	Neisseria meningitidis	NG	cnl	cnl		16	1	1	60	4	3	6	9	11	11	6	21	ST-41/44 complex	1957	4	4	2
3193	OX0051350	UK [England]	2000	carrier	Neisseria meningitidis	NG	B	B		4	2	1	84	2	16	6	9	5	9	6	9	ST-41/44 complex	2189	2	3	1
28914	OX0051387	UK [England]	2000	carrier	Neisseria meningitidis	B	B	B		24	1	2	25	4	135	6	9	3	9	6	6	ST-41/44 complex	1473	4	4	2
28915	OX0051404	UK [England]	2000	carrier	Neisseria meningitidis	NG	B	B		19	1	1	65	4	9	20	9	9	9	6	2	ST-41/44 complex	1097	4	4	2
35929	OX0051410	UK [England]	2000	carrier	Neisseria meningitidis		E	E		13	3	1	145	3	17	5	19	17	3	26	2	ST-60 complex	60	3	3	1
35930	OX0051415	UK [England]	2000	carrier	Neisseria meningitidis		Y	Y		16	1	1	60	4	11	5	18	8	11	24	21	ST-22 complex	22	4	4	2
28916	OX0051423	UK [England]	2000	carrier	Neisseria meningitidis	NG	B	B		19	1	1	65	4	12	6	9	9	9	6	9	ST-41/44 complex	43	4	4	2
28917	OX0052019	UK [England]	2000	carrier	Neisseria meningitidis	B	B	B		557	2	1	407	2	3	6	9	5	9	6	9	ST-41/44 complex	41	2	4	2
35931	OX0052051	UK [England]	2000	carrier	Neisseria meningitidis		W	W		16	1	1	60	4	11	5	18	8	11	4	21	ST-22 complex	184	4	4	2
36213	OX0052076	UK [England]	2000	carrier	Neisseria meningitidis	B	B	B		24	1	2	25	4	27	6	9	3	9	6	16	ST-41/44 complex	136	4	4	2
28919	OX0052120	UK [England]	2000	carrier	Neisseria meningitidis	B	B	B		24	1	2	25	4	27	6	9	3	9	6	16	ST-41/44 complex	136	4	4	2
28920	OX0052129	UK [England]	2000	carrier	Neisseria meningitidis	NG	B	B		19	1	1	65	4	9	6	9	9	9	64	9	ST-41/44 complex	409	4	4	2
35932	OX0052158	UK [England]	2000	carrier	Neisseria meningitidis		E	E		13	3	1	209	3	8	25	7	17	21	26	2	ST-1157 complex	1421	3	3	1
28921	OX0052162	UK [England]	2000	carrier	Neisseria meningitidis	NG	B	B		4	2	1	84	2	3	6	9	5	9	6	9	ST-41/44 complex	41	2	3	1
28922	OX0052296	UK [England]	2000	carrier	Neisseria meningitidis	B	B	B		24	1	1	25	4	9	6	9	9	9	6	2	ST-41/44 complex	180	4	4	2
35096	OX0052328	UK [England]	2000	carrier	Neisseria meningitidis	Y	Y	Y		23	1	1	4	4	2	7	6	9	16	9	8	ST-167 complex	767	4	4	2
28923	OX0052474	UK [England]	2000	carrier	Neisseria meningitidis	B	B	B		19	1	1	65	4	9	20	9	9	9	6	2	ST-41/44 complex	1097	4	4	2
28924	OX0052529	UK [England]	2000	carrier	Neisseria meningitidis	B	B	B		4	2	1	84	2	3	6	9	5	9	6	9	ST-41/44 complex	41	2	3	1
35933	OX0052813	UK [England]	2000	carrier	Neisseria meningitidis		cnl	cnl		102	8	1	2	20	16	2	6	25	17	25	22	ST-53 complex	53	20	4	2
35934	OX0052881	UK [England]	2000	carrier	Neisseria meningitidis		B	B		15	4	2	193	1	4	10	15	9	8	11	9	ST-269 complex	269	1	1	1
28925	OX0052958	UK [England]	2000	carrier	Neisseria meningitidis	B	B	B		19	1	1	65	4	3	6	9	60	9	26	9	ST-41/44 complex	1415	4	4	2
29461	OX0053039	UK [England]	2000	carrier	Neisseria meningitidis	NG	E	E		13	3	57	209	3	8	6	7	17	21	26	49	ST-1157 complex	1419	3	3	1
29462	OX0053048	UK [England]	2000	carrier	Neisseria meningitidis		cnl	cnl		102	8	1	2	20	16	2	6	25	17	25	22	ST-53 complex	53	20	4	2
28926	OX01060046	UK [England]	2001	carrier	Neisseria meningitidis	B	B	B		4	2	1	84	2	16	6	9	5	9	6	9	ST-41/44 complex	2189	2	3	1
28927	OX01060062	UK [England]	2001	carrier	Neisseria meningitidis	NG	NG	NG		19	1	1	336	4	6	6	12	9	9	6	17	ST-41/44 complex	1857	4	4	2
35935	OX01060129	UK [England]	2001	carrier	Neisseria meningitidis		E	E		13	3	1	145	3	17	5	19	17	3	26	2	ST-60 complex	60	3	3	1
3479	OX01060163	UK [England]	2001	carrier	Neisseria meningitidis	B	B	B		19	1	1	65	4	9	20	212	9	9	6	2	ST-41/44 complex	2417	4	4	2
36214	OX01060217	UK [England]	2001	carrier	Neisseria meningitidis	B	B	B		19	1	1	65	4	3	6	9	60	9	26	9	ST-41/44 complex	1415	4	4	2
35936	OX01060268	UK [England]	2001	carrier	Neisseria meningitidis		E	E		13	3	NA	209	3	8	25	7	17	21	26	49	ST-1157 complex	1157	3	3	1
35937	OX01060273	UK [England]	2001	carrier	Neisseria meningitidis		E	E		13	3	1	145	3	17	5	19	17	3	26	2	ST-60 complex	60	3	3	1
28929	OX01060279	UK [England]	2001	carrier	Neisseria meningitidis	B	B	B		14	1	1	142	13	3	6	108	9	9	6	9	ST-41/44 complex	2364	13	3	1
35938	OX01060284	UK [England]	2001	carrier	Neisseria meningitidis		cnl	cnl		119	1	1	98	4	6	3	4	17	8	13	169		2196	4	4	2
28930	OX01060297	UK [England]	2001	carrier	Neisseria meningitidis	B	B	B		21	1	90	7	4	9	6	9	17	9	6	9	ST-41/44 complex	437	4	4	2
36215	OX01060367	UK [England]	2001	carrier	Neisseria meningitidis	B	B	B		24	1	2	25	4	27	6	9	3	9	6	16	ST-41/44 complex	136	4	4	2
35939	OX01060373	UK [England]	2001	carrier	Neisseria meningitidis		B	B		558	8	77	478	20	7	5	1	13	36	53	15	ST-213 complex	213	20	4	3
35940	OX01060380	UK [England]	2001	carrier	Neisseria meningitidis		B	B		1	6	3	100	7	4	10	5	4	5	3	2	ST-32 complex	74	7	1	1
35941	OX01060426	UK [England]	2001	carrier	Neisseria meningitidis		B	B		45	8	77	87	20	7	5	1	13	36	53	15	ST-213 complex	213	20	4	3
28932	OX01060472	UK [England]	2001	carrier	Neisseria meningitidis	NG	B	B		4	2	1	84	2	3	6	9	5	9	6	9	ST-41/44 complex	41	2	3	1
35942	OX01060508	UK [England]	2001	carrier	Neisseria meningitidis		W	W		16	1	1	60	4	11	5	18	8	11	4	21	ST-22 complex	184	4	4	2
28933	OX01060561	UK [England]	2001	carrier	Neisseria meningitidis	NG	B	B		14	1	1	142	13	3	6	9	17	9	6	9	ST-41/44 complex	482	13	3	1
28934	OX01060596	UK [England]	2001	carrier	Neisseria meningitidis	B	B	B		202	1	1	35	4	27	6	9	3	9	6	16	ST-41/44 complex	136	4	4	2
28935	OX01060652	UK [England]	2001	carrier	Neisseria meningitidis	B	B	B		19	1	1	65	4	9	20	9	9	9	6	2	ST-41/44 complex	1097	4	4	2
35943	OX01060686	UK [England]	2001	carrier	Neisseria meningitidis		B	B		45	8	77	87	20	7	5	1	13	36	53	15	ST-213 complex	213	20	4	3
35944	OX01060731	UK [England]	2001	carrier	Neisseria meningitidis		B	B		15	4	2	193	1	4	10	15	9	5	11	9	ST-269 complex	2091	1	1	1
35945	OX01060795	UK [England]	2001	carrier	Neisseria meningitidis		B	B		13	3	1	145	3	12	5	19	160	3	174	124		1653	3	3	1
35946	OX01060836	UK [England]	2001	carrier	Neisseria meningitidis		B	B		47	1	57	90	15	12	5	12	35	60	22	17	ST-461 complex	461	15	4	3
35947	OX01060852	UK [England]	2001	carrier	Neisseria meningitidis		cnl	cnl		4	1	1	206	4	5	4	17	15	30	7	12	ST-198 complex	823	4	3	1
4204	OX01060886	UK [England]	2001	carrier	Neisseria meningitidis	B	B	B		19	1	1	65	4	3	6	9	9	9	13	9	ST-41/44 complex	3049	4	4	2
36216	OX01060896	UK [England]	2001	carrier	Neisseria meningitidis	B	B	B		19	1	1	65	4	12	26	9	9	665	6	9	ST-41/44 complex	10309	4	4	2
28936	OX01060915	UK [England]	2001	carrier	Neisseria meningitidis	B	B	B		19	1	1	65	4	9	20	9	9	9	6	2	ST-41/44 complex	1097	4	4	2
28937	OX01060954	UK [England]	2001	carrier	Neisseria meningitidis	NG	B	B		24	1	2	25	4	75	6	83	9	9	6	9	ST-41/44 complex	3259	4	4	2
28938	OX01060960	UK [England]	2001	carrier	Neisseria meningitidis	NG	E	E		13	3	1	145	3	12	5	4	17	3	10	124		2183	3	3	1

28939	OX01061044	UK [England]	2001	carrier	Neisseria meningitidis	NG	NG	NG	24	1	2	25	4	9	6	9	9	15	6	9	ST-41/44 complex	2413	4	4	2
35948	OX01061080	UK [England]	2001	carrier	Neisseria meningitidis		B	B	19	1	1	83	4	4	10	2	5	38	11	9	ST-269 complex	275	4	4	2
4398	OX01061086	UK [England]	2001	carrier	Neisseria meningitidis	NG	B	B	24	1	2	25	4	28	6	9	17	9	6	9	ST-41/44 complex	3248	4	4	2
35949	OX01061099	UK [England]	2001	carrier	Neisseria meningitidis		B	B	16	1	4	59	4	4	10	11	18	6	10	12	ST-35 complex	35	4	4	2
28940	OX01061115	UK [England]	2001	carrier	Neisseria meningitidis	NG	B	B	19	1	1	65	4	9	6	9	9	9	6	2	ST-41/44 complex	180	4	4	2
35950	OX01061117	UK [England]	2001	carrier	Neisseria meningitidis		cnl	cnl	94	184	56	89	NA	5	4	38	15	22	40	13	ST-1136 complex	1136	NA	NA	3
28941	OX01061123	UK [England]	2001	carrier	Neisseria meningitidis	NG	B	B	19	1	1	65	4	9	6	9	9	9	6	2	ST-41/44 complex	180	4	4	2
35951	OX01061128	UK [England]	2001	carrier	Neisseria meningitidis		B	B	21	1	1	7	4	1	5	13	53	26	41	3	ST-162 complex	162	4	4	2
28942	OX01061131	UK [England]	2001	carrier	Neisseria meningitidis	B	B	B	19	1	1	65	4	12	6	9	9	9	6	9	ST-41/44 complex	43	4	4	2
36217	OX01061140	UK [England]	2001	carrier	Neisseria meningitidis	NG	NG	NG	24	1	2	25	4	9	6	9	9	15	6	9	ST-41/44 complex	2413	4	4	2
35952	OX01061141	UK [England]	2001	carrier	Neisseria meningitidis		X	X	13	3	57	145	3	8	4	6	17	11	18	2	ST-103 complex	862	3	3	1
36218	OX01061310	UK [England]	2001	carrier	Neisseria meningitidis	B	B	B	19	1	1	65	4	12	6	9	9	9	6	9	ST-41/44 complex	43	4	4	2
28944	OX01061328	UK [England]	2001	carrier	Neisseria meningitidis	NG	B	B	4	2	1	84	2	3	6	9	5	9	6	9	ST-41/44 complex	41	2	3	1
35953	OX01061379	UK [England]	2001	carrier	Neisseria meningitidis			NA	16	1	1	477	NA	11	5	18	8	11	24	21	ST-22 complex	22	NA	NA	2
3661	OX01061392	UK [England]	2001	carrier	Neisseria meningitidis	NG	W	W	16	1	1	60	4	11	5	18	8	11	38	21	ST-22 complex	2638	4	4	2
28945	OX01061416	UK [England]	2001	carrier	Neisseria meningitidis	B	B	B	19	1	1	65	4	12	6	9	9	9	6	9	ST-41/44 complex	43	4	4	2
28946	OX01061431	UK [England]	2001	carrier	Neisseria meningitidis	NG	B	B	19	1	1	65	4	9	20	9	9	9	6	2	ST-41/44 complex	1097	4	4	2
28947	OX01061451	UK [England]	2001	carrier	Neisseria meningitidis	NG	B	B	19	1	1	65	4	9	6	9	9	9	64	9	ST-41/44 complex	409	4	4	2
28948	OX01061511	UK [England]	2001	carrier	Neisseria meningitidis	NG	cnl	cnl	14	18	1	166	87	3	6	9	5	11	6	9	ST-41/44 complex	154	NA	NA	1
29463	OX01061541	UK [England]	2001	carrier	Neisseria meningitidis		B	B	19	1	1	65	4	NA	6	15	9	9	6	18		NA	4	4	2
36219	OX01061577	UK [England]	2001	carrier	Neisseria meningitidis	NG	B	B	4	2	1	84	2	3	6	9	5	9	6	9	ST-41/44 complex	41	2	3	1
36220	OX01061579	UK [England]	2001	carrier	Neisseria meningitidis	B	B	B	19	1	1	65	4	12	6	6	9	9	6	9	ST-41/44 complex	2644	4	4	2
28950	OX01061587	UK [England]	2001	carrier	Neisseria meningitidis	B	B	B	19	1	1	65	4	3	6	9	5	9	18	9	ST-41/44 complex	2080	4	4	2
28951	OX01061635	UK [England]	2001	carrier	Neisseria meningitidis	NG	C	C	19	1	1	93	13	12	6	9	17	9	6	9	ST-41/44 complex	206	13	4	2
3664	OX01061649	UK [England]	2001	carrier	Neisseria meningitidis	B	B	B	19	1	1	65	4	3	6	9	9	9	6	206	ST-41/44 complex	2631	4	4	2
28952	OX01061707	UK [England]	2001	carrier	Neisseria meningitidis	NG	B	B	4	2	1	84	2	3	6	9	5	9	6	9	ST-41/44 complex	41	2	3	1
28953	OX01061748	UK [England]	2001	carrier	Neisseria meningitidis	B	B	B	4	2	1	84	2	3	6	9	5	9	6	9	ST-41/44 complex	41	2	3	1
28954	OX01061840	UK [England]	2001	carrier	Neisseria meningitidis	NG	B	B	19	1	1	335	4	9	6	9	9	9	6	9	ST-41/44 complex	44	4	4	2
35954	OX01061911	UK [England]	2001	carrier	Neisseria meningitidis		cnl	cnl	102	8	1	2	20	16	2	6	25	17	25	22	ST-53 complex	53	20	4	2
35955	OX01061948	UK [England]	2001	carrier	Neisseria meningitidis		B	B	15	4	2	193	1	4	10	15	9	8	11	9	ST-269 complex	269	1	1	1
4395	OX01061972	UK [England]	2001	carrier	Neisseria meningitidis	NG	B	B	19	1	1	65	4	27	6	9	5	6	6	12	ST-41/44 complex	3245	4	4	2
4231	OX01061987	UK [England]	2001	carrier	Neisseria meningitidis	B	B	B	16	1	NA	59	4	4	10	11	11	6	18	12	ST-35 complex	3073	4	4	2
28955	OX01062035	UK [England]	2001	carrier	Neisseria meningitidis	B	B	B	19	1	1	93	13	27	6	11	5	9	6	9	ST-41/44 complex	1399	13	4	2
28956	OX01062098	UK [England]	2001	carrier	Neisseria meningitidis	NG	cnl	cnl	16	1	1	60	4	3	6	9	11	11	6	21	ST-41/44 complex	1957	4	4	2
3490	OX01062124	UK [England]	2001	carrier	Neisseria meningitidis	B	B	B	14	1	1	142	13	3	6	9	5	5	6	9	ST-41/44 complex	2362	13	3	1
28957	OX01062156	UK [England]	2001	carrier	Neisseria meningitidis	B	B	B	19	1	1	65	4	12	6	9	9	9	6	9	ST-41/44 complex	43	4	4	2
28958	OX01062220	UK [England]	2001	carrier	Neisseria meningitidis	B	B	B	4	2	1	84	2	3	6	9	5	9	6	9	ST-41/44 complex	41	2	3	1
28959	OX01062252	UK [England]	2001	carrier	Neisseria meningitidis	B	B	B	4	2	1	84	2	3	6	9	5	9	6	9	ST-41/44 complex	41	2	3	1
28960	OX01062298	UK [England]	2001	carrier	Neisseria meningitidis	NG	B	B	4	2	79	84	2	3	6	9	5	9	6	9	ST-41/44 complex	41	2	3	1
36221	OX01062311	UK [England]	2001	carrier	Neisseria meningitidis	B	B	B	14	1	1	142	13	3	6	9	60	9	6	156	ST-41/44 complex	3048	13	3	1
29464	OX01062316	UK [England]	2001	carrier	Neisseria meningitidis	NG	NG	NG	23	1	1	4	4	2	7	6	9	16	9	8	ST-167 complex	767	4	4	2
28961	OX01062341	UK [England]	2001	carrier	Neisseria meningitidis	NG	B	B	4	2	1	84	2	3	6	9	5	9	6	9	ST-41/44 complex	41	2	3	1
28962	OX01062350	UK [England]	2001	carrier	Neisseria meningitidis	B	B	B	19	1	1	65	4	3	6	9	24	9	38	9	ST-41/44 complex	1414	4	4	2
28963	OX01062357	UK [England]	2001	carrier	Neisseria meningitidis	NG	B	B	4	2	1	84	2	3	6	9	5	9	6	9	ST-41/44 complex	41	2	3	1
28964	OX01062462	UK [England]	2001	carrier	Neisseria meningitidis	B	B	B	19	1	1	65	4	9	20	9	9	9	6	2	ST-41/44 complex	1097	4	4	2
36222	OX01062468	UK [England]	2001	carrier	Neisseria meningitidis	NG	NG	NG	24	1	2	25	4	9	6	9	9	15	6	9	ST-41/44 complex	2413	4	4	2
50884	OX40017	UK [England]	2014	carrier	Neisseria meningitidis	NG	E	E	13	3	1	145	3	12	5	9	17	3	172	124		3207	3	3	1
44440	OX40020	UK [England]	2014	carrier	Neisseria meningitidis	NG	E	E	13	3	1	145	3	12	5	9	17	3	172	124		3207	3	3	1
44441	OX40027	UK [England]	2014	carrier	Neisseria meningitidis	NG	cnl	cnl	4	1	1	206	4	5	4	17	15	30	7	12	ST-198 complex	823	4	3	1
52636	OX40028	UK [England]	2014	carrier	Neisseria meningitidis	NG	NG	NG	525	98	1	457	122	285	6	9	1	9	6	9	ST-41/44 complex	4184	NA	NA	1
44443	OX40041	UK [England]	2014	carrier	Neisseria meningitidis	NG	cnl	cnl	4	1	1	206	4	5	4	17	15	30	7	12	ST-198 complex	823	4	3	1
44444	OX40046	UK [England]	2014	carrier	Neisseria meningitidis	NG	W	W	22	1	1	1	4	2	3	4	3	8	4	6	ST-11 complex	11	4	4	2
44445	OX40048	UK [England]	2014	carrier	Neisseria meningitidis	NG	W	W	22	1	1	1	4	2	3	4	3	8	4	6	ST-11 complex	11	4	4	2
52637	OX40064	UK [England]	2014	carrier	Neisseria meningitidis	NG	B	B	19	1	2	65	4	7	5	1	17	36	53	15	ST-213 complex	2391	4	4	2
44447	OX40081	UK [England]	2014	carrier	Neisseria meningitidis	NG	cnl	cnl	4	1	1	206	4	5	4	17	15	30	7	12	ST-198 complex	823	4	3	1
44449	OX40119	UK [England]	2014	carrier	Neisseria meningitidis	W	W	W	22	1	1	1	4	662	3	4	3	8	4	6	ST-11 complex	10651	4	4	2
44450	OX40121	UK [England]	2014	carrier	Neisseria meningitidis	B	B	B	24	1	63	25	4	27	6	9	3	9	6	16	ST-41/44 complex	136	4	4	2
44451	OX40133	UK [England]	2014	carrier	Neisseria meningitidis	NG	cnl	cnl	102	156	1	2	NA	16	2	6	25	17	25	22	ST-53 complex	53	NA	NA	2
44454	OX40154	UK [England]	2014	carrier	Neisseria meningitidis	B	B	B	24	1	63	25	4	27	6	9	3	9	6	16	ST-41/44 complex	136	4	4	2
44456	OX40159	UK [England]	2014	carrier	Neisseria meningitidis	NG	cnl	cnl	1137	48	1	891	92	16	2	6	25	17	25	22	ST-53 complex	53	NA	NA	1
44457	OX40167	UK [England]	2014	carrier	Neisseria meningitidis	W	W	W	22	1	1	1	4	2	3	4	3	8	4	6	ST-11 complex	11	4	4	2
44458	OX40168	UK [England]	2014	carrier	Neisseria meningitidis	B	B	B	21	1	1	7	4	1	5	814	53	26	41	3	ST-162 complex	12234	4	4	2
44460	OX40199	UK [England]	2014	carrier	Neisseria meningitidis	NG	cnl	cnl	334	1	1	527	4	6	41	108	15	9	6	9		845	4	4	2
44461	OX40207	UK [England]	2014	carrier	Neisseria meningitidis	NG	NG	NG	19	1	1	65	4	28	6	9	35	9	6	9	ST-41/44 complex	12064	4	4	2
44462	OX40221	UK [England]	2015	carrier	Neisseria meningitidis	NG	Y	Y	25	1	1	26	4	10	5	18	9	11	9	17	ST-23 complex	23	4	4	2
52639	OX40224	UK [England]	2015	carrier	Neisseria meningitidis	NG	W	W	22	1	1	1	4	2	3	4	3	8	4	6	ST-11 complex	11	4	4	2
52640	OX40226	UK [England]	2014	carrier	Neisseria meningitidis	NG	W	W	22	1	1	1	4	2	3	4	3	8	4	6	ST-11 complex	11	4	4	2
52641	OX40264	UK [England]	2015	carrier	Neisseria meningitidis	NG	Y	Y	14	1	1	142	13	10	5	18	9	11	9	17	ST-23 complex	23	13	3	1
44466	OX40267	UK [England]	2015	carrier	Neisseria meningitidis	NG	cnl	cnl	94	157	56	69	NA	5	4	17	15	14	7	12	ST-198 complex	198	NA	NA	3
52642	OX40273	UK [England]	2015	carrier	Neisseria meningitidis	B	B	B	45	33	77	87	14	17	5	1	13	36	53	8	ST-213 complex	12011	NA	NA	3
44469	OX40288	UK [England]	2015	carrier	Neisseria meningitidis	NG	cnl	cnl	4	1	1	206	4	5	4	17	15	30	7	12	ST-198 complex	823	4	3	1
44470	OX40294	UK [England]	2015	carrier	Neisseria meningitidis	NG	cnl	cnl	4	1	1	206	4	5	4	17	15	30	7	12	ST-198 complex	823	4	3	1
52643	OX40300	UK [England]	2015	carrier	Neisseria meningitidis	NG	B	B	45	33	77	87	14	17	5	1	13	36	53	8	ST-213 complex	12011	NA	NA	3
44474	OX40325	UK [England]	2014	carrier	Neisseria meningitidis	NG	B	B	19	1	1	65	4	9	6	9	9	9	64	9	ST-41/44 complex	409	4	4	2
44475	OX40328	UK [England]	2014	carrier	Neisseria meningitidis	NG	Y	Y	25	1	1	26	4	12	5	18	9	11	9	17	ST-23 complex	1655	4	4	2
52644	OX40340	UK [England]	2014	carrier	Neisseria meningitidis	NG	B	B	45	8	77	87	20	7	5	1	13	36	53	15	ST-213 complex	213	20	4	3
44477	OX40363	UK [England]	2014	carrier	Neisseria meningitidis	NG	cnl	cnl	334	1	1	527	4	6	41	108	15	9	6	9		845	4	4	2
52645	OX40397	UK [England]	2015	carrier	Neisseria meningitidis	B	B	B	19	1	2	65	4	6	6	63	9	9	11	9	ST-41/44 complex	839	4	4	2
44479	OX40403	UK [England]	2015	carrier	Neisseria meningitidis	NG	Y	Y	23	1	1	4	4	2	7	159	92	93	6	8		6464	4	4	2
44482	OX40447	UK [England]	2015	carrier	Neisseria meningitidis	B	B	B	4	2	1	84	2	3	6	9	5	3	6	9	ST-41/44 complex	46	2	3	1
44483	OX40482	UK [England]	2015	carrier	Neisseria meningitidis	NG		NG	22	1	1	1	4	2	3	4	3	8	4	6	ST-11 complex	11	4	4	2
44484	OX40503	UK [England]	2015	carrier	Neisseria meningitidis	NG	cnl	cnl	102	8	10	2	20	16	2	6	25	17	25	22	ST-53 complex	53	20	4	2
44485	OX40516	UK [England]	2015	carrier	Neisseria meningitidis	Y	Y	Y	23	1	1	4	4	2	7	159	92	93	6	8		6464	4	4	2
52647	OX40533	UK [England]	2015	carrier	Neisseria meningitidis	Y	Y	Y	25	1	1	26	4	12	5	18	9	11	9	17	ST-23 complex	1655	4	4	2
44489	OX40625	UK [England]	2015	carrier	Neisseria meningitidis	NG	cnl	cnl	102	8	10	2	20	16	2	6	25	17	25	22	ST-53 complex	53	20	4	2
44490	OX40627	UK [England]	2015	carrier	Neisseria meningitidis	B	B	B	13	3	57	209	3	8	25	7	17	21	26	49	ST-1157 complex	1157	3	3	1
44491	OX40628	UK [England]	2015	carrier	Neisseria meningitidis	B	B	B	13	3	57	209	3	8	25	7	17	21	26	49	ST-1157 complex	1157	3	3	1
52649	OX40640	UK [England]	2015	carrier	Neisseria meningitidis	NG	B	B	45	8	77	87	20	7	5	1	13	36	53	15	ST-213 complex	213	20	4	3
52650	OX40657	UK [England]	2015	carrier	Neisseria meningitidis	NG	B	B	47	1	57	90	15	12	5	12	35	192	22	17	ST-461 complex	1946	15	4	3
44497	OX40666	UK [England]	2015	carrier	Neisseria meningitidis	NG	cnl	cnl	102	8	10	2	20	16	2	6	25	17	25	22	ST-53 complex	53	20	4	2
44498	OX40667	UK [England]	2015	carrier	Neisseria meningitidis	NG	cnl	cnl	102	8	1	2	20	16	2	6	25	17	25	22	ST-53 complex	53	20	4	2
52651	OX40672	UK [England]	2015	carrier	Neisseria meningitidis	B	B	B	45	8	1	87	20	637	5	1	13	36	53	15	ST-213 complex	10338	20	4	3
44500	OX40673	UK [England]	2015	carrier	Neisseria meningitidis	NG	E	E	13	3	57	209	3	8	25	7	17	21	26	49	ST-1157 complex	1157	3	3	1
52652	OX40674	UK [England]	2015	carrier	Neisseria meningitidis	Y	Y	Y	25	1	1	26	4	10	5	18	9	11	9	17	ST-23 complex	23	4	4	2
44502	OX40681	UK [England]	2015	carrier	Neisseria meningitidis	NG	B	B	23	250	1	431	NA	8	5	15	17	8	21	2	ST-865 complex	865	NA	NA	2
44503	OX40726	UK [England]	2015	carrier	Neisseria meningitidis	W	W	W	22	1	1	1	4	2	3	4	3	8	4	6	ST-11 complex	11	4	4	2
44504	OX40732	UK [England]	2015	carrier	Neisseria meningitidis	W	W	W	22	1	1	1	4	2	3	4	3	8	4	6	ST-11 complex	11	4	4	2
44507	OX40812	UK [England]	2015	carrier	Neisseria meningitidis	NG	Y	Y	25	1	1	26	4	12	5	18	25	11	9	17	ST-23 complex	9842	4	4	2
44509	OX40834	UK [England]	2015	carrier	Neisseria meningitidis	NG	cnl	cnl	4	1	1	206	4	5	4	17	15	30	7	12	ST-198 complex	823	4	3	1
44513	OX40860	UK [England]	2015	carrier	Neisseria meningitidis	NG	cnl	cnl	102	8	1	2	20	16	2	6	25	17	25	22	ST-53 complex	53	20	4	2
44514	OX40864	UK [England]	2015	carrier	Neisseria meningitidis	NG	E	E	13	3	57	209	3	8	25	7	17	21	26	49	ST-1157 complex	1157	3	3	1
44516	OX40902	UK [England]	2015	carrier	Neisseria meningitidis	NG	cnl	cnl	94	58	56	89	NA	5	4	38	15	784	40	13	ST-1136 complex	12147	NA	NA	3
44517	OX40905	UK [England]	2015	carrier	Neisseria meningitidis	NG	cnl	cnl	4	1	1	206	4	5	4	17	15	30	7	12	ST-198 complex	823	4	3	1
44519	OX40944	UK [England]	2015	carrier	Neisseria meningitidis	Y	Y	Y	104	1	57	10	4	10	5	18	9	22	9	17	ST-23 complex	10833	4	4	2
44520	OX40949	UK [England]	2015	carrier	Neisseria meningitidis	Y	Y	Y	25	1	1	26	4	12	5	18	9	11	9	17	ST-23 complex	1655	4	4	2
52654	OX40952	UK [England]	2015	carrier	Neisseria meningitidis	NG	NG	NG	13	3	1	145	3	4	10	47	17	6	2	15		12235	3	3	1
43804	OX40986	UK [England]	2015	carrier	Neisseria meningitidis	B	B	B	14	1	1	166	4	3	6	9	5	9	6	9	ST-41/44 complex	41	4	3	1
44523	OX41005	UK [England]	2015	carrier	Neisseria meningitidis	NG	cnl	cnl	102	8	1	2	20	16	2	6	25	17	25	22	ST-53 complex	53	20	4	2
44524	OX41014	UK [England]	2015	carrier	Neisseria meningitidis	B	B	B	19	1	1	93	13	12	6	9	17	9	6	9	ST-41/44 complex	206	13	4	2
44525	OX41015	UK [England]	2015	carrier	Neisseria meningitidis	NG	cnl	cnl	102	8	1	2	20	16	2	6	25	17	25	22	ST-53 complex	53	20	4	2
44526	OX41036	UK [England]	2015	carrier	Neisseria meningitidis	B	B	B	19	1	1	65	4	12	6	9	9	9	6	9	ST-41/44 complex	43	4	4	2
44527	OX41068	UK [England]	2015	carrier	Neisseria meningitidis	NG	cnl	cnl	4	1	1	206	4	5	4	17	15	30	7	12	ST-198 complex	823	4	3	1
52655	OX41085	UK [England]	2015	carrier	Neisseria meningitidis	B	B	B	22	1	1	1	4	7	5	1	13	36	53	15	ST-213 complex	213	4	4	2
44529	OX41086	UK [England]	2015	carrier	Neisseria meningitidis	NG	B	B	22	1	1	1	4	8	10	5	4	6	3	8	ST-32 complex	33	4	4	2
44530	OX41109	UK [England]	2015	carrier	Neisseria meningitidis	Y	Y	Y	25	1	1	26	4	12	5	18	9	11	9	17	ST-23 complex	1655	4	4	2
44531	OX41116	UK [England]	2015	carrier	Neisseria meningitidis	NG	cnl	cnl	4	1	1	206	4	5	4	17	15	30	7	12	ST-198 complex	823	4	3	1
44532	OX41125	UK [England]	2015	carrier	Neisseria meningitidis	NG	X	X	13	3	1	209	3	8	25	7	17	15	26	49	ST-1157 complex	12073	3	3	1
44533	OX41149	UK [England]	2015	carrier	Neisseria meningitidis	NG	cnl	cnl	102	8	10	2	20	16	2	6	25	17	25	22	ST-53 complex	53	20	4	2
44534	OX41162	UK [England]	2015	carrier	Neisseria meningitidis	B	B	B	19	1	1	93	13	12	6	9	17	9	6	9	ST-41/44 complex	206	13	4	2
44536	OX41178	UK [England]	2015	carrier	Neisseria meningitidis	NG	cnl	cnl	102	8	1	2	20	16	2	6	25	17	25	22	ST-53 complex	53	20	4	2
44537	OX41184	UK [England]	2015	carrier	Neisseria meningitidis	B	B	B	19	1	1	93	13	12	6	9	17	9	6	9	ST-41/44 complex	206	13	4	2
44538	OX41228	UK [England]	2015	carrier	Neisseria meningitidis	NG	cnl	cnl	102	8	1	2	20	16	2	6	25	17	25	22	ST-53 complex	53	20	4	2
44539	OX41230	UK [England]	2015	carrier	Neisseria meningitidis	Y	Y	Y	25	1	1	26	4	12	5	18	9	11	9	17	ST-23 complex	1655	4	4	2
44540	OX41234	UK [England]	2015	carrier	Neisseria meningitidis	B	B	B	19	1	1	65	4	9	20	9	9	9	6	2	ST-41/44 complex	1097	4	4	2
44541	OX41235	UK [England]	2015	carrier	Neisseria meningitidis	NG	cnl	cnl	4	1	1	206	4	5	4	17	15	30	7	12	ST-198 complex	823	4	3	1
44070	OX41236	UK [England]	2015	carrier	Neisseria meningitidis	NG	cnl	cnl	102	8	1	2	20	16	2	6	25	17	25	22	ST-53 complex	53	20	4	2
44543	OX41243	UK [England]	2015	carrier	Neisseria meningitidis	Y	Y	Y	23	1	1	4	4	2	7	6	9	16	9	8	ST-167 complex	767	4	4	2
44544	OX41244	UK [England]	2015	carrier	Neisseria meningitidis	Y	Y	Y	23	1	1	4	4	2	7	6	9	16	9	8	ST-167 complex	767	4	4	2
44545	OX41247	UK [England]	2015	carrier	Neisseria meningitidis	W	W	W	22	1	1	1	4	2	3	4	3	8	4	6	ST-11 complex	11	4	4	2
44547	OX41257	UK [England]	2015	carrier	Neisseria meningitidis	Y	Y	Y	23	1	1	4	4	2	7	6	9	16	9	8	ST-167 complex	767	4	4	2
44328	OX41261	UK [England]	2015	carrier	Neisseria meningitidis	B	B	B	19	1	1	93	13	12	6	9	17	9	6	9	ST-41/44 complex	206	13	4	2
44327	OX41274	UK [England]	2015	carrier	Neisseria meningitidis	Y	Y	Y	25	1	1	26	4	10	5	NA	9	11	9	17		NA	4	4	2
44550	OX41278	UK [England]	2015	carrier	Neisseria meningitidis	NG	B	B	47	1	57	90	15	12	5	12	35	60	22	17	ST-461 complex	461	15	4	3
44551	OX41306	UK [England]	2015	carrier	Neisseria meningitidis	Y	Y	Y	25	1	1	26	4	10	5	18	9	11	9	17	ST-23 complex	23	4	4	2
44552	OX41317	UK [England]	2015	carrier	Neisseria meningitidis	NG	E	E	13	3	57	209	3	8	25	7	17	21	26	49	ST-1157 complex	1157	3	3	1
44554	OX41324	UK [England]	2015	carrier	Neisseria meningitidis	NG	B	B	14	7	1	142	6	3	6	9	350	9	6	9	ST-41/44 complex	4893	6	2	1
52658	OX41354	UK [England]	2015	carrier	Neisseria meningitidis	NG	L	L	13	3	57	145	3	57	5	2	35	6	68	20		963	3	3	1
44557	OX41392	UK [England]	2015	carrier	Neisseria meningitidis	NG	B	B	101	1	57	13	4	4	10	2	740	11	3	8	ST-32 complex	11527	4	4	2

44559	OX41446	UK [England]	2015	carrier	Neisseria meningitidis	NG	cnl	cnl	21	1	1	7	4	16	2	159	92	77	25	112	ST-1117 complex	1117	4	4	2
44561	OX41460	UK [England]	2015	carrier	Neisseria meningitidis	Y	Y	Y	25	1	1	26	4	10	5	18	9	11	9	17	ST-23 complex	23	4	4	2
44562	OX41482	UK [England]	2015	carrier	Neisseria meningitidis	NG	E	E	13	3	57	145	3	2	16	12	11	3	60	7	ST-254 complex	254	3	3	1
44563	OX41486	UK [England]	2015	carrier	Neisseria meningitidis	NG	E	E	13	3	57	145	3	2	16	12	11	3	60	7	ST-254 complex	254	3	3	1
44564	OX41493	UK [England]	2015	carrier	Neisseria meningitidis	Y	Y	Y	25	1	1	26	4	12	5	18	9	11	9	17	ST-23 complex	1655	4	4	2
44565	OX41498	UK [England]	2015	carrier	Neisseria meningitidis	B	B	B	45	8	77	87	20	7	5	1	13	36	53	15	ST-213 complex	213	20	4	3
44566	OX41501	UK [England]	2015	carrier	Neisseria meningitidis	NG	cnl	cnl	334	1	1	527	4	6	41	108	15	9	6	9		845	4	4	2
44568	OX41504	UK [England]	2015	carrier	Neisseria meningitidis	Y	Y	Y	911	5	1	710	60	12	5	18	9	11	9	17	ST-23 complex	1655	NA	NA	1
44570	OX41508	UK [England]	2015	carrier	Neisseria meningitidis	NG	cnl	cnl	334	1	1	527	4	6	41	108	15	9	6	9		845	4	4	2
44571	OX41521	UK [England]	2015	carrier	Neisseria meningitidis	NG	E	E	13	3	1	145	3	17	5	19	17	3	26	2	ST-60 complex	60	3	3	1
44572	OX41538	UK [England]	2015	carrier	Neisseria meningitidis	NG	cnl	cnl	102	8	10	2	20	782	2	6	25	17	25	22	ST-53 complex	12356	20	4	2
44574	OX41571	UK [England]	2015	carrier	Neisseria meningitidis	Y	Y	Y	25	1	1	26	4	12	5	18	9	11	9	17	ST-23 complex	1655	4	4	2
44575	OX41580	UK [England]	2015	carrier	Neisseria meningitidis	W	W	W	22	1	1	1	4	2	3	4	3	8	4	6	ST-11 complex	11	4	4	2
44576	OX41582	UK [England]	2015	carrier	Neisseria meningitidis	NG	B	B	19	1	1	65	4	9	6	9	9	9	64	9	ST-41/44 complex	409	4	4	2
44633	OX41588	UK [England]	2015	carrier	Neisseria meningitidis	NG	Y	Y	25	1	1	26	4	12	5	18	9	11	9	17	ST-23 complex	1655	4	4	2
44577	OX41591	UK [England]	2015	carrier	Neisseria meningitidis	NG	Y	Y	104	1	57	10	4	10	5	18	9	11	9	17	ST-23 complex	23	4	4	2
44578	OX41593	UK [England]	2015	carrier	Neisseria meningitidis	Y	Y	Y	25	1	1	26	4	12	5	18	9	11	9	17	ST-23 complex	1655	4	4	2
44579	OX41676	UK [England]	2015	carrier	Neisseria meningitidis	NG	X	X	104	1	57	10	4	132	10	832	17	62	21	2		12359	4	4	2
44580	OX41679	UK [England]	2015	carrier	Neisseria meningitidis	NG	cnl	cnl	102	8	10	2	20	16	2	6	25	17	25	22	ST-53 complex	53	20	4	2
44581	OX41682	UK [England]	2015	carrier	Neisseria meningitidis	NG	E	E	13	3	57	209	3	8	25	7	17	21	26	49	ST-1157 complex	1157	3	3	1
44582	OX41686	UK [England]	2015	carrier	Neisseria meningitidis	NG	NG	NG	19	1	1	65	4	17	6	9	9	9	64	9	ST-41/44 complex	12360	4	4	2
44583	OX41687	UK [England]	2015	carrier	Neisseria meningitidis	NG	NG	NG	19	1	1	65	4	17	6	9	9	9	64	9	ST-41/44 complex	12360	4	4	2
44586	OX41700	UK [England]	2015	carrier	Neisseria meningitidis	NG	B	B	19	1	1	65	4	9	6	9	9	9	64	9	ST-41/44 complex	409	4	4	2
44587	OX41703	UK [England]	2015	carrier	Neisseria meningitidis	Y	Y	Y	25	1	1	26	4	12	5	18	9	11	9	17	ST-23 complex	1655	4	4	2
44588	OX41706	UK [England]	2015	carrier	Neisseria meningitidis	Y	Y	Y	25	1	1	26	4	12	5	18	9	11	9	17	ST-23 complex	1655	4	4	2
44589	OX41707	UK [England]	2015	carrier	Neisseria meningitidis	Y	Y	Y	25	1	1	26	4	12	5	18	9	11	9	17	ST-23 complex	1655	4	4	2
44591	OX41723	UK [England]	2015	carrier	Neisseria meningitidis	NG	cnl	cnl	15	4	3	193	1	8	10	5	4	5	3	8	ST-32 complex	34	1	1	1
44592	OX41725	UK [England]	2015	carrier	Neisseria meningitidis	Y	Y	Y	25	1	1	26	4	12	5	18	9	11	9	17	ST-23 complex	1655	4	4	2
44593	OX41741	UK [England]	2015	carrier	Neisseria meningitidis	NG	NG	NG	19	1	1	65	4	17	6	9	9	9	64	9	ST-41/44 complex	12360	4	4	2
44595	OX41779	UK [England]	2015	carrier	Neisseria meningitidis	W	W	W	22	1	1	1	4	2	3	4	3	8	4	6	ST-11 complex	11	4	4	2
44596	OX41788	UK [England]	2015	carrier	Neisseria meningitidis	NG	cnl	cnl	102	8	10	2	20	16	2	6	25	17	25	22	ST-53 complex	53	20	4	2
44598	OX41792	UK [England]	2015	carrier	Neisseria meningitidis	NG	E	E	13	3	57	209	3	8	25	7	17	21	26	49	ST-1157 complex	1157	3	3	1
44599	OX41807	UK [England]	2015	carrier	Neisseria meningitidis	Y	Y	Y	24	1	1	27	4	2	7	6	17	9	503	8	ST-167 complex	9579	4	4	2
44600	OX41808	UK [England]	2015	carrier	Neisseria meningitidis	NG	E	E	13	3	57	209	3	8	25	7	17	21	26	49	ST-1157 complex	1157	3	3	1
44601	OX41820	UK [England]	2015	carrier	Neisseria meningitidis	NG	cnl	cnl	102	8	10	2	20	16	2	6	25	17	25	22	ST-53 complex	53	20	4	2
44602	OX41822	UK [England]	2015	carrier	Neisseria meningitidis	NG	B	B	21	1	1	7	4	1	5	13	53	26	41	3	ST-162 complex	162	4	4	2
44603	OX41843	UK [England]	2015	carrier	Neisseria meningitidis	NG	cnl	cnl	102	8	10	2	20	16	2	6	25	17	25	22	ST-53 complex	53	20	4	2
44604	OX41866	UK [England]	2015	carrier	Neisseria meningitidis	Y	Y	Y	25	1	1	26	4	12	5	18	9	11	9	17	ST-23 complex	1655	4	4	2
44608	OX41936	UK [England]	2015	carrier	Neisseria meningitidis	W	W	W	22	4	2	715	1	2	3	4	3	8	4	6	ST-11 complex	11	1	1	2
44609	OX41953	UK [England]	2015	carrier	Neisseria meningitidis	NG	E	E	13	3	57	209	3	8	25	7	17	21	26	49	ST-1157 complex	1157	3	3	1
44610	OX41955	UK [England]	2015	carrier	Neisseria meningitidis	NG	cnl	cnl	102	8	10	2	20	16	2	18	25	17	25	22	ST-53 complex	2441	20	4	2
44612	OX41960	UK [England]	2015	carrier	Neisseria meningitidis	Y	Y	Y	16	1	1	60	4	219	5	4	17	11	8	21		5436	4	4	2
44614	OX41981	UK [England]	2015	carrier	Neisseria meningitidis	Y	Y	Y	25	1	1	26	4	10	5	18	9	11	9	17	ST-23 complex	23	4	4	2
44617	OX42015	UK [England]	2015	carrier	Neisseria meningitidis	NG	cnl	cnl	94	58	56	89	NA	5	4	38	15	22	40	13	ST-1136 complex	1136	NA	NA	3
44618	OX42040	UK [England]	2015	carrier	Neisseria meningitidis	NG	E	E	14	7	57	142	6	8	25	7	17	21	26	49	ST-1157 complex	1157	6	2	1
44619	OX42047	UK [England]	2015	carrier	Neisseria meningitidis	Y	Y	Y	25	1	1	26	4	12	5	18	9	11	9	17	ST-23 complex	1655	4	4	2
44621	OX42050	UK [England]	2015	carrier	Neisseria meningitidis	NG	E	E	13	3	1	145	3	12	5	19	17	3	10	124	ST-60 complex	1754	3	3	1
44623	OX42063	UK [England]	2015	carrier	Neisseria meningitidis	NG	cnl	cnl	4	1	1	206	4	5	4	17	15	30	7	12	ST-198 complex	823	4	3	1
44624	OX42066	UK [England]	2015	carrier	Neisseria meningitidis	Y	Y	Y	25	1	1	26	4	12	5	18	9	11	9	17	ST-23 complex	1655	4	4	2
44625	OX42120	UK [England]	2015	carrier	Neisseria meningitidis	NG	Y	Y	25	1	1	26	4	10	5	18	9	11	9	17	ST-23 complex	23	4	4	2
44626	OX42130	UK [England]	2015	carrier	Neisseria meningitidis	Y	Y	Y	25	1	1	26	4	10	5	18	9	11	9	17	ST-23 complex	23	4	4	2
44627	OX42147	UK [England]	2015	carrier	Neisseria meningitidis	NG	Y	Y	104	1	57	10	4	10	5	18	9	11	9	17	ST-23 complex	23	4	4	2
44628	OX42148	UK [England]	2015	carrier	Neisseria meningitidis	Y	Y	Y	25	1	1	26	4	10	5	18	9	11	9	17	ST-23 complex	23	4	4	2
44629	OX42149	UK [England]	2015	carrier	Neisseria meningitidis	NG	E	E	13	3	57	209	3	8	555	7	17	433	26	49	ST-1157 complex	12364	3	3	1
44439	OX42156	UK [England]	2015	carrier	Neisseria meningitidis	Y	Y	Y	104	1	57	10	4	10	5	18	9	11	9	17	ST-23 complex	23	4	4	2
44632	OX42197	UK [England]	2015	carrier	Neisseria meningitidis	Y	Y	Y	25	1	1	26	4	12	5	18	9	11	9	17	ST-23 complex	1655	4	4	2
56881	OX9930005	UK [England]	1999	carrier	Neisseria meningitidis	Y	Y	Y	25	1	1	26	4	10	5	18	9	11	9	17	ST-23 complex	23	4	4	2
56884	OX9930019	UK [England]	1999	carrier	Neisseria meningitidis	B	B	B	90	NA	118	1005	193	4	3	11	18	6	10	12	ST-35 complex	160	NA	NA	1
28850	OX9930030	UK [England]	1999	carrier	Neisseria meningitidis	E	B	discrepancy	338	7	1	NA	6	3	6	9	5	9	6	9	ST-41/44 complex	41	6	2	1
56887	OX9930033	UK [England]	1999	carrier	Neisseria meningitidis	NG	cnl	cnl	102	8	1	2	20	16	2	6	25	17	25	22	ST-53 complex	53	20	4	2
57039	OX9930036	UK [England]	1999	carrier	Neisseria meningitidis	B	B	B	19	1	1	65	4	12	6	9	9	9	6	9	ST-41/44 complex	43	4	4	2
57040	OX9930040	UK [England]	1999	carrier	Neisseria meningitidis	B	B	B	13	3	57	145	3	4	10	NA	5	38	11	9		NA	3	3	1
872	OX9930042	UK [England]	1999	carrier	Neisseria meningitidis	E	cnl	discrepancy	94	58	56	89	NA	11	4	38	15	22	40	13	ST-1136 complex	1630	NA	NA	3
56892	OX9930044	UK [England]	1999	carrier	Neisseria meningitidis	NG	Y	Y	19	1	1	93	13	2	16	6	17	9	18	8	ST-167 complex	168	13	4	2
873	OX9930046	UK [England]	1999	carrier	Neisseria meningitidis	B	B	B	45	8	77	87	20	NA	NA	NA	NA	NA	NA	NA		NA	20	4	3
874	OX9930056	UK [England]	1999	carrier	Neisseria meningitidis	B	B	B	13	3	57	145	3	NA	NA	NA	NA	NA	NA	NA		NA	3	3	1
5564	OX9930081	UK [England]	1999	carrier	Neisseria meningitidis	NG	E	E	13	3	1	145	3	17	5	19	17	3	26	2	ST-60 complex	60	3	3	1
3387	OX9930084	UK [England]	1999	carrier	Neisseria meningitidis	NG	W	W	16	1	1	60	4	11	5	5	8	11	24	21	ST-22 complex	1617	4	4	2
876	OX9930086	UK [England]	1999	carrier	Neisseria meningitidis	E	E	E	13	3	1	145	3	17	5	19	17	3	26	2	ST-60 complex	60	3	3	1
877	OX9930092	UK [England]	1999	carrier	Neisseria meningitidis	Y	Y	Y	19	1	1	93	13	2	16	6	17	9	18	8	ST-167 complex	168	13	4	2
878	OX9930097	UK [England]	1999	carrier	Neisseria meningitidis	NG	NG	NG	23	1	1	4	4	9	7	6	17	11	18	17	ST-167 complex	1636	4	4	2
28851	OX9930099	UK [England]	1999	carrier	Neisseria meningitidis	B	B	B	19	1	1	65	4	9	20	9	9	9	6	2	ST-41/44 complex	1097	4	4	2
56901	OX9930101	UK [England]	1999	carrier	Neisseria meningitidis	C	C	C	201	1	1	23	4	2	3	4	3	8	4	6	ST-11 complex	11	4	4	2
3388	OX9930102	UK [England]	1999	carrier	Neisseria meningitidis	NG	W	W	22	1	1	1	4	11	5	9	17	11	24	21	ST-22 complex	1221	4	4	2
35890	OX9930103	UK [England]	1999	carrier	Neisseria meningitidis		W	W	22	1	1	1	4	11	5	9	17	11	24	21	ST-22 complex	1221	4	4	2
56908	OX9930104	UK [England]	1999	carrier	Neisseria meningitidis	B	B	B	45	8	77	87	20	7	5	2	13	36	53	15	ST-213 complex	4549	20	4	3
56910	OX9930107	UK [England]	1999	carrier	Neisseria meningitidis	NG	cnl	cnl	102	8	10	2	20	16	2	6	25	17	25	22	ST-53 complex	53	20	4	2
56863	OX9930110	UK [England]	1999	carrier	Neisseria meningitidis	B	B	B	90	NA	118	1005	193	4	3	11	18	6	10	12	ST-35 complex	160	NA	NA	1
56903	OX9930114	UK [England]	1999	carrier	Neisseria meningitidis	C	C	C	19	1	1	65	4	13	5	15	9	3	8	18	ST-334 complex	1031	4	4	2
56913	OX9930118	UK [England]	1999	carrier	Neisseria meningitidis	NG	B	B	106	1	2	146	4	4	10	2	161	8	11	20	ST-269 complex	2369	4	4	2
879	OX9930124	UK [England]	1999	carrier	Neisseria meningitidis	Y	Y	Y	23	1	1	4	4	8	7	6	17	9	18	8	ST-167 complex	279	4	4	2
56916	OX9930125	UK [England]	1999	carrier	Neisseria meningitidis	NG	C	C	19	1	1	65	4	8	26	9	24	26	20	18	ST-212 complex	212	4	4	2
880	OX9930126	UK [England]	1999	carrier	Neisseria meningitidis	B	W	discrepancy	16	1	1	60	4	11	5	18	8	11	4	21	ST-22 complex	184	4	4	2
881	OX9930132	UK [England]	1999	carrier	Neisseria meningitidis	B	B	B	45	8	77	87	20	7	5	1	13	36	53	15	ST-213 complex	213	20	4	3
56905	OX9930134	UK [England]	1999	carrier	Neisseria meningitidis	NG	cnl	cnl	94	157	56	69	NA	5	4	17	15	14	7	16	ST-198 complex	39	NA	NA	3
882	OX9930136	UK [England]	1999	carrier	Neisseria meningitidis	B	B	B	15	4	2	193	1	4	10	15	9	8	11	9	ST-269 complex	269	1	1	1
4314	OX9930141	UK [England]	1999	carrier	Neisseria meningitidis	B	B	B	24	1	1	25	4	9	6	9	9	9	6	2	ST-41/44 complex	180	4	4	2
883	OX9930142	UK [England]	1999	carrier	Neisseria meningitidis	B	W	discrepancy	16	1	1	60	4	11	5	18	8	11	4	21	ST-22 complex	184	4	4	2
56920	OX9930148	UK [England]	1999	carrier	Neisseria meningitidis	NG	cnl	cnl	21	1	1	7	4	16	2	159	92	77	25	112	ST-1117 complex	1117	4	4	2
884	OX9930150	UK [England]	1999	carrier	Neisseria meningitidis	B	cnl	discrepancy	21	1	1	7	4	16	2	159	92	77	25	112	ST-1117 complex	1117	4	4	2
885	OX9930151	UK [England]	1999	carrier	Neisseria meningitidis	B	B	B	14	1	1	142	13	2	6	9	5	9	6	156	ST-41/44 complex	13201	13	3	1
56922	OX9930161	UK [England]	1999	carrier	Neisseria meningitidis	NG	C	C	201	1	1	23	4	2	3	4	3	8	4	6	ST-11 complex	11	4	4	2
3389	OX9930167	UK [England]	1999	carrier	Neisseria meningitidis	NG	E	E	13	3	1	145	3	17	5	19	17	3	26	2	ST-60 complex	60	3	3	1
56923	OX9930170	UK [England]	1999	carrier	Neisseria meningitidis	B	B	B	22	1	1	1	4	4	6	2	26	8	11	20		793	4	4	2
56927	OX9930178	UK [England]	1999	carrier	Neisseria meningitidis	B	NG	discrepancy	23	1	1	4	4	2	7	6	17	9	6	8	ST-167 complex	1624	4	4	2
56929	OX9930179	UK [England]	1999	carrier	Neisseria meningitidis	E	E	E	13	3	1	145	3	17	5	19	17	3	26	2	ST-60 complex	60	3	3	1
4218	OX9930181	UK [England]	1999	carrier	Neisseria meningitidis	B	B	B	16	158	113	NA	163	4	16	161	9	6	21	3		13203	NA	NA	2
56925	OX9930198	UK [England]	1999	carrier	Neisseria meningitidis	NG	Y	Y	25	1	1	26	4	10	5	18	9	11	9	17	ST-23 complex	23	4	4	2
56906	OX9930200	UK [England]	1999	carrier	Neisseria meningitidis	B	B	B	45	8	77	87	20	7	5	1	13	36	53	15	ST-213 complex	213	20	4	3
886	OX9930203	UK [England]	1999	carrier	Neisseria meningitidis	B	B	B	187	1	62	77	4	7	5	4	13	36	6	15	ST-213 complex	1644	4	4	3
56932	OX9930211	UK [England]	1999	carrier	Neisseria meningitidis	E	E	E	13	3	1	145	3	17	5	19	17	3	26	2	ST-60 complex	60	3	3	1
56934	OX9930218	UK [England]	1999	carrier	Neisseria meningitidis	E	E	E	13	3	1	145	3	17	5	19	17	3	26	2	ST-60 complex	60	3	3	1
56936	OX9930228	UK [England]	1999	carrier	Neisseria meningitidis	E	E	E	13	3	1	145	3	17	5	19	17	3	26	2	ST-60 complex	60	3	3	1
56790	OX9930234	UK [England]	1999	carrier	Neisseria meningitidis	E	E	E	13	3	1	145	3	17	5	47	17	3	26	2	ST-60 complex	1430	3	3	1
56792	OX9930239	UK [England]	1999	carrier	Neisseria meningitidis	E	E	E	13	3	1	145	3	17	5	19	17	3	26	2	ST-60 complex	60	3	3	1
56866	OX9930240	UK [England]	1999	carrier	Neisseria meningitidis	NG	W	W	16	1	1	60	4	11	5	18	8	78	24	21	ST-22 complex	1224	4	4	2
35887	OX9930244	UK [England]	1999	carrier	Neisseria meningitidis		cnl	cnl	102	8	1	2	20	16	2	6	25	17	25	22	ST-53 complex	53	20	4	2
56794	OX9930245	UK [England]	1999	carrier	Neisseria meningitidis	NG	E	E	13	3	1	145	3	17	5	19	17	3	26	2	ST-60 complex	60	3	3	1
56796	OX9930251	UK [England]	1999	carrier	Neisseria meningitidis	NG	Y	Y	19	1	1	93	13	2	16	6	17	9	18	8	ST-167 complex	168	13	4	2
56798	OX9930252	UK [England]	1999	carrier	Neisseria meningitidis	E	E	E	13	3	1	145	3	17	5	19	17	3	26	2	ST-60 complex	60	3	3	1
56800	OX9930253	UK [England]	1999	carrier	Neisseria meningitidis	E	E	E	13	3	1	145	3	17	5	19	17	3	26	2	ST-60 complex	60	3	3	1
56802	OX9930254	UK [England]	1999	carrier	Neisseria meningitidis	NG	cnl	cnl	94	138	56	69	NA	5	4	17	15	14	7	12	ST-198 complex	198	NA	NA	3
56804	OX9930260	UK [England]	1999	carrier	Neisseria meningitidis	B	B	B	21	1	1	7	4	4	10	5	4	6	3	8	ST-32 complex	32	4	4	2
56805	OX9930261	UK [England]	1999	carrier	Neisseria meningitidis	B	B	B	45	8	77	87	20	7	5	1	13	36	53	15	ST-213 complex	213	20	4	3
56807	OX9930289	UK [England]	1999	carrier	Neisseria meningitidis	NG	Y	Y	19	1	1	93	13	2	16	6	17	9	18	8	ST-167 complex	168	13	4	2
56907	OX9930292	UK [England]	1999	carrier	Neisseria meningitidis	E	E	E	13	3	1	145	3	17	5	19	17	3	26	2	ST-60 complex	60	3	3	1
35894	OX9930296	UK [England]	1999	carrier	Neisseria meningitidis		cnl	cnl	94	138	56	69	NA	5	4	17	15	14	7	12	ST-198 complex	198	NA	NA	3
56809	OX9930298	UK [England]	1999	carrier	Neisseria meningitidis	NG	E	E	13	3	1	145	3	17	5	19	17	3	26	2	ST-60 complex	60	3	3	1
14745	OX9930304	UK [England]	1999	carrier	Neisseria meningitidis	B	B	B	691	1	1	404	4	12	6	9	9	9	6	9	ST-41/44 complex	43	4	4	2
56811	OX9930310	UK [England]	1999	carrier	Neisseria meningitidis	NG	cnl	cnl	94	138	56	69	NA	5	4	17	15	14	7	12	ST-198 complex	198	NA	NA	3
56813	OX9930311	UK [England]	1999	carrier	Neisseria meningitidis	E	E	E	13	3	1	145	3	17	5	19	17	3	26	2	ST-60 complex	60	3	3	1
56815	OX9930314	UK [England]	1999	carrier	Neisseria meningitidis	NG	cnl	cnl	102	8	1	2	20	16	2	6	25	17	25	22	ST-53 complex	53	20	4	2
56817	OX9930317	UK [England]	1999	carrier	Neisseria meningitidis	NG	B	B	284	1	1	NA	4	20	3	13	93	5	155	77		1457	4	4	2
56818	OX9930326	UK [England]	1999	carrier	Neisseria meningitidis	NG	B	B	119	1	1	98	4	87	10	105	2	5	13	8		1459	4	4	2
56819	OX9930338	UK [England]	1999	carrier	Neisseria meningitidis	NG	E	E	25	1	1	26	4	8	6	15	17	8	21	2	ST-865 complex	1445	4	4	2
14746	OX9930342	UK [England]	1999	carrier	Neisseria meningitidis	B	B	B	13	3	57	NA	3	20	6	63	9	9	515	9	ST-41/44 complex	8095	3	3	1
56820	OX9930346	UK [England]	1999	carrier	Neisseria meningitidis	NG	B	B	19	1	1	65	4	4	10	34	7	8	18	1		13258	4	4	2
887	OX9930357	UK [England]	1999	carrier	Neisseria meningitidis	NG	B	B	19	1	1	65	4	12	6	9	9	9	6	9	ST-41/44 complex	43	4	4	2
888	OX9930372	UK [England]	1999	carrier	Neisseria meningitidis	NG	Z	Z	25	1	1	26	4	8	3	6	17	29	18	9	ST-103 complex	1418	4	4	2
3390	OX9930377	UK [England]	1999	carrier	Neisseria meningitidis	NG	cnl	cnl	102	8	1	2	20	16	2	6	25	17	25	22	ST-53 complex	53	20	4	2
889	OX9930386	UK [England]	1999	carrier	Neisseria meningitidis	NG	NG	NG	16	1	1	477	195	11	5	18	8	11	24	21	ST-22 complex	22	NA	NA	2
35891	OX9930387	UK [England]	1999	carrier	Neisseria meningitidis		W	W	138	5	1	112	11	11	5	18	8	11	24	21	ST-22 complex	22	11	2	2
56826	OX9930390	UK [England]	1999	carrier	Neisseria meningitidis	NG	NG	NG	15	4	2	193	1	4	10	15	9	8	11	13	ST-269 complex	467	1	1	1
56827	OX9930397	UK [England]	1999	carrier	Neisseria meningitidis	NG	B	B	19	1	1	83	4	4	10	2	5	38	11	9	ST-269 complex	275	4	4	2
890	OX9930407	UK [England]	1999	carrier	Neisseria meningitidis	NG	cnl	cnl	285	1	1	5	4	12	91	6	181	177	186	8		2121	4	4	2
56869	OX9930417	UK [England]	1999	carrier	Neisseria meningitidis	NG	W	W	16	1	1	60	4	11	5	18	8	11	4	21	ST-22 complex	184	4	4	2
3391	OX9930427	UK [England]	1999	carrier	Neisseria meningitidis	NG	L	L	13	3	57	145	3	57	5	2	35	6	68	20		963	3	3	1
56828	OX9930433	UK [England]	1999	carrier	Neisseria meningitidis	B	B	B	141	1	4	116	4	8	5	19	17	11	24	54		1052	4	4	3

28852	OX9930434	UK [England]	1999	carrier	Neisseria meningitidis	NG	B	B	4	2	1	84	2	3	6	9	5	9	6	9	ST-41/44 complex	41	2	3	1
56872	OX9930440	UK [England]	1999	carrier	Neisseria meningitidis	W	W	W	16	1	1	60	4	11	5	18	8	11	4	21	ST-22 complex	184	4	4	2
56830	OX9930443	UK [England]	1999	carrier	Neisseria meningitidis	NG	E	E	31	9	57	58	16	23	3	4	25	3	22	7		1431	16	2	3
56832	OX9930445	UK [England]	1999	carrier	Neisseria meningitidis	NG	E	E	13	3	57	209	3	8	25	7	17	21	26	49	ST-1157 complex	1157	3	3	1
56833	OX9930459	UK [England]	1999	carrier	Neisseria meningitidis	E	E	E	13	3	1	145	3	17	5	19	17	3	26	2	ST-60 complex	60	3	3	1
891	OX9930467	UK [England]	1999	carrier	Neisseria meningitidis	NG	E	E	13	3	57	209	3	8	25	7	17	21	26	17	ST-1157 complex	1649	3	3	1
3392	OX9930468	UK [England]	1999	carrier	Neisseria meningitidis	NG	B	B	144	1	4	232	4	8	10	5	4	6	3	8	ST-32 complex	33	4	3	1
892	OX9930476	UK [England]	1999	carrier	Neisseria meningitidis	E	E	E	13	3	1	145	3	17	5	19	5	3	26	2	ST-60 complex	1650	3	3	1
56836	OX9930485	UK [England]	1999	carrier	Neisseria meningitidis	C	C	C	22	1	1	1	4	2	3	4	3	8	20	6	ST-11 complex	1410	4	4	2
28853	OX9930486	UK [England]	1999	carrier	Neisseria meningitidis	B	B	B	19	1	1	65	4	9	6	9	9	9	6	9	ST-41/44 complex	44	4	4	2
28854	OX9930499	UK [England]	1999	carrier	Neisseria meningitidis	B	B	B	19	1	1	333	4	3	6	9	24	9	38	9	ST-41/44 complex	1414	4	4	2
56837	OX9930509	UK [England]	1999	carrier	Neisseria meningitidis	E	E	E	13	3	57	209	3	8	25	7	17	21	26	49	ST-1157 complex	1157	3	3	1
4229	OX9930513	UK [England]	1999	carrier	Neisseria meningitidis	B	B	B	24	1	2	25	4	4	10	11	17	6	20	12	ST-35 complex	3074	4	4	2
56840	OX9930515	UK [England]	1999	carrier	Neisseria meningitidis	B	B	B	276	26	4	316	100	4	10	11	18	6	10	12	ST-35 complex	35	NA	NA	1
3393	OX9930516	UK [England]	1999	carrier	Neisseria meningitidis	NG	cnl	cnl	21	1	1	7	4	16	2	159	92	182	25	112	ST-1117 complex	2445	4	4	2
56843	OX9930517	UK [England]	1999	carrier	Neisseria meningitidis	B	B	B	45	8	77	87	20	7	5	1	13	36	53	15	ST-213 complex	213	20	4	3
56845	OX9930520	UK [England]	1999	carrier	Neisseria meningitidis	NG	cnl	cnl	102	8	1	2	20	16	2	6	25	17	25	22	ST-53 complex	53	20	4	2
56847	OX9930525	UK [England]	1999	carrier	Neisseria meningitidis	B	B	B	21	1	1	7	4	1	5	13	53	26	41	3	ST-162 complex	162	4	4	2
35893	OX9930530	UK [England]	1999	carrier	Neisseria meningitidis		Y	Y	16	1	1	60	4	11	5	18	8	11	24	21	ST-22 complex	22	4	4	2
56849	OX9930533	UK [England]	1999	carrier	Neisseria meningitidis	E	E	E	13	3	1	145	3	17	5	19	9	3	26	2	ST-60 complex	466	3	3	1
56850	OX9930534	UK [England]	1999	carrier	Neisseria meningitidis	NG	cnl	cnl	102	8	1	2	20	16	2	6	25	17	25	22	ST-53 complex	53	20	4	2
28855	OX9930539	UK [England]	1999	carrier	Neisseria meningitidis	B	B	B	24	1	2	25	4	135	6	9	3	9	6	6	ST-41/44 complex	1473	4	4	2
56851	OX9930547	UK [England]	1999	carrier	Neisseria meningitidis	C	C	C	1106	5	1	608	11	2	3	4	3	8	4	6	ST-11 complex	11	11	2	1
4381	OX9930552	UK [England]	1999	carrier	Neisseria meningitidis	NG	E	E	13	3	57	145	3	17	5	19	11	3	22	9	ST-60 complex	3231	3	3	1
56854	OX9930563	UK [England]	1999	carrier	Neisseria meningitidis	B	B	B	25	1	1	26	4	8	4	6	17	5	18	2	ST-103 complex	103	4	4	2
56857	OX9930564	UK [England]	1999	carrier	Neisseria meningitidis	NG	B	B	286	1	57	411	4	8	16	88	143	3	6	9		1420	4	4	2
56859	OX9930565	UK [England]	1999	carrier	Neisseria meningitidis	NG	Z	Z	25	1	1	26	4	8	3	6	17	29	18	9	ST-103 complex	1418	4	4	2
56860	OX9930569	UK [England]	1999	carrier	Neisseria meningitidis	NG	Z	Z	25	1	1	26	4	8	3	6	17	29	18	9	ST-103 complex	1418	4	4	2
3394	OX9930573	UK [England]	1999	carrier	Neisseria meningitidis	NG	B	B	19	1	1	65	4	12	2	9	9	5	54	10		13202	4	4	2
28856	OX9930579	UK [England]	1999	carrier	Neisseria meningitidis	B	B	B	24	1	1	25	4	9	6	9	9	9	6	2	ST-41/44 complex	180	4	4	2
56862	OX9930581	UK [England]	1999	carrier	Neisseria meningitidis	C	C	C	22	1	1	1	4	2	3	4	3	8	20	6	ST-11 complex	1410	4	4	2
56864	OX9930593	UK [England]	1999	carrier	Neisseria meningitidis	NG	Z	Z	25	1	1	26	4	8	4	6	17	5	18	2	ST-103 complex	103	4	4	2
56875	OX9930602	UK [England]	1999	carrier	Neisseria meningitidis	NG	W	W	16	1	1	60	4	11	5	18	8	78	24	21	ST-22 complex	1224	4	4	2
56867	OX9930604	UK [England]	1999	carrier	Neisseria meningitidis	B	B	B	202	1	1	35	4	42	26	46	24	6	20	17	ST-282 complex	1802	4	4	2
56870	OX9930605	UK [England]	1999	carrier	Neisseria meningitidis	B	B	B	13	3	57	145	3	4	10	2	5	38	11	9	ST-269 complex	275	3	3	1
56877	OX9930611	UK [England]	1999	carrier	Neisseria meningitidis	NG	W	W	16	1	1	60	4	11	5	18	8	78	24	21	ST-22 complex	1224	4	4	2
56873	OX9930612	UK [England]	1999	carrier	Neisseria meningitidis	NG	cnl	cnl	94	138	56	69	NA	5	4	17	15	14	7	12	ST-198 complex	198	NA	NA	3
14588	OX9930614	UK [England]	1999	carrier	Neisseria meningitidis	NG	C	C	19	1	1	65	4	8	26	9	24	26	20	18	ST-212 complex	212	4	4	2
35892	OX9930617	UK [England]	1999	carrier	Neisseria meningitidis		W	W	138	5	1	112	11	11	5	18	8	11	24	21	ST-22 complex	22	11	2	2
893	OX9930621	UK [England]	1999	carrier	Neisseria meningitidis	B	B	B	16	1	4	59	4	4	10	11	17	6	10	12	ST-35 complex	457	4	4	2
56880	OX9930644	UK [England]	1999	carrier	Neisseria meningitidis	NG	E	E	125	1	57	268	13	7	16	6	10	3	56	13	ST-178 complex	2392	13	3	1
56883	OX9930648	UK [England]	1999	carrier	Neisseria meningitidis	NG	Y	Y	19	1	1	93	13	2	16	6	17	9	18	8	ST-167 complex	168	13	4	2
56886	OX9930650	UK [England]	1999	carrier	Neisseria meningitidis	E	E	E	13	3	1	145	3	12	2	19	17	3	26	2	ST-60 complex	913	3	3	1
56889	OX9930654	UK [England]	1999	carrier	Neisseria meningitidis	NG	cnl	cnl	285	1	1	5	4	12	91	6	181	177	186	8		2121	4	4	2
56891	OX9930656	UK [England]	1999	carrier	Neisseria meningitidis	NG	E	E	13	3	57	209	3	8	25	7	17	21	26	49	ST-1157 complex	1157	3	3	1
4353	OX9930668	UK [England]	1999	carrier	Neisseria meningitidis	NG	E	E	1104	3	57	NA	3	8	25	7	17	21	26	49	ST-1157 complex	1157	3	3	1
56879	OX9930670	UK [England]	1999	carrier	Neisseria meningitidis	NG	W	W	16	1	1	60	4	11	5	18	8	11	4	21	ST-22 complex	184	4	4	2
56894	OX9930678	UK [England]	1999	carrier	Neisseria meningitidis	NG	cnl	cnl	94	138	56	69	NA	5	4	17	15	14	7	12	ST-198 complex	198	NA	NA	3
895	OX9930679	UK [England]	1999	carrier	Neisseria meningitidis	B	B	B	13	3	1	145	3	12	5	19	160	3	174	124		1653	3	3	1
3395	OX9930681	UK [England]	1999	carrier	Neisseria meningitidis	B	B	B	14	1	1	142	13	3	6	108	9	9	6	9	ST-41/44 complex	2364	13	3	1
896	OX9930685	UK [England]	1999	carrier	Neisseria meningitidis	NG	cnl	cnl	297	15	1	276	58	16	2	6	25	17	25	22	ST-53 complex	53	NA	NA	1
56897	OX9930687	UK [England]	1999	carrier	Neisseria meningitidis	Y	L	discrepancy	13	3	57	145	3	57	5	2	35	6	68	20		963	3	3	1
4219	OX9930691	UK [England]	1999	carrier	Neisseria meningitidis	B	B	B	13	3	1	145	3	4	10	2	9	11	11	20	ST-269 complex	3064	3	3	1
897	OX9930699	UK [England]	1999	carrier	Neisseria meningitidis	NG	Y	Y	25	1	1	26	4	12	5	18	9	11	9	17	ST-23 complex	1655	4	4	2
4230	OX9930704	UK [England]	1999	carrier	Neisseria meningitidis	Y	Y	Y	16	1	4	59	4	4	10	11	17	6	10	17	ST-35 complex	3075	4	4	2
56899	OX9930707	UK [England]	1999	carrier	Neisseria meningitidis	B	B	B	45	8	77	87	20	7	5	1	13	36	53	15	ST-213 complex	213	20	4	3
56900	OX9930710	UK [England]	1999	carrier	Neisseria meningitidis	Y	Y	Y	23	1	1	4	4	8	7	6	17	9	18	8	ST-167 complex	279	4	4	2
56902	OX9930712	UK [England]	1999	carrier	Neisseria meningitidis	NG	B	B	45	8	77	87	20	7	5	1	13	36	53	15	ST-213 complex	213	20	4	3
56904	OX9930714	UK [England]	1999	carrier	Neisseria meningitidis	NG	E	E	13	3	57	209	3	8	25	7	17	21	26	49	ST-1157 complex	1157	3	3	1
898	OX9930715	UK [England]	1999	carrier	Neisseria meningitidis	NG	B	B	19	1	1	65	4	12	6	9	162	9	6	9	ST-41/44 complex	1656	4	4	2
28857	OX9930716	UK [England]	1999	carrier	Neisseria meningitidis	NG	B	B	19	1	1	65	4	9	6	15	9	9	6	18	ST-41/44 complex	1228	4	4	2
3396	OX9930719	UK [England]	1999	carrier	Neisseria meningitidis		B	B	15	4	2	193	1	4	10	15	9	8	11	9	ST-269 complex	269	1	1	1
6004	OX9930720	UK [England]	1999	carrier	Neisseria meningitidis	NG	Z	Z	14	1	63	142	13	6	3	2	24	10	306	96		4147	13	3	1
4291	OX9930723	UK [England]	1999	carrier	Neisseria meningitidis	W	W	W	16	1	1	60	4	11	5	18	8	11	24	21	ST-22 complex	22	4	4	2
56909	OX9930727	UK [England]	1999	carrier	Neisseria meningitidis	NG	E	E	13	3	1	209	3	8	25	7	17	21	26	49	ST-1157 complex	1157	3	3	1
56911	OX9930728	UK [England]	1999	carrier	Neisseria meningitidis	E	E	E	13	3	1	145	3	17	5	19	17	3	26	2	ST-60 complex	60	3	3	1
56912	OX9930730	UK [England]	1999	carrier	Neisseria meningitidis	NG	cnl	cnl	102	8	1	2	20	16	2	6	25	17	25	22	ST-53 complex	53	20	4	2
56914	OX9930735	UK [England]	1999	carrier	Neisseria meningitidis	B	B	B	15	4	2	193	1	4	10	15	9	8	11	9	ST-269 complex	269	1	1	1
56915	OX9930738	UK [England]	1999	carrier	Neisseria meningitidis	NG	C	C	19	1	1	65	4	8	26	9	24	26	20	18	ST-212 complex	212	4	4	2
56917	OX9930740	UK [England]	1999	carrier	Neisseria meningitidis	NG	cnl	cnl	4	1	1	206	4	5	4	17	15	30	7	12	ST-198 complex	823	4	3	1
899	OX9930754	UK [England]	1999	carrier	Neisseria meningitidis	Y	Y	Y	104	1	NA	10	4	10	5	18	9	11	9	17	ST-23 complex	23	4	4	2
56918	OX9930757	UK [England]	1999	carrier	Neisseria meningitidis	B	B	B	13	3	1	145	3	12	5	19	160	3	174	124		1653	3	3	1
56919	OX9930760	UK [England]	1999	carrier	Neisseria meningitidis	Y	Y	Y	104	1	NA	10	4	10	5	18	9	11	9	17	ST-23 complex	23	4	4	2
28858	OX9930765	UK [England]	1999	carrier	Neisseria meningitidis	B	B	B	19	1	1	65	4	9	5	9	9	9	6	2	ST-41/44 complex	1423	4	4	2
3397	OX9930766	UK [England]	1999	carrier	Neisseria meningitidis	NG	C	C	16	1	4	59	4	10	10	11	18	170	10	129	ST-35 complex	1679	4	4	2
900	OX9930767	UK [England]	1999	carrier	Neisseria meningitidis	E	E	E	13	3	1	145	3	17	5	19	5	3	26	2	ST-60 complex	1650	3	3	1
4301	OX9930771	UK [England]	1999	carrier	Neisseria meningitidis	NG	E	E	13	3	57	145	3	2	16	12	11	3	60	7	ST-254 complex	254	3	3	1
56921	OX9930773	UK [England]	1999	carrier	Neisseria meningitidis	NG	E	E	13	3	57	209	3	8	25	7	17	21	26	49	ST-1157 complex	1157	3	3	1
3398	OX9930776	UK [England]	1999	carrier	Neisseria meningitidis	NG	B	B	106	1	2	146	4	4	10	2	161	8	11	20	ST-269 complex	2369	4	4	2
56924	OX9930780	UK [England]	1999	carrier	Neisseria meningitidis	NG	Y	Y	1107	1	1	NA	4	2	7	6	17	1	18	8	ST-167 complex	1627	4	4	2
56882	OX9930788	UK [England]	1999	carrier	Neisseria meningitidis	NG	W	W	16	1	1	60	4	11	5	18	8	11	24	21	ST-22 complex	22	4	4	2
56926	OX9930791	UK [England]	1999	carrier	Neisseria meningitidis	E	E	E	23	1	1	4	4	17	5	19	17	3	26	2	ST-60 complex	60	4	4	2
56928	OX9930799	UK [England]	1999	carrier	Neisseria meningitidis	NG	Y	Y	23	1	1	4	4	2	7	6	17	9	6	8	ST-167 complex	1624	4	4	2
56930	OX9930801	UK [England]	1999	carrier	Neisseria meningitidis	NG	cnl	cnl	94	138	56	69	NA	5	4	17	15	14	7	12	ST-198 complex	198	NA	NA	3
56931	OX9930802	UK [England]	1999	carrier	Neisseria meningitidis	Y	Y	Y	25	1	1	26	4	12	5	18	9	11	9	17	ST-23 complex	1655	4	4	2
901	OX9930804	UK [England]	1999	carrier	Neisseria meningitidis	NG	W	W	22	1	1	1	4	2	5	9	17	11	24	21	ST-22 complex	1659	4	4	2
56933	OX9930809	UK [England]	1999	carrier	Neisseria meningitidis	NG	cnl	cnl	297	15	1	276	58	16	2	6	25	17	25	22	ST-53 complex	53	NA	NA	1
56935	OX9930810	UK [England]	1999	carrier	Neisseria meningitidis	NG	Y	Y	23	1	1	4	4	2	16	6	17	9	18	8	ST-167 complex	168	4	4	2
56791	OX9930811	UK [England]	1999	carrier	Neisseria meningitidis	NG	Y	Y	25	1	1	26	4	10	5	18	9	11	9	17	ST-23 complex	23	4	4	2
56793	OX9930812	UK [England]	1999	carrier	Neisseria meningitidis	NG	E	E	13	3	57	209	3	8	25	7	17	21	26	49	ST-1157 complex	1157	3	3	1
56795	OX9930813	UK [England]	1999	carrier	Neisseria meningitidis	NG	E	E	13	3	1	145	3	863	5	6	17	3	26	2	ST-60 complex	13596	3	3	1
902	OX9930817	UK [England]	1999	carrier	Neisseria meningitidis	W	W	W	16	1	1	60	4	11	5	18	8	154	24	21	ST-22 complex	13246	4	4	2
56797	OX9930818	UK [England]	1999	carrier	Neisseria meningitidis	NG	B	B	13	3	4	145	3	8	92	12	9	3	22	6		1422	3	3	1
56799	OX9930820	UK [England]	1999	carrier	Neisseria meningitidis	NG	E	E	13	3	1	145	3	17	5	19	17	3	26	2	ST-60 complex	60	3	3	1
56801	OX9930822	UK [England]	1999	carrier	Neisseria meningitidis	NG	Y	Y	19	1	1	93	13	2	16	6	17	9	18	8	ST-167 complex	168	13	4	2
56803	OX9930825	UK [England]	1999	carrier	Neisseria meningitidis	E	E	E	13	3	1	145	3	17	5	19	17	3	26	2	ST-60 complex	60	3	3	1
28859	OX9930826	UK [England]	1999	carrier	Neisseria meningitidis	B	B	B	19	1	1	65	4	12	6	9	9	9	6	9	ST-41/44 complex	43	4	4	2
56806	OX9930827	UK [England]	1999	carrier	Neisseria meningitidis	NG	E	E	13	3	57	145	3	2	16	12	11	3	60	7	ST-254 complex	254	3	3	1
56808	OX9930828	UK [England]	1999	carrier	Neisseria meningitidis	C	C	C	201	1	1	23	4	2	3	4	3	8	4	6	ST-11 complex	11	4	4	2
56810	OX9930853	UK [England]	1999	carrier	Neisseria meningitidis	NG	B	B	19	1	1	83	4	4	10	2	5	38	11	9	ST-269 complex	275	4	4	2
4382	OX9930854	UK [England]	1999	carrier	Neisseria meningitidis		E	E	13	3	1	145	3	17	5	19	164	3	26	2	ST-60 complex	3232	3	3	1
28966	OX9930855	UK [England]	1999	carrier	Neisseria meningitidis		B	B	24	1	1	25	4	9	6	9	9	9	6	2	ST-41/44 complex	180	4	4	2
56812	OX9930857	UK [England]	1999	carrier	Neisseria meningitidis	C	C	C	0	5	1	917	11	2	3	4	3	8	4	6	ST-11 complex	11	11	2	NA
56814	OX9930863	UK [England]	1999	carrier	Neisseria meningitidis	C	C	C	22	1	1	1	4	2	3	4	3	8	4	6	ST-11 complex	11	4	4	2
28860	OX9930864	UK [England]	1999	carrier	Neisseria meningitidis	B	B	B	24	1	1	25	4	9	6	9	9	9	6	2	ST-41/44 complex	180	4	4	2
56816	OX9930868	UK [England]	1999	carrier	Neisseria meningitidis	B	B	B	45	8	77	87	20	7	5	1	13	36	53	15	ST-213 complex	213	20	4	3
903	OX9930872	UK [England]	1999	carrier	Neisseria meningitidis	W	W	W	16	1	1	60	4	11	5	18	8	11	24	21	ST-22 complex	22	4	4	2
4331	OX9930874	UK [England]	1999	carrier	Neisseria meningitidis	NG	cnl	cnl	102	8	1	2	20	16	2	6	25	17	25	22	ST-53 complex	53	20	4	2
56821	OX9930875	UK [England]	1999	carrier	Neisseria meningitidis	B	B	B	19	1	1	83	4	4	10	2	5	38	11	9	ST-269 complex	275	4	4	2
56885	OX9930876	UK [England]	1999	carrier	Neisseria meningitidis	NG	W	W	16	1	1	60	4	11	5	18	8	11	24	21	ST-22 complex	22	4	4	2
56822	OX9930883	UK [England]	1999	carrier	Neisseria meningitidis	NG	cnl	cnl	102	8	1	2	20	16	2	6	25	17	25	22	ST-53 complex	53	20	4	2
56823	OX9930885	UK [England]	1999	carrier	Neisseria meningitidis	NG	Y	Y	25	1	1	26	4	10	5	18	9	11	9	17	ST-23 complex	23	4	4	2
35897	OX9930886	UK [England]	1999	carrier	Neisseria meningitidis		W	W	16	1	1	60	4	11	5	18	8	153	4	21	ST-22 complex	1426	4	4	2
56824	OX9930892	UK [England]	1999	carrier	Neisseria meningitidis	NG	B	B	1	111	3	178	NA	8	10	5	4	6	3	8	ST-32 complex	33	NA	NA	1
56825	OX9930898	UK [England]	1999	carrier	Neisseria meningitidis	Y	Y	Y	25	1	1	26	4	10	5	18	9	11	9	17	ST-23 complex	23	4	4	2
905	OX9930902	UK [England]	1999	carrier	Neisseria meningitidis	NG	cnl	cnl	102	8	1	2	20	16	2	6	25	17	25	22	ST-53 complex	53	20	4	2
28861	OX9930906	UK [England]	1999	carrier	Neisseria meningitidis	B	B	B	19	1	1	65	4	12	6	9	9	9	6	9	ST-41/44 complex	43	4	4	2
906	OX9930917	UK [England]	1999	carrier	Neisseria meningitidis	Y	Y	Y	25	1	1	26	4	10	5	18	9	11	26	17	ST-23 complex	1664	4	4	2
56829	OX9930918	UK [England]	1999	carrier	Neisseria meningitidis	NG	E	E	13	3	1	145	3	17	5	19	17	3	26	2	ST-60 complex	60	3	3	1
56831	OX9930920	UK [England]	1999	carrier	Neisseria meningitidis	E	E	E	13	3	1	145	3	17	5	19	17	3	26	2	ST-60 complex	60	3	3	1
3399	OX9930923	UK [England]	1999	carrier	Neisseria meningitidis	W	W	W	16	1	1	60	4	11	5	18	8	11	157	21	ST-22 complex	1667	4	4	2
56834	OX9930928	UK [England]	1999	carrier	Neisseria meningitidis	NG	cnl	cnl	1105	1	1	1004	4	5	4	17	15	30	7	12	ST-198 complex	823	4	3	1
4375	OX9930930	UK [England]	1999	carrier	Neisseria meningitidis	NG	cnl	cnl	21	1	1	7	4	16	2	7	92	5	25	112	ST-1117 complex	13238	4	4	2
3400	OX9930931	UK [England]	1999	carrier	Neisseria meningitidis	NG	cnl	cnl	94	138	56	69	NA	5	4	17	15	14	7	12	ST-198 complex	198	NA	NA	3
56835	OX9930932	UK [England]	1999	carrier	Neisseria meningitidis	NG	cnl	cnl	94	157	56	69	NA	5	4	17	15	14	7	12	ST-198 complex	198	NA	NA	3
4257	OX9930938	UK [England]	1999	carrier	Neisseria meningitidis	B	C	discrepancy	19	1	1	65	4	8	26	9	24	26	20	18	ST-212 complex	212	4	4	2
56838	OX9930943	UK [England]	1999	carrier	Neisseria meningitidis	NG	B	B	15	4	2	193	1	4	10	15	9	8	11	9	ST-269 complex	269	1	1	1
28862	OX9930945	UK [England]	1999	carrier	Neisseria meningitidis	B	B	B	14	1	1	142	13	3	6	9	5	9	6	9	ST-41/44 complex	41	13	3	1
28863	OX9930951	UK [England]	1999	carrier	Neisseria meningitidis	B	B	B	336	1	1	9	4	9	6	9	9	9	6	2	ST-41/44 complex	180	4	4	2
56839	OX9930952	UK [England]	1999	carrier	Neisseria meningitidis	B	B	B	16	1	4	59	4	4	10	11	17	6	10	12	ST-35 complex	457	4	4	2
907	OX9930955	UK [England]	1999	carrier	Neisseria meningitidis	NG	Y	Y	25	1	1	26	4	6	5	15	9	26	68	17		3107	4	4	2
56841	OX9930957	UK [England]	1999	carrier	Neisseria meningitidis	NG	NG	NG	1	6	3	100	7	8	10	5	4	5	3	8	ST-32 complex	34	7	1	1
56842	OX9930961	UK [England]	1999	carrier	Neisseria meningitidis	B	B	B	45	8	77	87	20	7	5	1	13	36	53	15	ST-213 complex	213	20	4	3
28864	OX9930963	UK [England]	1999	carrier	Neisseria meningitidis	B	cnl	discrepancy	285	1	1	5	4	12	91	6	181	177	186	8		2121	4	4	2
56844	OX9930966	UK [England]	1999	carrier	Neisseria meningitidis	Y	Y	Y	25	1	1	26	4	10	5	18	9	11	9	17	ST-23 complex	23	4	4	2
28865	OX9930970	UK [England]	1999	carrier	Neisseria meningitidis	B	B	B	19	1	1	65	4	9	5	9	9	9	6	2	ST-41/44 complex	1423	4	4	2
56846	OX9930971	UK [England]	1999	carrier	Neisseria meningitidis	E	E	E	13	5	1	220	11	2	5	19	9	3	26	2	ST-60 complex	1411	11	2	1
56848	OX9930975	UK [England]	1999	carrier	Neisseria meningitidis	B	B	B	45	8	77	87	20	7	5	1	13	36	53	15	ST-213 complex	213	20	4	3

14591	OX9930980	UK [England]	1999	carrier	Neisseria meningitidis	NG	E	E	125	1	57	268	13	7	16	55	10	3	17	46	ST-178 complex	2393	13	3	1
56888	OX9930982	UK [England]	1999	carrier	Neisseria meningitidis	Y	Y	Y	16	1	1	60	4	11	5	18	17	11	24	21	ST-22 complex	114	4	4	2
56852	OX9930986	UK [England]	1999	carrier	Neisseria meningitidis	Y	Y	Y	23	1	1	4	4	2	16	6	17	9	18	8	ST-167 complex	168	4	4	2
56853	OX9930989	UK [England]	1999	carrier	Neisseria meningitidis	NG	B	B	15	4	2	193	1	4	10	15	9	8	11	9	ST-269 complex	269	1	1	1
28866	OX9930996	UK [England]	1999	carrier	Neisseria meningitidis	B	B	B	19	1	1	65	4	9	20	9	9	9	6	2	ST-41/44 complex	1097	4	4	2
56855	OX9931000	UK [England]	1999	carrier	Neisseria meningitidis	NG	cnl	cnl	297	15	1	276	58	16	2	6	25	17	25	22	ST-53 complex	53	NA	NA	1
56856	OX9931006	UK [England]	1999	carrier	Neisseria meningitidis	NG	cnl	cnl	102	8	1	2	20	16	2	6	25	17	25	22	ST-53 complex	53	20	4	2
56858	OX9931008	UK [England]	1999	carrier	Neisseria meningitidis	NG	cnl	cnl	94	138	56	69	NA	5	4	17	15	14	7	12	ST-198 complex	198	NA	NA	3
57042	OX9931013	UK [England]	1999	carrier	Neisseria meningitidis	B	B	B	45	8	1	87	20	7	5	1	13	36	53	15	ST-213 complex	213	20	4	3
4268	OX9931017	UK [England]	1999	carrier	Neisseria meningitidis	B	B	B	45	8	77	87	20	7	5	1	13	36	53	15	ST-213 complex	213	20	4	3
28867	OX9931018	UK [England]	1999	carrier	Neisseria meningitidis	B	B	B	14	1	1	142	13	3	6	9	5	9	6	156	ST-41/44 complex	1823	13	3	1
4200	OX9931019	UK [England]	1999	carrier	Neisseria meningitidis		B	B	14	1	1	142	13	3	6	9	5	9	6	156	ST-41/44 complex	1823	13	3	1
56861	OX9931024	UK [England]	1999	carrier	Neisseria meningitidis	NG	E	E	13	3	57	209	3	8	25	7	17	21	26	49	ST-1157 complex	1157	3	3	1
3401	OX9931026	UK [England]	1999	carrier	Neisseria meningitidis	NG	E	E	141	1	4	116	4	8	6	19	17	11	24	54		2407	4	4	3
56865	OX9931033	UK [England]	1999	carrier	Neisseria meningitidis	NG	B	B	19	1	1	83	4	4	10	2	5	38	11	9	ST-269 complex	275	4	4	2
28868	OX9931037	UK [England]	1999	carrier	Neisseria meningitidis	NG	B	B	19	1	1	65	4	9	6	9	14	9	6	2	ST-41/44 complex	47	4	4	2
28869	OX9931039	UK [England]	1999	carrier	Neisseria meningitidis	B	B	B	337	2	1	334	2	3	6	9	5	9	6	9	ST-41/44 complex	41	2	3	1
56868	OX9931040	UK [England]	1999	carrier	Neisseria meningitidis	NG	W	W	151	1	1	130	4	2	3	4	3	8	4	6	ST-11 complex	11	4	4	2
908	OX9931045	UK [England]	1999	carrier	Neisseria meningitidis	B	B	B	34	41	1	3	51	8	3	15	32	3	11	9	ST-364 complex	1666	NA	NA	2
28870	OX9931049	UK [England]	1999	carrier	Neisseria meningitidis	B	B	B	19	1	1	65	4	3	6	9	60	9	6	9	ST-41/44 complex	170	4	4	2
56871	OX9931050	UK [England]	1999	carrier	Neisseria meningitidis	NG	cnl	cnl	94	138	56	69	NA	5	4	17	15	14	7	12	ST-198 complex	198	NA	NA	3
56874	OX9931051	UK [England]	1999	carrier	Neisseria meningitidis	B	B	B	15	4	2	193	1	4	10	15	9	8	11	9	ST-269 complex	269	1	1	1
56876	OX9931052	UK [England]	1999	carrier	Neisseria meningitidis	E	E	E	13	3	1	145	3	17	5	47	17	3	26	2	ST-60 complex	1430	3	3	1
56878	OX9931061	UK [England]	1999	carrier	Neisseria meningitidis	NG	E	E	13	3	1	209	3	8	25	7	17	21	26	49	ST-1157 complex	1157	3	3	1
28871	OX9931066	UK [England]	1999	carrier	Neisseria meningitidis	NG	B	B	338	7	1	NA	6	3	6	9	5	9	6	9	ST-41/44 complex	41	6	2	1
909	OX9931067	UK [England]	1999	carrier	Neisseria meningitidis	W	W	W	16	1	1	60	4	11	5	18	8	11	157	21	ST-22 complex	1667	4	4	2
57008	OX9931077	UK [England]	1999	carrier	Neisseria meningitidis	NG	cnl	cnl	297	15	1	276	58	16	2	6	25	17	25	22	ST-53 complex	53	NA	NA	1
57009	OX9931100	UK [England]	1999	carrier	Neisseria meningitidis	NG	E	E	13	3	1	209	3	8	25	7	17	21	26	49	ST-1157 complex	1157	3	3	1
57010	OX9931101	UK [England]	1999	carrier	Neisseria meningitidis	NG	cnl	cnl	102	8	1	2	20	16	2	6	25	17	25	22	ST-53 complex	53	20	4	2
28872	OX9931111	UK [England]	1999	carrier	Neisseria meningitidis	NG	B	B	19	160	1	65	200	27	6	9	5	9	6	9	ST-41/44 complex	577	NA	NA	2
57011	OX9931119	UK [England]	1999	carrier	Neisseria meningitidis	NG	cnl	cnl	102	8	1	2	20	16	2	6	25	17	25	22	ST-53 complex	53	20	4	2
57012	OX9931122	UK [England]	1999	carrier	Neisseria meningitidis	X	X	X	84	12	1	70	28	2	5	2	9	15	20	5	ST-750 complex	750	NA	NA	3
57014	OX9931141	UK [England]	1999	carrier	Neisseria meningitidis	X	X	X	84	12	1	70	28	2	5	2	9	15	20	5	ST-750 complex	750	NA	NA	3
57015	OX9931145	UK [England]	1999	carrier	Neisseria meningitidis	Y	Y	Y	25	1	1	26	4	10	5	18	9	11	9	17	ST-23 complex	23	4	4	2
28873	OX9931152	UK [England]	1999	carrier	Neisseria meningitidis	B	B	B	19	1	1	65	4	12	6	9	9	9	6	9	ST-41/44 complex	43	4	4	2
57016	OX9931154	UK [England]	1999	carrier	Neisseria meningitidis	NG	cnl	cnl	94	157	56	69	NA	5	4	17	15	14	7	12	ST-198 complex	198	NA	NA	3
57018	OX9931157	UK [England]	1999	carrier	Neisseria meningitidis	NG	B	B	19	1	1	83	4	4	10	2	5	38	11	9	ST-269 complex	275	4	4	2
910	OX9931161	UK [England]	1999	carrier	Neisseria meningitidis	NG	Z	Z	25	1	1	26	4	8	3	6	17	29	18	9	ST-103 complex	1418	4	4	2
28874	OX9931165	UK [England]	1999	carrier	Neisseria meningitidis	B	B	B	19	1	1	65	4	12	6	9	9	9	6	9	ST-41/44 complex	43	4	4	2
56890	OX9931166	UK [England]	1999	carrier	Neisseria meningitidis	W	W	W	16	1	1	60	4	11	5	18	8	11	4	21	ST-22 complex	184	4	4	2
57021	OX9931185	UK [England]	1999	carrier	Neisseria meningitidis	NG	Y	Y	23	1	1	4	4	2	7	9	17	16	18	8	ST-167 complex	1412	4	4	2
57023	OX9931188	UK [England]	1999	carrier	Neisseria meningitidis	X	X	X	84	12	1	70	28	2	5	2	9	15	20	5	ST-750 complex	750	NA	NA	3
57025	OX9931191	UK [England]	1999	carrier	Neisseria meningitidis	E	E	E	13	3	1	145	3	17	5	19	17	3	26	2	ST-60 complex	60	3	3	1
35895	OX9931193	UK [England]	1999	carrier	Neisseria meningitidis		E	E	13	3	1	209	3	8	25	7	17	21	26	2	ST-1157 complex	1421	3	3	1
57027	OX9931195	UK [England]	1999	carrier	Neisseria meningitidis	NG	E	E	13	3	1	145	3	17	5	19	17	3	26	2	ST-60 complex	60	3	3	1
36223	OX9931197	UK [England]	1999	carrier	Neisseria meningitidis	B	B	B	24	1	2	25	4	27	6	9	3	9	6	16	ST-41/44 complex	136	4	4	2
57029	OX9931198	UK [England]	1999	carrier	Neisseria meningitidis	Y	Y	Y	23	1	1	4	4	8	7	6	17	9	18	8	ST-167 complex	279	4	4	2
57031	OX9931201	UK [England]	1999	carrier	Neisseria meningitidis	NG	Y	Y	25	1	1	26	4	10	5	18	9	11	9	17	ST-23 complex	23	4	4	2
57033	OX9931203	UK [England]	1999	carrier	Neisseria meningitidis	NG	C	C	24	1	4	25	4	4	10	47	17	6	2	12	ST-35 complex	278	4	4	2
57035	OX9931204	UK [England]	1999	carrier	Neisseria meningitidis	B	B	B	1108	NA	NA	NA	194	7	5	1	13	36	53	15	ST-213 complex	213	NA	NA	3
28876	OX9931218	UK [England]	1999	carrier	Neisseria meningitidis	B	B	B	19	1	1	65	4	3	6	9	60	9	26	9	ST-41/44 complex	1415	4	4	2
57038	OX9931231	UK [England]	1999	carrier	Neisseria meningitidis	NG	C	C	0	5	1	917	11	2	3	4	3	8	4	6	ST-11 complex	11	11	2	NA
911	OX9931234	UK [England]	1999	carrier	Neisseria meningitidis	B	B	B	13	3	57	209	3	8	25	7	17	21	26	49	ST-1157 complex	1157	3	3	1
36224	OX9931238	UK [England]	1999	carrier	Neisseria meningitidis	B	B	B	24	1	2	25	4	27	6	9	3	9	6	16	ST-41/44 complex	136	4	4	2
57041	OX9931239	UK [England]	1999	carrier	Neisseria meningitidis	NG	cnl	cnl	21	1	1	7	4	16	2	15	92	77	25	112	ST-1117 complex	2896	4	4	2
57043	OX9931240	UK [England]	1999	carrier	Neisseria meningitidis	Y	Y	Y	23	1	1	4	4	8	7	6	17	9	18	8	ST-167 complex	279	4	4	2
28878	OX9931242	UK [England]	1999	carrier	Neisseria meningitidis	B	B	B	19	1	1	65	4	9	20	9	9	9	6	2	ST-41/44 complex	1097	4	4	2
57045	OX9931245	UK [England]	1999	carrier	Neisseria meningitidis	NG	C	C	16	1	4	59	4	132	3	19	17	62	21	2		1434	4	4	2
57046	OX9931248	UK [England]	1999	carrier	Neisseria meningitidis	NG	E	E	1109	3	57	NA	3	2	16	12	11	3	60	7	ST-254 complex	254	3	3	1
28879	OX9931249	UK [England]	1999	carrier	Neisseria meningitidis	B	B	B	19	1	1	65	4	9	20	9	9	9	6	2	ST-41/44 complex	1097	4	4	2
56893	OX9931256	UK [England]	1999	carrier	Neisseria meningitidis	NG	W	W	16	1	1	60	4	11	5	18	8	11	24	21	ST-22 complex	22	4	4	2
57048	OX9931260	UK [England]	1999	carrier	Neisseria meningitidis	NG	L	L	13	3	57	145	3	57	5	2	35	6	68	20		963	3	3	1
28880	OX9931268	UK [England]	1999	carrier	Neisseria meningitidis	B	B	B	19	1	1	65	4	9	20	9	9	9	6	2	ST-41/44 complex	1097	4	4	2
57050	OX9931276	UK [England]	1999	carrier	Neisseria meningitidis	E	E	E	13	3	57	209	3	8	6	7	17	21	26	49	ST-1157 complex	1419	3	3	1
57052	OX9931278	UK [England]	1999	carrier	Neisseria meningitidis	B	B	B	106	1	1	146	4	4	10	15	9	8	11	16	ST-269 complex	1416	4	4	2
57055	OX9931279	UK [England]	1999	carrier	Neisseria meningitidis	NG	L	L	13	3	57	145	3	57	5	2	35	6	68	20		963	3	3	1
57057	OX9931282	UK [England]	1999	carrier	Neisseria meningitidis	C	C	C	1110	125	1	NA	147	2	3	4	3	8	4	6	ST-11 complex	11	NA	NA	1
28881	OX9931283	UK [England]	1999	carrier	Neisseria meningitidis	B	B	B	19	1	1	65	4	9	20	9	9	9	6	2	ST-41/44 complex	1097	4	4	2
912	OX9931284	UK [England]	1999	carrier	Neisseria meningitidis	B	B	B	16	1	4	59	4	17	5	7	17	157	26	2	ST-60 complex	1670	4	4	2
57060	OX9931285	UK [England]	1999	carrier	Neisseria meningitidis	E	E	E	13	3	1	209	3	8	25	7	17	21	26	49	ST-1157 complex	1157	3	3	1
57061	OX9931289	UK [England]	1999	carrier	Neisseria meningitidis	E	E	E	19	1	1	65	4	8	26	9	24	26	20	18	ST-212 complex	212	4	4	2
57063	OX9931294	UK [England]	1999	carrier	Neisseria meningitidis	NG	cnl	cnl	102	8	1	2	20	16	2	6	25	17	25	22	ST-53 complex	53	20	4	2
57065	OX9931295	UK [England]	1999	carrier	Neisseria meningitidis	E	L	discrepancy	13	3	57	145	3	57	5	2	35	6	68	20		963	3	3	1
57066	OX9931309	UK [England]	1999	carrier	Neisseria meningitidis	Y	Y	Y	25	1	1	26	4	10	5	18	9	11	9	17	ST-23 complex	23	4	4	2
56895	OX9931315	UK [England]	1999	carrier	Neisseria meningitidis	W	W	W	16	1	1	60	4	11	5	18	8	11	4	21	ST-22 complex	184	4	4	2
3402	OX9931333	UK [England]	1999	carrier	Neisseria meningitidis	E	C	discrepancy	499	1	1	134	15	144	10	5	4	6	3	8	ST-32 complex	2472	15	4	3
57068	OX9931344	UK [England]	1999	carrier	Neisseria meningitidis	NG	cnl	cnl	102	8	1	2	20	16	2	6	25	17	25	22	ST-53 complex	53	20	4	2
57069	OX9931346	UK [England]	1999	carrier	Neisseria meningitidis	B	B	B	45	8	77	87	20	7	5	1	13	36	53	15	ST-213 complex	213	20	4	3
57071	OX9931349	UK [England]	1999	carrier	Neisseria meningitidis	NG	B	B	16	1	4	59	4	4	10	11	18	3	10	12	ST-35 complex	1417	4	4	2
56937	OX9931351	UK [England]	1999	carrier	Neisseria meningitidis	NG	cnl	cnl	102	8	1	2	20	16	2	6	25	17	25	22	ST-53 complex	53	20	4	2
28882	OX9931352	UK [England]	1999	carrier	Neisseria meningitidis	NG	B	B	19	1	1	65	4	9	95	9	9	9	6	9	ST-41/44 complex	1425	4	4	2
56939	OX9931353	UK [England]	1999	carrier	Neisseria meningitidis	NG	B	B	45	8	77	87	20	7	5	1	13	36	53	15	ST-213 complex	213	20	4	3
56941	OX9931358	UK [England]	1999	carrier	Neisseria meningitidis	NG	cnl	cnl	102	8	1	2	20	16	2	6	25	17	25	22	ST-53 complex	53	20	4	2
56943	OX9931359	UK [England]	1999	carrier	Neisseria meningitidis	NG	cnl	cnl	102	8	1	2	20	16	2	6	25	17	25	22	ST-53 complex	53	20	4	2
56945	OX9931363	UK [England]	1999	carrier	Neisseria meningitidis	NG	cnl	cnl	94	184	56	89	NA	5	4	38	15	22	40	13	ST-1136 complex	1136	NA	NA	3
56997	OX9931366	UK [England]	1999	carrier	Neisseria meningitidis	NG	Y	Y	25	1	1	26	4	10	5	18	9	11	9	17	ST-23 complex	23	4	4	2
56947	OX9931387	UK [England]	1999	carrier	Neisseria meningitidis	NG	B	B	19	1	1	83	4	4	10	2	5	38	11	9	ST-269 complex	275	4	4	2
4302	OX9931415	UK [England]	1999	carrier	Neisseria meningitidis	NG	E	E	13	3	57	209	3	8	25	7	17	21	26	49	ST-1157 complex	1157	3	3	1
56950	OX9931417	UK [England]	1999	carrier	Neisseria meningitidis	B	B	B	15	4	2	193	1	4	10	15	9	8	11	17	ST-269 complex	283	1	1	1
56951	OX9931421	UK [England]	1999	carrier	Neisseria meningitidis	Y	Y	Y	25	1	1	26	4	10	5	18	9	11	9	17	ST-23 complex	23	4	4	2
35886	OX9931422	UK [England]	1999	carrier	Neisseria meningitidis		E	E	13	3	57	145	3	2	16	12	11	3	60	7	ST-254 complex	254	3	3	1
56952	OX9931427	UK [England]	1999	carrier	Neisseria meningitidis	NG	cnl	cnl	102	8	1	2	20	16	2	6	25	17	25	22	ST-53 complex	53	20	4	2
913	OX9931428	UK [England]	1999	carrier	Neisseria meningitidis	B	B	B	19	1	1	83	4	4	10	2	5	38	11	9	ST-269 complex	275	4	4	2
56955	OX9931429	UK [England]	1999	carrier	Neisseria meningitidis	C	C	C	22	1	1	1	4	2	3	4	3	8	4	6	ST-11 complex	11	4	4	2
4325	OX9931431	UK [England]	1999	carrier	Neisseria meningitidis	Y	Y	Y	25	1	1	26	4	10	5	18	9	11	9	17	ST-23 complex	23	4	4	2
56958	OX9931433	UK [England]	1999	carrier	Neisseria meningitidis	B	B	B	45	8	77	87	20	7	5	1	13	36	53	15	ST-213 complex	213	20	4	3
4201	OX9931436	UK [England]	1999	carrier	Neisseria meningitidis	B	B	B	4	2	1	84	2	3	6	9	17	9	6	9	ST-41/44 complex	482	2	3	1
56960	OX9931441	UK [England]	1999	carrier	Neisseria meningitidis	NG	B	B	15	4	2	193	1	4	10	15	9	8	11	9	ST-269 complex	269	1	1	1
56962	OX9931449	UK [England]	1999	carrier	Neisseria meningitidis	E	E	E	125	1	57	268	13	7	16	55	10	3	56	46	ST-178 complex	178	13	3	1
56963	OX9931468	UK [England]	1999	carrier	Neisseria meningitidis	NG	E	E	13	3	57	209	3	8	25	7	17	21	26	49	ST-1157 complex	1157	3	3	1
56965	OX9931473	UK [England]	1999	carrier	Neisseria meningitidis	NG	Y	Y	25	1	1	26	4	10	5	18	9	11	9	17	ST-23 complex	23	4	4	2
56966	OX9931481	UK [England]	1999	carrier	Neisseria meningitidis	NG	B	B	15	4	2	193	1	4	10	15	9	8	11	9	ST-269 complex	269	1	1	1
4192	OX9931489	UK [England]	1999	carrier	Neisseria meningitidis	NG	C	C	16	1	4	59	4	2	3	7	6	8	5	2	ST-8 complex	66	4	4	2
56970	OX9931491	UK [England]	1999	carrier	Neisseria meningitidis	NG	cnl	cnl	4	1	1	206	4	5	4	17	15	30	7	12	ST-198 complex	823	4	3	1
56896	OX9931495	UK [England]	1999	carrier	Neisseria meningitidis	W	W	W	16	1	1	60	4	11	5	18	8	11	4	21	ST-22 complex	184	4	4	2
56971	OX9931497	UK [England]	1999	carrier	Neisseria meningitidis	Y	Y	Y	25	1	1	26	4	10	5	18	9	11	9	17	ST-23 complex	23	4	4	2
56972	OX9931498	UK [England]	1999	carrier	Neisseria meningitidis	B	B	B	409	9	77	1006	16	7	5	1	13	36	53	15	ST-213 complex	213	16	2	3
56973	OX9931502	UK [England]	1999	carrier	Neisseria meningitidis	B	B	B	13	3	57	145	3	4	10	2	5	38	11	9	ST-269 complex	275	3	3	1
914	OX9931503	UK [England]	1999	carrier	Neisseria meningitidis	B	B	B	24	1	2	25	4	12	2	15	8	26	163	16		1672	4	4	2
56898	OX9931508	UK [England]	1999	carrier	Neisseria meningitidis	NG	W	W	16	NA	1	60	NA	11	5	18	8	11	4	21	ST-22 complex	184	NA	NA	2
56975	OX9931509	UK [England]	1999	carrier	Neisseria meningitidis	Y	Y	Y	25	1	1	26	4	10	5	18	9	11	9	17	ST-23 complex	23	4	4	2
56977	OX9931513	UK [England]	1999	carrier	Neisseria meningitidis	NG	B	B	15	4	2	193	1	4	10	15	9	8	11	9	ST-269 complex	269	1	1	1
56980	OX9931514	UK [England]	1999	carrier	Neisseria meningitidis	NG	Y	Y	27	1	1	NA	4	2	7	6	17	9	18	8	ST-167 complex	766	4	4	2
5587	OX9931515	UK [England]	1999	carrier	Neisseria meningitidis		Y	Y	23	1	1	4	4	9	7	6	24	11	18	8	ST-167 complex	1760	4	4	2
56982	OX9931516	UK [England]	1999	carrier	Neisseria meningitidis	NG	Y	Y	25	1	1	26	4	10	5	18	9	11	9	17	ST-23 complex	23	4	4	2
56984	OX9931523	UK [England]	1999	carrier	Neisseria meningitidis	Y	Y	Y	25	1	1	26	4	2	5	18	17	11	24	21	ST-22 complex	2180	4	4	2
56985	OX9931526	UK [England]	1999	carrier	Neisseria meningitidis	B	B	B	15	4	2	193	1	4	10	15	9	8	11	9	ST-269 complex	269	1	1	1
56987	OX9931529	UK [England]	1999	carrier	Neisseria meningitidis	NG	cnl	cnl	102	8	1	2	20	16	2	6	25	17	25	22	ST-53 complex	53	20	4	2
57017	OX9931530	UK [England]	1999	carrier	Neisseria meningitidis	W	W	W	16	1	1	60	4	11	5	18	8	11	4	21	ST-22 complex	184	4	4	2
56989	OX9931532	UK [England]	1999	carrier	Neisseria meningitidis	NG	E	E	13	3	57	145	3	23	3	4	25	3	22	7		1431	3	3	1
56991	OX9931534	UK [England]	1999	carrier	Neisseria meningitidis	B	B	B	210	1	1	267	4	4	10	15	9	8	11	9	ST-269 complex	269	4	3	1
4423	OX9931535	UK [England]	1999	carrier	Neisseria meningitidis	B	B	B	45	8	77	87	20	7	5	1	13	36	53	15	ST-213 complex	213	20	4	3
4390	OX9931541	UK [England]	1999	carrier	Neisseria meningitidis	NG	B	B	23	250	1	431	196	137	5	15	17	8	21	2	ST-865 complex	1675	NA	NA	2
56995	OX9931543	UK [England]	1999	carrier	Neisseria meningitidis	NG	E	E	13	3	57	145	3	2	16	12	11	123	22	6	ST-254 complex	1827	3	3	1
14592	OX9931545	UK [England]	1999	carrier	Neisseria meningitidis	NG	Y	Y	23	1	1	4	4	2	7	6	17	16	18	8	ST-167 complex	167	4	4	2
28883	OX9931550	UK [England]	1999	carrier	Neisseria meningitidis	B	B	B	19	1	1	65	4	9	20	9	9	9	6	2	ST-41/44 complex	1097	4	4	2
56996	OX9931551	UK [England]	1999	carrier	Neisseria meningitidis	E	E	E	13	3	1	145	3	17	5	19	17	3	26	2	ST-60 complex	60	3	3	1
28884	OX9931553	UK [England]	1999	carrier	Neisseria meningitidis	E	B	discrepancy	19	1	1	65	4	9	6	9	9	9	6	9	ST-41/44 complex	44	4	4	2
14593	OX9931556	UK [England]	1999	carrier	Neisseria meningitidis		E	E	13	3	57	209	3	8	25	7	17	21	26	49	ST-1157 complex	1157	3	3	1
14594	OX9931559	UK [England]	1999	carrier	Neisseria meningitidis	NG	cnl	cnl	94	138	56	69	NA	5	4	17	15	14	7	12	ST-198 complex	198	NA	NA	3
4193	OX9931563	UK [England]	1999	carrier	Neisseria meningitidis	C	C	C	24	1	63	25	4	27	6	9	3	9	6	16	ST-41/44 complex	136	4	4	2
4233	OX9931565	UK [England]	1999	carrier	Neisseria meningitidis	B	B	B	16	1	4	59	4	4	10	11	18	6	10	12	ST-35 complex	35	4	4	2
4226	OX9931567	UK [England]	1999	carrier	Neisseria meningitidis	B	B	B	1103	1	1	NA	4	3	10	48	9	159	10	12		4214	4	4	2
3403	OX9931568	UK [England]	1999	carrier	Neisseria meningitidis	NG	NG	NG	23	1	1	4	4	9	7	6	17	11	18	17	ST-167 complex	1636	4	4	2
4312	OX9931575	UK [England]	1999	carrier	Neisseria meningitidis	Y	Y	Y	104	1	57	10	4	10	5	18	9	11	9	17	ST-23 complex	23	4	4	2
4330	OX9931582	UK [England]	1999	carrier	Neisseria meningitidis	B	B	B	16	1	4	59	4	4	10	11	18	6	10	12	ST-35 complex	35	4	4	2
57001	OX9931585	UK [England]	1999	carrier	Neisseria meningitidis	NG	cnl	cnl	297	15	1	276	58	16	2	6	25	17	25	22	ST-53 complex	53	NA	NA	1
3404	OX9931589	UK [England]	1999	carrier	Neisseria meningitidis	X	X	X	84	12	1	70	28	2	5	2	9	15	20	5	ST-750 complex	750	NA	NA	3

14595	OX9931590	UK [England]	1999	carrier	Neisseria meningitidis	NG	cnl	cnl	102	8	1	2	20	16	2	6	25	17	25	22	ST-53 complex	53	20	4	2
57019	OX9931591	UK [England]	1999	carrier	Neisseria meningitidis	NG	NG	NG	16	1	1	477	NA	11	5	18	8	11	24	21	ST-22 complex	22	NA	NA	2
3405	OX9931593	UK [England]	1999	carrier	Neisseria meningitidis	NG	cnl	cnl	102	8	1	2	20	16	2	6	25	17	25	22	ST-53 complex	53	20	4	2
3406	OX9931596	UK [England]	1999	carrier	Neisseria meningitidis	E	E	E	13	3	1	145	3	17	5	19	17	3	6	2	ST-60 complex	1677	3	3	1
57003	OX9931597	UK [England]	1999	carrier	Neisseria meningitidis	NG	B	B	15	4	2	193	1	4	10	15	9	8	11	9	ST-269 complex	269	1	1	1
4224	OX9931600	UK [England]	1999	carrier	Neisseria meningitidis	NG	B	B	15	4	2	193	1	4	10	4	9	8	11	9	ST-269 complex	2746	1	1	1
57006	OX9931605	UK [England]	1999	carrier	Neisseria meningitidis	NG	cnl	cnl	21	1	1	7	4	16	2	159	92	77	25	112	ST-1117 complex	1117	4	4	2
57007	OX9931608	UK [England]	1999	carrier	Neisseria meningitidis	NG	E	E	13	3	57	209	3	8	25	7	17	21	26	49	ST-1157 complex	1157	3	3	1
3407	OX9931612	UK [England]	1999	carrier	Neisseria meningitidis	B	B	B	16	1	4	59	4	4	3	11	18	6	10	12	ST-35 complex	160	4	4	2
4214	OX9931620	UK [England]	1999	carrier	Neisseria meningitidis	NG	NG	NG	16	1	1	477	195	11	5	18	8	11	24	21	ST-22 complex	22	NA	NA	2
3408	OX9931621	UK [England]	1999	carrier	Neisseria meningitidis	NG	cnl	cnl	31	9	1	58	16	16	2	6	25	17	25	22	ST-53 complex	53	16	2	3
4377	OX9931625	UK [England]	1999	carrier	Neisseria meningitidis	NG	W	W	16	NA	1	60	197	11	5	18	8	11	4	21	ST-22 complex	184	NA	NA	2
4313	OX9931626	UK [England]	1999	carrier	Neisseria meningitidis	W	W	W	16	1	1	60	4	11	5	18	8	11	4	21	ST-22 complex	184	4	4	2
57013	OX9931632	UK [England]	1999	carrier	Neisseria meningitidis	Y	Y	Y	25	1	1	26	4	10	5	18	14	11	9	17	ST-23 complex	1625	4	4	2
4340	OX9931636	UK [England]	1999	carrier	Neisseria meningitidis	NG	Y	Y	25	1	1	26	4	10	5	18	9	11	9	17	ST-23 complex	23	4	4	2
3410	OX9931638	UK [England]	1999	carrier	Neisseria meningitidis	Y	Y	Y	16	1	1	60	4	11	5	18	8	11	24	21	ST-22 complex	22	4	4	2
4311	OX9931646	UK [England]	1999	carrier	Neisseria meningitidis	B	B	B	19	1	2	65	4	9	26	6	37	9	20	18		13205	4	4	2
57020	OX9931647	UK [England]	1999	carrier	Neisseria meningitidis	NG	W	W	16	1	1	60	4	11	5	18	8	11	4	21	ST-22 complex	184	4	4	2
4333	OX9931648	UK [England]	1999	carrier	Neisseria meningitidis	NG	Y	Y	23	1	1	4	4	9	7	6	17	11	18	17	ST-167 complex	1636	4	4	2
57022	OX9931653	UK [England]	1999	carrier	Neisseria meningitidis	W	W	W	16	1	1	60	4	11	5	18	8	11	24	21	ST-22 complex	22	4	4	2
57024	OX9931667	UK [England]	1999	carrier	Neisseria meningitidis	NG	W	W	16	1	1	60	4	11	5	18	8	11	4	21	ST-22 complex	184	4	4	2
4419	OX9931668	UK [England]	1999	carrier	Neisseria meningitidis	B	B	B	24	1	4	25	4	4	10	47	17	6	2	12	ST-35 complex	278	4	4	2
3411	OX9931673	UK [England]	1999	carrier	Neisseria meningitidis	E	E	E	13	3	1	145	3	17	5	19	17	3	6	2	ST-60 complex	1677	3	3	1
4217	OX9931674	UK [England]	1999	carrier	Neisseria meningitidis	E	E	E	13	3	57	209	3	8	6	7	17	21	26	49	ST-1157 complex	1419	3	3	1
4337	OX9931675	UK [England]	1999	carrier	Neisseria meningitidis	Y	Y	Y	104	1	57	10	4	10	5	18	9	11	9	17	ST-23 complex	23	4	4	2
3412	OX9931676	UK [England]	1999	carrier	Neisseria meningitidis	NG	B	B	19	1	1	65	4	9	6	15	9	9	6	18	ST-41/44 complex	1228	4	4	2
4324	OX9931681	UK [England]	1999	carrier	Neisseria meningitidis	W	W	W	16	NA	1	60	197	11	5	18	8	11	4	21	ST-22 complex	184	NA	NA	2
3413	OX9931682	UK [England]	1999	carrier	Neisseria meningitidis	E	E	E	13	3	57	209	3	8	6	7	17	21	26	49	ST-1157 complex	1419	3	3	1
4379	OX9931685	UK [England]	1999	carrier	Neisseria meningitidis	NG	cnl	cnl	102	8	1	2	20	16	2	6	25	17	25	22	ST-53 complex	53	20	4	2
4187	OX9931687	UK [England]	1999	carrier	Neisseria meningitidis	B	B	B	21	1	1	7	4	1	5	13	53	26	41	3	ST-162 complex	162	4	4	2
57037	OX9931705	UK [England]	1999	carrier	Neisseria meningitidis	NG	W	W	16	1	1	60	4	11	5	18	8	11	157	21	ST-22 complex	1667	4	4	2
28885	OX9931715	UK [England]	1999	carrier	Neisseria meningitidis	E	cnl	discrepancy	14	7	1	142	6	3	6	9	5	9	6	9	ST-41/44 complex	41	6	2	1
3414	OX9931731	UK [England]	1999	carrier	Neisseria meningitidis	E	cnl	discrepancy	102	8	1	2	20	16	2	6	25	17	25	22	ST-53 complex	53	20	4	2
915	OX9931754	UK [England]	1999	carrier	Neisseria meningitidis	W	W	W	16	1	1	60	4	11	5	18	8	174	24	21	ST-22 complex	1673	4	4	2
4362	OX9931770	UK [England]	1999	carrier	Neisseria meningitidis	NG	E	E	13	3	57	145	3	2	16	12	11	123	22	6	ST-254 complex	1827	3	3	1
3415	OX9931776	UK [England]	1999	carrier	Neisseria meningitidis	B	B	B	187	1	62	77	4	7	5	4	13	36	6	15	ST-213 complex	1644	4	4	3
4267	OX9931781	UK [England]	1999	carrier	Neisseria meningitidis	NG	cnl	cnl	94	58	56	89	NA	11	4	38	15	22	40	13	ST-1136 complex	1630	NA	NA	3
35898	OX9931800	UK [England]	1999	carrier	Neisseria meningitidis		B	B	15	4	2	193	1	4	10	15	9	8	11	9	ST-269 complex	269	1	1	1
4182	OX9931817	UK [England]	1999	carrier	Neisseria meningitidis	B	B	B	21	1	1	7	4	1	5	13	53	26	41	3	ST-162 complex	162	4	4	2
57044	OX9931819	UK [England]	1999	carrier	Neisseria meningitidis	W	W	W	16	1	1	60	4	11	5	18	8	11	157	21	ST-22 complex	1667	4	4	2
57047	OX9931822	UK [England]	1999	carrier	Neisseria meningitidis	Y	Y	Y	25	1	1	26	4	10	5	18	9	11	9	17	ST-23 complex	23	4	4	2
57049	OX9931826	UK [England]	1999	carrier	Neisseria meningitidis	NG	cnl	cnl	21	1	1	7	4	16	2	159	92	77	25	112	ST-1117 complex	1117	4	4	2
57051	OX9931829	UK [England]	1999	carrier	Neisseria meningitidis	B	B	B	25	1	1	26	4	8	3	6	17	29	18	9	ST-103 complex	1418	4	4	2
57053	OX9931834	UK [England]	1999	carrier	Neisseria meningitidis	W	W	W	16	1	1	60	4	11	5	18	8	11	157	21	ST-22 complex	1667	4	4	2
57054	OX9931840	UK [England]	1999	carrier	Neisseria meningitidis	NG	W	W	16	1	1	60	4	11	5	18	8	11	157	21	ST-22 complex	1667	4	4	2
28886	OX9931875	UK [England]	1999	carrier	Neisseria meningitidis	B	B	B	19	1	1	65	4	12	6	9	9	9	6	9	ST-41/44 complex	43	4	4	2
57056	OX9931876	UK [England]	1999	carrier	Neisseria meningitidis	NG	E	E	13	3	57	209	3	8	25	7	17	21	26	49	ST-1157 complex	1157	3	3	1
57058	OX9931878	UK [England]	1999	carrier	Neisseria meningitidis	Y	Y	Y	25	1	1	26	4	2	5	18	17	11	24	21	ST-22 complex	2180	4	4	2
57059	OX9931879	UK [England]	1999	carrier	Neisseria meningitidis	NG	W	W	16	1	1	60	4	11	5	18	8	11	157	21	ST-22 complex	1667	4	4	2
57062	OX9931894	UK [England]	1999	carrier	Neisseria meningitidis	W	W	W	16	1	1	60	4	11	5	18	8	11	157	21	ST-22 complex	1667	4	4	2
57026	OX9931897	UK [England]	1999	carrier	Neisseria meningitidis	W	W	W	16	1	1	60	4	11	5	18	8	174	24	21	ST-22 complex	1673	4	4	2
57028	OX9931901	UK [England]	1999	carrier	Neisseria meningitidis	W	W	W	16	1	1	60	4	11	5	5	8	11	24	21	ST-22 complex	1617	4	4	2
57064	OX9931905	UK [England]	1999	carrier	Neisseria meningitidis	Y	Y	Y	25	1	1	26	4	10	5	18	9	11	9	17	ST-23 complex	23	4	4	2
916	OX9931909	UK [England]	1999	carrier	Neisseria meningitidis	NG	Y	Y	16	1	1	60	4	11	5	6	8	11	24	21	ST-22 complex	1674	4	4	2
28887	OX9931911	UK [England]	1999	carrier	Neisseria meningitidis	B	B	B	19	1	1	65	4	12	6	9	9	9	6	9	ST-41/44 complex	43	4	4	2
57067	OX9931912	UK [England]	1999	carrier	Neisseria meningitidis	NG	E	E	13	3	57	209	3	8	25	7	17	21	26	49	ST-1157 complex	1157	3	3	1
3417	OX9931937	UK [England]	1999	carrier	Neisseria meningitidis	NG	B	B	25	1	1	26	4	25	10	105	2	5	13	8		2457	4	4	2
35889	OX9931942	UK [England]	1999	carrier	Neisseria meningitidis		B	B	21	1	1	7	4	1	5	13	53	26	41	3	ST-162 complex	162	4	4	2
3418	OX9931958	UK [England]	1999	carrier	Neisseria meningitidis	E	E	E	13	3	57	145	3	2	16	12	227	169	60	7	ST-254 complex	2360	3	3	1
57070	OX9931960	UK [England]	1999	carrier	Neisseria meningitidis	NG	cnl	cnl	297	15	1	276	58	16	2	6	25	17	25	22	ST-53 complex	53	NA	NA	1
56938	OX9931963	UK [England]	1999	carrier	Neisseria meningitidis	NG	Y	Y	23	1	1	4	4	9	7	6	17	11	18	17	ST-167 complex	1636	4	4	2
36225	OX9931966	UK [England]	1999	carrier	Neisseria meningitidis	B	B	B	338	7	1	NA	6	3	6	9	5	9	6	9	ST-41/44 complex	41	6	2	1
28889	OX9931967	UK [England]	1999	carrier	Neisseria meningitidis	NG	B	B	19	1	1	65	4	9	6	9	9	9	64	9	ST-41/44 complex	409	4	4	2
56940	OX9931970	UK [England]	1999	carrier	Neisseria meningitidis	NG	B	B	25	1	1	26	4	8	4	6	17	5	18	2	ST-103 complex	103	4	4	2
3419	OX9931971	UK [England]	1999	carrier	Neisseria meningitidis	E	cnl	discrepancy	297	15	1	447	58	16	2	6	25	17	25	22	ST-53 complex	53	NA	NA	1
3420	OX9931975	UK [England]	1999	carrier	Neisseria meningitidis	B	B	B	21	1	1	7	4	1	NA	13	17	26	41	3		NA	4	4	2
56942	OX9931981	UK [England]	1999	carrier	Neisseria meningitidis	B	B	B	21	1	1	7	4	1	5	13	53	26	41	3	ST-162 complex	162	4	4	2
4184	OX9931982	UK [England]	1999	carrier	Neisseria meningitidis	B	B	B	21	1	1	7	4	1	5	13	53	26	41	3	ST-162 complex	162	4	4	2
4228	OX9931983	UK [England]	1999	carrier	Neisseria meningitidis	B	B	B	16	1	4	59	4	4	10	11	11	6	18	12	ST-35 complex	3073	4	4	2
56944	OX9931984	UK [England]	1999	carrier	Neisseria meningitidis	B	B	B	21	1	1	7	4	1	5	13	53	26	41	3	ST-162 complex	162	4	4	2
28890	OX9931990	UK [England]	1999	carrier	Neisseria meningitidis	B	B	B	19	1	1	65	4	12	6	9	9	9	6	9	ST-41/44 complex	43	4	4	2
28891	OX9932003	UK [England]	1999	carrier	Neisseria meningitidis	B	B	B	19	1	2	65	4	9	6	9	9	9	6	9	ST-41/44 complex	44	4	4	2
56946	OX9932006	UK [England]	1999	carrier	Neisseria meningitidis	B	B	B	45	8	77	87	20	7	5	1	13	36	53	15	ST-213 complex	213	20	4	3
57030	OX9932010	UK [England]	1999	carrier	Neisseria meningitidis	W	W	W	16	1	1	60	4	11	5	5	8	11	24	21	ST-22 complex	1617	4	4	2
28892	OX9932015	UK [England]	1999	carrier	Neisseria meningitidis	B	B	B	19	1	1	65	4	3	6	9	24	9	38	9	ST-41/44 complex	1414	4	4	2
56948	OX9932018	UK [England]	1999	carrier	Neisseria meningitidis	NG	NG	NG	16	1	4	59	4	4	10	11	18	6	10	12	ST-35 complex	35	4	4	2
28893	OX9932020	UK [England]	1999	carrier	Neisseria meningitidis	NG	B	B	338	7	1	NA	6	3	6	9	5	9	6	9	ST-41/44 complex	41	6	2	1
56949	OX9932027	UK [England]	1999	carrier	Neisseria meningitidis	NG	E	E	13	3	57	209	3	8	25	7	17	21	26	49	ST-1157 complex	1157	3	3	1
5988	OX9932037	UK [England]	1999	carrier	Neisseria meningitidis	E	cnl	discrepancy	102	8	1	2	20	16	2	6	25	17	25	22	ST-53 complex	53	20	4	2
56953	OX9932061	UK [England]	1999	carrier	Neisseria meningitidis	NG	E	E	13	3	1	209	3	8	25	7	17	21	26	49	ST-1157 complex	1157	3	3	1
56954	OX9932065	UK [England]	1999	carrier	Neisseria meningitidis	NG	Y	Y	25	1	1	26	4	10	5	18	9	11	9	17	ST-23 complex	23	4	4	2
56956	OX9932070	UK [England]	1999	carrier	Neisseria meningitidis	NG	Y	Y	23	1	1	4	4	2	7	6	17	16	18	8	ST-167 complex	167	4	4	2
3421	OX9932075	UK [England]	1999	carrier	Neisseria meningitidis	NG	E	E	125	1	57	268	13	7	16	6	10	3	56	13	ST-178 complex	2392	13	3	1
56957	OX9932076	UK [England]	1999	carrier	Neisseria meningitidis	E	E	E	13	3	1	145	3	17	5	19	10	3	26	2	ST-60 complex	1383	3	3	1
4315	OX9932077	UK [England]	1999	carrier	Neisseria meningitidis	B	B	B	336	1	1	9	4	9	6	9	9	9	6	2	ST-41/44 complex	180	4	4	2
56959	OX9932082	UK [England]	1999	carrier	Neisseria meningitidis	B	B	B	19	1	1	83	4	4	10	2	5	3	11	9	ST-269 complex	1163	4	4	2
56961	OX9932083	UK [England]	1999	carrier	Neisseria meningitidis	NG	C	C	94	218	1	69	NA	6	41	9	9	171	20	180		1958	NA	NA	3
14923	OX9932088	UK [England]	1999	carrier	Neisseria meningitidis	B	B	B	19	1	1	65	4	3	6	9	60	9	26	9	ST-41/44 complex	1415	4	4	2
28894	OX9932093	UK [England]	1999	carrier	Neisseria meningitidis	B	B	B	19	1	1	65	4	3	6	9	60	9	26	9	ST-41/44 complex	1415	4	4	2
56964	OX9932094	UK [England]	1999	carrier	Neisseria meningitidis	NG	cnl	cnl	94	138	56	69	NA	5	4	17	15	14	7	12	ST-198 complex	198	NA	NA	3
917	OX9932097	UK [England]	1999	carrier	Neisseria meningitidis	NG	B	B	23	250	1	431	196	137	5	15	17	8	21	2	ST-865 complex	1675	NA	NA	2
918	OX9932099	UK [England]	1999	carrier	Neisseria meningitidis	B	B	B	19	1	1	65	4	12	41	9	9	9	166	9	ST-41/44 complex	1676	4	4	2
56967	OX9932133	UK [England]	1999	carrier	Neisseria meningitidis	E	cnl	discrepancy	102	8	1	2	20	16	2	6	25	17	25	22	ST-53 complex	53	20	4	2
14596	OX9932139	UK [England]	1999	carrier	Neisseria meningitidis	NG	cnl	cnl	94	58	56	89	NA	11	4	38	15	22	40	13	ST-1136 complex	1630	NA	NA	3
3422	OX9932142	UK [England]	1999	carrier	Neisseria meningitidis	NG	B	B	19	1	1	65	4	9	20	9	9	9	6	2	ST-41/44 complex	1097	4	4	2
56968	OX9932164	UK [England]	1999	carrier	Neisseria meningitidis	NG	E	E	125	1	57	268	13	7	16	55	10	3	17	46	ST-178 complex	2393	13	3	1
35885	OX9932169	UK [England]	1999	carrier	Neisseria meningitidis		B	B	15	4	2	193	1	4	10	15	9	8	11	9	ST-269 complex	269	1	1	1
28895	OX9932180	UK [England]	1999	carrier	Neisseria meningitidis	B	B	B	19	1	1	65	4	9	20	9	9	9	6	2	ST-41/44 complex	1097	4	4	2
56969	OX9932190	UK [England]	1999	carrier	Neisseria meningitidis	B	B	B	15	10	2	193	17	4	10	15	9	8	11	9	ST-269 complex	269	NA	NA	1
919	OX9932203	UK [England]	1999	carrier	Neisseria meningitidis	NG	E	E	13	3	1	145	3	17	5	19	17	3	6	2	ST-60 complex	1677	3	3	1
3423	OX9932210	UK [England]	1999	carrier	Neisseria meningitidis	NG	B	B	16	1	4	59	4	4	103	11	13	6	10	12	ST-35 complex	2381	4	4	2
35888	OX9932212	UK [England]	1999	carrier	Neisseria meningitidis		cnl	cnl	94	138	56	69	NA	5	4	17	15	14	181	12	ST-198 complex	1956	NA	NA	3
57032	OX9932215	UK [England]	1999	carrier	Neisseria meningitidis	NG	W	W	16	1	1	60	4	11	5	18	8	11	24	21	ST-22 complex	22	4	4	2
56974	OX9932222	UK [England]	1999	carrier	Neisseria meningitidis	NG	E	E	13	3	57	209	3	8	25	7	17	21	26	49	ST-1157 complex	1157	3	3	1
920	OX9932231	UK [England]	1999	carrier	Neisseria meningitidis	E	E	E	13	3	57	145	3	8	5	12	9	8	22	7		1678	3	3	1
56976	OX9932238	UK [England]	1999	carrier	Neisseria meningitidis	NG	C	C	19	1	1	65	4	8	26	9	24	26	20	18	ST-212 complex	212	4	4	2
56978	OX9932241	UK [England]	1999	carrier	Neisseria meningitidis	X	X	X	84	12	1	70	28	2	5	2	9	15	20	5	ST-750 complex	750	NA	NA	3
56979	OX9932245	UK [England]	1999	carrier	Neisseria meningitidis	E	X	discrepancy	13	3	57	209	3	8	25	7	17	21	26	49	ST-1157 complex	1157	3	3	1
4402	OX9932247	UK [England]	1999	carrier	Neisseria meningitidis	B	B	B	0	NA	59	NA	NA	43	5	12	11	26	19	15		3252	NA	NA	NA
56981	OX9932257	UK [England]	1999	carrier	Neisseria meningitidis	E	L	discrepancy	13	3	57	145	3	57	5	2	35	6	68	20		963	3	3	1
56983	OX9932259	UK [England]	1999	carrier	Neisseria meningitidis	NG	cnl	cnl	102	8	1	2	20	16	2	6	25	17	25	22	ST-53 complex	53	20	4	2
56986	OX9932263	UK [England]	1999	carrier	Neisseria meningitidis	B	B	B	202	1	1	35	4	42	26	46	24	6	20	17	ST-282 complex	1802	4	4	2
56988	OX9932264	UK [England]	1999	carrier	Neisseria meningitidis	E	Z	discrepancy	25	1	1	26	4	8	4	6	17	5	18	2	ST-103 complex	103	4	4	2
56990	OX9932270	UK [England]	1999	carrier	Neisseria meningitidis	NG	L	L	13	3	57	145	3	57	5	2	35	6	68	20		963	3	3	1
56992	OX9932271	UK [England]	1999	carrier	Neisseria meningitidis	W	W	W	22	1	1	1	4	2	5	NA	17	11	24	21		NA	4	4	2
56993	OX9932274	UK [England]	1999	carrier	Neisseria meningitidis	NG	B	B	45	8	77	87	20	7	5	1	13	36	53	15	ST-213 complex	213	20	4	3
921	OX9932276	UK [England]	1999	carrier	Neisseria meningitidis	C	C	C	16	1	4	59	4	10	10	11	18	170	10	129	ST-35 complex	1679	4	4	2
57034	OX9932278	UK [England]	1999	carrier	Neisseria meningitidis	W	W	W	16	1	1	60	4	11	5	18	8	11	4	21	ST-22 complex	184	4	4	2
56994	OX9932279	UK [England]	1999	carrier	Neisseria meningitidis	NG	E	E	13	3	57	209	3	8	25	7	17	21	26	49	ST-1157 complex	1157	3	3	1
922	OX9932287	UK [England]	1999	carrier	Neisseria meningitidis	B	B	B	144	1	1	1096	4	8	102	5	4	5	3	8	ST-32 complex	1680	4	3	1
57036	OX9932288	UK [England]	1999	carrier	Neisseria meningitidis	W	W	W	16	1	1	60	4	11	5	18	8	11	4	21	ST-22 complex	184	4	4	2
56998	OX9932291	UK [England]	1999	carrier	Neisseria meningitidis	NG	cnl	cnl	102	8	1	2	20	16	2	6	25	17	25	22	ST-53 complex	53	20	4	2
56999	OX9932293	UK [England]	1999	carrier	Neisseria meningitidis	NG	cnl	cnl	102	8	1	2	20	16	2	6	25	17	25	22	ST-53 complex	53	20	4	2
923	OX9932310	UK [England]	1999	carrier	Neisseria meningitidis	NG	cnl	cnl	94	NA	56	69	NA	5	4	17	15	14	7	12	ST-198 complex	198	NA	NA	3
57000	OX9932315	UK [England]	1999	carrier	Neisseria meningitidis	C	C	C	15	4	2	193	1	4	10	15	9	8	11	13	ST-269 complex	467	1	1	1
36226	OX9932316	UK [England]	1999	carrier	Neisseria meningitidis	B	B	B	4	2	1	84	2	3	6	9	5	9	6	9	ST-41/44 complex	41	2	3	1
4367	OX9932317	UK [England]	1999	carrier	Neisseria meningitidis		C	C	34	41	1	3	51	13	5	181	5	3	8	9		13204	NA	NA	2
4321	OX9932320	UK [England]	1999	carrier	Neisseria meningitidis	NG	B	B	19	1	1	65	4	9	20	9	9	9	6	2	ST-41/44 complex	1097	4	4	2
35896	OX9932322	UK [England]	1999	carrier	Neisseria meningitidis		E	E	13	3	1	209	3	NA	25	7	17	21	26	2		NA	3	3	1
4369	OX9932346	UK [England]	1999	carrier	Neisseria meningitidis	NG	cnl	cnl	102	8	1	2	20	16	2	6	25	17	25	22	ST-53 complex	53	20	4	2
57002	OX9932370	UK [England]	1999	carrier	Neisseria meningitidis	NG	cnl	cnl	102	NA	1	NA	NA	16	2	6	25	17	25	254	ST-53 complex	3671	NA	NA	2
4335	OX9932374	UK [England]	1999	carrier	Neisseria meningitidis	NG	W	W	16	1	1	60	4	11	5	18	8	11	4	21	ST-22 complex	184	4	4	2
57004	OX9932383	UK [England]	1999	carrier	Neisseria meningitidis	Z	Z	Z	13	3	57	145	3	8	4	6	17	5	18	2	ST-103 complex	103	3	3	1
36227	OX9932386	UK [England]	1999	carrier	Neisseria meningitidis	NG	B	B	4	2	1	84	2	3	6	9	5	9	6	9	ST-41/44 complex	41	2	3	1
28898	OX9932389	UK [England]	1999	carrier	Neisseria meningitidis	B	B	B	336	1	1	9	4	9	6	9	9	9	6	2	ST-41/44 complex	180	4	4	2
57005	OX9932399	UK [England]	1999	carrier	Neisseria meningitidis	Z	Z	Z	25	1	1	26	4	8	4	6	17	5	18	2	ST-103 complex	103	4	4	2
3427	OX9932400	UK [England]	1999	carrier	Neisseria meningitidis	NG	B	B	13	3	4	145	3	4	10	11	8	6	10	12	ST-35 complex	809	3	3	1
924	OX9932406	UK [England]	1999	carrier	Neisseria meningitidis	NG	C	C	15	4	2	193	1	4	10	15	32	8	11	13	ST-269 complex	1682	1	1	1

14597	OX9932412	UK [England]	1999	carrier	Neisseria meningitidis	NG	C	C	24	1	1	25	4	6	6	9	9	9	6	17	ST-41/44 complex	833	4	4	2
49839	PL40022	UK [England]	2015	carrier	Neisseria meningitidis	NG	B	B	510	NA	8	599	NA	4	10	48	4	6	3	8	ST-32 complex	7460	NA	NA	1
49841	PL40074	UK [England]	2015	carrier	Neisseria meningitidis	NG	cnl	cnl	21	1	1	7	4	16	2	159	92	77	25	112	ST-1117 complex	1117	4	4	2
49842	PL40092	UK [England]	2015	carrier	Neisseria meningitidis	NG	cnl	cnl	21	1	1	7	4	16	2	159	92	77	25	112	ST-1117 complex	1117	4	4	2
49843	PL40124	UK [England]	2015	carrier	Neisseria meningitidis	NG	E	E	13	3	57	209	3	8	25	7	17	21	813	49	ST-1157 complex	12580	3	3	1
46724	PL40144	UK [England]	2015	carrier	Neisseria meningitidis	NG	cnl	cnl	16	1	1	60	4	3	6	9	8	9	6	9	ST-41/44 complex	3617	4	4	2
49846	PL40186	UK [England]	2015	carrier	Neisseria meningitidis	NG	Y	Y	25	1	1	26	4	12	5	18	9	11	9	17	ST-23 complex	1655	4	4	2
49847	PL40195	UK [England]	2015	carrier	Neisseria meningitidis	NG		NG	25	1	1	26	4	12	5	18	9	11	25	17	ST-23 complex	10458	4	4	2
50934	PL40211	UK [England]	2015	carrier	Neisseria meningitidis		E	E	13	NA	1	145	NA	17	5	19	17	3	26	12	ST-60 complex	2002	NA	NA	1
46725	PL40213	UK [England]	2015	carrier	Neisseria meningitidis	B	B	B	13	3	57	145	3	4	10	34	5	38	11	9	ST-269 complex	1161	3	3	1
52660	PL40261	UK [England]	2015	carrier	Neisseria meningitidis	Y	Y	Y	25	1	1	26	4	12	5	18	9	11	9	17	ST-23 complex	1655	4	4	2
49849	PL40271	UK [England]	2015	carrier	Neisseria meningitidis	NG	cnl	cnl	102	8	1	2	20	16	2	6	25	17	25	22	ST-53 complex	53	20	4	2
49850	PL40347	UK [England]	2015	carrier	Neisseria meningitidis	B	B	B	19	1	56	65	4	9	6	351	9	536	21	311		8379	4	4	2
49851	PL40360	UK [England]	2015	carrier	Neisseria meningitidis	NG	E	E	13	3	1	145	3	17	5	19	17	3	26	2	ST-60 complex	60	3	3	1
49852	PL40379	UK [England]	2015	carrier	Neisseria meningitidis	Y	Y	Y	25	1	1	26	4	10	5	18	9	11	810	17	ST-23 complex	12578	4	4	2
49853	PL40384	UK [England]	2015	carrier	Neisseria meningitidis	NG	B	B	19	1	1	65	4	3	6	9	5	9	6	70	ST-41/44 complex	414	4	4	2
49855	PL40423	UK [England]	2015	carrier	Neisseria meningitidis	NG	Y	Y	25	1	1	26	4	12	5	18	9	11	9	17	ST-23 complex	1655	4	4	2
50053	PL40457	UK [England]	2015	carrier	Neisseria meningitidis		B	B	24	1	63	25	4	27	6	9	9	9	6	16	ST-41/44 complex	1213	4	4	2
49856	PL40465	UK [England]	2015	carrier	Neisseria meningitidis	NG	B	B	19	1	2	65	4	6	6	63	9	9	11	9	ST-41/44 complex	839	4	4	2
49857	PL40477	UK [England]	2015	carrier	Neisseria meningitidis	NG	B	B	13	3	57	209	3	8	25	7	17	21	26	49	ST-1157 complex	1157	3	3	1
49858	PL40489	UK [England]	2015	carrier	Neisseria meningitidis	B	B	B	19	1	2	65	4	6	6	63	9	9	11	9	ST-41/44 complex	839	4	4	2
49859	PL40493	UK [England]	2015	carrier	Neisseria meningitidis	NG	E	E	13	3	57	209	3	8	25	7	17	21	26	49	ST-1157 complex	1157	3	3	1
50936	PL40501	UK [England]	2015	carrier	Neisseria meningitidis	NG	C	C	16	1	1	60	4	132	3	19	17	62	21	2		1434	4	4	2
49860	PL40532	UK [England]	2015	carrier	Neisseria meningitidis	NG	cnl	cnl	4	1	1	206	4	5	4	17	15	30	7	12	ST-198 complex	823	4	3	1
49861	PL40535	UK [England]	2015	carrier	Neisseria meningitidis	NG	E	E	13	3	57	209	3	8	25	7	17	21	26	49	ST-1157 complex	1157	3	3	1
49862	PL40538	UK [England]	2015	carrier	Neisseria meningitidis	NG	E	E	13	3	57	209	3	8	25	7	17	21	26	49	ST-1157 complex	1157	3	3	1
50937	PL40549	UK [England]	2015	carrier	Neisseria meningitidis	NG	E	E	13	3	57	209	3	8	25	7	17	21	26	49	ST-1157 complex	1157	3	3	1
52661	PL40555	UK [England]	2015	carrier	Neisseria meningitidis	NG	B	B	45	8	77	87	20	7	5	1	13	36	53	15	ST-213 complex	213	20	4	3
49866	PL40562	UK [England]	2015	carrier	Neisseria meningitidis	NG	Y	Y	25	1	1	26	4	10	5	18	9	11	9	17	ST-23 complex	23	4	4	2
49867	PL40582	UK [England]	2015	carrier	Neisseria meningitidis	NG	cnl	cnl	102	8	1	2	20	16	2	6	25	17	25	22	ST-53 complex	53	20	4	2
49868	PL40588	UK [England]	2015	carrier	Neisseria meningitidis	Y	Y	Y	25	1	1	26	4	12	5	18	9	11	9	17	ST-23 complex	1655	4	4	2
49869	PL40600	UK [England]	2015	carrier	Neisseria meningitidis	NG	cnl	cnl	21	1	1	7	4	16	2	159	92	77	25	112	ST-1117 complex	1117	4	4	2
49871	PL40630	UK [England]	2015	carrier	Neisseria meningitidis	NG	cnl	cnl	4	1	1	206	4	5	4	17	15	30	7	12	ST-198 complex	823	4	3	1
49872	PL40635	UK [England]	2015	carrier	Neisseria meningitidis	NG	B	B	45	8	77	87	20	7	5	1	13	36	53	15	ST-213 complex	213	20	4	3
49875	PL40755	UK [England]	2015	carrier	Neisseria meningitidis	B	B	B	49	1	57	102	13	4	10	5	4	6	3	8	ST-32 complex	32	13	4	2
49876	PL40778	UK [England]	2015	carrier	Neisseria meningitidis	NG	E	E	13	3	57	209	3	8	25	7	17	21	26	49	ST-1157 complex	1157	3	3	1
49877	PL40789	UK [England]	2015	carrier	Neisseria meningitidis	W	W	W	16	1	1	60	4	11	5	18	8	11	4	21	ST-22 complex	184	4	4	2
49878	PL40791	UK [England]	2015	carrier	Neisseria meningitidis	W	W	W	16	1	1	60	4	11	5	18	8	11	4	21	ST-22 complex	184	4	4	2
49879	PL40797	UK [England]	2015	carrier	Neisseria meningitidis	NG	E	E	13	3	1	145	3	17	5	19	17	3	26	2	ST-60 complex	60	3	3	1
49880	PL40804	UK [England]	2015	carrier	Neisseria meningitidis	NG	NG	NG	23	1	1	4	4	2	7	6	17	16	18	8	ST-167 complex	167	4	4	2
49882	PL40814	UK [England]	2015	carrier	Neisseria meningitidis	NG	cnl	cnl	985	8	10	787	20	16	2	6	25	17	25	22	ST-53 complex	53	20	4	2
49883	PL40856	UK [England]	2015	carrier	Neisseria meningitidis	Y	Y	Y	25	1	1	26	4	12	5	18	9	11	9	17	ST-23 complex	1655	4	4	2
52662	PL40857	UK [England]	2015	carrier	Neisseria meningitidis	W	W/Y	discrepancy	25	1	1	26	4	12	5	18	9	11	9	17	ST-23 complex	1655	4	4	2
49885	PL40887	UK [England]	2015	carrier	Neisseria meningitidis	NG	cnl	cnl	19	1	1	83	4	4	10	2	5	3	11	9	ST-269 complex	1163	4	4	2
49886	PL40947	UK [England]	2015	carrier	Neisseria meningitidis	Y	Y	Y	25	1	1	26	4	10	5	18	9	11	9	17	ST-23 complex	23	4	4	2
49887	PL40967	UK [England]	2015	carrier	Neisseria meningitidis	NG	cnl	cnl	94	58	56	89	NA	5	4	38	15	22	40	13	ST-1136 complex	1136	NA	NA	3
49888	PL40971	UK [England]	2015	carrier	Neisseria meningitidis	NG	cnl	cnl	985	8	10	787	20	16	2	6	25	17	25	22	ST-53 complex	53	20	4	2
49889	PL40976	UK [England]	2015	carrier	Neisseria meningitidis	NG	cnl	cnl	94	58	56	89	NA	5	4	38	15	22	40	13	ST-1136 complex	1136	NA	NA	3
49890	PL40990	UK [England]	2015	carrier	Neisseria meningitidis	Y	Y	Y	25	1	1	26	4	12	286	18	9	11	9	17	ST-23 complex	6463	4	4	2
49892	PL41006	UK [England]	2015	carrier	Neisseria meningitidis	NG	B	B	125	1	57	268	13	8	5	19	17	5	24	54		3138	13	3	1
49893	PL41007	UK [England]	2015	carrier	Neisseria meningitidis	NG	cnl	cnl	985	8	10	787	20	16	2	6	25	17	25	22	ST-53 complex	53	20	4	2
49894	PL41022	UK [England]	2015	carrier	Neisseria meningitidis	NG	E	E	13	3	1	209	3	8	25	7	17	21	26	53	ST-1157 complex	12847	3	3	1
49895	PL41026	UK [England]	2015	carrier	Neisseria meningitidis	B	B	B	45	8	77	87	20	7	5	1	13	3	53	15	ST-213 complex	2101	20	4	3
49896	PL41028	UK [England]	2015	carrier	Neisseria meningitidis	NG	cnl	cnl	334	1	1	527	4	6	41	108	15	9	6	9		845	4	4	2
49897	PL41034	UK [England]	2015	carrier	Neisseria meningitidis	B	B	B	45	8	77	87	20	7	5	1	13	3	53	15	ST-213 complex	2101	20	4	3
49898	PL41036	UK [England]	2015	carrier	Neisseria meningitidis	NG	E	E	13	3	57	209	3	8	25	7	17	21	26	49	ST-1157 complex	1157	3	3	1
49899	PL41037	UK [England]	2015	carrier	Neisseria meningitidis	NG	E	E	499	15	57	134	58	8	25	7	17	21	26	49	ST-1157 complex	1157	NA	NA	3
49901	PL41050	UK [England]	2015	carrier	Neisseria meningitidis	NG	Y	Y	19	1	1	93	13	2	16	6	17	9	18	8	ST-167 complex	168	13	4	2
49904	PL41106	UK [England]	2015	carrier	Neisseria meningitidis	NG	E	E	13	3	57	209	3	8	25	7	17	21	26	49	ST-1157 complex	1157	3	3	1
49906	PL41139	UK [England]	2015	carrier	Neisseria meningitidis	Y	Y	Y	23	1	1	4	4	2	7	6	17	1	18	8	ST-167 complex	1627	4	4	2
49907	PL41143	UK [England]	2015	carrier	Neisseria meningitidis	NG	cnl	cnl	102	8	10	2	20	16	2	6	25	17	25	22	ST-53 complex	53	20	4	2
49909	PL41155	UK [England]	2015	carrier	Neisseria meningitidis	B	B	B	19	1	1	65	4	9	20	9	9	9	6	2	ST-41/44 complex	1097	4	4	2
49910	PL41161	UK [England]	2015	carrier	Neisseria meningitidis	NG	cnl	cnl	102	8	1	2	20	16	2	6	25	17	25	22	ST-53 complex	53	20	4	2
49911	PL41166	UK [England]	2015	carrier	Neisseria meningitidis	NG	Y	Y	25	1	1	26	4	10	5	18	9	11	9	17	ST-23 complex	23	4	4	2
49912	PL41167	UK [England]	2015	carrier	Neisseria meningitidis	Y	Y	Y	104	1	57	10	4	10	5	18	59	11	9	17	ST-23 complex	183	4	4	2
49913	PL41169	UK [England]	2015	carrier	Neisseria meningitidis	NG	W	W	16	1	1	60	4	11	5	18	8	11	4	21	ST-22 complex	184	4	4	2
49917	PL41192	UK [England]	2015	carrier	Neisseria meningitidis	NG	E	E	13	3	57	209	3	8	342	7	17	21	26	49	ST-1157 complex	10301	3	3	1
50938	PL41236	UK [England]	2015	carrier	Neisseria meningitidis	B	B	B	45	8	77	87	20	13	5	1	13	36	53	15	ST-213 complex	12848	20	4	3
49920	PL41254	UK [England]	2015	carrier	Neisseria meningitidis	W	W	W	16	1	1	60	4	11	5	18	8	11	4	21	ST-22 complex	184	4	4	2
49922	PL41276	UK [England]	2015	carrier	Neisseria meningitidis	NG	E	E	13	3	1	209	3	8	25	827	17	21	26	49	ST-1157 complex	12354	3	3	1
49924	PL41328	UK [England]	2015	carrier	Neisseria meningitidis	NG	E	E	13	3	57	209	3	8	25	7	17	21	26	49	ST-1157 complex	1157	3	3	1
49925	PL41345	UK [England]	2015	carrier	Neisseria meningitidis	Y	Y	Y	25	1	1	26	4	12	286	18	9	11	9	17	ST-23 complex	6463	4	4	2
49926	PL41355	UK [England]	2015	carrier	Neisseria meningitidis	NG	E	E	13	3	1	209	3	8	25	827	17	21	26	49	ST-1157 complex	12354	3	3	1
49928	PL41362	UK [England]	2015	carrier	Neisseria meningitidis	NG	E	E	13	3	1	209	3	8	25	827	17	21	26	49	ST-1157 complex	12354	3	3	1
49929	PL41375	UK [England]	2015	carrier	Neisseria meningitidis	Y	Y	Y	25	1	1	26	4	12	286	18	9	11	9	17	ST-23 complex	6463	4	4	2
49930	PL41392	UK [England]	2015	carrier	Neisseria meningitidis	NG	E	E	13	3	1	209	3	8	25	827	17	21	26	49	ST-1157 complex	12354	3	3	1
49931	PL41393	UK [England]	2015	carrier	Neisseria meningitidis	Y	Y	Y	25	1	1	26	4	12	286	18	9	11	9	17	ST-23 complex	6463	4	4	2
49932	PL41396	UK [England]	2015	carrier	Neisseria meningitidis	Y	Y	Y	25	1	1	26	4	12	286	18	9	11	9	17	ST-23 complex	6463	4	4	2
50238	PL41409	UK [England]	2015	carrier	Neisseria meningitidis	Y	Y	Y	25	1	1	26	4	10	5	18	9	11	810	17	ST-23 complex	12578	4	4	2
49934	PL41432	UK [England]	2015	carrier	Neisseria meningitidis	NG	E	E	13	3	57	209	3	8	25	7	17	21	26	49	ST-1157 complex	1157	3	3	1
49935	PL41433	UK [England]	2015	carrier	Neisseria meningitidis	Y	Y	Y	25	1	1	26	4	10	5	18	9	11	810	17	ST-23 complex	12578	4	4	2
52663	PL41434	UK [England]	2015	carrier	Neisseria meningitidis	NG	NG	NG	15	4	2	193	1	4	10	15	9	8	11	79	ST-269 complex	9823	1	1	1
49937	PL41446	UK [England]	2015	carrier	Neisseria meningitidis	Y	Y	Y	25	1	1	26	4	12	5	847	9	11	9	17	ST-23 complex	12677	4	4	2
49938	PL41463	UK [England]	2015	carrier	Neisseria meningitidis	W	W	W	16	1	1	60	4	11	5	18	8	11	4	21	ST-22 complex	184	4	4	2
49939	PL41465	UK [England]	2015	carrier	Neisseria meningitidis	W	W	W	16	1	1	60	4	11	5	18	8	11	4	21	ST-22 complex	184	4	4	2
49940	PL41500	UK [England]	2015	carrier	Neisseria meningitidis	Y	Y	Y	25	1	1	26	4	12	5	847	9	11	9	17	ST-23 complex	12677	4	4	2
49941	PL41535	UK [England]	2015	carrier	Neisseria meningitidis	NG	cnl	cnl	4	1	1	206	4	5	4	17	15	30	7	12	ST-198 complex	823	4	3	1
49942	PL41541	UK [England]	2015	carrier	Neisseria meningitidis	B	B	B	13	3	57	145	3	4	10	34	5	38	11	9	ST-269 complex	1161	3	3	1
52664	PL41580	UK [England]	2015	carrier	Neisseria meningitidis	Y	Y	Y	25	1	1	26	4	12	5	18	9	11	9	17	ST-23 complex	1655	4	4	2
49944	PL41581	UK [England]	2015	carrier	Neisseria meningitidis	NG	cnl	cnl	19	1	1	83	4	4	10	2	5	3	11	9	ST-269 complex	1163	4	4	2
49945	PL41582	UK [England]	2015	carrier	Neisseria meningitidis	NG	cnl	cnl	19	1	1	83	4	4	10	2	5	3	11	9	ST-269 complex	1163	4	4	2
49946	PL41583	UK [England]	2015	carrier	Neisseria meningitidis	NG	B	B	19	1	2	65	4	20	6	9	15	9	11	9	ST-41/44 complex	12589	4	4	2
50233	PL41608	UK [England]	2015	carrier	Neisseria meningitidis	Y	Y	Y	25	1	1	26	4	12	5	18	9	11	9	17	ST-23 complex	1655	4	4	2
46572	PL41617	UK [England]	2015	carrier	Neisseria meningitidis	NG	cnl	cnl	102	8	1	2	20	16	2	6	25	17	25	22	ST-53 complex	53	20	4	2
49947	PL41621	UK [England]	2015	carrier	Neisseria meningitidis	Y	Y	Y	25	1	1	26	4	12	5	18	9	11	25	17	ST-23 complex	10458	4	4	2
49948	PL41638	UK [England]	2015	carrier	Neisseria meningitidis	NG	E	E	13	3	57	209	3	8	25	7	17	21	26	49	ST-1157 complex	1157	3	3	1
49949	PL41642	UK [England]	2015	carrier	Neisseria meningitidis	Y	Y	Y	25	1	1	26	4	10	5	18	9	11	9	17	ST-23 complex	23	4	4	2
49950	PL41655	UK [England]	2015	carrier	Neisseria meningitidis	Y	Y	Y	25	1	1	26	4	12	5	18	9	11	9	17	ST-23 complex	1655	4	4	2
52665	PL41733	UK [England]	2015	carrier	Neisseria meningitidis	NG	W/Y	W/Y	25	1	1	26	4	12	5	18	9	11	9	17	ST-23 complex	1655	4	4	2
49953	PL41762	UK [England]	2015	carrier	Neisseria meningitidis	NG	E	E	13	3	57	209	3	8	25	7	17	21	26	49	ST-1157 complex	1157	3	3	1
49954	PL41763	UK [England]	2015	carrier	Neisseria meningitidis	NG	B	B	16	1	57	60	4	222	3	58	275	386	812	255	ST-4821 complex	12579	4	4	2
49955	PL41776	UK [England]	2015	carrier	Neisseria meningitidis	NG	cnl	cnl	499	15	10	134	58	16	2	6	25	17	25	22	ST-53 complex	53	NA	NA	3
49956	PL41792	UK [England]	2015	carrier	Neisseria meningitidis	NG	X	X	13	3	57	209	3	8	25	7	17	21	26	49	ST-1157 complex	1157	3	3	1
49957	PL41794	UK [England]	2015	carrier	Neisseria meningitidis	NG	Y	Y	25	1	1	26	4	12	286	18	9	11	9	17	ST-23 complex	6463	4	4	2
49958	PL41799	UK [England]	2015	carrier	Neisseria meningitidis	NG	Y	Y	104	1	57	10	4	10	5	18	59	11	9	17	ST-23 complex	183	4	4	2
49959	PL41811	UK [England]	2015	carrier	Neisseria meningitidis	NG	cnl	cnl	94	138	56	69	NA	5	4	17	17	14	7	12	ST-198 complex	2146	NA	NA	3
49960	PL41818	UK [England]	2015	carrier	Neisseria meningitidis	NG	NG	NG	151	1	1	130	4	6	7	4	56	26	18	8	ST-175 complex	175	4	4	2
49961	PL41825	UK [England]	2015	carrier	Neisseria meningitidis	NG	E	E	13	3	57	209	3	8	25	7	17	21	26	49	ST-1157 complex	1157	3	3	1
49963	PL41851	UK [England]	2015	carrier	Neisseria meningitidis	B	B	B	19	1	2	65	4	805	6	846	9	9	11	9	ST-41/44 complex	12680	4	4	2
49964	PL41900	UK [England]	2015	carrier	Neisseria meningitidis	NG	cnl	cnl	102	8	1	2	20	16	2	6	25	17	25	22	ST-53 complex	53	20	4	2
50315	PL41926	UK [England]	2015	carrier	Neisseria meningitidis	NG	Y	Y	16	1	1	60	4	219	5	275	17	11	8	21		3015	4	4	2
49965	PL41931	UK [England]	2015	carrier	Neisseria meningitidis	NG	Y	Y	16	1	1	60	4	219	5	275	17	11	8	21		3015	4	4	2
49966	PL41937	UK [England]	2015	carrier	Neisseria meningitidis	NG	cnl	cnl	102	8	10	2	20	16	2	6	25	17	25	22	ST-53 complex	53	20	4	2
49967	PL42019	UK [England]	2015	carrier	Neisseria meningitidis	Y	Y	Y	25	1	1	26	4	12	5	18	9	11	9	17	ST-23 complex	1655	4	4	2
49969	PL42044	UK [England]	2015	carrier	Neisseria meningitidis	NG	W	W	16	1	1	60	4	11	5	18	8	78	24	21	ST-22 complex	1224	4	4	2
49970	PL42046	UK [England]	2015	carrier	Neisseria meningitidis	B	B	B	45	8	77	87	20	7	5	1	13	36	53	15	ST-213 complex	213	20	4	3
52667	PL42080	UK [England]	2015	carrier	Neisseria meningitidis	NG	W	W	22	1	1	1	4	2	3	4	3	8	4	6	ST-11 complex	11	4	4	2
49973	PL42084	UK [England]	2015	carrier	Neisseria meningitidis	NG	Z	Z	16	56	1	60	NA	13	29	2	17	26	65	53		2123	NA	NA	2
49974	PL42095	UK [England]	2015	carrier	Neisseria meningitidis	NG	Y	Y	25	1	1	26	4	10	5	18	9	11	9	17	ST-23 complex	23	4	4	2
49975	PL42118	UK [England]	2015	carrier	Neisseria meningitidis	NG	Y	Y	25	1	1	26	4	10	5	18	9	11	9	17	ST-23 complex	23	4	4	2
52668	PL42154	UK [England]	2015	carrier	Neisseria meningitidis	NG	B	B	45	8	77	87	20	7	5	1	13	36	53	15	ST-213 complex	213	20	4	3
49977	PL42156	UK [England]	2015	carrier	Neisseria meningitidis	NG	cnl	cnl	102	8	10	2	20	16	2	6	25	17	25	22	ST-53 complex	53	20	4	2
29782	PM10B1	UK	2000	invasive (unspecified/other)	Neisseria meningitidis	C	C	C	453	5	1	182	11	2	3	4	3	8	4	6	ST-11 complex	11	11	2	1
29783	PM10B2	UK	2000	invasive (unspecified/other)	Neisseria meningitidis	C	C	C	453	5	1	182	11	2	3	4	3	8	4	6	ST-11 complex	11	11	2	1
29781	PM10Jo	UK	2000	invasive (unspecified/other)	Neisseria meningitidis	C	C	C	453	5	1	182	11	2	3	4	3	8	4	6	ST-11 complex	11	11	2	1
29790	PM11B	UK	2001	invasive (unspecified/other)	Neisseria meningitidis	C	C	C	95	5	1	40	11	2	3	4	3	8	4	6	ST-11 complex	11	11	2	2
29789	PM11CSF	UK	2001	invasive (unspecified/other)	Neisseria meningitidis	C	C	C	95	5	1	40	11	2	3	4	3	8	4	6	ST-11 complex	11	11	2	2
29791	PM11TS	UK	2001	invasive (unspecified/other)	Neisseria meningitidis	C	C	C	95	5	1	40	11	2	3	4	NA	8	4	6		NA	11	2	2
29813	PM12B	UK	2011	invasive (unspecified/other)	Neisseria meningitidis	W	W	W	22	1	1	1	4	2	3	4	3	8	4	6	ST-11 complex	11	4	4	2
29814	PM12TS	UK	2011	invasive (unspecified/other)	Neisseria meningitidis	W	W	W	22	1	1	1	4	2	3	4	3	8	4	6	ST-11 complex	11	4	4	2
29820	PM13B	UK	1998	invasive (unspecified/other)	Neisseria meningitidis	C	C	C	89	5	5	323	23	2	3	4	3	8	4	6	ST-11 complex	11	NA	NA	1
29821	PM13TS1	UK	1998	invasive (unspecified/other)	Neisseria meningitidis	C	C	C	89	5	5	323	23	2	3	4	3	8	4	6	ST-11 complex	11	NA	NA	1
29822	PM13TS2	UK	1998	invasive (unspecified/other)	Neisseria meningitidis	C	C	C	89	5	5	323	23	2	3	4	3	8	4	6	ST-11 complex	11	NA	NA	1
29761	PM14B	UK	1998	invasive (unspecified/other)	Neisseria meningitidis	C	C	C	22	1	1	1	4	2	3	4	24	8	4	6	ST-11 complex	67	4	4	2
29760	PM14CSF	UK	1998	invasive (unspecified/other)	Neisseria meningitidis	C	C	C	22	1	1	1	4	2	3	4	24	8	4	6	ST-11 complex	67	4	4	2
29762	PM14TS	UK	1998	invasive (unspecified/other)	Neisseria meningitidis	C	C	C	22	1	1	1	4	2	3	4	24	8	4	6	ST-11 complex	67	4	4	2
29816	PM16NS	UK	2012	invasive (unspecified/other)	Neisseria meningitidis	Y	Y	Y	24	1	1	27	4	2	7	6	17	9	6	8	ST-167 complex	1624	4	4	2
29798	PM17B	UK	2003	invasive (unspecified/other)	Neisseria meningitidis	B	B	B	37	1	1	408	4	7	8	10	19	10	1	2	ST-18 complex	18	4	3	1

29797	PM17NS	UK	2003	invasive (unspecified/other)	Neisseria meningitidis	B	B	B	37	1	1	408	4	7	8	10	19	10	1	2	ST-18 complex	18	4	3	1
29796	PM17TS	UK	2003	invasive (unspecified/other)	Neisseria meningitidis	B	B	B	37	1	1	408	4	7	8	10	19	10	1	2	ST-18 complex	18	4	3	1
29756	PM23B	UK	1998	invasive (unspecified/other)	Neisseria meningitidis	B	B	B	13	3	1	145	3	17	5	1	13	36	53	2	ST-213 complex	2127	3	3	1
20981	PM23CSF	UK	1998	invasive (unspecified/other)	Neisseria meningitidis		B	B	13	3	1	145	3	17	5	1	13	36	53	2	ST-213 complex	2127	3	3	1
29787	PM25B	UK	2000	invasive (unspecified/other)	Neisseria meningitidis	W	W	W	16	1	1	60	4	11	5	18	8	11	24	21	ST-22 complex	22	4	4	2
29788	PM25TS	UK	2000	invasive (unspecified/other)	Neisseria meningitidis	W	W	W	16	1	1	60	4	11	5	18	8	11	24	21	ST-22 complex	22	4	4	2
30124	PM26B	UK	1997	invasive (unspecified/other)	Neisseria meningitidis	W	W	W	16	1	1	60	4	11	5	18	8	11	24	21	ST-22 complex	22	4	4	2
30125	PM26Jo	UK	1997	invasive (unspecified/other)	Neisseria meningitidis	W	W	W	16	1	1	60	4	11	5	18	NA	11	24	21		NA	4	4	2
29804	PM28NS	UK	2003	invasive (unspecified/other)	Neisseria meningitidis	Y	Y	Y	25	1	1	26	4	10	5	18	9	11	9	17	ST-23 complex	23	4	4	2
29803	PM28TS1	UK	2003	invasive (unspecified/other)	Neisseria meningitidis	Y	Y	Y	25	1	1	26	4	10	5	18	9	11	9	17	ST-23 complex	23	4	4	2
29805	PM28TS2	UK	2003	invasive (unspecified/other)	Neisseria meningitidis	Y	Y	Y	25	1	1	26	4	10	5	18	9	11	9	17	ST-23 complex	23	4	4	2
29792	PM29B	UK	2001	invasive (unspecified/other)	Neisseria meningitidis	B	B	B	15	4	2	193	1	4	10	15	9	8	11	9	ST-269 complex	269	1	1	1
29794	PM29BM	UK	2001	invasive (unspecified/other)	Neisseria meningitidis	B	B	B	15	4	2	193	1	4	10	15	9	8	11	9	ST-269 complex	269	1	1	1
29793	PM29CSF	UK	2001	invasive (unspecified/other)	Neisseria meningitidis	B	B	B	15	4	2	193	1	4	10	15	9	8	11	9	ST-269 complex	269	1	1	1
30110	PM2B	UK	1999	invasive (unspecified/other)	Neisseria meningitidis	B	B	B	14	1	57	142	13	11	5	12	12	12	217	14		2823	13	3	1
30109	PM2CSF	UK	1999	invasive (unspecified/other)	Neisseria meningitidis	B	B	B	14	1	57	142	13	11	5	12	12	12	217	14		2823	13	3	1
30111	PM2NS	UK	1999	invasive (unspecified/other)	Neisseria meningitidis	B	B	B	14	1	57	142	13	11	5	12	12	12	217	14		2823	13	3	1
29795	PM35NS	UK	2002	invasive (unspecified/other)	Neisseria meningitidis	B	B	B	13	3	57	145	3	4	10	2	5	38	11	9	ST-269 complex	275	3	3	1
20975	PM38B	UK	1997	invasive (unspecified/other)	Neisseria meningitidis		B	B	13	3	57	145	3	4	10	2	5	38	11	9	ST-269 complex	275	3	3	1
29828	PM3CSF	UK	1998	invasive (unspecified/other)	Neisseria meningitidis	B	B	B	25	1	1	26	4	15	3	133	17	8	19	15		5507	4	4	2
29826	PM3NS	UK	1998	invasive (unspecified/other)	Neisseria meningitidis	B	B	B	25	1	1	26	4	15	3	133	17	8	19	15		5507	4	4	2
29827	PM3TS	UK	1998	invasive (unspecified/other)	Neisseria meningitidis	B	B	B	25	1	1	26	4	15	3	133	17	8	19	15		5507	4	4	2
20977	PM42CSF	UK	1997	invasive (unspecified/other)	Neisseria meningitidis		B	B	15	4	2	193	1	4	10	6	9	8	11	9	ST-269 complex	1092	1	1	1
29806	PM46TS2	UK	2004	invasive (unspecified/other)	Neisseria meningitidis	B	B	B	15	4	1	193	1	NA	10	15	9	5	11	17		NA	1	1	1
29749	PM53B	UK	1998	invasive (unspecified/other)	Neisseria meningitidis	B	B	B	1	6	3	100	7	8	10	5	4	6	3	8	ST-32 complex	33	7	1	1
29748	PM53CSF	UK	1998	invasive (unspecified/other)	Neisseria meningitidis	B	B	B	1	6	3	100	7	8	10	5	4	6	3	8	ST-32 complex	33	7	1	1
29747	PM53TS	UK	1998	invasive (unspecified/other)	Neisseria meningitidis	B	B	B	1	6	3	100	7	8	10	5	4	6	3	8	ST-32 complex	33	7	1	1
29812	PM54B	UK	2009	invasive (unspecified/other)	Neisseria meningitidis	B	B	B	1	6	3	100	7	8	10	5	4	5	3	8	ST-32 complex	34	7	1	1
29810	PM54NS	UK	2009	invasive (unspecified/other)	Neisseria meningitidis	B	B	B	1	6	3	100	7	8	10	5	4	5	3	8	ST-32 complex	34	7	1	1
29811	PM54TS	UK	2009	invasive (unspecified/other)	Neisseria meningitidis	B	B	B	1	6	3	100	7	8	10	5	4	5	3	8	ST-32 complex	34	7	1	1
29808	PM55B	UK	2005	invasive (unspecified/other)	Neisseria meningitidis	B	B	B	1	6	3	100	7	8	10	77	4	6	3	8	ST-32 complex	749	7	1	1
29807	PM55TS1	UK	2005	invasive (unspecified/other)	Neisseria meningitidis	B	B	B	1	6	3	100	7	8	10	77	4	6	3	8	ST-32 complex	749	7	1	1
29809	PM55TS2	UK	2005	invasive (unspecified/other)	Neisseria meningitidis	B	B	B	1	6	3	100	7	8	10	77	4	6	3	8	ST-32 complex	749	7	1	1
29817	PM57B	UK	1998	invasive (unspecified/other)	Neisseria meningitidis	B	B	B	1	6	7	100	7	702	10	5	4	6	3	8	ST-32 complex	11163	7	1	1
29819	PM57TS	UK	1998	invasive (unspecified/other)	Neisseria meningitidis	B	B	B	1	6	7	100	7	702	10	5	4	6	3	8	ST-32 complex	11163	7	1	1
29784	PM5B	UK	2000	invasive (unspecified/other)	Neisseria meningitidis	C	C	C	4	2	1	84	2	2	625	4	3	8	4	6	ST-11 complex	13244	2	3	1
29785	PM5NS1	UK	2000	invasive (unspecified/other)	Neisseria meningitidis	C	C	C	4	2	1	84	2	2	625	4	3	8	4	6	ST-11 complex	13244	2	3	1
29786	PM5NS2	UK	2000	invasive (unspecified/other)	Neisseria meningitidis	C	C	C	4	2	1	84	2	2	625	4	3	8	4	6	ST-11 complex	13244	2	3	1
30118	PM61CSF	UK	1999	invasive (unspecified/other)	Neisseria meningitidis	B	B	B	22	1	1	1	4	13	3	16	1	3	11	9	ST-364 complex	15	4	4	2
30119	PM61TS	UK	1999	invasive (unspecified/other)	Neisseria meningitidis	B	B	B	22	1	1	1	4	13	3	16	1	3	11	9	ST-364 complex	15	4	4	2
20905	PM62CSF	UK	1999	invasive (unspecified/other)	Neisseria meningitidis	B	B	B	4	2	1	84	2	3	6	9	5	9	6	9	ST-41/44 complex	41	2	3	1
20963	PM63B	UK	2007	invasive (unspecified/other)	Neisseria meningitidis	B	B	B	4	2	79	84	2	3	6	9	5	9	6	9	ST-41/44 complex	41	2	3	1
29742	PM64B	UK	1997	invasive (unspecified/other)	Neisseria meningitidis	B	B	B	4	2	1	84	2	3	6	9	5	9	6	9	ST-41/44 complex	41	2	3	1
29741	PM64CSF	UK	1997	invasive (unspecified/other)	Neisseria meningitidis	B	B	B	4	2	1	84	2	3	6	9	5	9	6	9	ST-41/44 complex	41	2	3	1
29743	PM64TS	UK	1997	invasive (unspecified/other)	Neisseria meningitidis	B	B	B	4	2	1	84	2	3	6	9	5	9	6	9	ST-41/44 complex	41	2	3	1
29764	PM65CSF	UK	2000	invasive (unspecified/other)	Neisseria meningitidis	B	B	B	4	2	6	84	2	3	6	9	5	9	6	9	ST-41/44 complex	41	2	3	1
29765	PM65NS	UK	2000	invasive (unspecified/other)	Neisseria meningitidis	NG	B	B	4	2	6	84	2	3	6	9	5	9	6	9	ST-41/44 complex	41	2	3	1
29766	PM65TS	UK	2000	invasive (unspecified/other)	Neisseria meningitidis	B	B	B	4	2	6	84	2	3	6	9	5	9	6	9	ST-41/44 complex	41	2	3	1
29769	PM66B	UK	2000	invasive (unspecified/other)	Neisseria meningitidis	B	B	B	14	7	1	142	6	3	6	9	5	9	6	9	ST-41/44 complex	41	6	2	1
29767	PM66NS	UK	2000	invasive (unspecified/other)	Neisseria meningitidis	B	B	B	14	7	1	142	6	3	6	9	5	9	6	9	ST-41/44 complex	41	6	2	1
29768	PM66TS	UK	2000	invasive (unspecified/other)	Neisseria meningitidis	B	B	B	14	7	1	142	6	3	6	9	5	9	6	9	ST-41/44 complex	41	6	2	1
29773	PM67B	UK	2000	invasive (unspecified/other)	Neisseria meningitidis	B	B	B	354	165	5	41	169	3	6	9	5	9	6	9	ST-41/44 complex	41	NA	NA	3
29774	PM67CSF	UK	2000	invasive (unspecified/other)	Neisseria meningitidis	B	B	B	354	165	5	41	169	3	6	9	5	9	6	9	ST-41/44 complex	41	NA	NA	3
29801	PM68B	UK	2003	invasive (unspecified/other)	Neisseria meningitidis	B	B	B	4	2	1	84	2	3	6	9	5	9	6	9	ST-41/44 complex	41	2	3	1
29800	PM68CSF1	UK	2003	invasive (unspecified/other)	Neisseria meningitidis	B	B	B	4	2	1	84	2	3	6	9	5	9	6	9	ST-41/44 complex	41	2	3	1
29802	PM68CSF2	UK	2003	invasive (unspecified/other)	Neisseria meningitidis	B	B	B	4	2	1	84	2	3	6	9	5	9	6	9	ST-41/44 complex	41	2	3	1
29824	PM69B	UK	1998	invasive (unspecified/other)	Neisseria meningitidis	B	B	B	4	2	1	84	2	3	6	9	5	9	6	9	ST-41/44 complex	41	2	3	1
29823	PM69CSF	UK	1998	invasive (unspecified/other)	Neisseria meningitidis	B	B	B	4	2	1	84	2	3	6	9	5	9	6	9	ST-41/44 complex	41	2	3	1
29825	PM69TS	UK	1998	invasive (unspecified/other)	Neisseria meningitidis	B	B	B	4	2	1	84	2	3	6	9	5	9	6	9	ST-41/44 complex	41	2	3	1
30115	PM70B	UK	1999	invasive (unspecified/other)	Neisseria meningitidis	B	B	B	14	7	1	142	6	3	6	9	5	9	6	9	ST-41/44 complex	41	6	2	1
30116	PM70CSF	UK	1999	invasive (unspecified/other)	Neisseria meningitidis	B	B	B	14	7	1	142	6	3	6	9	5	9	6	9	ST-41/44 complex	41	6	2	1
30117	PM70TS	UK	1999	invasive (unspecified/other)	Neisseria meningitidis	B	B	B	14	7	1	142	6	3	6	9	5	9	6	9	ST-41/44 complex	41	6	2	1
20887	PM71B	UK	2001	invasive (unspecified/other)	Neisseria meningitidis	B	B	B	14	7	1	142	6	10	6	9	5	9	6	9	ST-41/44 complex	42	6	2	1
20915	PM72B	UK	2001	invasive (unspecified/other)	Neisseria meningitidis	B	B	B	14	7	1	142	6	10	6	9	5	9	6	9	ST-41/44 complex	42	6	2	1
29745	PM73B	UK	1998	invasive (unspecified/other)	Neisseria meningitidis	B	B	B	14	7	1	142	6	10	6	9	5	9	6	9	ST-41/44 complex	42	6	2	1
29744	PM73CSF	UK	1998	invasive (unspecified/other)	Neisseria meningitidis	B	B	B	14	7	1	142	6	10	6	9	5	9	6	9	ST-41/44 complex	42	6	2	1
29746	PM73TS	UK	1998	invasive (unspecified/other)	Neisseria meningitidis	B	B	B	14	7	1	142	6	10	6	9	5	9	6	9	ST-41/44 complex	42	6	2	1
29771	PM74B	UK	2000	invasive (unspecified/other)	Neisseria meningitidis	B	B	B	14	7	1	257	6	10	6	9	5	9	6	9	ST-41/44 complex	42	6	2	1
29770	PM74CSF	UK	2000	invasive (unspecified/other)	Neisseria meningitidis	B	B	B	14	7	1	257	6	10	6	9	5	9	6	9	ST-41/44 complex	42	6	2	1
29772	PM74TS	UK	2000	invasive (unspecified/other)	Neisseria meningitidis	B	B	B	14	7	1	257	6	10	6	9	5	9	6	9	ST-41/44 complex	42	6	2	1
20944	PM75B	UK	2005	invasive (unspecified/other)	Neisseria meningitidis	B	B	B	4	2	1	84	2	3	6	9	5	3	6	9	ST-41/44 complex	46	2	3	1
20884	PM76B	UK	2000	invasive (unspecified/other)	Neisseria meningitidis	B	B	B	4	2	1	84	2	3	6	9	5	11	6	9	ST-41/44 complex	154	2	3	1
20941	PM78B	UK	2005	invasive (unspecified/other)	Neisseria meningitidis	B	B	B	19	1	1	65	4	3	6	9	24	9	38	9	ST-41/44 complex	1414	4	4	2
29758	PM79B	UK	1998	invasive (unspecified/other)	Neisseria meningitidis	B	B	B	24	1	63	25	4	27	6	9	17	9	6	16	ST-41/44 complex	1770	4	4	2
29757	PM79CSF	UK	1998	invasive (unspecified/other)	Neisseria meningitidis	B	B	B	24	1	63	25	4	27	6	9	17	9	6	16	ST-41/44 complex	1770	4	4	2
29759	PM79TS	UK	1998	invasive (unspecified/other)	Neisseria meningitidis	B	B	B	24	1	63	25	4	27	6	9	17	9	6	16	ST-41/44 complex	1770	4	4	2
29738	PM7B	UK	1997	invasive (unspecified/other)	Neisseria meningitidis	C	C	C	771	5	1	322	11	2	3	4	3	8	4	6	ST-11 complex	11	11	2	1
29739	PM7CSF	UK	1997	invasive (unspecified/other)	Neisseria meningitidis	C	C	C	771	5	1	322	11	2	3	4	3	8	4	6	ST-11 complex	11	11	2	1
29740	PM7TS	UK	1997	invasive (unspecified/other)	Neisseria meningitidis	C	C	C	771	5	1	322	11	2	3	4	3	8	4	6	ST-11 complex	11	11	2	1
30114	PM80B	UK	1999	invasive (unspecified/other)	Neisseria meningitidis	B	B	B	4	2	1	84	2	3	6	9	NA	9	18	9		NA	2	3	1
30112	PM80NS	UK	1999	invasive (unspecified/other)	Neisseria meningitidis	B	B	B	4	2	1	84	2	3	6	9	5	9	18	9	ST-41/44 complex	2080	2	3	1
30113	PM80TS	UK	1999	invasive (unspecified/other)	Neisseria meningitidis	B	B	B	4	2	1	84	2	3	6	9	5	9	18	9	ST-41/44 complex	2080	2	3	1
20965	PM81B	UK	2007	invasive (unspecified/other)	Neisseria meningitidis	B	B	B	4	2	1	84	2	3	6	9	25	9	6	9	ST-41/44 complex	2799	2	3	1
29763	PM82B	UK	2000	invasive (unspecified/other)	Neisseria meningitidis	B	B	B	4	2	1	84	2	306	6	9	5	9	6	9	ST-41/44 complex	11162	2	3	1
30120	PM82CSF	UK	1999	invasive (unspecified/other)	Neisseria meningitidis	B	B	B	4	2	1	84	2	306	6	9	5	9	6	9	ST-41/44 complex	11162	2	3	1
30121	PM82NS	UK	1999	invasive (unspecified/other)	Neisseria meningitidis	B	B	B	4	2	1	84	2	306	6	9	5	9	6	9	ST-41/44 complex	11162	2	3	1
29753	PM85B	UK	1998	invasive (unspecified/other)	Neisseria meningitidis	B	B	B	13	3	1	145	3	17	5	19	17	3	26	2	ST-60 complex	60	3	3	1
29754	PM85NS	UK	1998	invasive (unspecified/other)	Neisseria meningitidis	B	B	B	13	3	1	145	3	17	5	19	17	3	26	2	ST-60 complex	60	3	3	1
29755	PM85TS	UK	1998	invasive (unspecified/other)	Neisseria meningitidis	B	B	B	13	3	1	145	3	17	5	19	17	3	26	2	ST-60 complex	60	3	3	1
20979	PM86B	UK	1998	invasive (unspecified/other)	Neisseria meningitidis		B	B	16	1	4	59	4	2	3	7	2	34	5	2	ST-8 complex	153	4	4	2
29829	PM87CSF	UK	1998	invasive (unspecified/other)	Neisseria meningitidis	B	B	B	22	1	1	1	4	2	3	7	2	34	5	2	ST-8 complex	153	4	4	2
29830	PM87TS1	UK	1999	invasive (unspecified/other)	Neisseria meningitidis	B	B	B	22	1	1	1	4	2	3	7	2	34	5	2	ST-8 complex	153	4	4	2
30108	PM87TS2	UK	1999	invasive (unspecified/other)	Neisseria meningitidis	B	B	B	22	1	1	1	4	2	3	7	2	34	5	2	ST-8 complex	153	4	4	2
29750	PM8CSF	UK	1998	invasive (unspecified/other)	Neisseria meningitidis	C	C	C	776	5	1	NA	11	2	3	4	3	8	4	6	ST-11 complex	11	11	2	NA
29751	PM8NS	UK	1998	invasive (unspecified/other)	Neisseria meningitidis	C	C	C	776	5	1	NA	11	2	3	4	3	8	4	6	ST-11 complex	11	11	2	NA
29752	PM8TS	UK	1998	invasive (unspecified/other)	Neisseria meningitidis	C	C	C	776	5	1	NA	11	2	3	4	3	8	4	6	ST-11 complex	11	11	2	NA
29778	PM9CSF	UK	2000	invasive (unspecified/other)	Neisseria meningitidis	W	W	W	9	1	1	187	47	2	3	4	3	8	4	6	ST-11 complex	11	NA	NA	1
29776	PM9TS	UK	2000	invasive (unspecified/other)	Neisseria meningitidis	W	W	W	9	1	1	187	47	2	3	4	3	8	4	6	ST-11 complex	11	NA	NA	1
49978	PR40009	UK [England]	2015	carrier	Neisseria meningitidis	B	B	B	19	1	1	83	4	4	10	2	5	38	11	9	ST-269 complex	275	4	4	2
52669	PR40013	UK [England]	2015	carrier	Neisseria meningitidis	B	B	B	19	1	2	65	4	6	6	63	9	9	11	9	ST-41/44 complex	839	4	4	2
49980	PR40017	UK [England]	2015	carrier	Neisseria meningitidis	NG	E	E	13	3	57	209	3	8	25	7	17	21	26	49	ST-1157 complex	1157	3	3	1
49981	PR40019	UK [England]	2015	carrier	Neisseria meningitidis	NG	E	E	12	258	57	266	NA	7	16	55	10	3	56	46	ST-178 complex	178	NA	NA	1
49982	PR40021	UK [England]	2015	carrier	Neisseria meningitidis	NG	E	E	13	3	57	209	3	8	25	7	17	21	26	49	ST-1157 complex	1157	3	3	1
52670	PR40026	UK [England]	2015	carrier	Neisseria meningitidis	B	B	B	45	8	77	87	20	7	5	1	13	36	53	15	ST-213 complex	213	20	4	3
52671	PR40027	UK [England]	2015	carrier	Neisseria meningitidis	B	B	B	14	7	1	142	6	3	6	9	5	9	6	9	ST-41/44 complex	41	6	2	1
50316	PR40050	UK [England]	2015	carrier	Neisseria meningitidis	Y	Y	Y	25	1	1	26	4	10	5	18	9	11	9	17	ST-23 complex	23	4	4	2
50317	PR40056	UK [England]	2015	carrier	Neisseria meningitidis	NG	NG	NG	24	1	4	27	4	4	10	72	17	6	10	12	ST-35 complex	12401	4	4	2
49983	PR40072	UK [England]	2015	carrier	Neisseria meningitidis	Y	Y	Y	25	1	1	26	4	12	5	18	9	11	9	17	ST-23 complex	1655	4	4	2
49984	PR40077	UK [England]	2015	carrier	Neisseria meningitidis	NG	B	B	19	1	1	65	4	9	6	9	9	58	6	847	ST-41/44 complex	12849	4	4	2
49985	PR40083	UK [England]	2015	carrier	Neisseria meningitidis	B	B	B	47	1	57	90	15	12	5	12	35	60	22	17	ST-461 complex	461	15	4	3
49986	PR40090	UK [England]	2015	carrier	Neisseria meningitidis	W	W/Y	discrepancy	25	1	1	26	4	12	5	18	9	11	9	17	ST-23 complex	1655	4	4	2
49987	PR40108	UK [England]	2015	carrier	Neisseria meningitidis	B	B	B	19	1	1	83	4	4	10	2	5	38	11	9	ST-269 complex	275	4	4	2
49988	PR40109	UK [England]	2015	carrier	Neisseria meningitidis	NG	C	C	24	1	2	25	4	49	2	270	8	13	21	53	ST-231 complex	12681	4	4	2
49989	PR40113	UK [England]	2015	carrier	Neisseria meningitidis	NG	cnl	cnl	102	8	10	2	20	16	2	6	25	17	25	22	ST-53 complex	53	20	4	2
49990	PR40117	UK [England]	2015	carrier	Neisseria meningitidis	NG	Z	Z	16	56	1	60	NA	13	29	2	17	26	65	53		2123	NA	NA	2
52673	PR40118	UK [England]	2015	carrier	Neisseria meningitidis	NG	Z	Z	208	1	1	701	4	46	29	186	176	67	175	96		1975	4	3	1
49992	PR40141	UK [England]	2015	carrier	Neisseria meningitidis	NG	L	L	13	3	57	145	3	57	5	9	35	6	68	20		6068	3	3	1
49993	PR40142	UK [England]	2015	carrier	Neisseria meningitidis	B	B	B	24	1	4	25	4	4	5	47	17	6	2	12		12682	4	4	2
49994	PR40149	UK [England]	2015	carrier	Neisseria meningitidis	B	B	B	24	1	4	25	4	4	10	47	17	6	2	12	ST-35 complex	278	4	4	2
49995	PR40167	UK [England]	2015	carrier	Neisseria meningitidis	NG	Y	Y	23	1	1	4	4	2	7	6	17	1	18	8	ST-167 complex	1627	4	4	2
49996	PR40174	UK [England]	2015	carrier	Neisseria meningitidis	B	B	B	23	1	1	481	4	7	5	1	13	82	53	15	ST-213 complex	575	4	4	2
49997	PR40189	UK [England]	2015	carrier	Neisseria meningitidis	Y	Y	Y	25	1	1	26	4	12	5	1	9	11	9	17	ST-23 complex	11388	4	4	2
49998	PR40193	UK [England]	2015	carrier	Neisseria meningitidis	Y	Y	Y	25	1	1	26	4	10	5	18	9	11	9	17	ST-23 complex	23	4	4	2
49999	PR40206	UK [England]	2015	carrier	Neisseria meningitidis	NG	E	E	13	3	57	209	3	8	25	7	17	21	26	49	ST-1157 complex	1157	3	3	1
50000	PR40207	UK [England]	2015	carrier	Neisseria meningitidis	Y	Y	Y	24	1	1	27	4	12	7	6	17	9	6	8	ST-167 complex	10730	4	4	2
50001	PR40209	UK [England]	2015	carrier	Neisseria meningitidis	NG	X	X	13	3	57	209	3	8	25	7	17	21	26	49	ST-1157 complex	1157	3	3	1
50002	PR40214	UK [England]	2015	carrier	Neisseria meningitidis	NG	C	C	16	1	4	59	4	132	3	19	17	62	21	2		1434	4	4	2
50003	PR40237	UK [England]	2015	carrier	Neisseria meningitidis	NG	cnl	cnl	94	NA	56	69	NA	5	4	17	15	14	7	12	ST-198 complex	198	NA	NA	3
50004	PR40239	UK [England]	2015	carrier	Neisseria meningitidis	B	B	B	13	3	57	145	3	4	10	34	5	38	11	9	ST-269 complex	1161	3	3	1
50005	PR40250	UK [England]	2015	carrier	Neisseria meningitidis	NG	B	B	4	2	1	84	2	3	6	9	5	8	6	9	ST-41/44 complex	485	2	3	1
50006	PR40289	UK [England]	2015	carrier	Neisseria meningitidis	NG	cnl	cnl	334	1	1	527	4	6	41	108	15	9	6	9		845	4	4	2
50007	PR40293	UK [England]	2015	carrier	Neisseria meningitidis	NG	cnl	cnl	102	8	10	2	20	16	2	6	25	17	25	22	ST-53 complex	53	20	4	2
50008	PR40294	UK [England]	2015	carrier	Neisseria meningitidis	NG	E	E	13	3	1	145	3	17	5	19	17	62	26	2	ST-60 complex	10454	3	3	1
50009	PR40299	UK [England]	2015	carrier	Neisseria meningitidis	B	B	B	19	1	1	83	4	4	10	861	5	38	11	9	ST-269 complex	12850	4	4	2
50010	PR40301	UK [England]	2015	carrier	Neisseria meningitidis	NG	Z	Z	0	56	1	923	NA	13	29	2	17	26	65	53		2123	NA	NA	NA
50011	PR40307	UK [England]	2015	carrier	Neisseria meningitidis	B	B	B	13	3	57	145	3	175	5	484	17	3	4	124		6798	3	3	1
50012	PR40312	UK [England]	2015	carrier	Neisseria meningitidis	NG	B	B	848	1	57	900	13	4	10	5	4	6	3	8	ST-32 complex	32	13	3	1
50013	PR40316	UK [England]	2015	carrier	Neisseria meningitidis	B	B	B	21	1	1	7	4	1	5	13	53	26	41	3	ST-162 complex	162	4	4	2

50014	PR40317	UK [England]	2015	carrier	Neisseria meningitidis	NG	NG	NG	104	1	57	10	4	821	5	18	59	11	9	17	ST-23 complex	12851	4	4	2
50015	PR40328	UK [England]	2015	carrier	Neisseria meningitidis	NG	E	E	13	3	57	209	3	8	25	7	17	21	26	49	ST-1157 complex	1157	3	3	1
50016	PR40332	UK [England]	2015	carrier	Neisseria meningitidis	B	B	B	21	1	1	7	4	1	5	13	53	26	41	3	ST-162 complex	162	4	4	2
50017	PR40344	UK [England]	2015	carrier	Neisseria meningitidis	NG	E	E	13	3	1	145	3	17	5	19	17	3	26	2	ST-60 complex	60	3	3	1
50018	PR40365	UK [England]	2015	carrier	Neisseria meningitidis	B	B	B	47	1	57	90	15	12	5	862	35	192	22	17	ST-461 complex	12852	15	4	3
50019	PR40374	UK [England]	2015	carrier	Neisseria meningitidis	NG	cnl	cnl	4	1	1	206	4	5	4	17	15	30	7	12	ST-198 complex	823	4	3	1
50020	PR40377	UK [England]	2015	carrier	Neisseria meningitidis	NG	E	E	13	3	1	145	3	17	5	19	17	62	26	2	ST-60 complex	10454	3	3	1
50021	PR40379	UK [England]	2015	carrier	Neisseria meningitidis	NG	E	E	13	3	57	209	3	8	25	7	17	21	26	49	ST-1157 complex	1157	3	3	1
50022	PR40385	UK [England]	2015	carrier	Neisseria meningitidis	NG	cnl	cnl	21	1	1	7	4	16	2	159	92	77	25	112	ST-1117 complex	1117	4	4	2
50023	PR40391	UK [England]	2015	carrier	Neisseria meningitidis	B	B	B	23	1	1	898	4	7	5	1	13	36	53	15	ST-213 complex	213	4	4	2
50024	PR40392	UK [England]	2015	carrier	Neisseria meningitidis	B	B	B	45	8	77	87	20	7	5	1	13	36	53	15	ST-213 complex	213	20	4	3
50025	PR40396	UK [England]	2015	carrier	Neisseria meningitidis	NG	B	B	45	8	77	87	20	7	596	1	13	36	53	15	ST-213 complex	12853	20	4	3
50026	PR40412	UK [England]	2015	carrier	Neisseria meningitidis	NG	cnl	cnl	1027	8	10	901	20	7	5	1	13	36	53	15	ST-213 complex	213	20	4	2
50027	PR40415	UK [England]	2015	carrier	Neisseria meningitidis	NG	cnl	cnl	102	8	10	2	20	16	2	6	25	17	25	22	ST-53 complex	53	20	4	2
50028	PR40427	UK [England]	2015	carrier	Neisseria meningitidis	NG	B	B	21	1	1	7	4	1	5	13	53	26	41	3	ST-162 complex	162	4	4	2
50029	PR40439	UK [England]	2015	carrier	Neisseria meningitidis	NG	E	E	4	2	1	84	2	8	25	7	17	21	26	49	ST-1157 complex	1157	2	3	1
50030	PR40453	UK [England]	2015	carrier	Neisseria meningitidis	NG	cnl	cnl	102	8	10	2	20	16	2	18	25	17	25	22	ST-53 complex	2441	20	4	2
50031	PR40454	UK [England]	2015	carrier	Neisseria meningitidis	NG	B	B	16	1	4	59	4	4	10	136	17	5	10	12	ST-35 complex	815	4	4	2
50033	PR40459	UK [England]	2015	carrier	Neisseria meningitidis	NG	E	E	13	3	1	145	3	17	5	19	17	62	26	2	ST-60 complex	10454	3	3	1
50034	PR40471	UK [England]	2015	carrier	Neisseria meningitidis	NG	B	B	23	1	1	898	4	7	5	1	13	36	53	15	ST-213 complex	213	4	4	2
50035	PR40474	UK [England]	2015	carrier	Neisseria meningitidis	NG	cnl	cnl	21	1	1	7	4	16	2	159	92	77	25	112	ST-1117 complex	1117	4	4	2
50036	PR40479	UK [England]	2015	carrier	Neisseria meningitidis	NG	B	B	15	4	2	193	1	4	10	15	9	8	11	9	ST-269 complex	269	1	1	1
50037	PR40483	UK [England]	2015	carrier	Neisseria meningitidis	NG	B	B	45	61	77	87	NA	7	5	1	17	36	53	15	ST-213 complex	2391	NA	NA	3
52674	PR40486	UK [England]	2015	carrier	Neisseria meningitidis	NG	B	B	24	1	4	27	4	4	10	72	9	6	10	12	ST-35 complex	2380	4	4	2
50039	PR40487	UK [England]	2015	carrier	Neisseria meningitidis	Y	Y	Y	23	1	1	4	4	2	7	6	17	1	18	8	ST-167 complex	1627	4	4	2
50040	PR40493	UK [England]	2015	carrier	Neisseria meningitidis	NG	E	E	13	3	57	209	3	8	25	7	17	21	26	49	ST-1157 complex	1157	3	3	1
50041	PR40494	UK [England]	2015	carrier	Neisseria meningitidis	B	B	B	16	1	1	60	4	27	7	15	3	9	6	16		12683	4	4	2
50042	PR40498	UK [England]	2015	carrier	Neisseria meningitidis	NG	B	B	0	6	3	922	7	4	10	5	4	6	3	847	ST-32 complex	12684	7	1	NA
50043	PR40499	UK [England]	2015	carrier	Neisseria meningitidis	Y	Y	Y	25	1	1	26	4	12	5	18	9	11	9	17	ST-23 complex	1655	4	4	2
50045	PR40506	UK [England]	2015	carrier	Neisseria meningitidis	NG	cnl	cnl	21	1	1	7	4	16	2	159	92	77	25	112	ST-1117 complex	1117	4	4	2
50046	PR40518	UK [England]	2015	carrier	Neisseria meningitidis	NG	Z	Z	25	1	1	26	4	8	4	6	17	5	18	2	ST-103 complex	103	4	4	2
50047	PR40522	UK [England]	2015	carrier	Neisseria meningitidis	NG	B	B	24	1	63	25	4	27	6	9	9	9	6	16	ST-41/44 complex	1213	4	4	2
50048	PR40535	UK [England]	2015	carrier	Neisseria meningitidis	NG	B	B	16	1	4	59	4	4	10	136	17	5	10	12	ST-35 complex	815	4	4	2
50049	PR40545	UK [England]	2015	carrier	Neisseria meningitidis	NG	Z	Z	16	1	1	60	4	8	4	6	17	5	18	2	ST-103 complex	103	4	4	2
50050	PR40568	UK [England]	2015	carrier	Neisseria meningitidis	NG	Z	Z	16	56	1	60	NA	13	29	2	17	26	65	53		2123	NA	NA	2
50051	PR40570	UK [England]	2015	carrier	Neisseria meningitidis	B	B	B	45	8	77	87	20	7	5	1	13	36	53	15	ST-213 complex	213	20	4	3
50052	PR40573	UK [England]	2015	carrier	Neisseria meningitidis	NG	E	E	13	3	1	145	3	17	5	19	17	62	26	2	ST-60 complex	10454	3	3	1
50032	PR40574	UK [England]	2015	carrier	Neisseria meningitidis	NG	E	E	13	3	57	209	3	8	25	7	17	21	26	49	ST-1157 complex	1157	3	3	1
50054	PR40594	UK [England]	2015	carrier	Neisseria meningitidis	B	B	B	24	1	63	25	4	27	6	9	3	9	6	16	ST-41/44 complex	136	4	4	2
50055	PR40634	UK [England]	2015	carrier	Neisseria meningitidis	Y	Y	Y	25	1	1	26	4	12	5	18	9	11	9	17	ST-23 complex	1655	4	4	2
50056	PR40636	UK [England]	2015	carrier	Neisseria meningitidis	NG	cnl	cnl	94	58	56	89	NA	5	4	38	15	22	40	13	ST-1136 complex	1136	NA	NA	3
50057	PR40648	UK [England]	2015	carrier	Neisseria meningitidis	B	B	B	21	1	1	7	4	4	10	5	26	6	3	8	ST-32 complex	1096	4	4	2
50941	PR40657	UK [England]	2015	carrier	Neisseria meningitidis	NG	E	E	13	3	57	209	3	8	25	7	17	21	26	49	ST-1157 complex	1157	3	3	1
50058	PR40664	UK [England]	2015	carrier	Neisseria meningitidis	B	B	B	45	8	77	87	20	7	5	1	13	36	53	15	ST-213 complex	213	20	4	3
50059	PR40673	UK [England]	2015	carrier	Neisseria meningitidis	NG	X	X	16	1	56	60	4	807	5	781	7	818	2	187		12686	4	4	2
50060	PR40701	UK [England]	2015	carrier	Neisseria meningitidis	B	B	B	13	3	57	145	3	4	10	2	5	38	11	15		2776	3	3	1
50061	PR40702	UK [England]	2015	carrier	Neisseria meningitidis	Y	Y	Y	25	1	1	26	4	12	5	18	9	11	9	17	ST-23 complex	1655	4	4	2
50062	PR40709	UK [England]	2015	carrier	Neisseria meningitidis	NG	cnl	cnl	985	8	10	787	20	16	2	6	25	17	25	22	ST-53 complex	53	20	4	2
50063	PR40736	UK [England]	2015	carrier	Neisseria meningitidis	NG	B	B	13	3	77	145	3	7	5	1	13	36	53	15	ST-213 complex	213	3	3	1
50064	PR40756	UK [England]	2015	carrier	Neisseria meningitidis	NG	cnl	cnl	14	7	1	142	6	3	6	9	5	9	6	9	ST-41/44 complex	41	6	2	1
50065	PR40761	UK [England]	2015	carrier	Neisseria meningitidis	NG	cnl	cnl	102	8	10	2	20	16	2	6	25	17	25	22	ST-53 complex	53	20	4	2
50066	PR40765	UK [England]	2015	carrier	Neisseria meningitidis	NG	Y	Y	25	1	1	26	4	12	5	18	9	11	9	17	ST-23 complex	1655	4	4	2
50067	PR40773	UK [England]	2015	carrier	Neisseria meningitidis	NG	E	E	991	66	2	794	38	23	5	9	1	26	32	123		12687	NA	NA	1
50068	PR40805	UK [England]	2015	carrier	Neisseria meningitidis	B	B	B	13	3	57	145	3	4	10	2	5	38	11	15		2776	3	3	1
50069	PR40820	UK [England]	2015	carrier	Neisseria meningitidis	Y	Y	Y	25	1	1	26	4	10	5	18	9	11	9	17	ST-23 complex	23	4	4	2
50070	PR40840	UK [England]	2015	carrier	Neisseria meningitidis	NG	cnl	cnl	94	157	56	69	NA	5	4	17	15	14	7	12	ST-198 complex	198	NA	NA	3
50071	PR40870	UK [England]	2015	carrier	Neisseria meningitidis	Y	Y	Y	25	1	1	26	4	10	5	18	9	11	9	17	ST-23 complex	23	4	4	2
50072	PR40875	UK [England]	2015	carrier	Neisseria meningitidis	Y	Y	Y	25	1	1	26	4	12	5	18	9	11	9	17	ST-23 complex	1655	4	4	2
52675	PR40877	UK [England]	2015	carrier	Neisseria meningitidis	NG	B	B	23	250	1	431	NA	8	5	15	17	8	21	2	ST-865 complex	865	NA	NA	2
50074	PR40882	UK [England]	2015	carrier	Neisseria meningitidis	Y	Y	Y	25	1	1	26	4	10	5	18	9	11	9	17	ST-23 complex	23	4	4	2
50075	PR40893	UK [England]	2015	carrier	Neisseria meningitidis	B	B	B	22	1	1	1	4	7	2	1	13	8	53	6	ST-213 complex	12688	4	4	2
50076	PR40907	UK [England]	2015	carrier	Neisseria meningitidis	NG	E	E	13	3	1	145	3	3	585	19	5	3	26	2	ST-60 complex	12689	3	3	1
50077	PR40922	UK [England]	2015	carrier	Neisseria meningitidis	NG	Y	Y	23	1	1	4	4	2	7	159	92	93	6	8		6464	4	4	2
50078	PR40929	UK [England]	2015	carrier	Neisseria meningitidis	NG	Y	Y	25	1	1	26	4	10	5	18	9	11	9	17	ST-23 complex	23	4	4	2
50079	PR40939	UK [England]	2015	carrier	Neisseria meningitidis	NG	E	E	13	3	57	209	3	8	25	7	17	21	26	49	ST-1157 complex	1157	3	3	1
50080	PR40941	UK [England]	2015	carrier	Neisseria meningitidis	W	W	W	16	1	1	60	4	11	5	168	8	11	4	21	ST-22 complex	1286	4	4	2
50081	PR40945	UK [England]	2015	carrier	Neisseria meningitidis	NG	B	B	49	1	57	102	13	4	10	5	4	6	3	8	ST-32 complex	32	13	4	2
50082	PR40977	UK [England]	2015	carrier	Neisseria meningitidis	NG	NG	NG	151	1	1	130	4	6	7	4	56	26	18	8	ST-175 complex	175	4	4	2
50083	PR40987	UK [England]	2015	carrier	Neisseria meningitidis	NG	cnl	cnl	102	8	10	2	20	16	2	6	25	17	25	22	ST-53 complex	53	20	4	2
50084	PR40990	UK [England]	2015	carrier	Neisseria meningitidis	NG	cnl	cnl	102	8	10	2	20	16	2	6	25	17	25	22	ST-53 complex	53	20	4	2
51172	R001	UK [England]	2015	carrier	Neisseria meningitidis		W	W	22	1	1	1	4	2	3	4	3	8	4	6	ST-11 complex	11	4	4	2
51313	R011	UK [England]	2015	carrier	Neisseria meningitidis		Y	Y	25	1	1	26	4	12	5	18	332	77	9	17	ST-23 complex	12176	4	4	2
51314	R023	UK [England]	2015	carrier	Neisseria meningitidis		cnl	cnl	102	8	10	2	20	16	2	6	25	17	25	22	ST-53 complex	53	20	4	2
51316	R035	UK [England]	2015	carrier	Neisseria meningitidis		cnl	cnl	102	8	1	2	20	16	2	6	25	17	25	911	ST-53 complex	13443	20	4	2
51317	R048	UK [England]	2015	carrier	Neisseria meningitidis		cnl	cnl	4	1	1	206	4	5	4	17	15	30	7	12	ST-198 complex	823	4	3	1
51318	R056	UK [England]	2015	carrier	Neisseria meningitidis		cnl	cnl	94	58	56	89	NA	5	4	38	15	22	40	13	ST-1136 complex	1136	NA	NA	3
51319	R062	UK [England]	2015	carrier	Neisseria meningitidis		Y	Y	25	1	1	26	4	12	5	18	9	11	9	17	ST-23 complex	1655	4	4	2
51324	R109	UK [England]	2015	carrier	Neisseria meningitidis		Y	Y	12	8	57	899	20	8	25	7	17	21	26	49	ST-1157 complex	1157	20	3	1
51326	R135	UK [England]	2015	carrier	Neisseria meningitidis		B	B	23	250	1	431	NA	8	5	15	17	8	21	2	ST-865 complex	865	NA	NA	2
51327	R136	UK [England]	2015	carrier	Neisseria meningitidis		cnl	cnl	4	1	1	206	4	5	4	17	15	30	7	12	ST-198 complex	823	4	3	1
51328	R156	UK [England]	2015	carrier	Neisseria meningitidis		cnl	cnl	94	58	56	89	NA	NA	4	38	15	22	40	13		NA	NA	NA	3
51329	R164	UK [England]	2015	carrier	Neisseria meningitidis		cnl	cnl	21	1	1	7	4	16	2	159	92	77	25	112	ST-1117 complex	1117	4	4	2
51331	R172	UK [England]	2015	carrier	Neisseria meningitidis		NG	NG	15	4	2	193	1	4	10	15	9	8	11	9	ST-269 complex	269	1	1	1
51332	R174	UK [England]	2015	carrier	Neisseria meningitidis		cnl	cnl	21	1	1	7	4	16	2	159	92	77	25	112	ST-1117 complex	1117	4	4	2
51333	R178	UK [England]	2015	carrier	Neisseria meningitidis		NG	NG	45	8	77	87	20	7	5	1	13	36	53	15	ST-213 complex	213	20	4	3
51173	R191	UK [England]	2015	carrier	Neisseria meningitidis		W	W	22	1	1	1	4	2	3	4	3	8	4	6	ST-11 complex	11	4	4	2
51335	R192	UK [England]	2015	carrier	Neisseria meningitidis		cnl	cnl	4	1	1	206	4	5	4	17	15	30	7	12	ST-198 complex	823	4	3	1
51337	R199	UK [England]	2015	carrier	Neisseria meningitidis		cnl	cnl	4	1	1	206	4	5	4	17	15	30	7	12	ST-198 complex	823	4	3	1
51174	R258	UK [England]	2015	carrier	Neisseria meningitidis		W	W	22	1	1	1	4	2	3	4	3	8	4	6	ST-11 complex	11	4	4	2
51343	R265	UK [England]	2015	carrier	Neisseria meningitidis		cnl	cnl	102	132	10	2	NA	16	2	6	25	17	25	22	ST-53 complex	53	NA	NA	2
51348	R286	UK [England]	2015	carrier	Neisseria meningitidis		Y	Y	25	1	1	26	4	12	5	18	9	11	9	17	ST-23 complex	1655	4	4	2
51350	R297	UK [England]	2015	carrier	Neisseria meningitidis		Y	Y	25	1	1	26	4	12	5	18	9	11	9	17	ST-23 complex	1655	4	4	2
51351	R300	UK [England]	2015	carrier	Neisseria meningitidis		Y	Y	25	1	1	26	4	12	5	18	9	11	9	17	ST-23 complex	1655	4	4	2
51358	R346	UK [England]	2015	carrier	Neisseria meningitidis		Y	Y	25	1	1	26	4	12	5	18	9	11	9	17	ST-23 complex	1655	4	4	2
51362	R383	UK [England]	2015	carrier	Neisseria meningitidis		Y	Y	25	1	1	26	4	12	5	18	9	11	9	17	ST-23 complex	1655	4	4	2
51365	R424	UK [England]	2015	carrier	Neisseria meningitidis		Y	Y	104	1	57	10	4	10	5	18	59	11	9	17	ST-23 complex	183	4	4	2
51368	R477	UK [England]	2015	carrier	Neisseria meningitidis		Y	Y	25	1	1	26	4	12	5	18	9	11	9	17	ST-23 complex	1655	4	4	2
51369	R488	UK [England]	2015	carrier	Neisseria meningitidis		NG	NG	24	1	4	27	4	4	10	72	9	6	10	12	ST-35 complex	2380	4	4	2
51371	R504	UK [England]	2015	carrier	Neisseria meningitidis		cnl	cnl	4	1	1	206	4	5	4	17	15	30	7	12	ST-198 complex	823	4	3	1
51373	R513	UK [England]	2015	carrier	Neisseria meningitidis		cnl	cnl	102	8	10	2	20	855	2	6	25	17	25	22	ST-53 complex	13398	20	4	2
51374	R531	UK [England]	2015	carrier	Neisseria meningitidis		cnl	cnl	627	1	1	892	4	217	3	79	7	260	249	123		7129	4	4	3
51375	R534	UK [England]	2015	carrier	Neisseria meningitidis		Y	Y	25	1	1	26	4	10	5	18	9	11	9	17	ST-23 complex	23	4	4	2
51376	R542	UK [England]	2015	carrier	Neisseria meningitidis		cnl	cnl	21	1	57	7	4	8	25	7	17	21	26	49	ST-1157 complex	1157	4	4	2
51176	R558	UK [England]	2015	carrier	Neisseria meningitidis		W	W	22	1	1	1	4	662	3	4	3	8	4	6	ST-11 complex	10651	4	4	2
51379	R570	UK [England]	2015	carrier	Neisseria meningitidis		NG	NG	16	1	4	59	4	4	10	11	17	6	10	12	ST-35 complex	457	4	4	2
51380	R575	UK [England]	2015	carrier	Neisseria meningitidis		W	W	22	1	1	1	4	2	3	4	3	8	4	6	ST-11 complex	11	4	4	2
51382	R581	UK [England]	2015	carrier	Neisseria meningitidis		cnl	cnl	102	8	10	2	20	852	2	6	25	17	25	22	ST-53 complex	13397	20	4	2
51385	R589	UK [England]	2015	carrier	Neisseria meningitidis		cnl	cnl	21	1	1	7	4	16	2	159	92	77	25	112	ST-1117 complex	1117	4	4	2
51386	R598	UK [England]	2015	carrier	Neisseria meningitidis		B	B	456	73	80	243	56	8	5	15	17	8	21	2	ST-865 complex	865	NA	NA	1
51389	R621	UK [England]	2015	carrier	Neisseria meningitidis		cnl	cnl	4	1	1	206	4	5	4	17	15	30	7	12	ST-198 complex	823	4	3	1
51392	R640	UK [England]	2015	carrier	Neisseria meningitidis		X	X	13	3	57	209	3	8	25	7	17	21	26	5	ST-1157 complex	3655	3	3	1
51393	R642	UK [England]	2015	carrier	Neisseria meningitidis		cnl	cnl	4	1	1	206	4	5	4	17	15	30	650	12	ST-198 complex	10042	4	3	1
51394	R663	UK [England]	2015	carrier	Neisseria meningitidis		Y	Y	25	1	1	26	4	12	5	18	9	11	9	17	ST-23 complex	1655	4	4	2
51400	R683	UK [England]	2015	carrier	Neisseria meningitidis		cnl	cnl	4	1	1	206	4	5	4	17	15	30	7	12	ST-198 complex	823	4	3	1
51403	R704	UK [England]	2015	carrier	Neisseria meningitidis		cnl	cnl	4	1	10	206	4	16	2	6	25	17	25	22	ST-53 complex	53	4	3	1
51406	R741	UK [England]	2015	carrier	Neisseria meningitidis		cnl	cnl	4	1	1	206	4	5	4	17	15	30	7	12	ST-198 complex	823	4	3	1
51407	R747	UK [England]	2015	carrier	Neisseria meningitidis		cnl	cnl	334	1	1	527	4	6	41	108	15	9	6	913		13445	4	4	2
51409	R765	UK [England]	2015	carrier	Neisseria meningitidis		Y	Y	24	1	1	27	4	2	7	6	17	9	6	8	ST-167 complex	1624	4	4	2
51410	R769	UK [England]	2015	carrier	Neisseria meningitidis		Y	Y	25	1	1	26	4	10	5	18	9	11	9	17	ST-23 complex	23	4	4	2
51411	R777	UK [England]	2015	carrier	Neisseria meningitidis		Y	Y	25	1	1	26	4	10	5	18	9	11	9	17	ST-23 complex	23	4	4	2
51177	R779	UK [England]	2015	carrier	Neisseria meningitidis		W	W	22	1	1	1	4	2	3	4	3	8	4	6	ST-11 complex	11	4	4	2
51415	R788	UK [England]	2015	carrier	Neisseria meningitidis		cnl	cnl	102	8	10	2	20	16	2	6	25	17	25	22	ST-53 complex	53	20	4	2
51417	R796	UK [England]	2015	carrier	Neisseria meningitidis		cnl	cnl	21	1	1	7	4	16	2	159	92	77	25	112	ST-1117 complex	1117	4	4	2
51419	R803	UK [England]	2015	carrier	Neisseria meningitidis		cnl	cnl	4	1	1	206	4	5	4	17	15	30	7	12	ST-198 complex	823	4	3	1
51420	R805	UK [England]	2015	carrier	Neisseria meningitidis		Y	Y	19	1	1	93	13	2	16	6	17	9	18	8	ST-167 complex	168	13	4	2
51421	R808	UK [England]	2015	carrier	Neisseria meningitidis		B	B	23	111	1	431	NA	8	5	197	17	8	21	2	ST-865 complex	2403	NA	NA	2
51178	R811	UK [England]	2015	carrier	Neisseria meningitidis		W	W	22	1	1	1	4	2	3	4	3	8	4	6	ST-11 complex	11	4	4	2
51422	R814	UK [England]	2015	carrier	Neisseria meningitidis		B	B	23	111	1	431	NA	8	5	197	17	8	21	2	ST-865 complex	2403	NA	NA	2
51423	R815	UK [England]	2015	carrier	Neisseria meningitidis		Y	Y	25	1	1	26	4	10	5	18	9	11	9	17	ST-23 complex	23	4	4	2
51424	R822	UK [England]	2015	carrier	Neisseria meningitidis		Y	Y	25	1	1	26	4	10	5	18	9	11	9	17	ST-23 complex	23	4	4	2
51179	R825	UK [England]	2015	carrier	Neisseria meningitidis		W	W	22	1	1	1	4	2	3	4	3	8	4	6	ST-11 complex	11	4	4	2
51426	R832	UK [England]	2015	carrier	Neisseria meningitidis		cnl	cnl	94	58	56	89	NA	5	4	38	15	22	40	13	ST-1136 complex	1136	NA	NA	3
51929	R837	UK [England]	2015	carrier	Neisseria meningitidis		cnl	cnl	4	1	1	206	4	5	4	17	15	30	7	12	ST-198 complex	823	4	3	1
51180	R838	UK [England]	2015	carrier	Neisseria meningitidis		W	W	22	1	1	1	4	2	3	4	3	8	4	6	ST-11 complex	11	4	4	2
51427	R839	UK [England]	2015	carrier	Neisseria meningitidis		cnl	cnl	102	8	10	2	20	16	2	6	25	875	25	22	ST-53 complex	13400	20	4	2
51430	R850	UK [England]	2015	carrier	Neisseria meningitidis		cnl	cnl	102	8	10	2	20	16	2	6	25	17	25	22	ST-53 complex	53	20	4	2
51181	R852	UK [England]	2015	carrier	Neisseria meningitidis		W	W	4	112	1	206	106	2	3	4	3	8	4	6	ST-11 complex	11	NA	NA	1
51431	R855	UK [England]	2015	carrier	Neisseria meningitidis		cnl	cnl	25	1	1	26	4	8	5	15	56	9	21	2	ST-865 complex	4237	4	4	2

51432	R856	UK [England]	2015	carrier	Neisseria meningitidis		cnl	cnl	102	8	10	2	20	16	2	6	25	17	25	22	ST-53 complex	53	20	4	2
51433	R866	UK [England]	2015	carrier	Neisseria meningitidis		cnl	cnl	334	1	1	527	4	6	41	108	15	9	6	913		13445	4	4	2
51434	R872	UK [England]	2015	carrier	Neisseria meningitidis		Y	Y	25	1	1	26	4	12	5	18	332	77	9	17	ST-23 complex	12176	4	4	2
51182	R888	UK [England]	2015	carrier	Neisseria meningitidis		W	W	22	1	1	1	4	2	3	4	3	8	4	6	ST-11 complex	11	4	4	2
51183	R889	UK [England]	2015	carrier	Neisseria meningitidis		W	W	22	1	1	1	4	2	3	4	3	8	4	6	ST-11 complex	11	4	4	2
51438	R892	UK [England]	2015	carrier	Neisseria meningitidis		B	B	23	250	1	431	NA	8	5	15	17	8	21	2	ST-865 complex	865	NA	NA	2
51933	R902	UK [England]	2015	carrier	Neisseria meningitidis		Y	Y	25	1	1	26	4	12	5	18	9	11	9	17	ST-23 complex	1655	4	4	2
51439	R910	UK [England]	2015	carrier	Neisseria meningitidis		cnl	cnl	4	1	1	206	4	5	4	17	15	30	7	12	ST-198 complex	823	4	3	1
51442	R914	UK [England]	2015	carrier	Neisseria meningitidis		cnl	cnl	102	8	1	2	20	16	2	4	13	17	25	22	ST-53 complex	13395	20	4	2
51443	R915	UK [England]	2015	carrier	Neisseria meningitidis		cnl	cnl	4	1	10	206	4	16	2	6	25	17	25	22	ST-53 complex	53	4	3	1
51184	R920	UK [England]	2015	carrier	Neisseria meningitidis		W	W	22	1	1	1	4	2	3	4	3	8	4	6	ST-11 complex	11	4	4	2
51446	R937	UK [England]	2015	carrier	Neisseria meningitidis		cnl	cnl	14	7	1	142	6	3	6	9	5	9	6	9	ST-41/44 complex	41	6	2	1
51185	R941	UK [England]	2015	carrier	Neisseria meningitidis		W	W	4	112	1	206	106	2	3	4	3	8	4	6	ST-11 complex	11	NA	NA	1
51186	R949	UK [England]	2015	carrier	Neisseria meningitidis		W	W	4	112	1	206	106	2	3	4	3	8	4	6	ST-11 complex	11	NA	NA	1
51449	R950	UK [England]	2015	carrier	Neisseria meningitidis		Y	Y	25	1	1	26	4	12	4	6	17	5	24	2	ST-103 complex	13221	4	4	2
51450	R951	UK [England]	2015	carrier	Neisseria meningitidis		cnl	cnl	4	1	1	206	4	5	4	17	15	30	7	12	ST-198 complex	823	4	3	1
51452	R953	UK [England]	2015	carrier	Neisseria meningitidis		cnl	cnl	102	8	10	2	20	16	2	6	25	17	25	22	ST-53 complex	53	20	4	2
51187	R958	UK [England]	2015	carrier	Neisseria meningitidis		W	W	22	1	1	1	4	2	3	4	3	8	4	6	ST-11 complex	11	4	4	2
51456	R962	UK [England]	2015	carrier	Neisseria meningitidis		cnl	cnl	21	1	1	7	4	16	2	159	92	77	25	112	ST-1117 complex	1117	4	4	2
51458	R965	UK [England]	2015	carrier	Neisseria meningitidis		cnl	cnl	985	8	10	787	20	16	2	6	25	17	25	22	ST-53 complex	53	20	4	2
51462	R973	UK [England]	2015	carrier	Neisseria meningitidis		cnl	cnl	985	8	10	787	20	16	2	6	25	17	25	22	ST-53 complex	53	20	4	2
51188	R980	UK [England]	2015	carrier	Neisseria meningitidis		W	W	22	1	1	1	4	2	3	4	3	8	4	6	ST-11 complex	11	4	4	2
51189	R990	UK [England]	2015	carrier	Neisseria meningitidis		W	W	22	1	1	1	4	2	3	4	3	8	4	6	ST-11 complex	11	4	4	2
51190	R998	UK [England]	2015	carrier	Neisseria meningitidis		W	W	22	1	1	1	4	2	3	4	3	8	4	6	ST-11 complex	11	4	4	2
91586	SMG-19-1068	UK [Scotland]	2013	invasive (unspecified/other)	Neisseria meningitidis		B	B	260	1	1	452	13	4	10	2	9	38	11	16	ST-269 complex	5372	13	3	1
91587	SMG-19-1077	UK [Scotland]	2013	invasive (unspecified/other)	Neisseria meningitidis		B	B	260	1	1	452	13	4	10	2	9	38	11	16	ST-269 complex	5372	13	3	1
91588	SMG-19-1089	UK [Scotland]	2012	invasive (unspecified/other)	Neisseria meningitidis		B	B	260	1	1	452	13	4	10	2	9	38	11	16	ST-269 complex	5372	13	3	1
91589	SMG-19-1098	UK [Scotland]	2012	invasive (unspecified/other)	Neisseria meningitidis		B	B	260	1	1	452	13	4	10	2	9	38	11	16	ST-269 complex	5372	13	3	1
91545	SMG-19-1707	UK [Scotland]	2015	invasive (unspecified/other)	Neisseria meningitidis		Y	Y	19	1	1	93	13	2	16	6	17	9	11	2		11520	13	4	2
63138	SMG-19-1964	UK [Scotland]	2014	invasive (unspecified/other)	Neisseria meningitidis		Y	Y	25	1	1	26	4	12	5	18	9	11	9	17	ST-23 complex	1655	4	4	2
71428	SMG-19-2267	UK [Scotland]	2011	invasive (unspecified/other)	Neisseria meningitidis		C	C	22	1	1	1	4	2	3	4	3	8	4	6	ST-11 complex	11	4	4	2
71429	SMG-19-2270	UK [Scotland]	2012	invasive (unspecified/other)	Neisseria meningitidis		B	B	8	145	121	399	124	9	6	9	9	9	6	9	ST-41/44 complex	44	NA	NA	1
71430	SMG-19-2274	UK [Scotland]	2011	invasive (unspecified/other)	Neisseria meningitidis		C	C	1	6	7	100	7	4	10	5	4	6	3	8	ST-32 complex	32	7	1	1
71431	SMG-19-2279	UK [Scotland]	2013	invasive (unspecified/other)	Neisseria meningitidis		B	B	15	4	2	231	1	4	10	15	17	8	11	9	ST-269 complex	1049	1	1	1
71432	SMG-19-2285	UK [Scotland]	2011	invasive (unspecified/other)	Neisseria meningitidis		B	B	13	3	1	145	3	17	5	1	17	38	26	2	ST-60 complex	8787	3	3	1
71433	SMG-19-2287	UK [Scotland]	2011	invasive (unspecified/other)	Neisseria meningitidis		Y	Y	23	1	1	4	4	9	7	6	560	11	18	8	ST-167 complex	8725	4	4	2
71434	SMG-19-2288	UK [Scotland]	2011	invasive (unspecified/other)	Neisseria meningitidis		NG	NG	1	6	3	100	7	8	10	5	4	5	3	8	ST-32 complex	34	7	1	1
71435	SMG-19-2292	UK [Scotland]	2011	invasive (unspecified/other)	Neisseria meningitidis		cnl	cnl	102	8	10	2	20	16	2	6	25	17	25	22	ST-53 complex	53	20	4	2
71436	SMG-19-2293	UK [Scotland]	2011	invasive (unspecified/other)	Neisseria meningitidis		W	W	16	1	1	60	4	11	5	18	154	11	24	21	ST-22 complex	1281	4	4	2
71801	SMG-19-2329	UK [Scotland]	2009	invasive (unspecified/other)	Neisseria meningitidis		cnl	cnl	14	7	1	142	6	3	6	9	5	9	6	252	ST-41/44 complex	7710	6	2	1
71802	SMG-19-2334	UK [Scotland]	2009	invasive (unspecified/other)	Neisseria meningitidis		cnl	cnl	102	8	10	2	20	16	2	6	25	17	53	22	ST-53 complex	2440	20	4	2
71803	SMG-19-2340	UK [Scotland]	2010	invasive (unspecified/other)	Neisseria meningitidis		E	E	1294	87	118	1151	NA	8	25	7	17	21	26	49	ST-1157 complex	1157	NA	NA	1
71804	SMG-19-2343	UK [Scotland]	2009	invasive (unspecified/other)	Neisseria meningitidis		B	B	19	1	1	65	4	9	20	9	9	9	6	2	ST-41/44 complex	1097	4	4	2
71805	SMG-19-2347	UK [Scotland]	2009	invasive (unspecified/other)	Neisseria meningitidis		cnl	cnl	14	7	1	142	6	3	6	9	5	9	6	252	ST-41/44 complex	7710	6	2	1
89507	SMG-19-924	UK [Scotland]	2015	invasive (unspecified/other)	Neisseria meningitidis		C	C	15	4	2	193	1	4	10	15	9	8	11	15	ST-269 complex	5494	1	1	1
89511	SMG-19-932	UK [Scotland]	2014	invasive (unspecified/other)	Neisseria meningitidis		Y	Y	25	1	1	26	4	12	5	18	9	11	9	17	ST-23 complex	1655	4	4	2
89512	SMG-19-937	UK [Scotland]	2014	invasive (unspecified/other)	Neisseria meningitidis		B	B	45	8	77	87	20	7	5	1	13	36	53	15	ST-213 complex	213	20	4	3
71949	SMG-20-102	UK [Scotland]	2006	invasive (unspecified/other)	Neisseria meningitidis		B	B	14	1	57	NA	13	427	NA	12	9	12	13	10		NA	13	3	1
77692	SMG-20-1025	UK [Scotland]	2015	invasive (unspecified/other)	Neisseria meningitidis		Y	Y	25	1	1	26	4	12	5	18	9	11	9	17	ST-23 complex	1655	4	4	2
79042	SMG-20-1087	UK [Scotland]	2012	invasive (unspecified/other)	Neisseria meningitidis	B	B	B	170	9	77	94	21	604	5	1	13	36	53	15	ST-213 complex	9753	NA	NA	3
79047	SMG-20-1104	UK [Scotland]	2012	invasive (unspecified/other)	Neisseria meningitidis	C	C	C	819	53	1	454	105	8	3	154	17	26	3	2		6610	NA	NA	1
71950	SMG-20-111	UK [Scotland]	2005	invasive (unspecified/other)	Neisseria meningitidis		X	X	84	12	1	70	28	2	5	2	9	15	20	5	ST-750 complex	750	NA	NA	3
79048	SMG-20-1110	UK [Scotland]	2011	invasive (unspecified/other)	Neisseria meningitidis	B	B	B	45	8	77	87	20	7	5	1	13	36	6	15	ST-213 complex	2660	20	4	3
71951	SMG-20-116	UK [Scotland]	2005	invasive (unspecified/other)	Neisseria meningitidis		B	B	15	4	2	193	1	4	10	15	9	8	11	9	ST-269 complex	269	1	1	1
71952	SMG-20-117	UK [Scotland]	2005	invasive (unspecified/other)	Neisseria meningitidis		Y	Y	104	1	57	10	4	10	5	18	59	11	9	17	ST-23 complex	183	4	4	2
71953	SMG-20-120	UK [Scotland]	2005	invasive (unspecified/other)	Neisseria meningitidis		cnl	cnl	970	1	10	714	15	16	2	6	25	17	25	22	ST-53 complex	53	15	4	2
81776	SMG-20-1206	UK [Scotland]	2015	invasive (unspecified/other)	Neisseria meningitidis		W	W	22	1	1	1	4	2	3	4	3	8	4	6	ST-11 complex	11	4	4	2
81777	SMG-20-1208	UK [Scotland]	2014	invasive (unspecified/other)	Neisseria meningitidis		B	B	47	1	1	90	15	12	5	12	35	60	22	17	ST-461 complex	461	15	4	3
81778	SMG-20-1209	UK [Scotland]	2015	invasive (unspecified/other)	Neisseria meningitidis		W	W	22	1	1	1	4	2	3	4	3	8	4	6	ST-11 complex	11	4	4	2
81788	SMG-20-1218	UK [Scotland]	2015	invasive (unspecified/other)	Neisseria meningitidis		B	B	19	1	1	83	4	4	10	2	5	38	6	9		3068	4	4	2
81789	SMG-20-1251	UK [Scotland]	2015	invasive (unspecified/other)	Neisseria meningitidis		B	B	950	113	1	722	131	8	25	7	17	21	24	49	ST-1157 complex	1727	NA	NA	3
81790	SMG-20-1263	UK [Scotland]	2014	invasive (unspecified/other)	Neisseria meningitidis		Y	Y	25	1	1	26	4	10	5	18	9	11	9	17	ST-23 complex	23	4	4	2
71908	SMG-20-2	UK [Scotland]	2007	invasive (unspecified/other)	Neisseria meningitidis		C	C	1	6	7	100	7	4	10	5	4	6	188	8	ST-32 complex	2000	7	1	1
99852	SMG-20-2096	UK [Scotland]	2015	invasive (unspecified/other)	Neisseria meningitidis		C	C	22	1	1	1	4	2	3	4	3	8	4	6	ST-11 complex	11	4	4	2
71913	SMG-20-21	UK [Scotland]	2007	invasive (unspecified/other)	Neisseria meningitidis		W	W	16	1	1	60	4	11	5	18	8	78	24	21	ST-22 complex	1224	4	4	2
71914	SMG-20-23	UK [Scotland]	2008	invasive (unspecified/other)	Neisseria meningitidis		B	B	45	33	77	87	14	7	6	1	13	36	53	15	ST-213 complex	4250	NA	NA	3
71915	SMG-20-25	UK [Scotland]	2010	invasive (unspecified/other)	Neisseria meningitidis		cnl	cnl	25	1	1	26	4	16	2	6	25	17	25	22	ST-53 complex	53	4	4	2
71916	SMG-20-28	UK [Scotland]	2008	invasive (unspecified/other)	Neisseria meningitidis		cnl	cnl	4	1	1	206	4	5	4	17	15	30	7	12	ST-198 complex	823	4	3	1
72594	SMG-20-288	UK [Scotland]	2004	invasive (unspecified/other)	Neisseria meningitidis		B	B	15	4	2	193	1	4	10	15	9	8	11	9	ST-269 complex	269	1	1	1
71917	SMG-20-29	UK [Scotland]	2008	invasive (unspecified/other)	Neisseria meningitidis		E	E	13	3	57	209	3	8	25	7	17	21	26	49	ST-1157 complex	1157	3	3	1
72595	SMG-20-290	UK [Scotland]	2004	invasive (unspecified/other)	Neisseria meningitidis		NG	NG	15	4	2	193	1	4	10	23	9	8	11	13	ST-269 complex	3500	1	1	1
72596	SMG-20-297	UK [Scotland]	2002	invasive (unspecified/other)	Neisseria meningitidis		E	E	13	3	1	145	3	17	5	19	5	3	26	2	ST-60 complex	1650	3	3	1
72597	SMG-20-299	UK [Scotland]	2004	invasive (unspecified/other)	Neisseria meningitidis		B	B	19	1	1	83	4	4	10	2	5	3	11	9	ST-269 complex	1163	4	4	2
71909	SMG-20-3	UK [Scotland]	2008	invasive (unspecified/other)	Neisseria meningitidis		B	B	13	3	1	145	3	17	5	19	17	3	26	2	ST-60 complex	60	3	3	1
72598	SMG-20-301	UK [Scotland]	2004	invasive (unspecified/other)	Neisseria meningitidis		B	B	14	7	4	142	6	3	6	9	5	9	6	9	ST-41/44 complex	41	6	2	1
72599	SMG-20-309	UK [Scotland]	2004	invasive (unspecified/other)	Neisseria meningitidis		B	B	45	8	77	87	20	7	5	1	13	36	53	15	ST-213 complex	213	20	4	3
71918	SMG-20-31	UK [Scotland]	2010	invasive (unspecified/other)	Neisseria meningitidis		cnl	cnl	102	8	10	2	20	16	2	6	25	17	25	22	ST-53 complex	53	20	4	2
71919	SMG-20-32	UK [Scotland]	2010	invasive (unspecified/other)	Neisseria meningitidis		cnl	cnl	4	1	1	206	4	5	4	17	15	30	7	12	ST-198 complex	823	4	3	1
72626	SMG-20-369	UK [Scotland]	2004	invasive (unspecified/other)	Neisseria meningitidis		B	B	24	1	2	25	4	15	3	133	13	8	19	15		1162	4	4	2
72627	SMG-20-372	UK [Scotland]	2004	invasive (unspecified/other)	Neisseria meningitidis		B	B	25	1	1	26	4	8	4	6	17	5	18	21	ST-103 complex	1347	4	4	2
71910	SMG-20-4	UK [Scotland]	2008	invasive (unspecified/other)	Neisseria meningitidis		B	B	19	1	1	65	4	9	20	9	9	9	6	2	ST-41/44 complex	1097	4	4	2
71911	SMG-20-5	UK [Scotland]	2007	invasive (unspecified/other)	Neisseria meningitidis		C	C	15	4	2	193	1	4	10	15	9	8	11	13	ST-269 complex	467	1	1	1
71934	SMG-20-69	UK [Scotland]	2006	invasive (unspecified/other)	Neisseria meningitidis		B	B	15	4	2	193	1	4	10	15	9	8	11	9	ST-269 complex	269	1	1	1
71912	SMG-20-7	UK [Scotland]	2010	invasive (unspecified/other)	Neisseria meningitidis		B	B	16	1	4	59	4	4	NA	550	9	6	4	12		NA	4	4	2
71935	SMG-20-71	UK [Scotland]	2008	invasive (unspecified/other)	Neisseria meningitidis		NG	NG	15	4	2	193	1	4	10	15	9	8	11	9	ST-269 complex	269	1	1	1
71936	SMG-20-79	UK [Scotland]	2006	invasive (unspecified/other)	Neisseria meningitidis		B	B	13	3	1	145	3	7	5	1	13	82	53	15	ST-213 complex	575	3	3	1
71937	SMG-20-80	UK [Scotland]	2008	invasive (unspecified/other)	Neisseria meningitidis		B	B	19	1	1	83	4	4	10	2	5	3	11	9	ST-269 complex	1163	4	4	2
71938	SMG-20-88	UK [Scotland]	2006	invasive (unspecified/other)	Neisseria meningitidis		B	B	15	4	2	193	1	4	10	15	9	8	11	9	ST-269 complex	269	1	1	1
71939	SMG-20-89	UK [Scotland]	2006	invasive (unspecified/other)	Neisseria meningitidis		E	E	25	1	1	26	4	8	3	15	60	8	21	2	ST-865 complex	1872	4	4	2
71948	SMG-20-94	UK [Scotland]	2006	invasive (unspecified/other)	Neisseria meningitidis		NG	NG	15	4	2	193	1	4	10	15	9	8	11	13	ST-269 complex	467	1	1	1
50318	ST40001	UK [England]	2015	carrier	Neisseria meningitidis	Y	Y	Y	25	1	1	26	4	12	5	18	9	11	9	17	ST-23 complex	1655	4	4	2
43820	ST40016	UK [England]	2015	carrier	Neisseria meningitidis	NG	cnl	cnl	102	8	10	2	20	16	2	6	25	17	25	22	ST-53 complex	53	20	4	2
44367	ST40031	UK [England]	2015	carrier	Neisseria meningitidis	W	W	W	22	1	1	1	4	2	3	4	3	8	4	6	ST-11 complex	11	4	4	2
44426	ST40036	UK [England]	2015	carrier	Neisseria meningitidis	NG	W	W	22	1	1	1	4	2	3	4	3	8	4	6	ST-11 complex	11	4	4	2
52677	ST40037	UK [England]	2015	carrier	Neisseria meningitidis	B	B	B	213	NA	1	711	191	3	6	9	9	9	6	9	ST-41/44 complex	303	NA	NA	1
44395	ST40045	UK [England]	2015	carrier	Neisseria meningitidis	NG	cnl	cnl	102	8	10	2	20	16	2	6	25	17	25	22	ST-53 complex	53	20	4	2
50319	ST40050	UK [England]	2015	carrier	Neisseria meningitidis	NG	cnl	cnl	970	1	10	714	15	806	2	6	25	17	25	22	ST-53 complex	12700	15	4	2
43822	ST40059	UK [England]	2015	carrier	Neisseria meningitidis	NG	B	B	16	1	4	59	4	4	10	136	17	5	10	12	ST-35 complex	815	4	4	2
50320	ST40070	UK [England]	2015	carrier	Neisseria meningitidis	NG	NG	NG	683	4	2	176	1	4	10	15	9	8	11	9	ST-269 complex	269	1	1	2
50321	ST40119	UK [England]	2015	carrier	Neisseria meningitidis	Y	Y	Y	25	1	1	26	4	10	5	18	9	11	9	17	ST-23 complex	23	4	4	2
44391	ST40125	UK [England]	2015	carrier	Neisseria meningitidis	NG	E	E	13	3	1	145	3	17	5	19	17	62	26	2	ST-60 complex	10454	3	3	1
44390	ST40138	UK [England]	2015	carrier	Neisseria meningitidis	Y	Y	Y	25	1	1	26	4	10	5	18	9	11	9	17	ST-23 complex	23	4	4	2
44416	ST40147	UK [England]	2015	carrier	Neisseria meningitidis	B	B	B	45	8	77	87	20	7	5	12	2	36	797	15	ST-213 complex	12352	20	4	3
44418	ST40158	UK [England]	2015	carrier	Neisseria meningitidis	W	W	W	22	1	1	1	4	2	3	4	3	8	4	6	ST-11 complex	11	4	4	2
44399	ST40164	UK [England]	2015	carrier	Neisseria meningitidis	NG	E	E	13	3	57	209	3	8	25	7	17	21	26	49	ST-1157 complex	1157	3	3	1
50322	ST40172	UK [England]	2015	carrier	Neisseria meningitidis	NG	cnl	cnl	21	1	1	7	4	16	2	159	92	77	25	112	ST-1117 complex	1117	4	4	2
44424	ST40190	UK [England]	2015	carrier	Neisseria meningitidis	NG	cnl	cnl	21	1	1	7	4	16	2	159	92	77	25	112	ST-1117 complex	1117	4	4	2
43823	ST40197	UK [England]	2015	carrier	Neisseria meningitidis	Y	Y	Y	25	1	1	26	4	10	5	18	9	11	9	17	ST-23 complex	23	4	4	2
44423	ST40200	UK [England]	2015	carrier	Neisseria meningitidis	NG	W	W	22	1	1	1	4	662	3	4	3	8	4	6	ST-11 complex	10651	4	4	2
50085	ST40217	UK [England]	2015	carrier	Neisseria meningitidis	NG	cnl	cnl	4	1	1	785	4	5	4	17	15	30	7	12	ST-198 complex	823	4	3	1
44422	ST40229	UK [England]	2015	carrier	Neisseria meningitidis	NG	E	E	13	3	57	209	3	8	25	7	17	21	26	49	ST-1157 complex	1157	3	3	1
44420	ST40264	UK [England]	2015	carrier	Neisseria meningitidis	Y	Y	Y	25	1	1	26	4	10	5	18	9	11	9	17	ST-23 complex	23	4	4	2
44419	ST40271	UK [England]	2015	carrier	Neisseria meningitidis	B	B	B	45	8	77	87	20	7	5	1	13	36	53	15	ST-213 complex	213	20	4	3
44408	ST40285	UK [England]	2015	carrier	Neisseria meningitidis	B	B	B	45	8	1	87	20	7	5	1	13	36	53	15	ST-213 complex	213	20	4	3
43824	ST40291	UK [England]	2015	carrier	Neisseria meningitidis	NG	E	E	13	3	57	209	3	8	25	7	17	21	26	49	ST-1157 complex	1157	3	3	1
44417	ST40292	UK [England]	2015	carrier	Neisseria meningitidis	W	W	W	971	1	1	716	4	11	5	18	8	11	24	21	ST-22 complex	22	4	4	2
50323	ST40310	UK [England]	2015	carrier	Neisseria meningitidis	NG	cnl	cnl	102	8	10	2	20	16	2	6	25	17	25	22	ST-53 complex	53	20	4	2
44427	ST40326	UK [England]	2015	carrier	Neisseria meningitidis	Y	Y	Y	25	1	1	26	4	12	5	18	332	77	9	17	ST-23 complex	12176	4	4	2
44415	ST40335	UK [England]	2015	carrier	Neisseria meningitidis	NG	cnl	cnl	14	7	1	142	6	3	6	9	5	9	6	9	ST-41/44 complex	41	6	2	1
44414	ST40338	UK [England]	2015	carrier	Neisseria meningitidis	W	W	W	16	22	1	97	32	11	5	168	8	11	4	21	ST-22 complex	1286	NA	NA	2
44412	ST40348	UK [England]	2015	carrier	Neisseria meningitidis	NG	B	B	19	1	NA	65	4	6	5	75	9	1	34	307		12232	4	4	2
44411	ST40352	UK [England]	2015	carrier	Neisseria meningitidis	NG	Y	Y	25	1	1	26	4	12	5	18	9	11	9	17	ST-23 complex	1655	4	4	2
50324	ST40366	UK [England]	2015	carrier	Neisseria meningitidis	NG	NG	NG	16	1	4	59	4	4	10	11	18	6	10	17	ST-35 complex	8593	4	4	2
44409	ST40369	UK [England]	2015	carrier	Neisseria meningitidis	W	W	W	22	1	1	1	4	2	3	4	3	8	4	6	ST-11 complex	11	4	4	2
44387	ST40394	UK [England]	2015	carrier	Neisseria meningitidis	B	B	B	25	1	1	26	4	17	6	9	5	9	6	9	ST-41/44 complex	3447	4	4	2
50239	ST40435	UK [England]	2015	carrier	Neisseria meningitidis	Y	Y	Y	25	1	1	26	4	10	5	18	9	681	9	17	ST-23 complex	12231	4	4	2
44365	ST40485	UK [England]	2015	carrier	Neisseria meningitidis	Y	Y	Y	104	1	57	10	4	10	5	18	59	11	9	17	ST-23 complex	183	4	4	2
44364	ST40497	UK [England]	2015	carrier	Neisseria meningitidis	Y	Y	Y	25	1	1	26	4	12	5	18	9	11	9	17	ST-23 complex	1655	4	4	2
44363	ST40506	UK [England]	2015	carrier	Neisseria meningitidis	NG	Y	Y	109	1	1	712	4	3	7	15	17	9	6	8		12230	4	4	2
50326	ST40513	UK [England]	2015	carrier	Neisseria meningitidis	NG	cnl	cnl	102	8	10	2	20	16	2	18	25	17	25	22	ST-53 complex	2441	20	4	2
44361	ST40514	UK [England]	2015	carrier	Neisseria meningitidis	NG	cnl	cnl	4	1	1	206	4	5	4	17	15	30	7	12	ST-198 complex	823	4	3	1
44350	ST40527	UK [England]	2015	carrier	Neisseria meningitidis	NG	Y	Y	25	1	1	26	4	12	5	18	9	11	9	17	ST-23 complex	1655	4	4	2
44368	ST40538	UK [England]	2015	carrier	Neisseria meningitidis	NG	cnl	cnl	4	1	1	206	4	5	4	17	15	30	7	12	ST-198 complex	823	4	3	1
44357	ST40568	UK [England]	2015	carrier	Neisseria meningitidis	NG	cnl	cnl	16	1	1	60	4	3	6	9	8	9	6	9	ST-41/44 complex	3617	4	4	2
44356	ST40578	UK [England]	2015	carrier	Neisseria meningitidis	NG	cnl	cnl	21	1	1	7	4	16	2	159	92	77	25	112	ST-1117 complex	1117	4	4	2
44355	ST40587	UK [England]	2015	carrier	Neisseria meningitidis	NG	B	B	19	1	1	83	4	4	10	2	5	3	11	9	ST-269 complex	1163	4	4	2
44353	ST40610	UK [England]	2015	carrier	Neisseria meningitidis	NG	E	E	13	3	57	209	3	8	25	7	17	21	26	49	ST-1157 complex	1157	3	3	1
44377	ST40633	UK [England]	2015	carrier	Neisseria meningitidis	NG	B	B	969	1	4	713	4	4	10	72	9	6	1	12	ST-35 complex	5423	4	4	2

44379	ST40640	UK [England]	2015	carrier	Neisseria meningitidis	NG	cnl	cnl	102	8	10	2	20	16	2	6	25	17	25	22	ST-53 complex	53	20	4	2
50327	ST40691	UK [England]	2015	carrier	Neisseria meningitidis	W	W	W	22	1	1	1	4	2	3	4	3	8	4	6	ST-11 complex	11	4	4	2
44383	ST40755	UK [England]	2015	carrier	Neisseria meningitidis	B	B	B	4	1	59	84	4	4	10	11	17	6	10	12	ST-35 complex	457	4	3	1
44382	ST40774	UK [England]	2015	carrier	Neisseria meningitidis	NG	Y	Y	19	1	1	93	13	2	16	6	17	9	18	2	ST-103 complex	5987	13	4	2
52680	ST40801	UK [England]	2015	carrier	Neisseria meningitidis	NG	B	B	13	3	59	145	3	8	5	15	17	8	21	2	ST-865 complex	865	3	3	1
50328	ST40803	UK [England]	2015	carrier	Neisseria meningitidis	NG	Y	Y	25	1	1	26	4	12	5	18	9	11	9	17	ST-23 complex	1655	4	4	2
44369	ST40808	UK [England]	2015	carrier	Neisseria meningitidis	NG	cnl	cnl	21	1	1	7	4	16	2	159	92	77	25	112	ST-1117 complex	1117	4	4	2
44378	ST40814	UK [England]	2015	carrier	Neisseria meningitidis	NG	cnl	cnl	102	8	10	2	20	16	2	6	25	17	25	22	ST-53 complex	53	20	4	2
44388	ST40816	UK [England]	2015	carrier	Neisseria meningitidis	B	B	B	4	1	1	206	4	4	10	34	5	38	11	9	ST-269 complex	1161	4	3	1
44376	ST40826	UK [England]	2015	carrier	Neisseria meningitidis	Y	Y	Y	25	1	1	26	4	12	5	18	9	11	9	17	ST-23 complex	1655	4	4	2
50329	ST40837	UK [England]	2015	carrier	Neisseria meningitidis	NG	E	E	13	3	1	145	3	17	5	19	17	3	26	2	ST-60 complex	60	3	3	1
50330	ST40899	UK [England]	2015	carrier	Neisseria meningitidis	NG	cnl	cnl	102	8	10	2	20	16	2	6	25	17	25	22	ST-53 complex	53	20	4	2
44372	ST40907	UK [England]	2015	carrier	Neisseria meningitidis	B	B	B	47	1	57	90	15	451	5	12	35	192	22	17	ST-461 complex	7261	15	4	3
50331	ST40951	UK [England]	2015	carrier	Neisseria meningitidis	NG	Y	Y	23	1	1	4	4	2	7	6	9	16	9	8	ST-167 complex	767	4	4	2
50332	ST40967	UK [England]	2015	carrier	Neisseria meningitidis	NG	Z	Z	502	1	1	747	4	8	3	6	17	29	18	9	ST-103 complex	1418	4	3	1
52682	ST40978	UK [England]	2015	carrier	Neisseria meningitidis	B	B	B	45	8	77	87	20	7	5	1	13	36	53	15	ST-213 complex	213	20	4	3
44119	ST40982	UK [England]	2015	carrier	Neisseria meningitidis	NG	cnl	cnl	102	8	10	705	20	16	2	6	25	17	25	22	ST-53 complex	53	20	4	2
44274	ST41002	UK [England]	2015	carrier	Neisseria meningitidis	NG	X	X	527	3	57	709	3	8	5	12	11	8	22	7		4011	3	4	2
50333	ST41005	UK [England]	2015	carrier	Neisseria meningitidis	Y	Y	Y	25	1	1	26	4	12	5	18	9	11	9	17	ST-23 complex	1655	4	4	2
52683	ST41048	UK [England]	2015	carrier	Neisseria meningitidis	NG	NG	NG	24	1	1	25	4	6	6	9	9	11	6	17	ST-41/44 complex	12219	4	4	2
50335	ST41053	UK [England]	2015	carrier	Neisseria meningitidis	Y	Y	Y	25	1	1	26	4	12	286	18	9	11	9	17	ST-23 complex	6463	4	4	2
44156	ST41071	UK [England]	2015	carrier	Neisseria meningitidis	B	B	B	45	8	77	87	20	7	5	1	13	36	53	15	ST-213 complex	213	20	4	3
50336	ST41086	UK [England]	2015	carrier	Neisseria meningitidis	W	W	W	22	1	1	1	4	2	3	4	3	8	4	6	ST-11 complex	11	4	4	2
44174	ST41087	UK [England]	2015	carrier	Neisseria meningitidis	NG	Y	Y	25	1	1	26	4	12	5	18	9	11	9	17	ST-23 complex	1655	4	4	2
44163	ST41089	UK [England]	2015	carrier	Neisseria meningitidis	NG	B	B	14	1	1	142	13	3	6	19	5	3	6	9	ST-41/44 complex	340	13	3	1
44162	ST41108	UK [England]	2015	carrier	Neisseria meningitidis	W	W	W	22	1	1	1	4	2	3	4	3	8	4	6	ST-11 complex	11	4	4	2
44161	ST41109	UK [England]	2015	carrier	Neisseria meningitidis	W	W	W	22	1	1	1	4	2	3	4	3	8	4	6	ST-11 complex	11	4	4	2
44160	ST41111	UK [England]	2015	carrier	Neisseria meningitidis	NG	cnl	cnl	21	1	1	7	4	16	2	159	92	77	25	112	ST-1117 complex	1117	4	4	2
50337	ST41115	UK [England]	2015	carrier	Neisseria meningitidis	NG	E	E	13	3	1	145	3	17	5	19	17	3	26	2	ST-60 complex	60	3	3	1
44158	ST41118	UK [England]	2015	carrier	Neisseria meningitidis	NG	cnl	cnl	4	1	1	206	4	5	4	17	15	30	7	12	ST-198 complex	823	4	3	1
44157	ST41121	UK [England]	2015	carrier	Neisseria meningitidis	Y	Y	Y	25	1	1	26	4	12	5	18	9	11	9	17	ST-23 complex	1655	4	4	2
50338	ST41125	UK [England]	2015	carrier	Neisseria meningitidis	NG	Y	Y	25	1	1	26	4	12	5	18	9	11	9	17	ST-23 complex	1655	4	4	2
44185	ST41126	UK [England]	2015	carrier	Neisseria meningitidis	NG	cnl	cnl	102	8	10	2	20	16	2	6	25	17	25	22	ST-53 complex	53	20	4	2
44166	ST41130	UK [England]	2015	carrier	Neisseria meningitidis	B	B	B	45	8	1	87	20	7	5	4	13	36	53	15	ST-213 complex	3722	20	4	3
44192	ST41136	UK [England]	2015	carrier	Neisseria meningitidis	B	B	B	47	1	57	90	15	451	5	12	35	192	22	17	ST-461 complex	7261	15	4	3
50339	ST41140	UK [England]	2015	carrier	Neisseria meningitidis	NG	E	E	13	3	57	209	3	8	25	7	17	21	26	49	ST-1157 complex	1157	3	3	1
44190	ST41142	UK [England]	2015	carrier	Neisseria meningitidis	Y	Y	Y	25	1	1	26	4	12	5	18	17	11	9	17	ST-23 complex	9453	4	4	2
50086	ST41151	UK [England]	2015	carrier	Neisseria meningitidis	NG	cnl	cnl	102	8	10	2	20	16	2	6	25	17	25	22	ST-53 complex	53	20	4	2
44189	ST41154	UK [England]	2015	carrier	Neisseria meningitidis	Y	Y	Y	25	1	1	26	4	12	5	18	9	11	798	17	ST-23 complex	12353	4	4	2
50087	ST41155	UK [England]	2015	carrier	Neisseria meningitidis	NG	cnl	cnl	102	8	10	2	20	16	2	6	25	17	25	22	ST-53 complex	53	20	4	2
50340	ST41161	UK [England]	2015	carrier	Neisseria meningitidis	W	W	W	22	1	1	1	4	2	3	4	3	8	4	6	ST-11 complex	11	4	4	2
50341	ST41162	UK [England]	2015	carrier	Neisseria meningitidis	NG	Y	Y	16	1	1	60	4	11	5	18	8	11	4	21	ST-22 complex	184	4	4	2
50088	ST41167	UK [England]	2015	carrier	Neisseria meningitidis	NG	B	B	510	28	8	599	30	4	10	48	4	6	3	8	ST-32 complex	7460	NA	NA	1
44186	ST41174	UK [England]	2015	carrier	Neisseria meningitidis	B	B	B	69	NA	1	248	188	4	5	679	4	6	3	8	ST-32 complex	9989	NA	NA	1
50089	ST41197	UK [England]	2015	carrier	Neisseria meningitidis	NG	E	E	13	3	57	209	3	8	581	7	17	21	26	49	ST-1157 complex	12690	3	3	1
44184	ST41205	UK [England]	2015	carrier	Neisseria meningitidis	W	W	W	16	1	1	60	4	11	5	18	154	11	24	21	ST-22 complex	1281	4	4	2
50342	ST41215	UK [England]	2015	carrier	Neisseria meningitidis	NG	Y	Y	119	1	1	98	4	6	3	4	9	8	13	169		1768	4	4	2
44182	ST41219	UK [England]	2015	carrier	Neisseria meningitidis	B	B	B	21	1	1	7	4	4	10	5	4	6	3	8	ST-32 complex	32	4	4	2
44181	ST41229	UK [England]	2015	carrier	Neisseria meningitidis	Y	Y	Y	25	1	1	26	4	10	5	18	9	11	9	17	ST-23 complex	23	4	4	2
50090	ST41241	UK [England]	2015	carrier	Neisseria meningitidis	NG	cnl	cnl	627	1	1	892	4	217	3	79	7	260	819	123		12691	4	4	3
50091	ST41251	UK [England]	2015	carrier	Neisseria meningitidis	Y	Y	Y	25	1	1	26	4	10	5	18	9	11	9	17	ST-23 complex	23	4	4	2
50092	ST41255	UK [England]	2015	carrier	Neisseria meningitidis	B	B	B	45	8	77	87	20	7	5	1	13	36	6	15	ST-213 complex	2660	20	4	3
50093	ST41268	UK [England]	2015	carrier	Neisseria meningitidis	W	W	W	22	1	1	1	4	2	3	4	3	8	4	6	ST-11 complex	11	4	4	2
50094	ST41288	UK [England]	2015	carrier	Neisseria meningitidis	NG	Z	Z	22	1	1	1	4	20	3	17	26	26	15	9		12692	4	4	2
50095	ST41293	UK [England]	2015	carrier	Neisseria meningitidis	NG	cnl	cnl	102	8	10	2	20	16	2	6	25	17	25	22	ST-53 complex	53	20	4	2
50096	ST41308	UK [England]	2015	carrier	Neisseria meningitidis	Y	Y	Y	25	1	1	26	4	10	5	18	9	11	9	17	ST-23 complex	23	4	4	2
44179	ST41315	UK [England]	2015	carrier	Neisseria meningitidis	NG	Z	Z	22	1	1	1	4	20	3	2	26	26	15	112		5953	4	4	2
50097	ST41323	UK [England]	2015	carrier	Neisseria meningitidis	NG	cnl	cnl	334	1	1	527	4	6	41	108	15	9	6	9		845	4	4	2
50098	ST41325	UK [England]	2015	carrier	Neisseria meningitidis	NG	cnl	cnl	21	1	1	7	4	16	2	159	92	77	25	112	ST-1117 complex	1117	4	4	2
50099	ST41328	UK [England]	2015	carrier	Neisseria meningitidis	W	W	W	16	1	1	60	4	11	5	18	8	78	24	21	ST-22 complex	1224	4	4	2
50100	ST41337	UK [England]	2015	carrier	Neisseria meningitidis	Y	Y	Y	25	1	1	26	4	12	5	18	9	11	9	17	ST-23 complex	1655	4	4	2
50101	ST41338	UK [England]	2015	carrier	Neisseria meningitidis	Y	Y	Y	25	1	1	26	4	12	5	18	9	11	9	17	ST-23 complex	1655	4	4	2
50102	ST41340	UK [England]	2015	carrier	Neisseria meningitidis	B	B	B	762	NA	1	679	189	7	5	1	13	36	53	15	ST-213 complex	213	NA	NA	1
50103	ST41343	UK [England]	2015	carrier	Neisseria meningitidis	W	W	W	22	1	1	1	4	2	3	4	3	8	4	6	ST-11 complex	11	4	4	2
50343	ST41347	UK [England]	2015	carrier	Neisseria meningitidis	Y	Y	Y	25	1	1	26	4	12	5	18	9	11	9	17	ST-23 complex	1655	4	4	2
44147	ST41353	UK [England]	2015	carrier	Neisseria meningitidis	B	B	B	45	8	77	87	20	7	5	1	13	36	53	15	ST-213 complex	213	20	4	3
52685	ST41357	UK [England]	2015	carrier	Neisseria meningitidis	NG	NG	NG	24	1	1	25	4	6	6	9	9	11	6	17	ST-41/44 complex	12219	4	4	2
44164	ST41366	UK [England]	2015	carrier	Neisseria meningitidis	NG	cnl	cnl	21	1	1	7	4	16	2	159	92	77	25	112	ST-1117 complex	1117	4	4	2
44134	ST41376	UK [England]	2015	carrier	Neisseria meningitidis	NG	NG	NG	13	3	57	145	3	9	5	9	15	50	112	16		12221	3	3	1
44133	ST41386	UK [England]	2015	carrier	Neisseria meningitidis	Y	Y	Y	25	1	1	26	4	12	5	18	9	11	9	17	ST-23 complex	1655	4	4	2
44131	ST41397	UK [England]	2015	carrier	Neisseria meningitidis	NG	E	E	13	3	1	209	3	8	25	7	17	21	26	49	ST-1157 complex	1157	3	3	1
50345	ST41402	UK [England]	2015	carrier	Neisseria meningitidis	Y	Y	Y	25	1	1	26	4	10	5	18	9	11	9	17	ST-23 complex	23	4	4	2
44129	ST41403	UK [England]	2015	carrier	Neisseria meningitidis	NG	cnl	cnl	4	1	1	206	4	5	4	17	15	30	7	12	ST-198 complex	823	4	3	1
44118	ST41413	UK [England]	2015	carrier	Neisseria meningitidis	Y	Y	Y	25	1	1	26	4	12	5	18	9	11	9	17	ST-23 complex	1655	4	4	2
44127	ST41424	UK [England]	2015	carrier	Neisseria meningitidis	B	B	B	45	8	1	87	20	7	5	1	13	36	53	15	ST-213 complex	213	20	4	3
50346	ST41427	UK [England]	2015	carrier	Neisseria meningitidis	Y	Y	Y	25	1	1	26	4	12	5	18	9	11	9	17	ST-23 complex	1655	4	4	2
44125	ST41439	UK [England]	2015	carrier	Neisseria meningitidis	NG	Z	Z	22	1	1	1	4	20	3	2	26	26	15	112		5953	4	4	2
52686	ST41447	UK [England]	2015	carrier	Neisseria meningitidis	B	B	B	45	8	77	87	20	7	5	1	13	36	53	15	ST-213 complex	213	20	4	3
44123	ST41451	UK [England]	2015	carrier	Neisseria meningitidis	W	W	W	22	1	1	1	4	2	3	4	3	8	4	6	ST-11 complex	11	4	4	2
50348	ST41452	UK [England]	2015	carrier	Neisseria meningitidis	W	W	W	16	1	1	60	4	11	5	18	8	11	24	21	ST-22 complex	22	4	4	2
50349	ST41455	UK [England]	2015	carrier	Neisseria meningitidis	NG	NG	NG	24	1	1	25	4	6	6	9	9	11	6	17	ST-41/44 complex	12219	4	4	2
44120	ST41456	UK [England]	2015	carrier	Neisseria meningitidis	NG	cnl	cnl	4	1	1	206	4	5	4	17	15	30	7	12	ST-198 complex	823	4	3	1
44145	ST41459	UK [England]	2015	carrier	Neisseria meningitidis	NG	Z	Z	22	1	1	1	4	20	3	2	26	26	15	112		5953	4	4	2
52688	ST41464	UK [England]	2015	carrier	Neisseria meningitidis	NG	L	L	13	3	57	145	3	57	5	2	35	6	68	20		963	3	3	1
52689	ST41465	UK [England]	2015	carrier	Neisseria meningitidis	B	B	B	16	1	1	59	4	3	6	9	5	9	679	9	ST-41/44 complex	10535	4	4	2
44154	ST41497	UK [England]	2015	carrier	Neisseria meningitidis	NG	E	E	19	1	1	65	4	175	5	484	17	3	4	124		6798	4	4	2
44153	ST41503	UK [England]	2015	carrier	Neisseria meningitidis	NG	cnl	cnl	102	8	10	2	20	16	2	6	25	17	25	22	ST-53 complex	53	20	4	2
44152	ST41520	UK [England]	2015	carrier	Neisseria meningitidis	NG	cnl	cnl	334	1	1	527	4	6	41	108	15	9	6	9		845	4	4	2
44151	ST41524	UK [England]	2015	carrier	Neisseria meningitidis	W	W	W	22	1	1	1	4	2	3	4	3	8	4	6	ST-11 complex	11	4	4	2
44150	ST41538	UK [England]	2015	carrier	Neisseria meningitidis	NG	B	B	13	3	57	145	3	4	10	34	5	38	11	9	ST-269 complex	1161	3	3	1
44148	ST41551	UK [England]	2015	carrier	Neisseria meningitidis	NG	cnl	cnl	4	1	1	206	4	5	4	17	15	30	7	12	ST-198 complex	823	4	3	1
44137	ST41552	UK [England]	2015	carrier	Neisseria meningitidis	NG	E	E	13	3	57	209	3	8	25	7	17	21	26	49	ST-1157 complex	1157	3	3	1
44146	ST41558	UK [England]	2015	carrier	Neisseria meningitidis	NG	B	B	19	1	1	65	4	9	6	9	9	9	64	9	ST-41/44 complex	409	4	4	2
44155	ST41559	UK [England]	2015	carrier	Neisseria meningitidis	NG	E	E	967	1	1	706	4	8	25	7	17	21	26	49	ST-1157 complex	1157	4	4	2
44144	ST41563	UK [England]	2015	carrier	Neisseria meningitidis	NG	cnl	cnl	21	1	1	7	4	16	2	159	92	77	25	112	ST-1117 complex	1117	4	4	2
50350	ST41570	UK [England]	2015	carrier	Neisseria meningitidis	NG	E	E	13	3	57	209	3	8	25	7	17	21	26	49	ST-1157 complex	1157	3	3	1
44142	ST41573	UK [England]	2015	carrier	Neisseria meningitidis	Y	Y	Y	25	1	1	26	4	10	5	18	9	11	9	17	ST-23 complex	23	4	4	2
50351	ST41575	UK [England]	2015	carrier	Neisseria meningitidis	Y	Y	Y	25	1	1	26	4	10	5	18	9	11	9	17	ST-23 complex	23	4	4	2
50352	ST41582	UK [England]	2015	carrier	Neisseria meningitidis	NG	NG	NG	24	1	1	25	4	6	6	9	9	11	6	17	ST-41/44 complex	12219	4	4	2
44227	ST41587	UK [England]	2015	carrier	Neisseria meningitidis	B	B	B	4	2	1	84	2	3	6	9	3	11	6	9	ST-41/44 complex	3818	2	3	1
50354	ST41605	UK [England]	2015	carrier	Neisseria meningitidis	Y	Y	Y	25	1	1	26	4	12	5	18	9	11	9	17	ST-23 complex	1655	4	4	2
52692	ST41612	UK [England]	2015	carrier	Neisseria meningitidis	B	B	B	45	8	1	87	20	7	5	1	627	36	53	15	ST-213 complex	9881	20	4	3
44106	ST41616	UK [England]	2015	carrier	Neisseria meningitidis	Y	Y	Y	25	1	1	26	4	10	5	314	9	11	9	17	ST-23 complex	3582	4	4	2
52693	ST41619	UK [England]	2015	carrier	Neisseria meningitidis	B	B	B	45	8	10	87	20	7	5	1	13	36	53	15	ST-213 complex	213	20	4	3
44223	ST41633	UK [England]	2015	carrier	Neisseria meningitidis	Y	Y	Y	25	1	1	26	4	12	5	18	9	11	9	17	ST-23 complex	1655	4	4	2
44233	ST41634	UK [England]	2015	carrier	Neisseria meningitidis	W	W	W	22	1	1	1	4	2	3	4	3	8	4	6	ST-11 complex	11	4	4	2
52694	ST41640	UK [England]	2015	carrier	Neisseria meningitidis	B	B	B	45	8	1	87	20	7	5	1	13	36	53	15	ST-213 complex	213	20	4	3
52695	ST41648	UK [England]	2015	carrier	Neisseria meningitidis	NG	B	B	16	1	4	59	4	4	10	11	18	3	10	12	ST-35 complex	1417	4	4	2
52696	ST41653	UK [England]	2015	carrier	Neisseria meningitidis	NG	NG	NG	24	1	1	25	4	6	6	9	9	11	6	17	ST-41/44 complex	12219	4	4	2
50356	ST41661	UK [England]	2015	carrier	Neisseria meningitidis	NG	W	W	22	1	1	1	4	2	3	4	3	8	4	6	ST-11 complex	11	4	4	2
50357	ST41663	UK [England]	2015	carrier	Neisseria meningitidis	NG	cnl	cnl	24	1	4	25	4	4	10	47	17	6	2	12	ST-35 complex	278	4	4	2
44117	ST41665	UK [England]	2015	carrier	Neisseria meningitidis	NG	Y	Y	25	1	1	26	4	10	5	18	9	11	9	17	ST-23 complex	23	4	4	2
52697	ST41668	UK [England]	2015	carrier	Neisseria meningitidis	B	B	B	45	61	1	87	192	7	5	1	13	36	53	15	ST-213 complex	213	NA	NA	3
52698	ST41671	UK [England]	2015	carrier	Neisseria meningitidis	NG	Y	Y	25	1	1	26	4	12	5	18	9	11	9	17	ST-23 complex	1655	4	4	2
50360	ST41696	UK [England]	2015	carrier	Neisseria meningitidis	Y	Y	Y	25	1	1	26	4	12	5	18	9	11	9	17	ST-23 complex	1655	4	4	2
60971	ST41710	UK [England]	2015	carrier	Neisseria meningitidis	Y	Y	Y	25	1	1	26	4	12	5	18	9	11	9	17	ST-23 complex	1655	4	4	2
50361	ST41713	UK [England]	2015	carrier	Neisseria meningitidis	NG	Y	Y	966	6	3	704	7	8	10	5	4	5	3	8	ST-32 complex	34	7	1	1
50105	ST41714	UK [England]	2015	carrier	Neisseria meningitidis	Y	Y	Y	25	1	1	26	4	10	5	116	9	11	9	17	ST-23 complex	10294	4	4	2
50362	ST41715	UK [England]	2015	carrier	Neisseria meningitidis	NG	cnl	cnl	102	8	10	2	20	16	2	6	25	17	25	22	ST-53 complex	53	20	4	2
50363	ST41716	UK [England]	2015	carrier	Neisseria meningitidis	NG	E	E	13	3	57	209	3	8	25	7	17	21	26	49	ST-1157 complex	1157	3	3	1
50364	ST41720	UK [England]	2015	carrier	Neisseria meningitidis	NG	W	W	22	1	1	1	4	2	3	4	3	8	4	6	ST-11 complex	11	4	4	2
50365	ST41732	UK [England]	2015	carrier	Neisseria meningitidis	NG	NG	NG	24	1	1	25	4	6	6	9	9	11	6	17	ST-41/44 complex	12219	4	4	2
50366	ST41742	UK [England]	2015	carrier	Neisseria meningitidis	NG	W	W	22	1	1	1	4	2	3	4	3	8	4	6	ST-11 complex	11	4	4	2
50367	ST41746	UK [England]	2015	carrier	Neisseria meningitidis	B	B	B	45	8	1	87	20	7	5	4	13	36	53	15	ST-213 complex	3722	20	4	3
50368	ST41750	UK [England]	2015	carrier	Neisseria meningitidis	NG	B	B	23	250	1	431	NA	8	5	4	17	8	21	2	ST-865 complex	12701	NA	NA	2
50369	ST41751	UK [England]	2015	carrier	Neisseria meningitidis	NG	cnl	cnl	4	1	1	206	4	5	4	17	15	30	7	12	ST-198 complex	823	4	3	1
50370	ST41758	UK [England]	2015	carrier	Neisseria meningitidis	NG	E	E	19	1	1	65	4	175	5	484	17	3	4	124		6798	4	4	2
50371	ST41772	UK [England]	2015	carrier	Neisseria meningitidis	NG	B	B	45	8	77	87	20	7	5	1	13	36	53	15	ST-213 complex	213	20	4	3
50372	ST41775	UK [England]	2015	carrier	Neisseria meningitidis	B	B	B	14	1	1	142	13	3	6	826	5	3	6	9	ST-41/44 complex	12702	13	3	1
50373	ST41777	UK [England]	2015	carrier	Neisseria meningitidis	NG	cnl	cnl	4	1	1	206	4	5	4	17	15	30	7	12	ST-198 complex	823	4	3	1
50374	ST41778	UK [England]	2015	carrier	Neisseria meningitidis	Y	Y	Y	25	1	1	26	4	12	5	18	9	11	9	820	ST-23 complex	12703	4	4	2
50375	ST41780	UK [England]	2015	carrier	Neisseria meningitidis	NG	C	C	21	1	1	7	4	1	5	13	53	26	41	3	ST-162 complex	162	4	4	2
50376	ST41785	UK [England]	2015	carrier	Neisseria meningitidis	NG	cnl	cnl	21	1	1	7	4	16	2	159	92	77	25	112	ST-1117 complex	1117	4	4	2
50377	ST41810	UK [England]	2015	carrier	Neisseria meningitidis	NG	cnl	cnl	30	17	1	54	37	3	6	9	5	9	6	9	ST-41/44 complex	41	NA	NA	3
50378	ST41812	UK [England]	2015	carrier	Neisseria meningitidis	NG	cnl	cnl	14	7	1	142	6	3	6	9	5	9	6	9	ST-41/44 complex	41	6	2	1
50379	ST41820	UK [England]	2015	carrier	Neisseria meningitidis	B	B	B	45	8	77	87	20	7	5	1	13	36	53	15	ST-213 complex	213	20	4	3
50380	ST41823	UK [England]	2015	carrier	Neisseria meningitidis	W	W	W	16	1	1	60	4	11	5	18	8	78	24	21	ST-22 complex	1224	4	4	2
50381	ST41824	UK [England]	2015	carrier	Neisseria meningitidis	NG	cnl	cnl	102	8	10	2	20	16	2	6	25	17	25	22	ST-53 complex	53	20	4	2

50382	ST41825	UK [England]	2015	carrier	Neisseria meningitidis	Y	Y	Y	25	1	1	26	4	10	5	18	9	11	9	17	ST-23 complex	23	4	4	2
50383	ST41830	UK [England]	2015	carrier	Neisseria meningitidis	NG	Y	Y	25	1	1	26	4	12	5	18	9	11	9	17	ST-23 complex	1655	4	4	2
50384	ST41854	UK [England]	2015	carrier	Neisseria meningitidis	NG	E	E	13	3	1	145	3	17	4	19	17	3	26	2	ST-60 complex	4146	3	3	1
50385	ST41856	UK [England]	2015	carrier	Neisseria meningitidis	NG	B	B	226	66	4	228	38	4	10	11	18	6	10	12	ST-35 complex	35	NA	NA	1
50386	ST41860	UK [England]	2015	carrier	Neisseria meningitidis	NG	cnl	cnl	21	1	1	7	4	16	2	159	92	77	25	112	ST-1117 complex	1117	4	4	2
52701	ST41868	UK [England]	2015	carrier	Neisseria meningitidis	NG	NG	NG	24	1	1	25	4	6	6	9	9	11	6	17	ST-41/44 complex	12219	4	4	2
50388	ST41870	UK [England]	2015	carrier	Neisseria meningitidis	Y	Y	Y	25	1	1	26	4	12	5	18	9	11	9	17	ST-23 complex	1655	4	4	2
50389	ST41876	UK [England]	2015	carrier	Neisseria meningitidis	NG	cnl	cnl	102	8	10	2	20	16	2	6	25	17	25	22	ST-53 complex	53	20	4	2
50390	ST41878	UK [England]	2015	carrier	Neisseria meningitidis	NG	cnl	cnl	21	1	1	7	4	16	2	159	92	77	25	112	ST-1117 complex	1117	4	4	2
50391	ST41884	UK [England]	2015	carrier	Neisseria meningitidis	NG	Y	Y	13	3	57	209	3	8	25	7	17	21	26	49	ST-1157 complex	1157	3	3	1
50392	ST41887	UK [England]	2015	carrier	Neisseria meningitidis	Y	Y	Y	25	1	1	26	4	12	5	18	9	11	9	17	ST-23 complex	1655	4	4	2
50393	ST41896	UK [England]	2015	carrier	Neisseria meningitidis	NG	cnl	cnl	102	8	10	2	20	16	2	6	25	17	25	22	ST-53 complex	53	20	4	2
50394	ST41910	UK [England]	2015	carrier	Neisseria meningitidis	NG	Z	Z	13	3	1	209	3	8	25	7	17	21	26	49	ST-1157 complex	1157	3	3	1
50395	ST41914	UK [England]	2015	carrier	Neisseria meningitidis	NG	E	E	13	3	57	209	3	8	553	7	17	21	26	49	ST-1157 complex	12704	3	3	1
50396	ST41934	UK [England]	2015	carrier	Neisseria meningitidis	NG	Y	Y	25	1	1	26	4	12	5	18	9	11	9	17	ST-23 complex	1655	4	4	2
50397	ST41936	UK [England]	2015	carrier	Neisseria meningitidis	NG	E	E	13	3	1	145	3	17	5	19	17	3	26	2	ST-60 complex	60	3	3	1
50398	ST41951	UK [England]	2015	carrier	Neisseria meningitidis	NG	Y	Y	147	195	1	708	NA	12	5	18	9	11	9	17	ST-23 complex	1655	NA	NA	2
44240	ST41959	UK [England]	2015	carrier	Neisseria meningitidis	NG	Y	Y	25	1	1	26	4	12	5	18	9	11	9	17	ST-23 complex	1655	4	4	2
50399	ST41963	UK [England]	2015	carrier	Neisseria meningitidis	NG	NG	NG	24	1	1	25	4	6	6	9	9	11	6	17	ST-41/44 complex	12219	4	4	2
50400	ST41988	UK [England]	2015	carrier	Neisseria meningitidis	NG	cnl	cnl	102	8	10	2	20	16	2	6	25	17	25	22	ST-53 complex	53	20	4	2
44236	ST41994	UK [England]	2015	carrier	Neisseria meningitidis	NG	Y	Y	25	1	1	26	4	12	5	18	9	11	9	17	ST-23 complex	1655	4	4	2
50401	ST41997	UK [England]	2015	carrier	Neisseria meningitidis	NG	cnl	cnl	4	1	1	206	4	5	4	17	15	30	7	12	ST-198 complex	823	4	3	1
50402	ST42003	UK [England]	2015	carrier	Neisseria meningitidis	W	W	W	16	1	1	60	4	11	5	18	8	11	26	21	ST-22 complex	1686	4	4	2
50403	ST42016	UK [England]	2015	carrier	Neisseria meningitidis	NG	cnl	cnl	21	1	1	7	4	1	5	13	53	26	41	3	ST-162 complex	162	4	4	2
50404	ST42017	UK [England]	2015	carrier	Neisseria meningitidis	Y	Y	Y	25	1	1	26	4	12	5	18	9	11	798	17	ST-23 complex	12353	4	4	2
50405	ST42025	UK [England]	2015	carrier	Neisseria meningitidis	NG	B	B	45	8	77	87	20	7	5	1	13	36	53	15	ST-213 complex	213	20	4	3
50406	ST42032	UK [England]	2015	carrier	Neisseria meningitidis	W	W	W	22	1	1	1	4	662	3	4	3	8	4	6	ST-11 complex	10651	4	4	2
50407	ST42036	UK [England]	2015	carrier	Neisseria meningitidis	NG	Y	Y	25	1	1	26	4	12	5	18	9	11	9	17	ST-23 complex	1655	4	4	2
50408	ST42038	UK [England]	2015	carrier	Neisseria meningitidis	NG	cnl	cnl	334	1	1	527	4	6	41	108	15	9	6	9		845	4	4	2
52702	ST42061	UK [England]	2015	carrier	Neisseria meningitidis	NG	C	C	15	4	2	193	1	781	10	15	9	8	11	13	ST-269 complex	12705	1	1	1
50410	ST42073	UK [England]	2015	carrier	Neisseria meningitidis	NG	C	C	15	4	2	193	1	781	10	15	9	8	11	13	ST-269 complex	12705	1	1	1
50411	ST42087	UK [England]	2015	carrier	Neisseria meningitidis	Y	Y	Y	25	1	1	26	4	12	5	18	9	11	9	17	ST-23 complex	1655	4	4	2
50412	ST42103	UK [England]	2015	carrier	Neisseria meningitidis	NG	NG	NG	0	4	2	921	1	4	10	15	9	8	11	9	ST-269 complex	269	1	1	NA
50413	ST42117	UK [England]	2015	carrier	Neisseria meningitidis	Y	Y	Y	25	1	1	26	4	12	5	18	9	11	9	17	ST-23 complex	1655	4	4	2
50414	ST42119	UK [England]	2015	carrier	Neisseria meningitidis	NG	cnl	cnl	111	182	77	37	NA	35	24	15	48	6	48	41	ST-192 complex	192	NA	NA	3
44258	ST42154	UK [England]	2015	carrier	Neisseria meningitidis	B	B	B	19	1	1	65	4	9	20	9	9	9	6	2	ST-41/44 complex	1097	4	4	2
50415	ST42156	UK [England]	2015	carrier	Neisseria meningitidis	NG	cnl	cnl	4	1	1	206	4	5	4	17	15	30	7	12	ST-198 complex	823	4	3	1
50416	ST42192	UK [England]	2015	carrier	Neisseria meningitidis	NG	E	E	13	3	57	209	3	8	25	7	17	21	26	49	ST-1157 complex	1157	3	3	1
50417	ST42203	UK [England]	2015	carrier	Neisseria meningitidis	NG	cnl	cnl	102	8	10	2	20	16	2	6	25	17	25	22	ST-53 complex	53	20	4	2
50418	ST42215	UK [England]	2015	carrier	Neisseria meningitidis	NG	E	E	13	3	57	209	3	8	25	7	17	21	26	49	ST-1157 complex	1157	3	3	1
50419	ST42227	UK [England]	2015	carrier	Neisseria meningitidis	NG	cnl	cnl	4	1	1	206	4	5	4	17	15	30	7	12	ST-198 complex	823	4	3	1
50420	ST42231	UK [England]	2015	carrier	Neisseria meningitidis	W	W	W	22	1	1	1	4	2	3	4	3	8	4	6	ST-11 complex	11	4	4	2
50421	ST42235	UK [England]	2015	carrier	Neisseria meningitidis	NG	NG	NG	16	1	4	59	4	4	10	11	18	6	10	17	ST-35 complex	8593	4	4	2
52705	ST42251	UK [England]	2015	carrier	Neisseria meningitidis	NG	NG	NG	622	4	2	190	1	4	10	15	9	8	11	79	ST-269 complex	9823	1	1	1
50423	ST42258	UK [England]	2015	carrier	Neisseria meningitidis	NG	B	B	16	1	2	60	4	222	3	58	261	263	5	255	ST-4821 complex	3200	4	4	2
50424	ST42261	UK [England]	2015	carrier	Neisseria meningitidis	NG	cnl	cnl	102	8	10	2	20	16	2	6	25	17	25	22	ST-53 complex	53	20	4	2
50425	ST42262	UK [England]	2015	carrier	Neisseria meningitidis	NG	E	E	499	15	121	134	58	8	25	827	17	21	26	49	ST-1157 complex	12354	NA	NA	3
50426	ST42264	UK [England]	2015	carrier	Neisseria meningitidis	NG	B	B	45	8	77	87	20	7	5	1	13	36	53	15	ST-213 complex	213	20	4	3
50427	ST42267	UK [England]	2015	carrier	Neisseria meningitidis	NG	E	E	13	3	1	145	3	17	5	19	17	3	26	2	ST-60 complex	60	3	3	1
50428	ST42268	UK [England]	2015	carrier	Neisseria meningitidis	B	B	B	24	1	4	25	4	4	10	47	17	6	2	12	ST-35 complex	278	4	4	2
50429	ST42270	UK [England]	2015	carrier	Neisseria meningitidis	B	B	B	45	8	77	87	20	7	5	1	13	36	53	15	ST-213 complex	213	20	4	3
50430	ST42275	UK [England]	2015	carrier	Neisseria meningitidis	NG	B	B	16	1	2	60	4	222	3	58	261	263	5	255	ST-4821 complex	3200	4	4	2
50431	ST42288	UK [England]	2015	carrier	Neisseria meningitidis	NG	cnl	cnl	4	1	1	206	4	5	4	17	15	30	7	12	ST-198 complex	823	4	3	1
50432	ST42303	UK [England]	2015	carrier	Neisseria meningitidis	NG	cnl	cnl	4	1	1	206	4	5	4	828	15	30	7	12	ST-198 complex	12706	4	3	1
50433	ST42305	UK [England]	2015	carrier	Neisseria meningitidis	NG	NG	NG	683	4	2	176	1	4	10	15	9	8	11	9	ST-269 complex	269	1	1	2
50434	ST42315	UK [England]	2015	carrier	Neisseria meningitidis	W	W	W	22	1	1	1	4	2	3	4	3	8	4	6	ST-11 complex	11	4	4	2
50435	ST42319	UK [England]	2015	carrier	Neisseria meningitidis	NG	cnl	cnl	102	8	10	2	20	16	2	6	25	17	25	22	ST-53 complex	53	20	4	2
50436	ST42324	UK [England]	2015	carrier	Neisseria meningitidis	NG	cnl	cnl	968	157	56	707	NA	5	4	17	15	14	7	12	ST-198 complex	198	NA	NA	3
50437	ST42326	UK [England]	2015	carrier	Neisseria meningitidis	Y	Y	Y	25	1	1	26	4	10	5	18	9	11	9	17	ST-23 complex	23	4	4	2
44224	ST42333	UK [England]	2015	carrier	Neisseria meningitidis	NG	E	E	13	3	1	209	3	8	25	827	17	21	26	49	ST-1157 complex	12354	3	3	1
52707	ST42397	UK [England]	2015	carrier	Neisseria meningitidis	NG	B	B	4	1	59	84	4	4	10	11	17	6	10	12	ST-35 complex	457	4	3	1
44231	ST42420	UK [England]	2015	carrier	Neisseria meningitidis	NG	cnl	cnl	102	8	10	2	20	16	2	6	25	17	25	22	ST-53 complex	53	20	4	2
50109	WG40004	UK [England]	2015	carrier	Neisseria meningitidis	NG	E	E	13	3	NA	145	3	17	4	19	17	3	26	2	ST-60 complex	4146	3	3	1
50110	WG40008	UK [England]	2015	carrier	Neisseria meningitidis	B	B	B	19	1	1	65	4	9	20	9	9	9	6	2	ST-41/44 complex	1097	4	4	2
50111	WG40014	UK [England]	2015	carrier	Neisseria meningitidis	Y	Y	Y	21	1	1	7	4	6	5	173	13	5	24	17	ST-174 complex	1466	4	4	2
50112	WG40017	UK [England]	2015	carrier	Neisseria meningitidis	NG	Y	Y	21	1	1	7	4	6	5	173	13	5	24	17	ST-174 complex	1466	4	4	2
50113	WG40022	UK [England]	2015	carrier	Neisseria meningitidis	NG	NG	NG	13	3	1	145	3	17	5	19	17	3	26	2	ST-60 complex	60	3	3	1
50114	WG40023	UK [England]	2015	carrier	Neisseria meningitidis	Y	Y	Y	25	1	1	26	4	12	5	18	9	11	9	17	ST-23 complex	1655	4	4	2
50115	WG40024	UK [England]	2015	carrier	Neisseria meningitidis	NG	B	B	13	3	57	209	3	8	25	7	17	21	26	49	ST-1157 complex	1157	3	3	1
50117	WG40035	UK [England]	2015	carrier	Neisseria meningitidis	NG	cnl	cnl	102	8	10	2	20	16	2	6	25	17	25	22	ST-53 complex	53	20	4	2
50118	WG40040	UK [England]	2015	carrier	Neisseria meningitidis	NG	Y	Y	25	1	1	26	4	10	5	18	9	11	9	17	ST-23 complex	23	4	4	2
50119	WG40041	UK [England]	2015	carrier	Neisseria meningitidis	NG	E	E	13	3	1	145	3	17	5	19	17	3	6	2	ST-60 complex	1677	3	3	1
50120	WG40047	UK [England]	2015	carrier	Neisseria meningitidis	NG	X	X	13	3	57	209	3	8	5	7	5	21	26	9	ST-1157 complex	12695	3	3	1
50438	WG40056	UK [England]	2015	carrier	Neisseria meningitidis	Y	Y	Y	25	1	1	26	4	10	5	18	9	11	9	17	ST-23 complex	23	4	4	2
52709	WG40058	UK [England]	2015	carrier	Neisseria meningitidis	B	B	B	13	3	57	209	3	12	5	12	35	192	22	17	ST-461 complex	1946	3	3	1
52710	WG40060	UK [England]	2015	carrier	Neisseria meningitidis	B	B	B	19	1	1	65	4	3	6	9	5	9	6	70	ST-41/44 complex	414	4	4	2
50121	WG40065	UK [England]	2015	carrier	Neisseria meningitidis	NG	cnl	cnl	4	1	1	206	4	5	4	17	15	30	7	12	ST-198 complex	823	4	3	1
50122	WG40619	UK [England]	2015	carrier	Neisseria meningitidis	NG	cnl	cnl	102	8	10	2	20	16	2	6	25	17	25	17	ST-53 complex	12854	20	4	2
50123	WG40625	UK [England]	2015	carrier	Neisseria meningitidis	NG	cnl	cnl	102	8	10	2	20	16	2	6	25	17	25	22	ST-53 complex	53	20	4	2
50124	WG40634	UK [England]	2015	carrier	Neisseria meningitidis	NG	B	B	13	3	59	145	3	8	5	15	17	8	21	2	ST-865 complex	865	3	3	1
50125	WG40639	UK [England]	2015	carrier	Neisseria meningitidis	NG	cnl	cnl	985	8	10	787	20	16	2	6	25	17	25	22	ST-53 complex	53	20	4	2
50126	WG40658	UK [England]	2015	carrier	Neisseria meningitidis	W	W	W	22	1	1	1	4	2	3	4	3	8	4	6	ST-11 complex	11	4	4	2
50127	WG40661	UK [England]	2015	carrier	Neisseria meningitidis	NG	cnl	cnl	102	8	10	2	20	16	2	6	25	17	25	22	ST-53 complex	53	20	4	2
50128	WG40681	UK [England]	2015	carrier	Neisseria meningitidis	NG	B	B	19	1	1	65	4	110	2	15	5	26	191	46		2130	4	4	2
52712	WG40739	UK [England]	2015	carrier	Neisseria meningitidis	B	B	B	986	8	1	786	20	7	587	1	13	36	53	15	ST-213 complex	12696	20	4	3
50130	WG40743	UK [England]	2015	carrier	Neisseria meningitidis	W	W	W	22	1	1	1	4	2	3	4	3	8	4	6	ST-11 complex	11	4	4	2
50131	WG40747	UK [England]	2015	carrier	Neisseria meningitidis	NG	X	X	13	3	1	209	3	8	25	7	17	15	26	49	ST-1157 complex	12073	3	3	1
50132	WG40753	UK [England]	2015	carrier	Neisseria meningitidis	NG	E	E	13	3	NA	145	3	17	4	19	17	3	26	2	ST-60 complex	4146	3	3	1
52713	WG40761	UK [England]	2015	carrier	Neisseria meningitidis	NG	Z	Z	22	1	1	1	4	20	3	2	26	26	15	9		3882	4	4	2
50135	WG40768	UK [England]	2015	carrier	Neisseria meningitidis	NG	cnl	cnl	21	1	1	7	4	16	2	159	92	77	25	112	ST-1117 complex	1117	4	4	2
50136	WG40811	UK [England]	2015	carrier	Neisseria meningitidis	W	W	W	22	1	1	1	4	2	3	4	3	8	4	6	ST-11 complex	11	4	4	2
50137	WG40820	UK [England]	2015	carrier	Neisseria meningitidis	W	W	W	22	1	1	1	4	2	3	4	3	8	4	6	ST-11 complex	11	4	4	2
50138	WG40823	UK [England]	2015	carrier	Neisseria meningitidis	NG	Y	Y	25	1	1	26	4	12	5	18	9	11	9	17	ST-23 complex	1655	4	4	2
50139	WG40825	UK [England]	2015	carrier	Neisseria meningitidis	Y	Y	Y	218	24	101	784	115	10	5	18	9	11	9	17	ST-23 complex	23	NA	NA	1
50140	WG40834	UK [England]	2015	carrier	Neisseria meningitidis	NG	cnl	cnl	4	1	1	206	4	5	4	17	15	30	7	12	ST-198 complex	823	4	3	1
50141	WG40840	UK [England]	2015	carrier	Neisseria meningitidis	Y	Y	Y	25	1	1	26	4	12	5	18	332	11	9	17	ST-23 complex	11280	4	4	2
52714	WG40850	UK [England]	2015	carrier	Neisseria meningitidis	NG	B	B	47	1	57	90	15	12	5	12	35	192	22	17	ST-461 complex	1946	15	4	3
50143	WG40854	UK [England]	2015	carrier	Neisseria meningitidis	NG	cnl	cnl	21	1	1	7	4	16	2	159	92	77	25	112	ST-1117 complex	1117	4	4	2
50144	WG40855	UK [England]	2015	carrier	Neisseria meningitidis	NG	Z	Z	25	1	1	26	4	430	4	6	17	5	18	2	ST-103 complex	12585	4	4	2
50145	WG40868	UK [England]	2015	carrier	Neisseria meningitidis	NG	cnl	cnl	102	8	10	2	20	16	2	6	25	17	25	22	ST-53 complex	53	20	4	2
52715	WG40897	UK [England]	2015	carrier	Neisseria meningitidis	NG	NG	NG	151	1	1	130	4	6	7	4	56	26	18	8	ST-175 complex	175	4	4	2
50148	WG40899	UK [England]	2015	carrier	Neisseria meningitidis	NG	B	B	45	8	1	87	20	7	5	1	627	36	53	15	ST-213 complex	9881	20	4	3
50149	WG40906	UK [England]	2015	carrier	Neisseria meningitidis	NG	E	E	13	3	57	209	3	8	25	7	17	21	26	49	ST-1157 complex	1157	3	3	1
52716	WG40912	UK [England]	2015	carrier	Neisseria meningitidis	NG	B	B	19	1	1	65	4	110	2	15	5	26	191	46		2130	4	4	2
50152	WG40923	UK [England]	2015	carrier	Neisseria meningitidis	W	W	W	16	1	1	60	4	11	5	18	8	11	4	21	ST-22 complex	184	4	4	2
50153	WG40930	UK [England]	2015	carrier	Neisseria meningitidis	B	B	B	19	1	1	65	4	110	2	15	5	26	191	46		2130	4	4	2
52717	WG40932	UK [England]	2015	carrier	Neisseria meningitidis	W	W	W	22	1	1	1	4	2	3	4	3	8	4	6	ST-11 complex	11	4	4	2
52718	WG40933	UK [England]	2015	carrier	Neisseria meningitidis	NG	B	B	45	8	77	87	20	7	5	1	13	36	53	15	ST-213 complex	213	20	4	3
52719	WG40939	UK [England]	2015	carrier	Neisseria meningitidis	NG	W	W	22	1	1	1	4	2	3	4	3	8	4	6	ST-11 complex	11	4	4	2
50157	WG40949	UK [England]	2015	carrier	Neisseria meningitidis	NG	B	B	13	3	57	145	3	8	105	52	3	9	11	7		1572	3	3	1
50158	WG40952	UK [England]	2015	carrier	Neisseria meningitidis	NG	Z	Z	16	56	79	60	NA	13	29	2	17	26	65	53		2123	NA	NA	2
50159	WG40976	UK [England]	2015	carrier	Neisseria meningitidis	NG	E	E	13	3	NA	145	3	17	4	19	17	3	26	2	ST-60 complex	4146	3	3	1
52720	WG40990	UK [England]	2015	carrier	Neisseria meningitidis	NG	B	B	45	8	77	87	20	7	5	1	13	36	53	15	ST-213 complex	213	20	4	3
50161	WG41001	UK [England]	2015	carrier	Neisseria meningitidis	NG	cnl	cnl	102	8	10	2	20	16	2	18	25	17	25	22	ST-53 complex	2441	20	4	2
50162	WG41017	UK [England]	2015	carrier	Neisseria meningitidis	NG	E	E	13	3	1	209	3	8	25	7	17	21	26	49	ST-1157 complex	1157	3	3	1
52721	WG41026	UK [England]	2015	carrier	Neisseria meningitidis	B	B	B	19	1	1	83	4	4	10	2	201	38	11	9	ST-269 complex	6791	4	4	2
50164	WG41027	UK [England]	2015	carrier	Neisseria meningitidis	NG	E	E	13	3	57	209	3	8	25	7	17	21	26	49	ST-1157 complex	1157	3	3	1
50165	WG41032	UK [England]	2015	carrier	Neisseria meningitidis	W	W	W	22	1	1	1	4	2	3	4	3	8	4	6	ST-11 complex	11	4	4	2
52722	WG41050	UK [England]	2015	carrier	Neisseria meningitidis	NG	Y	Y	1057	NA	1	920	NA	12	5	18	9	11	9	17	ST-23 complex	1655	NA	NA	1
50167	WG41053	UK [England]	2015	carrier	Neisseria meningitidis	NG	Z	Z	25	1	1	26	4	8	4	34	17	194	18	2	ST-103 complex	8023	4	4	2
52724	WG41062	UK [England]	2015	carrier	Neisseria meningitidis	NG	B	B	510	28	8	599	30	4	10	48	4	6	3	8	ST-32 complex	7460	NA	NA	1
50170	WG41068	UK [England]	2015	carrier	Neisseria meningitidis	Y	Y	Y	25	1	1	26	4	12	5	18	9	11	9	17	ST-23 complex	1655	4	4	2
50171	WG41073	UK [England]	2015	carrier	Neisseria meningitidis	NG	B	B	16	1	1	60	4	4	10	11	8	6	10	12	ST-35 complex	809	4	4	2
50172	WG41079	UK [England]	2015	carrier	Neisseria meningitidis	Y	Y	Y	25	1	1	26	4	12	5	18	9	11	9	17	ST-23 complex	1655	4	4	2
50173	WG41082	UK [England]	2015	carrier	Neisseria meningitidis	Y	Y	Y	25	1	1	26	4	12	5	18	9	11	9	17	ST-23 complex	1655	4	4	2
50174	WG41085	UK [England]	2015	carrier	Neisseria meningitidis	B	B	B	202	1	1	35	4	42	26	46	24	2	20	17	ST-282 complex	282	4	4	2
52725	WG41087	UK [England]	2015	carrier	Neisseria meningitidis	Y	Y	Y	25	1	1	26	4	10	5	18	9	11	9	17	ST-23 complex	23	4	4	2
50176	WG41091	UK [England]	2015	carrier	Neisseria meningitidis	B	B	B	420	26	4	790	25	4	10	11	18	6	10	12	ST-35 complex	35	NA	NA	1
50439	WG41094	UK [England]	2015	carrier	Neisseria meningitidis	B	B	B	49	1	57	102	13	4	10	5	4	6	3	8	ST-32 complex	32	13	4	2
50179	WG41099	UK [England]	2015	carrier	Neisseria meningitidis	NG	Y	Y	25	1	1	26	4	10	5	116	9	11	9	17	ST-23 complex	10294	4	4	2
50180	WG41105	UK [England]	2015	carrier	Neisseria meningitidis	Y	Y	Y	13	3	57	209	3	12	5	18	332	11	9	17	ST-23 complex	11280	3	3	1
52726	WG41108	UK [England]	2015	carrier	Neisseria meningitidis	NG	NG	NG	25	1	1	26	4	10	5	18	9	11	9	17	ST-23 complex	23	4	4	2
50182	WG41112	UK [England]	2015	carrier	Neisseria meningitidis	Y	Y	Y	25	1	1	26	4	12	5	18	9	11	9	17	ST-23 complex	1655	4	4	2
50183	WG41116	UK [England]	2015	carrier	Neisseria meningitidis	NG	E	E	13	3	57	209	3	8	25	7	17	21	26	49	ST-1157 complex	1157	3	3	1
50184	WG41120	UK [England]	2015	carrier	Neisseria meningitidis	NG	E	E	13	3	57	209	3	8	25	7	17	21	813	49	ST-1157 complex	12580	3	3	1
50185	WG41121	UK [England]	2015	carrier	Neisseria meningitidis	Y	Y	Y	25	1	1	26	4	12	5	18	9	11	9	17	ST-23 complex	1655	4	4	2
50186	WG41133	UK [England]	2015	carrier	Neisseria meningitidis	W	W	W	22	1	1	1	4	2	3	4	3	8	4	6	ST-11 complex	11	4	4	2
50187	WG41261	UK [England]	2015	carrier	Neisseria meningitidis	NG	cnl	cnl	94	58	56	89	NA	5	4	38	15	22	40	13	ST-1136 complex	1136	NA	NA	3

50188	WG41274	UK [England]	2015	carrier	Neisseria meningitidis	NG	X	X	13	3	1	209	3	8	25	7	17	15	26	49	ST-1157 complex	12073	3	3	1
50189	WG41279	UK [England]	2015	carrier	Neisseria meningitidis	Y	Y	Y	25	1	1	26	4	12	5	18	9	11	9	17	ST-23 complex	1655	4	4	2
50190	WG41297	UK [England]	2015	carrier	Neisseria meningitidis	Y	Y	Y	104	1	57	10	4	10	5	18	59	11	9	17	ST-23 complex	183	4	4	2
50191	WG41299	UK [England]	2015	carrier	Neisseria meningitidis	NG	cnl	cnl	102	8	10	2	20	16	2	6	25	17	25	22	ST-53 complex	53	20	4	2
50192	WG41302	UK [England]	2015	carrier	Neisseria meningitidis	NG	E	E	13	3	57	209	3	8	25	1	826	21	26	49	ST-1157 complex	12582	3	3	1
50195	WG41314	UK [England]	2015	carrier	Neisseria meningitidis	NG	E	E	125	1	57	268	13	7	16	55	10	3	56	13	ST-178 complex	2394	13	3	1
50196	WG41317	UK [England]	2015	carrier	Neisseria meningitidis	NG	B	B	45	8	77	87	20	7	5	1	13	36	53	15	ST-213 complex	213	20	4	3
52727	WG41318	UK [England]	2015	carrier	Neisseria meningitidis	NG	B	B	152	12	1	113	28	6	6	63	9	9	11	9	ST-41/44 complex	839	NA	NA	3
50198	WG41319	UK [England]	2015	carrier	Neisseria meningitidis	B	B	B	19	1	2	65	4	6	6	63	9	9	11	9	ST-41/44 complex	839	4	4	2
50199	WG41330	UK [England]	2015	carrier	Neisseria meningitidis	W	W	W	22	1	1	1	4	2	3	4	3	8	4	6	ST-11 complex	11	4	4	2
50201	WG41340	UK [England]	2015	carrier	Neisseria meningitidis	Y	Y	Y	25	1	1	26	4	12	5	18	9	11	9	17	ST-23 complex	1655	4	4	2
52728	WG41345	UK [England]	2015	carrier	Neisseria meningitidis	NG	B	B	45	8	77	87	20	7	5	844	13	36	53	15	ST-213 complex	12581	20	4	3
50203	WG41352	UK [England]	2015	carrier	Neisseria meningitidis	NG	E	E	13	3	57	209	3	8	25	7	17	21	26	49	ST-1157 complex	1157	3	3	1
50204	WG41359	UK [England]	2015	carrier	Neisseria meningitidis	NG	E	E	13	3	57	209	3	8	25	1	826	21	26	49	ST-1157 complex	12582	3	3	1
50205	WG41360	UK [England]	2015	carrier	Neisseria meningitidis	NG	Y	Y	25	1	1	26	4	12	5	18	9	11	9	17	ST-23 complex	1655	4	4	2
50206	WG41361	UK [England]	2015	carrier	Neisseria meningitidis	NG	B	B	13	3	1	209	3	8	25	7	5	21	26	49	ST-1157 complex	12583	3	3	1
50208	WG41367	UK [England]	2015	carrier	Neisseria meningitidis	NG	cnl	cnl	102	8	10	2	20	16	2	6	25	17	25	22	ST-53 complex	53	20	4	2
50209	WG41368	UK [England]	2015	carrier	Neisseria meningitidis	W	W	W	16	1	1	60	4	8	5	18	8	11	4	21	ST-22 complex	3849	4	4	2
50210	WG41371	UK [England]	2015	carrier	Neisseria meningitidis	Y	Y	Y	25	1	1	26	4	12	5	18	9	11	9	17	ST-23 complex	1655	4	4	2
50211	WG41374	UK [England]	2015	carrier	Neisseria meningitidis	NG	cnl	cnl	102	8	10	2	20	16	2	6	25	17	25	22	ST-53 complex	53	20	4	2
50212	WG41375	UK [England]	2015	carrier	Neisseria meningitidis	NG	E	E	13	3	57	209	3	8	25	7	17	21	26	49	ST-1157 complex	1157	3	3	1
50213	WG41382	UK [England]	2015	carrier	Neisseria meningitidis	Y	Y	Y	21	1	1	7	4	6	5	173	13	5	24	17	ST-174 complex	1466	4	4	2
50214	WG41394	UK [England]	2015	carrier	Neisseria meningitidis	NG	B	B	14	7	1	142	6	3	6	9	5	9	6	9	ST-41/44 complex	41	6	2	1
50215	WG41396	UK [England]	2015	carrier	Neisseria meningitidis	NG	cnl	cnl	94	157	56	69	NA	5	4	17	15	14	7	12	ST-198 complex	198	NA	NA	3
50216	WG41414	UK [England]	2015	carrier	Neisseria meningitidis	NG	E	E	13	3	57	209	3	8	25	7	17	21	26	842	ST-1157 complex	12584	3	3	1
