# Supplementary material for: Deconvolution of intergenic polymorphisms determining high expression of Factor H binding protein in meningococcus and their association with invasive disease
Source: PLoS Pathog. 2021 Mar 26;17(3):e1009461. doi: 10.1371/journal.ppat.1009461 (PMC8026042; doi:10.1371/journal.ppat.1009461)
Supplement: S7 Table — The absolute number of isolates in each group is reported along with the percentages for each expression group. (DOCX) [file ppat.1009461.s014.docx]

**S7 Table. Summary of carrier and invasive isolates for each expression group in the public UK dataset.**

|  | High | Medium | Low var1 | Low var2/3 |
| --- | --- | --- | --- | --- |
| carrier | 43 (11%) | 45 (11%) | 567 (40%) | 1484 (51%) |
| invasive | 349 (89%) | 372 (89%) | 850 (60%) | 1406 (49%) |
| Total | 392 | 417 | 1417 | 2890 |

The absolute number of isolates in each group is reported along with the percentages for each expression group.
