# Supplementary material for: Deconvolution of intergenic polymorphisms determining high expression of Factor H binding protein in meningococcus and their association with invasive disease
Source: PLoS Pathog. 2021 Mar 26;17(3):e1009461. doi: 10.1371/journal.ppat.1009461 (PMC8026042; doi:10.1371/journal.ppat.1009461)
Supplement: Attachment — Submitted filename: PPATHOGENS-D-20-02160 review (1).docx [file ppat.1009461.s015.docx]

The manuscript by Spinsanti et al is a detailed characterization of the intergenic region between *cbb*A and *fHbp* (designated fIR) mediating fHbp expression in *Neisseria meningitidis*. The authors defined 11 fIR sequence alleles, representing 91% of ~1100 meningococcal strains of UK circulating isolates. The authors also examined transcription by qRT-PCR, and protein levels by SRM-MS and Western blots. Further, polymorphisms within the Rho-independent terminator, -10 and -35 elements and its spacer as well as coding and the reported RNA thermosensor region were studied by site-specific mutagenesis using an isogenic MC58 strain background. The effects of fHbp peptide allele sequences on overall protein levels were also examined under the same inducible Ptac promoter control in MC58 by SRM-MS. The abundance of fHbp divided into four expression groups was correlated with susceptibility to killing by anti-fHbp antibodies and the ability to survive within human serum. The authors reported that strains with higher expressing fIR alleles and other cis factors contributing to high fHbp expression are more likely associated with invasive disease. The distributions of fIRs of >1100 strains were analyzed in terms of clonal complex, 3 fHbp protein variants, and fHbp peptide alleles.

This detailed comprehensive study is an extension of a previous 2016 study by Biagini et al from the same group. The previously defined 8 promoter clades are further refined to the 11 fIR sequence alleles. Many aspects of the *cis* regulation and a large collection of isolates were investigated to provide a better understanding of fHbp expression control, especially by the fIR sequence polymorphisms. While the data presented are quite comprehensive they do require greater clarity (see specific comment below). There is also another report by Cayrou et al. (ref #33) that applied analogous approaches to cluster the intergenic region to predict fHbp expression patterns in meningococci and a comparison of the conclusions from these two studies should be discussed.

The use of the nucleotide sequence of the *fHbp* intergenic region as a predictive tool remains speculative. As the data generated show, fHbp regulation is complex, including the read-through from upstream *cbb*A expression, which has its own regulation profile, the presence or absence of RNA thermoregulation and the regulation of transcriptional regulators (FNR and FUR) and their interactions with fIR. While it follows that that increased levels of fHbp expression are associated with increased serum resistance and will be seen in invasive isolates, not clear that the small SNP changes in the fIR alone will provide a clear prediction *a priori* on overall fHbp production of a particular isolate within a diverse meningococcal population.

Specific comments:

The polymorphisms depicted in Fig 1D are from the outliners plotted in 1C and are specific to individual representative allele in each group in Fig 2E (not compared to a single universal sequence). Except one isolate in the fIR4 group, no additional details are provided subsequently for any of these outliners and these outliners were not studied further. This polymorphism presentation in the first figure in the manuscript is thus confusing with those polymorphisms shown in Fig2E and its purpose for the overall manuscript is not clear.

Considering the multiple environmental signal inputs can influence the expression of fHbp, the growth conditions, i.e. temperature, iron and oxygen levels, should be described in more details for sample preparations used for all FHbp quantifications.

Analysis of fIR11 and fIR13 should be included in Fig. 2.

Why are the *cbb*A transcriptional levels varied in different fIR (Fig. 2B) located downstream? Are there any polymorphisms within the *cbb*A promoter region that segregate according to each fIR? Does the recombination of the various fIR constructs in locus somehow alter *cbb*A expression?

Is there an explanation that the single SNP in the -35 box of the outlier strain M08-0240104 yields 10 fold higher activity and this is only found in 2 out of >1000 strains? It appears that there is a selection pressure against such a SNP associated with the highest fHbp expression (Fig. 3B).

With an identical -35 element, the less optimal -10 elements (TACCGC) in fIR 1 showed a similar RNA level as fIR7 (TACCAT). Both fIR1 and fIR20 have identical promoter elements and only 1 SNP at +170 of the thermosensor region, yet these two fIRs yielded significantly different expression patterns. The data appears to suggest that the read through from cbbA plays an outweighed role in the total fHbp RNA level, yet the cbbA expression are not very different among these. It would be prudent to consider the cbbA regulation in various fIR group.

Is there a statistically significant difference between fIR3 and fIR6? What would be the possible explanation that 2 SNPs within the RNA thermosensor sequence that is downstream of +1 site affect transcription of fIR6 vs fIR3 as detected by qRT-PCR (Fig. 2D)? The RNA thermosensor presumably only influences translational efficiency.

While the protein levels of fIR6 showed thermoregulation between 30oC and 42oC, the SRM-MS measurement in Fig. 1C was presumably performed at 37oC for all samples, is there a difference in RNA structure and -ΔG between fIR3 and fIR6 at 37oC?

Please provide the temp of growth condition for data in Fig 3B, and describe whether the RNA thermosensor is in play for the different protein levels.

Data for thermoregulation is shown in Fig. 5, not Fig 4. Providing the -ΔG values for each RNA secondary structures in this figure will aid in the interpretation of the data.

There appears to be no differences in the protein levels of fIR13, 4 and 20 in Fig. 6A and no differences between fIR13, 15, 4 and 20 in Fig. 6B. Why do these separate into difference groups? There is hardly any difference between medium low and low groups.

Why would the survival be different between fIR1 and 7 in Fig. 6D if these two fIRs express similar levels of FHbp as shown in Fig 6A and 6B?

Line 345, based on the data presented in Fig. 2D and Fig 6B, the statement that fIR1 strain expresses the highest amount of fHbp is incorrect and thus could not be the reason that fIR1 strain survived better than all other strains.

The Y scale in Fig 1C does not appear to be linear scale, please clarify.

Fig. 6D legend, the final concentration of serum is 57%, not 60%, according to the description of procedure.

The discussion can be shortened and made less speculative (lines 484-486 as an example).
