# Supplementary material for: Deconvolution of intergenic polymorphisms determining high expression of Factor H binding protein in meningococcus and their association with invasive disease
Source: PLoS Pathog. 2021 Mar 26;17(3):e1009461. doi: 10.1371/journal.ppat.1009461 (PMC8026042; doi:10.1371/journal.ppat.1009461)
Supplement: Attachment — Submitted filename: PPATHOGENS-D-20-02160 reviews.docx [file ppat.1009461.s016.docx]

**Reviewer 3:**

The manuscript by Spinsanti et al is a detailed characterization of the intergenic region between *cbb*A and *fHbp* (designated fIR) mediating fHbp expression in *Neisseria meningitidis*. The authors defined 11 fIR sequence alleles, representing 91% of ~1100 meningococcal strains of UK circulating isolates. The authors also examined transcription by qRT-PCR, and protein levels by SRM-MS and Western blots. Further, polymorphisms within the Rho-independent terminator, -10 and -35 elements and its spacer as well as coding and the reported RNA thermosensor region were studied by site-specific mutagenesis using an isogenic MC58 strain background. The effects of fHbp peptide allele sequences on overall protein levels were also examined under the same inducible Ptac promoter control in MC58 by SRM-MS. The abundance of fHbp divided into four expression groups was correlated with susceptibility to killing by anti-fHbp antibodies and the ability to survive within human serum. The authors reported that strains with higher expressing fIR alleles and other cis factors contributing to high fHbp expression are more likely associated with invasive disease. The distributions of fIRs of >1100 strains were analyzed in terms of clonal complex, 3 fHbp protein variants, and fHbp peptide alleles.

This detailed comprehensive study is an extension of a previous 2016 study by Biagini et al from the same group. The previously defined 8 promoter clades are further refined to the 11 fIR sequence alleles. Many aspects of the *cis* regulation and a large collection of isolates were investigated to provide a better understanding of fHbp expression control, especially by the fIR sequence polymorphisms. While the data presented are quite comprehensive they do require greater clarity (see specific comment below). There is also another report by Cayrou et al. (ref #33) that applied analogous approaches to cluster the intergenic region to predict fHbp expression patterns in meningococci and a comparison of the conclusions from these two studies should be discussed.

Cayrou and colleagues used mRNA measurements in 80 strains expressing variant 1 to define five distinct expression groupings, and through statistical analyses they predict expression-controlling polymorphism combinations. Here we look at all fHbp variants and experimentally confirm the influence of each polymorphism we identify, including promoter, terminator, RNA thermosensor, and coding sequence differences.

We include in our tables the corresponding alleles for the fHbp and cbbA promoters as determined by Cayrou to facilitate comparison of the 2 studies.

We have added a comparison of the 2 studies to the discussion: Line 486 ‘Cayrou and colleagues recently reported that mRNA level analysis of *fHbp* from nearly 80 strains could cluster var1-expressing strains into five expression groups based on their intergenic region sequences [33]. Here, through analysis of nearly 6000 meningococcal isolates, we found a relatively small number of fIR alleles which commonly control fHbp expression (11 alleles out of 171 covering 88% isolates) including isolates of var2 and 3, and define major expression groups based on experimental data.

The use of the nucleotide sequence of the *fHbp* intergenic region as a predictive tool remains speculative. As the data generated show, fHbp regulation is complex, including the read-through from upstream *cbb*A expression, which has its own regulation profile, the presence or absence of RNA thermoregulation and the regulation of transcriptional regulators (FNR and FUR) and their interactions with fIR. While it follows that that increased levels of fHbp expression are associated with increased serum resistance and will be seen in invasive isolates, not clear that the small SNP changes in the fIR alone will provide a clear prediction *a priori* on overall fHbp production of a particular isolate within a diverse meningococcal population.

We agree that the regulation is highly complex and the use of sequence-based prediction is still limited due to the huge and ever growing diversity in sequence of the fHbp locus however we believe that the prediction into 4 main groupings of this study is based on experimentally confirmed differences in levels of the 11 fIR sequences together with higher levels in Variant 1 protein levels determined here.

We added a comment in the text:

Line 582: “The regulation of fHbp expression is highly complex and in this study we have limited the use of sequence-based prediction to those fIR for which we have experimental data. The sequence alone can ultimately be informative on the likelihood of the strain to progress to IMD or to be killed by anti-fHbp antibodies that may be induced through vaccination; however, due to the diversity in sequence of the *fHbp* locus there may be some strains for which no precise information through the sequence can be inferred.”

Specific comments:

1. The polymorphisms depicted in Fig 1D are from the outliners plotted in 1C and are specific to individual representative allele in each group in Fig 2E (not compared to a single universal sequence). Except one isolate in the fIR4 group, no additional details are provided subsequently for any of these outliners and these outliners were not studied further. This polymorphism presentation in the first figure in the manuscript is thus confusing with those polymorphisms shown in Fig2E and its purpose for the overall manuscript is not clear.

A suggested we have revised Figure 1 reducing it to 2 panels, 1D is now included in S1 supplementary and 1A is incorporated into 2A. We added information on further outliers in the text.

Line 190: “In clade II the strain ISS-2033 exhibiting low expression harbors a SNP within the Rho-independent terminator region with respect to the high expressing fIR1 alleles within the clade and in clade VI a number of outlier higher expressors exhibit SNPs again in the terminator region with respect to the fIR20 containing strains, which have all expression below the lower limit of quantification.”

2. Considering the multiple environmental signal inputs can influence the expression of fHbp, the growth conditions, i.e. temperature, iron and oxygen levels, should be described in more details for sample preparations used for all FHbp quantifications.

Experimental conditions for each growth of the isolates are specified in the Materials and Methods and added to the figure legends.

3. Analysis of fIR11 and fIR13 should be included in Fig. 2.

These fIR were not in the original 105 data set and experimental results measuring expression are included in Figure 6 and in the text.

4. Why are the *cbb*A transcriptional levels varied in different fIR (Fig. 2B) located downstream? Are there any polymorphisms within the *cbb*A promoter region that segregate according to each fIR? Does the recombination of the various fIR constructs in locus somehow alter *cbb*A expression?

We repeated the analysis adding a second housekeeping gene (*adk*) and the differences in *cbbA* within the new analysis do not result to be statistically significant. The *cbbA* promoter and gene sequence are identical in these strains as the recombination occurs downstream; however, there remains a slight increase in the level in the fIR1 isogenic strain, these are not statistically significant.

The text has been changed accordingly, Line 211 “mRNA levels of *cbbA* were less variable than the *fHbp* transcript levels between the isogenic strains (Figure 2B vs Figure 2D) and, as expected, no statistical differences between mutants were measured.”

5. Is there an explanation that the single SNP in the -35 box of the outlier strain M08-0240104 yields 10 fold higher activity and this is only found in 2 out of >1000 strains? It appears that there is a selection pressure against such a SNP associated with the highest fHbp expression (Fig. 3B).

We have added an explanation to the test Line 432: “This mutation in the -35 box may render the promoter less dependent on FNR activation and therefore with higher transcriptional initiation under aerobic conditions, resulting in the increases measured.”

6. With an identical -35 element, the less optimal -10 elements (TACCGC) in fIR 1 showed a similar RNA level as fIR7 (TACCAT). Both fIR1 and fIR20 have identical promoter elements and only 1 SNP at +170 of the thermosensor region, yet these two fIRs yielded significantly different expression patterns. The data appears to suggest that the read through from cbbA plays an outweighed role in the total fHbp RNA level, yet the cbbA expression are not very different among these. It would be prudent to consider the cbbA regulation in various fIR group.

We agree that the expression from the *cbbA* promoter is dominating the expression in the strains with the bicistronic transcript.

We have added a discussion on the *cbbA* promoter contribution to the discussion section:

Line 449 “Cayrou and colleagues investigated polymorphisms in the upstream *cbbA* promoter and observed only one allele, allele 6, with significantly lower expression. This is in line with previous observations that the expression of *cbbA* in 41 distinct strains was largely stable, when compared to the magnitude of diverse fHbp expression levels. In addition, strains where fHbp may be expressed also from the *cbbA* promoter through read-through due to fIR 1, 7 or 16 alleles were found not associated with the *cbbA* promoter allele 6 (Table 1 and Figure S7) and carry alleles that have been reported to have similar expression levels; therefore, the distinct fHbp expression levels are likely driven through fIR intergenic polymorphisms alone.”

7. Is there a statistically significant difference between fIR3 and fIR6? What would be the possible explanation that 2 SNPs within the RNA thermosensor sequence that is downstream of +1 site affect transcription of fIR6 vs fIR3 as detected by qRT-PCR (Fig. 2D)? The RNA thermosensor presumably only influences translational efficiency.

Yes, the difference between fIR3 and fIR6 is statistically significant. Translational efficiency and access to the RBS can effect transcriptional steady state levels: We have added this to the text

Line 469 “we propose that alternate structures resulting from polymorphisms in this region may also occlude the RBS driving decreased steady state levels and differential expression levels, similar to what has been described by Bhattacharyya and colleagues.”

While the protein levels of fIR6 showed thermoregulation between 30oC and 42oC, the SRM-MS measurement in Fig. 1C was presumably performed at 37oC for all samples, is there a difference in RNA structure and -ΔG between fIR3 and fIR6 at 37oC?

SRM-MS was performed on samples grown at 37°C. At this temperature the RNA thermosensor of fIR3 (ΔG = -6.30 kcal/mol) has the same structure as the one at 42°C (Fig4A, right panel) and the thermosensor of fIR6 (ΔG = -4.80 kcal/mol) has the same structure as the one at 42°C (Fig4B, right panel)

Please provide the temp of growth condition for data in Fig 3B, and describe whether the RNA thermosensor is in play for the different protein levels.

Growth conditions in which samples were generated have been added to the figure legend. In generating those mutants in the isogenic background each of these have identical RNA thermosensors as these are that of the MC58 strain.

Data for thermoregulation is shown in Fig. 5, not Fig 4. Providing the -ΔG values for each RNA secondary structures in this figure will aid in the interpretation of the data.

We altered figure numbers and we added ΔG values

There appears to be no differences in the protein levels of fIR13, 4 and 20 in Fig. 6A and no differences between fIR13, 15, 4 and 20 in Fig. 6B. Why do these separate into difference groups? There is hardly any difference between medium low and low groups.

We have modified this into 3 expression levels and have now grouped together fIR alleles 2, 3, 13, 15, 4 and 20 into the same low expressor group. The further segregation of the low expressing group into variant 1 and variant 2/3 which should have distinct levels from the data reported here have been explained in the text.

Why would the survival be different between fIR1 and 7 in Fig. 6D if these two fIRs express similar levels of FHbp as shown in Fig 6A and 6B?

We observed greater differences for distinct fIR in the experiment of survival in the presences of human serum. We have already discussed reasons for this in the text

Line 363 “These results could suggest 1) a possible regulation of *fHbp* in the presence of human serum for at least some of the fIR alleles under investigation or alternatively 2) that other additional factors may be responsible for the serum resistance in these strains outside of the *fHbp* locus and this was not investigated further.”

Line 345, based on the data presented in Fig. 2D and Fig 6B, the statement that fIR1 strain expresses the highest amount of fHbp is incorrect and thus could not be the reason that fIR1 strain survived better than all other strains.

Correct. fIR1 expresses the same amount of fHbp as fIR7. We removed this part to reduce the length of the results.

The Y scale in Fig 1C does not appear to be linear scale, please clarify.

Yes, the scale of the Y axis in Fig1C (now Fig 1B) is in logarithmic scale. This information has been added to the figure legend.

Fig. 6D legend, the final concentration of serum is 57%, not 60%, according to the description of procedure.

Correct. We have changed the final concentration of serum to 57% in the figure legend.

The discussion can be shortened and made less speculative (lines 484-486 as an example).

We have generally shortened the results and discussion. We have changed these lines in the discussion:

Line 520: “It would be interesting to investigate the presence of any combination of polymorphisms in the *CFH* region of the host and polymorphisms in the intergenic region of *fHbp* and whether this might somehow be one of the causes for the development of IMD rather than carriage.”

***********************


Reviewer's Responses to Questions

**Part I - Summary**
Please use this section to discuss strengths/weaknesses of study, novelty/significance, general execution and scholarship.

Reviewer #1: (No Response)

Reviewer #2: The manuscript "Deconvolution of intergenic polymorphisms determining high expression of Factor H binding protein in meningococcus and their association with invasive disease" by Spinsanti et al. is a molecular and epidemiological study on the production level of Factor H binding protein and their association to reported meningococcal invasive cases.
The overall principle and idea of the work is good, however, the authors did not show convincing data to support on the mechanism of FHbp production, the epidemiological section is unsupported and the conclusion in association to invasive cases is too bold and not supported.

We have significantly revised the epidemiological section and presented stronger new analyses, see below for details.

Reviewer #3: The manuscript entitled “Deconvolution of intergenic polymorphisms determining high expression of Factor H binding protein in meningococcus and their association with invasive disease” by Spinsanti et al. extends previous studies of the promotor region of FHbp. The authors identify fHbp intergenic regions (fIRs) that were previously referred to as “clades” by Biagini et al. (PNAS, 2016, 113(10): 2714). The authors perform a detailed analysis of the major allele sequences within each fIR and correlate fHbp expression levels (grouped into 4 categories) with the prevalence of the corresponding fIR among carriage and disease isolates from the U.K. The experimental approaches are sound, however the organization and presentation of the manuscript could be improved.

We have addressed the comments on the organization of the manuscript and have shortened both the results and discussion considerably to improve the presentation.

**Part II – Major Issues: Key Experiments Required for Acceptance**
Please use this section to detail the key new experiments or modifications of existing experiments that should be absolutely required to validate study conclusions.

Generally, there should be no more than 3 such required experiments or major modifications for a "Major Revision" recommendation. If more than 3 experiments are necessary to validate the study conclusions, then you are encouraged to recommend "Reject".

Reviewer #1: (No Response)

Reviewer #2: Major issues.
1) As the authors mentioned and shown in figure 1A, the expression of fHbp is dependent on a bicistronic and monocistronic transcript. While it is interesting to speculate polymorphisms within the intergenic region between the first gene cbbA and fHbp contribute to the production of FHbp, the authors have neglected to investigate the polymorphism within the promoter region of *cbbA*. Work by Oriente et al., and Delaney in J. Bact (2010) has previously shown that the major expression of fHbp and the production of FHbp originate from the bicistronic mRNA transcript. With cbbA (nmb1869) deleted, the expression of fHbp is down to perhaps 10%.
Next, the authors identified several polymorphisms within the cbbA-fHbp intergenic region and grouped them into different classes (fIR). The authors then generated isogenic mutants in a WT cbbA promoter with these fIRs and showed that the FHbp were expressed differently. It is unclear whether the promoter region of cbbA would have massive affect the expression of fHbp. To confirm this, the authors need to show polymorphisms within the cbbA promoter regions to the basal expression of fHbp and their association to the polymorphisms within the fIRs. Example, if there are several known polymorphisms within the promoter of cbbA, and the fIR is in WT configuration, what is the basal level of FHbp production? Are the specific polymorphisms in the promoter of cbbA associated to the fIR polymorphisms? The best way for authors to show this is to have the fIRs in a cbbA mutant background used by them in Oriente et al. J. Bact (2010).

In our previous work, Oriente *et al*., 2010 the expression of *cbbA* in 41 distinct strains was largely stable, when compared to the magnitude of diverse fHbp expression levels. Cayrou observed only one *cbbA* promoter with significantly lower expression (allele 6), however strains where fHbp may be expressed also from the *cbbA* promoter through read-through due to fIR 1, 7 or 16 alleles are not associated with the *cbbA* allele 6 and carry alleles 2, 4 and 5 which all have been reported to have similar expression levels (Cayrou et al,. 2018). We have added another supplementary figure (Fig S7) showing these association.

In addition, the experiment that the reviewer suggests, testing expression from fIR in a *cbbA* mutant, would not take into account the expression levels from read-through.

We have added a discussion on the contribution of the *cbbA* promoter this to the discussion section:

Line 449 “Cayrou and colleagues investigated polymorphisms in the upstream *cbbA* promoter and observed only one allele, allele 6, with significantly lower expression. This is in line with previous observations that the expression of *cbbA* in 41 distinct strains was largely stable, when compared to the magnitude of diverse fHbp expression levels. In addition, strains where fHbp may be expressed also from the *cbbA* promoter through read-through due to fIR 1, 7 or 16 alleles were found not associated with the *cbbA* promoter allele 6 (Table 1 and Figure S7) and carry alleles that have been reported to have similar expression levels; therefore, the distinct fHbp expression levels are likely driven through fIR intergenic polymorphisms alone.”

2) Figure 2B,C,D. qRT-PCR on the RNA transcript levels are not convincing and there was only 1 loading control using 16S RNA (minimum of 2 loading controls are required). The authors should perform Northern blots instead to show that the effect seen on FHbp is not due to stability of the bicistronic mRNA.

We have rerun the qRT-PCR in the paper using the *adk* housekeeping gene as another comparator control. We have substituted all figures to include the new data.

We do not agree that the Northern blots are necessary. They would show steady state levels at the moment of RNA preparation of the 2 transcripts (similar to the comparative qRT-PCR analyses included), as has been performed in Oriente et al., 2010, but not the stability of the transcripts in the absence of rifampycin testing.

3) What is the loading control in Figure 3B? It was neither mentioned in the Figure legend nor in the material and methods. The signal for this loading control is extremely weak. Please repeat this blot and consider showing a stronger signal loading control (perhaps CbbA). Is this Western blot repeated or was it just performed one time?

The loading control used in Figure 3B was an aspecific band with different exposure time.

We have repeated the Western blot using the protein YraP (NMB2091) as loading control. The Wb has been repeated with three biological replicates as stated in the legend. We also loaded the samples according to the description in the text. We have replaced this figure with a figure with the new analysis.

4) Figure 6A is fully not acceptable even though it is used to show an example level of FHbp which was later quantified in 6B. The author should never crop western blot bands and place them in boxes next to each other. The authors should consider repeating the gel and load them in the proper sequential manner to convince the reader. There are also no loading controls, how do the authors know the various fIRs were not loaded differently to show the production of FHbp? It is also not clear what the quantification of the band intensities was ratio to in Fig 6B.

We have repeated the Western blots and loaded the samples to avoid cropping. We used the protein YraP (NMB2091) as loading control and performed quantification of band intensities. fHbp values were normalized to YraP values. The figure has been replaced.

5) Figure 6C and D. It is unclear human plasma from volunteer donors and the human serum (Sigma) has been verified to contain meningococcal FHbp antibody. The authors should perform Western blots using FHbp variant antibodies on the serum to show that the serum used in Fig6D, does not harbour specific antibody/antibodies towards FHbp different variants. Otherwise, the serum survival results can be skewed and is unreliable. I.e. how many of the subjects had prior vaccination against Bexsero or recent colonisation/infection of meningococci? The author should also raise this issue in the discussion.

We have attempted to clarify better the use of these reagents. The human plasma from volunteer donors from the clinical trial was used for the hSBA assay as human complement source and in the experiments with the MC58 background strain expressing variant 1.1, in the absence of specific antibody (anti-fHbp variant 1.1 antiserum) did not kill the strain. This is now clarified in the text:

Line 681 “The final assay mixture contained 25% human plasma and serial dilutions of mouse anti-fHbp variant 1.1 antiserum”

The human serum from Sigma is a pool of normal human serum from anonymous donors. This is explained in the methods section. The same pool of human serum was used to test generic serum resistance of all the isogenic strains and all strains express to different levels the same variant 1.1 protein. We did not verify the presence of anti-fHbp antibodies as the KO mutant is fully sensitive to the serum while strains expressing more fHbp are resistant and all strains express the same variant. We do concede that these experiments are not as linear as the SBA killing experiments, and have discussed this in the text.

Line 363 “These results could suggest 1) a possible regulation of *fHbp* in the presence of human serum for at least some of the fIR alleles under investigation or alternatively 2) that other additional factors may be responsible for the serum resistance in these strains outside of the *fHbp* locus and this was not investigated further”

6) Figure 7 (epidemiological part) claiming that fIRs with high, medium high to low production of FHbp is not convincing and difficult to understand. It is understandable that the authors correlate the higher production of FHbp would enable to the bacterium survival better in the host with immune evasion thus leading the manifestation of meningococcal invasive diseases. However, the epidemiological data do not add up.
There are several major points the authors need to investigate and raise prior to making such conclusions.

We have repeated the epidemiological analyses taking the same timeframe for the carriage and invasive strains and have explained better the grouping of the strains according the to experimentally derived data in the study.

A) Invasive isolates isolated from year 2010-2012 and carriers from year 1996-2015. There is a bias in this sampling due to several factors and a few big outbreaks can influence the variety of invasive isolates obtained from 2010-2012. There is an attempt to deal with this in the “Statistical analyses” however, in the methods used; it is unclear exactly how this bias is being dealt with. Creating 1000 datasets with random sampling from the invasive isolates will still contain isolates with a great dependency on possible outbreaks, making them still potentially very similar and biased – just in a 1000 very similar ways.

We thank the reviewer for highlighting this important point. We have repeated our analysis on a wider dataset including all the carriage and invasive isolates available in the PubMLST for UK until 2015, before the national immunization campaign with Bexsero. Accordingly, the results section and Figure 7 has been modified.

However, the imbalance in the dataset is still skewed towards invasiveness apart from the final years of 2014 and 2015 when the MenCar4 study on meningococcal carrier strains, was performed and more carriage strains were sequenced and we know that, typically, the diversity is greater in the carriage compared to the invasive strains.

In order to address the comment from the review and test whether the bias could affect the conclusions, we also decided to run the analysis on a smaller set concentrated in years 2014/15, when the MenCar4 study on meningococcal carrier strains, was performed. During this timeframe (from personal communication), we know that in the dataset, for England, Wales and Northern Ireland, we have 10 outbreaks, for a total of 22 strains. Below, we report for the reviewers’ interest the result of the regression performed on this smaller dataset. Since the number of carrier strains was higher compared to invasive strains (1583 and 1099, respectively), we used the same randomization method described in the paper to obtain 1000 subsets with a 50:50 proportion. In this case, the sampling was conducted in the carrier group, to have the same number compared to invasive strains. Even if the variability observed in the regressions is bigger than the one reported in the paper, due to smaller numbers, the trend of the regression is the same. For this reason, we are confident that the observations are confirming our hypothesis, while remaining difficult to dissect the fHbp and its fIR from the genetic background of the strains.

B) Among these 1100 strains (should be renamed to isolates and not strains) from the UK, how many of them are of various capsule group/CC types also have effect on the fIRs of association to invasive cases? The supplementary figure showing number of isolates of different serogroups and CC is not informative.

We have modified Supplementary Figure S3 in order to address this comment and in addition within each of the supplementary figures reporting correlations of fIR with variants, peptides, STs we present the data segregated into invasive and carriage strains. New figures have been substituted for each.

C) The majority of the isolates are of sergroup B (under capsule) and ST-41/44 (under CC) and if one looks into the supplementary data, ST-41/44contained up to 40% of fIR2 (medium low FHbp production), how is this even relate to the high association with invasive cases? How many isolates of serogroup B and are of ST-41/44 too?

As we have extended the analysis to almost 6000 strains we have inserted a new figure S3 describing these with respect to capsule and particularly describing the ST in the invasive or carriage groups per year. For this new data set there are a we also include in Fig S6 a description of the invasive and carriage strains within the major clonal complexes and the fIRs associated with each of these.

Within this new dataset we attempt to address this reviewers comment, we report a table with the absolute numbers of isolates from the ST41/44 for each expression group dividing them in invasive and carriage. The total number of strains in the high and medium groups are low in this dataset but the percentage is clearly in favor of the invasiveness. On the other hand, in the low group, the fHbp peptide variant plays an important role. In fact, we see that variant 1, that from our results tends to have higher expression levels, is more associated with the invasive phenotype, variants 2 and 3, that are generally less expressed, have a higher carrier percentage. By comparing the proportions of carrier and invasive isolates in the high and Low var2/3 groups with the test on difference of proportions, we obtain a *p*-value of 1.226e-05 for their difference.

| **ST-41/44 complex** | **High** | **Medium** | **Low - var1** | **Low – var2/3** |
| --- | --- | --- | --- | --- |
| **carrier** | 0 | 2 | 31 | 109 |
| **invasive** | 11 | 78 | 386 | 75 |

Furthermore, the new supplementary Figure 4S panel B, shows that, even variants 2 and 3, when in presence of high expression, are associated with invasive

D) ST-11, serogroup W is a rising “clade” of hypervirulent strains responsible for a big part of meningococcal infections, although as presented in this data is one of the lowest (fIR4) production of fHbp – Data shows that the invasiveness properties of fIR variants seems to be more attributed to specific ccs such as cc32 and cc269. Please investigate and report

We have substituted the supplementary figure 6 including a subpanel B in which we report for each clonal complex the different fIR found. While there are numerous isolates of the ST-11 complex strains carrying fIR4 that can be both carriage and invasive, there is a higher proportion of high or medium expressing fir alleles such as fIR1 and fIR11 alleles associated with the invasive group in the ST-11 clade as shown in the new figure Fig S6B.

We have added a comment to the text on this finding:

Line 392: “Moreover, for each of the clonal complexes ST-11, ST-41/44, ST-269, ST-32, and ST-213, it is clear that there is a larger proportion of higher expressing fIRs associated with the invasive rather than carriage isolates (Figure S6B).”

In fact, we do agree that invasive properties cannot be solely attributed to fHbp expression levels and have added this to the discussion:

Line 496 “Nevertheless, even though we cannot predict the disease status solely on the inferred fHbp expression levels, we detected an augmented proportion of isolates with predicted high fHbp expression in IMD isolates.”

E) Why is ST-22 not shown with the distribution of fIR?

This is shown in Fig S6 Panel A.

F) Under the statistical analyses, the authors used several packages such as pR2 and vcd R. Please elaborate on what they do to the dataset.

We have added a better explanation, in the Methods section, on the rational for using those packages in the statistical analysis.

Reviewer #3: 1. It is not clear how exactly what criteria were used to define the four classes of expression were determined. The text on page 14, line 329, indicates protein expression (in isogenic mutant strains) and the data in Figure 6 show the clearest distinction in susceptibility to bactericidal activity of the same strains to anti-fHbp antiserum.

Within our new analysis of 6000 strains the four groupings were based on the experimental data that are reported in this study, firstly on 3 different expression level groups and taking into account within the Low expression group, containing the largest number of isolates, those expressing variant 1, which are predicted to express higher that the variant 2/3 isolates. We have clarified this in the text:

Line 395 “Finally, using the fIR-based analysis and taking into account the greater expression of var1 when compared to var2/3 strains, we divided this panel of strains into four groups, namely high (fIR1 and 7, 7.7%), medium (fIR6, 11 and 16, 8.2%) and two low groups segregated based on the variant they express, low var1 (fIR2, 3 and 13, 27.7%), and low var2/3 (fIR4, 15 and 20, 56.5%).”

2. Figure 7. It would be helpful to indicate how many strains used in the analysis were in each expression group (and within each expression group, N for each carriage and invasive). Also, the logistic regression suggests that more disease is caused by isolates with medium high expression whereas the data show that the opposite is true. Could this an artifact of a small sample size for some of the expression groups or trying to fit data for which there might be many other variables (strain clonal complex, fHbp sequence variant, etc.)?

We have now repeated the analysis with a much larger group of isolates (nearly 6000). We have added the total number of isolates in the distinct groups to figure 7 and have included a new Table S7 reporting the numbers of carriage versus invasive groupings. However we do concede that it is difficult to dissect entirely the single role of fIR, fHbp peptide, variant and clonal complex, due to their strong association (Supplementary Figures S4-S6).

|  | High | Medium | Low - var1 | Low - var2/3 |
| --- | --- | --- | --- | --- |
| carrier | 43 | 45 | 567 | 1484 |
| invasive | 349 | 372 | 850 | 1406 |

**Part III – Minor Issues: Editorial and Data Presentation Modifications**
Please use this section for editorial suggestions as well as relatively minor modifications of existing data that would enhance clarity.

Reviewer #1: (No Response)

**Reviewer #2:** Minor issues
1) Figure 4 and 5 are wrongly labelled

These have been relabeled.
2) Please use unit for the Delta G values (Lines 227, 228)

We have added the units
3) Line 242. Authors claim that the ribosome binding site (AGGAG) would affect translatability. Please show the result otherwise the tone down the claim.

We have removed this claim.

Reviewer #3: 1. Figures. Figure 1B and C contain significant overlap with Biagini et al. (data replotted with a different nomenclature). Fig. 1A is not very helpful and duplicates with Figure 2A. Figs 1A and D could be combined to show the promotor elements and the polymorphisms could be indicated, as they already are, in Fig. 2E

We have altered figure 1, combining Fig1A with Fig2A and putting Figure 1D in supplementary.

2. Organization of text. First paragraph of Results belongs elsewhere (Introduction).

This paragraph has been moved to the introduction accordingly

Paragraph beginning on page 10, line 212 should be moved to the Discussion section as it is somewhat speculative. Elsewhere, there excess discussion with Results sections. Overall, text in Results and Discussion is lengthy.

We have attempted to reduce the results sections removing the discussive sections and leaving only the descriptive text.

3. Page 11, line 249. Regarding the weak vs. strong terminator hypothesis, the text says “sequence of wild-type fIR intergenic region was modified with the substitution of a single nucleotide within the stem region.” Figure 3 seems to indicate that this substitution was in the background of the -10 box substitutions, not the WT background.

We have altered the text to make this clearer:

Line 259: “The sequence of the wild-type fIR7 intergenic region was modified with the substitution of a single nucleotide within the stem region of the terminator sufficient to restore a correct base pairing in the stem (position 46 of the alignment, from A to G), increasing its strength from ΔG = -14.8 to -27.3 kcal/mol, for testing of the ‘weak’ and ‘strong’ terminator hypothesis (c*fHbp* and c*fHbp* term, respectively).”

Also, line 257, description of Figure 3: this section would be more clear if the text were written to follow the sequence in the Figure or vice versa.

The substituted figure now consists of a Western Blot with samples in the order of presentation in the text

4. The abstract mentions a correlation between FHbp expression and survival. This could be assessed directly by plotting the data in Figure 6C versus the data in 6B. Have the authors performed this analysis and is there a significant correlation?

We have added the Pearson correlation coefficient and relative *p*-value in the text.

Line 350 “Furthermore, by correlating the SBA titers with fHbp expression levels we determined a Pearson correlation coefficient of 0.96 (*p*-value: 0.0002).”

5. Figures 4 and 5. Order of Figures does not match text.

This has been altered.
